# Supplementary material for: Platelet-Rich plasma Injection Management for Ankle osteoarthritis study (PRIMA): protocol of a Dutch multicentre, stratified, block-randomised, double-blind, placebo-controlled trial
Source: BMJ Open. 2019 Oct 7;9(10):e030961. doi: 10.1136/bmjopen-2019-030961 (PMC6797250; doi:10.1136/bmjopen-2019-030961)
Supplement: Supplementary data [file bmjopen-2019-030961supp001.pdf]

Surveys of PRIMA trial - version 83.01

Printed on 16-09-2019 11:34:13 by Liam Paget

Survey 'Vragenlijst PRIMA studie'

Vragenlijst PRIMA studie - AOFAS - Pijn

Number	Question	Answers
1.1	Hoeveel pijn ervaart u aan uw voet??	☐ Geen<br>☐ Milde pijnklachten of af en toe pijn<br>☐ Matige pijnklachten, dagelijks<br>☐ Vrijwel continu erge pijn

Vragenlijst PRIMA studie - AOFAS resterende gedeelte

Number	Question	Answers
2.1	Hoeveel beperkingen in het dagelijks leven ervaart u?	◯ Geen beperkingen, geen hulpmiddelen nodig<br>◯ Geen beperkingen in algemeen dagelijkse bezigheden, wel beperkingen in recreatieve activiteiten (sport, hobbies, etc) geen ondersteuning<br>◯ Beperkingen bij dagelijkse- en recreatieve activiteiten, stok<br>◯ Ernstige beperkingen bij dagelijkse- en recreatieactiviteiten, walker, krukken, rolstoel, brace
2.2	Wat is de afstand die u in één keer kunt lopen?	◯ Meer dan 600 meter<br>◯ 400 - 600 meter<br>◯ 100 - 300 meter<br>◯ Minder dan 100 meter
2.3	Heeft u moeite met lopen op een bepaalde ondergrond?	◯ Geen problemen bij lopen<br>◯ Enig problemen op ongelijke ondergrond, trap, helling, ladders<br>◯ Ernstige problemen bij ongelijke ondergrond, trap, helling, ladders

Paget LDA, *et al.* *BMJ Open* 2019; 9:e030961. doi: 10.1136/bmjopen-2019-030961

2.4	Hoe zou u uw looppatroon beoordelen?	◯ Normaal of minimaal afwijkend<br>◯ Duidelijk<br>◯ Ernstig
2.5	Hoe zou u de beweeglijkheid van uw voet beoordelen ten opzicht van de andere zijde of ten opzicte van vóór het ongeval?	◯ Hetzelfde of licht beperkt t.o.v. de niet aangedane zijde of zoals voor het ongeval<br>◯ Ongeveer de helft beperkt<br>◯ Sterk verminderd, bijna geen bewegingen mogelijk
2.6	Hoe zou u de beweeglijkheid van uw enkel beoordelen ten opzichte van de andere zijde of ten opzichte van voor het ongeval?	◯ Hetzelfde of licht beperkt t.o.v. de niet aangedane zijde of zoals voor het ongeval<br>◯ Ongeveer de helft beperkt<br>◯ Sterk verminderd, bijna geen bewegingen mogelijk
2.7	Hoe zou u de stabiliteit van uw voet en enkel beoordelen	◯ Stabiel, ik verzwik mijn enkel hooguit incidenteel<br>◯ Niet stabiel, ik verzwik mijn enkel vaak
2.8	Hoe zou u de stand van uw voet beoordelen ten opzichte van de andere zijde of ten opzichte van voor het ongeval?	◯ Goed, ik kan mijn voet goed plat op de grond zetten, waarbij de stand niet afwijkend is<br>◯ Ik sta vrijwel alleen op de buitenzijde van mijn voet<br>◯ Ik kan mijn voet niet plat op de grond zetten en loop vrijwel alleen op mijn voorvoet

Vragenlijst PRIMA studie - VAS Pain score

Number	Question	Answers	
3.1	VAS pijnscore (Geef uw minimaal pijnscore aan tijdens dagelijkse activiteiten op een schaal van 0 tot 100, waarbij 0 geen pijn is en 100 de ergste pijn denkbaar is)	(0.00)	100 (100.00)

Vragenlijst PRIMA studie - AAS

Paget LDA, *et al. BMJ Open* 2019; 9:e030961. doi: 10.1136/bmjopen-2019-030961

Number	Question	Answers

Paget LDA, *et al. BMJ Open* 2019; 9:e030961. doi: 10.1136/bmjopen-2019-030961

4.1	Wat is het meest bij u van toepassing ten aanzien van uw acitviteit?	☐ American Football<br>☐ Basketbal<br>☐ Gymnastiek/turnen<br>☐ Handbal<br>☐ Rugby<br>☐ Voetbal<br>☐ Hockey<br>☐ Korfbal<br>☐ Gevechtssporten: judo, karate, kung fu, taekwando, jiu jitsu, etc.<br>☐ Oriëntatielopen<br>☐ Rhytmische gymnastiek<br>☐ Volleybal<br>☐ Boxen<br>☐ Freestyle snowboarden<br>☐ Ijshockey<br>☐ Tennis<br>☐ Worstelen<br>☐ Fitness, aerobics<br>☐ Badminton<br>☐ Baseball<br>☐ Cross-country hardlopen<br>☐ Moderne pentathlon<br>☐ Squash<br>☐ Surfen, windsurfen<br>☐ Tafel tennis<br>☐ Atletiek: spring-, werponderdelen<br>☐ Waterskieën<br>☐ Dans<br>☐ Schermen<br>☐ Zaalhockey<br>☐ Bergbeklimmen<br>☐ Langlauf<br>☐ Parachute springen<br>☐ Softball<br>☐ Speciale beroepen en werkactiviteiten; speciale beroepen: ballet, professioneel soldaat, speciale reddingswerker, stuntman, etc.<br>☐ Duiken<br>☐ Scubaduiken<br>☐ Skaten, in-line skaten<br>☐ Atletiek: looponderdelen<br>☐ Triatlon<br>☐ Gewichtsheffen, body-building

Paget LDA, *et al*. *BMJ Open* 2019; 9:e030961. doi: 10.1136/bmjopen-2019-030961

		☐ Alle competitieve sporten hieronder met 'seasonal' conditioning<br>☐ Zwaar fysiek werk<br>☐ Alpine skiën en snowboarden<br>☐ Bowlen/curlen<br>☐ Golf<br>☐ Mountainbike/BMX<br>☐ Powerliften<br>☐ Zeilen<br>☐ Fysiek werk<br>☐ Wielrennen<br>☐ Paardrijden<br>☐ Motorsporten/technische sporten<br>☐ Roeien, kayakken<br>☐ Boogschieten<br>☐ Water polo en zwemmen<br>☐ Kunnen lopen op oneven grond<br>☐ Geen sport, echter geen limitatie in dagelijkse activiteiten<br>☐ Kunnen lopen op even grond, maar de dagelijkste activiteiten zijn gelimiteerd<br>☐ Niet kunnen lopen, fysieke handicap wegens enkelproblemen
4.1.1	***If 'Wat is het meest bij u van toepassing ten aanzien van uw acitviteit?' is equal to 'American Football' answer this question:***<br>American Football	○ Professioneel<br>○ Competitief<br>○ Recreatief
4.1.2	***If 'Wat is het meest bij u van toepassing ten aanzien van uw acitviteit?' is equal to 'Basketbal' answer this question:***<br>Basketbal	○ Professioneel<br>○ Competitief<br>○ Recreatief
4.1.3	***If 'Wat is het meest bij u van toepassing ten aanzien van uw acitviteit?' is equal to 'Gymnastiek/turnen' answer this question:***<br>Gymnastiek/turnen	○ Professioneel<br>○ Competitief<br>○ Recreatief
4.1.4	***If 'Wat is het meest bij u van toepassing ten aanzien van uw acitviteit?' is equal to 'Handbal' answer this question:***<br>Handbal	○ Professioneel<br>○ Competitief<br>○ Recreatief
4.1.5	***If 'Wat is het meest bij u van toepassing ten aanzien van uw acitviteit?' is equal to 'Rugby' answer this question:***<br>Rugby	○ Professioneel<br>○ Competitief<br>○ Recreatief

Paget LDA, *et al. BMJ Open* 2019; 9:e030961. doi: 10.1136/bmjopen-2019-030961

4.1.6	***If 'Wat is het meest bij u van toepassing ten aanzien van uw acitviteit?' is equal to 'Voetbal' answer this question:***<br>Voetbal	◯ Professioneel<br>◯ Competitief<br>◯ Recreatief
4.1.7	***If 'Wat is het meest bij u van toepassing ten aanzien van uw acitviteit?' is equal to 'Hockey' answer this question:***<br>Hockey	◯ Professioneel<br>◯ Competitief<br>◯ Recreatief
4.1.8	***If 'Wat is het meest bij u van toepassing ten aanzien van uw acitviteit?' is equal to 'Korfbal' answer this question:***<br>Korfbal	◯ Professioneel<br>◯ Competitief<br>◯ Recreatief
4.1.9	***If 'Wat is het meest bij u van toepassing ten aanzien van uw acitviteit?' is equal to 'Gevechtssporten: judo, karate, kung fu, taekwando, jiu jitsu, etc.' answer this question:***<br>Gevechtssporten: judo, karate, kung fu, taekwando, jiu jitsu, etc.	◯ Professioneel<br>◯ Competitief<br>◯ Recreatief
4.1.10	***If 'Wat is het meest bij u van toepassing ten aanzien van uw acitviteit?' is equal to 'Oriëntatielopen' answer this question:***<br>Oriëntatielopen	◯ Professioneel<br>◯ Competitief<br>◯ Recreatief
4.1.11	***If 'Wat is het meest bij u van toepassing ten aanzien van uw acitviteit?' is equal to 'Rhytmische gymnastiek' answer this question:***<br>Rhytmisch gymnastiek	◯ Professioneel<br>◯ Competitief<br>◯ Recreatief
4.1.12	***If 'Wat is het meest bij u van toepassing ten aanzien van uw acitviteit?' is equal to 'Volleybal' answer this question:***<br>Volleybal	◯ Professioneel<br>◯ Competitief<br>◯ Recreatief
4.1.13	***If 'Wat is het meest bij u van toepassing ten aanzien van uw acitviteit?' is equal to 'Boxen' answer this question:***<br>Boxen	◯ Professioneel<br>◯ Competitief<br>◯ Recreatief
4.1.14	***If 'Wat is het meest bij u van toepassing ten aanzien van uw acitviteit?' is equal to 'Freestyle snowboarden' answer this question:***<br>Freestyle snowboarden	◯ Professioneel<br>◯ Competitief<br>◯ Recreatief
4.1.15	***If 'Wat is het meest bij u van toepassing ten aanzien van uw acitviteit?' is equal to 'Freestyle snowboarden' answer this question:***<br>IJshockey	◯ Professioneel<br>◯ Competitief<br>◯ Recreatief

Paget LDA, *et al*. *BMJ Open* 2019; 9:e030961. doi: 10.1136/bmjopen-2019-030961

4.1.16	***If 'Wat is het meest bij u van toepassing ten aanzien van uw acitviteit?' is equal to 'Tennis' answer this question:***<br>Tennis	◯ Professioneel<br>◯ Competitief<br>◯ Recreatief
4.1.17	***If 'Wat is het meest bij u van toepassing ten aanzien van uw acitviteit?' is equal to 'Worstelen' answer this question:***<br>Worstelen	◯ Professioneel<br>◯ Competitief<br>◯ Recreatief
4.1.18	***If 'Wat is het meest bij u van toepassing ten aanzien van uw acitviteit?' is equal to 'Fitness, aerobics' answer this question:***<br>Fitness, aerobics	◯ Professioneel<br>◯ Competitief<br>◯ Recreatief
4.1.19	***If 'Wat is het meest bij u van toepassing ten aanzien van uw acitviteit?' is equal to 'Badminton' answer this question:***<br>Badminton	◯ Professioneel<br>◯ Competitief<br>◯ Recreatief
4.1.20	***If 'Wat is het meest bij u van toepassing ten aanzien van uw acitviteit?' is equal to 'Baseball' answer this question:***<br>Baseball	◯ Professioneel<br>◯ Competitief<br>◯ Recreatief
4.1.21	***If 'Wat is het meest bij u van toepassing ten aanzien van uw acitviteit?' is equal to 'Cross-country hardlopen' answer this question:***<br>Cross-country hardlopen	◯ Professioneel<br>◯ Competitief<br>◯ Recreatief
4.1.22	***If 'Wat is het meest bij u van toepassing ten aanzien van uw acitviteit?' is equal to 'Moderne pentathlon' answer this question:***<br>Moderne pentathlon	◯ Professioneel<br>◯ Competitief<br>◯ Recreatief
4.1.23	***If 'Wat is het meest bij u van toepassing ten aanzien van uw acitviteit?' is equal to 'Squash' answer this question:***<br>Squash	◯ Professioneel<br>◯ Competitief<br>◯ Recreatief
4.1.24	***If 'Wat is het meest bij u van toepassing ten aanzien van uw acitviteit?' is equal to 'Surfen, windsurfen' answer this question:***<br>Surfen, windsurfen	◯ Professioneel<br>◯ Competitief<br>◯ Recreatief
4.1.25	***If 'Wat is het meest bij u van toepassing ten aanzien van uw acitviteit?' is equal to 'Tafel tennis' answer this question:***<br>Tafel tennis	◯ Professioneel<br>◯ Competitief<br>◯ Recreatief

Paget LDA, *et al*. *BMJ Open* 2019; 9:e030961. doi: 10.1136/bmjopen-2019-030961

4.1.26	***If 'Wat is het meest bij u van toepassing ten aanzien van uw acitviteit?' is equal to 'Atletiek: spring-, werponderdelen' answer this question:***<br>Atletiek: spring-, werponderdelen	○ Professioneel<br>○ Competitief<br>○ Recreatief
4.1.27	***If 'Wat is het meest bij u van toepassing ten aanzien van uw acitviteit?' is equal to 'Waterskieën' answer this question:***<br>Waterskien	○ Professioneel<br>○ Competitief<br>○ Recreatief
4.1.28	***If 'Wat is het meest bij u van toepassing ten aanzien van uw acitviteit?' is equal to 'Dans' answer this question:***<br>Dans	○ Professioneel<br>○ Competitief<br>○ Recreatief
4.1.29	***If 'Wat is het meest bij u van toepassing ten aanzien van uw acitviteit?' is equal to 'Schermen' answer this question:***<br>Schermen	○ Professioneel<br>○ Competitief<br>○ Recreatief
4.1.30	***If 'Wat is het meest bij u van toepassing ten aanzien van uw acitviteit?' is equal to 'Zaalhockey' answer this question:***<br>Zaalhockey	○ Professioneel<br>○ Competitief<br>○ Recreatief
4.1.31	***If 'Wat is het meest bij u van toepassing ten aanzien van uw acitviteit?' is equal to 'Bergbeklimmen' answer this question:***<br>Bergbeklimmen	○ Professioneel<br>○ Competitief<br>○ Recreatief
4.1.32	***If 'Wat is het meest bij u van toepassing ten aanzien van uw acitviteit?' is equal to 'Langlauf' answer this question:***<br>Langlauf	○ Professioneel<br>○ Competitief<br>○ Recreatief
4.1.33	***If 'Wat is het meest bij u van toepassing ten aanzien van uw acitviteit?' is equal to 'Parachute springen' answer this question:***<br>Parachute springen	○ Professioneel<br>○ Competitief<br>○ Recreatief
4.1.34	***If 'Wat is het meest bij u van toepassing ten aanzien van uw acitviteit?' is equal to 'Softball' answer this question:***<br>Softball	○ Professioneel<br>○ Competitief<br>○ Recreatief

Paget LDA, *et al. BMJ Open* 2019; 9:e030961. doi: 10.1136/bmjopen-2019-030961

4.1.35	***If 'Wat is het meest bij u van toepassing ten aanzien van uw acitviteit?' is equal to 'Speciale beroepen en werkactiviteiten; speciale beroepen: ballet, professioneel soldaat, speciale reddingswerker, stuntman, etc.' answer this question:***<br>Speciale beroepen en werkactiviteiten speciale beroepen: ballet, professioneel soldaat, speciale reddingswerker, stuntman, etc.	◯ ballet, professioneel soldaat, speciale reddingswerker, stuntman, etc.
4.1.36	***If 'Wat is het meest bij u van toepassing ten aanzien van uw acitviteit?' is equal to 'Duiken' answer this question:***<br>Duiken	◯ Professioneel/competitief<br>◯ Recreatief
4.1.37	***If 'Wat is het meest bij u van toepassing ten aanzien van uw acitviteit?' is equal to 'Scubaduiken' answer this question:***<br>Scubaduiken	◯ Professioneel/competitief<br>◯ Recreatief
4.1.38	***If 'Wat is het meest bij u van toepassing ten aanzien van uw acitviteit?' is equal to 'Skaten, in-line skaten' answer this question:***<br>skaten, in-linen skaten	◯ Professioneel/competitief<br>◯ Recreatief
4.1.39	***If 'Wat is het meest bij u van toepassing ten aanzien van uw acitviteit?' is equal to 'Atletiek: looponderdelen' answer this question:***<br>Atletiek: looponderdelen	◯ Professioneel/competitief<br>◯ Recreatief
4.1.40	***If 'Wat is het meest bij u van toepassing ten aanzien van uw acitviteit?' is equal to 'Triatlon' answer this question:***<br>triatlon	◯ Professioneel/competitief<br>◯ Recreatief
4.1.41	***If 'Wat is het meest bij u van toepassing ten aanzien van uw acitviteit?' is equal to 'Gewichtsheffen, body-building' answer this question:***<br>Gewichtsheffen, body-building	◯ Professioneel/competitief<br>◯ Recreatief
4.1.42	***If 'Wat is het meest bij u van toepassing ten aanzien van uw acitviteit?' is equal to 'Alle competitieve sporten hieronder met 'seasonal' conditioning' answer this question:***<br>Alle competitieve sporten hieronder met 'seasonal' conditioning	◯ Alle competitieve sporten hieronder met 'seasonal' conditioning
4.1.43	***If 'Wat is het meest bij u van toepassing ten aanzien van uw acitviteit?' is equal to 'Zwaar fysiek werk' answer this question:***<br>Zwaar fysiek werk	◯ Alle competitieve sporten hieronder met 'seasonal' conditioning

Paget LDA, *et al. BMJ Open* 2019; 9:e030961. doi: 10.1136/bmjopen-2019-030961

4.1.44	***If 'Wat is het meest bij u van toepassing ten aanzien van uw acitviteit?' is equal to 'Alpine skiën en snowboarden' answer this question:***<br>Alpine skien en snowboarden	◯ Alpine skien en snowboarden
4.1.45	***If 'Wat is het meest bij u van toepassing ten aanzien van uw acitviteit?' is equal to 'Bowlen/curlen' answer this question:***<br>Bowlen/curlen	◯ Bowlen/curlen
4.1.46	***If 'Wat is het meest bij u van toepassing ten aanzien van uw acitviteit?' is equal to 'Golf' answer this question:***<br>Golf	◯ Golf
4.1.47	***If 'Wat is het meest bij u van toepassing ten aanzien van uw acitviteit?' is equal to 'Mountainbike/BMX' answer this question:***<br>Mountainbike/BMX	◯ Mountainbike/BMX
4.1.48	***If 'Wat is het meest bij u van toepassing ten aanzien van uw acitviteit?' is equal to 'Powerliften' answer this question:***<br>Powerliften	◯ Powerliften
4.1.49	***If 'Wat is het meest bij u van toepassing ten aanzien van uw acitviteit?' is equal to 'Zeilen' answer this question:***<br>Zeilen	◯ Zeilen
4.1.50	***If 'Wat is het meest bij u van toepassing ten aanzien van uw acitviteit?' is equal to 'Fysiek werk' answer this question:***<br>Fysiek werk	◯ Fysiek werk
4.1.51	***If 'Wat is het meest bij u van toepassing ten aanzien van uw acitviteit?' is equal to 'Wielrennen' answer this question:***<br>Wielrennen	◯ Wielrennen
4.1.52	***If 'Wat is het meest bij u van toepassing ten aanzien van uw acitviteit?' is equal to 'Paardrijden' answer this question:***<br>Paardrijden	◯ Paardrijden
4.1.53	***If 'Wat is het meest bij u van toepassing ten aanzien van uw acitviteit?' is equal to 'Motorsporten/technische sporten' answer this question:***<br>Motorsporten/technische sporten	◯ Motorsporten/technische sporten
4.1.54	***If 'Wat is het meest bij u van toepassing ten aanzien van uw acitviteit?' is equal to 'Roeien, kayakken' answer this question:***<br>Roeien, kayakken	◯ Roeien, kayakken

Paget LDA, *et al. BMJ Open* 2019; 9:e030961. doi: 10.1136/bmjopen-2019-030961

4.1.55	***If 'Wat is het meest bij u van toepassing ten aanzien van uw acitviteit?' is equal to 'Boogschieten' answer this question:*** Boogschieten	◯ Boogschieten
4.1.56	***If 'Wat is het meest bij u van toepassing ten aanzien van uw acitviteit?' is equal to 'Water polo en zwemmen' answer this question:*** Water polo en zwemmen	◯ Water polo en zwemmen
4.1.57	***If 'Wat is het meest bij u van toepassing ten aanzien van uw acitviteit?' is equal to 'Kunnen lopen op oneven grond' answer this question:*** Kunnen lopen op oneven grond	◯ Kunnen lopen op oneven grond
4.1.58	***If 'Wat is het meest bij u van toepassing ten aanzien van uw acitviteit?' is equal to 'Geen sport, echter geen limitatie in dagelijkse activiteiten' answer this question:*** Geen sport, echter geen limitatie in dagelijkse activiteiten	◯ Geen sport, echter geen limitatie in dagelijkse activiteiten
4.1.59	***If 'Wat is het meest bij u van toepassing ten aanzien van uw acitviteit?' is equal to 'Kunnen lopen op even grond, maar de dagelijkste activiteiten zijn gelimiteerd' answer this question:*** Kunnen lopen op even grond, maar de dagelijkste activiteiten zijn gelimiteerd	◯ Kunnen lopen op even grond, maar de dagelijkste activiteiten zijn gelimiteerd
4.1.60	***If 'Wat is het meest bij u van toepassing ten aanzien van uw acitviteit?' is equal to 'Niet kunnen lopen, fysieke handicap wegens enkelproblemen' answer this question:*** Niet kunnen lopen, fysieke handicap wegens enkelproblemen	◯ Niet kunnen lopen, fysieke handicap wegens enkelproblemen

Vragenlijst PRIMA studie - Hoe tevreden bent u over de enkelklachten?

Number	Question	Answers
5.1	Hoe tevreden bent u over de enkelklachten?	◯ Slecht<br>◯ Matig<br>◯ Goed<br>◯ Uitstekend

Paget LDA, *et al. BMJ Open* 2019; 9:e030961. doi: 10.1136/bmjopen-2019-030961

Vragenlijst PRIMA studie - SF-36

Number	Question	Answers
	Deze vragenlijst gaat over uw standpunten t.a.v. uw gezondheid. Met behulp van deze gegevens kan worden bijgehouden hoe u zich voelt en hoe goed u in staat bent uw gebruikelijke bezigheden uit te voeren. Beantwoord elke vraag door een antwoord aan te klikken. Als u niet zeker weet hoe u een vraag moet beantwoorden, geef dan het best mogelijke antwoord.	
6.1	Hoe zou u over het algemeen uw gezondheid noemen?	◯ Uitstekend ◯ Zeer goed ◯ Goed ◯ Matig ◯ Slecht
6.2	Hoe beoordeelt u nu uw gezondheid over het algemeen vergeleken met een jaar geleden?	◯ Veel beter dan een jaar geleden ◯ Wat beter dan een jaar geleden ◯ Ongeveer hetzelfde als een jaar geleden ◯ Wat slechter dan een jaar geleden ◯ Veel slechter dan een jaar geleden
6.3	Wordt u door uw gezondheid op dit moment beperkt bij forse inspanning, zoals hardlopen, tillen van zware voorwerpen of een veeleisende sport beoefenen?	◯ Ja, ernstig beperkt ◯ Ja, een beetje beperkt ◯ Nee, helemaal niet beperkt
6.4	Wordt u door uw gezondheid op dit moment beperkt bij matige inspanning zoals een tafel verplaatsen, stofzuigen, zwemmen of fietsen?	◯ Ja, ernstig beperkt ◯ Ja, een beetje beperkt ◯ Nee, helemaal niet beperkt
6.5	Wordt u door uw gezondheid op dit moment beperkt bij boodschappen tillen of dragen?	◯ Ja, ernstig beperkt ◯ Ja, een beetje beperkt ◯ Nee, helemaal niet beperkt
6.6	Wordt u door uw gezondheid op dit moment beperkt bij een paar trappen oplopen?	◯ Ja, ernstig beperkt ◯ Ja, een beetje beperkt ◯ Nee, helemaal niet beperkt
6.7	Wordt u door uw gezondheid op dit moment beperkt bij één trap oplopen?	◯ Ja, ernstig beperkt ◯ Ja, een beetje beperkt ◯ Nee, helemaal niet beperkt
6.8	Wordt u door uw gezondheid op dit moment beperkt bij bukken, knielen of hurken?	◯ Ja, ernstig beperkt ◯ Ja, een beetje beperkt ◯ Nee, helemaal niet beperkt
6.9	Wordt u door uw gezondheid op dit moment beperkt bij meer dan één kilometer lopen?	◯ Ja, ernstig beperkt ◯ Ja, een beetje beperkt ◯ Nee, helemaal niet beperkt
6.10	Wordt u door uw gezondheid op dit moment beperkt bij een paar honderd meter lopen?	◯ Ja, ernstig beperkt ◯ Ja, een beetje beperkt ◯ Nee, helemaal niet beperkt

Paget LDA, *et al*. *BMJ Open* 2019; 9:e030961. doi: 10.1136/bmjopen-2019-030961

6.11	Wordt u door uw gezondheid op dit moment beperkt bij ongeveer honderd meter lopen?	◯ Ja, ernstig beperkt ◯ Ja, een beetje beperkt ◯ Nee, helemaal niet beperkt
6.12	Wordt u door uw gezondheid op dit moment beperkt bij uzelf wassen of aankleden?	◯ Ja, ernstig beperkt ◯ Ja, een beetje beperkt ◯ Nee, helemaal niet beperkt
6.13	U besteedde in de afgelopen 4 weken minder tijd aan werk of andere bezigheden	◯ Ja ◯ Nee
6.14	U heeft in de afgelopen 4 weken minder bereikt dan u zou willen	◯ Ja ◯ Nee
6.15	U was in de afgelopen 4 weken beperkt in het soort werk of andere bezigheden.	◯ Ja ◯ Nee
6.16	U had de afgelopen 4 weken moeite om uw werk of andere bezigheden uit te voeren (het kostte u bijvoorbeeld extra inspanning).	◯ Ja ◯ Nee
6.17	U besteedde in de afgelopen 4 weken minder tijd aan werk of andere bezigheden ten gevolge van emotionele problemen	◯ Ja ◯ Nee
6.18	U heeft in de afgelopen 4 weken minder bereikt dan u zou willen ten gevolge van emotionele problemen.	◯ Ja ◯ Nee
6.19	U deed de afgelopen 4 weken uw werk of andere bezigheden niet zo zorgvuldig als gewoonlijk ten gevolge van emotionele problemen.	◯ Ja ◯ Nee
6.20	In hoeverre hebben uw lichamelijke gezondheid of emotionele problemen u gedurende de afgelopen 4 weken gehinderd in uw normale omgang met familie, vrienden of buren, of bij activiteiten in groepsverband?	◯ Helemaal niet ◯ Enigszins ◯ Nogal ◯ Veel ◯ Heel erg veel
6.21	Hoeveel lichamelijke pijn heeft u de afgelopen 4 weken gehad?	◯ Geen<br>◯ Heel licht<br>◯ Licht<br>◯ Nogal<br>◯ Ernstig<br>◯ Heel ernstig
6.22	In welke mate bent u de afgelopen 4 weken door de pijn gehinderd in uw normale werk (zowel werk buitenshuis als huishoudelijk werk)?	◯ Helemaal niet<br>◯ Een klein beetje<br>◯ Nogal<br>◯ Veel<br>◯ Heel erg veel

Paget LDA, *et al. BMJ Open* 2019; 9:e030961. doi: 10.1136/bmjopen-2019-030961

6.23	Voelde u zich levenslustig?	◯ Altijd<br>◯ Meestal<br>◯ Vaak<br>◯ Soms<br>◯ Zelden<br>◯ Nooit
6.24	Was u erg zenuwachtig?	◯ Altijd<br>◯ Meestal<br>◯ Vaak<br>◯ Soms<br>◯ Zelden<br>◯ Nooit
6.25	Zat u zo in de put dat u niets kon?	◯ Altijd<br>◯ Meestal<br>◯ Vaak<br>◯ Soms<br>◯ Zelden<br>◯ Nooit
6.26	Voelde u zich rustig en tevreden?	◯ Altijd<br>◯ Meestal<br>◯ Vaak<br>◯ Soms<br>◯ Zelden<br>◯ Nooit
6.27	Had u veel energie?	◯ Altijd<br>◯ Meestal<br>◯ Vaak<br>◯ Soms<br>◯ Zelden<br>◯ Nooit
6.28	Voelde u zich somber en neerslachtig?	◯ Altijd<br>◯ Meestal<br>◯ Vaak<br>◯ Soms<br>◯ Zelden<br>◯ Nooit

Paget LDA, *et al. BMJ Open* 2019; 9:e030961. doi: 10.1136/bmjopen-2019-030961

6.29	Voelde u zich uitgeput?	◯ Altijd<br>◯ Meestal<br>◯ Vaak<br>◯ Soms<br>◯ Zelden<br>◯ Nooit
6.30	Was u een gelukkig mens?	◯ Altijd<br>◯ Meestal<br>◯ Vaak<br>◯ Soms<br>◯ Zelden<br>◯ Nooit
6.31	Voelde u zich moe?	◯ Altijd<br>◯ Meestal<br>◯ Vaak<br>◯ Soms<br>◯ Zelden<br>◯ Nooit
6.32	Hoe vaak hebben uw lichamelijke gezondheid of emotionele problemen u gedurende de afgelopen 4 weken gehinderd bij uw sociale activiteiten (zoals vrienden of familie bezoeken etc.)?	◯ Altijd<br>◯ Meestal<br>◯ Soms<br>◯ Zelden<br>◯ Nooit
6.33	Ik lijk wat gemakkelijker ziek te worden dan andere mensen	◯ Volkomen juist<br>◯ Grotendeels juist<br>◯ Weet ik niet<br>◯ Grotendeels onjuist<br>◯ Volkomen onjuist
6.34	Ik ben even gezond als andere mensen die ik ken	◯ Volkomen juist<br>◯ Grotendeels juist<br>◯ Weet ik niet<br>◯ Grotendeels onjuist<br>◯ Volkomen onjuist

Paget LDA, *et al. BMJ Open* 2019; 9:e030961. doi: 10.1136/bmjopen-2019-030961

6.35	Ik verwacht dat mijn gezondheid achteruit zal gaan	◯ Volkomen juist<br>◯ Grotendeels juist<br>◯ Weet ik niet<br>◯ Grotendeels onjuist<br>◯ Volkomen onjuist
6.36	Mijn gezondheid is uitstekend	◯ Volkomen juist<br>◯ Grotendeels juist<br>◯ Weet ik niet<br>◯ Grotendeels onjuist<br>◯ Volkomen onjuist

Vragenlijst PRIMA studie - EQ-5D-3L

Number	Question	Answers
	Deze vragenlijst gaat over uw standpunten t.a.v. uw gezondheid. Met behulp van deze gegevens kan worden bijgehouden hoe u zich voelt en hoe goed u in staat bent uw gebruikelijke bezigheden uit te voeren. Beantwoord elke vraag door een antwoord aan te klikken. Als u niet zeker weet hoe u een vraag moet beantwoorden, geef dan het best mogelijke antwoord.	
7.1	Hoe is het met uw Mobiliteit gesteld?	◯ Ik heb geen problemen met lopen<br>◯ Ik heb enige problemen met lopen<br>◯ Ik ben bedlegerig
7.2	Hoe is het met uw Zelfzorg gesteld?	◯ Ik heb geen problemen om mijzelf te wassen of aan te kleden<br>◯ Ik heb enige problemen om mijzelf te wassen of aan te kleden<br>◯ Ik ben niet in staat om mijzelf te wassen of aan te kleden
7.3	Hoe is het met de Dagelijkse activiteiten (werk, studie, huishouden, gezins- en vrijetijdsactiviteiten) gesteld?	◯ Ik heb geen problemen met mijn dagelijkse activiteiten<br>◯ Ik heb enige problemen met mijn dagelijkse activiteiten<br>◯ Ik ben niet in staat om mijn dagelijkse activiteiten uit te voeren

Paget LDA, *et al. BMJ Open* 2019; 9:e030961. doi: 10.1136/bmjopen-2019-030961

7.4	Hoe is het met de Pijn/klachten gesteld?	◯ Ik heb geen pijn of andere klachten<br>◯ Ik heb matige pijn of andere klachten<br>◯ Ik heb zeer ernstige pijn of andere klachten
7.5	Hoe is het met de Stemming gesteld?	◯ Ik ben niet angstig of somber<br>◯ Ik ben matig angstig of somber<br>◯ Ik ben erg angstig of somber
	We willen weten hoe goed of slecht uw gezondheid VANDAAG is. Deze meetschaal loopt van 0 tot 100: 100 staat voor de BESTE gezondheid die u zich kunt voorstellen - 0 staat voor de SLECHTSTE gezondheid die u zich kunt voorstellen	
7.6	Uw Gezondheid vandaag	(0.00) (100.00)

Vragenlijst PRIMA studie - AOS

Number	Question	Answers
	Instructies: De lijn naast elke vraag staat voor hoeveel PIJN u heeft in verschillende situaties. De linker kant (0) is “geen pijn” en de rechter kant (100) is “ergste pijn denkbaar”. Geef voor de onderstaande situaties op de lijn aan hoeveel PIJN u in de afgelopen week in de enkel had. Als een of meerdere van deze situaties niet van toepassing waren, dan kiest u de "niet van toepassing" optie.	
8.1	Wat was de hoogte van de ergste pijn in de afgelopen week?	(0.00) (100.00)
8.2	Heeft u pijn als u 's ochtends opstaat?	◯ Ja<br>◯ Nee<br>◯ Niet van toepassing
8.2.1	***If 'Heeft u pijn als u 's ochtends opstaat?' is equal to 'Ja' answer this question:***<br>Hoeveel pijn heeft u voordat u 's ochtends opstaat?	(0.00) (100.00)
8.3	Heeft u pijn wanneer u op blote voeten loopt?	◯ Ja<br>◯ Nee<br>◯ Niet van toepassing
8.3.1	***If 'Heeft u pijn wanneer u op blote voeten loopt?' is equal to 'Ja' answer this question:***<br>Hoeveel pijn heeft u als u op blote voeten loopt?	(0.00) (100.00)

Paget LDA, *et al*. *BMJ Open* 2019; 9:e030961. doi: 10.1136/bmjopen-2019-030961

8.4	Heeft u pijn wanneer u op blote voeten staat?	◯ Ja<br>◯ Nee<br>◯ Niet van toepassing	
8.4.1	***If 'Heeft u pijn wanneer u op blote voeten staat?' is equal to 'Ja' answer this question:***<br>Hoeveel pijn heeft u als u op blote voeten staat?	(0.00)	(100.00)
8.5	Heeft u pijn wanneer u met schoenen loopt?	◯ Ja<br>◯ Nee<br>◯ Niet van toepassing	
8.5.1	***If 'Heeft u pijn wanneer u met schoenen loopt?' is equal to 'Ja' answer this question:***<br>Hoeveel pijn heeft u wanneer u met schoenen loopt?	(0.00)	(100.00)
8.6	Heeft u pijn wanneer u met schoenen staat?	◯ Ja<br>◯ Nee<br>◯ Niet van toepassing	
8.6.1	***If 'Heeft u pijn wanneer u met schoenen staat?' is equal to 'Ja' answer this question:***<br>Hoeveel pijn heeft u wanneer u met schoenen staat?	(0.00)	(100.00)
8.7	Heeft u pijn wanneer u loopt met steunzolen of een brace?	◯ Ja<br>◯ Nee<br>◯ Niet van toepassing	
8.7.1	***If 'Heeft u pijn wanneer u loopt met steunzolen of een brace?' is equal to 'Ja' answer this question:***<br>Hoeveel pijn heeft u wanneer u loopt met steunzolen of een brace?	(0.00)	(100.00)
8.8	Heeft u pijn wanneer u staat met steunzolen of een brace?	◯ Ja<br>◯ Nee<br>◯ Niet van toepassing	
8.8.1	***If 'Heeft u pijn wanneer u staat met steunzolen of een brace?' is equal to 'Ja' answer this question:***<br>Hoeveel pijn heeft u wanneer u staat met steunzolen of een brace?	(0.00)	(100.00)
8.9	Heeft u pijn aan het einde van de dag?	◯ Ja<br>◯ Nee<br>◯ Niet van toepassing	

Paget LDA, *et al. BMJ Open* 2019; 9:e030961. doi: 10.1136/bmjopen-2019-030961

8.9.1	***If 'Heeft u pijn aan het einde van de dag?' is equal to 'Ja' answer this question:***<br>Hoeveel pijn heeft u aan het einde van de dag?	(0.00)	(100.00)
	Instructies:De lijn naast elke vraag staat voor hoeveel MOEITE u heeft met verschillende activiteiten. De linker kant (0) is “Niet moeilijk” en de rechter kant (100) is “Te moeilijk, niet uitvoerbaar”. Geef voor de onderstaande activiteiten op de lijn aan hoeveel MOEITE u in de afgelopen week door enkelklachten had met onderstaande activiteiten. Als een situatie niet van toepassing is, kies dan de optie "niet van toepassing".		
8.10	Heeft u moeite met door het huis lopen?	◯ Ja<br>◯ Nee<br>◯ Niet van toepassing	
8.10.1	***If 'Heeft u moeite met door het huis lopen?' is equal to 'Ja' answer this question:***<br>Hoeveel moeite heeft u met door het huis lopen?	(0.00)	(100.00)
8.11	Heeft u moeite met buiten lopen op oneven ondergrond?	◯ Ja<br>◯ Nee<br>◯ Niet van toepassing	
8.11.1	***If 'Heeft u moeite met buiten lopen op oneven ondergrond?' is equal to 'Ja' answer this question:***<br>Hoeveel moeite heeft u met buiten lopen op oneven ondergrond?	(0.00)	(100.00)
8.12	Heeft u moeite met een paar honderd meter lopen?	◯ Ja<br>◯ Nee<br>◯ Niet van toepassing	
8.12.1	***If 'Heeft u moeite met een paar honderd meter lopen?' is equal to 'Ja' answer this question:***<br>Hoeveel moeite heeft u met een paar honderd meter lopen?	(0.00)	(100.00)
8.13	Heeft u moeite met een trap oplopen?	◯ Ja<br>◯ Nee<br>◯ Niet van toepassing	
8.13.1	***If 'Heeft u moeite met een trap oplopen?' is equal to 'Ja' answer this question:***<br>Hoeveel moeite heeft u met een trap oplopen?	(0.00)	(100.00)
8.14	Heeft u moeite met een trap aflopen?	◯ Ja<br>◯ Nee<br>◯ Niet van toepassing	

Paget LDA, *et al. BMJ Open* 2019; 9:e030961. doi: 10.1136/bmjopen-2019-030961

8.14.1	***If 'Heeft u moeite met een trap aflopen?' is equal to 'Ja' answer this question:***<br>Hoeveel moeite heeft u met een trap aflopen?	(0.00)	(100.00)
8.15	Heeft u moeite met op de tenen staan?	◯ Ja<br>◯ Nee<br>◯ Niet van toepassing	
8.15.1	***If 'Heeft u moeite met op de tenen staan?' is equal to 'Ja' answer this question:***<br>Hoeveel moeite heeft u met op de tenen staan?	(0.00)	(100.00)
8.16	Heeft u moeite met opstaan uit de stoel?	◯ Ja<br>◯ Nee<br>◯ Niet van toepassing	
8.16.1	***If 'Heeft u moeite met opstaan uit de stoel?' is equal to 'Ja' answer this question:***<br>Hoeveel moeite heeft u met opstaan uit de stoel?	(0.00)	(100.00)
8.17	Heeft u moeite met het op- of afstappen van stoepranden?	◯ Ja<br>◯ Nee<br>◯ Niet van toepassing	
8.17.1	***If 'Heeft u moeite met het op- of afstappen van stoepranden?' is equal to 'Ja' answer this question:***<br>Hoeveel moeite heeft u met het op- of afstappen van stoepranden?	(0.00)	(100.00)
8.18	Heeft u moeite met snel lopen of rennen?	◯ Ja<br>◯ Nee<br>◯ Niet van toepassing	
8.18.1	***If 'Heeft u moeite met snel lopen of rennen?' is equal to 'Ja' answer this question:***<br>Hoeveel moeite heeft u met snel lopen of rennen?	(0.00)	(100.00)

Vragenlijst PRIMA studie - FAOS

Number	Question	Answers

Paget LDA, *et al. BMJ Open* 2019; 9:e030961. doi: 10.1136/bmjopen-2019-030961

	Deze lijst vraagt naar uw mening over uw voet/enkel. Uw antwoorden geven ons een beeld van uw voet/enkel klachten en hoe u in staat bent om alledaagse activiteiten uit te voeren in uw huidige situatie. Beantwoorden van een vraag doet u door het aanklikken van een vakje met het volgens u meest juiste antwoord (één vakje per vraag). Als u niet zeker weet hoe u een vraag moet beantwoorden, geeft u dan het antwoord dat volgens u het meest op uw situatie van toepassing is. Deze vraag heeft betrekking op het voorkomen van voet/enkel klachten in de afgelopen week.	
9.1	Is uw voet/enkel gezwollen?	◯ Nooit ◯ Zelden ◯ Soms ◯ Vaak ◯ Altijd
9.2	Voelt u gekraak of hoort u klikken of een ander vreemd geluid wanneer u de voet/enkel beweegt?	◯ Nooit ◯ Zelden ◯ Soms ◯ Vaak ◯ Altijd
9.3	Hapert uw enkel of blokkeert uw enkel ('op slot' gaan zitten) wanneer u deze beweegt?	◯ Nooit ◯ Zelden ◯ Soms ◯ Vaak ◯ Altijd
9.4	Kunt u de voet/enkel volledig strekken?	◯ Altijd ◯ Vaak ◯ Soms ◯ Zelden ◯ Nooit
9.5	Kunt u de voet volledig naar u toe buigen?	◯ Altijd ◯ Vaak ◯ Soms ◯ Zelden ◯ Nooit
9.6	In welke mate heeft u een stijf gevoel in de voet/enkel 's ochtends bij het wakker worden?	◯ Niet ◯ Mild ◯ Matig ◯ Ernstig ◯ Zeer ernstig
9.7	In welke mate heeft u een stijf gevoel in de voet/enkel na zitten, liggen of rusten later op de dag?	◯ Niet ◯ Mild ◯ Matig ◯ Ernstig ◯ Zeer ernstig
9.8	Hoe vaak heeft u pijn in uw voet/enkel?	◯ Nooit ◯ Maandelijks ◯ Wekelijks ◯ Dagelijks ◯ Altijd
9.9	Hoeveel voet/enkel pijn heeft u gehad in de afgelopen week bij draaien als uw voet/enkel op de grond staat?	◯ Geen<br>◯ Mild<br>◯ Matig<br>◯ Ernstig<br>◯ Zeer ernstig
9.10	Hoeveel voet/enkel pijn heeft u gehad in de afgelopen week bij het volledig uitstrekken van de voet/enkel?	◯ Geen<br>◯ Mild<br>◯ Matig<br>◯ Ernstig<br>◯ Zeer ernstig

Paget LDA, *et al. BMJ Open* 2019; 9:e030961. doi: 10.1136/bmjopen-2019-030961

9.11	Hoeveel voet/enkel pijn heeft u gehad in de afgelopen week bij het volledig naar u toe buigen/optrekken van de voet/enkel	◯ Geen<br>◯ Mild<br>◯ Matig<br>◯ Ernstig<br>◯ Zeer ernstig
9.12	Hoeveel voet/enkel pijn heeft u gehad in de afgelopen week bij het lopen op een vlakke ondergrond	◯ Geen<br>◯ Mild<br>◯ Matig<br>◯ Ernstig<br>◯ Zeer ernstig
9.13	Hoeveel voet/enkel pijn heeft u gehad in de afgelopen week bij het trap op- en trap af lopen?	◯ Geen<br>◯ Mild<br>◯ Matig<br>◯ Ernstig<br>◯ Zeer ernstig
9.14	Hoeveel voet/enkel pijn heeft u gehad in de afgelopen week 's nachts in bed?	◯ Geen<br>◯ Mild<br>◯ Matig<br>◯ Ernstig<br>◯ Zeer ernstig
9.15	Hoeveel voet/enkel pijn heeft u gehad in de afgelopen week bij het zitten of liggen?	◯ Geen<br>◯ Mild<br>◯ Matig<br>◯ Ernstig<br>◯ Zeer ernstig
9.16	Hoeveel voet/enkel pijn heeft u gehad in de afgelopen week bij het rechtop staan?	◯ Geen<br>◯ Mild<br>◯ Matig<br>◯ Ernstig<br>◯ Zeer ernstig
9.17	In welke mate werd u gehinderd bij het trap aflopen?	◯ Niet ◯ Mild ◯ Matig ◯ Ernstig ◯ Zeer ernstig
9.18	In welke mate werd u gehinderd bij het trap op lopen?	◯ Niet ◯ Mild ◯ Matig ◯ Ernstig ◯ Zeer ernstig

Paget LDA, *et al. BMJ Open* 2019; 9:e030961. doi: 10.1136/bmjopen-2019-030961

9.19	In welke mate werd u gehinderd als u vanuit een zittende positie ging staan?	◯ Niet ◯ Mild ◯ Matig ◯ Ernstig ◯ Zeer ernstig
9.20	In welke mate werd u gehinderd bij het staan?	◯ Niet ◯ Mild ◯ Matig ◯ Ernstig ◯ Zeer ernstig
9.21	In welke mate werd u gehinderd bij het naar de grond buigen/iets oprapen?	◯ Niet ◯ Mild ◯ Matig ◯ Ernstig ◯ Zeer ernstig
9.22	In welke mate werd u gehinderd bij het lopen op een vlakke ondergrond?	◯ Niet ◯ Mild ◯ Matig ◯ Ernstig ◯ Zeer ernstig
9.23	In welke mate werd u gehinderd bij het in- en uit de auto stappen?	◯ Niet ◯ Mild ◯ Matig ◯ Ernstig ◯ Zeer ernstig
9.24	In welke mate werd u gehinderd bij het boodschappen doen?	◯ Niet ◯ Mild ◯ Matig ◯ Ernstig ◯ Zeer ernstig
9.25	In welke mate werd u gehinderd bij sokken/panty's aantrekken?	◯ Niet ◯ Mild ◯ Matig ◯ Ernstig ◯ Zeer ernstig
9.26	In welke mate werd u gehinderd bij het opstaan uit bed?	◯ Niet ◯ Mild ◯ Matig ◯ Ernstig ◯ Zeer ernstig
9.27	In welke mate werd u gehinderd bij het sokken uittrekken?	◯ Niet ◯ Mild ◯ Matig ◯ Ernstig ◯ Zeer ernstig
9.28	In welke mate werd u gehinderd bij het in bed liggen (omdraaien, lange tijd uw voet/enkel in dezelfde positie houden)?	◯ Niet ◯ Mild ◯ Matig ◯ Ernstig ◯ Zeer ernstig
9.29	In welke mate werd u gehinderd bij het in/uit bad stappen?	◯ Niet ◯ Mild ◯ Matig ◯ Ernstig ◯ Zeer ernstig
9.30	In welke mate werd u gehinderd bij zitten?	◯ Niet ◯ Mild ◯ Matig ◯ Ernstig ◯ Zeer ernstig
9.31	In welke mate werd u gehinderd bij het toilet op en af gaan?	◯ Niet ◯ Mild ◯ Matig ◯ Ernstig ◯ Zeer ernstig

Paget LDA, *et al. BMJ Open* 2019; 9:e030961. doi: 10.1136/bmjopen-2019-030961

9.32	In welke mate werd u gehinderd bij zwaar huishoudelijk werk (bijvoorbeeld zware dozen sjouwen, vloer schrobben)?	◯ Niet ◯ Mild ◯ Matig ◯ Ernstig ◯ Zeer ernstig
9.33	In welke mate werd u gehinderd bij licht huishoudeijk werk (bijvoorbeeld koken, afstoffen)?	◯ Niet ◯ Mild ◯ Matig ◯ Ernstig ◯ Zeer ernstig
9.34	In welke mate werd u gehinderd bij hurken?	◯ Niet ◯ Mild ◯ Matig ◯ Ernstig ◯ Zeer ernstig
9.35	In welke mate werd u gehinderd bij hardlopen?	◯ Niet ◯ Mild ◯ Matig ◯ Ernstig ◯ Zeer ernstig
9.36	In welke mate werd u gehinderd bij springen?	◯ Niet ◯ Mild ◯ Matig ◯ Ernstig ◯ Zeer ernstig
9.37	In welke mate werd u gehinderd bij ronddraaien op uw aangedane voet/enkel?	◯ Niet ◯ Mild ◯ Matig ◯ Ernstig ◯ Zeer ernstig
9.38	In welke mate werd u gehinderd bij knielen?	◯ Niet ◯ Mild ◯ Matig ◯ Ernstig ◯ Zeer ernstig
9.39	Hoe vaak bent u zich bewust van uw voet/enkel probleem?	◯ Nooit ◯ Maandelijks ◯ Wekelijks ◯ Dagelijks ◯ Altijd
9.40	Heeft u uw leven veranderd om activiteiten te vermijden die schadelijk kunnen zijn voor uw voet/enkel?	◯ Niet<br>◯ Enigszins<br>◯ Matig<br>◯ Behoorlijk<br>◯ Volledig
9.41	In hoeverre kunt u op uw voet/enkel vertrouwen?	◯ Volledig<br>◯ Behoorlijk<br>◯ Matig<br>◯ Enigzins<br>◯ Niet
9.42	In het algemeen, in welke mate ondervindt u hinder van uw voet/enkel	◯ Geen<br>◯ Mild<br>◯ Matig<br>◯ Ernstig<br>◯ Zeer ernstig

Paget LDA, *et al. BMJ Open* 2019; 9:e030961. doi: 10.1136/bmjopen-2019-030961

Vragenlijst PRIMA studie - PRODISQ - Gezondheid en werk

Number	Question	Answers
10.1	Wat is uw leeftijd?	______ jaar
10.2	Wat is uw geslacht?	○ Man<br>○ Vrouw
10.3	Wat is de hoogste opleiding die u heeft afgemaakt (Zoek uw hoogste opleiding en kruis het hokje daarvoor aan)?	☐ Ik heb geen school of opleiding afgemaakt<br>☐ Lagere school of basisschool<br>☐ Huishoudschool, vbo, lbo, lts, leao of lhno<br>☐ Mavo, mulo, ivo of vmbo<br>☐ Mbo, mts, meao, mhno, inas of intas<br>☐ Havo, vwo, hbs, mms, atheneum of gymnasium<br>☐ Hbo, hts, heao of hhno<br>☐ Universiteit<br>☐ Ik heb een andere opleiding afgemaakt, namelijk
10.3.1	***If 'Wat is de hoogste opleiding die u heeft afgemaakt (Zoek uw hoogste opleiding en kruis het hokje daarvoor aan)?' is equal to 'Ik heb een andere opleiding afgemaakt, namelijk' answer this question:***<br>Wat is de hoogste opleiding die u heeft afgemaakt?	______
10.4	Wat doet u in het dagelijks leven?	☐ Ik zit op school, ik studeer<br>☐ Ik werk in loondienst<br>☐ Ik ben zelfstandig ondernemer<br>☐ Ik ben huisvrouw, huisman<br>☐ Ik ben werkloos<br>☐ Ik ben arbeidsongeschikt<br>☐ Ik ben met pensioen of prepensioen<br>☐ Ik doe iets anders, namelijk
10.4.1	***If 'Wat doet u in het dagelijks leven?' is equal to 'Ik ben arbeidsongeschikt' answer this question:***<br>Wat doet u in het dagelijks leven?	______

Paget LDA, *et al. BMJ Open* 2019; 9:e030961. doi: 10.1136/bmjopen-2019-030961

10.4.2	***If 'Wat doet u in het dagelijks leven?' is equal to 'Ik doe iets anders, namelijk' answer this question:*** Wat doet u in het dagelijks leven?	
10.5	Hebt u betaald werk?	◯ Nee<br>◯ Ja
10.5.1	***If 'Hebt u betaald werk?' is equal to 'Ja' answer this question:*** Wat is uw beroep?	
10.5.2	***If 'Hebt u betaald werk?' is equal to 'Ja' answer this question:*** Hoeveel uur per week werkt u (Tel alleen de uren waarvoor u betaald wordt)?	uren
10.5.3	***If 'Hebt u betaald werk?' is equal to 'Ja' answer this question:*** Hoeveel dagen in de week werkt u?	Dagen
10.5.4	***If 'Hebt u betaald werk?' is equal to 'Ja' answer this question:*** Bent u in de afgelopen 4 weken afwezig geweest van uw werk omdat u ziek was?	◯ Nee<br>◯ Ja
10.5.4.1	***If 'Bent u in de afgelopen 4 weken afwezig geweest van uw werk omdat u ziek was?' is equal to 'Ja' answer this question:*** Bent u in de afgelopen 4 weken afwezig geweest van uw werk omdat u ziek was (Tel alleen de werkdagen in de afgelopen 4 weken)?	dagen afwezig geweest
10.5.4.2	***If 'Bent u in de afgelopen 4 weken afwezig geweest van uw werk omdat u ziek was?' is equal to 'Ja' answer this question:*** Was u langer dan de gehele periode van 4 weken afwezig van uw werk doordat u ziek was?	◯ Nee<br>◯ Ja
10.5.4.2.1	***If 'Was u langer dan de gehele periode van 4 weken afwezig van uw werk doordat u ziek was?' is equal to 'Ja' answer this question:*** Wanneer heeft u zich ziek gemeld?	*(dd-mm-yyyy)*
10.5.5	***If 'Hebt u betaald werk?' is equal to 'Ja' answer this question:*** Waren er in de afgelopen 4 weken, dagen waarop u wel gewerkt heeft, maar tijdens uw werk last had van lichamelijke of psychische problemen?	◯ Ja<br>◯ Nee

Paget LDA, *et al. BMJ Open* 2019; 9:e030961. doi: 10.1136/bmjopen-2019-030961

10.5.5.1	***If 'Waren er in de afgelopen 4 weken, dagen waarop u wel gewerkt heeft, maar tijdens uw werk last had van lichamelijke of psychische problemen?' is equal to 'Ja' answer this question:*** Op hoeveel werkdagen had u tijdens uw werk last van uw lichamelijke of psychische problemen?	[ ] werkdagen
10.5.5.2	***If 'Waren er in de afgelopen 4 weken, dagen waarop u wel gewerkt heeft, maar tijdens uw werk last had van lichamelijke of psychische problemen?' is equal to 'Ja' answer this question:*** Op de dagen dat u last had, kon u misschien niet zoveel werk doen als normaal. Hoeveel werk kon u op deze dagen gemiddeld doen?	Ik kon op deze dagen niks doen (0.00) — Ik kon net zoveel doen als normaal (10.00)
10.6	Waren er dagen waarop u minder onbetaald werk kon doen door uw lichamelijke of psychische problemen?	◯ Nee<br>◯ Ja
10.6.1	***If 'Waren er dagen waarop u minder onbetaald werk kon doen door uw lichamelijke of psychische problemen?' is equal to 'Ja' answer this question:*** Op hoeveel dagen was dit zo?	[ ] Dagen
10.6.2	***If 'Waren er dagen waarop u minder onbetaald werk kon doen door uw lichamelijke of psychische problemen?' is equal to 'Ja' answer this question:*** Stel dat iemand, bijvoorbeeld uw partner, familielid of een bekende, u op deze dagen had geholpen. En al het onbetaalde werk wat u niet kon doen, voor u had gedaan. Hoeveel uur was die persoon hier op deze dagen dan gemiddeld mee bezig geweest?	[ ] uur op deze dagen

Vragenlijst PRIMA studie - PRODISQ - Zorggebruik

Number	Question	Answers
	Wij willen graag weten met welke dokters u in de afgelopen 3 maanden een afspraak had. Het gaat om afspraken voor uzelf. Ook andere zorgverleners tellen mee. Bijvoorbeeld de fysiotherapeut.	
11.1	Bent u in de afgelopen 3 maanden naar uw huisarts geweest?	◯ Nee<br>◯ Ja
11.1.1	***If 'Bent u in de afgelopen 3 maanden naar uw huisarts geweest?' is equal to 'Ja' answer this question:*** Hoeveel afspraken had u?	[ ] afspraken

Paget LDA, *et al*. *BMJ Open* 2019; 9:e030961. doi: 10.1136/bmjopen-2019-030961

11.2	Bent u in de afgelopen 3 maanden in contact geweest met een maatschappelijk werker?	◯ Nee<br>◯ Ja
11.2.1	***If 'Bent u in de afgelopen 3 maanden in contact geweest met een maatschappelijk werker?' is equal to 'Ja' answer this question:***<br>Hoeveel afspraken had u?	[ ] afspraken
11.3	Bent u in de afgelopen 3 maanden naar een fysiotherapeut geweest? Of een caesartherapeut, therapeut mensendieck of een manueel therapeut?	◯ Nee<br>◯ Ja
11.3.1	***If 'Bent u in de afgelopen 3 maanden naar een fysiotherapeut geweest? Of een caesartherapeut, therapeut mensendieck of een manueel therapeut?' is equal to 'Ja' answer this question:***<br>Hoeveel afspraken had u?	[ ] afspraken
11.4	Bent u in de afgelopen 3 maanden naar een ergotherapeut geweest?	◯ Nee<br>◯ Ja
11.4.1	***If 'Bent u in de afgelopen 3 maanden naar een ergotherapeut geweest?' is equal to 'Ja' answer this question:***<br>Hoeveel afspraken had u?	[ ] afspraken
11.5	Bent u in de afgelopen 3 maanden naar een logopedist geweest?	◯ Nee<br>◯ Ja
11.5.1	***If 'Bent u in de afgelopen 3 maanden naar een logopedist geweest?' is equal to 'Ja' answer this question:***<br>Hoeveel afspraken had u?	[ ] afspraken
11.6	Bent u in de afgelopen 3 maanden naar een diëtist geweest?	◯ Nee<br>◯ Ja
11.6.1	***If 'Bent u in de afgelopen 3 maanden naar een diëtist geweest?' is equal to 'Ja' answer this question:***<br>Hoeveel afspraken had u?	[ ] afspraken
11.7	Bent u in de afgelopen 3 maanden naar een homeopaat geweest? Of een acupuncturist?	◯ Nee<br>◯ Ja
11.7.1	***If 'Bent u in de afgelopen 3 maanden naar een homeopaat geweest? Of een acupuncturist?' is equal to 'Ja' answer this question:***<br>Hoeveel afspraken had u?	[ ] afspraken

Paget LDA, *et al. BMJ Open* 2019; 9:e030961. doi: 10.1136/bmjopen-2019-030961

11.8	Bent u in de afgelopen 3 maanden naar een psycholoog geweest? Of een psychotherapeut of psychiater?	◯ Nee<br>◯ Ja
11.8.1	***If 'Bent u in de afgelopen 3 maanden naar een psycholoog geweest? Of een psychotherapeut of psychiater?' is equal to 'Ja' answer this question:***<br>Hoeveel afspraken had u?	______ afspraken
11.9	Heeft u in de afgelopen 3 maanden afspraken gehad met de bedrijfsarts?	◯ Nee<br>◯ Ja
11.9.1	***If 'Heeft u in de afgelopen 3 maanden afspraken gehad met de bedrijfsarts?' is equal to 'Ja' answer this question:***<br>Hoeveel afspraken had u?	______ afspraken
11.10	Heeft u in de afgelopen 3 maanden hulp van de thuiszorg gehad?	◯ Ja<br>◯ Nee
11.10.1	***If 'Heeft u in de afgelopen 3 maanden hulp van de thuiszorg gehad?' is equal to 'Ja' answer this question:***<br>Wat voor hulp van de thuiszorg heeft u gehad in de afgelopen 3 maanden?	☐ Huishoudelijke hulp; voorbeeld: stofzuigen, bed opmaken, boodschappen doen<br>☐ Verzorging van uzelf; voorbeeld: hulp bij douchen of aankleden<br>☐ Verpleging; voorbeeld: verband omdoen, medicijnen geven, bloeddruk meten
11.10.1.1	***If 'Wat voor hulp van de thuiszorg heeft u gehad in de afgelopen 3 maanden?' is equal to 'Huishoudelijke hulp; voorbeeld: stofzuigen, bed opmaken, boodschappen doen' answer this question:***<br>Hoeveel weken heeft u deze thuiszorg gehad? Huishoudelijke hulp:	______ weken in de afgelopen 3 maanden
11.10.1.2	***If 'Wat voor hulp van de thuiszorg heeft u gehad in de afgelopen 3 maanden?' is equal to 'Verzorging van uzelf; voorbeeld: hulp bij douchen of aankleden' answer this question:***<br>Hoeveel weken heeft u deze thuiszorg gehad? Verzorging van uzelf:	______ weken in de afgelopen 3 maanden
11.10.1.3	***If 'Wat voor hulp van de thuiszorg heeft u gehad in de afgelopen 3 maanden?' is equal to 'Verpleging; voorbeeld: verband omdoen, medicijnen geven, bloeddruk meten' answer this question:***<br>Hoeveel weken heeft u deze thuiszorg gehad? Verpleging:	______ weken in de afgelopen 3 maanden

Paget LDA, *et al. BMJ Open* 2019; 9:e030961. doi: 10.1136/bmjopen-2019-030961

11.10.1.4	***If 'Wat voor hulp van de thuiszorg heeft u gehad in de afgelopen 3 maanden?' is equal to 'Huishoudelijke hulp; voorbeeld: stofzuigen, bed opmaken, boodschappen doen' answer this question:***<br>Hoeveel uur thuiszorg kreeg u in deze weken gemiddeld? Huishoudelijke hulp: gemiddeld	[ ] uur in de week
11.10.1.5	***If 'Wat voor hulp van de thuiszorg heeft u gehad in de afgelopen 3 maanden?' is equal to 'Verzorging van uzelf; voorbeeld: hulp bij douchen of aankleden' answer this question:***<br>Hoeveel uur thuiszorg kreeg u in deze weken gemiddeld? Verzorging van uzelf: gemiddeld	[ ] uur in de week
11.10.1.6	***If 'Wat voor hulp van de thuiszorg heeft u gehad in de afgelopen 3 maanden?' is equal to 'Verpleging; voorbeeld: verband omdoen, medicijnen geven, bloeddruk meten' answer this question:***<br>Hoeveel uur thuiszorg kreeg u in deze weken gemiddeld? Verpleging: gemiddeld	[ ] uur in de week
11.11	Heeft u in de afgelopen 3 maanden medicijnen gebruikt?	○ Ja<br>○ Nee
11.11.1	***If 'Heeft u in de afgelopen 3 maanden medicijnen gebruikt?' is equal to 'Ja' answer this question:***<br>Welke medicijnen heeft u in de afgelopen 3 maanden gebruikt?	[ ]
11.12	Bent u in de afgelopen 3 maanden op de spoedeisende eerste hulp van een ziekenhuis geweest? (Een andere naam voor spoedeisende eerste hulp is EHBO)	○ Nee<br>○ Ja
11.12.1	***If 'Bent u in de afgelopen 3 maanden op de spoedeisende eerste hulp van een ziekenhuis geweest? (Een andere naam voor spoedeisende eerste hulp is EHBO)' is equal to 'Ja' answer this question:***<br>Hoevaak bent u geweest?	[ ] keer
11.13	Bent u in de afgelopen 3 maanden met een ambulance naar het ziekenhuis gebracht? (Een andere naam voor ambulance is ziekenauto)	○ Nee<br>○ Ja
11.13.1	***If 'Bent u in de afgelopen 3 maanden met een ambulance naar het ziekenhuis gebracht? (Een andere naam voor ambulance is ziekenauto)' is equal to 'Ja' answer this question:***<br>Hoe vaak bent u naar het ziekenhuis gebracht?	[ ] keer

Paget LDA, *et al. BMJ Open* 2019; 9:e030961. doi: 10.1136/bmjopen-2019-030961

11.14	Had u in de afgelopen 3 maanden een afspraak bij de polikliniek van het ziekenhuis? (Het gaat om afspraken voor uzelf met een dokter. Bijvoorbeeld met de cardioloog, reumatoloog of neuroloog)	◯ Ja<br>◯ Nee
11.14.1	***If 'Had u in de afgelopen 3 maanden een afspraak bij de polikliniek van het ziekenhuis? (Het gaat om afspraken voor uzelf met een dokter. Bijvoorbeeld met de cardioloog, reumatoloog of neuroloog)' is equal to 'Ja' answer this question:***<br>Bij welke soorten dokters bent u in de afgelopen 3 maanden in het ziekenhuis geweest? En hoe vaak?	
11.15	Bent u in de afgelopen 3 maanden overdag in het ziekenhuis geweest voor een behandeling? (U bleef dus niet slapen. U kwam bijvoorbeeld voor een bloedtransfusie, nierdialyse of chemokuur)	◯ Ja<br>◯ Nee
11.15.1	***If 'Bent u in de afgelopen 3 maanden overdag in het ziekenhuis geweest voor een behandeling? (U bleef dus niet slapen. U kwam bijvoorbeeld voor een bloedtransfusie, nierdialyse of chemokuur)' is equal to 'Ja' answer this question:***<br>Voor welke soort behandeling was dit?	
11.15.2	***If 'Bent u in de afgelopen 3 maanden overdag in het ziekenhuis geweest voor een behandeling? (U bleef dus niet slapen. U kwam bijvoorbeeld voor een bloedtransfusie, nierdialyse of chemokuur)' is equal to 'Ja' answer this question:***<br>Hoeveel keer moest u in de afgelopen 3 maanden voor deze behandelingen naar het ziekenhuis?	
11.16	Bent u in de afgelopen 3 maanden ergens anders geweest voor een behandeling overdag? (U bleef dus niet slapen. U ging bijvoorbeeld naar de dagopvang van een woon-/zorgcentrum of een psychiatrische instelling. Of naar de dagbehandeling van een revalidatiecentrum)	◯ Ja<br>◯ Nee
11.16.1	***If 'Bent u in de afgelopen 3 maanden ergens anders geweest voor een behandeling overdag? (U bleef dus niet slapen. U ging bijvoorbeeld naar de dagopvang van een woon-/zorgcentrum of een psychiatrische instelling. Of naar de dagbehandeling van een revalidatiecentrum)' is equal to 'Ja' answer this question:***<br>Wat voor instelling was dit?	☐ Woon-/zorgcentrum<br>☐ Revalidatiecentrum<br>☐ Psychiatrische instelling<br>☐ Een andere instelling, namelijk
11.16.1.1	***If 'Wat voor instelling was dit?' is equal to 'Een andere instelling, namelijk' answer this question:***<br>Wat voor instelling was dit?	

Paget LDA, *et al. BMJ Open* 2019; 9:e030961. doi: 10.1136/bmjopen-2019-030961

11.16.1.2	***If 'Wat voor instelling was dit?' is equal to 'Woon-/zorgcentrum' answer this question:*** Hoe vaak moest u hier in de afgelopen 3 maanden naartoe? Naar het woon-/zorgcentrum:	______ keer in de afgelopen 3 maanden
11.16.1.3	***If 'Wat voor instelling was dit?' is equal to 'Revalidatiecentrum' answer this question:*** Hoe vaak moest u hier in de afgelopen 3 maanden naartoe? Naar het revalidatiecentrum:	______ keer in de afgelopen 3 maanden
11.16.1.4	***If 'Wat voor instelling was dit?' is equal to 'Psychiatrische instelling' answer this question:*** Hoe vaak moest u hier in de afgelopen 3 maanden naartoe? Naar de psychiatrische instelling:	______ keer in de afgelopen 3 maanden
11.16.1.5	***If 'Wat voor instelling was dit?' is equal to 'Een andere instelling, namelijk' answer this question:*** Hoe vaak moest u hier in de afgelopen 3 maanden naartoe? Naar de andere instelling:	______ keer in de afgelopen 3 maanden
11.17	Heeft u in de afgelopen 3 maanden weleens in het ziekenhuis gelegen? (U moest dus blijven slapen. Bijvoorbeeld omdat u geopereerd was en niet meteen naar huis kon)	○ Ja<br>○ Nee
11.17.1	***If 'Heeft u in de afgelopen 3 maanden weleens in het ziekenhuis gelegen? (U moest dus blijven slapen. Bijvoorbeeld omdat u geopereerd was en niet meteen naar huis kon)' is equal to 'Ja' answer this question:*** Hoe vaak heeft u in de afgelopen 3 maanden in het ziekenhuis gelegen?	______ keer in de afgelopen 3 maanden
11.17.2	***If 'Heeft u in de afgelopen 3 maanden weleens in het ziekenhuis gelegen? (U moest dus blijven slapen. Bijvoorbeeld omdat u geopereerd was en niet meteen naar huis kon)' is equal to 'Ja' answer this question:*** Hoe lang heeft u in het ziekenhuis gelegen?	______ dagen in de afgelopen 3 maanden
11.18	Moest u in de afgelopen 3 maanden ergens anders blijven slapen voor uw gezondheid? (Bijvoorbeeld in een woon-/zorgcentrum, psychiatrische instelling of revalidatiecentrum)	○ Ja<br>○ Nee
11.18.1	***If 'Moest u in de afgelopen 3 maanden ergens anders blijven slapen voor uw gezondheid? (Bijvoorbeeld in een woon-/zorgcentrum, psychiatrische instelling of revalidatiecentrum)' is equal to 'Ja' answer this question:*** Wat voor instelling was dit?	☐ Woon-/zorgcentrum<br>☐ Revalidatiecentrum<br>☐ Psychiatrische instelling<br>☐ Een andere instelling, namelijk

Paget LDA, *et al. BMJ Open* 2019; 9:e030961. doi: 10.1136/bmjopen-2019-030961

11.18.1.1	***If 'Wat voor instelling was dit?' is equal to 'Een andere instelling, namelijk' answer this question:*** Een andere instelling, namelijk	[ ]
11.18.1.2	***If 'Wat voor instelling was dit?' is equal to 'Woon-/zorgcentrum' answer this question:*** Hoe lang bent u in deze instelling geweest? In het woon-/zorgcentrum:	[ ] dagen in de afgelopen 3 maanden
11.18.1.3	***If 'Wat voor instelling was dit?' is equal to 'Revalidatiecentrum' answer this question:*** Hoe lang bent u in deze instelling geweest? In het revalidatiecentrum:	[ ] dagen in de afgelopen 3 maanden
11.18.1.4	***If 'Wat voor instelling was dit?' is equal to 'Psychiatrische instelling' answer this question:*** Hoe lang bent u in deze instelling geweest? In de psychiatrische instelling:	[ ] dagen in de afgelopen 3 maanden
11.18.1.5	***If 'Wat voor instelling was dit?' is equal to 'Een andere instelling, namelijk' answer this question:*** Hoe lang bent u in deze instelling geweest? In de andere instelling:	[ ] dagen in de afgelopen 3 maanden
11.19	Heeft u in de afgelopen 3 maanden hulp gekregen van een familielid of een bekende vanwege uw lichamelijke of psychische problemen?	○ Ja<br>○ Nee
11.19.1	***If 'Heeft u in de afgelopen 3 maanden hulp gekregen van een familielid of een bekende vanwege uw lichamelijke of psychische problemen?' is equal to 'Ja' answer this question:*** Wat voor hulp van familieleden of bekenden heeft u gehad in de afgelopen 3 maanden?	☐ Huishoudelijke hulp - voorbeeld: stofzuigen, bed opmaken, boodschappen doen, klaarmaken van eten en drinken, verzorgen van kinderen<br>☐ Verzorging van uzelf - voorbeeld: hulp bij douchen of aankleden, hulp bij het eten en drinken of het geven van medicijnen<br>☐ Praktische hulp - voorbeeld: ondersteuning bij wandelen, het maken van uitstapjes of bezoekjes aan bekenden, bezoeken aan de huisarts of het ziekenhuis, het regelen van hulp of het regelen van financiële zaken
11.19.1.1	***If 'Wat voor hulp van familieleden of bekenden heeft u gehad in de afgelopen 3 maanden?' is equal to 'Huishoudelijke hulp - voorbeeld: stofzuigen, bed opmaken, boodschappen doen, klaarmaken van eten en drinken, verzorgen van kinderen' answer this question:*** Hoeveel weken heeft u deze hulp gehad? Huishoudelijke hulp:	[ ] weken in de afgelopen 3 maanden

Paget LDA, *et al. BMJ Open* 2019; 9:e030961. doi: 10.1136/bmjopen-2019-030961

11.19.1.2	***If 'Wat voor hulp van familieleden of bekenden heeft u gehad in de afgelopen 3 maanden?' is equal to 'Verzorging van uzelf - voorbeeld: hulp bij douchen of aankleden, hulp bij het eten en drinken of het geven van medicijnen' answer this question:*** Hoeveel weken heeft u deze hulp gehad? Verzorging van uzelf:	______ weken in de afgelopen 3 maanden
11.19.1.3	***If 'Wat voor hulp van familieleden of bekenden heeft u gehad in de afgelopen 3 maanden?' is equal to 'Praktische hulp - voorbeeld: ondersteuning bij wandelen, het maken van uitstapjes of bezoekjes aan bekenden, bezoeken aan de huisarts of het ziekenhuis, het regelen van hulp of het regelen van financiële zaken' answer this question:*** Hoeveel uur hulp kreeg u in deze weken gemiddeld? Praktische hulp:	______ weken in de afgelopen 3 maanden
11.19.1.4	***If 'Wat voor hulp van familieleden of bekenden heeft u gehad in de afgelopen 3 maanden?' is equal to 'Huishoudelijke hulp - voorbeeld: stofzuigen, bed opmaken, boodschappen doen, klaarmaken van eten en drinken, verzorgen van kinderen' answer this question:*** Hoeveel uur hulp kreeg u in deze weken gemiddeld? Huishoudelijke hulp: gemiddeld	______ uur in de week
11.19.1.5	***If 'Wat voor hulp van familieleden of bekenden heeft u gehad in de afgelopen 3 maanden?' is equal to 'Verzorging van uzelf - voorbeeld: hulp bij douchen of aankleden, hulp bij het eten en drinken of het geven van medicijnen' answer this question:*** Hoeveel uur hulp kreeg u in deze weken gemiddeld? Verzorging van uzelf: gemiddeld	______ uur in de week
11.19.1.6	***If 'Wat voor hulp van familieleden of bekenden heeft u gehad in de afgelopen 3 maanden?' is equal to 'Praktische hulp - voorbeeld: ondersteuning bij wandelen, het maken van uitstapjes of bezoekjes aan bekenden, bezoeken aan de huisarts of het ziekenhuis, het regelen van hulp of het regelen van financiële zaken' answer this question:*** Hoeveel uur hulp kreeg u in deze weken gemiddeld? Praktische hulp: gemiddeld	______ uur in de week

Vragenlijst PRIMA studie - Dank

Number	Question	Answers

Paget LDA, *et al. BMJ Open* 2019; 9:e030961. doi: 10.1136/bmjopen-2019-030961

12.1	Hartelijk dank voor de tijd die u heeft genomen om de vragenlijsten in te vullen. Indien u nog opmerkingen heeft kunt u deze hierin plaatsen.	

Survey 'Vragenlijst PRIMA studie 12 weken'

Vragenlijst PRIMA studie 12 weken - AOFAS - Pijn

Number	Question	Answers
1.1	Hoeveel pijn ervaart u aan uw voet?	☐ Geen<br>☐ Milde pijnklachten of af en toe pijn<br>☐ Matige pijnklachten, dagelijks<br>☐ Vrijwel continu erge pijn

Vragenlijst PRIMA studie 12 weken - AOFAS resterende gedeelte

Number	Question	Answers
2.1	Hoeveel beperkingen in het dagelijks leven ervaart u?	○ Geen beperkingen, geen hulpmiddelen nodig<br>○ Geen beperkingen in algemeen dagelijkse bezigheden, wel beperkingen in recreatieve activiteiten (sport, hobbies, etc) geen ondersteuning<br>○ Beperkingen bij dagelijkse- en recreatieve activiteiten, stok<br>○ Ernstige beperkingen bij dagelijkse- en recreatieactiviteiten, walker, krukken, rolstoel, brace
2.2	Wat is de afstand die u in één keer kunt lopen?	○ Meer dan 600 meter<br>○ 400 - 600 meter<br>○ 100 - 300 meter<br>○ Minder dan 100 meter

Paget LDA, *et al. BMJ Open* 2019; 9:e030961. doi: 10.1136/bmjopen-2019-030961

2.3	Heeft u moeite met lopen op een bepaalde ondergrond?	◯ Geen problemen bij lopen<br>◯ Enig problemen op ongelijke ondergrond, trap, helling, ladders<br>◯ Ernstige problemen bij ongelijke ondergrond, trap, helling, ladders
2.4	Hoe zou u uw looppatroon beoordelen?	◯ Normaal of minimaal afwijkend<br>◯ Duidelijk<br>◯ Ernstig
2.5	Hoe zou u de beweeglijkheid van uw voet beoordelen ten opzicht van de andere zijde of ten opzicte van vóór het ongeval?	◯ Hetzelfde of licht beperkt t.o.v. de niet aangedane zijde of zoals voor het ongeval<br>◯ Ongeveer de helft beperkt<br>◯ Sterk verminderd, bijna geen bewegingen mogelijk
2.6	Hoe zou u de beweeglijkheid van uw enkel beoordelen ten opzichte van de andere zijde of ten opzichte van voor het ongeval?	◯ Hetzelfde of licht beperkt t.o.v. de niet aangedane zijde of zoals voor het ongeval<br>◯ Ongeveer de helft beperkt<br>◯ Sterk verminderd, bijna geen bewegingen mogelijk
2.7	Hoe zou u de stabiliteit van uw voet en enkel beoordelen	◯ Stabiel, ik verzwik mijn enkel hooguit incidenteel<br>◯ Niet stabiel, ik verzwik mijn enkel vaak
2.8	Hoe zou u de stand van uw voet beoordelen ten opzichte van de andere zijde of ten opzichte van voor het ongeval?	◯ Goed, ik kan mijn voet goed plat op de grond zetten, waarbij de stand niet afwijkend is<br>◯ Ik sta vrijwel alleen op de buitenzijde van mijn voet<br>◯ Ik kan mijn voet niet plat op de grond zetten en loop vrijwel alleen op mijn voorvoet

Vragenlijst PRIMA studie 12 weken - VAS Pain score

Number	Question	Answers	
3.1	VAS pijnscore (Geef uw minimaal pijnscore aan tijdens dagelijkse activiteiten op een schaal van 0 tot 100, waarbij 0 geen pijn is en 100 de ergste pijn denkbaar is)	(0.00)	100 (100.00)

Vragenlijst PRIMA studie 12 weken - AAS

Number	Question	Answers

Paget LDA, *et al. BMJ Open* 2019; 9:e030961. doi: 10.1136/bmjopen-2019-030961

4.1	Wat is het meest bij u van toepassing ten aanzien van uw acitviteit?	☐ American Football<br>☐ Basketbal<br>☐ Gymnastiek/turnen<br>☐ Handbal<br>☐ Rugby<br>☐ Voetbal<br>☐ Hockey<br>☐ Korfbal<br>☐ Gevechtssporten: judo, karate, kung fu, taekwando, jiu jitsu, etc.<br>☐ Oriëntatielopen<br>☐ Rhytmische gymnastiek<br>☐ Volleybal<br>☐ Boxen<br>☐ Freestyle snowboarden<br>☐ Ijshockey<br>☐ Tennis<br>☐ Worstelen<br>☐ Fitness, aerobics<br>☐ Badminton<br>☐ Baseball<br>☐ Cross-country hardlopen<br>☐ Moderne pentathlon<br>☐ Squash<br>☐ Surfen, windsurfen<br>☐ Tafel tennis<br>☐ Atletiek: spring-, werponderdelen<br>☐ Waterskieën<br>☐ Dans<br>☐ Schermen<br>☐ Zaalhockey<br>☐ Bergbeklimmen<br>☐ Langlauf<br>☐ Parachute springen<br>☐ Softball<br>☐ Speciale beroepen en werkactiviteiten; speciale beroepen: ballet, professioneel soldaat, speciale reddingswerker, stuntman, etc.<br>☐ Duiken<br>☐ Scubaduiken<br>☐ Skaten, in-line skaten<br>☐ Atletiek: looponderdelen<br>☐ Triatlon<br>☐ Gewichtsheffen, body-building

Paget LDA, *et al. BMJ Open* 2019; 9:e030961. doi: 10.1136/bmjopen-2019-030961

		☐ Alle competitieve sporten hieronder met 'seasonal' conditioning<br>☐ Zwaar fysiek werk<br>☐ Alpine skiën en snowboarden<br>☐ Bowlen/curlen<br>☐ Golf<br>☐ Mountainbike/BMX<br>☐ Powerliften<br>☐ Zeilen<br>☐ Fysiek werk<br>☐ Wielrennen<br>☐ Paardrijden<br>☐ Motorsporten/technische sporten<br>☐ Roeien, kayakken<br>☐ Boogschieten<br>☐ Water polo en zwemmen<br>☐ Kunnen lopen op oneven grond<br>☐ Geen sport, echter geen limitatie in dagelijkse activiteiten<br>☐ Kunnen lopen op even grond, maar de dagelijkste activiteiten zijn gelimiteerd<br>☐ Niet kunnen lopen, fysieke handicap wegens enkelproblemen
4.1.1	***If 'Wat is het meest bij u van toepassing ten aanzien van uw acitviteit?' is equal to 'American Football' answer this question:***<br>American Football	◯ Professioneel<br>◯ Competitief<br>◯ Recreatief
4.1.2	***If 'Wat is het meest bij u van toepassing ten aanzien van uw acitviteit?' is equal to 'Basketbal' answer this question:***<br>Basketbal	◯ Professioneel<br>◯ Competitief<br>◯ Recreatief
4.1.3	***If 'Wat is het meest bij u van toepassing ten aanzien van uw acitviteit?' is equal to 'Gymnastiek/turnen' answer this question:***<br>Gymnastiek/turnen	◯ Professioneel<br>◯ Competitief<br>◯ Recreatief
4.1.4	***If 'Wat is het meest bij u van toepassing ten aanzien van uw acitviteit?' is equal to 'Handbal' answer this question:***<br>Handbal	◯ Professioneel<br>◯ Competitief<br>◯ Recreatief
4.1.5	***If 'Wat is het meest bij u van toepassing ten aanzien van uw acitviteit?' is equal to 'Rugby' answer this question:***<br>Rugby	◯ Professioneel<br>◯ Competitief<br>◯ Recreatief

Paget LDA, *et al. BMJ Open* 2019; 9:e030961. doi: 10.1136/bmjopen-2019-030961

4.1.6	***If 'Wat is het meest bij u van toepassing ten aanzien van uw acitviteit?' is equal to 'Voetbal' answer this question:***<br>Voetbal	◯ Professioneel<br>◯ Competitief<br>◯ Recreatief
4.1.7	***If 'Wat is het meest bij u van toepassing ten aanzien van uw acitviteit?' is equal to 'Hockey' answer this question:***<br>Hockey	◯ Professioneel<br>◯ Competitief<br>◯ Recreatief
4.1.8	***If 'Wat is het meest bij u van toepassing ten aanzien van uw acitviteit?' is equal to 'Korfbal' answer this question:***<br>Korfbal	◯ Professioneel<br>◯ Competitief<br>◯ Recreatief
4.1.9	***If 'Wat is het meest bij u van toepassing ten aanzien van uw acitviteit?' is equal to 'Gevechtssporten: judo, karate, kung fu, taekwando, jiu jitsu, etc.' answer this question:***<br>Gevechtssporten: judo, karate, kung fu, taekwando, jiu jitsu, etc.	◯ Professioneel<br>◯ Competitief<br>◯ Recreatief
4.1.10	***If 'Wat is het meest bij u van toepassing ten aanzien van uw acitviteit?' is equal to 'Oriëntatielopen' answer this question:***<br>Oriëntatielopen	◯ Professioneel<br>◯ Competitief<br>◯ Recreatief
4.1.11	***If 'Wat is het meest bij u van toepassing ten aanzien van uw acitviteit?' is equal to 'Rhytmische gymnastiek' answer this question:***<br>Rhytmisch gymnastiek	◯ Professioneel<br>◯ Competitief<br>◯ Recreatief
4.1.12	***If 'Wat is het meest bij u van toepassing ten aanzien van uw acitviteit?' is equal to 'Volleybal' answer this question:***<br>Volleybal	◯ Professioneel<br>◯ Competitief<br>◯ Recreatief
4.1.13	***If 'Wat is het meest bij u van toepassing ten aanzien van uw acitviteit?' is equal to 'Boxen' answer this question:***<br>Boxen	◯ Professioneel<br>◯ Competitief<br>◯ Recreatief
4.1.14	***If 'Wat is het meest bij u van toepassing ten aanzien van uw acitviteit?' is equal to 'Freestyle snowboarden' answer this question:***<br>Freestyle snowboarden	◯ Professioneel<br>◯ Competitief<br>◯ Recreatief
4.1.15	***If 'Wat is het meest bij u van toepassing ten aanzien van uw acitviteit?' is equal to 'Freestyle snowboarden' answer this question:***<br>IJshockey	◯ Professioneel<br>◯ Competitief<br>◯ Recreatief

Paget LDA, *et al. BMJ Open* 2019; 9:e030961. doi: 10.1136/bmjopen-2019-030961

4.1.16	***If 'Wat is het meest bij u van toepassing ten aanzien van uw acitviteit?' is equal to 'Tennis' answer this question:***<br>Tennis	◯ Professioneel<br>◯ Competitief<br>◯ Recreatief
4.1.17	***If 'Wat is het meest bij u van toepassing ten aanzien van uw acitviteit?' is equal to 'Worstelen' answer this question:***<br>Worstelen	◯ Professioneel<br>◯ Competitief<br>◯ Recreatief
4.1.18	***If 'Wat is het meest bij u van toepassing ten aanzien van uw acitviteit?' is equal to 'Fitness, aerobics' answer this question:***<br>Fitness, aerobics	◯ Professioneel<br>◯ Competitief<br>◯ Recreatief
4.1.19	***If 'Wat is het meest bij u van toepassing ten aanzien van uw acitviteit?' is equal to 'Badminton' answer this question:***<br>Badminton	◯ Professioneel<br>◯ Competitief<br>◯ Recreatief
4.1.20	***If 'Wat is het meest bij u van toepassing ten aanzien van uw acitviteit?' is equal to 'Baseball' answer this question:***<br>Baseball	◯ Professioneel<br>◯ Competitief<br>◯ Recreatief
4.1.21	***If 'Wat is het meest bij u van toepassing ten aanzien van uw acitviteit?' is equal to 'Cross-country hardlopen' answer this question:***<br>Cross-country hardlopen	◯ Professioneel<br>◯ Competitief<br>◯ Recreatief
4.1.22	***If 'Wat is het meest bij u van toepassing ten aanzien van uw acitviteit?' is equal to 'Moderne pentathlon' answer this question:***<br>Moderne pentathlon	◯ Professioneel<br>◯ Competitief<br>◯ Recreatief
4.1.23	***If 'Wat is het meest bij u van toepassing ten aanzien van uw acitviteit?' is equal to 'Squash' answer this question:***<br>Squash	◯ Professioneel<br>◯ Competitief<br>◯ Recreatief
4.1.24	***If 'Wat is het meest bij u van toepassing ten aanzien van uw acitviteit?' is equal to 'Surfen, windsurfen' answer this question:***<br>Surfen, windsurfen	◯ Professioneel<br>◯ Competitief<br>◯ Recreatief
4.1.25	***If 'Wat is het meest bij u van toepassing ten aanzien van uw acitviteit?' is equal to 'Tafel tennis' answer this question:***<br>Tafel tennis	◯ Professioneel<br>◯ Competitief<br>◯ Recreatief

Paget LDA, *et al. BMJ Open* 2019; 9:e030961. doi: 10.1136/bmjopen-2019-030961

4.1.26	***If 'Wat is het meest bij u van toepassing ten aanzien van uw acitviteit?' is equal to 'Atletiek: spring-, werponderdelen' answer this question:***<br>Atletiek: spring-, werponderdelen	◯ Professioneel<br>◯ Competitief<br>◯ Recreatief
4.1.27	***If 'Wat is het meest bij u van toepassing ten aanzien van uw acitviteit?' is equal to 'Waterskieën' answer this question:***<br>Waterskien	◯ Professioneel<br>◯ Competitief<br>◯ Recreatief
4.1.28	***If 'Wat is het meest bij u van toepassing ten aanzien van uw acitviteit?' is equal to 'Dans' answer this question:***<br>Dans	◯ Professioneel<br>◯ Competitief<br>◯ Recreatief
4.1.29	***If 'Wat is het meest bij u van toepassing ten aanzien van uw acitviteit?' is equal to 'Schermen' answer this question:***<br>Schermen	◯ Professioneel<br>◯ Competitief<br>◯ Recreatief
4.1.30	***If 'Wat is het meest bij u van toepassing ten aanzien van uw acitviteit?' is equal to 'Zaalhockey' answer this question:***<br>Zaalhockey	◯ Professioneel<br>◯ Competitief<br>◯ Recreatief
4.1.31	***If 'Wat is het meest bij u van toepassing ten aanzien van uw acitviteit?' is equal to 'Bergbeklimmen' answer this question:***<br>Bergbeklimmen	◯ Professioneel<br>◯ Competitief<br>◯ Recreatief
4.1.32	***If 'Wat is het meest bij u van toepassing ten aanzien van uw acitviteit?' is equal to 'Langlauf' answer this question:***<br>Langlauf	◯ Professioneel<br>◯ Competitief<br>◯ Recreatief
4.1.33	***If 'Wat is het meest bij u van toepassing ten aanzien van uw acitviteit?' is equal to 'Parachute springen' answer this question:***<br>Parachute springen	◯ Professioneel<br>◯ Competitief<br>◯ Recreatief
4.1.34	***If 'Wat is het meest bij u van toepassing ten aanzien van uw acitviteit?' is equal to 'Softball' answer this question:***<br>Softball	◯ Professioneel<br>◯ Competitief<br>◯ Recreatief

Paget LDA, *et al. BMJ Open* 2019; 9:e030961. doi: 10.1136/bmjopen-2019-030961

4.1.35	***If 'Wat is het meest bij u van toepassing ten aanzien van uw acitviteit?' is equal to 'Speciale beroepen en werkactiviteiten; speciale beroepen: ballet, professioneel soldaat, speciale reddingswerker, stuntman, etc.' answer this question:***<br>Speciale beroepen en werkactiviteiten speciale beroepen: ballet, professioneel soldaat, speciale reddingswerker, stuntman, etc.	○ ballet, professioneel soldaat, speciale reddingswerker, stuntman, etc.
4.1.36	***If 'Wat is het meest bij u van toepassing ten aanzien van uw acitviteit?' is equal to 'Duiken' answer this question:***<br>Duiken	○ Professioneel/competitief<br>○ Recreatief
4.1.37	***If 'Wat is het meest bij u van toepassing ten aanzien van uw acitviteit?' is equal to 'Scubaduiken' answer this question:***<br>Scubaduiken	○ Professioneel/competitief<br>○ Recreatief
4.1.38	***If 'Wat is het meest bij u van toepassing ten aanzien van uw acitviteit?' is equal to 'Skaten, in-line skaten' answer this question:***<br>skaten, in-linen skaten	○ Professioneel/competitief<br>○ Recreatief
4.1.39	***If 'Wat is het meest bij u van toepassing ten aanzien van uw acitviteit?' is equal to 'Atletiek: looponderdelen' answer this question:***<br>Atletiek: looponderdelen	○ Professioneel/competitief<br>○ Recreatief
4.1.40	***If 'Wat is het meest bij u van toepassing ten aanzien van uw acitviteit?' is equal to 'Triatlon' answer this question:***<br>triatlon	○ Professioneel/competitief<br>○ Recreatief
4.1.41	***If 'Wat is het meest bij u van toepassing ten aanzien van uw acitviteit?' is equal to 'Gewichtsheffen, body-building' answer this question:***<br>Gewichtsheffen, body-building	○ Professioneel/competitief<br>○ Recreatief
4.1.42	***If 'Wat is het meest bij u van toepassing ten aanzien van uw acitviteit?' is equal to 'Alle competitieve sporten hieronder met ‘seasonal’ conditioning' answer this question:***<br>Alle competitieve sporten hieronder met ‘seasonal’ conditioning	○ Alle competitieve sporten hieronder met ‘seasonal’ conditioning
4.1.43	***If 'Wat is het meest bij u van toepassing ten aanzien van uw acitviteit?' is equal to 'Zwaar fysiek werk' answer this question:***<br>Zwaar fysiek werk	○ Alle competitieve sporten hieronder met ‘seasonal’ conditioning

Paget LDA, *et al*. *BMJ Open* 2019; 9:e030961. doi: 10.1136/bmjopen-2019-030961

4.1.44	***If 'Wat is het meest bij u van toepassing ten aanzien van uw acitviteit?' is equal to 'Alpine skiën en snowboarden' answer this question:*** Alpine skien en snowboarden	◯ Alpine skien en snowboarden
4.1.45	***If 'Wat is het meest bij u van toepassing ten aanzien van uw acitviteit?' is equal to 'Bowlen/curlen' answer this question:*** Bowlen/curlen	◯ Bowlen/curlen
4.1.46	***If 'Wat is het meest bij u van toepassing ten aanzien van uw acitviteit?' is equal to 'Golf' answer this question:*** Golf	◯ Golf
4.1.47	***If 'Wat is het meest bij u van toepassing ten aanzien van uw acitviteit?' is equal to 'Mountainbike/BMX' answer this question:*** Mountainbike/BMX	◯ Mountainbike/BMX
4.1.48	***If 'Wat is het meest bij u van toepassing ten aanzien van uw acitviteit?' is equal to 'Powerliften' answer this question:*** Powerliften	◯ Powerliften
4.1.49	***If 'Wat is het meest bij u van toepassing ten aanzien van uw acitviteit?' is equal to 'Zeilen' answer this question:*** Zeilen	◯ Zeilen
4.1.50	***If 'Wat is het meest bij u van toepassing ten aanzien van uw acitviteit?' is equal to 'Fysiek werk' answer this question:*** Fysiek werk	◯ Fysiek werk
4.1.51	***If 'Wat is het meest bij u van toepassing ten aanzien van uw acitviteit?' is equal to 'Wielrennen' answer this question:*** Wielrennen	◯ Wielrennen
4.1.52	***If 'Wat is het meest bij u van toepassing ten aanzien van uw acitviteit?' is equal to 'Paardrijden' answer this question:*** Paardrijden	◯ Paardrijden
4.1.53	***If 'Wat is het meest bij u van toepassing ten aanzien van uw acitviteit?' is equal to 'Motorsporten/technische sporten' answer this question:*** Motorsporten/technische sporten	◯ Motorsporten/technische sporten
4.1.54	***If 'Wat is het meest bij u van toepassing ten aanzien van uw acitviteit?' is equal to 'Roeien, kayakken' answer this question:*** Roeien, kayakken	◯ Roeien, kayakken

Paget LDA, *et al. BMJ Open* 2019; 9:e030961. doi: 10.1136/bmjopen-2019-030961

4.1.55	***If 'Wat is het meest bij u van toepassing ten aanzien van uw acitviteit?' is equal to 'Boogschieten' answer this question:*** Boogschieten	◯ Boogschieten
4.1.56	***If 'Wat is het meest bij u van toepassing ten aanzien van uw acitviteit?' is equal to 'Water polo en zwemmen' answer this question:*** Water polo en zwemmen	◯ Water polo en zwemmen
4.1.57	***If 'Wat is het meest bij u van toepassing ten aanzien van uw acitviteit?' is equal to 'Kunnen lopen op oneven grond' answer this question:*** Kunnen lopen op oneven grond	◯ Kunnen lopen op oneven grond
4.1.58	***If 'Wat is het meest bij u van toepassing ten aanzien van uw acitviteit?' is equal to 'Geen sport, echter geen limitatie in dagelijkse activiteiten' answer this question:*** Geen sport, echter geen limitatie in dagelijkse activiteiten	◯ Geen sport, echter geen limitatie in dagelijkse activiteiten
4.1.59	***If 'Wat is het meest bij u van toepassing ten aanzien van uw acitviteit?' is equal to 'Kunnen lopen op even grond, maar de dagelijkste activiteiten zijn gelimiteerd' answer this question:*** Kunnen lopen op even grond, maar de dagelijkste activiteiten zijn gelimiteerd	◯ Kunnen lopen op even grond, maar de dagelijkste activiteiten zijn gelimiteerd
4.1.60	***If 'Wat is het meest bij u van toepassing ten aanzien van uw acitviteit?' is equal to 'Niet kunnen lopen, fysieke handicap wegens enkelproblemen' answer this question:*** Niet kunnen lopen, fysieke handicap wegens enkelproblemen	◯ Niet kunnen lopen, fysieke handicap wegens enkelproblemen

Vragenlijst PRIMA studie 12 weken - Hoe tevreden bent u over de enkelklachten?

Number	Question	Answers
5.1	Hoe tevreden bent u over de enkelklachten?	◯ Slecht<br>◯ Matig<br>◯ Goed<br>◯ Uitstekend

Paget LDA, *et al. BMJ Open* 2019; 9:e030961. doi: 10.1136/bmjopen-2019-030961

Vragenlijst PRIMA studie 12 weken - SF-36

Number	Question	Answers
	Deze vragenlijst gaat over uw standpunten t.a.v. uw gezondheid. Met behulp van deze gegevens kan worden bijgehouden hoe u zich voelt en hoe goed u in staat bent uw gebruikelijke bezigheden uit te voeren. Beantwoord elke vraag door een antwoord aan te klikken. Als u niet zeker weet hoe u een vraag moet beantwoorden, geef dan het best mogelijke antwoord.	
6.1	Hoe zou u over het algemeen uw gezondheid noemen?	◯ Uitstekend ◯ Zeer goed ◯ Goed ◯ Matig ◯ Slecht
6.2	Hoe beoordeelt u nu uw gezondheid over het algemeen vergeleken met een jaar geleden?	◯ Veel beter dan een jaar geleden ◯ Wat beter dan een jaar geleden ◯ Ongeveer hetzelfde als een jaar geleden ◯ Wat slechter dan een jaar geleden ◯ Veel slechter dan een jaar geleden
6.3	Wordt u door uw gezondheid op dit moment beperkt bij forse inspanning, zoals hardlopen, tillen van zware voorwerpen of een veeleisende sport beoefenen?	◯ Ja, ernstig beperkt ◯ Ja, een beetje beperkt ◯ Nee, helemaal niet beperkt
6.4	Wordt u door uw gezondheid op dit moment beperkt bij matige inspanning zoals een tafel verplaatsen, stofzuigen, zwemmen of fietsen?	◯ Ja, ernstig beperkt ◯ Ja, een beetje beperkt ◯ Nee, helemaal niet beperkt
6.5	Wordt u door uw gezondheid op dit moment beperkt bij boodschappen tillen of dragen?	◯ Ja, ernstig beperkt ◯ Ja, een beetje beperkt ◯ Nee, helemaal niet beperkt
6.6	Wordt u door uw gezondheid op dit moment beperkt bij een paar trappen oplopen?	◯ Ja, ernstig beperkt ◯ Ja, een beetje beperkt ◯ Nee, helemaal niet beperkt
6.7	Wordt u door uw gezondheid op dit moment beperkt bij één trap oplopen?	◯ Ja, ernstig beperkt ◯ Ja, een beetje beperkt ◯ Nee, helemaal niet beperkt
6.8	Wordt u door uw gezondheid op dit moment beperkt bij bukken, knielen of hurken?	◯ Ja, ernstig beperkt ◯ Ja, een beetje beperkt ◯ Nee, helemaal niet beperkt
6.9	Wordt u door uw gezondheid op dit moment beperkt bij meer dan één kilometer lopen?	◯ Ja, ernstig beperkt ◯ Ja, een beetje beperkt ◯ Nee, helemaal niet beperkt
6.10	Wordt u door uw gezondheid op dit moment beperkt bij een paar honderd meter lopen?	◯ Ja, ernstig beperkt ◯ Ja, een beetje beperkt ◯ Nee, helemaal niet beperkt

Paget LDA, *et al. BMJ Open* 2019; 9:e030961. doi: 10.1136/bmjopen-2019-030961

6.11	Wordt u door uw gezondheid op dit moment beperkt bij ongeveer honderd meter lopen?	◯ Ja, ernstig beperkt ◯ Ja, een beetje beperkt ◯ Nee, helemaal niet beperkt
6.12	Wordt u door uw gezondheid op dit moment beperkt bij uzelf wassen of aankleden?	◯ Ja, ernstig beperkt ◯ Ja, een beetje beperkt ◯ Nee, helemaal niet beperkt
6.13	U besteedde in de afgelopen 4 weken minder tijd aan werk of andere bezigheden	◯ Ja ◯ Nee
6.14	U heeft in de afgelopen 4 weken minder bereikt dan u zou willen	◯ Ja ◯ Nee
6.15	U was in de afgelopen 4 weken beperkt in het soort werk of andere bezigheden.	◯ Ja ◯ Nee
6.16	U had de afgelopen 4 weken moeite om uw werk of andere bezigheden uit te voeren (het kostte u bijvoorbeeld extra inspanning).	◯ Ja ◯ Nee
6.17	U besteedde in de afgelopen 4 weken minder tijd aan werk of andere bezigheden ten gevolge van emotionele problemen	◯ Ja ◯ Nee
6.18	U heeft in de afgelopen 4 weken minder bereikt dan u zou willen ten gevolge van emotionele problemen.	◯ Ja ◯ Nee
6.19	U deed de afgelopen 4 weken uw werk of andere bezigheden niet zo zorgvuldig als gewoonlijk ten gevolge van emotionele problemen.	◯ Ja ◯ Nee
6.20	In hoeverre hebben uw lichamelijke gezondheid of emotionele problemen u gedurende de afgelopen 4 weken gehinderd in uw normale omgang met familie, vrienden of buren, of bij activiteiten in groepsverband?	◯ Helemaal niet ◯ Enigszins ◯ Nogal ◯ Veel ◯ Heel erg veel
6.21	Hoeveel lichamelijke pijn heeft u de afgelopen 4 weken gehad?	◯ Geen<br>◯ Heel licht<br>◯ Licht<br>◯ Nogal<br>◯ Ernstig<br>◯ Heel ernstig
6.22	In welke mate bent u de afgelopen 4 weken door de pijn gehinderd in uw normale werk (zowel werk buitenshuis als huishoudelijk werk)?	◯ Helemaal niet<br>◯ Een klein beetje<br>◯ Nogal<br>◯ Veel<br>◯ Heel erg veel

Paget LDA, *et al. BMJ Open* 2019; 9:e030961. doi: 10.1136/bmjopen-2019-030961

6.23	Voelde u zich levenslustig?	◯ Altijd<br>◯ Meestal<br>◯ Vaak<br>◯ Soms<br>◯ Zelden<br>◯ Nooit
6.24	Was u erg zenuwachtig?	◯ Altijd<br>◯ Meestal<br>◯ Vaak<br>◯ Soms<br>◯ Zelden<br>◯ Nooit
6.25	Zat u zo in de put dat u niets kon?	◯ Altijd<br>◯ Meestal<br>◯ Vaak<br>◯ Soms<br>◯ Zelden<br>◯ Nooit
6.26	Voelde u zich rustig en tevreden?	◯ Altijd<br>◯ Meestal<br>◯ Vaak<br>◯ Soms<br>◯ Zelden<br>◯ Nooit
6.27	Had u veel energie?	◯ Altijd<br>◯ Meestal<br>◯ Vaak<br>◯ Soms<br>◯ Zelden<br>◯ Nooit
6.28	Voelde u zich somber en neerslachtig?	◯ Altijd<br>◯ Meestal<br>◯ Vaak<br>◯ Soms<br>◯ Zelden<br>◯ Nooit

Paget LDA, *et al. BMJ Open* 2019; 9:e030961. doi: 10.1136/bmjopen-2019-030961

6.29	Voelde u zich uitgeput?	◯ Altijd<br>◯ Meestal<br>◯ Vaak<br>◯ Soms<br>◯ Zelden<br>◯ Nooit
6.30	Was u een gelukkig mens?	◯ Altijd<br>◯ Meestal<br>◯ Vaak<br>◯ Soms<br>◯ Zelden<br>◯ Nooit
6.31	Voelde u zich moe?	◯ Altijd<br>◯ Meestal<br>◯ Vaak<br>◯ Soms<br>◯ Zelden<br>◯ Nooit
6.32	Hoe vaak hebben uw lichamelijke gezondheid of emotionele problemen u gedurende de afgelopen 4 weken gehinderd bij uw sociale activiteiten (zoals vrienden of familie bezoeken etc.)?	◯ Altijd<br>◯ Meestal<br>◯ Soms<br>◯ Zelden<br>◯ Nooit
6.33	Ik lijk wat gemakkelijker ziek te worden dan andere mensen	◯ Volkomen juist<br>◯ Grotendeels juist<br>◯ Weet ik niet<br>◯ Grotendeels onjuist<br>◯ Volkomen onjuist
6.34	Ik ben even gezond als andere mensen die ik ken	◯ Volkomen juist<br>◯ Grotendeels juist<br>◯ Weet ik niet<br>◯ Grotendeels onjuist<br>◯ Volkomen onjuist

Paget LDA, *et al. BMJ Open* 2019; 9:e030961. doi: 10.1136/bmjopen-2019-030961

6.35	Ik verwacht dat mijn gezondheid achteruit zal gaan	○ Volkomen juist<br>○ Grotendeels juist<br>○ Weet ik niet<br>○ Grotendeels onjuist<br>○ Volkomen onjuist
6.36	Mijn gezondheid is uitstekend	○ Volkomen juist<br>○ Grotendeels juist<br>○ Weet ik niet<br>○ Grotendeels onjuist<br>○ Volkomen onjuist

Vragenlijst PRIMA studie 12 weken - GAS

Number	Question	Answers
	Tijdens de eerste afspraak werd een duidelijk doel met u afgesproken. Dit werd tevens per email naar u gestuurd. Hoe is de situatie nu ten aanzien van de destijds gestelde doelstelling?	
7.1	Wat is nu de situatie ten aanzien van het tijdens de eerste afspraak afgesproken doelstelling (Goal attainment Scaling)?	○ Achteruitgang (minder dan de utigangssituatie)<br>○ Uitgangssituatie<br>○ Minder dan het doel<br>○ Doel<br>○ Meer dan het doel<br>○ Veel meer dan het doel

Vragenlijst PRIMA studie 12 weken - EQ-5D-3L

Number	Question	Answers
	Deze vragenlijst gaat over uw standpunten t.a.v. uw gezondheid. Met behulp van deze gegevens kan worden bijgehouden hoe u zich voelt en hoe goed u in staat bent uw gebruikelijke bezigheden uit te voeren. Beantwoord elke vraag door een antwoord aan te klikken. Als u niet zeker weet hoe u een vraag moet beantwoorden, geef dan het best mogelijke antwoord.	
8.1	Hoe is het met uw Mobiliteit gesteld?	○ Ik heb geen problemen met lopen<br>○ Ik heb enige problemen met lopen<br>○ Ik ben bedlegerig

Paget LDA, *et al. BMJ Open* 2019; 9:e030961. doi: 10.1136/bmjopen-2019-030961

8.2	Hoe is het met uw Zelfzorg gesteld?	◯ Ik heb geen problemen om mijzelf te wassen of aan te kleden<br>◯ Ik heb enige problemen om mijzelf te wassen of aan te kleden<br>◯ Ik ben niet in staat om mijzelf te wassen of aan te kleden
8.3	Hoe is het met de Dagelijkse activiteiten (werk, studie, huishouden, gezins- en vrijetijdsactiviteiten) gesteld?	◯ Ik heb geen problemen met mijn dagelijkse activiteiten<br>◯ Ik heb enige problemen met mijn dagelijkse activiteiten<br>◯ Ik ben niet in staat om mijn dagelijkse activiteiten uit te voeren
8.4	Hoe is het met de Pijn/klachten gesteld?	◯ Ik heb geen pijn of andere klachten<br>◯ Ik heb matige pijn of andere klachten<br>◯ Ik heb zeer ernstige pijn of andere klachten
8.5	Hoe is het met de Stemming gesteld?	◯ Ik ben niet angstig of somber<br>◯ Ik ben matig angstig of somber<br>◯ Ik ben erg angstig of somber
	We willen weten hoe goed of slecht uw gezondheid VANDAAG is. Deze meetschaal loopt van 0 tot 100: 100 staat voor de BESTE gezondheid die u zich kunt voorstellen - 0 staat voor de SLECHTSTE gezondheid die u zich kunt voorstellen	
8.6	Uw Gezondheid vandaag	(0.00) (100.00)

Vragenlijst PRIMA studie 12 weken - AOS

Number	Question	Answers
	Instructies: De lijn naast elke vraag staat voor hoeveel PIJN u heeft in verschillende situaties. De linker kant (0) is “geen pijn” en de rechter kant (100) is “ergste pijn denkbaar”. Geef voor de onderstaande situaties op de lijn aan hoeveel PIJN u in de afgelopen week in de enkel had. Als een of meerdere van deze situaties niet van toepassing waren, dan kiest u de "niet van toepassing" optie.	
9.1	Wat was de hoogte van de ergste pijn in de afgelopen week?	(0.00) (100.00)
9.2	Heeft u pijn als u 's ochtends opstaat?	◯ Ja<br>◯ Nee<br>◯ Niet van toepassing

Paget LDA, *et al*. *BMJ Open* 2019; 9:e030961. doi: 10.1136/bmjopen-2019-030961

9.2.1	***If 'Heeft u pijn als u 's ochtends opstaat?' is equal to 'Ja' answer this question:***<br>Hoeveel pijn heeft u voordat u 's ochtends opstaat?	(0.00)	(100.00)
9.3	Heeft u pijn wanneer u op blote voeten loopt?	◯ Ja<br>◯ Nee<br>◯ Niet van toepassing	
9.3.1	***If 'Heeft u pijn wanneer u op blote voeten loopt?' is equal to 'Ja' answer this question:***<br>Hoeveel pijn heeft u als u op blote voeten loopt?	(0.00)	(100.00)
9.4	Heeft u pijn wanneer u op blote voeten staat?	◯ Ja<br>◯ Nee<br>◯ Niet van toepassing	
9.4.1	***If 'Heeft u pijn wanneer u op blote voeten staat?' is equal to 'Ja' answer this question:***<br>Hoeveel pijn heeft u als u op blote voeten staat?	(0.00)	(100.00)
9.5	Heeft u pijn wanneer u met schoenen loopt?	◯ Ja<br>◯ Nee<br>◯ Niet van toepassing	
9.5.1	***If 'Heeft u pijn wanneer u met schoenen loopt?' is equal to 'Ja' answer this question:***<br>Hoeveel pijn heeft u wanneer u met schoenen loopt?	(0.00)	(100.00)
9.6	Heeft u pijn wanneer u met schoenen staat?	◯ Ja<br>◯ Nee<br>◯ Niet van toepassing	
9.6.1	***If 'Heeft u pijn wanneer u met schoenen staat?' is equal to 'Ja' answer this question:***<br>Hoeveel pijn heeft u wanneer u met schoenen staat?	(0.00)	(100.00)
9.7	Heeft u pijn wanneer u loopt met steunzolen of een brace?	◯ Ja<br>◯ Nee<br>◯ Niet van toepassing	
9.7.1	***If 'Heeft u pijn wanneer u loopt met steunzolen of een brace?' is equal to 'Ja' answer this question:***<br>Hoeveel pijn heeft u wanneer u loopt met steunzolen of een brace?	(0.00)	(100.00)

Paget LDA, *et al. BMJ Open* 2019; 9:e030961. doi: 10.1136/bmjopen-2019-030961

9.8	Heeft u pijn wanneer u staat met steunzolen of een brace?	◯ Ja<br>◯ Nee<br>◯ Niet van toepassing	
9.8.1	***If 'Heeft u pijn wanneer u staat met steunzolen of een brace?' is equal to 'Ja' answer this question:***<br>Hoeveel pijn heeft u wanneer u staat met steunzolen of een brace?	(0.00)	(100.00)
9.9	Heeft u pijn aan het einde van de dag?	◯ Ja<br>◯ Nee<br>◯ Niet van toepassing	
9.9.1	***If 'Heeft u pijn aan het einde van de dag?' is equal to 'Ja' answer this question:***<br>Hoeveel pijn heeft u aan het einde van de dag?	(0.00)	(100.00)
	Instructies:De lijn naast elke vraag staat voor hoeveel MOEITE u heeft met verschillende activiteiten. De linker kant (0) is “Niet moeilijk” en de rechter kant (100) is “Te moeilijk, niet uitvoerbaar”. Geef voor de onderstaande activiteiten op de lijn aan hoeveel MOEITE u in de afgelopen week door enkelklachten had met onderstaande activiteiten. Als een situatie niet van toepassing is, kies dan de optie "niet van toepassing".		
9.10	Heeft u moeite met door het huis lopen?	◯ Ja<br>◯ Nee<br>◯ Niet van toepassing	
9.10.1	***If 'Heeft u moeite met door het huis lopen?' is equal to 'Ja' answer this question:***<br>Hoeveel moeite heeft u met door het huis lopen?	(0.00)	(100.00)
9.11	Heeft u moeite met buiten lopen op oneven ondergrond?	◯ Ja<br>◯ Nee<br>◯ Niet van toepassing	
9.11.1	***If 'Heeft u moeite met buiten lopen op oneven ondergrond?' is equal to 'Ja' answer this question:***<br>Hoeveel moeite heeft u met buiten lopen op oneven ondergrond?	(0.00)	(100.00)
9.12	Heeft u moeite met een paar honderd meter lopen?	◯ Ja<br>◯ Nee<br>◯ Niet van toepassing	
9.12.1	***If 'Heeft u moeite met een paar honderd meter lopen?' is equal to 'Ja' answer this question:***<br>Hoeveel moeite heeft u met een paar honderd meter lopen?	(0.00)	(100.00)

Paget LDA, *et al*. *BMJ Open* 2019; 9:e030961. doi: 10.1136/bmjopen-2019-030961

9.13	Heeft u moeite met een trap oplopen?	◯ Ja<br>◯ Nee<br>◯ Niet van toepassing	
9.13.1	***If 'Heeft u moeite met een trap oplopen?' is equal to 'Ja' answer this question:***<br>Hoeveel moeite heeft u met een trap oplopen?	(0.00)	(100.00)
9.14	Heeft u moeite met een trap aflopen?	◯ Ja<br>◯ Nee<br>◯ Niet van toepassing	
9.14.1	***If 'Heeft u moeite met een trap aflopen?' is equal to 'Ja' answer this question:***<br>Hoeveel moeite heeft u met een trap aflopen?	(0.00)	(100.00)
9.15	Heeft u moeite met op de tenen staan?	◯ Ja<br>◯ Nee<br>◯ Niet van toepassing	
9.15.1	***If 'Heeft u moeite met op de tenen staan?' is equal to 'Ja' answer this question:***<br>Hoeveel moeite heeft u met op de tenen staan?	(0.00)	(100.00)
9.16	Heeft u moeite met opstaan uit de stoel?	◯ Ja<br>◯ Nee<br>◯ Niet van toepassing	
9.16.1	***If 'Heeft u moeite met opstaan uit de stoel?' is equal to 'Ja' answer this question:***<br>Hoeveel moeite heeft u met opstaan uit de stoel?	(0.00)	(100.00)
9.17	Heeft u moeite met het op- of afstappen van stoepranden?	◯ Ja<br>◯ Nee<br>◯ Niet van toepassing	
9.17.1	***If 'Heeft u moeite met het op- of afstappen van stoepranden?' is equal to 'Ja' answer this question:***<br>Hoeveel moeite heeft u met het op- of afstappen van stoepranden?	(0.00)	(100.00)
9.18	Heeft u moeite met snel lopen of rennen?	◯ Ja<br>◯ Nee<br>◯ Niet van toepassing	

Paget LDA, *et al. BMJ Open* 2019; 9:e030961. doi: 10.1136/bmjopen-2019-030961

9.18.1	***If 'Heeft u moeite met snel lopen of rennen?' is equal to 'Ja' answer this question:*** Hoeveel moeite heeft u met snel lopen of rennen?	(0.00)	(100.00)

Vragenlijst PRIMA studie 12 weken - FAOS

Number	Question	Answers
	Deze lijst vraagt naar uw mening over uw voet/enkel. Uw antwoorden geven ons een beeld van uw voet/enkel klachten en hoe u in staat bent om alledaagse activiteiten uit te voeren in uw huidige situatie. Beantwoorden van een vraag doet u door het aanklikken van een vakje met het volgens u meest juiste antwoord (één vakje per vraag). Als u niet zeker weet hoe u een vraag moet beantwoorden, geeft u dan het antwoord dat volgens u het meest op uw situatie van toepassing is. Deze vraag heeft betrekking op het voorkomen van voet/enkel klachten in de afgelopen week.	
10.1	Is uw voet/enkel gezwollen?	◯ Nooit ◯ Zelden ◯ Soms ◯ Vaak ◯ Altijd
10.2	Voelt u gekraak of hoort u klikken of een ander vreemd geluid wanneer u de voet/enkel beweegt?	◯ Nooit ◯ Zelden ◯ Soms ◯ Vaak ◯ Altijd
10.3	Hapert uw enkel of blokkeert uw enkel ('op slot' gaan zitten) wanneer u deze beweegt?	◯ Nooit ◯ Zelden ◯ Soms ◯ Vaak ◯ Altijd
10.4	Kunt u de voet/enkel volledig strekken?	◯ Altijd ◯ Vaak ◯ Soms ◯ Zelden ◯ Nooit
10.5	Kunt u de voet volledig naar u toe buigen?	◯ Altijd ◯ Vaak ◯ Soms ◯ Zelden ◯ Nooit
10.6	In welke mate heeft u een stijf gevoel in de voet/enkel 's ochtends bij het wakker worden?	◯ Niet ◯ Mild ◯ Matig ◯ Ernstig ◯ Zeer ernstig
10.7	In welke mate heeft u een stijf gevoel in de voet/enkel na zitten, liggen of rusten later op de dag?	◯ Niet ◯ Mild ◯ Matig ◯ Ernstig ◯ Zeer ernstig
10.8	Hoe vaak heeft u pijn in uw voet/enkel?	◯ Nooit ◯ Maandelijks ◯ Wekelijks ◯ Dagelijks ◯ Altijd

Paget LDA, *et al. BMJ Open* 2019; 9:e030961. doi: 10.1136/bmjopen-2019-030961

10.9	Hoeveel voet/enkel pijn heeft u gehad in de afgelopen week bij draaien als uw voet/enkel op de grond staat?	◯ Geen<br>◯ Mild<br>◯ Matig<br>◯ Ernstig<br>◯ Zeer ernstig
10.10	Hoeveel voet/enkel pijn heeft u gehad in de afgelopen week bij het volledig uitstrekken van de voet/enkel?	◯ Geen<br>◯ Mild<br>◯ Matig<br>◯ Ernstig<br>◯ Zeer ernstig
10.11	Hoeveel voet/enkel pijn heeft u gehad in de afgelopen week bij het volledig naar u toe buigen/optrekken van de voet/enkel	◯ Geen<br>◯ Mild<br>◯ Matig<br>◯ Ernstig<br>◯ Zeer ernstig
10.12	Hoeveel voet/enkel pijn heeft u gehad in de afgelopen week bij het lopen op een vlakke ondergrond	◯ Geen<br>◯ Mild<br>◯ Matig<br>◯ Ernstig<br>◯ Zeer ernstig
10.13	Hoeveel voet/enkel pijn heeft u gehad in de afgelopen week bij het trap op- en trap af lopen?	◯ Geen<br>◯ Mild<br>◯ Matig<br>◯ Ernstig<br>◯ Zeer ernstig
10.14	Hoeveel voet/enkel pijn heeft u gehad in de afgelopen week 's nachts in bed?	◯ Geen<br>◯ Mild<br>◯ Matig<br>◯ Ernstig<br>◯ Zeer ernstig
10.15	Hoeveel voet/enkel pijn heeft u gehad in de afgelopen week bij het zitten of liggen?	◯ Geen<br>◯ Mild<br>◯ Matig<br>◯ Ernstig<br>◯ Zeer ernstig

Paget LDA, *et al. BMJ Open* 2019; 9:e030961. doi: 10.1136/bmjopen-2019-030961

10.16	Hoeveel voet/enkel pijn heeft u gehad in de afgelopen week bij het rechtop staan?	◯ Geen<br>◯ Mild<br>◯ Matig<br>◯ Ernstig<br>◯ Zeer ernstig
10.17	In welke mate werd u gehinderd bij het trap aflopen?	◯ Niet ◯ Mild ◯ Matig ◯ Ernstig ◯ Zeer ernstig
10.18	In welke mate werd u gehinderd bij het trap op lopen?	◯ Niet ◯ Mild ◯ Matig ◯ Ernstig ◯ Zeer ernstig
10.19	In welke mate werd u gehinderd als u vanuit een zittende positie ging staan?	◯ Niet ◯ Mild ◯ Matig ◯ Ernstig ◯ Zeer ernstig
10.20	In welke mate werd u gehinderd bij het staan?	◯ Niet ◯ Mild ◯ Matig ◯ Ernstig ◯ Zeer ernstig
10.21	In welke mate werd u gehinderd bij het naar de grond buigen/iets oprapen?	◯ Niet ◯ Mild ◯ Matig ◯ Ernstig ◯ Zeer ernstig
10.22	In welke mate werd u gehinderd bij het lopen op een vlakke ondergrond?	◯ Niet ◯ Mild ◯ Matig ◯ Ernstig ◯ Zeer ernstig
10.23	In welke mate werd u gehinderd bij het in- en uit de auto stappen?	◯ Niet ◯ Mild ◯ Matig ◯ Ernstig ◯ Zeer ernstig
10.24	In welke mate werd u gehinderd bij het boodschappen doen?	◯ Niet ◯ Mild ◯ Matig ◯ Ernstig ◯ Zeer ernstig
10.25	In welke mate werd u gehinderd bij sokken/panty's aantrekken?	◯ Niet ◯ Mild ◯ Matig ◯ Ernstig ◯ Zeer ernstig
10.26	In welke mate werd u gehinderd bij het opstaan uit bed?	◯ Niet ◯ Mild ◯ Matig ◯ Ernstig ◯ Zeer ernstig
10.27	In welke mate werd u gehinderd bij het sokken uittrekken?	◯ Niet ◯ Mild ◯ Matig ◯ Ernstig ◯ Zeer ernstig
10.28	In welke mate werd u gehinderd bij het in bed liggen (omdraaien, lange tijd uw voet/enkel in dezelfde positie houden)?	◯ Niet ◯ Mild ◯ Matig ◯ Ernstig ◯ Zeer ernstig

Paget LDA, *et al. BMJ Open* 2019; 9:e030961. doi: 10.1136/bmjopen-2019-030961

10.29	In welke mate werd u gehinderd bij het in/uit bad stappen?	◯ Niet ◯ Mild ◯ Matig ◯ Ernstig ◯ Zeer ernstig
10.30	In welke mate werd u gehinderd bij zitten?	◯ Niet ◯ Mild ◯ Matig ◯ Ernstig ◯ Zeer ernstig
10.31	In welke mate werd u gehinderd bij het toilet op en af gaan?	◯ Niet ◯ Mild ◯ Matig ◯ Ernstig ◯ Zeer ernstig
10.32	In welke mate werd u gehinderd bij zwaar huishoudelijk werk (bijvoorbeeld zware dozen sjouwen, vloer schrobben)?	◯ Niet ◯ Mild ◯ Matig ◯ Ernstig ◯ Zeer ernstig
10.33	In welke mate werd u gehinderd bij licht huishoudeijk werk (bijvoorbeeld koken, afstoffen)?	◯ Niet ◯ Mild ◯ Matig ◯ Ernstig ◯ Zeer ernstig
10.34	In welke mate werd u gehinderd bij hurken?	◯ Niet ◯ Mild ◯ Matig ◯ Ernstig ◯ Zeer ernstig
10.35	In welke mate werd u gehinderd bij hardlopen?	◯ Niet ◯ Mild ◯ Matig ◯ Ernstig ◯ Zeer ernstig
10.36	In welke mate werd u gehinderd bij springen?	◯ Niet ◯ Mild ◯ Matig ◯ Ernstig ◯ Zeer ernstig
10.37	In welke mate werd u gehinderd bij ronddraaien op uw aangedane voet/enkel?	◯ Niet ◯ Mild ◯ Matig ◯ Ernstig ◯ Zeer ernstig
10.38	In welke mate werd u gehinderd bij knielen?	◯ Niet ◯ Mild ◯ Matig ◯ Ernstig ◯ Zeer ernstig
10.39	Hoe vaak bent u zich bewust van uw voet/enkel probleem?	◯ Nooit ◯ Maandelijks ◯ Wekelijks ◯ Dagelijks ◯ Altijd
10.40	Heeft u uw leven veranderd om activiteiten te vermijden die schadelijk kunnen zijn voor uw voet/enkel?	◯ Niet<br>◯ Enigszins<br>◯ Matig<br>◯ Behoorlijk<br>◯ Volledig

Paget LDA, *et al. BMJ Open* 2019; 9:e030961. doi: 10.1136/bmjopen-2019-030961

10.41	In hoeverre kunt u op uw voet/enkel vertrouwen?	◯ Volledig<br>◯ Behoorlijk<br>◯ Matig<br>◯ Enigzins<br>◯ Niet
10.42	In het algemeen, in welke mate ondervindt u hinder van uw voet/enkel	◯ Geen<br>◯ Mild<br>◯ Matig<br>◯ Ernstig<br>◯ Zeer ernstig

Vragenlijst PRIMA studie 12 weken - PRODISQ - Gezondheid en werk

Number	Question	Answers
11.1	Wat is uw leeftijd?	[ ] jaar
11.2	Wat is uw geslacht?	◯ Man<br>◯ Vrouw
11.3	Wat is de hoogste opleiding die u heeft afgemaakt (Zoek uw hoogste opleiding en kruis het hokje daarvoor aan)?	☐ Ik heb geen school of opleiding afgemaakt<br>☐ Lagere school of basisschool<br>☐ Huishoudschool, vbo, lbo, lts, leao of lhno<br>☐ Mavo, mulo, ivo of vmbo<br>☐ Mbo, mts, meao, mhno, inas of intas<br>☐ Havo, vwo, hbs, mms, atheneum of gymnasium<br>☐ Hbo, hts, heao of hhno<br>☐ Universiteit<br>☐ Ik heb een andere opleiding afgemaakt, namelijk
11.3.1	***If 'Wat is de hoogste opleiding die u heeft afgemaakt (Zoek uw hoogste opleiding en kruis het hokje daarvoor aan)?' is equal to 'Ik heb een andere opleiding afgemaakt, namelijk' answer this question:***<br>Wat is de hoogste opleiding die u heeft afgemaakt?	[ ]

Paget LDA, *et al. BMJ Open* 2019; 9:e030961. doi: 10.1136/bmjopen-2019-030961

11.4	Wat doet u in het dagelijks leven?	☐ Ik zit op school, ik studeer<br>☐ Ik werk in loondienst<br>☐ Ik ben zelfstandig ondernemer<br>☐ Ik ben huisvrouw, huisman<br>☐ Ik ben werkloos<br>☐ Ik ben arbeidsongeschikt<br>☐ Ik ben met pensioen of prepensioen<br>☐ Ik doe iets anders, namelijk
11.4.1	***If 'Wat doet u in het dagelijks leven?' is equal to 'Ik ben arbeidsongeschikt' answer this question:***<br>Wat doet u in het dagelijks leven?	
11.4.2	***If 'Wat doet u in het dagelijks leven?' is equal to 'Ik doe iets anders, namelijk' answer this question:***<br>Wat doet u in het dagelijks leven?	
11.5	Hebt u betaald werk?	○ Nee<br>○ Ja
11.5.1	***If 'Hebt u betaald werk?' is equal to 'Ja' answer this question:***<br>Wat is uw beroep?	
11.5.2	***If 'Hebt u betaald werk?' is equal to 'Ja' answer this question:***<br>Hoeveel uur per week werkt u (Tel alleen de uren waarvoor u betaald wordt)?	uren
11.5.3	***If 'Hebt u betaald werk?' is equal to 'Ja' answer this question:***<br>Hoeveel dagen in de week werkt u?	Dagen
11.5.4	***If 'Hebt u betaald werk?' is equal to 'Ja' answer this question:***<br>Bent u in de afgelopen 4 weken afwezig geweest van uw werk omdat u ziek was?	○ Nee<br>○ Ja
11.5.4.1	***If 'Bent u in de afgelopen 4 weken afwezig geweest van uw werk omdat u ziek was?' is equal to 'Ja' answer this question:***<br>Bent u in de afgelopen 4 weken afwezig geweest van uw werk omdat u ziek was (Tel alleen de werkdagen in de afgelopen 4 weken)?	dagen afwezig geweest
11.5.4.2	***If 'Bent u in de afgelopen 4 weken afwezig geweest van uw werk omdat u ziek was?' is equal to 'Ja' answer this question:***<br>Was u langer dan de gehele periode van 4 weken afwezig van uw werk doordat u ziek was?	○ Nee<br>○ Ja

Paget LDA, *et al. BMJ Open* 2019; 9:e030961. doi: 10.1136/bmjopen-2019-030961

11.5.4.2.1	***If 'Was u langer dan de gehele periode van 4 weken afwezig van uw werk doordat u ziek was?' is equal to 'Ja' answer this question:*** Wanneer heeft u zich ziek gemeld?	___ ___ ___ *(dd-mm-yyyy)*
11.5.5	***If 'Hebt u betaald werk?' is equal to 'Ja' answer this question:*** Waren er in de afgelopen 4 weken, dagen waarop u wel gewerkt heeft, maar tijdens uw werk last had van lichamelijke of psychische problemen?	◯ Ja<br>◯ Nee
11.5.5.1	***If 'Waren er in de afgelopen 4 weken, dagen waarop u wel gewerkt heeft, maar tijdens uw werk last had van lichamelijke of psychische problemen?' is equal to 'Ja' answer this question:*** Op hoeveel werkdagen had u tijdens uw werk last van uw lichamelijke of psychische problemen?	___ werkdagen
11.5.5.2	***If 'Waren er in de afgelopen 4 weken, dagen waarop u wel gewerkt heeft, maar tijdens uw werk last had van lichamelijke of psychische problemen?' is equal to 'Ja' answer this question:*** Op de dagen dat u last had, kon u misschien niet zoveel werk doen als normaal. Hoeveel werk kon u op deze dagen gemiddeld doen?	Ik kon op deze dagen niks doen (0.00) — Ik kon net zoveel doen als normaal (10.00)
11.6	Waren er dagen waarop u minder onbetaald werk kon doen door uw lichamelijke of psychische problemen?	◯ Nee<br>◯ Ja
11.6.1	***If 'Waren er dagen waarop u minder onbetaald werk kon doen door uw lichamelijke of psychische problemen?' is equal to 'Ja' answer this question:*** Op hoeveel dagen was dit zo?	___ Dagen
11.6.2	***If 'Waren er dagen waarop u minder onbetaald werk kon doen door uw lichamelijke of psychische problemen?' is equal to 'Ja' answer this question:*** Stel dat iemand, bijvoorbeeld uw partner, familielid of een bekende, u op deze dagen had geholpen. En al het onbetaalde werk wat u niet kon doen, voor u had gedaan. Hoeveel uur was die persoon hier op deze dagen dan gemiddeld mee bezig geweest?	___ uur op deze dagen

Vragenlijst PRIMA studie 12 weken - PRODISQ - Zorggebruik

Number	Question	Answers
	Wij willen graag weten met welke dokters u in de afgelopen 3 maanden een afspraak had. Het gaat om afspraken voor uzelf. Ook andere zorgverleners tellen mee. Bijvoorbeeld de fysiotherapeut.	

Paget LDA, *et al. BMJ Open* 2019; 9:e030961. doi: 10.1136/bmjopen-2019-030961

12.1	Bent u in de afgelopen 3 maanden naar uw huisarts geweest?	◯ Nee<br>◯ Ja
12.1.1	***If 'Bent u in de afgelopen 3 maanden naar uw huisarts geweest?' is equal to 'Ja' answer this question:***<br>Hoeveel afspraken had u?	[ ] afspraken
12.2	Bent u in de afgelopen 3 maanden in contact geweest met een maatschappelijk werker?	◯ Nee<br>◯ Ja
12.2.1	***If 'Bent u in de afgelopen 3 maanden in contact geweest met een maatschappelijk werker?' is equal to 'Ja' answer this question:***<br>Hoeveel afspraken had u?	[ ] afspraken
12.3	Bent u in de afgelopen 3 maanden naar een fysiotherapeut geweest? Of een caesartherapeut, therapeut mensendieck of een manueel therapeut?	◯ Nee<br>◯ Ja
12.3.1	***If 'Bent u in de afgelopen 3 maanden naar een fysiotherapeut geweest? Of een caesartherapeut, therapeut mensendieck of een manueel therapeut?' is equal to 'Ja' answer this question:***<br>Hoeveel afspraken had u?	[ ] afspraken
12.4	Bent u in de afgelopen 3 maanden naar een ergotherapeut geweest?	◯ Nee<br>◯ Ja
12.4.1	***If 'Bent u in de afgelopen 3 maanden naar een ergotherapeut geweest?' is equal to 'Ja' answer this question:***<br>Hoeveel afspraken had u?	[ ] afspraken
12.5	Bent u in de afgelopen 3 maanden naar een logopedist geweest?	◯ Nee<br>◯ Ja
12.5.1	***If 'Bent u in de afgelopen 3 maanden naar een logopedist geweest?' is equal to 'Ja' answer this question:***<br>Hoeveel afspraken had u?	[ ] afspraken
12.6	Bent u in de afgelopen 3 maanden naar een diëtist geweest?	◯ Nee<br>◯ Ja
12.6.1	***If 'Bent u in de afgelopen 3 maanden naar een diëtist geweest?' is equal to 'Ja' answer this question:***<br>Hoeveel afspraken had u?	[ ] afspraken

Paget LDA, *et al. BMJ Open* 2019; 9:e030961. doi: 10.1136/bmjopen-2019-030961

12.7	Bent u in de afgelopen 3 maanden naar een homeopaat geweest? Of een acupuncturist?	◯ Nee<br>◯ Ja
12.7.1	***If 'Bent u in de afgelopen 3 maanden naar een homeopaat geweest? Of een acupuncturist?' is equal to 'Ja' answer this question:***<br>Hoeveel afspraken had u?	[ ] afspraken
12.8	Bent u in de afgelopen 3 maanden naar een psycholoog geweest? Of een psychotherapeut of psychiater?	◯ Nee<br>◯ Ja
12.8.1	***If 'Bent u in de afgelopen 3 maanden naar een psycholoog geweest? Of een psychotherapeut of psychiater?' is equal to 'Ja' answer this question:***<br>Hoeveel afspraken had u?	[ ] afspraken
12.9	Heeft u in de afgelopen 3 maanden afspraken gehad met de bedrijfsarts?	◯ Nee<br>◯ Ja
12.9.1	***If 'Heeft u in de afgelopen 3 maanden afspraken gehad met de bedrijfsarts?' is equal to 'Ja' answer this question:***<br>Hoeveel afspraken had u?	[ ] afspraken
12.10	Heeft u in de afgelopen 3 maanden hulp van de thuiszorg gehad?	◯ Ja<br>◯ Nee
12.10.1	***If 'Heeft u in de afgelopen 3 maanden hulp van de thuiszorg gehad?' is equal to 'Ja' answer this question:***<br>Wat voor hulp van de thuiszorg heeft u gehad in de afgelopen 3 maanden?	☐ Huishoudelijke hulp; voorbeeld: stofzuigen, bed opmaken, boodschappen doen<br>☐ Verzorging van uzelf; voorbeeld: hulp bij douchen of aankleden<br>☐ Verpleging; voorbeeld: verband omdoen, medicijnen geven, bloeddruk meten
12.10.1.1	***If 'Wat voor hulp van de thuiszorg heeft u gehad in de afgelopen 3 maanden?' is equal to 'Huishoudelijke hulp; voorbeeld: stofzuigen, bed opmaken, boodschappen doen' answer this question:***<br>Hoeveel weken heeft u deze thuiszorg gehad? Huishoudelijke hulp:	[ ] weken in de afgelopen 3 maanden
12.10.1.2	***If 'Wat voor hulp van de thuiszorg heeft u gehad in de afgelopen 3 maanden?' is equal to 'Verzorging van uzelf; voorbeeld: hulp bij douchen of aankleden' answer this question:***<br>Hoeveel weken heeft u deze thuiszorg gehad? Verzorging van uzelf:	[ ] weken in de afgelopen 3 maanden

Paget LDA, *et al. BMJ Open* 2019; 9:e030961. doi: 10.1136/bmjopen-2019-030961

12.10.1.3	***If 'Wat voor hulp van de thuiszorg heeft u gehad in de afgelopen 3 maanden?' is equal to 'Verpleging; voorbeeld: verband omdoen, medicijnen geven, bloeddruk meten' answer this question:***<br>Hoeveel weken heeft u deze thuiszorg gehad? Verpleging:	______ weken in de afgelopen 3 maanden
12.10.1.4	***If 'Wat voor hulp van de thuiszorg heeft u gehad in de afgelopen 3 maanden?' is equal to 'Huishoudelijke hulp; voorbeeld: stofzuigen, bed opmaken, boodschappen doen' answer this question:***<br>Hoeveel uur thuiszorg kreeg u in deze weken gemiddeld? Huishoudelijke hulp: gemiddeld	______ uur in de week
12.10.1.5	***If 'Wat voor hulp van de thuiszorg heeft u gehad in de afgelopen 3 maanden?' is equal to 'Verzorging van uzelf; voorbeeld: hulp bij douchen of aankleden' answer this question:***<br>Hoeveel uur thuiszorg kreeg u in deze weken gemiddeld? Verzorging van uzelf: gemiddeld	______ uur in de week
12.10.1.6	***If 'Wat voor hulp van de thuiszorg heeft u gehad in de afgelopen 3 maanden?' is equal to 'Verpleging; voorbeeld: verband omdoen, medicijnen geven, bloeddruk meten' answer this question:***<br>Hoeveel uur thuiszorg kreeg u in deze weken gemiddeld? Verpleging: gemiddeld	______ uur in de week
12.11	Heeft u in de afgelopen 3 maanden medicijnen gebruikt?	○ Ja<br>○ Nee
12.11.1	***If 'Heeft u in de afgelopen 3 maanden medicijnen gebruikt?' is equal to 'Ja' answer this question:***<br>Welke medicijnen heeft u in de afgelopen 3 maanden gebruikt?	______
12.12	Bent u in de afgelopen 3 maanden op de spoedeisende eerste hulp van een ziekenhuis geweest? (Een andere naam voor spoedeisende eerste hulp is EHBO)	○ Nee<br>○ Ja
12.12.1	***If 'Bent u in de afgelopen 3 maanden op de spoedeisende eerste hulp van een ziekenhuis geweest? (Een andere naam voor spoedeisende eerste hulp is EHBO)' is equal to 'Ja' answer this question:***<br>Hoevaak bent u geweest?	______ keer
12.13	Bent u in de afgelopen 3 maanden met een ambulance naar het ziekenhuis gebracht? (Een andere naam voor ambulance is ziekenauto)	○ Nee<br>○ Ja

Paget LDA, *et al. BMJ Open* 2019; 9:e030961. doi: 10.1136/bmjopen-2019-030961

12.13.1	***If 'Bent u in de afgelopen 3 maanden met een ambulance naar het ziekenhuis gebracht? (Een andere naam voor ambulance is ziekenauto)' is equal to 'Ja' answer this question:***<br>Hoe vaak bent u naar het ziekenhuis gebracht?	[ ] keer
12.14	Had u in de afgelopen 3 maanden een afspraak bij de polikliniek van het ziekenhuis? (Het gaat om afspraken voor uzelf met een dokter. Bijvoorbeeld met de cardioloog, reumatoloog of neuroloog)	○ Ja<br>○ Nee
12.14.1	***If 'Had u in de afgelopen 3 maanden een afspraak bij de polikliniek van het ziekenhuis? (Het gaat om afspraken voor uzelf met een dokter. Bijvoorbeeld met de cardioloog, reumatoloog of neuroloog)' is equal to 'Ja' answer this question:***<br>Bij welke soorten dokters bent u in de afgelopen 3 maanden in het ziekenhuis geweest? En hoe vaak?	[ ]
12.15	Bent u in de afgelopen 3 maanden overdag in het ziekenhuis geweest voor een behandeling? (U bleef dus niet slapen. U kwam bijvoorbeeld voor een bloedtransfusie, nierdialyse of chemokuur)	○ Ja<br>○ Nee
12.15.1	***If 'Bent u in de afgelopen 3 maanden overdag in het ziekenhuis geweest voor een behandeling? (U bleef dus niet slapen. U kwam bijvoorbeeld voor een bloedtransfusie, nierdialyse of chemokuur)' is equal to 'Ja' answer this question:***<br>Voor welke soort behandeling was dit?	[ ]
12.15.2	***If 'Bent u in de afgelopen 3 maanden overdag in het ziekenhuis geweest voor een behandeling? (U bleef dus niet slapen. U kwam bijvoorbeeld voor een bloedtransfusie, nierdialyse of chemokuur)' is equal to 'Ja' answer this question:***<br>Hoeveel keer moest u in de afgelopen 3 maanden voor deze behandelingen naar het ziekenhuis?	[ ]
12.16	Bent u in de afgelopen 3 maanden ergens anders geweest voor een behandeling overdag? (U bleef dus niet slapen. U ging bijvoorbeeld naar de dagopvang van een woon-/zorgcentrum of een psychiatrische instelling. Of naar de dagbehandeling van een revalidatiecentrum)	○ Ja<br>○ Nee
12.16.1	***If 'Bent u in de afgelopen 3 maanden ergens anders geweest voor een behandeling overdag? (U bleef dus niet slapen. U ging bijvoorbeeld naar de dagopvang van een woon-/zorgcentrum of een psychiatrische instelling. Of naar de dagbehandeling van een revalidatiecentrum)' is equal to 'Ja' answer this question:***<br>Wat voor instelling was dit?	☐ Woon-/zorgcentrum<br>☐ Revalidatiecentrum<br>☐ Psychiatrische instelling<br>☐ Een andere instelling, namelijk

Paget LDA, *et al. BMJ Open* 2019; 9:e030961. doi: 10.1136/bmjopen-2019-030961

12.16.1.1	***If 'Wat voor instelling was dit?' is equal to 'Een andere instelling, namelijk' answer this question:***<br>Wat voor instelling was dit?	
12.16.1.2	***If 'Wat voor instelling was dit?' is equal to 'Woon-/zorgcentrum' answer this question:***<br>Hoe vaak moest u hier in de afgelopen 3 maanden naartoe? Naar het woon-/zorgcentrum:	keer in de afgelopen 3 maanden
12.16.1.3	***If 'Wat voor instelling was dit?' is equal to 'Revalidatiecentrum' answer this question:***<br>Hoe vaak moest u hier in de afgelopen 3 maanden naartoe? Naar het revalidatiecentrum:	keer in de afgelopen 3 maanden
12.16.1.4	***If 'Wat voor instelling was dit?' is equal to 'Psychiatrische instelling' answer this question:***<br>Hoe vaak moest u hier in de afgelopen 3 maanden naartoe? Naar de psychiatrische instelling:	keer in de afgelopen 3 maanden
12.16.1.5	***If 'Wat voor instelling was dit?' is equal to 'Een andere instelling, namelijk' answer this question:***<br>Hoe vaak moest u hier in de afgelopen 3 maanden naartoe? Naar de andere instelling:	keer in de afgelopen 3 maanden
12.17	Heeft u in de afgelopen 3 maanden weleens in het ziekenhuis gelegen? (U moest dus blijven slapen. Bijvoorbeeld omdat u geopereerd was en niet meteen naar huis kon)	◯ Ja<br>◯ Nee
12.17.1	***If 'Heeft u in de afgelopen 3 maanden weleens in het ziekenhuis gelegen? (U moest dus blijven slapen. Bijvoorbeeld omdat u geopereerd was en niet meteen naar huis kon)' is equal to 'Ja' answer this question:***<br>Hoe vaak heeft u in de afgelopen 3 maanden in het ziekenhuis gelegen?	keer in de afgelopen 3 maanden
12.17.2	***If 'Heeft u in de afgelopen 3 maanden weleens in het ziekenhuis gelegen? (U moest dus blijven slapen. Bijvoorbeeld omdat u geopereerd was en niet meteen naar huis kon)' is equal to 'Ja' answer this question:***<br>Hoe lang heeft u in het ziekenhuis gelegen?	dagen in de afgelopen 3 maanden
12.18	Moest u in de afgelopen 3 maanden ergens anders blijven slapen voor uw gezondheid? (Bijvoorbeeld in een woon-/zorgcentrum, psychiatrische instelling of revalidatiecentrum)	◯ Ja<br>◯ Nee

Paget LDA, *et al*. *BMJ Open* 2019; 9:e030961. doi: 10.1136/bmjopen-2019-030961

12.18.1	***If 'Moest u in de afgelopen 3 maanden ergens anders blijven slapen voor uw gezondheid? (Bijvoorbeeld in een woon-/zorgcentrum, psychiatrische instelling of revalidatiecentrum)' is equal to 'Ja' answer this question:***<br>Wat voor instelling was dit?	☐ Woon-/zorgcentrum<br>☐ Revalidatiecentrum<br>☐ Psychiatrische instelling<br>☐ Een andere instelling, namelijk
12.18.1.1	***If 'Wat voor instelling was dit?' is equal to 'Een andere instelling, namelijk' answer this question:***<br>Een andere instelling, namelijk	
12.18.1.2	***If 'Wat voor instelling was dit?' is equal to 'Woon-/zorgcentrum' answer this question:***<br>Hoe lang bent u in deze instelling geweest? In het woon-/zorgcentrum:	____ dagen in de afgelopen 3 maanden
12.18.1.3	***If 'Wat voor instelling was dit?' is equal to 'Revalidatiecentrum' answer this question:***<br>Hoe lang bent u in deze instelling geweest? In het revalidatiecentrum:	____ dagen in de afgelopen 3 maanden
12.18.1.4	***If 'Wat voor instelling was dit?' is equal to 'Psychiatrische instelling' answer this question:***<br>Hoe lang bent u in deze instelling geweest? In de psychiatrische instelling:	____ dagen in de afgelopen 3 maanden
12.18.1.5	***If 'Wat voor instelling was dit?' is equal to 'Een andere instelling, namelijk' answer this question:***<br>Hoe lang bent u in deze instelling geweest? In de andere instelling:	____ dagen in de afgelopen 3 maanden
12.19	Heeft u in de afgelopen 3 maanden hulp gekregen van een familielid of een bekende vanwege uw lichamelijke of psychische problemen?	○ Ja<br>○ Nee
12.19.1	***If 'Heeft u in de afgelopen 3 maanden hulp gekregen van een familielid of een bekende vanwege uw lichamelijke of psychische problemen?' is equal to 'Ja' answer this question:***<br>Wat voor hulp van familieleden of bekenden heeft u gehad in de afgelopen 3 maanden?	☐ Huishoudelijke hulp - voorbeeld: stofzuigen, bed opmaken, boodschappen doen, klaarmaken van eten en drinken, verzorgen van kinderen<br>☐ Verzorging van uzelf - voorbeeld: hulp bij douchen of aankleden, hulp bij het eten en drinken of het geven van medicijnen<br>☐ Praktische hulp - voorbeeld: ondersteuning bij wandelen, het maken van uitstapjes of bezoekjes aan bekenden, bezoeken aan de huisarts of het ziekenhuis, het regelen van hulp of het regelen van financiële zaken

Paget LDA, *et al*. *BMJ Open* 2019; 9:e030961. doi: 10.1136/bmjopen-2019-030961

12.19.1.1	***If 'Wat voor hulp van familieleden of bekenden heeft u gehad in de afgelopen 3 maanden?' is equal to 'Huishoudelijke hulp - voorbeeld: stofzuigen, bed opmaken, boodschappen doen, klaarmaken van eten en drinken, verzorgen van kinderen' answer this question:*** Hoeveel weken heeft u deze hulp gehad? Huishoudelijke hulp:	[ ] weken in de afgelopen 3 maanden
12.19.1.2	***If 'Wat voor hulp van familieleden of bekenden heeft u gehad in de afgelopen 3 maanden?' is equal to 'Verzorging van uzelf - voorbeeld: hulp bij douchen of aankleden, hulp bij het eten en drinken of het geven van medicijnen' answer this question:*** Hoeveel weken heeft u deze hulp gehad? Verzorging van uzelf:	[ ] weken in de afgelopen 3 maanden
12.19.1.3	***If 'Wat voor hulp van familieleden of bekenden heeft u gehad in de afgelopen 3 maanden?' is equal to 'Praktische hulp - voorbeeld: ondersteuning bij wandelen, het maken van uitstapjes of bezoekjes aan bekenden, bezoeken aan de huisarts of het ziekenhuis, het regelen van hulp of het regelen van financiële zaken' answer this question:*** Hoeveel uur hulp kreeg u in deze weken gemiddeld? Praktische hulp:	[ ] weken in de afgelopen 3 maanden
12.19.1.4	***If 'Wat voor hulp van familieleden of bekenden heeft u gehad in de afgelopen 3 maanden?' is equal to 'Huishoudelijke hulp - voorbeeld: stofzuigen, bed opmaken, boodschappen doen, klaarmaken van eten en drinken, verzorgen van kinderen' answer this question:*** Hoeveel uur hulp kreeg u in deze weken gemiddeld? Huishoudelijke hulp: gemiddeld	[ ] uur in de week
12.19.1.5	***If 'Wat voor hulp van familieleden of bekenden heeft u gehad in de afgelopen 3 maanden?' is equal to 'Verzorging van uzelf - voorbeeld: hulp bij douchen of aankleden, hulp bij het eten en drinken of het geven van medicijnen' answer this question:*** Hoeveel uur hulp kreeg u in deze weken gemiddeld? Verzorging van uzelf: gemiddeld	[ ] uur in de week
12.19.1.6	***If 'Wat voor hulp van familieleden of bekenden heeft u gehad in de afgelopen 3 maanden?' is equal to 'Praktische hulp - voorbeeld: ondersteuning bij wandelen, het maken van uitstapjes of bezoekjes aan bekenden, bezoeken aan de huisarts of het ziekenhuis, het regelen van hulp of het regelen van financiële zaken' answer this question:*** Hoeveel uur hulp kreeg u in deze weken gemiddeld? Praktische hulp: gemiddeld	[ ] uur in de week

Paget LDA, *et al*. *BMJ Open* 2019; 9:e030961. doi: 10.1136/bmjopen-2019-030961

Vragenlijst PRIMA studie 12 weken - Dank

Number	Question	Answers
13.1	Hartelijk dank voor de tijd die u heeft genomen om de vragenlijsten in te vullen. Indien u nog opmerkingen heeft kunt u deze hierin plaatsen.	

Survey 'Vragenlijst PRIMA studie 26 weken'

Vragenlijst PRIMA studie 26 weken - AOFAS - Pijn

Number	Question	Answers
1.1	Hoeveel pijn ervaart u aan uw voet?	☐ Geen<br>☐ Milde pijnklachten of af en toe pijn<br>☐ Matige pijnklachten, dagelijks<br>☐ Vrijwel continu erge pijn

Vragenlijst PRIMA studie 26 weken - AOFAS resterende gedeelte

Number	Question	Answers
2.1	Hoeveel beperkingen in het dagelijks leven ervaart u?	○ Geen beperkingen, geen hulpmiddelen nodig<br>○ Geen beperkingen in algemeen dagelijkse bezigheden, wel beperkingen in recreatieve activiteiten (sport, hobbies, etc) geen ondersteuning<br>○ Beperkingen bij dagelijkse- en recreatieve activiteiten, stok<br>○ Ernstige beperkingen bij dagelijkse- en recreatieactiviteiten, walker, krukken, rolstoel, brace

Paget LDA, *et al. BMJ Open* 2019; 9:e030961. doi: 10.1136/bmjopen-2019-030961

2.2	Wat is de afstand die u in één keer kunt lopen?	◯ Meer dan 600 meter<br>◯ 400 - 600 meter<br>◯ 100 - 300 meter<br>◯ Minder dan 100 meter
2.3	Heeft u moeite met lopen op een bepaalde ondergrond?	◯ Geen problemen bij lopen<br>◯ Enig problemen op ongelijke ondergrond, trap, helling, ladders<br>◯ Ernstige problemen bij ongelijke ondergrond, trap, helling, ladders
2.4	Hoe zou u uw looppatroon beoordelen?	◯ Normaal of minimaal afwijkend<br>◯ Duidelijk<br>◯ Ernstig
2.5	Hoe zou u de beweeglijkheid van uw voet beoordelen ten opzicht van de andere zijde of ten opzicte van vóór het ongeval?	◯ Hetzelfde of licht beperkt t.o.v. de niet aangedane zijde of zoals voor het ongeval<br>◯ Ongeveer de helft beperkt<br>◯ Sterk verminderd, bijna geen bewegingen mogelijk
2.6	Hoe zou u de beweeglijkheid van uw enkel beoordelen ten opzichte van de andere zijde of ten opzichte van voor het ongeval?	◯ Hetzelfde of licht beperkt t.o.v. de niet aangedane zijde of zoals voor het ongeval<br>◯ Ongeveer de helft beperkt<br>◯ Sterk verminderd, bijna geen bewegingen mogelijk
2.7	Hoe zou u de stabiliteit van uw voet en enkel beoordelen	◯ Stabiel, ik verzwik mijn enkel hooguit incidenteel<br>◯ Niet stabiel, ik verzwik mijn enkel vaak
2.8	Hoe zou u de stand van uw voet beoordelen ten opzichte van de andere zijde of ten opzichte van voor het ongeval?	◯ Goed, ik kan mijn voet goed plat op de grond zetten, waarbij de stand niet afwijkend is<br>◯ Ik sta vrijwel alleen op de buitenzijde van mijn voet<br>◯ Ik kan mijn voet niet plat op de grond zetten en loop vrijwel alleen op mijn voorvoet

Vragenlijst PRIMA studie 26 weken - VAS Pain score

Paget LDA, *et al. BMJ Open* 2019; 9:e030961. doi: 10.1136/bmjopen-2019-030961

Number	Question	Answers	
3.1	VAS pijnscore (Geef uw minimaal pijnscore aan tijdens dagelijkse activiteiten op een schaal van 0 tot 100, waarbij 0 geen pijn is en 100 de ergste pijn denkbaar is)	(0.00)	100 (100.00)

Vragenlijst PRIMA studie 26 weken - AAS

Number	Question	Answers

Paget LDA, *et al. BMJ Open* 2019; 9:e030961. doi: 10.1136/bmjopen-2019-030961

4.1	Wat is het meest bij u van toepassing ten aanzien van uw acitviteit?	☐ American Football<br>☐ Basketbal<br>☐ Gymnastiek/turnen<br>☐ Handbal<br>☐ Rugby<br>☐ Voetbal<br>☐ Hockey<br>☐ Korfbal<br>☐ Gevechtssporten: judo, karate, kung fu, taekwando, jiu jitsu, etc.<br>☐ Oriëntatielopen<br>☐ Rhytmische gymnastiek<br>☐ Volleybal<br>☐ Boxen<br>☐ Freestyle snowboarden<br>☐ Ijshockey<br>☐ Tennis<br>☐ Worstelen<br>☐ Fitness, aerobics<br>☐ Badminton<br>☐ Baseball<br>☐ Cross-country hardlopen<br>☐ Moderne pentathlon<br>☐ Squash<br>☐ Surfen, windsurfen<br>☐ Tafel tennis<br>☐ Atletiek: spring-, werponderdelen<br>☐ Waterskieën<br>☐ Dans<br>☐ Schermen<br>☐ Zaalhockey<br>☐ Bergbeklimmen<br>☐ Langlauf<br>☐ Parachute springen<br>☐ Softball<br>☐ Speciale beroepen en werkactiviteiten; speciale beroepen: ballet, professioneel soldaat, speciale reddingswerker, stuntman, etc.<br>☐ Duiken<br>☐ Scubaduiken<br>☐ Skaten, in-line skaten<br>☐ Atletiek: looponderdelen<br>☐ Triatlon<br>☐ Gewichtsheffen, body-building

Paget LDA, *et al. BMJ Open* 2019; 9:e030961. doi: 10.1136/bmjopen-2019-030961

		☐ Alle competitieve sporten hieronder met 'seasonal' conditioning<br>☐ Zwaar fysiek werk<br>☐ Alpine skiën en snowboarden<br>☐ Bowlen/curlen<br>☐ Golf<br>☐ Mountainbike/BMX<br>☐ Powerliften<br>☐ Zeilen<br>☐ Fysiek werk<br>☐ Wielrennen<br>☐ Paardrijden<br>☐ Motorsporten/technische sporten<br>☐ Roeien, kayakken<br>☐ Boogschieten<br>☐ Water polo en zwemmen<br>☐ Kunnen lopen op oneven grond<br>☐ Geen sport, echter geen limitatie in dagelijkse activiteiten<br>☐ Kunnen lopen op even grond, maar de dagelijkste activiteiten zijn gelimiteerd<br>☐ Niet kunnen lopen, fysieke handicap wegens enkelproblemen
4.1.1	***If 'Wat is het meest bij u van toepassing ten aanzien van uw acitviteit?' is equal to 'American Football' answer this question:***<br>American Football	○ Professioneel<br>○ Competitief<br>○ Recreatief
4.1.2	***If 'Wat is het meest bij u van toepassing ten aanzien van uw acitviteit?' is equal to 'Basketbal' answer this question:***<br>Basketbal	○ Professioneel<br>○ Competitief<br>○ Recreatief
4.1.3	***If 'Wat is het meest bij u van toepassing ten aanzien van uw acitviteit?' is equal to 'Gymnastiek/turnen' answer this question:***<br>Gymnastiek/turnen	○ Professioneel<br>○ Competitief<br>○ Recreatief
4.1.4	***If 'Wat is het meest bij u van toepassing ten aanzien van uw acitviteit?' is equal to 'Handbal' answer this question:***<br>Handbal	○ Professioneel<br>○ Competitief<br>○ Recreatief
4.1.5	***If 'Wat is het meest bij u van toepassing ten aanzien van uw acitviteit?' is equal to 'Rugby' answer this question:***<br>Rugby	○ Professioneel<br>○ Competitief<br>○ Recreatief

Paget LDA, *et al*. *BMJ Open* 2019; 9:e030961. doi: 10.1136/bmjopen-2019-030961

4.1.6	***If 'Wat is het meest bij u van toepassing ten aanzien van uw acitviteit?' is equal to 'Voetbal' answer this question:*** Voetbal	◯ Professioneel ◯ Competitief ◯ Recreatief
4.1.7	***If 'Wat is het meest bij u van toepassing ten aanzien van uw acitviteit?' is equal to 'Hockey' answer this question:*** Hockey	◯ Professioneel ◯ Competitief ◯ Recreatief
4.1.8	***If 'Wat is het meest bij u van toepassing ten aanzien van uw acitviteit?' is equal to 'Korfbal' answer this question:*** Korfbal	◯ Professioneel ◯ Competitief ◯ Recreatief
4.1.9	***If 'Wat is het meest bij u van toepassing ten aanzien van uw acitviteit?' is equal to 'Gevechtssporten: judo, karate, kung fu, taekwando, jiu jitsu, etc.' answer this question:*** Gevechtssporten: judo, karate, kung fu, taekwando, jiu jitsu, etc.	◯ Professioneel ◯ Competitief ◯ Recreatief
4.1.10	***If 'Wat is het meest bij u van toepassing ten aanzien van uw acitviteit?' is equal to 'Oriëntatielopen' answer this question:*** Oriëntatielopen	◯ Professioneel ◯ Competitief ◯ Recreatief
4.1.11	***If 'Wat is het meest bij u van toepassing ten aanzien van uw acitviteit?' is equal to 'Rhytmische gymnastiek' answer this question:*** Rhytmisch gymnastiek	◯ Professioneel ◯ Competitief ◯ Recreatief
4.1.12	***If 'Wat is het meest bij u van toepassing ten aanzien van uw acitviteit?' is equal to 'Volleybal' answer this question:*** Volleybal	◯ Professioneel ◯ Competitief ◯ Recreatief
4.1.13	***If 'Wat is het meest bij u van toepassing ten aanzien van uw acitviteit?' is equal to 'Boxen' answer this question:*** Boxen	◯ Professioneel ◯ Competitief ◯ Recreatief
4.1.14	***If 'Wat is het meest bij u van toepassing ten aanzien van uw acitviteit?' is equal to 'Freestyle snowboarden' answer this question:*** Freestyle snowboarden	◯ Professioneel ◯ Competitief ◯ Recreatief
4.1.15	***If 'Wat is het meest bij u van toepassing ten aanzien van uw acitviteit?' is equal to 'Freestyle snowboarden' answer this question:*** IJshockey	◯ Professioneel ◯ Competitief ◯ Recreatief

Paget LDA, *et al. BMJ Open* 2019; 9:e030961. doi: 10.1136/bmjopen-2019-030961

4.1.16	***If 'Wat is het meest bij u van toepassing ten aanzien van uw acitviteit?' is equal to 'Tennis' answer this question:***<br>Tennis	◯ Professioneel<br>◯ Competitief<br>◯ Recreatief
4.1.17	***If 'Wat is het meest bij u van toepassing ten aanzien van uw acitviteit?' is equal to 'Worstelen' answer this question:***<br>Worstelen	◯ Professioneel<br>◯ Competitief<br>◯ Recreatief
4.1.18	***If 'Wat is het meest bij u van toepassing ten aanzien van uw acitviteit?' is equal to 'Fitness, aerobics' answer this question:***<br>Fitness, aerobics	◯ Professioneel<br>◯ Competitief<br>◯ Recreatief
4.1.19	***If 'Wat is het meest bij u van toepassing ten aanzien van uw acitviteit?' is equal to 'Badminton' answer this question:***<br>Badminton	◯ Professioneel<br>◯ Competitief<br>◯ Recreatief
4.1.20	***If 'Wat is het meest bij u van toepassing ten aanzien van uw acitviteit?' is equal to 'Baseball' answer this question:***<br>Baseball	◯ Professioneel<br>◯ Competitief<br>◯ Recreatief
4.1.21	***If 'Wat is het meest bij u van toepassing ten aanzien van uw acitviteit?' is equal to 'Cross-country hardlopen' answer this question:***<br>Cross-country hardlopen	◯ Professioneel<br>◯ Competitief<br>◯ Recreatief
4.1.22	***If 'Wat is het meest bij u van toepassing ten aanzien van uw acitviteit?' is equal to 'Moderne pentathlon' answer this question:***<br>Moderne pentathlon	◯ Professioneel<br>◯ Competitief<br>◯ Recreatief
4.1.23	***If 'Wat is het meest bij u van toepassing ten aanzien van uw acitviteit?' is equal to 'Squash' answer this question:***<br>Squash	◯ Professioneel<br>◯ Competitief<br>◯ Recreatief
4.1.24	***If 'Wat is het meest bij u van toepassing ten aanzien van uw acitviteit?' is equal to 'Surfen, windsurfen' answer this question:***<br>Surfen, windsurfen	◯ Professioneel<br>◯ Competitief<br>◯ Recreatief
4.1.25	***If 'Wat is het meest bij u van toepassing ten aanzien van uw acitviteit?' is equal to 'Tafel tennis' answer this question:***<br>Tafel tennis	◯ Professioneel<br>◯ Competitief<br>◯ Recreatief

Paget LDA, *et al. BMJ Open* 2019; 9:e030961. doi: 10.1136/bmjopen-2019-030961

4.1.26	***If 'Wat is het meest bij u van toepassing ten aanzien van uw acitviteit?' is equal to 'Atletiek: spring-, werponderdelen' answer this question:***<br>Atletiek: spring-, werponderdelen	◯ Professioneel<br>◯ Competitief<br>◯ Recreatief
4.1.27	***If 'Wat is het meest bij u van toepassing ten aanzien van uw acitviteit?' is equal to 'Waterskieën' answer this question:***<br>Waterskien	◯ Professioneel<br>◯ Competitief<br>◯ Recreatief
4.1.28	***If 'Wat is het meest bij u van toepassing ten aanzien van uw acitviteit?' is equal to 'Dans' answer this question:***<br>Dans	◯ Professioneel<br>◯ Competitief<br>◯ Recreatief
4.1.29	***If 'Wat is het meest bij u van toepassing ten aanzien van uw acitviteit?' is equal to 'Schermen' answer this question:***<br>Schermen	◯ Professioneel<br>◯ Competitief<br>◯ Recreatief
4.1.30	***If 'Wat is het meest bij u van toepassing ten aanzien van uw acitviteit?' is equal to 'Zaalhockey' answer this question:***<br>Zaalhockey	◯ Professioneel<br>◯ Competitief<br>◯ Recreatief
4.1.31	***If 'Wat is het meest bij u van toepassing ten aanzien van uw acitviteit?' is equal to 'Bergbeklimmen' answer this question:***<br>Bergbeklimmen	◯ Professioneel<br>◯ Competitief<br>◯ Recreatief
4.1.32	***If 'Wat is het meest bij u van toepassing ten aanzien van uw acitviteit?' is equal to 'Langlauf' answer this question:***<br>Langlauf	◯ Professioneel<br>◯ Competitief<br>◯ Recreatief
4.1.33	***If 'Wat is het meest bij u van toepassing ten aanzien van uw acitviteit?' is equal to 'Parachute springen' answer this question:***<br>Parachute springen	◯ Professioneel<br>◯ Competitief<br>◯ Recreatief
4.1.34	***If 'Wat is het meest bij u van toepassing ten aanzien van uw acitviteit?' is equal to 'Softball' answer this question:***<br>Softball	◯ Professioneel<br>◯ Competitief<br>◯ Recreatief

Paget LDA, *et al. BMJ Open* 2019; 9:e030961. doi: 10.1136/bmjopen-2019-030961

4.1.35	***If 'Wat is het meest bij u van toepassing ten aanzien van uw acitviteit?' is equal to 'Speciale beroepen en werkactiviteiten; speciale beroepen: ballet, professioneel soldaat, speciale reddingswerker, stuntman, etc.' answer this question:***<br>Speciale beroepen en werkactiviteiten speciale beroepen: ballet, professioneel soldaat, speciale reddingswerker, stuntman, etc.	◯ ballet, professioneel soldaat, speciale reddingswerker, stuntman, etc.
4.1.36	***If 'Wat is het meest bij u van toepassing ten aanzien van uw acitviteit?' is equal to 'Duiken' answer this question:***<br>Duiken	◯ Professioneel/competitief<br>◯ Recreatief
4.1.37	***If 'Wat is het meest bij u van toepassing ten aanzien van uw acitviteit?' is equal to 'Scubaduiken' answer this question:***<br>Scubaduiken	◯ Professioneel/competitief<br>◯ Recreatief
4.1.38	***If 'Wat is het meest bij u van toepassing ten aanzien van uw acitviteit?' is equal to 'Skaten, in-line skaten' answer this question:***<br>skaten, in-linen skaten	◯ Professioneel/competitief<br>◯ Recreatief
4.1.39	***If 'Wat is het meest bij u van toepassing ten aanzien van uw acitviteit?' is equal to 'Atletiek: looponderdelen' answer this question:***<br>Atletiek: looponderdelen	◯ Professioneel/competitief<br>◯ Recreatief
4.1.40	***If 'Wat is het meest bij u van toepassing ten aanzien van uw acitviteit?' is equal to 'Triatlon' answer this question:***<br>triatlon	◯ Professioneel/competitief<br>◯ Recreatief
4.1.41	***If 'Wat is het meest bij u van toepassing ten aanzien van uw acitviteit?' is equal to 'Gewichtsheffen, body-building' answer this question:***<br>Gewichtsheffen, body-building	◯ Professioneel/competitief<br>◯ Recreatief
4.1.42	***If 'Wat is het meest bij u van toepassing ten aanzien van uw acitviteit?' is equal to 'Alle competitieve sporten hieronder met ‘seasonal’ conditioning' answer this question:***<br>Alle competitieve sporten hieronder met ‘seasonal’ conditioning	◯ Alle competitieve sporten hieronder met ‘seasonal’ conditioning
4.1.43	***If 'Wat is het meest bij u van toepassing ten aanzien van uw acitviteit?' is equal to 'Zwaar fysiek werk' answer this question:***<br>Zwaar fysiek werk	◯ Alle competitieve sporten hieronder met ‘seasonal’ conditioning

Paget LDA, *et al. BMJ Open* 2019; 9:e030961. doi: 10.1136/bmjopen-2019-030961

4.1.44	***If 'Wat is het meest bij u van toepassing ten aanzien van uw acitviteit?' is equal to 'Alpine skiën en snowboarden' answer this question:*** Alpine skien en snowboarden	◯ Alpine skien en snowboarden
4.1.45	***If 'Wat is het meest bij u van toepassing ten aanzien van uw acitviteit?' is equal to 'Bowlen/curlen' answer this question:*** Bowlen/curlen	◯ Bowlen/curlen
4.1.46	***If 'Wat is het meest bij u van toepassing ten aanzien van uw acitviteit?' is equal to 'Golf' answer this question:*** Golf	◯ Golf
4.1.47	***If 'Wat is het meest bij u van toepassing ten aanzien van uw acitviteit?' is equal to 'Mountainbike/BMX' answer this question:*** Mountainbike/BMX	◯ Mountainbike/BMX
4.1.48	***If 'Wat is het meest bij u van toepassing ten aanzien van uw acitviteit?' is equal to 'Powerliften' answer this question:*** Powerliften	◯ Powerliften
4.1.49	***If 'Wat is het meest bij u van toepassing ten aanzien van uw acitviteit?' is equal to 'Zeilen' answer this question:*** Zeilen	◯ Zeilen
4.1.50	***If 'Wat is het meest bij u van toepassing ten aanzien van uw acitviteit?' is equal to 'Fysiek werk' answer this question:*** Fysiek werk	◯ Fysiek werk
4.1.51	***If 'Wat is het meest bij u van toepassing ten aanzien van uw acitviteit?' is equal to 'Wielrennen' answer this question:*** Wielrennen	◯ Wielrennen
4.1.52	***If 'Wat is het meest bij u van toepassing ten aanzien van uw acitviteit?' is equal to 'Paardrijden' answer this question:*** Paardrijden	◯ Paardrijden
4.1.53	***If 'Wat is het meest bij u van toepassing ten aanzien van uw acitviteit?' is equal to 'Motorsporten/technische sporten' answer this question:*** Motorsporten/technische sporten	◯ Motorsporten/technische sporten
4.1.54	***If 'Wat is het meest bij u van toepassing ten aanzien van uw acitviteit?' is equal to 'Roeien, kayakken' answer this question:*** Roeien, kayakken	◯ Roeien, kayakken

Paget LDA, *et al. BMJ Open* 2019; 9:e030961. doi: 10.1136/bmjopen-2019-030961

4.1.55	***If 'Wat is het meest bij u van toepassing ten aanzien van uw acitviteit?' is equal to 'Boogschieten' answer this question:*** Boogschieten	◯ Boogschieten
4.1.56	***If 'Wat is het meest bij u van toepassing ten aanzien van uw acitviteit?' is equal to 'Water polo en zwemmen' answer this question:*** Water polo en zwemmen	◯ Water polo en zwemmen
4.1.57	***If 'Wat is het meest bij u van toepassing ten aanzien van uw acitviteit?' is equal to 'Kunnen lopen op oneven grond' answer this question:*** Kunnen lopen op oneven grond	◯ Kunnen lopen op oneven grond
4.1.58	***If 'Wat is het meest bij u van toepassing ten aanzien van uw acitviteit?' is equal to 'Geen sport, echter geen limitatie in dagelijkse activiteiten' answer this question:*** Geen sport, echter geen limitatie in dagelijkse activiteiten	◯ Geen sport, echter geen limitatie in dagelijkse activiteiten
4.1.59	***If 'Wat is het meest bij u van toepassing ten aanzien van uw acitviteit?' is equal to 'Kunnen lopen op even grond, maar de dagelijkste activiteiten zijn gelimiteerd' answer this question:*** Kunnen lopen op even grond, maar de dagelijkste activiteiten zijn gelimiteerd	◯ Kunnen lopen op even grond, maar de dagelijkste activiteiten zijn gelimiteerd
4.1.60	***If 'Wat is het meest bij u van toepassing ten aanzien van uw acitviteit?' is equal to 'Niet kunnen lopen, fysieke handicap wegens enkelproblemen' answer this question:*** Niet kunnen lopen, fysieke handicap wegens enkelproblemen	◯ Niet kunnen lopen, fysieke handicap wegens enkelproblemen

Vragenlijst PRIMA studie 26 weken - Hoe tevreden bent u over de enkelklachten?

Number	Question	Answers
5.1	Hoe tevreden bent u over de enkelklachten?	◯ Slecht<br>◯ Matig<br>◯ Goed<br>◯ Uitstekend

Paget LDA, *et al. BMJ Open* 2019; 9:e030961. doi: 10.1136/bmjopen-2019-030961

Vragenlijst PRIMA studie 26 weken - SF-36

Number	Question	Answers
	Deze vragenlijst gaat over uw standpunten t.a.v. uw gezondheid. Met behulp van deze gegevens kan worden bijgehouden hoe u zich voelt en hoe goed u in staat bent uw gebruikelijke bezigheden uit te voeren. Beantwoord elke vraag door een antwoord aan te klikken. Als u niet zeker weet hoe u een vraag moet beantwoorden, geef dan het best mogelijke antwoord.	
6.1	Hoe zou u over het algemeen uw gezondheid noemen?	◯ Uitstekend ◯ Zeer goed ◯ Goed ◯ Matig ◯ Slecht
6.2	Hoe beoordeelt u nu uw gezondheid over het algemeen vergeleken met een jaar geleden?	◯ Veel beter dan een jaar geleden ◯ Wat beter dan een jaar geleden ◯ Ongeveer hetzelfde als een jaar geleden ◯ Wat slechter dan een jaar geleden ◯ Veel slechter dan een jaar geleden
6.3	Wordt u door uw gezondheid op dit moment beperkt bij forse inspanning, zoals hardlopen, tillen van zware voorwerpen of een veeleisende sport beoefenen?	◯ Ja, ernstig beperkt ◯ Ja, een beetje beperkt ◯ Nee, helemaal niet beperkt
6.4	Wordt u door uw gezondheid op dit moment beperkt bij matige inspanning zoals een tafel verplaatsen, stofzuigen, zwemmen of fietsen?	◯ Ja, ernstig beperkt ◯ Ja, een beetje beperkt ◯ Nee, helemaal niet beperkt
6.5	Wordt u door uw gezondheid op dit moment beperkt bij boodschappen tillen of dragen?	◯ Ja, ernstig beperkt ◯ Ja, een beetje beperkt ◯ Nee, helemaal niet beperkt
6.6	Wordt u door uw gezondheid op dit moment beperkt bij een paar trappen oplopen?	◯ Ja, ernstig beperkt ◯ Ja, een beetje beperkt ◯ Nee, helemaal niet beperkt
6.7	Wordt u door uw gezondheid op dit moment beperkt bij één trap oplopen?	◯ Ja, ernstig beperkt ◯ Ja, een beetje beperkt ◯ Nee, helemaal niet beperkt
6.8	Wordt u door uw gezondheid op dit moment beperkt bij bukken, knielen of hurken?	◯ Ja, ernstig beperkt ◯ Ja, een beetje beperkt ◯ Nee, helemaal niet beperkt
6.9	Wordt u door uw gezondheid op dit moment beperkt bij meer dan één kilometer lopen?	◯ Ja, ernstig beperkt ◯ Ja, een beetje beperkt ◯ Nee, helemaal niet beperkt
6.10	Wordt u door uw gezondheid op dit moment beperkt bij een paar honderd meter lopen?	◯ Ja, ernstig beperkt ◯ Ja, een beetje beperkt ◯ Nee, helemaal niet beperkt

Paget LDA, *et al. BMJ Open* 2019; 9:e030961. doi: 10.1136/bmjopen-2019-030961

6.11	Wordt u door uw gezondheid op dit moment beperkt bij ongeveer honderd meter lopen?	◯ Ja, ernstig beperkt ◯ Ja, een beetje beperkt ◯ Nee, helemaal niet beperkt
6.12	Wordt u door uw gezondheid op dit moment beperkt bij uzelf wassen of aankleden?	◯ Ja, ernstig beperkt ◯ Ja, een beetje beperkt ◯ Nee, helemaal niet beperkt
6.13	U besteedde in de afgelopen 4 weken minder tijd aan werk of andere bezigheden	◯ Ja ◯ Nee
6.14	U heeft in de afgelopen 4 weken minder bereikt dan u zou willen	◯ Ja ◯ Nee
6.15	U was in de afgelopen 4 weken beperkt in het soort werk of andere bezigheden.	◯ Ja ◯ Nee
6.16	U had de afgelopen 4 weken moeite om uw werk of andere bezigheden uit te voeren (het kostte u bijvoorbeeld extra inspanning).	◯ Ja ◯ Nee
6.17	U besteedde in de afgelopen 4 weken minder tijd aan werk of andere bezigheden ten gevolge van emotionele problemen	◯ Ja ◯ Nee
6.18	U heeft in de afgelopen 4 weken minder bereikt dan u zou willen ten gevolge van emotionele problemen.	◯ Ja ◯ Nee
6.19	U deed de afgelopen 4 weken uw werk of andere bezigheden niet zo zorgvuldig als gewoonlijk ten gevolge van emotionele problemen.	◯ Ja ◯ Nee
6.20	In hoeverre hebben uw lichamelijke gezondheid of emotionele problemen u gedurende de afgelopen 4 weken gehinderd in uw normale omgang met familie, vrienden of buren, of bij activiteiten in groepsverband?	◯ Helemaal niet ◯ Enigszins ◯ Nogal ◯ Veel ◯ Heel erg veel
6.21	Hoeveel lichamelijke pijn heeft u de afgelopen 4 weken gehad?	◯ Geen<br>◯ Heel licht<br>◯ Licht<br>◯ Nogal<br>◯ Ernstig<br>◯ Heel ernstig
6.22	In welke mate bent u de afgelopen 4 weken door de pijn gehinderd in uw normale werk (zowel werk buitenshuis als huishoudelijk werk)?	◯ Helemaal niet<br>◯ Een klein beetje<br>◯ Nogal<br>◯ Veel<br>◯ Heel erg veel

6.23	Voelde u zich levenslustig?	◯ Altijd<br>◯ Meestal<br>◯ Vaak<br>◯ Soms<br>◯ Zelden<br>◯ Nooit
6.24	Was u erg zenuwachtig?	◯ Altijd<br>◯ Meestal<br>◯ Vaak<br>◯ Soms<br>◯ Zelden<br>◯ Nooit
6.25	Zat u zo in de put dat u niets kon?	◯ Altijd<br>◯ Meestal<br>◯ Vaak<br>◯ Soms<br>◯ Zelden<br>◯ Nooit
6.26	Voelde u zich rustig en tevreden?	◯ Altijd<br>◯ Meestal<br>◯ Vaak<br>◯ Soms<br>◯ Zelden<br>◯ Nooit
6.27	Had u veel energie?	◯ Altijd<br>◯ Meestal<br>◯ Vaak<br>◯ Soms<br>◯ Zelden<br>◯ Nooit
6.28	Voelde u zich somber en neerslachtig?	◯ Altijd<br>◯ Meestal<br>◯ Vaak<br>◯ Soms<br>◯ Zelden<br>◯ Nooit

6.29	Voelde u zich uitgeput?	◯ Altijd<br>◯ Meestal<br>◯ Vaak<br>◯ Soms<br>◯ Zelden<br>◯ Nooit
6.30	Was u een gelukkig mens?	◯ Altijd<br>◯ Meestal<br>◯ Vaak<br>◯ Soms<br>◯ Zelden<br>◯ Nooit
6.31	Voelde u zich moe?	◯ Altijd<br>◯ Meestal<br>◯ Vaak<br>◯ Soms<br>◯ Zelden<br>◯ Nooit
6.32	Hoe vaak hebben uw lichamelijke gezondheid of emotionele problemen u gedurende de afgelopen 4 weken gehinderd bij uw sociale activiteiten (zoals vrienden of familie bezoeken etc.)?	◯ Altijd<br>◯ Meestal<br>◯ Soms<br>◯ Zelden<br>◯ Nooit
6.33	Ik lijk wat gemakkelijker ziek te worden dan andere mensen	◯ Volkomen juist<br>◯ Grotendeels juist<br>◯ Weet ik niet<br>◯ Grotendeels onjuist<br>◯ Volkomen onjuist
6.34	Ik ben even gezond als andere mensen die ik ken	◯ Volkomen juist<br>◯ Grotendeels juist<br>◯ Weet ik niet<br>◯ Grotendeels onjuist<br>◯ Volkomen onjuist

Paget LDA, *et al. BMJ Open* 2019; 9:e030961. doi: 10.1136/bmjopen-2019-030961

6.35	Ik verwacht dat mijn gezondheid achteruit zal gaan	◯ Volkomen juist<br>◯ Grotendeels juist<br>◯ Weet ik niet<br>◯ Grotendeels onjuist<br>◯ Volkomen onjuist
6.36	Mijn gezondheid is uitstekend	◯ Volkomen juist<br>◯ Grotendeels juist<br>◯ Weet ik niet<br>◯ Grotendeels onjuist<br>◯ Volkomen onjuist

Vragenlijst PRIMA studie 26 weken - GAS

Number	Question	Answers
	Tijdens de eerste afspraak werd een duidelijk doel met u afgesproken. Dit werd tevens per email naar u gestuurd. Hoe is de situatie nu ten aanzien van de destijds gestelde doelstelling?	
7.1	Wat is nu de situatie ten aanzien van het tijdens de eerste afspraak afgesproken doelstelling (Goal attainment Scaling)?	◯ Achteruitgang (minder dan de utigangssituatie)<br>◯ Uitgangssituatie<br>◯ Minder dan het doel<br>◯ Doel<br>◯ Meer dan het doel<br>◯ Veel meer dan het doel

Vragenlijst PRIMA studie 26 weken - EQ-5D-3L

Number	Question	Answers
	Deze vragenlijst gaat over uw standpunten t.a.v. uw gezondheid. Met behulp van deze gegevens kan worden bijgehouden hoe u zich voelt en hoe goed u in staat bent uw gebruikelijke bezigheden uit te voeren. Beantwoord elke vraag door een antwoord aan te klikken. Als u niet zeker weet hoe u een vraag moet beantwoorden, geef dan het best mogelijke antwoord.	
8.1	Hoe is het met uw Mobiliteit gesteld?	◯ Ik heb geen problemen met lopen<br>◯ Ik heb enige problemen met lopen<br>◯ Ik ben bedlegerig

Paget LDA, *et al*. *BMJ Open* 2019; 9:e030961. doi: 10.1136/bmjopen-2019-030961

8.2	Hoe is het met uw Zelfzorg gesteld?	◯ Ik heb geen problemen om mijzelf te wassen of aan te kleden<br>◯ Ik heb enige problemen om mijzelf te wassen of aan te kleden<br>◯ Ik ben niet in staat om mijzelf te wassen of aan te kleden
8.3	Hoe is het met de Dagelijkse activiteiten (werk, studie, huishouden, gezins- en vrijetijdsactiviteiten) gesteld?	◯ Ik heb geen problemen met mijn dagelijkse activiteiten<br>◯ Ik heb enige problemen met mijn dagelijkse activiteiten<br>◯ Ik ben niet in staat om mijn dagelijkse activiteiten uit te voeren
8.4	Hoe is het met de Pijn/klachten gesteld?	◯ Ik heb geen pijn of andere klachten<br>◯ Ik heb matige pijn of andere klachten<br>◯ Ik heb zeer ernstige pijn of andere klachten
8.5	Hoe is het met de Stemming gesteld?	◯ Ik ben niet angstig of somber<br>◯ Ik ben matig angstig of somber<br>◯ Ik ben erg angstig of somber
	We willen weten hoe goed of slecht uw gezondheid VANDAAG is. Deze meetschaal loopt van 0 tot 100: 100 staat voor de BESTE gezondheid die u zich kunt voorstellen - 0 staat voor de SLECHTSTE gezondheid die u zich kunt voorstellen	
8.6	Uw Gezondheid vandaag	(0.00) (100.00)

Vragenlijst PRIMA studie 26 weken - AOS

Number	Question	Answers
	Instructies: De lijn naast elke vraag staat voor hoeveel PIJN u heeft in verschillende situaties. De linker kant (0) is “geen pijn” en de rechter kant (100) is “ergste pijn denkbaar”. Geef voor de onderstaande situaties op de lijn aan hoeveel PIJN u in de afgelopen week in de enkel had. Als een of meerdere van deze situaties niet van toepassing waren, dan kiest u de "niet van toepassing" optie.	
9.1	Wat was de hoogte van de ergste pijn in de afgelopen week?	(0.00) (100.00)
9.2	Heeft u pijn als u 's ochtends opstaat?	◯ Ja<br>◯ Nee<br>◯ Niet van toepassing

Paget LDA, *et al*. *BMJ Open* 2019; 9:e030961. doi: 10.1136/bmjopen-2019-030961

9.2.1	***If 'Heeft u pijn als u 's ochtends opstaat?' is equal to 'Ja' answer this question:***<br>Hoeveel pijn heeft u voordat u 's ochtends opstaat?	(0.00)	(100.00)
9.3	Heeft u pijn wanneer u op blote voeten loopt?	◯ Ja<br>◯ Nee<br>◯ Niet van toepassing	
9.3.1	***If 'Heeft u pijn wanneer u op blote voeten loopt?' is equal to 'Ja' answer this question:***<br>Hoeveel pijn heeft u als u op blote voeten loopt?	(0.00)	(100.00)
9.4	Heeft u pijn wanneer u op blote voeten staat?	◯ Ja<br>◯ Nee<br>◯ Niet van toepassing	
9.4.1	***If 'Heeft u pijn wanneer u op blote voeten staat?' is equal to 'Ja' answer this question:***<br>Hoeveel pijn heeft u als u op blote voeten staat?	(0.00)	(100.00)
9.5	Heeft u pijn wanneer u met schoenen loopt?	◯ Ja<br>◯ Nee<br>◯ Niet van toepassing	
9.5.1	***If 'Heeft u pijn wanneer u met schoenen loopt?' is equal to 'Ja' answer this question:***<br>Hoeveel pijn heeft u wanneer u met schoenen loopt?	(0.00)	(100.00)
9.6	Heeft u pijn wanneer u met schoenen staat?	◯ Ja<br>◯ Nee<br>◯ Niet van toepassing	
9.6.1	***If 'Heeft u pijn wanneer u met schoenen staat?' is equal to 'Ja' answer this question:***<br>Hoeveel pijn heeft u wanneer u met schoenen staat?	(0.00)	(100.00)
9.7	Heeft u pijn wanneer u loopt met steunzolen of een brace?	◯ Ja<br>◯ Nee<br>◯ Niet van toepassing	
9.7.1	***If 'Heeft u pijn wanneer u loopt met steunzolen of een brace?' is equal to 'Ja' answer this question:***<br>Hoeveel pijn heeft u wanneer u loopt met steunzolen of een brace?	(0.00)	(100.00)

Paget LDA, *et al. BMJ Open* 2019; 9:e030961. doi: 10.1136/bmjopen-2019-030961

9.8	Heeft u pijn wanneer u staat met steunzolen of een brace?	◯ Ja<br>◯ Nee<br>◯ Niet van toepassing	
9.8.1	***If 'Heeft u pijn wanneer u staat met steunzolen of een brace?' is equal to 'Ja' answer this question:***<br>Hoeveel pijn heeft u wanneer u staat met steunzolen of een brace?	(0.00)	(100.00)
9.9	Heeft u pijn aan het einde van de dag?	◯ Ja<br>◯ Nee<br>◯ Niet van toepassing	
9.9.1	***If 'Heeft u pijn aan het einde van de dag?' is equal to 'Ja' answer this question:***<br>Hoeveel pijn heeft u aan het einde van de dag?	(0.00)	(100.00)
	Instructies:De lijn naast elke vraag staat voor hoeveel MOEITE u heeft met verschillende activiteiten. De linker kant (0) is “Niet moeilijk” en de rechter kant (100) is “Te moeilijk, niet uitvoerbaar”. Geef voor de onderstaande activiteiten op de lijn aan hoeveel MOEITE u in de afgelopen week door enkelklachten had met onderstaande activiteiten. Als een situatie niet van toepassing is, kies dan de optie "niet van toepassing".		
9.10	Heeft u moeite met door het huis lopen?	◯ Ja<br>◯ Nee<br>◯ Niet van toepassing	
9.10.1	***If 'Heeft u moeite met door het huis lopen?' is equal to 'Ja' answer this question:***<br>Hoeveel moeite heeft u met door het huis lopen?	(0.00)	(100.00)
9.11	Heeft u moeite met buiten lopen op oneven ondergrond?	◯ Ja<br>◯ Nee<br>◯ Niet van toepassing	
9.11.1	***If 'Heeft u moeite met buiten lopen op oneven ondergrond?' is equal to 'Ja' answer this question:***<br>Hoeveel moeite heeft u met buiten lopen op oneven ondergrond?	(0.00)	(100.00)
9.12	Heeft u moeite met een paar honderd meter lopen?	◯ Ja<br>◯ Nee<br>◯ Niet van toepassing	
9.12.1	***If 'Heeft u moeite met een paar honderd meter lopen?' is equal to 'Ja' answer this question:***<br>Hoeveel moeite heeft u met een paar honderd meter lopen?	(0.00)	(100.00)

Paget LDA, *et al*. *BMJ Open* 2019; 9:e030961. doi: 10.1136/bmjopen-2019-030961

9.13	Heeft u moeite met een trap oplopen?	◯ Ja<br>◯ Nee<br>◯ Niet van toepassing	
9.13.1	***If 'Heeft u moeite met een trap oplopen?' is equal to 'Ja' answer this question:***<br>Hoeveel moeite heeft u met een trap oplopen?	(0.00)	(100.00)
9.14	Heeft u moeite met een trap aflopen?	◯ Ja<br>◯ Nee<br>◯ Niet van toepassing	
9.14.1	***If 'Heeft u moeite met een trap aflopen?' is equal to 'Ja' answer this question:***<br>Hoeveel moeite heeft u met een trap aflopen?	(0.00)	(100.00)
9.15	Heeft u moeite met op de tenen staan?	◯ Ja<br>◯ Nee<br>◯ Niet van toepassing	
9.15.1	***If 'Heeft u moeite met op de tenen staan?' is equal to 'Ja' answer this question:***<br>Hoeveel moeite heeft u met op de tenen staan?	(0.00)	(100.00)
9.16	Heeft u moeite met opstaan uit de stoel?	◯ Ja<br>◯ Nee<br>◯ Niet van toepassing	
9.16.1	***If 'Heeft u moeite met opstaan uit de stoel?' is equal to 'Ja' answer this question:***<br>Hoeveel moeite heeft u met opstaan uit de stoel?	(0.00)	(100.00)
9.17	Heeft u moeite met het op- of afstappen van stoepranden?	◯ Ja<br>◯ Nee<br>◯ Niet van toepassing	
9.17.1	***If 'Heeft u moeite met het op- of afstappen van stoepranden?' is equal to 'Ja' answer this question:***<br>Hoeveel moeite heeft u met het op- of afstappen van stoepranden?	(0.00)	(100.00)
9.18	Heeft u moeite met snel lopen of rennen?	◯ Ja<br>◯ Nee<br>◯ Niet van toepassing	

Paget LDA, *et al*. *BMJ Open* 2019; 9:e030961. doi: 10.1136/bmjopen-2019-030961

9.18.1	***If 'Heeft u moeite met snel lopen of rennen?' is equal to 'Ja' answer this question:*** Hoeveel moeite heeft u met snel lopen of rennen?	(0.00)	(100.00)

Vragenlijst PRIMA studie 26 weken - FAOS

Number	Question	Answers
	Deze lijst vraagt naar uw mening over uw voet/enkel. Uw antwoorden geven ons een beeld van uw voet/enkel klachten en hoe u in staat bent om alledaagse activiteiten uit te voeren in uw huidige situatie. Beantwoorden van een vraag doet u door het aanklikken van een vakje met het volgens u meest juiste antwoord (één vakje per vraag). Als u niet zeker weet hoe u een vraag moet beantwoorden, geeft u dan het antwoord dat volgens u het meest op uw situatie van toepassing is. Deze vraag heeft betrekking op het voorkomen van voet/enkel klachten in de afgelopen week.	
10.1	Is uw voet/enkel gezwollen?	◯ Nooit ◯ Zelden ◯ Soms ◯ Vaak ◯ Altijd
10.2	Voelt u gekraak of hoort u klikken of een ander vreemd geluid wanneer u de voet/enkel beweegt?	◯ Nooit ◯ Zelden ◯ Soms ◯ Vaak ◯ Altijd
10.3	Hapert uw enkel of blokkeert uw enkel ('op slot' gaan zitten) wanneer u deze beweegt?	◯ Nooit ◯ Zelden ◯ Soms ◯ Vaak ◯ Altijd
10.4	Kunt u de voet/enkel volledig strekken?	◯ Altijd ◯ Vaak ◯ Soms ◯ Zelden ◯ Nooit
10.5	Kunt u de voet volledig naar u toe buigen?	◯ Altijd ◯ Vaak ◯ Soms ◯ Zelden ◯ Nooit
10.6	In welke mate heeft u een stijf gevoel in de voet/enkel 's ochtends bij het wakker worden?	◯ Niet ◯ Mild ◯ Matig ◯ Ernstig ◯ Zeer ernstig
10.7	In welke mate heeft u een stijf gevoel in de voet/enkel na zitten, liggen of rusten later op de dag?	◯ Niet ◯ Mild ◯ Matig ◯ Ernstig ◯ Zeer ernstig
10.8	Hoe vaak heeft u pijn in uw voet/enkel?	◯ Nooit ◯ Maandelijks ◯ Wekelijks ◯ Dagelijks ◯ Altijd

Paget LDA, *et al. BMJ Open* 2019; 9:e030961. doi: 10.1136/bmjopen-2019-030961

10.9	Hoeveel voet/enkel pijn heeft u gehad in de afgelopen week bij draaien als uw voet/enkel op de grond staat?	◯ Geen<br>◯ Mild<br>◯ Matig<br>◯ Ernstig<br>◯ Zeer ernstig
10.10	Hoeveel voet/enkel pijn heeft u gehad in de afgelopen week bij het volledig uitstrekken van de voet/enkel?	◯ Geen<br>◯ Mild<br>◯ Matig<br>◯ Ernstig<br>◯ Zeer ernstig
10.11	Hoeveel voet/enkel pijn heeft u gehad in de afgelopen week bij het volledig naar u toe buigen/optrekken van de voet/enkel	◯ Geen<br>◯ Mild<br>◯ Matig<br>◯ Ernstig<br>◯ Zeer ernstig
10.12	Hoeveel voet/enkel pijn heeft u gehad in de afgelopen week bij het lopen op een vlakke ondergrond	◯ Geen<br>◯ Mild<br>◯ Matig<br>◯ Ernstig<br>◯ Zeer ernstig
10.13	Hoeveel voet/enkel pijn heeft u gehad in de afgelopen week bij het trap op- en trap af lopen?	◯ Geen<br>◯ Mild<br>◯ Matig<br>◯ Ernstig<br>◯ Zeer ernstig
10.14	Hoeveel voet/enkel pijn heeft u gehad in de afgelopen week 's nachts in bed?	◯ Geen<br>◯ Mild<br>◯ Matig<br>◯ Ernstig<br>◯ Zeer ernstig
10.15	Hoeveel voet/enkel pijn heeft u gehad in de afgelopen week bij het zitten of liggen?	◯ Geen<br>◯ Mild<br>◯ Matig<br>◯ Ernstig<br>◯ Zeer ernstig

Paget LDA, *et al. BMJ Open* 2019; 9:e030961. doi: 10.1136/bmjopen-2019-030961

10.16	Hoeveel voet/enkel pijn heeft u gehad in de afgelopen week bij het rechtop staan?	◯ Geen<br>◯ Mild<br>◯ Matig<br>◯ Ernstig<br>◯ Zeer ernstig
10.17	In welke mate werd u gehinderd bij het trap aflopen?	◯ Niet ◯ Mild ◯ Matig ◯ Ernstig ◯ Zeer ernstig
10.18	In welke mate werd u gehinderd bij het trap op lopen?	◯ Niet ◯ Mild ◯ Matig ◯ Ernstig ◯ Zeer ernstig
10.19	In welke mate werd u gehinderd als u vanuit een zittende positie ging staan?	◯ Niet ◯ Mild ◯ Matig ◯ Ernstig ◯ Zeer ernstig
10.20	In welke mate werd u gehinderd bij het staan?	◯ Niet ◯ Mild ◯ Matig ◯ Ernstig ◯ Zeer ernstig
10.21	In welke mate werd u gehinderd bij het naar de grond buigen/iets oprapen?	◯ Niet ◯ Mild ◯ Matig ◯ Ernstig ◯ Zeer ernstig
10.22	In welke mate werd u gehinderd bij het lopen op een vlakke ondergrond?	◯ Niet ◯ Mild ◯ Matig ◯ Ernstig ◯ Zeer ernstig
10.23	In welke mate werd u gehinderd bij het in- en uit de auto stappen?	◯ Niet ◯ Mild ◯ Matig ◯ Ernstig ◯ Zeer ernstig
10.24	In welke mate werd u gehinderd bij het boodschappen doen?	◯ Niet ◯ Mild ◯ Matig ◯ Ernstig ◯ Zeer ernstig
10.25	In welke mate werd u gehinderd bij sokken/panty's aantrekken?	◯ Niet ◯ Mild ◯ Matig ◯ Ernstig ◯ Zeer ernstig
10.26	In welke mate werd u gehinderd bij het opstaan uit bed?	◯ Niet ◯ Mild ◯ Matig ◯ Ernstig ◯ Zeer ernstig
10.27	In welke mate werd u gehinderd bij het sokken uittrekken?	◯ Niet ◯ Mild ◯ Matig ◯ Ernstig ◯ Zeer ernstig
10.28	In welke mate werd u gehinderd bij het in bed liggen (omdraaien, lange tijd uw voet/enkel in dezelfde positie houden)?	◯ Niet ◯ Mild ◯ Matig ◯ Ernstig ◯ Zeer ernstig

Paget LDA, *et al*. *BMJ Open* 2019; 9:e030961. doi: 10.1136/bmjopen-2019-030961

10.29	In welke mate werd u gehinderd bij het in/uit bad stappen?	◯ Niet ◯ Mild ◯ Matig ◯ Ernstig ◯ Zeer ernstig
10.30	In welke mate werd u gehinderd bij zitten?	◯ Niet ◯ Mild ◯ Matig ◯ Ernstig ◯ Zeer ernstig
10.31	In welke mate werd u gehinderd bij het toilet op en af gaan?	◯ Niet ◯ Mild ◯ Matig ◯ Ernstig ◯ Zeer ernstig
10.32	In welke mate werd u gehinderd bij zwaar huishoudelijk werk (bijvoorbeeld zware dozen sjouwen, vloer schrobben)?	◯ Niet ◯ Mild ◯ Matig ◯ Ernstig ◯ Zeer ernstig
10.33	In welke mate werd u gehinderd bij licht huishoudeijk werk (bijvoorbeeld koken, afstoffen)?	◯ Niet ◯ Mild ◯ Matig ◯ Ernstig ◯ Zeer ernstig
10.34	In welke mate werd u gehinderd bij hurken?	◯ Niet ◯ Mild ◯ Matig ◯ Ernstig ◯ Zeer ernstig
10.35	In welke mate werd u gehinderd bij hardlopen?	◯ Niet ◯ Mild ◯ Matig ◯ Ernstig ◯ Zeer ernstig
10.36	In welke mate werd u gehinderd bij springen?	◯ Niet ◯ Mild ◯ Matig ◯ Ernstig ◯ Zeer ernstig
10.37	In welke mate werd u gehinderd bij ronddraaien op uw aangedane voet/enkel?	◯ Niet ◯ Mild ◯ Matig ◯ Ernstig ◯ Zeer ernstig
10.38	In welke mate werd u gehinderd bij knielen?	◯ Niet ◯ Mild ◯ Matig ◯ Ernstig ◯ Zeer ernstig
10.39	Hoe vaak bent u zich bewust van uw voet/enkel probleem?	◯ Nooit ◯ Maandelijks ◯ Wekelijks ◯ Dagelijks ◯ Altijd
10.40	Heeft u uw leven veranderd om activiteiten te vermijden die schadelijk kunnen zijn voor uw voet/enkel?	◯ Niet<br>◯ Enigszins<br>◯ Matig<br>◯ Behoorlijk<br>◯ Volledig

Paget LDA, *et al. BMJ Open* 2019; 9:e030961. doi: 10.1136/bmjopen-2019-030961

10.41	In hoeverre kunt u op uw voet/enkel vertrouwen?	◯ Volledig<br>◯ Behoorlijk<br>◯ Matig<br>◯ Enigzins<br>◯ Niet
10.42	In het algemeen, in welke mate ondervindt u hinder van uw voet/enkel	◯ Geen<br>◯ Mild<br>◯ Matig<br>◯ Ernstig<br>◯ Zeer ernstig

Vragenlijst PRIMA studie 26 weken - PRODISQ - Gezondheid en werk

Number	Question	Answers
11.1	Wat is uw leeftijd?	[ ] jaar
11.2	Wat is uw geslacht?	◯ Man<br>◯ Vrouw
11.3	Wat is de hoogste opleiding die u heeft afgemaakt (Zoek uw hoogste opleiding en kruis het hokje daarvoor aan)?	☐ Ik heb geen school of opleiding afgemaakt<br>☐ Lagere school of basisschool<br>☐ Huishoudschool, vbo, lbo, lts, leao of lhno<br>☐ Mavo, mulo, ivo of vmbo<br>☐ Mbo, mts, meao, mhno, inas of intas<br>☐ Havo, vwo, hbs, mms, atheneum of gymnasium<br>☐ Hbo, hts, heao of hhno<br>☐ Universiteit<br>☐ Ik heb een andere opleiding afgemaakt, namelijk
11.3.1	***If 'Wat is de hoogste opleiding die u heeft afgemaakt (Zoek uw hoogste opleiding en kruis het hokje daarvoor aan)?' is equal to 'Ik heb een andere opleiding afgemaakt, namelijk' answer this question:***<br>Wat is de hoogste opleiding die u heeft afgemaakt?	[ ]

Paget LDA, *et al. BMJ Open* 2019; 9:e030961. doi: 10.1136/bmjopen-2019-030961

11.4	Wat doet u in het dagelijks leven?	☐ Ik zit op school, ik studeer<br>☐ Ik werk in loondienst<br>☐ Ik ben zelfstandig ondernemer<br>☐ Ik ben huisvrouw, huisman<br>☐ Ik ben werkloos<br>☐ Ik ben arbeidsongeschikt<br>☐ Ik ben met pensioen of prepensioen<br>☐ Ik doe iets anders, namelijk
11.4.1	***If 'Wat doet u in het dagelijks leven?' is equal to 'Ik ben arbeidsongeschikt' answer this question:***<br>Wat doet u in het dagelijks leven?	
11.4.2	***If 'Wat doet u in het dagelijks leven?' is equal to 'Ik doe iets anders, namelijk' answer this question:***<br>Wat doet u in het dagelijks leven?	
11.5	Hebt u betaald werk?	◯ Nee<br>◯ Ja
11.5.1	***If 'Hebt u betaald werk?' is equal to 'Ja' answer this question:***<br>Wat is uw beroep?	
11.5.2	***If 'Hebt u betaald werk?' is equal to 'Ja' answer this question:***<br>Hoeveel uur per week werkt u (Tel alleen de uren waarvoor u betaald wordt)?	uren
11.5.3	***If 'Hebt u betaald werk?' is equal to 'Ja' answer this question:***<br>Hoeveel dagen in de week werkt u?	Dagen
11.5.4	***If 'Hebt u betaald werk?' is equal to 'Ja' answer this question:***<br>Bent u in de afgelopen 4 weken afwezig geweest van uw werk omdat u ziek was?	◯ Nee<br>◯ Ja
11.5.4.1	***If 'Bent u in de afgelopen 4 weken afwezig geweest van uw werk omdat u ziek was?' is equal to 'Ja' answer this question:***<br>Bent u in de afgelopen 4 weken afwezig geweest van uw werk omdat u ziek was (Tel alleen de werkdagen in de afgelopen 4 weken)?	dagen afwezig geweest
11.5.4.2	***If 'Bent u in de afgelopen 4 weken afwezig geweest van uw werk omdat u ziek was?' is equal to 'Ja' answer this question:***<br>Was u langer dan de gehele periode van 4 weken afwezig van uw werk doordat u ziek was?	◯ Nee<br>◯ Ja

Paget LDA, *et al. BMJ Open* 2019; 9:e030961. doi: 10.1136/bmjopen-2019-030961

11.5.4.2.1	***If 'Was u langer dan de gehele periode van 4 weken afwezig van uw werk doordat u ziek was?' is equal to 'Ja' answer this question:*** Wanneer heeft u zich ziek gemeld?	[ ] [ ] [ ] *(dd-mm-yyyy)*
11.5.5	***If 'Hebt u betaald werk?' is equal to 'Ja' answer this question:*** Waren er in de afgelopen 4 weken, dagen waarop u wel gewerkt heeft, maar tijdens uw werk last had van lichamelijke of psychische problemen?	◯ Ja<br>◯ Nee
11.5.5.1	***If 'Waren er in de afgelopen 4 weken, dagen waarop u wel gewerkt heeft, maar tijdens uw werk last had van lichamelijke of psychische problemen?' is equal to 'Ja' answer this question:*** Op hoeveel werkdagen had u tijdens uw werk last van uw lichamelijke of psychische problemen?	[ ] werkdagen
11.5.5.2	***If 'Waren er in de afgelopen 4 weken, dagen waarop u wel gewerkt heeft, maar tijdens uw werk last had van lichamelijke of psychische problemen?' is equal to 'Ja' answer this question:*** Op de dagen dat u last had, kon u misschien niet zoveel werk doen als normaal. Hoeveel werk kon u op deze dagen gemiddeld doen?	Ik kon op deze dagen niks doen (0.00) — Ik kon net zoveel doen als normaal (10.00)
11.6	Waren er dagen waarop u minder onbetaald werk kon doen door uw lichamelijke of psychische problemen?	◯ Nee<br>◯ Ja
11.6.1	***If 'Waren er dagen waarop u minder onbetaald werk kon doen door uw lichamelijke of psychische problemen?' is equal to 'Ja' answer this question:*** Op hoeveel dagen was dit zo?	[ ] Dagen
11.6.2	***If 'Waren er dagen waarop u minder onbetaald werk kon doen door uw lichamelijke of psychische problemen?' is equal to 'Ja' answer this question:*** Stel dat iemand, bijvoorbeeld uw partner, familielid of een bekende, u op deze dagen had geholpen. En al het onbetaalde werk wat u niet kon doen, voor u had gedaan. Hoeveel uur was die persoon hier op deze dagen dan gemiddeld mee bezig geweest?	[ ] uur op deze dagen

Vragenlijst PRIMA studie 26 weken - PRODISQ - Zorggebruik

Number	Question	Answers
	Wij willen graag weten met welke dokters u in de afgelopen 3 maanden een afspraak had. Het gaat om afspraken voor uzelf. Ook andere zorgverleners tellen mee. Bijvoorbeeld de fysiotherapeut.	

Paget LDA, *et al. BMJ Open* 2019; 9:e030961. doi: 10.1136/bmjopen-2019-030961

12.1	Bent u in de afgelopen 3 maanden naar uw huisarts geweest?	◯ Nee<br>◯ Ja
12.1.1	***If 'Bent u in de afgelopen 3 maanden naar uw huisarts geweest?' is equal to 'Ja' answer this question:***<br>Hoeveel afspraken had u?	[ ] afspraken
12.2	Bent u in de afgelopen 3 maanden in contact geweest met een maatschappelijk werker?	◯ Nee<br>◯ Ja
12.2.1	***If 'Bent u in de afgelopen 3 maanden in contact geweest met een maatschappelijk werker?' is equal to 'Ja' answer this question:***<br>Hoeveel afspraken had u?	[ ] afspraken
12.3	Bent u in de afgelopen 3 maanden naar een fysiotherapeut geweest? Of een caesartherapeut, therapeut mensendieck of een manueel therapeut?	◯ Nee<br>◯ Ja
12.3.1	***If 'Bent u in de afgelopen 3 maanden naar een fysiotherapeut geweest? Of een caesartherapeut, therapeut mensendieck of een manueel therapeut?' is equal to 'Ja' answer this question:***<br>Hoeveel afspraken had u?	[ ] afspraken
12.4	Bent u in de afgelopen 3 maanden naar een ergotherapeut geweest?	◯ Nee<br>◯ Ja
12.4.1	***If 'Bent u in de afgelopen 3 maanden naar een ergotherapeut geweest?' is equal to 'Ja' answer this question:***<br>Hoeveel afspraken had u?	[ ] afspraken
12.5	Bent u in de afgelopen 3 maanden naar een logopedist geweest?	◯ Nee<br>◯ Ja
12.5.1	***If 'Bent u in de afgelopen 3 maanden naar een logopedist geweest?' is equal to 'Ja' answer this question:***<br>Hoeveel afspraken had u?	[ ] afspraken
12.6	Bent u in de afgelopen 3 maanden naar een diëtist geweest?	◯ Nee<br>◯ Ja
12.6.1	***If 'Bent u in de afgelopen 3 maanden naar een diëtist geweest?' is equal to 'Ja' answer this question:***<br>Hoeveel afspraken had u?	[ ] afspraken

Paget LDA, et al. BMJ Open 2019; 9:e030961. doi: 10.1136/bmjopen-2019-030961

12.7	Bent u in de afgelopen 3 maanden naar een homeopaat geweest? Of een acupuncturist?	◯ Nee<br>◯ Ja
12.7.1	***If 'Bent u in de afgelopen 3 maanden naar een homeopaat geweest? Of een acupuncturist?' is equal to 'Ja' answer this question:***<br>Hoeveel afspraken had u?	[ ] afspraken
12.8	Bent u in de afgelopen 3 maanden naar een psycholoog geweest? Of een psychotherapeut of psychiater?	◯ Nee<br>◯ Ja
12.8.1	***If 'Bent u in de afgelopen 3 maanden naar een psycholoog geweest? Of een psychotherapeut of psychiater?' is equal to 'Ja' answer this question:***<br>Hoeveel afspraken had u?	[ ] afspraken
12.9	Heeft u in de afgelopen 3 maanden afspraken gehad met de bedrijfsarts?	◯ Nee<br>◯ Ja
12.9.1	***If 'Heeft u in de afgelopen 3 maanden afspraken gehad met de bedrijfsarts?' is equal to 'Ja' answer this question:***<br>Hoeveel afspraken had u?	[ ] afspraken
12.10	Heeft u in de afgelopen 3 maanden hulp van de thuiszorg gehad?	◯ Ja<br>◯ Nee
12.10.1	***If 'Heeft u in de afgelopen 3 maanden hulp van de thuiszorg gehad?' is equal to 'Ja' answer this question:***<br>Wat voor hulp van de thuiszorg heeft u gehad in de afgelopen 3 maanden?	☐ Huishoudelijke hulp; voorbeeld: stofzuigen, bed opmaken, boodschappen doen<br>☐ Verzorging van uzelf; voorbeeld: hulp bij douchen of aankleden<br>☐ Verpleging; voorbeeld: verband omdoen, medicijnen geven, bloeddruk meten
12.10.1.1	***If 'Wat voor hulp van de thuiszorg heeft u gehad in de afgelopen 3 maanden?' is equal to 'Huishoudelijke hulp; voorbeeld: stofzuigen, bed opmaken, boodschappen doen' answer this question:***<br>Hoeveel weken heeft u deze thuiszorg gehad? Huishoudelijke hulp:	[ ] weken in de afgelopen 3 maanden
12.10.1.2	***If 'Wat voor hulp van de thuiszorg heeft u gehad in de afgelopen 3 maanden?' is equal to 'Verzorging van uzelf; voorbeeld: hulp bij douchen of aankleden' answer this question:***<br>Hoeveel weken heeft u deze thuiszorg gehad? Verzorging van uzelf:	[ ] weken in de afgelopen 3 maanden

Paget LDA, *et al*. *BMJ Open* 2019; 9:e030961. doi: 10.1136/bmjopen-2019-030961

12.10.1.3	***If 'Wat voor hulp van de thuiszorg heeft u gehad in de afgelopen 3 maanden?' is equal to 'Verpleging; voorbeeld: verband omdoen, medicijnen geven, bloeddruk meten' answer this question:*** Hoeveel weken heeft u deze thuiszorg gehad? Verpleging:	______ weken in de afgelopen 3 maanden
12.10.1.4	***If 'Wat voor hulp van de thuiszorg heeft u gehad in de afgelopen 3 maanden?' is equal to 'Huishoudelijke hulp; voorbeeld: stofzuigen, bed opmaken, boodschappen doen' answer this question:*** Hoeveel uur thuiszorg kreeg u in deze weken gemiddeld? Huishoudelijke hulp: gemiddeld	______ uur in de week
12.10.1.5	***If 'Wat voor hulp van de thuiszorg heeft u gehad in de afgelopen 3 maanden?' is equal to 'Verzorging van uzelf; voorbeeld: hulp bij douchen of aankleden' answer this question:*** Hoeveel uur thuiszorg kreeg u in deze weken gemiddeld? Verzorging van uzelf: gemiddeld	______ uur in de week
12.10.1.6	***If 'Wat voor hulp van de thuiszorg heeft u gehad in de afgelopen 3 maanden?' is equal to 'Verpleging; voorbeeld: verband omdoen, medicijnen geven, bloeddruk meten' answer this question:*** Hoeveel uur thuiszorg kreeg u in deze weken gemiddeld? Verpleging: gemiddeld	______ uur in de week
12.11	Heeft u in de afgelopen 3 maanden medicijnen gebruikt?	◯ Ja<br>◯ Nee
12.11.1	***If 'Heeft u in de afgelopen 3 maanden medicijnen gebruikt?' is equal to 'Ja' answer this question:*** Welke medicijnen heeft u in de afgelopen 3 maanden gebruikt?	
12.12	Bent u in de afgelopen 3 maanden op de spoedeisende eerste hulp van een ziekenhuis geweest? (Een andere naam voor spoedeisende eerste hulp is EHBO)	◯ Nee<br>◯ Ja
12.12.1	***If 'Bent u in de afgelopen 3 maanden op de spoedeisende eerste hulp van een ziekenhuis geweest? (Een andere naam voor spoedeisende eerste hulp is EHBO)' is equal to 'Ja' answer this question:*** Hoevaak bent u geweest?	______ keer
12.13	Bent u in de afgelopen 3 maanden met een ambulance naar het ziekenhuis gebracht? (Een andere naam voor ambulance is ziekenauto)	◯ Nee<br>◯ Ja

Paget LDA, *et al. BMJ Open* 2019; 9:e030961. doi: 10.1136/bmjopen-2019-030961

12.13.1	***If 'Bent u in de afgelopen 3 maanden met een ambulance naar het ziekenhuis gebracht? (Een andere naam voor ambulance is ziekenauto)' is equal to 'Ja' answer this question:***<br>Hoe vaak bent u naar het ziekenhuis gebracht?	[ ] keer
12.14	Had u in de afgelopen 3 maanden een afspraak bij de polikliniek van het ziekenhuis? (Het gaat om afspraken voor uzelf met een dokter. Bijvoorbeeld met de cardioloog, reumatoloog of neuroloog)	◯ Ja<br>◯ Nee
12.14.1	***If 'Had u in de afgelopen 3 maanden een afspraak bij de polikliniek van het ziekenhuis? (Het gaat om afspraken voor uzelf met een dokter. Bijvoorbeeld met de cardioloog, reumatoloog of neuroloog)' is equal to 'Ja' answer this question:***<br>Bij welke soorten dokters bent u in de afgelopen 3 maanden in het ziekenhuis geweest? En hoe vaak?	[ ]
12.15	Bent u in de afgelopen 3 maanden overdag in het ziekenhuis geweest voor een behandeling? (U bleef dus niet slapen. U kwam bijvoorbeeld voor een bloedtransfusie, nierdialyse of chemokuur)	◯ Ja<br>◯ Nee
12.15.1	***If 'Bent u in de afgelopen 3 maanden overdag in het ziekenhuis geweest voor een behandeling? (U bleef dus niet slapen. U kwam bijvoorbeeld voor een bloedtransfusie, nierdialyse of chemokuur)' is equal to 'Ja' answer this question:***<br>Voor welke soort behandeling was dit?	[ ]
12.15.2	***If 'Bent u in de afgelopen 3 maanden overdag in het ziekenhuis geweest voor een behandeling? (U bleef dus niet slapen. U kwam bijvoorbeeld voor een bloedtransfusie, nierdialyse of chemokuur)' is equal to 'Ja' answer this question:***<br>Hoeveel keer moest u in de afgelopen 3 maanden voor deze behandelingen naar het ziekenhuis?	[ ]
12.16	Bent u in de afgelopen 3 maanden ergens anders geweest voor een behandeling overdag? (U bleef dus niet slapen. U ging bijvoorbeeld naar de dagopvang van een woon-/zorgcentrum of een psychiatrische instelling. Of naar de dagbehandeling van een revalidatiecentrum)	◯ Ja<br>◯ Nee
12.16.1	***If 'Bent u in de afgelopen 3 maanden ergens anders geweest voor een behandeling overdag? (U bleef dus niet slapen. U ging bijvoorbeeld naar de dagopvang van een woon-/zorgcentrum of een psychiatrische instelling. Of naar de dagbehandeling van een revalidatiecentrum)' is equal to 'Ja' answer this question:***<br>Wat voor instelling was dit?	☐ Woon-/zorgcentrum<br>☐ Revalidatiecentrum<br>☐ Psychiatrische instelling<br>☐ Een andere instelling, namelijk

Paget LDA, *et al. BMJ Open* 2019; 9:e030961. doi: 10.1136/bmjopen-2019-030961

12.16.1.1	***If 'Wat voor instelling was dit?' is equal to 'Een andere instelling, namelijk' answer this question:***<br>Wat voor instelling was dit?	
12.16.1.2	***If 'Wat voor instelling was dit?' is equal to 'Woon-/zorgcentrum' answer this question:***<br>Hoe vaak moest u hier in de afgelopen 3 maanden naartoe? Naar het woon-/zorgcentrum:	keer in de afgelopen 3 maanden
12.16.1.3	***If 'Wat voor instelling was dit?' is equal to 'Revalidatiecentrum' answer this question:***<br>Hoe vaak moest u hier in de afgelopen 3 maanden naartoe? Naar het revalidatiecentrum:	keer in de afgelopen 3 maanden
12.16.1.4	***If 'Wat voor instelling was dit?' is equal to 'Psychiatrische instelling' answer this question:***<br>Hoe vaak moest u hier in de afgelopen 3 maanden naartoe? Naar de psychiatrische instelling:	keer in de afgelopen 3 maanden
12.16.1.5	***If 'Wat voor instelling was dit?' is equal to 'Een andere instelling, namelijk' answer this question:***<br>Hoe vaak moest u hier in de afgelopen 3 maanden naartoe? Naar de andere instelling:	keer in de afgelopen 3 maanden
12.17	Heeft u in de afgelopen 3 maanden weleens in het ziekenhuis gelegen? (U moest dus blijven slapen. Bijvoorbeeld omdat u geopereerd was en niet meteen naar huis kon)	◯ Ja<br>◯ Nee
12.17.1	***If 'Heeft u in de afgelopen 3 maanden weleens in het ziekenhuis gelegen? (U moest dus blijven slapen. Bijvoorbeeld omdat u geopereerd was en niet meteen naar huis kon)' is equal to 'Ja' answer this question:***<br>Hoe vaak heeft u in de afgelopen 3 maanden in het ziekenhuis gelegen?	keer in de afgelopen 3 maanden
12.17.2	***If 'Heeft u in de afgelopen 3 maanden weleens in het ziekenhuis gelegen? (U moest dus blijven slapen. Bijvoorbeeld omdat u geopereerd was en niet meteen naar huis kon)' is equal to 'Ja' answer this question:***<br>Hoe lang heeft u in het ziekenhuis gelegen?	dagen in de afgelopen 3 maanden
12.18	Moest u in de afgelopen 3 maanden ergens anders blijven slapen voor uw gezondheid? (Bijvoorbeeld in een woon-/zorgcentrum, psychiatrische instelling of revalidatiecentrum)	◯ Ja<br>◯ Nee

Paget LDA, *et al. BMJ Open* 2019; 9:e030961. doi: 10.1136/bmjopen-2019-030961

12.18.1	***If 'Moest u in de afgelopen 3 maanden ergens anders blijven slapen voor uw gezondheid? (Bijvoorbeeld in een woon-/zorgcentrum, psychiatrische instelling of revalidatiecentrum)' is equal to 'Ja' answer this question:*** Wat voor instelling was dit?	☐ Woon-/zorgcentrum<br>☐ Revalidatiecentrum<br>☐ Psychiatrische instelling<br>☐ Een andere instelling, namelijk
12.18.1.1	***If 'Wat voor instelling was dit?' is equal to 'Een andere instelling, namelijk' answer this question:*** Een andere instelling, namelijk	
12.18.1.2	***If 'Wat voor instelling was dit?' is equal to 'Woon-/zorgcentrum' answer this question:*** Hoe lang bent u in deze instelling geweest? In het woon-/zorgcentrum:	______ dagen in de afgelopen 3 maanden
12.18.1.3	***If 'Wat voor instelling was dit?' is equal to 'Revalidatiecentrum' answer this question:*** Hoe lang bent u in deze instelling geweest? In het revalidatiecentrum:	______ dagen in de afgelopen 3 maanden
12.18.1.4	***If 'Wat voor instelling was dit?' is equal to 'Psychiatrische instelling' answer this question:*** Hoe lang bent u in deze instelling geweest? In de psychiatrische instelling:	______ dagen in de afgelopen 3 maanden
12.18.1.5	***If 'Wat voor instelling was dit?' is equal to 'Een andere instelling, namelijk' answer this question:*** Hoe lang bent u in deze instelling geweest? In de andere instelling:	______ dagen in de afgelopen 3 maanden
12.19	Heeft u in de afgelopen 3 maanden hulp gekregen van een familielid of een bekende vanwege uw lichamelijke of psychische problemen?	◯ Ja<br>◯ Nee
12.19.1	***If 'Heeft u in de afgelopen 3 maanden hulp gekregen van een familielid of een bekende vanwege uw lichamelijke of psychische problemen?' is equal to 'Ja' answer this question:*** Wat voor hulp van familieleden of bekenden heeft u gehad in de afgelopen 3 maanden?	☐ Huishoudelijke hulp - voorbeeld: stofzuigen, bed opmaken, boodschappen doen, klaarmaken van eten en drinken, verzorgen van kinderen<br>☐ Verzorging van uzelf - voorbeeld: hulp bij douchen of aankleden, hulp bij het eten en drinken of het geven van medicijnen<br>☐ Praktische hulp - voorbeeld: ondersteuning bij wandelen, het maken van uitstapjes of bezoekjes aan bekenden, bezoeken aan de huisarts of het ziekenhuis, het regelen van hulp of het regelen van financiële zaken

Paget LDA, *et al*. *BMJ Open* 2019; 9:e030961. doi: 10.1136/bmjopen-2019-030961

12.19.1.1	***If 'Wat voor hulp van familieleden of bekenden heeft u gehad in de afgelopen 3 maanden?' is equal to 'Huishoudelijke hulp - voorbeeld: stofzuigen, bed opmaken, boodschappen doen, klaarmaken van eten en drinken, verzorgen van kinderen' answer this question:*** Hoeveel weken heeft u deze hulp gehad? Huishoudelijke hulp:	______ weken in de afgelopen 3 maanden
12.19.1.2	***If 'Wat voor hulp van familieleden of bekenden heeft u gehad in de afgelopen 3 maanden?' is equal to 'Verzorging van uzelf - voorbeeld: hulp bij douchen of aankleden, hulp bij het eten en drinken of het geven van medicijnen' answer this question:*** Hoeveel weken heeft u deze hulp gehad? Verzorging van uzelf:	______ weken in de afgelopen 3 maanden
12.19.1.3	***If 'Wat voor hulp van familieleden of bekenden heeft u gehad in de afgelopen 3 maanden?' is equal to 'Praktische hulp - voorbeeld: ondersteuning bij wandelen, het maken van uitstapjes of bezoekjes aan bekenden, bezoeken aan de huisarts of het ziekenhuis, het regelen van hulp of het regelen van financiële zaken' answer this question:*** Hoeveel uur hulp kreeg u in deze weken gemiddeld? Praktische hulp:	______ weken in de afgelopen 3 maanden
12.19.1.4	***If 'Wat voor hulp van familieleden of bekenden heeft u gehad in de afgelopen 3 maanden?' is equal to 'Huishoudelijke hulp - voorbeeld: stofzuigen, bed opmaken, boodschappen doen, klaarmaken van eten en drinken, verzorgen van kinderen' answer this question:*** Hoeveel uur hulp kreeg u in deze weken gemiddeld? Huishoudelijke hulp: gemiddeld	______ uur in de week
12.19.1.5	***If 'Wat voor hulp van familieleden of bekenden heeft u gehad in de afgelopen 3 maanden?' is equal to 'Verzorging van uzelf - voorbeeld: hulp bij douchen of aankleden, hulp bij het eten en drinken of het geven van medicijnen' answer this question:*** Hoeveel uur hulp kreeg u in deze weken gemiddeld? Verzorging van uzelf: gemiddeld	______ uur in de week
12.19.1.6	***If 'Wat voor hulp van familieleden of bekenden heeft u gehad in de afgelopen 3 maanden?' is equal to 'Praktische hulp - voorbeeld: ondersteuning bij wandelen, het maken van uitstapjes of bezoekjes aan bekenden, bezoeken aan de huisarts of het ziekenhuis, het regelen van hulp of het regelen van financiële zaken' answer this question:*** Hoeveel uur hulp kreeg u in deze weken gemiddeld? Praktische hulp: gemiddeld	______ uur in de week

Paget LDA, *et al*. *BMJ Open* 2019; 9:e030961. doi: 10.1136/bmjopen-2019-030961

Vragenlijst PRIMA studie 26 weken - Dank

Number	Question	Answers
13.1	Hartelijk dank voor de tijd die u heeft genomen om de vragenlijsten in te vullen. Indien u nog opmerkingen heeft kunt u deze hierin plaatsen.	

Survey 'Vragenlijst PRIMA studie 39 weken'

Vragenlijst PRIMA studie 39 weken - PRODISQ - Gezondheid en werk

Number	Question	Answers
1.1	Wat is uw leeftijd?	jaar
1.2	Wat is uw geslacht?	◯ Man<br>◯ Vrouw
1.3	Wat is de hoogste opleiding die u heeft afgemaakt (Zoek uw hoogste opleiding en kruis het hokje daarvoor aan)?	☐ Ik heb geen school of opleiding afgemaakt<br>☐ Lagere school of basisschool<br>☐ Huishoudschool, vbo, lbo, lts, leao of lhno<br>☐ Mavo, mulo, ivo of vmbo<br>☐ Mbo, mts, meao, mhno, inas of intas<br>☐ Havo, vwo, hbs, mms, atheneum of gymnasium<br>☐ Hbo, hts, heao of hhno<br>☐ Universiteit<br>☐ Ik heb een andere opleiding afgemaakt, namelijk
1.3.1	***If 'Wat is de hoogste opleiding die u heeft afgemaakt (Zoek uw hoogste opleiding en kruis het hokje daarvoor aan)?' is equal to 'Ik heb een andere opleiding afgemaakt, namelijk' answer this question:***<br>Wat is de hoogste opleiding die u heeft afgemaakt?	

Paget LDA, *et al. BMJ Open* 2019; 9:e030961. doi: 10.1136/bmjopen-2019-030961

1.4	Wat doet u in het dagelijks leven?	☐ Ik zit op school, ik studeer<br>☐ Ik werk in loondienst<br>☐ Ik ben zelfstandig ondernemer<br>☐ Ik ben huisvrouw, huisman<br>☐ Ik ben werkloos<br>☐ Ik ben arbeidsongeschikt<br>☐ Ik ben met pensioen of prepensioen<br>☐ Ik doe iets anders, namelijk
1.4.1	***If 'Wat doet u in het dagelijks leven?' is equal to 'Ik ben arbeidsongeschikt' answer this question:***<br>Wat doet u in het dagelijks leven?	
1.4.2	***If 'Wat doet u in het dagelijks leven?' is equal to 'Ik doe iets anders, namelijk' answer this question:***<br>Wat doet u in het dagelijks leven?	
1.5	Hebt u betaald werk?	○ Nee<br>○ Ja
1.5.1	***If 'Hebt u betaald werk?' is equal to 'Ja' answer this question:***<br>Wat is uw beroep?	
1.5.2	***If 'Hebt u betaald werk?' is equal to 'Ja' answer this question:***<br>Hoeveel uur per week werkt u (Tel alleen de uren waarvoor u betaald wordt)?	uren
1.5.3	***If 'Hebt u betaald werk?' is equal to 'Ja' answer this question:***<br>Hoeveel dagen in de week werkt u?	Dagen
1.5.4	***If 'Hebt u betaald werk?' is equal to 'Ja' answer this question:***<br>Bent u in de afgelopen 4 weken afwezig geweest van uw werk omdat u ziek was?	○ Nee<br>○ Ja
1.5.4.1	***If 'Bent u in de afgelopen 4 weken afwezig geweest van uw werk omdat u ziek was?' is equal to 'Ja' answer this question:***<br>Bent u in de afgelopen 4 weken afwezig geweest van uw werk omdat u ziek was (Tel alleen de werkdagen in de afgelopen 4 weken)?	dagen afwezig geweest
1.5.4.2	***If 'Bent u in de afgelopen 4 weken afwezig geweest van uw werk omdat u ziek was?' is equal to 'Ja' answer this question:***<br>Was u langer dan de gehele periode van 4 weken afwezig van uw werk doordat u ziek was?	○ Nee<br>○ Ja

Paget LDA, *et al. BMJ Open* 2019; 9:e030961. doi: 10.1136/bmjopen-2019-030961

1.5.4.2.1	***If 'Was u langer dan de gehele periode van 4 weken afwezig van uw werk doordat u ziek was?' is equal to 'Ja' answer this question:*** Wanneer heeft u zich ziek gemeld?	*(dd-mm-yyyy)*
1.5.5	***If 'Hebt u betaald werk?' is equal to 'Ja' answer this question:*** Waren er in de afgelopen 4 weken, dagen waarop u wel gewerkt heeft, maar tijdens uw werk last had van lichamelijke of psychische problemen?	◯ Ja<br>◯ Nee
1.5.5.1	***If 'Waren er in de afgelopen 4 weken, dagen waarop u wel gewerkt heeft, maar tijdens uw werk last had van lichamelijke of psychische problemen?' is equal to 'Ja' answer this question:*** Op hoeveel werkdagen had u tijdens uw werk last van uw lichamelijke of psychische problemen?	werkdagen
1.5.5.2	***If 'Waren er in de afgelopen 4 weken, dagen waarop u wel gewerkt heeft, maar tijdens uw werk last had van lichamelijke of psychische problemen?' is equal to 'Ja' answer this question:*** Op de dagen dat u last had, kon u misschien niet zoveel werk doen als normaal. Hoeveel werk kon u op deze dagen gemiddeld doen?	Ik kon op deze dagen niks doen (0.00) — Ik kon net zoveel doen als normaal (10.00)
1.6	Waren er dagen waarop u minder onbetaald werk kon doen door uw lichamelijke of psychische problemen?	◯ Nee<br>◯ Ja
1.6.1	***If 'Waren er dagen waarop u minder onbetaald werk kon doen door uw lichamelijke of psychische problemen?' is equal to 'Ja' answer this question:*** Op hoeveel dagen was dit zo?	Dagen
1.6.2	***If 'Waren er dagen waarop u minder onbetaald werk kon doen door uw lichamelijke of psychische problemen?' is equal to 'Ja' answer this question:*** Stel dat iemand, bijvoorbeeld uw partner, familielid of een bekende, u op deze dagen had geholpen. En al het onbetaalde werk wat u niet kon doen, voor u had gedaan. Hoeveel uur was die persoon hier op deze dagen dan gemiddeld mee bezig geweest?	uur op deze dagen

Vragenlijst PRIMA studie 39 weken - PRODISQ - Zorggebruik

Number	Question	Answers
	Wij willen graag weten met welke dokters u in de afgelopen 3 maanden een afspraak had. Het gaat om afspraken voor uzelf. Ook andere zorgverleners tellen mee. Bijvoorbeeld de fysiotherapeut.	

Paget LDA, *et al. BMJ Open* 2019; 9:e030961. doi: 10.1136/bmjopen-2019-030961

2.1	Bent u in de afgelopen 3 maanden naar uw huisarts geweest?	◯ Nee<br>◯ Ja
2.1.1	***If 'Bent u in de afgelopen 3 maanden naar uw huisarts geweest?' is equal to 'Ja' answer this question:***<br>Hoeveel afspraken had u?	[ ] afspraken
2.2	Bent u in de afgelopen 3 maanden in contact geweest met een maatschappelijk werker?	◯ Nee<br>◯ Ja
2.2.1	***If 'Bent u in de afgelopen 3 maanden in contact geweest met een maatschappelijk werker?' is equal to 'Ja' answer this question:***<br>Hoeveel afspraken had u?	[ ] afspraken
2.3	Bent u in de afgelopen 3 maanden naar een fysiotherapeut geweest? Of een caesartherapeut, therapeut mensendieck of een manueel therapeut?	◯ Nee<br>◯ Ja
2.3.1	***If 'Bent u in de afgelopen 3 maanden naar een fysiotherapeut geweest? Of een caesartherapeut, therapeut mensendieck of een manueel therapeut?' is equal to 'Ja' answer this question:***<br>Hoeveel afspraken had u?	[ ] afspraken
2.4	Bent u in de afgelopen 3 maanden naar een ergotherapeut geweest?	◯ Nee<br>◯ Ja
2.4.1	***If 'Bent u in de afgelopen 3 maanden naar een ergotherapeut geweest?' is equal to 'Ja' answer this question:***<br>Hoeveel afspraken had u?	[ ] afspraken
2.5	Bent u in de afgelopen 3 maanden naar een logopedist geweest?	◯ Nee<br>◯ Ja
2.5.1	***If 'Bent u in de afgelopen 3 maanden naar een logopedist geweest?' is equal to 'Ja' answer this question:***<br>Hoeveel afspraken had u?	[ ] afspraken
2.6	Bent u in de afgelopen 3 maanden naar een diëtist geweest?	◯ Nee<br>◯ Ja
2.6.1	***If 'Bent u in de afgelopen 3 maanden naar een diëtist geweest?' is equal to 'Ja' answer this question:***<br>Hoeveel afspraken had u?	[ ] afspraken

Paget LDA, *et al. BMJ Open* 2019; 9:e030961. doi: 10.1136/bmjopen-2019-030961

2.7	Bent u in de afgelopen 3 maanden naar een homeopaat geweest? Of een acupuncturist?	◯ Nee<br>◯ Ja
2.7.1	***If 'Bent u in de afgelopen 3 maanden naar een homeopaat geweest? Of een acupuncturist?' is equal to 'Ja' answer this question:***<br>Hoeveel afspraken had u?	[ ] afspraken
2.8	Bent u in de afgelopen 3 maanden naar een psycholoog geweest? Of een psychotherapeut of psychiater?	◯ Nee<br>◯ Ja
2.8.1	***If 'Bent u in de afgelopen 3 maanden naar een psycholoog geweest? Of een psychotherapeut of psychiater?' is equal to 'Ja' answer this question:***<br>Hoeveel afspraken had u?	[ ] afspraken
2.9	Heeft u in de afgelopen 3 maanden afspraken gehad met de bedrijfsarts?	◯ Nee<br>◯ Ja
2.9.1	***If 'Heeft u in de afgelopen 3 maanden afspraken gehad met de bedrijfsarts?' is equal to 'Ja' answer this question:***<br>Hoeveel afspraken had u?	[ ] afspraken
2.10	Heeft u in de afgelopen 3 maanden hulp van de thuiszorg gehad?	◯ Ja<br>◯ Nee
2.10.1	***If 'Heeft u in de afgelopen 3 maanden hulp van de thuiszorg gehad?' is equal to 'Ja' answer this question:***<br>Wat voor hulp van de thuiszorg heeft u gehad in de afgelopen 3 maanden?	☐ Huishoudelijke hulp; voorbeeld: stofzuigen, bed opmaken, boodschappen doen<br>☐ Verzorging van uzelf; voorbeeld: hulp bij douchen of aankleden<br>☐ Verpleging; voorbeeld: verband omdoen, medicijnen geven, bloeddruk meten
2.10.1.1	***If 'Wat voor hulp van de thuiszorg heeft u gehad in de afgelopen 3 maanden?' is equal to 'Huishoudelijke hulp; voorbeeld: stofzuigen, bed opmaken, boodschappen doen' answer this question:***<br>Hoeveel weken heeft u deze thuiszorg gehad? Huishoudelijke hulp:	[ ] weken in de afgelopen 3 maanden
2.10.1.2	***If 'Wat voor hulp van de thuiszorg heeft u gehad in de afgelopen 3 maanden?' is equal to 'Verzorging van uzelf; voorbeeld: hulp bij douchen of aankleden' answer this question:***<br>Hoeveel weken heeft u deze thuiszorg gehad? Verzorging van uzelf:	[ ] weken in de afgelopen 3 maanden

Paget LDA, *et al*. *BMJ Open* 2019; 9:e030961. doi: 10.1136/bmjopen-2019-030961

2.10.1.3	***If 'Wat voor hulp van de thuiszorg heeft u gehad in de afgelopen 3 maanden?' is equal to 'Verpleging; voorbeeld: verband omdoen, medicijnen geven, bloeddruk meten' answer this question:*** Hoeveel weken heeft u deze thuiszorg gehad? Verpleging:	[ ] weken in de afgelopen 3 maanden
2.10.1.4	***If 'Wat voor hulp van de thuiszorg heeft u gehad in de afgelopen 3 maanden?' is equal to 'Huishoudelijke hulp; voorbeeld: stofzuigen, bed opmaken, boodschappen doen' answer this question:*** Hoeveel uur thuiszorg kreeg u in deze weken gemiddeld? Huishoudelijke hulp: gemiddeld	[ ] uur in de week
2.10.1.5	***If 'Wat voor hulp van de thuiszorg heeft u gehad in de afgelopen 3 maanden?' is equal to 'Verzorging van uzelf; voorbeeld: hulp bij douchen of aankleden' answer this question:*** Hoeveel uur thuiszorg kreeg u in deze weken gemiddeld? Verzorging van uzelf: gemiddeld	[ ] uur in de week
2.10.1.6	***If 'Wat voor hulp van de thuiszorg heeft u gehad in de afgelopen 3 maanden?' is equal to 'Verpleging; voorbeeld: verband omdoen, medicijnen geven, bloeddruk meten' answer this question:*** Hoeveel uur thuiszorg kreeg u in deze weken gemiddeld? Verpleging: gemiddeld	[ ] uur in de week
2.11	Heeft u in de afgelopen 3 maanden medicijnen gebruikt?	◯ Ja<br>◯ Nee
2.11.1	***If 'Heeft u in de afgelopen 3 maanden medicijnen gebruikt?' is equal to 'Ja' answer this question:*** Welke medicijnen heeft u in de afgelopen 3 maanden gebruikt?	[ ]
2.12	Bent u in de afgelopen 3 maanden op de spoedeisende eerste hulp van een ziekenhuis geweest? (Een andere naam voor spoedeisende eerste hulp is EHBO)	◯ Nee<br>◯ Ja
2.12.1	***If 'Bent u in de afgelopen 3 maanden op de spoedeisende eerste hulp van een ziekenhuis geweest? (Een andere naam voor spoedeisende eerste hulp is EHBO)' is equal to 'Ja' answer this question:*** Hoevaak bent u geweest?	[ ] keer
2.13	Bent u in de afgelopen 3 maanden met een ambulance naar het ziekenhuis gebracht? (Een andere naam voor ambulance is ziekenauto)	◯ Nee<br>◯ Ja

Paget LDA, *et al. BMJ Open* 2019; 9:e030961. doi: 10.1136/bmjopen-2019-030961

2.13.1	***If 'Bent u in de afgelopen 3 maanden met een ambulance naar het ziekenhuis gebracht? (Een andere naam voor ambulance is ziekenauto)' is equal to 'Ja' answer this question:***<br>Hoe vaak bent u naar het ziekenhuis gebracht?	______ keer
2.14	Had u in de afgelopen 3 maanden een afspraak bij de polikliniek van het ziekenhuis? (Het gaat om afspraken voor uzelf met een dokter. Bijvoorbeeld met de cardioloog, reumatoloog of neuroloog)	◯ Ja<br>◯ Nee
2.14.1	***If 'Had u in de afgelopen 3 maanden een afspraak bij de polikliniek van het ziekenhuis? (Het gaat om afspraken voor uzelf met een dokter. Bijvoorbeeld met de cardioloog, reumatoloog of neuroloog)' is equal to 'Ja' answer this question:***<br>Bij welke soorten dokters bent u in de afgelopen 3 maanden in het ziekenhuis geweest? En hoe vaak?	
2.15	Bent u in de afgelopen 3 maanden overdag in het ziekenhuis geweest voor een behandeling? (U bleef dus niet slapen. U kwam bijvoorbeeld voor een bloedtransfusie, nierdialyse of chemokuur)	◯ Ja<br>◯ Nee
2.15.1	***If 'Bent u in de afgelopen 3 maanden overdag in het ziekenhuis geweest voor een behandeling? (U bleef dus niet slapen. U kwam bijvoorbeeld voor een bloedtransfusie, nierdialyse of chemokuur)' is equal to 'Ja' answer this question:***<br>Voor welke soort behandeling was dit?	
2.15.2	***If 'Bent u in de afgelopen 3 maanden overdag in het ziekenhuis geweest voor een behandeling? (U bleef dus niet slapen. U kwam bijvoorbeeld voor een bloedtransfusie, nierdialyse of chemokuur)' is equal to 'Ja' answer this question:***<br>Hoeveel keer moest u in de afgelopen 3 maanden voor deze behandelingen naar het ziekenhuis?	
2.16	Bent u in de afgelopen 3 maanden ergens anders geweest voor een behandeling overdag? (U bleef dus niet slapen. U ging bijvoorbeeld naar de dagopvang van een woon-/zorgcentrum of een psychiatrische instelling. Of naar de dagbehandeling van een revalidatiecentrum)	◯ Ja<br>◯ Nee
2.16.1	***If 'Bent u in de afgelopen 3 maanden ergens anders geweest voor een behandeling overdag? (U bleef dus niet slapen. U ging bijvoorbeeld naar de dagopvang van een woon-/zorgcentrum of een psychiatrische instelling. Of naar de dagbehandeling van een revalidatiecentrum)' is equal to 'Ja' answer this question:***<br>Wat voor instelling was dit?	☐ Woon-/zorgcentrum<br>☐ Revalidatiecentrum<br>☐ Psychiatrische instelling<br>☐ Een andere instelling, namelijk

Paget LDA, *et al. BMJ Open* 2019; 9:e030961. doi: 10.1136/bmjopen-2019-030961

2.16.1.1	***If 'Wat voor instelling was dit?' is equal to 'Een andere instelling, namelijk' answer this question:***<br>Wat voor instelling was dit?	
2.16.1.2	***If 'Wat voor instelling was dit?' is equal to 'Woon-/zorgcentrum' answer this question:***<br>Hoe vaak moest u hier in de afgelopen 3 maanden naartoe? Naar het woon-/zorgcentrum:	keer in de afgelopen 3 maanden
2.16.1.3	***If 'Wat voor instelling was dit?' is equal to 'Revalidatiecentrum' answer this question:***<br>Hoe vaak moest u hier in de afgelopen 3 maanden naartoe? Naar het revalidatiecentrum:	keer in de afgelopen 3 maanden
2.16.1.4	***If 'Wat voor instelling was dit?' is equal to 'Psychiatrische instelling' answer this question:***<br>Hoe vaak moest u hier in de afgelopen 3 maanden naartoe? Naar de psychiatrische instelling:	keer in de afgelopen 3 maanden
2.16.1.5	***If 'Wat voor instelling was dit?' is equal to 'Een andere instelling, namelijk' answer this question:***<br>Hoe vaak moest u hier in de afgelopen 3 maanden naartoe? Naar de andere instelling:	keer in de afgelopen 3 maanden
2.17	Heeft u in de afgelopen 3 maanden weleens in het ziekenhuis gelegen? (U moest dus blijven slapen. Bijvoorbeeld omdat u geopereerd was en niet meteen naar huis kon)	○ Ja<br>○ Nee
2.17.1	***If 'Heeft u in de afgelopen 3 maanden weleens in het ziekenhuis gelegen? (U moest dus blijven slapen. Bijvoorbeeld omdat u geopereerd was en niet meteen naar huis kon)' is equal to 'Ja' answer this question:***<br>Hoe vaak heeft u in de afgelopen 3 maanden in het ziekenhuis gelegen?	keer in de afgelopen 3 maanden
2.17.2	***If 'Heeft u in de afgelopen 3 maanden weleens in het ziekenhuis gelegen? (U moest dus blijven slapen. Bijvoorbeeld omdat u geopereerd was en niet meteen naar huis kon)' is equal to 'Ja' answer this question:***<br>Hoe lang heeft u in het ziekenhuis gelegen?	dagen in de afgelopen 3 maanden
2.18	Moest u in de afgelopen 3 maanden ergens anders blijven slapen voor uw gezondheid? (Bijvoorbeeld in een woon-/zorgcentrum, psychiatrische instelling of revalidatiecentrum)	○ Ja<br>○ Nee

Paget LDA, *et al. BMJ Open* 2019; 9:e030961. doi: 10.1136/bmjopen-2019-030961

2.18.1	***If 'Moest u in de afgelopen 3 maanden ergens anders blijven slapen voor uw gezondheid? (Bijvoorbeeld in een woon-/zorgcentrum, psychiatrische instelling of revalidatiecentrum)' is equal to 'Ja' answer this question:***<br>Wat voor instelling was dit?	☐ Woon-/zorgcentrum<br>☐ Revalidatiecentrum<br>☐ Psychiatrische instelling<br>☐ Een andere instelling, namelijk
2.18.1.1	***If 'Wat voor instelling was dit?' is equal to 'Een andere instelling, namelijk' answer this question:***<br>Een andere instelling, namelijk	
2.18.1.2	***If 'Wat voor instelling was dit?' is equal to 'Woon-/zorgcentrum' answer this question:***<br>Hoe lang bent u in deze instelling geweest? In het woon-/zorgcentrum:	______ dagen in de afgelopen 3 maanden
2.18.1.3	***If 'Wat voor instelling was dit?' is equal to 'Revalidatiecentrum' answer this question:***<br>Hoe lang bent u in deze instelling geweest? In het revalidatiecentrum:	______ dagen in de afgelopen 3 maanden
2.18.1.4	***If 'Wat voor instelling was dit?' is equal to 'Psychiatrische instelling' answer this question:***<br>Hoe lang bent u in deze instelling geweest? In de psychiatrische instelling:	______ dagen in de afgelopen 3 maanden
2.18.1.5	***If 'Wat voor instelling was dit?' is equal to 'Een andere instelling, namelijk' answer this question:***<br>Hoe lang bent u in deze instelling geweest? In de andere instelling:	______ dagen in de afgelopen 3 maanden
2.19	Heeft u in de afgelopen 3 maanden hulp gekregen van een familielid of een bekende vanwege uw lichamelijke of psychische problemen?	◯ Ja<br>◯ Nee
2.19.1	***If 'Heeft u in de afgelopen 3 maanden hulp gekregen van een familielid of een bekende vanwege uw lichamelijke of psychische problemen?' is equal to 'Ja' answer this question:***<br>Wat voor hulp van familieleden of bekenden heeft u gehad in de afgelopen 3 maanden?	☐ Huishoudelijke hulp - voorbeeld: stofzuigen, bed opmaken, boodschappen doen, klaarmaken van eten en drinken, verzorgen van kinderen<br>☐ Verzorging van uzelf - voorbeeld: hulp bij douchen of aankleden, hulp bij het eten en drinken of het geven van medicijnen<br>☐ Praktische hulp - voorbeeld: ondersteuning bij wandelen, het maken van uitstapjes of bezoekjes aan bekenden, bezoeken aan de huisarts of het ziekenhuis, het regelen van hulp of het regelen van financiële zaken

Paget LDA, *et al. BMJ Open* 2019; 9:e030961. doi: 10.1136/bmjopen-2019-030961

2.19.1.1	***If 'Wat voor hulp van familieleden of bekenden heeft u gehad in de afgelopen 3 maanden?' is equal to 'Huishoudelijke hulp - voorbeeld: stofzuigen, bed opmaken, boodschappen doen, klaarmaken van eten en drinken, verzorgen van kinderen' answer this question:*** Hoeveel weken heeft u deze hulp gehad? Huishoudelijke hulp:	______ weken in de afgelopen 3 maanden
2.19.1.2	***If 'Wat voor hulp van familieleden of bekenden heeft u gehad in de afgelopen 3 maanden?' is equal to 'Verzorging van uzelf - voorbeeld: hulp bij douchen of aankleden, hulp bij het eten en drinken of het geven van medicijnen' answer this question:*** Hoeveel weken heeft u deze hulp gehad? Verzorging van uzelf:	______ weken in de afgelopen 3 maanden
2.19.1.3	***If 'Wat voor hulp van familieleden of bekenden heeft u gehad in de afgelopen 3 maanden?' is equal to 'Praktische hulp - voorbeeld: ondersteuning bij wandelen, het maken van uitstapjes of bezoekjes aan bekenden, bezoeken aan de huisarts of het ziekenhuis, het regelen van hulp of het regelen van financiële zaken' answer this question:*** Hoeveel uur hulp kreeg u in deze weken gemiddeld? Praktische hulp:	______ weken in de afgelopen 3 maanden
2.19.1.4	***If 'Wat voor hulp van familieleden of bekenden heeft u gehad in de afgelopen 3 maanden?' is equal to 'Huishoudelijke hulp - voorbeeld: stofzuigen, bed opmaken, boodschappen doen, klaarmaken van eten en drinken, verzorgen van kinderen' answer this question:*** Hoeveel uur hulp kreeg u in deze weken gemiddeld? Huishoudelijke hulp: gemiddeld	______ uur in de week
2.19.1.5	***If 'Wat voor hulp van familieleden of bekenden heeft u gehad in de afgelopen 3 maanden?' is equal to 'Verzorging van uzelf - voorbeeld: hulp bij douchen of aankleden, hulp bij het eten en drinken of het geven van medicijnen' answer this question:*** Hoeveel uur hulp kreeg u in deze weken gemiddeld? Verzorging van uzelf: gemiddeld	______ uur in de week
2.19.1.6	***If 'Wat voor hulp van familieleden of bekenden heeft u gehad in de afgelopen 3 maanden?' is equal to 'Praktische hulp - voorbeeld: ondersteuning bij wandelen, het maken van uitstapjes of bezoekjes aan bekenden, bezoeken aan de huisarts of het ziekenhuis, het regelen van hulp of het regelen van financiële zaken' answer this question:*** Hoeveel uur hulp kreeg u in deze weken gemiddeld? Praktische hulp: gemiddeld	______ uur in de week

Paget LDA, *et al. BMJ Open* 2019; 9:e030961. doi: 10.1136/bmjopen-2019-030961

Vragenlijst PRIMA studie 39 weken - Dank

Number	Question	Answers
3.1	Hartelijk dank voor de tijd die u heeft genomen om de vragenlijsten in te vullen. Indien u nog opmerkingen heeft kunt u deze hierin plaatsen.	

Survey 'Vragenlijst PRIMA studie 5 jaar'

Vragenlijst PRIMA studie 5 jaar - AOFAS - Pijn

Number	Question	Answers
1.1	Hoeveel pijn ervaart u aan uw voet?	☐ Geen<br>☐ Milde pijnklachten of af en toe pijn<br>☐ Matige pijnklachten, dagelijks<br>☐ Vrijwel continu erge pijn

Vragenlijst PRIMA studie 5 jaar - AOFAS resterende gedeelte

Number	Question	Answers
2.1	Hoeveel beperkingen in het dagelijks leven ervaart u?	○ Geen beperkingen, geen hulpmiddelen nodig<br>○ Geen beperkingen in algemeen dagelijkse bezigheden, wel beperkingen in recreatieve activiteiten (sport, hobbies, etc) geen ondersteuning<br>○ Beperkingen bij dagelijkse- en recreatieve activiteiten, stok<br>○ Ernstige beperkingen bij dagelijkse- en recreatieactiviteiten, walker, krukken, rolstoel, brace

Paget LDA, *et al*. *BMJ Open* 2019; 9:e030961. doi: 10.1136/bmjopen-2019-030961

2.2	Wat is de afstand die u in één keer kunt lopen?	◯ Meer dan 600 meter<br>◯ 400 - 600 meter<br>◯ 100 - 300 meter<br>◯ Minder dan 100 meter
2.3	Heeft u moeite met lopen op een bepaalde ondergrond?	◯ Geen problemen bij lopen<br>◯ Enig problemen op ongelijke ondergrond, trap, helling, ladders<br>◯ Ernstige problemen bij ongelijke ondergrond, trap, helling, ladders
2.4	Hoe zou u uw looppatroon beoordelen?	◯ Normaal of minimaal afwijkend<br>◯ Duidelijk<br>◯ Ernstig
2.5	Hoe zou u de beweeglijkheid van uw voet beoordelen ten opzicht van de andere zijde of ten opzicte van vóór het ongeval?	◯ Hetzelfde of licht beperkt t.o.v. de niet aangedane zijde of zoals voor het ongeval<br>◯ Ongeveer de helft beperkt<br>◯ Sterk verminderd, bijna geen bewegingen mogelijk
2.6	Hoe zou u de beweeglijkheid van uw enkel beoordelen ten opzichte van de andere zijde of ten opzichte van voor het ongeval?	◯ Hetzelfde of licht beperkt t.o.v. de niet aangedane zijde of zoals voor het ongeval<br>◯ Ongeveer de helft beperkt<br>◯ Sterk verminderd, bijna geen bewegingen mogelijk
2.7	Hoe zou u de stabiliteit van uw voet en enkel beoordelen	◯ Stabiel, ik verzwik mijn enkel hooguit incidenteel<br>◯ Niet stabiel, ik verzwik mijn enkel vaak
2.8	Hoe zou u de stand van uw voet beoordelen ten opzichte van de andere zijde of ten opzichte van voor het ongeval?	◯ Goed, ik kan mijn voet goed plat op de grond zetten, waarbij de stand niet afwijkend is<br>◯ Ik sta vrijwel alleen op de buitenzijde van mijn voet<br>◯ Ik kan mijn voet niet plat op de grond zetten en loop vrijwel alleen op mijn voorvoet

Vragenlijst PRIMA studie 5 jaar - VAS Pain score

Paget LDA, *et al. BMJ Open* 2019; 9:e030961. doi: 10.1136/bmjopen-2019-030961

Number	Question	Answers	
3.1	VAS pijnscore (Geef uw minimaal pijnscore aan tijdens dagelijkse activiteiten op een schaal van 0 tot 100, waarbij 0 geen pijn is en 100 de ergste pijn denkbaar is)	(0.00)	100 (100.00)

Vragenlijst PRIMA studie 5 jaar - AAS

Number	Question	Answers

4.1	Wat is het meest bij u van toepassing ten aanzien van uw acitviteit?	☐ American Football<br>☐ Basketbal<br>☐ Gymnastiek/turnen<br>☐ Handbal<br>☐ Rugby<br>☐ Voetbal<br>☐ Hockey<br>☐ Korfbal<br>☐ Gevechtssporten: judo, karate, kung fu, taekwando, jiu jitsu, etc.<br>☐ Oriëntatielopen<br>☐ Rhytmische gymnastiek<br>☐ Volleybal<br>☐ Boxen<br>☐ Freestyle snowboarden<br>☐ Ijshockey<br>☐ Tennis<br>☐ Worstelen<br>☐ Fitness, aerobics<br>☐ Badminton<br>☐ Baseball<br>☐ Cross-country hardlopen<br>☐ Moderne pentathlon<br>☐ Squash<br>☐ Surfen, windsurfen<br>☐ Tafel tennis<br>☐ Atletiek: spring-, werponderdelen<br>☐ Waterskieën<br>☐ Dans<br>☐ Schermen<br>☐ Zaalhockey<br>☐ Bergbeklimmen<br>☐ Langlauf<br>☐ Parachute springen<br>☐ Softball<br>☐ Speciale beroepen en werkactiviteiten; speciale beroepen: ballet, professioneel soldaat, speciale reddingswerker, stuntman, etc.<br>☐ Duiken<br>☐ Scubaduiken<br>☐ Skaten, in-line skaten<br>☐ Atletiek: looponderdelen<br>☐ Triatlon<br>☐ Gewichtsheffen, body-building

Paget LDA, *et al*. *BMJ Open* 2019; 9:e030961. doi: 10.1136/bmjopen-2019-030961

☐ Alle competitieve sporten hieronder met 'seasonal' conditioning
☐ Zwaar fysiek werk
☐ Alpine skiën en snowboarden
☐ Bowlen/curlen
☐ Golf
☐ Mountainbike/BMX
☐ Powerliften
☐ Zeilen
☐ Fysiek werk
☐ Wielrennen
☐ Paardrijden
☐ Motorsporten/technische sporten
☐ Roeien, kayakken
☐ Boogschieten
☐ Water polo en zwemmen
☐ Kunnen lopen op oneven grond
☐ Geen sport, echter geen limitatie in dagelijkse activiteiten
☐ Kunnen lopen op even grond, maar de dagelijkste activiteiten zijn gelimiteerd
☐ Niet kunnen lopen, fysieke handicap wegens enkelproblemen

4.1.1	***If 'Wat is het meest bij u van toepassing ten aanzien van uw acitviteit?' is equal to 'American Football' answer this question:***<br>American Football	○ Professioneel<br>○ Competitief<br>○ Recreatief
4.1.2	***If 'Wat is het meest bij u van toepassing ten aanzien van uw acitviteit?' is equal to 'Basketbal' answer this question:***<br>Basketbal	○ Professioneel<br>○ Competitief<br>○ Recreatief
4.1.3	***If 'Wat is het meest bij u van toepassing ten aanzien van uw acitviteit?' is equal to 'Gymnastiek/turnen' answer this question:***<br>Gymnastiek/turnen	○ Professioneel<br>○ Competitief<br>○ Recreatief
4.1.4	***If 'Wat is het meest bij u van toepassing ten aanzien van uw acitviteit?' is equal to 'Handbal' answer this question:***<br>Handbal	○ Professioneel<br>○ Competitief<br>○ Recreatief
4.1.5	***If 'Wat is het meest bij u van toepassing ten aanzien van uw acitviteit?' is equal to 'Rugby' answer this question:***<br>Rugby	○ Professioneel<br>○ Competitief<br>○ Recreatief

Paget LDA, *et al*. *BMJ Open* 2019; 9:e030961. doi: 10.1136/bmjopen-2019-030961

4.1.6	***If 'Wat is het meest bij u van toepassing ten aanzien van uw acitviteit?' is equal to 'Voetbal' answer this question:***<br>Voetbal	◯ Professioneel<br>◯ Competitief<br>◯ Recreatief
4.1.7	***If 'Wat is het meest bij u van toepassing ten aanzien van uw acitviteit?' is equal to 'Hockey' answer this question:***<br>Hockey	◯ Professioneel<br>◯ Competitief<br>◯ Recreatief
4.1.8	***If 'Wat is het meest bij u van toepassing ten aanzien van uw acitviteit?' is equal to 'Korfbal' answer this question:***<br>Korfbal	◯ Professioneel<br>◯ Competitief<br>◯ Recreatief
4.1.9	***If 'Wat is het meest bij u van toepassing ten aanzien van uw acitviteit?' is equal to 'Gevechtssporten: judo, karate, kung fu, taekwando, jiu jitsu, etc.' answer this question:***<br>Gevechtssporten: judo, karate, kung fu, taekwando, jiu jitsu, etc.	◯ Professioneel<br>◯ Competitief<br>◯ Recreatief
4.1.10	***If 'Wat is het meest bij u van toepassing ten aanzien van uw acitviteit?' is equal to 'Oriëntatielopen' answer this question:***<br>Oriëntatielopen	◯ Professioneel<br>◯ Competitief<br>◯ Recreatief
4.1.11	***If 'Wat is het meest bij u van toepassing ten aanzien van uw acitviteit?' is equal to 'Rhytmische gymnastiek' answer this question:***<br>Rhytmisch gymnastiek	◯ Professioneel<br>◯ Competitief<br>◯ Recreatief
4.1.12	***If 'Wat is het meest bij u van toepassing ten aanzien van uw acitviteit?' is equal to 'Volleybal' answer this question:***<br>Volleybal	◯ Professioneel<br>◯ Competitief<br>◯ Recreatief
4.1.13	***If 'Wat is het meest bij u van toepassing ten aanzien van uw acitviteit?' is equal to 'Boxen' answer this question:***<br>Boxen	◯ Professioneel<br>◯ Competitief<br>◯ Recreatief
4.1.14	***If 'Wat is het meest bij u van toepassing ten aanzien van uw acitviteit?' is equal to 'Freestyle snowboarden' answer this question:***<br>Freestyle snowboarden	◯ Professioneel<br>◯ Competitief<br>◯ Recreatief
4.1.15	***If 'Wat is het meest bij u van toepassing ten aanzien van uw acitviteit?' is equal to 'Freestyle snowboarden' answer this question:***<br>IJshockey	◯ Professioneel<br>◯ Competitief<br>◯ Recreatief

Paget LDA, *et al. BMJ Open* 2019; 9:e030961. doi: 10.1136/bmjopen-2019-030961

4.1.16	***If 'Wat is het meest bij u van toepassing ten aanzien van uw acitviteit?' is equal to 'Tennis' answer this question:***<br>Tennis	◯ Professioneel<br>◯ Competitief<br>◯ Recreatief
4.1.17	***If 'Wat is het meest bij u van toepassing ten aanzien van uw acitviteit?' is equal to 'Worstelen' answer this question:***<br>Worstelen	◯ Professioneel<br>◯ Competitief<br>◯ Recreatief
4.1.18	***If 'Wat is het meest bij u van toepassing ten aanzien van uw acitviteit?' is equal to 'Fitness, aerobics' answer this question:***<br>Fitness, aerobics	◯ Professioneel<br>◯ Competitief<br>◯ Recreatief
4.1.19	***If 'Wat is het meest bij u van toepassing ten aanzien van uw acitviteit?' is equal to 'Badminton' answer this question:***<br>Badminton	◯ Professioneel<br>◯ Competitief<br>◯ Recreatief
4.1.20	***If 'Wat is het meest bij u van toepassing ten aanzien van uw acitviteit?' is equal to 'Baseball' answer this question:***<br>Baseball	◯ Professioneel<br>◯ Competitief<br>◯ Recreatief
4.1.21	***If 'Wat is het meest bij u van toepassing ten aanzien van uw acitviteit?' is equal to 'Cross-country hardlopen' answer this question:***<br>Cross-country hardlopen	◯ Professioneel<br>◯ Competitief<br>◯ Recreatief
4.1.22	***If 'Wat is het meest bij u van toepassing ten aanzien van uw acitviteit?' is equal to 'Moderne pentathlon' answer this question:***<br>Moderne pentathlon	◯ Professioneel<br>◯ Competitief<br>◯ Recreatief
4.1.23	***If 'Wat is het meest bij u van toepassing ten aanzien van uw acitviteit?' is equal to 'Squash' answer this question:***<br>Squash	◯ Professioneel<br>◯ Competitief<br>◯ Recreatief
4.1.24	***If 'Wat is het meest bij u van toepassing ten aanzien van uw acitviteit?' is equal to 'Surfen, windsurfen' answer this question:***<br>Surfen, windsurfen	◯ Professioneel<br>◯ Competitief<br>◯ Recreatief
4.1.25	***If 'Wat is het meest bij u van toepassing ten aanzien van uw acitviteit?' is equal to 'Tafel tennis' answer this question:***<br>Tafel tennis	◯ Professioneel<br>◯ Competitief<br>◯ Recreatief

Paget LDA, *et al. BMJ Open* 2019; 9:e030961. doi: 10.1136/bmjopen-2019-030961

4.1.26	***If 'Wat is het meest bij u van toepassing ten aanzien van uw acitviteit?' is equal to 'Atletiek: spring-, werponderdelen' answer this question:***<br>Atletiek: spring-, werponderdelen	◯ Professioneel<br>◯ Competitief<br>◯ Recreatief
4.1.27	***If 'Wat is het meest bij u van toepassing ten aanzien van uw acitviteit?' is equal to 'Waterskieën' answer this question:***<br>Waterskien	◯ Professioneel<br>◯ Competitief<br>◯ Recreatief
4.1.28	***If 'Wat is het meest bij u van toepassing ten aanzien van uw acitviteit?' is equal to 'Dans' answer this question:***<br>Dans	◯ Professioneel<br>◯ Competitief<br>◯ Recreatief
4.1.29	***If 'Wat is het meest bij u van toepassing ten aanzien van uw acitviteit?' is equal to 'Schermen' answer this question:***<br>Schermen	◯ Professioneel<br>◯ Competitief<br>◯ Recreatief
4.1.30	***If 'Wat is het meest bij u van toepassing ten aanzien van uw acitviteit?' is equal to 'Zaalhockey' answer this question:***<br>Zaalhockey	◯ Professioneel<br>◯ Competitief<br>◯ Recreatief
4.1.31	***If 'Wat is het meest bij u van toepassing ten aanzien van uw acitviteit?' is equal to 'Bergbeklimmen' answer this question:***<br>Bergbeklimmen	◯ Professioneel<br>◯ Competitief<br>◯ Recreatief
4.1.32	***If 'Wat is het meest bij u van toepassing ten aanzien van uw acitviteit?' is equal to 'Langlauf' answer this question:***<br>Langlauf	◯ Professioneel<br>◯ Competitief<br>◯ Recreatief
4.1.33	***If 'Wat is het meest bij u van toepassing ten aanzien van uw acitviteit?' is equal to 'Parachute springen' answer this question:***<br>Parachute springen	◯ Professioneel<br>◯ Competitief<br>◯ Recreatief
4.1.34	***If 'Wat is het meest bij u van toepassing ten aanzien van uw acitviteit?' is equal to 'Softball' answer this question:***<br>Softball	◯ Professioneel<br>◯ Competitief<br>◯ Recreatief

Paget LDA, *et al*. *BMJ Open* 2019; 9:e030961. doi: 10.1136/bmjopen-2019-030961

4.1.35	***If 'Wat is het meest bij u van toepassing ten aanzien van uw acitviteit?' is equal to 'Speciale beroepen en werkactiviteiten; speciale beroepen: ballet, professioneel soldaat, speciale reddingswerker, stuntman, etc.' answer this question:***<br>Speciale beroepen en werkactiviteiten speciale beroepen: ballet, professioneel soldaat, speciale reddingswerker, stuntman, etc.	○ ballet, professioneel soldaat, speciale reddingswerker, stuntman, etc.
4.1.36	***If 'Wat is het meest bij u van toepassing ten aanzien van uw acitviteit?' is equal to 'Duiken' answer this question:***<br>Duiken	○ Professioneel/competitief<br>○ Recreatief
4.1.37	***If 'Wat is het meest bij u van toepassing ten aanzien van uw acitviteit?' is equal to 'Scubaduiken' answer this question:***<br>Scubaduiken	○ Professioneel/competitief<br>○ Recreatief
4.1.38	***If 'Wat is het meest bij u van toepassing ten aanzien van uw acitviteit?' is equal to 'Skaten, in-line skaten' answer this question:***<br>skaten, in-linen skaten	○ Professioneel/competitief<br>○ Recreatief
4.1.39	***If 'Wat is het meest bij u van toepassing ten aanzien van uw acitviteit?' is equal to 'Atletiek: looponderdelen' answer this question:***<br>Atletiek: looponderdelen	○ Professioneel/competitief<br>○ Recreatief
4.1.40	***If 'Wat is het meest bij u van toepassing ten aanzien van uw acitviteit?' is equal to 'Triatlon' answer this question:***<br>triatlon	○ Professioneel/competitief<br>○ Recreatief
4.1.41	***If 'Wat is het meest bij u van toepassing ten aanzien van uw acitviteit?' is equal to 'Gewichtsheffen, body-building' answer this question:***<br>Gewichtsheffen, body-building	○ Professioneel/competitief<br>○ Recreatief
4.1.42	***If 'Wat is het meest bij u van toepassing ten aanzien van uw acitviteit?' is equal to 'Alle competitieve sporten hieronder met ‘seasonal’ conditioning' answer this question:***<br>Alle competitieve sporten hieronder met ‘seasonal’ conditioning	○ Alle competitieve sporten hieronder met ‘seasonal’ conditioning
4.1.43	***If 'Wat is het meest bij u van toepassing ten aanzien van uw acitviteit?' is equal to 'Zwaar fysiek werk' answer this question:***<br>Zwaar fysiek werk	○ Alle competitieve sporten hieronder met ‘seasonal’ conditioning

Paget LDA, *et al. BMJ Open* 2019; 9:e030961. doi: 10.1136/bmjopen-2019-030961

4.1.44	***If 'Wat is het meest bij u van toepassing ten aanzien van uw acitviteit?' is equal to 'Alpine skiën en snowboarden' answer this question:***<br>Alpine skien en snowboarden	◯ Alpine skien en snowboarden
4.1.45	***If 'Wat is het meest bij u van toepassing ten aanzien van uw acitviteit?' is equal to 'Bowlen/curlen' answer this question:***<br>Bowlen/curlen	◯ Bowlen/curlen
4.1.46	***If 'Wat is het meest bij u van toepassing ten aanzien van uw acitviteit?' is equal to 'Golf' answer this question:***<br>Golf	◯ Golf
4.1.47	***If 'Wat is het meest bij u van toepassing ten aanzien van uw acitviteit?' is equal to 'Mountainbike/BMX' answer this question:***<br>Mountainbike/BMX	◯ Mountainbike/BMX
4.1.48	***If 'Wat is het meest bij u van toepassing ten aanzien van uw acitviteit?' is equal to 'Powerliften' answer this question:***<br>Powerliften	◯ Powerliften
4.1.49	***If 'Wat is het meest bij u van toepassing ten aanzien van uw acitviteit?' is equal to 'Zeilen' answer this question:***<br>Zeilen	◯ Zeilen
4.1.50	***If 'Wat is het meest bij u van toepassing ten aanzien van uw acitviteit?' is equal to 'Fysiek werk' answer this question:***<br>Fysiek werk	◯ Fysiek werk
4.1.51	***If 'Wat is het meest bij u van toepassing ten aanzien van uw acitviteit?' is equal to 'Wielrennen' answer this question:***<br>Wielrennen	◯ Wielrennen
4.1.52	***If 'Wat is het meest bij u van toepassing ten aanzien van uw acitviteit?' is equal to 'Paardrijden' answer this question:***<br>Paardrijden	◯ Paardrijden
4.1.53	***If 'Wat is het meest bij u van toepassing ten aanzien van uw acitviteit?' is equal to 'Motorsporten/technische sporten' answer this question:***<br>Motorsporten/technische sporten	◯ Motorsporten/technische sporten
4.1.54	***If 'Wat is het meest bij u van toepassing ten aanzien van uw acitviteit?' is equal to 'Roeien, kayakken' answer this question:***<br>Roeien, kayakken	◯ Roeien, kayakken

Paget LDA, *et al. BMJ Open* 2019; 9:e030961. doi: 10.1136/bmjopen-2019-030961

4.1.55	***If 'Wat is het meest bij u van toepassing ten aanzien van uw acitviteit?' is equal to 'Boogschieten' answer this question:*** Boogschieten	◯ Boogschieten
4.1.56	***If 'Wat is het meest bij u van toepassing ten aanzien van uw acitviteit?' is equal to 'Water polo en zwemmen' answer this question:*** Water polo en zwemmen	◯ Water polo en zwemmen
4.1.57	***If 'Wat is het meest bij u van toepassing ten aanzien van uw acitviteit?' is equal to 'Kunnen lopen op oneven grond' answer this question:*** Kunnen lopen op oneven grond	◯ Kunnen lopen op oneven grond
4.1.58	***If 'Wat is het meest bij u van toepassing ten aanzien van uw acitviteit?' is equal to 'Geen sport, echter geen limitatie in dagelijkse activiteiten' answer this question:*** Geen sport, echter geen limitatie in dagelijkse activiteiten	◯ Geen sport, echter geen limitatie in dagelijkse activiteiten
4.1.59	***If 'Wat is het meest bij u van toepassing ten aanzien van uw acitviteit?' is equal to 'Kunnen lopen op even grond, maar de dagelijkste activiteiten zijn gelimiteerd' answer this question:*** Kunnen lopen op even grond, maar de dagelijkste activiteiten zijn gelimiteerd	◯ Kunnen lopen op even grond, maar de dagelijkste activiteiten zijn gelimiteerd
4.1.60	***If 'Wat is het meest bij u van toepassing ten aanzien van uw acitviteit?' is equal to 'Niet kunnen lopen, fysieke handicap wegens enkelproblemen' answer this question:*** Niet kunnen lopen, fysieke handicap wegens enkelproblemen	◯ Niet kunnen lopen, fysieke handicap wegens enkelproblemen

Vragenlijst PRIMA studie 5 jaar - Hoe tevreden bent u over de enkelklachten?

Number	Question	Answers
5.1	Hoe tevreden bent u over de enkelklachten?	◯ Slecht<br>◯ Matig<br>◯ Goed<br>◯ Uitstekend

Paget LDA, *et al. BMJ Open* 2019; 9:e030961. doi: 10.1136/bmjopen-2019-030961

Vragenlijst PRIMA studie 5 jaar - SF-36

Number	Question	Answers
	Deze vragenlijst gaat over uw standpunten t.a.v. uw gezondheid. Met behulp van deze gegevens kan worden bijgehouden hoe u zich voelt en hoe goed u in staat bent uw gebruikelijke bezigheden uit te voeren. Beantwoord elke vraag door een antwoord aan te klikken. Als u niet zeker weet hoe u een vraag moet beantwoorden, geef dan het best mogelijke antwoord.	
6.1	Hoe zou u over het algemeen uw gezondheid noemen?	◯ Uitstekend ◯ Zeer goed ◯ Goed ◯ Matig ◯ Slecht
6.2	Hoe beoordeelt u nu uw gezondheid over het algemeen vergeleken met een jaar geleden?	◯ Veel beter dan een jaar geleden ◯ Wat beter dan een jaar geleden ◯ Ongeveer hetzelfde als een jaar geleden ◯ Wat slechter dan een jaar geleden ◯ Veel slechter dan een jaar geleden
6.3	Wordt u door uw gezondheid op dit moment beperkt bij forse inspanning, zoals hardlopen, tillen van zware voorwerpen of een veeleisende sport beoefenen?	◯ Ja, ernstig beperkt ◯ Ja, een beetje beperkt ◯ Nee, helemaal niet beperkt
6.4	Wordt u door uw gezondheid op dit moment beperkt bij matige inspanning zoals een tafel verplaatsen, stofzuigen, zwemmen of fietsen?	◯ Ja, ernstig beperkt ◯ Ja, een beetje beperkt ◯ Nee, helemaal niet beperkt
6.5	Wordt u door uw gezondheid op dit moment beperkt bij boodschappen tillen of dragen?	◯ Ja, ernstig beperkt ◯ Ja, een beetje beperkt ◯ Nee, helemaal niet beperkt
6.6	Wordt u door uw gezondheid op dit moment beperkt bij een paar trappen oplopen?	◯ Ja, ernstig beperkt ◯ Ja, een beetje beperkt ◯ Nee, helemaal niet beperkt
6.7	Wordt u door uw gezondheid op dit moment beperkt bij één trap oplopen?	◯ Ja, ernstig beperkt ◯ Ja, een beetje beperkt ◯ Nee, helemaal niet beperkt
6.8	Wordt u door uw gezondheid op dit moment beperkt bij bukken, knielen of hurken?	◯ Ja, ernstig beperkt ◯ Ja, een beetje beperkt ◯ Nee, helemaal niet beperkt
6.9	Wordt u door uw gezondheid op dit moment beperkt bij meer dan één kilometer lopen?	◯ Ja, ernstig beperkt ◯ Ja, een beetje beperkt ◯ Nee, helemaal niet beperkt
6.10	Wordt u door uw gezondheid op dit moment beperkt bij een paar honderd meter lopen?	◯ Ja, ernstig beperkt ◯ Ja, een beetje beperkt ◯ Nee, helemaal niet beperkt

Paget LDA, *et al. BMJ Open* 2019; 9:e030961. doi: 10.1136/bmjopen-2019-030961

6.11	Wordt u door uw gezondheid op dit moment beperkt bij ongeveer honderd meter lopen?	◯ Ja, ernstig beperkt ◯ Ja, een beetje beperkt ◯ Nee, helemaal niet beperkt
6.12	Wordt u door uw gezondheid op dit moment beperkt bij uzelf wassen of aankleden?	◯ Ja, ernstig beperkt ◯ Ja, een beetje beperkt ◯ Nee, helemaal niet beperkt
6.13	U besteedde in de afgelopen 4 weken minder tijd aan werk of andere bezigheden	◯ Ja ◯ Nee
6.14	U heeft in de afgelopen 4 weken minder bereikt dan u zou willen	◯ Ja ◯ Nee
6.15	U was in de afgelopen 4 weken beperkt in het soort werk of andere bezigheden.	◯ Ja ◯ Nee
6.16	U had de afgelopen 4 weken moeite om uw werk of andere bezigheden uit te voeren (het kostte u bijvoorbeeld extra inspanning).	◯ Ja ◯ Nee
6.17	U besteedde in de afgelopen 4 weken minder tijd aan werk of andere bezigheden ten gevolge van emotionele problemen	◯ Ja ◯ Nee
6.18	U heeft in de afgelopen 4 weken minder bereikt dan u zou willen ten gevolge van emotionele problemen.	◯ Ja ◯ Nee
6.19	U deed de afgelopen 4 weken uw werk of andere bezigheden niet zo zorgvuldig als gewoonlijk ten gevolge van emotionele problemen.	◯ Ja ◯ Nee
6.20	In hoeverre hebben uw lichamelijke gezondheid of emotionele problemen u gedurende de afgelopen 4 weken gehinderd in uw normale omgang met familie, vrienden of buren, of bij activiteiten in groepsverband?	◯ Helemaal niet ◯ Enigszins ◯ Nogal ◯ Veel ◯ Heel erg veel
6.21	Hoeveel lichamelijke pijn heeft u de afgelopen 4 weken gehad?	◯ Geen<br>◯ Heel licht<br>◯ Licht<br>◯ Nogal<br>◯ Ernstig<br>◯ Heel ernstig
6.22	In welke mate bent u de afgelopen 4 weken door de pijn gehinderd in uw normale werk (zowel werk buitenshuis als huishoudelijk werk)?	◯ Helemaal niet<br>◯ Een klein beetje<br>◯ Nogal<br>◯ Veel<br>◯ Heel erg veel

Paget LDA, *et al. BMJ Open* 2019; 9:e030961. doi: 10.1136/bmjopen-2019-030961

6.23	Voelde u zich levenslustig?	◯ Altijd<br>◯ Meestal<br>◯ Vaak<br>◯ Soms<br>◯ Zelden<br>◯ Nooit
6.24	Was u erg zenuwachtig?	◯ Altijd<br>◯ Meestal<br>◯ Vaak<br>◯ Soms<br>◯ Zelden<br>◯ Nooit
6.25	Zat u zo in de put dat u niets kon?	◯ Altijd<br>◯ Meestal<br>◯ Vaak<br>◯ Soms<br>◯ Zelden<br>◯ Nooit
6.26	Voelde u zich rustig en tevreden?	◯ Altijd<br>◯ Meestal<br>◯ Vaak<br>◯ Soms<br>◯ Zelden<br>◯ Nooit
6.27	Had u veel energie?	◯ Altijd<br>◯ Meestal<br>◯ Vaak<br>◯ Soms<br>◯ Zelden<br>◯ Nooit
6.28	Voelde u zich somber en neerslachtig?	◯ Altijd<br>◯ Meestal<br>◯ Vaak<br>◯ Soms<br>◯ Zelden<br>◯ Nooit

Paget LDA, *et al. BMJ Open* 2019; 9:e030961. doi: 10.1136/bmjopen-2019-030961

6.29	Voelde u zich uitgeput?	◯ Altijd<br>◯ Meestal<br>◯ Vaak<br>◯ Soms<br>◯ Zelden<br>◯ Nooit
6.30	Was u een gelukkig mens?	◯ Altijd<br>◯ Meestal<br>◯ Vaak<br>◯ Soms<br>◯ Zelden<br>◯ Nooit
6.31	Voelde u zich moe?	◯ Altijd<br>◯ Meestal<br>◯ Vaak<br>◯ Soms<br>◯ Zelden<br>◯ Nooit
6.32	Hoe vaak hebben uw lichamelijke gezondheid of emotionele problemen u gedurende de afgelopen 4 weken gehinderd bij uw sociale activiteiten (zoals vrienden of familie bezoeken etc.)?	◯ Altijd<br>◯ Meestal<br>◯ Soms<br>◯ Zelden<br>◯ Nooit
6.33	Ik lijk wat gemakkelijker ziek te worden dan andere mensen	◯ Volkomen juist<br>◯ Grotendeels juist<br>◯ Weet ik niet<br>◯ Grotendeels onjuist<br>◯ Volkomen onjuist
6.34	Ik ben even gezond als andere mensen die ik ken	◯ Volkomen juist<br>◯ Grotendeels juist<br>◯ Weet ik niet<br>◯ Grotendeels onjuist<br>◯ Volkomen onjuist

Paget LDA, et al. BMJ Open 2019; 9:e030961. doi: 10.1136/bmjopen-2019-030961

6.35	Ik verwacht dat mijn gezondheid achteruit zal gaan	◯ Volkomen juist<br>◯ Grotendeels juist<br>◯ Weet ik niet<br>◯ Grotendeels onjuist<br>◯ Volkomen onjuist
6.36	Mijn gezondheid is uitstekend	◯ Volkomen juist<br>◯ Grotendeels juist<br>◯ Weet ik niet<br>◯ Grotendeels onjuist<br>◯ Volkomen onjuist

Vragenlijst PRIMA studie 5 jaar - GAS

Number	Question	Answers
	Tijdens de eerste afspraak werd een duidelijk doel met u afgesproken. Dit werd tevens per email naar u gestuurd. Hoe is de situatie nu ten aanzien van de destijds gestelde doelstelling?	
7.1	Wat is nu de situatie ten aanzien van het tijdens de eerste afspraak afgesproken doelstelling (Goal attainment Scaling)?	◯ Achteruitgang (minder dan de utigangssituatie)<br>◯ Uitgangssituatie<br>◯ Minder dan het doel<br>◯ Doel<br>◯ Meer dan het doel<br>◯ Veel meer dan het doel

Vragenlijst PRIMA studie 5 jaar - EQ-5D-3L

Number	Question	Answers
	Deze vragenlijst gaat over uw standpunten t.a.v. uw gezondheid. Met behulp van deze gegevens kan worden bijgehouden hoe u zich voelt en hoe goed u in staat bent uw gebruikelijke bezigheden uit te voeren. Beantwoord elke vraag door een antwoord aan te klikken. Als u niet zeker weet hoe u een vraag moet beantwoorden, geef dan het best mogelijke antwoord.	
8.1	Hoe is het met uw Mobiliteit gesteld?	◯ Ik heb geen problemen met lopen<br>◯ Ik heb enige problemen met lopen<br>◯ Ik ben bedlegerig

Paget LDA, *et al*. *BMJ Open* 2019; 9:e030961. doi: 10.1136/bmjopen-2019-030961

8.2	Hoe is het met uw Zelfzorg gesteld?	◯ Ik heb geen problemen om mijzelf te wassen of aan te kleden<br>◯ Ik heb enige problemen om mijzelf te wassen of aan te kleden<br>◯ Ik ben niet in staat om mijzelf te wassen of aan te kleden
8.3	Hoe is het met de Dagelijkse activiteiten (werk, studie, huishouden, gezins- en vrijetijdsactiviteiten) gesteld?	◯ Ik heb geen problemen met mijn dagelijkse activiteiten<br>◯ Ik heb enige problemen met mijn dagelijkse activiteiten<br>◯ Ik ben niet in staat om mijn dagelijkse activiteiten uit te voeren
8.4	Hoe is het met de Pijn/klachten gesteld?	◯ Ik heb geen pijn of andere klachten<br>◯ Ik heb matige pijn of andere klachten<br>◯ Ik heb zeer ernstige pijn of andere klachten
8.5	Hoe is het met de Stemming gesteld?	◯ Ik ben niet angstig of somber<br>◯ Ik ben matig angstig of somber<br>◯ Ik ben erg angstig of somber
	We willen weten hoe goed of slecht uw gezondheid VANDAAG is. Deze meetschaal loopt van 0 tot 100: 100 staat voor de BESTE gezondheid die u zich kunt voorstellen - 0 staat voor de SLECHTSTE gezondheid die u zich kunt voorstellen	
8.6	Uw Gezondheid vandaag	(0.00) (100.00)

Vragenlijst PRIMA studie 5 jaar - AOS

Number	Question	Answers
	Instructies: De lijn naast elke vraag staat voor hoeveel PIJN u heeft in verschillende situaties. De linker kant (0) is “geen pijn” en de rechter kant (100) is “ergste pijn denkbaar”. Geef voor de onderstaande situaties op de lijn aan hoeveel PIJN u in de afgelopen week in de enkel had. Als een of meerdere van deze situaties niet van toepassing waren, dan kiest u de "niet van toepassing" optie.	
9.1	Wat was de hoogte van de ergste pijn in de afgelopen week?	(0.00) (100.00)
9.2	Heeft u pijn als u 's ochtends opstaat?	◯ Ja<br>◯ Nee<br>◯ Niet van toepassing

Paget LDA, *et al. BMJ Open* 2019; 9:e030961. doi: 10.1136/bmjopen-2019-030961

9.2.1	***If 'Heeft u pijn als u 's ochtends opstaat?' is equal to 'Ja' answer this question:*** Hoeveel pijn heeft u voordat u 's ochtends opstaat?	(0.00)	(100.00)
9.3	Heeft u pijn wanneer u op blote voeten loopt?	◯ Ja<br>◯ Nee<br>◯ Niet van toepassing	
9.3.1	***If 'Heeft u pijn wanneer u op blote voeten loopt?' is equal to 'Ja' answer this question:*** Hoeveel pijn heeft u als u op blote voeten loopt?	(0.00)	(100.00)
9.4	Heeft u pijn wanneer u op blote voeten staat?	◯ Ja<br>◯ Nee<br>◯ Niet van toepassing	
9.4.1	***If 'Heeft u pijn wanneer u op blote voeten staat?' is equal to 'Ja' answer this question:*** Hoeveel pijn heeft u als u op blote voeten staat?	(0.00)	(100.00)
9.5	Heeft u pijn wanneer u met schoenen loopt?	◯ Ja<br>◯ Nee<br>◯ Niet van toepassing	
9.5.1	***If 'Heeft u pijn wanneer u met schoenen loopt?' is equal to 'Ja' answer this question:*** Hoeveel pijn heeft u wanneer u met schoenen loopt?	(0.00)	(100.00)
9.6	Heeft u pijn wanneer u met schoenen staat?	◯ Ja<br>◯ Nee<br>◯ Niet van toepassing	
9.6.1	***If 'Heeft u pijn wanneer u met schoenen staat?' is equal to 'Ja' answer this question:*** Hoeveel pijn heeft u wanneer u met schoenen staat?	(0.00)	(100.00)
9.7	Heeft u pijn wanneer u loopt met steunzolen of een brace?	◯ Ja<br>◯ Nee<br>◯ Niet van toepassing	
9.7.1	***If 'Heeft u pijn wanneer u loopt met steunzolen of een brace?' is equal to 'Ja' answer this question:*** Hoeveel pijn heeft u wanneer u loopt met steunzolen of een brace?	(0.00)	(100.00)

Paget LDA, *et al. BMJ Open* 2019; 9:e030961. doi: 10.1136/bmjopen-2019-030961

9.8	Heeft u pijn wanneer u staat met steunzolen of een brace?	◯ Ja<br>◯ Nee<br>◯ Niet van toepassing	
9.8.1	***If 'Heeft u pijn wanneer u staat met steunzolen of een brace?' is equal to 'Ja' answer this question:***<br>Hoeveel pijn heeft u wanneer u staat met steunzolen of een brace?	(0.00)	(100.00)
9.9	Heeft u pijn aan het einde van de dag?	◯ Ja<br>◯ Nee<br>◯ Niet van toepassing	
9.9.1	***If 'Heeft u pijn aan het einde van de dag?' is equal to 'Ja' answer this question:***<br>Hoeveel pijn heeft u aan het einde van de dag?	(0.00)	(100.00)
	Instructies:De lijn naast elke vraag staat voor hoeveel MOEITE u heeft met verschillende activiteiten. De linker kant (0) is “Niet moeilijk” en de rechter kant (100) is “Te moeilijk, niet uitvoerbaar”. Geef voor de onderstaande activiteiten op de lijn aan hoeveel MOEITE u in de afgelopen week door enkelklachten had met onderstaande activiteiten. Als een situatie niet van toepassing is, kies dan de optie "niet van toepassing".		
9.10	Heeft u moeite met door het huis lopen?	◯ Ja<br>◯ Nee<br>◯ Niet van toepassing	
9.10.1	***If 'Heeft u moeite met door het huis lopen?' is equal to 'Ja' answer this question:***<br>Hoeveel moeite heeft u met door het huis lopen?	(0.00)	(100.00)
9.11	Heeft u moeite met buiten lopen op oneven ondergrond?	◯ Ja<br>◯ Nee<br>◯ Niet van toepassing	
9.11.1	***If 'Heeft u moeite met buiten lopen op oneven ondergrond?' is equal to 'Ja' answer this question:***<br>Hoeveel moeite heeft u met buiten lopen op oneven ondergrond?	(0.00)	(100.00)
9.12	Heeft u moeite met een paar honderd meter lopen?	◯ Ja<br>◯ Nee<br>◯ Niet van toepassing	
9.12.1	***If 'Heeft u moeite met een paar honderd meter lopen?' is equal to 'Ja' answer this question:***<br>Hoeveel moeite heeft u met een paar honderd meter lopen?	(0.00)	(100.00)

Paget LDA, *et al*. *BMJ Open* 2019; 9:e030961. doi: 10.1136/bmjopen-2019-030961

9.13	Heeft u moeite met een trap oplopen?	◯ Ja<br>◯ Nee<br>◯ Niet van toepassing	
9.13.1	***If 'Heeft u moeite met een trap oplopen?' is equal to 'Ja' answer this question:***<br>Hoeveel moeite heeft u met een trap oplopen?	(0.00)	(100.00)
9.14	Heeft u moeite met een trap aflopen?	◯ Ja<br>◯ Nee<br>◯ Niet van toepassing	
9.14.1	***If 'Heeft u moeite met een trap aflopen?' is equal to 'Ja' answer this question:***<br>Hoeveel moeite heeft u met een trap aflopen?	(0.00)	(100.00)
9.15	Heeft u moeite met op de tenen staan?	◯ Ja<br>◯ Nee<br>◯ Niet van toepassing	
9.15.1	***If 'Heeft u moeite met op de tenen staan?' is equal to 'Ja' answer this question:***<br>Hoeveel moeite heeft u met op de tenen staan?	(0.00)	(100.00)
9.16	Heeft u moeite met opstaan uit de stoel?	◯ Ja<br>◯ Nee<br>◯ Niet van toepassing	
9.16.1	***If 'Heeft u moeite met opstaan uit de stoel?' is equal to 'Ja' answer this question:***<br>Hoeveel moeite heeft u met opstaan uit de stoel?	(0.00)	(100.00)
9.17	Heeft u moeite met het op- of afstappen van stoepranden?	◯ Ja<br>◯ Nee<br>◯ Niet van toepassing	
9.17.1	***If 'Heeft u moeite met het op- of afstappen van stoepranden?' is equal to 'Ja' answer this question:***<br>Hoeveel moeite heeft u met het op- of afstappen van stoepranden?	(0.00)	(100.00)
9.18	Heeft u moeite met snel lopen of rennen?	◯ Ja<br>◯ Nee<br>◯ Niet van toepassing	

Paget LDA, *et al. BMJ Open* 2019; 9:e030961. doi: 10.1136/bmjopen-2019-030961

9.18.1	***If 'Heeft u moeite met snel lopen of rennen?' is equal to 'Ja' answer this question:*** Hoeveel moeite heeft u met snel lopen of rennen?	(0.00)	(100.00)

Vragenlijst PRIMA studie 5 jaar - FAOS

Number	Question	Answers
	Deze lijst vraagt naar uw mening over uw voet/enkel. Uw antwoorden geven ons een beeld van uw voet/enkel klachten en hoe u in staat bent om alledaagse activiteiten uit te voeren in uw huidige situatie. Beantwoorden van een vraag doet u door het aanklikken van een vakje met het volgens u meest juiste antwoord (één vakje per vraag). Als u niet zeker weet hoe u een vraag moet beantwoorden, geeft u dan het antwoord dat volgens u het meest op uw situatie van toepassing is. Deze vraag heeft betrekking op het voorkomen van voet/enkel klachten in de afgelopen week.	
10.1	Is uw voet/enkel gezwollen?	◯ Nooit ◯ Zelden ◯ Soms ◯ Vaak ◯ Altijd
10.2	Voelt u gekraak of hoort u klikken of een ander vreemd geluid wanneer u de voet/enkel beweegt?	◯ Nooit ◯ Zelden ◯ Soms ◯ Vaak ◯ Altijd
10.3	Hapert uw enkel of blokkeert uw enkel ('op slot' gaan zitten) wanneer u deze beweegt?	◯ Nooit ◯ Zelden ◯ Soms ◯ Vaak ◯ Altijd
10.4	Kunt u de voet/enkel volledig strekken?	◯ Altijd ◯ Vaak ◯ Soms ◯ Zelden ◯ Nooit
10.5	Kunt u de voet volledig naar u toe buigen?	◯ Altijd ◯ Vaak ◯ Soms ◯ Zelden ◯ Nooit
10.6	In welke mate heeft u een stijf gevoel in de voet/enkel 's ochtends bij het wakker worden?	◯ Niet ◯ Mild ◯ Matig ◯ Ernstig ◯ Zeer ernstig
10.7	In welke mate heeft u een stijf gevoel in de voet/enkel na zitten, liggen of rusten later op de dag?	◯ Niet ◯ Mild ◯ Matig ◯ Ernstig ◯ Zeer ernstig
10.8	Hoe vaak heeft u pijn in uw voet/enkel?	◯ Nooit ◯ Maandelijks ◯ Wekelijks ◯ Dagelijks ◯ Altijd

Paget LDA, *et al. BMJ Open* 2019; 9:e030961. doi: 10.1136/bmjopen-2019-030961

10.9	Hoeveel voet/enkel pijn heeft u gehad in de afgelopen week bij draaien als uw voet/enkel op de grond staat?	◯ Geen<br>◯ Mild<br>◯ Matig<br>◯ Ernstig<br>◯ Zeer ernstig
10.10	Hoeveel voet/enkel pijn heeft u gehad in de afgelopen week bij het volledig uitstrekken van de voet/enkel?	◯ Geen<br>◯ Mild<br>◯ Matig<br>◯ Ernstig<br>◯ Zeer ernstig
10.11	Hoeveel voet/enkel pijn heeft u gehad in de afgelopen week bij het volledig naar u toe buigen/optrekken van de voet/enkel	◯ Geen<br>◯ Mild<br>◯ Matig<br>◯ Ernstig<br>◯ Zeer ernstig
10.12	Hoeveel voet/enkel pijn heeft u gehad in de afgelopen week bij het lopen op een vlakke ondergrond	◯ Geen<br>◯ Mild<br>◯ Matig<br>◯ Ernstig<br>◯ Zeer ernstig
10.13	Hoeveel voet/enkel pijn heeft u gehad in de afgelopen week bij het trap op- en trap af lopen?	◯ Geen<br>◯ Mild<br>◯ Matig<br>◯ Ernstig<br>◯ Zeer ernstig
10.14	Hoeveel voet/enkel pijn heeft u gehad in de afgelopen week 's nachts in bed?	◯ Geen<br>◯ Mild<br>◯ Matig<br>◯ Ernstig<br>◯ Zeer ernstig
10.15	Hoeveel voet/enkel pijn heeft u gehad in de afgelopen week bij het zitten of liggen?	◯ Geen<br>◯ Mild<br>◯ Matig<br>◯ Ernstig<br>◯ Zeer ernstig

Paget LDA, *et al. BMJ Open* 2019; 9:e030961. doi: 10.1136/bmjopen-2019-030961

10.16	Hoeveel voet/enkel pijn heeft u gehad in de afgelopen week bij het rechtop staan?	◯ Geen<br>◯ Mild<br>◯ Matig<br>◯ Ernstig<br>◯ Zeer ernstig
10.17	In welke mate werd u gehinderd bij het trap aflopen?	◯ Niet ◯ Mild ◯ Matig ◯ Ernstig ◯ Zeer ernstig
10.18	In welke mate werd u gehinderd bij het trap op lopen?	◯ Niet ◯ Mild ◯ Matig ◯ Ernstig ◯ Zeer ernstig
10.19	In welke mate werd u gehinderd als u vanuit een zittende positie ging staan?	◯ Niet ◯ Mild ◯ Matig ◯ Ernstig ◯ Zeer ernstig
10.20	In welke mate werd u gehinderd bij het staan?	◯ Niet ◯ Mild ◯ Matig ◯ Ernstig ◯ Zeer ernstig
10.21	In welke mate werd u gehinderd bij het naar de grond buigen/iets oprapen?	◯ Niet ◯ Mild ◯ Matig ◯ Ernstig ◯ Zeer ernstig
10.22	In welke mate werd u gehinderd bij het lopen op een vlakke ondergrond?	◯ Niet ◯ Mild ◯ Matig ◯ Ernstig ◯ Zeer ernstig
10.23	In welke mate werd u gehinderd bij het in- en uit de auto stappen?	◯ Niet ◯ Mild ◯ Matig ◯ Ernstig ◯ Zeer ernstig
10.24	In welke mate werd u gehinderd bij het boodschappen doen?	◯ Niet ◯ Mild ◯ Matig ◯ Ernstig ◯ Zeer ernstig
10.25	In welke mate werd u gehinderd bij sokken/panty's aantrekken?	◯ Niet ◯ Mild ◯ Matig ◯ Ernstig ◯ Zeer ernstig
10.26	In welke mate werd u gehinderd bij het opstaan uit bed?	◯ Niet ◯ Mild ◯ Matig ◯ Ernstig ◯ Zeer ernstig
10.27	In welke mate werd u gehinderd bij het sokken uittrekken?	◯ Niet ◯ Mild ◯ Matig ◯ Ernstig ◯ Zeer ernstig
10.28	In welke mate werd u gehinderd bij het in bed liggen (omdraaien, lange tijd uw voet/enkel in dezelfde positie houden)?	◯ Niet ◯ Mild ◯ Matig ◯ Ernstig ◯ Zeer ernstig

Paget LDA, *et al. BMJ Open* 2019; 9:e030961. doi: 10.1136/bmjopen-2019-030961

10.29	In welke mate werd u gehinderd bij het in/uit bad stappen?	◯ Niet ◯ Mild ◯ Matig ◯ Ernstig ◯ Zeer ernstig
10.30	In welke mate werd u gehinderd bij zitten?	◯ Niet ◯ Mild ◯ Matig ◯ Ernstig ◯ Zeer ernstig
10.31	In welke mate werd u gehinderd bij het toilet op en af gaan?	◯ Niet ◯ Mild ◯ Matig ◯ Ernstig ◯ Zeer ernstig
10.32	In welke mate werd u gehinderd bij zwaar huishoudelijk werk (bijvoorbeeld zware dozen sjouwen, vloer schrobben)?	◯ Niet ◯ Mild ◯ Matig ◯ Ernstig ◯ Zeer ernstig
10.33	In welke mate werd u gehinderd bij licht huishoudeijk werk (bijvoorbeeld koken, afstoffen)?	◯ Niet ◯ Mild ◯ Matig ◯ Ernstig ◯ Zeer ernstig
10.34	In welke mate werd u gehinderd bij hurken?	◯ Niet ◯ Mild ◯ Matig ◯ Ernstig ◯ Zeer ernstig
10.35	In welke mate werd u gehinderd bij hardlopen?	◯ Niet ◯ Mild ◯ Matig ◯ Ernstig ◯ Zeer ernstig
10.36	In welke mate werd u gehinderd bij springen?	◯ Niet ◯ Mild ◯ Matig ◯ Ernstig ◯ Zeer ernstig
10.37	In welke mate werd u gehinderd bij ronddraaien op uw aangedane voet/enkel?	◯ Niet ◯ Mild ◯ Matig ◯ Ernstig ◯ Zeer ernstig
10.38	In welke mate werd u gehinderd bij knielen?	◯ Niet ◯ Mild ◯ Matig ◯ Ernstig ◯ Zeer ernstig
10.39	Hoe vaak bent u zich bewust van uw voet/enkel probleem?	◯ Nooit ◯ Maandelijks ◯ Wekelijks ◯ Dagelijks ◯ Altijd
10.40	Heeft u uw leven veranderd om activiteiten te vermijden die schadelijk kunnen zijn voor uw voet/enkel?	◯ Niet<br>◯ Enigszins<br>◯ Matig<br>◯ Behoorlijk<br>◯ Volledig

Paget LDA, *et al. BMJ Open* 2019; 9:e030961. doi: 10.1136/bmjopen-2019-030961

10.41	In hoeverre kunt u op uw voet/enkel vertrouwen?	◯ Volledig<br>◯ Behoorlijk<br>◯ Matig<br>◯ Enigzins<br>◯ Niet
10.42	In het algemeen, in welke mate ondervindt u hinder van uw voet/enkel	◯ Geen<br>◯ Mild<br>◯ Matig<br>◯ Ernstig<br>◯ Zeer ernstig

Vragenlijst PRIMA studie 5 jaar - Dank

Number	Question	Answers
11.1	Hartelijk dank voor de tijd die u heeft genomen om de vragenlijsten in te vullen. Indien u nog opmerkingen heeft kunt u deze hierin plaatsen.	

Survey 'Vragenlijst PRIMA studie 52 weken'

Vragenlijst PRIMA studie 52 weken - AOFAS - Pijn

Number	Question	Answers
1.1	Hoeveel pijn ervaart u aan uw voet?	☐ Geen<br>☐ Milde pijnklachten of af en toe pijn<br>☐ Matige pijnklachten, dagelijks<br>☐ Vrijwel continu erge pijn

Vragenlijst PRIMA studie 52 weken - AOFAS resterende gedeelte

Paget LDA, *et al*. *BMJ Open* 2019; 9:e030961. doi: 10.1136/bmjopen-2019-030961

Number	Question	Answers
2.1	Hoeveel beperkingen in het dagelijks leven ervaart u?	◯ Geen beperkingen, geen hulpmiddelen nodig<br>◯ Geen beperkingen in algemeen dagelijkse bezigheden, wel beperkingen in recreatieve activiteiten (sport, hobbies, etc) geen ondersteuning<br>◯ Beperkingen bij dagelijkse- en recreatieve activiteiten, stok<br>◯ Ernstige beperkingen bij dagelijkse- en recreatieactiviteiten, walker, krukken, rolstoel, brace
2.2	Wat is de afstand die u in één keer kunt lopen?	◯ Meer dan 600 meter<br>◯ 400 - 600 meter<br>◯ 100 - 300 meter<br>◯ Minder dan 100 meter
2.3	Heeft u moeite met lopen op een bepaalde ondergrond?	◯ Geen problemen bij lopen<br>◯ Enig problemen op ongelijke ondergrond, trap, helling, ladders<br>◯ Ernstige problemen bij ongelijke ondergrond, trap, helling, ladders
2.4	Hoe zou u uw looppatroon beoordelen?	◯ Normaal of minimaal afwijkend<br>◯ Duidelijk<br>◯ Ernstig
2.5	Hoe zou u de beweeglijkheid van uw voet beoordelen ten opzicht van de andere zijde of ten opzicte van vóór het ongeval?	◯ Hetzelfde of licht beperkt t.o.v. de niet aangedane zijde of zoals voor het ongeval<br>◯ Ongeveer de helft beperkt<br>◯ Sterk verminderd, bijna geen bewegingen mogelijk
2.6	Hoe zou u de beweeglijkheid van uw enkel beoordelen ten opzichte van de andere zijde of ten opzichte van voor het ongeval?	◯ Hetzelfde of licht beperkt t.o.v. de niet aangedane zijde of zoals voor het ongeval<br>◯ Ongeveer de helft beperkt<br>◯ Sterk verminderd, bijna geen bewegingen mogelijk
2.7	Hoe zou u de stabiliteit van uw voet en enkel beoordelen	◯ Stabiel, ik verzwik mijn enkel hooguit incidenteel<br>◯ Niet stabiel, ik verzwik mijn enkel vaak

Paget LDA, *et al. BMJ Open* 2019; 9:e030961. doi: 10.1136/bmjopen-2019-030961

2.8	Hoe zou u de stand van uw voet beoordelen ten opzichte van de andere zijde of ten opzichte van voor het ongeval?	◯ Goed, ik kan mijn voet goed plat op de grond zetten, waarbij de stand niet afwijkend is<br>◯ Ik sta vrijwel alleen op de buitenzijde van mijn voet<br>◯ Ik kan mijn voet niet plat op de grond zetten en loop vrijwel alleen op mijn voorvoet

Vragenlijst PRIMA studie 52 weken - VAS Pain score

Number	Question	Answers	
3.1	VAS pijnscore (Geef uw minimaal pijnscore aan tijdens dagelijkse activiteiten op een schaal van 0 tot 100, waarbij 0 geen pijn is en 100 de ergste pijn denkbaar is)	(0.00)	100 (100.00)

Vragenlijst PRIMA studie 52 weken - AAS

Number	Question	Answers

Paget LDA, *et al*. *BMJ Open* 2019; 9:e030961. doi: 10.1136/bmjopen-2019-030961

4.1	Wat is het meest bij u van toepassing ten aanzien van uw acitviteit?	☐ American Football<br>☐ Basketbal<br>☐ Gymnastiek/turnen<br>☐ Handbal<br>☐ Rugby<br>☐ Voetbal<br>☐ Hockey<br>☐ Korfbal<br>☐ Gevechtssporten: judo, karate, kung fu, taekwando, jiu jitsu, etc.<br>☐ Oriëntatielopen<br>☐ Rhytmische gymnastiek<br>☐ Volleybal<br>☐ Boxen<br>☐ Freestyle snowboarden<br>☐ Ijshockey<br>☐ Tennis<br>☐ Worstelen<br>☐ Fitness, aerobics<br>☐ Badminton<br>☐ Baseball<br>☐ Cross-country hardlopen<br>☐ Moderne pentathlon<br>☐ Squash<br>☐ Surfen, windsurfen<br>☐ Tafel tennis<br>☐ Atletiek: spring-, werponderdelen<br>☐ Waterskieën<br>☐ Dans<br>☐ Schermen<br>☐ Zaalhockey<br>☐ Bergbeklimmen<br>☐ Langlauf<br>☐ Parachute springen<br>☐ Softball<br>☐ Speciale beroepen en werkactiviteiten; speciale beroepen: ballet, professioneel soldaat, speciale reddingswerker, stuntman, etc.<br>☐ Duiken<br>☐ Scubaduiken<br>☐ Skaten, in-line skaten<br>☐ Atletiek: looponderdelen<br>☐ Triatlon<br>☐ Gewichtsheffen, body-building

		☐ Alle competitieve sporten hieronder met 'seasonal' conditioning<br>☐ Zwaar fysiek werk<br>☐ Alpine skiën en snowboarden<br>☐ Bowlen/curlen<br>☐ Golf<br>☐ Mountainbike/BMX<br>☐ Powerliften<br>☐ Zeilen<br>☐ Fysiek werk<br>☐ Wielrennen<br>☐ Paardrijden<br>☐ Motorsporten/technische sporten<br>☐ Roeien, kayakken<br>☐ Boogschieten<br>☐ Water polo en zwemmen<br>☐ Kunnen lopen op oneven grond<br>☐ Geen sport, echter geen limitatie in dagelijkse activiteiten<br>☐ Kunnen lopen op even grond, maar de dagelijkste activiteiten zijn gelimiteerd<br>☐ Niet kunnen lopen, fysieke handicap wegens enkelproblemen
4.1.1	***If 'Wat is het meest bij u van toepassing ten aanzien van uw acitviteit?' is equal to 'American Football' answer this question:***<br>American Football	○ Professioneel<br>○ Competitief<br>○ Recreatief
4.1.2	***If 'Wat is het meest bij u van toepassing ten aanzien van uw acitviteit?' is equal to 'Basketbal' answer this question:***<br>Basketbal	○ Professioneel<br>○ Competitief<br>○ Recreatief
4.1.3	***If 'Wat is het meest bij u van toepassing ten aanzien van uw acitviteit?' is equal to 'Gymnastiek/turnen' answer this question:***<br>Gymnastiek/turnen	○ Professioneel<br>○ Competitief<br>○ Recreatief
4.1.4	***If 'Wat is het meest bij u van toepassing ten aanzien van uw acitviteit?' is equal to 'Handbal' answer this question:***<br>Handbal	○ Professioneel<br>○ Competitief<br>○ Recreatief
4.1.5	***If 'Wat is het meest bij u van toepassing ten aanzien van uw acitviteit?' is equal to 'Rugby' answer this question:***<br>Rugby	○ Professioneel<br>○ Competitief<br>○ Recreatief

4.1.6	***If 'Wat is het meest bij u van toepassing ten aanzien van uw acitviteit?' is equal to 'Voetbal' answer this question:***<br>Voetbal	◯ Professioneel<br>◯ Competitief<br>◯ Recreatief
4.1.7	***If 'Wat is het meest bij u van toepassing ten aanzien van uw acitviteit?' is equal to 'Hockey' answer this question:***<br>Hockey	◯ Professioneel<br>◯ Competitief<br>◯ Recreatief
4.1.8	***If 'Wat is het meest bij u van toepassing ten aanzien van uw acitviteit?' is equal to 'Korfbal' answer this question:***<br>Korfbal	◯ Professioneel<br>◯ Competitief<br>◯ Recreatief
4.1.9	***If 'Wat is het meest bij u van toepassing ten aanzien van uw acitviteit?' is equal to 'Gevechtssporten: judo, karate, kung fu, taekwando, jiu jitsu, etc.' answer this question:***<br>Gevechtssporten: judo, karate, kung fu, taekwando, jiu jitsu, etc.	◯ Professioneel<br>◯ Competitief<br>◯ Recreatief
4.1.10	***If 'Wat is het meest bij u van toepassing ten aanzien van uw acitviteit?' is equal to 'Oriëntatielopen' answer this question:***<br>Oriëntatielopen	◯ Professioneel<br>◯ Competitief<br>◯ Recreatief
4.1.11	***If 'Wat is het meest bij u van toepassing ten aanzien van uw acitviteit?' is equal to 'Rhytmische gymnastiek' answer this question:***<br>Rhytmisch gymnastiek	◯ Professioneel<br>◯ Competitief<br>◯ Recreatief
4.1.12	***If 'Wat is het meest bij u van toepassing ten aanzien van uw acitviteit?' is equal to 'Volleybal' answer this question:***<br>Volleybal	◯ Professioneel<br>◯ Competitief<br>◯ Recreatief
4.1.13	***If 'Wat is het meest bij u van toepassing ten aanzien van uw acitviteit?' is equal to 'Boxen' answer this question:***<br>Boxen	◯ Professioneel<br>◯ Competitief<br>◯ Recreatief
4.1.14	***If 'Wat is het meest bij u van toepassing ten aanzien van uw acitviteit?' is equal to 'Freestyle snowboarden' answer this question:***<br>Freestyle snowboarden	◯ Professioneel<br>◯ Competitief<br>◯ Recreatief
4.1.15	***If 'Wat is het meest bij u van toepassing ten aanzien van uw acitviteit?' is equal to 'Freestyle snowboarden' answer this question:***<br>IJshockey	◯ Professioneel<br>◯ Competitief<br>◯ Recreatief

Paget LDA, *et al. BMJ Open* 2019; 9:e030961. doi: 10.1136/bmjopen-2019-030961

4.1.16	***If 'Wat is het meest bij u van toepassing ten aanzien van uw acitviteit?' is equal to 'Tennis' answer this question:***<br>Tennis	◯ Professioneel<br>◯ Competitief<br>◯ Recreatief
4.1.17	***If 'Wat is het meest bij u van toepassing ten aanzien van uw acitviteit?' is equal to 'Worstelen' answer this question:***<br>Worstelen	◯ Professioneel<br>◯ Competitief<br>◯ Recreatief
4.1.18	***If 'Wat is het meest bij u van toepassing ten aanzien van uw acitviteit?' is equal to 'Fitness, aerobics' answer this question:***<br>Fitness, aerobics	◯ Professioneel<br>◯ Competitief<br>◯ Recreatief
4.1.19	***If 'Wat is het meest bij u van toepassing ten aanzien van uw acitviteit?' is equal to 'Badminton' answer this question:***<br>Badminton	◯ Professioneel<br>◯ Competitief<br>◯ Recreatief
4.1.20	***If 'Wat is het meest bij u van toepassing ten aanzien van uw acitviteit?' is equal to 'Baseball' answer this question:***<br>Baseball	◯ Professioneel<br>◯ Competitief<br>◯ Recreatief
4.1.21	***If 'Wat is het meest bij u van toepassing ten aanzien van uw acitviteit?' is equal to 'Cross-country hardlopen' answer this question:***<br>Cross-country hardlopen	◯ Professioneel<br>◯ Competitief<br>◯ Recreatief
4.1.22	***If 'Wat is het meest bij u van toepassing ten aanzien van uw acitviteit?' is equal to 'Moderne pentathlon' answer this question:***<br>Moderne pentathlon	◯ Professioneel<br>◯ Competitief<br>◯ Recreatief
4.1.23	***If 'Wat is het meest bij u van toepassing ten aanzien van uw acitviteit?' is equal to 'Squash' answer this question:***<br>Squash	◯ Professioneel<br>◯ Competitief<br>◯ Recreatief
4.1.24	***If 'Wat is het meest bij u van toepassing ten aanzien van uw acitviteit?' is equal to 'Surfen, windsurfen' answer this question:***<br>Surfen, windsurfen	◯ Professioneel<br>◯ Competitief<br>◯ Recreatief
4.1.25	***If 'Wat is het meest bij u van toepassing ten aanzien van uw acitviteit?' is equal to 'Tafel tennis' answer this question:***<br>Tafel tennis	◯ Professioneel<br>◯ Competitief<br>◯ Recreatief

Paget LDA, *et al. BMJ Open* 2019; 9:e030961. doi: 10.1136/bmjopen-2019-030961

4.1.26	***If 'Wat is het meest bij u van toepassing ten aanzien van uw acitviteit?' is equal to 'Atletiek: spring-, werponderdelen' answer this question:*** Atletiek: spring-, werponderdelen	◯ Professioneel<br>◯ Competitief<br>◯ Recreatief
4.1.27	***If 'Wat is het meest bij u van toepassing ten aanzien van uw acitviteit?' is equal to 'Waterskieën' answer this question:*** Waterskien	◯ Professioneel<br>◯ Competitief<br>◯ Recreatief
4.1.28	***If 'Wat is het meest bij u van toepassing ten aanzien van uw acitviteit?' is equal to 'Dans' answer this question:*** Dans	◯ Professioneel<br>◯ Competitief<br>◯ Recreatief
4.1.29	***If 'Wat is het meest bij u van toepassing ten aanzien van uw acitviteit?' is equal to 'Schermen' answer this question:*** Schermen	◯ Professioneel<br>◯ Competitief<br>◯ Recreatief
4.1.30	***If 'Wat is het meest bij u van toepassing ten aanzien van uw acitviteit?' is equal to 'Zaalhockey' answer this question:*** Zaalhockey	◯ Professioneel<br>◯ Competitief<br>◯ Recreatief
4.1.31	***If 'Wat is het meest bij u van toepassing ten aanzien van uw acitviteit?' is equal to 'Bergbeklimmen' answer this question:*** Bergbeklimmen	◯ Professioneel<br>◯ Competitief<br>◯ Recreatief
4.1.32	***If 'Wat is het meest bij u van toepassing ten aanzien van uw acitviteit?' is equal to 'Langlauf' answer this question:*** Langlauf	◯ Professioneel<br>◯ Competitief<br>◯ Recreatief
4.1.33	***If 'Wat is het meest bij u van toepassing ten aanzien van uw acitviteit?' is equal to 'Parachute springen' answer this question:*** Parachute springen	◯ Professioneel<br>◯ Competitief<br>◯ Recreatief
4.1.34	***If 'Wat is het meest bij u van toepassing ten aanzien van uw acitviteit?' is equal to 'Softball' answer this question:*** Softball	◯ Professioneel<br>◯ Competitief<br>◯ Recreatief

Paget LDA, *et al. BMJ Open* 2019; 9:e030961. doi: 10.1136/bmjopen-2019-030961

4.1.35	***If 'Wat is het meest bij u van toepassing ten aanzien van uw acitviteit?' is equal to 'Speciale beroepen en werkactiviteiten; speciale beroepen: ballet, professioneel soldaat, speciale reddingswerker, stuntman, etc.' answer this question:***<br>Speciale beroepen en werkactiviteiten speciale beroepen: ballet, professioneel soldaat, speciale reddingswerker, stuntman, etc.	○ ballet, professioneel soldaat, speciale reddingswerker, stuntman, etc.
4.1.36	***If 'Wat is het meest bij u van toepassing ten aanzien van uw acitviteit?' is equal to 'Duiken' answer this question:***<br>Duiken	○ Professioneel/competitief<br>○ Recreatief
4.1.37	***If 'Wat is het meest bij u van toepassing ten aanzien van uw acitviteit?' is equal to 'Scubaduiken' answer this question:***<br>Scubaduiken	○ Professioneel/competitief<br>○ Recreatief
4.1.38	***If 'Wat is het meest bij u van toepassing ten aanzien van uw acitviteit?' is equal to 'Skaten, in-line skaten' answer this question:***<br>skaten, in-linen skaten	○ Professioneel/competitief<br>○ Recreatief
4.1.39	***If 'Wat is het meest bij u van toepassing ten aanzien van uw acitviteit?' is equal to 'Atletiek: looponderdelen' answer this question:***<br>Atletiek: looponderdelen	○ Professioneel/competitief<br>○ Recreatief
4.1.40	***If 'Wat is het meest bij u van toepassing ten aanzien van uw acitviteit?' is equal to 'Triatlon' answer this question:***<br>triatlon	○ Professioneel/competitief<br>○ Recreatief
4.1.41	***If 'Wat is het meest bij u van toepassing ten aanzien van uw acitviteit?' is equal to 'Gewichtsheffen, body-building' answer this question:***<br>Gewichtsheffen, body-building	○ Professioneel/competitief<br>○ Recreatief
4.1.42	***If 'Wat is het meest bij u van toepassing ten aanzien van uw acitviteit?' is equal to 'Alle competitieve sporten hieronder met ‘seasonal’ conditioning' answer this question:***<br>Alle competitieve sporten hieronder met ‘seasonal’ conditioning	○ Alle competitieve sporten hieronder met ‘seasonal’ conditioning
4.1.43	***If 'Wat is het meest bij u van toepassing ten aanzien van uw acitviteit?' is equal to 'Zwaar fysiek werk' answer this question:***<br>Zwaar fysiek werk	○ Alle competitieve sporten hieronder met ‘seasonal’ conditioning

Paget LDA, *et al. BMJ Open* 2019; 9:e030961. doi: 10.1136/bmjopen-2019-030961

4.1.44	***If 'Wat is het meest bij u van toepassing ten aanzien van uw acitviteit?' is equal to 'Alpine skiën en snowboarden' answer this question:***<br>Alpine skien en snowboarden	◯ Alpine skien en snowboarden
4.1.45	***If 'Wat is het meest bij u van toepassing ten aanzien van uw acitviteit?' is equal to 'Bowlen/curlen' answer this question:***<br>Bowlen/curlen	◯ Bowlen/curlen
4.1.46	***If 'Wat is het meest bij u van toepassing ten aanzien van uw acitviteit?' is equal to 'Golf' answer this question:***<br>Golf	◯ Golf
4.1.47	***If 'Wat is het meest bij u van toepassing ten aanzien van uw acitviteit?' is equal to 'Mountainbike/BMX' answer this question:***<br>Mountainbike/BMX	◯ Mountainbike/BMX
4.1.48	***If 'Wat is het meest bij u van toepassing ten aanzien van uw acitviteit?' is equal to 'Powerliften' answer this question:***<br>Powerliften	◯ Powerliften
4.1.49	***If 'Wat is het meest bij u van toepassing ten aanzien van uw acitviteit?' is equal to 'Zeilen' answer this question:***<br>Zeilen	◯ Zeilen
4.1.50	***If 'Wat is het meest bij u van toepassing ten aanzien van uw acitviteit?' is equal to 'Fysiek werk' answer this question:***<br>Fysiek werk	◯ Fysiek werk
4.1.51	***If 'Wat is het meest bij u van toepassing ten aanzien van uw acitviteit?' is equal to 'Wielrennen' answer this question:***<br>Wielrennen	◯ Wielrennen
4.1.52	***If 'Wat is het meest bij u van toepassing ten aanzien van uw acitviteit?' is equal to 'Paardrijden' answer this question:***<br>Paardrijden	◯ Paardrijden
4.1.53	***If 'Wat is het meest bij u van toepassing ten aanzien van uw acitviteit?' is equal to 'Motorsporten/technische sporten' answer this question:***<br>Motorsporten/technische sporten	◯ Motorsporten/technische sporten
4.1.54	***If 'Wat is het meest bij u van toepassing ten aanzien van uw acitviteit?' is equal to 'Roeien, kayakken' answer this question:***<br>Roeien, kayakken	◯ Roeien, kayakken

Paget LDA, *et al. BMJ Open* 2019; 9:e030961. doi: 10.1136/bmjopen-2019-030961

4.1.55	***If 'Wat is het meest bij u van toepassing ten aanzien van uw acitviteit?' is equal to 'Boogschieten' answer this question:*** Boogschieten	◯ Boogschieten
4.1.56	***If 'Wat is het meest bij u van toepassing ten aanzien van uw acitviteit?' is equal to 'Water polo en zwemmen' answer this question:*** Water polo en zwemmen	◯ Water polo en zwemmen
4.1.57	***If 'Wat is het meest bij u van toepassing ten aanzien van uw acitviteit?' is equal to 'Kunnen lopen op oneven grond' answer this question:*** Kunnen lopen op oneven grond	◯ Kunnen lopen op oneven grond
4.1.58	***If 'Wat is het meest bij u van toepassing ten aanzien van uw acitviteit?' is equal to 'Geen sport, echter geen limitatie in dagelijkse activiteiten' answer this question:*** Geen sport, echter geen limitatie in dagelijkse activiteiten	◯ Geen sport, echter geen limitatie in dagelijkse activiteiten
4.1.59	***If 'Wat is het meest bij u van toepassing ten aanzien van uw acitviteit?' is equal to 'Kunnen lopen op even grond, maar de dagelijkste activiteiten zijn gelimiteerd' answer this question:*** Kunnen lopen op even grond, maar de dagelijkste activiteiten zijn gelimiteerd	◯ Kunnen lopen op even grond, maar de dagelijkste activiteiten zijn gelimiteerd
4.1.60	***If 'Wat is het meest bij u van toepassing ten aanzien van uw acitviteit?' is equal to 'Niet kunnen lopen, fysieke handicap wegens enkelproblemen' answer this question:*** Niet kunnen lopen, fysieke handicap wegens enkelproblemen	◯ Niet kunnen lopen, fysieke handicap wegens enkelproblemen

Vragenlijst PRIMA studie 52 weken - Hoe tevreden bent u over de enkelklachten?

Number	Question	Answers
5.1	Hoe tevreden bent u over de enkelklachten?	◯ Slecht<br>◯ Matig<br>◯ Goed<br>◯ Uitstekend

Paget LDA, *et al. BMJ Open* 2019; 9:e030961. doi: 10.1136/bmjopen-2019-030961

Vragenlijst PRIMA studie 52 weken - SF-36

Number	Question	Answers
	Deze vragenlijst gaat over uw standpunten t.a.v. uw gezondheid. Met behulp van deze gegevens kan worden bijgehouden hoe u zich voelt en hoe goed u in staat bent uw gebruikelijke bezigheden uit te voeren. Beantwoord elke vraag door een antwoord aan te klikken. Als u niet zeker weet hoe u een vraag moet beantwoorden, geef dan het best mogelijke antwoord.	
6.1	Hoe zou u over het algemeen uw gezondheid noemen?	○ Uitstekend ○ Zeer goed ○ Goed ○ Matig ○ Slecht
6.2	Hoe beoordeelt u nu uw gezondheid over het algemeen vergeleken met een jaar geleden?	○ Veel beter dan een jaar geleden ○ Wat beter dan een jaar geleden ○ Ongeveer hetzelfde als een jaar geleden ○ Wat slechter dan een jaar geleden ○ Veel slechter dan een jaar geleden
6.3	Wordt u door uw gezondheid op dit moment beperkt bij forse inspanning, zoals hardlopen, tillen van zware voorwerpen of een veeleisende sport beoefenen?	○ Ja, ernstig beperkt ○ Ja, een beetje beperkt ○ Nee, helemaal niet beperkt
6.4	Wordt u door uw gezondheid op dit moment beperkt bij matige inspanning zoals een tafel verplaatsen, stofzuigen, zwemmen of fietsen?	○ Ja, ernstig beperkt ○ Ja, een beetje beperkt ○ Nee, helemaal niet beperkt
6.5	Wordt u door uw gezondheid op dit moment beperkt bij boodschappen tillen of dragen?	○ Ja, ernstig beperkt ○ Ja, een beetje beperkt ○ Nee, helemaal niet beperkt
6.6	Wordt u door uw gezondheid op dit moment beperkt bij een paar trappen oplopen?	○ Ja, ernstig beperkt ○ Ja, een beetje beperkt ○ Nee, helemaal niet beperkt
6.7	Wordt u door uw gezondheid op dit moment beperkt bij één trap oplopen?	○ Ja, ernstig beperkt ○ Ja, een beetje beperkt ○ Nee, helemaal niet beperkt
6.8	Wordt u door uw gezondheid op dit moment beperkt bij bukken, knielen of hurken?	○ Ja, ernstig beperkt ○ Ja, een beetje beperkt ○ Nee, helemaal niet beperkt
6.9	Wordt u door uw gezondheid op dit moment beperkt bij meer dan één kilometer lopen?	○ Ja, ernstig beperkt ○ Ja, een beetje beperkt ○ Nee, helemaal niet beperkt
6.10	Wordt u door uw gezondheid op dit moment beperkt bij een paar honderd meter lopen?	○ Ja, ernstig beperkt ○ Ja, een beetje beperkt ○ Nee, helemaal niet beperkt

Paget LDA, *et al. BMJ Open* 2019; 9:e030961. doi: 10.1136/bmjopen-2019-030961

6.11	Wordt u door uw gezondheid op dit moment beperkt bij ongeveer honderd meter lopen?	◯ Ja, ernstig beperkt ◯ Ja, een beetje beperkt ◯ Nee, helemaal niet beperkt
6.12	Wordt u door uw gezondheid op dit moment beperkt bij uzelf wassen of aankleden?	◯ Ja, ernstig beperkt ◯ Ja, een beetje beperkt ◯ Nee, helemaal niet beperkt
6.13	U besteedde in de afgelopen 4 weken minder tijd aan werk of andere bezigheden	◯ Ja ◯ Nee
6.14	U heeft in de afgelopen 4 weken minder bereikt dan u zou willen	◯ Ja ◯ Nee
6.15	U was in de afgelopen 4 weken beperkt in het soort werk of andere bezigheden.	◯ Ja ◯ Nee
6.16	U had de afgelopen 4 weken moeite om uw werk of andere bezigheden uit te voeren (het kostte u bijvoorbeeld extra inspanning).	◯ Ja ◯ Nee
6.17	U besteedde in de afgelopen 4 weken minder tijd aan werk of andere bezigheden ten gevolge van emotionele problemen	◯ Ja ◯ Nee
6.18	U heeft in de afgelopen 4 weken minder bereikt dan u zou willen ten gevolge van emotionele problemen.	◯ Ja ◯ Nee
6.19	U deed de afgelopen 4 weken uw werk of andere bezigheden niet zo zorgvuldig als gewoonlijk ten gevolge van emotionele problemen.	◯ Ja ◯ Nee
6.20	In hoeverre hebben uw lichamelijke gezondheid of emotionele problemen u gedurende de afgelopen 4 weken gehinderd in uw normale omgang met familie, vrienden of buren, of bij activiteiten in groepsverband?	◯ Helemaal niet ◯ Enigszins ◯ Nogal ◯ Veel ◯ Heel erg veel
6.21	Hoeveel lichamelijke pijn heeft u de afgelopen 4 weken gehad?	◯ Geen<br>◯ Heel licht<br>◯ Licht<br>◯ Nogal<br>◯ Ernstig<br>◯ Heel ernstig
6.22	In welke mate bent u de afgelopen 4 weken door de pijn gehinderd in uw normale werk (zowel werk buitenshuis als huishoudelijk werk)?	◯ Helemaal niet<br>◯ Een klein beetje<br>◯ Nogal<br>◯ Veel<br>◯ Heel erg veel

Paget LDA, *et al. BMJ Open* 2019; 9:e030961. doi: 10.1136/bmjopen-2019-030961

6.23	Voelde u zich levenslustig?	◯ Altijd<br>◯ Meestal<br>◯ Vaak<br>◯ Soms<br>◯ Zelden<br>◯ Nooit
6.24	Was u erg zenuwachtig?	◯ Altijd<br>◯ Meestal<br>◯ Vaak<br>◯ Soms<br>◯ Zelden<br>◯ Nooit
6.25	Zat u zo in de put dat u niets kon?	◯ Altijd<br>◯ Meestal<br>◯ Vaak<br>◯ Soms<br>◯ Zelden<br>◯ Nooit
6.26	Voelde u zich rustig en tevreden?	◯ Altijd<br>◯ Meestal<br>◯ Vaak<br>◯ Soms<br>◯ Zelden<br>◯ Nooit
6.27	Had u veel energie?	◯ Altijd<br>◯ Meestal<br>◯ Vaak<br>◯ Soms<br>◯ Zelden<br>◯ Nooit
6.28	Voelde u zich somber en neerslachtig?	◯ Altijd<br>◯ Meestal<br>◯ Vaak<br>◯ Soms<br>◯ Zelden<br>◯ Nooit

Paget LDA, *et al. BMJ Open* 2019; 9:e030961. doi: 10.1136/bmjopen-2019-030961

6.29	Voelde u zich uitgeput?	◯ Altijd<br>◯ Meestal<br>◯ Vaak<br>◯ Soms<br>◯ Zelden<br>◯ Nooit
6.30	Was u een gelukkig mens?	◯ Altijd<br>◯ Meestal<br>◯ Vaak<br>◯ Soms<br>◯ Zelden<br>◯ Nooit
6.31	Voelde u zich moe?	◯ Altijd<br>◯ Meestal<br>◯ Vaak<br>◯ Soms<br>◯ Zelden<br>◯ Nooit
6.32	Hoe vaak hebben uw lichamelijke gezondheid of emotionele problemen u gedurende de afgelopen 4 weken gehinderd bij uw sociale activiteiten (zoals vrienden of familie bezoeken etc.)?	◯ Altijd<br>◯ Meestal<br>◯ Soms<br>◯ Zelden<br>◯ Nooit
6.33	Ik lijk wat gemakkelijker ziek te worden dan andere mensen	◯ Volkomen juist<br>◯ Grotendeels juist<br>◯ Weet ik niet<br>◯ Grotendeels onjuist<br>◯ Volkomen onjuist
6.34	Ik ben even gezond als andere mensen die ik ken	◯ Volkomen juist<br>◯ Grotendeels juist<br>◯ Weet ik niet<br>◯ Grotendeels onjuist<br>◯ Volkomen onjuist

Paget LDA, *et al. BMJ Open* 2019; 9:e030961. doi: 10.1136/bmjopen-2019-030961

6.35	Ik verwacht dat mijn gezondheid achteruit zal gaan	◯ Volkomen juist<br>◯ Grotendeels juist<br>◯ Weet ik niet<br>◯ Grotendeels onjuist<br>◯ Volkomen onjuist
6.36	Mijn gezondheid is uitstekend	◯ Volkomen juist<br>◯ Grotendeels juist<br>◯ Weet ik niet<br>◯ Grotendeels onjuist<br>◯ Volkomen onjuist

Vragenlijst PRIMA studie 52 weken - GAS

Number	Question	Answers
	Tijdens de eerste afspraak werd een duidelijk doel met u afgesproken. Dit werd tevens per email naar u gestuurd. Hoe is de situatie nu ten aanzien van de destijds gestelde doelstelling?	
7.1	Wat is nu de situatie ten aanzien van het tijdens de eerste afspraak afgesproken doelstelling (Goal attainment Scaling)?	◯ Achteruitgang (minder dan de utigangssituatie)<br>◯ Uitgangssituatie<br>◯ Minder dan het doel<br>◯ Doel<br>◯ Meer dan het doel<br>◯ Veel meer dan het doel

Vragenlijst PRIMA studie 52 weken - EQ-5D-3L

Number	Question	Answers
	Deze vragenlijst gaat over uw standpunten t.a.v. uw gezondheid. Met behulp van deze gegevens kan worden bijgehouden hoe u zich voelt en hoe goed u in staat bent uw gebruikelijke bezigheden uit te voeren. Beantwoord elke vraag door een antwoord aan te klikken. Als u niet zeker weet hoe u een vraag moet beantwoorden, geef dan het best mogelijke antwoord.	
8.1	Hoe is het met uw Mobiliteit gesteld?	◯ Ik heb geen problemen met lopen<br>◯ Ik heb enige problemen met lopen<br>◯ Ik ben bedlegerig

8.2	Hoe is het met uw Zelfzorg gesteld?	◯ Ik heb geen problemen om mijzelf te wassen of aan te kleden<br>◯ Ik heb enige problemen om mijzelf te wassen of aan te kleden<br>◯ Ik ben niet in staat om mijzelf te wassen of aan te kleden
8.3	Hoe is het met de Dagelijkse activiteiten (werk, studie, huishouden, gezins- en vrijetijdsactiviteiten) gesteld?	◯ Ik heb geen problemen met mijn dagelijkse activiteiten<br>◯ Ik heb enige problemen met mijn dagelijkse activiteiten<br>◯ Ik ben niet in staat om mijn dagelijkse activiteiten uit te voeren
8.4	Hoe is het met de Pijn/klachten gesteld?	◯ Ik heb geen pijn of andere klachten<br>◯ Ik heb matige pijn of andere klachten<br>◯ Ik heb zeer ernstige pijn of andere klachten
8.5	Hoe is het met de Stemming gesteld?	◯ Ik ben niet angstig of somber<br>◯ Ik ben matig angstig of somber<br>◯ Ik ben erg angstig of somber
	We willen weten hoe goed of slecht uw gezondheid VANDAAG is. Deze meetschaal loopt van 0 tot 100: 100 staat voor de BESTE gezondheid die u zich kunt voorstellen - 0 staat voor de SLECHTSTE gezondheid die u zich kunt voorstellen	
8.6	Uw Gezondheid vandaag	(0.00) (100.00)

Vragenlijst PRIMA studie 52 weken - AOS

Number	Question	Answers
	Instructies: De lijn naast elke vraag staat voor hoeveel PIJN u heeft in verschillende situaties. De linker kant (0) is “geen pijn” en de rechter kant (100) is “ergste pijn denkbaar”. Geef voor de onderstaande situaties op de lijn aan hoeveel PIJN u in de afgelopen week in de enkel had. Als een of meerdere van deze situaties niet van toepassing waren, dan kiest u de "niet van toepassing" optie.	
9.1	Wat was de hoogte van de ergste pijn in de afgelopen week?	(0.00) (100.00)
9.2	Heeft u pijn als u 's ochtends opstaat?	◯ Ja<br>◯ Nee<br>◯ Niet van toepassing

Paget LDA, *et al*. *BMJ Open* 2019; 9:e030961. doi: 10.1136/bmjopen-2019-030961

9.2.1	***If 'Heeft u pijn als u 's ochtends opstaat?' is equal to 'Ja' answer this question:*** Hoeveel pijn heeft u voordat u 's ochtends opstaat?	(0.00)	(100.00)
9.3	Heeft u pijn wanneer u op blote voeten loopt?	○ Ja<br>○ Nee<br>○ Niet van toepassing	
9.3.1	***If 'Heeft u pijn wanneer u op blote voeten loopt?' is equal to 'Ja' answer this question:*** Hoeveel pijn heeft u als u op blote voeten loopt?	(0.00)	(100.00)
9.4	Heeft u pijn wanneer u op blote voeten staat?	○ Ja<br>○ Nee<br>○ Niet van toepassing	
9.4.1	***If 'Heeft u pijn wanneer u op blote voeten staat?' is equal to 'Ja' answer this question:*** Hoeveel pijn heeft u als u op blote voeten staat?	(0.00)	(100.00)
9.5	Heeft u pijn wanneer u met schoenen loopt?	○ Ja<br>○ Nee<br>○ Niet van toepassing	
9.5.1	***If 'Heeft u pijn wanneer u met schoenen loopt?' is equal to 'Ja' answer this question:*** Hoeveel pijn heeft u wanneer u met schoenen loopt?	(0.00)	(100.00)
9.6	Heeft u pijn wanneer u met schoenen staat?	○ Ja<br>○ Nee<br>○ Niet van toepassing	
9.6.1	***If 'Heeft u pijn wanneer u met schoenen staat?' is equal to 'Ja' answer this question:*** Hoeveel pijn heeft u wanneer u met schoenen staat?	(0.00)	(100.00)
9.7	Heeft u pijn wanneer u loopt met steunzolen of een brace?	○ Ja<br>○ Nee<br>○ Niet van toepassing	
9.7.1	***If 'Heeft u pijn wanneer u loopt met steunzolen of een brace?' is equal to 'Ja' answer this question:*** Hoeveel pijn heeft u wanneer u loopt met steunzolen of een brace?	(0.00)	(100.00)

Paget LDA, *et al. BMJ Open* 2019; 9:e030961. doi: 10.1136/bmjopen-2019-030961

9.8	Heeft u pijn wanneer u staat met steunzolen of een brace?	◯ Ja<br>◯ Nee<br>◯ Niet van toepassing
9.8.1	***If 'Heeft u pijn wanneer u staat met steunzolen of een brace?' is equal to 'Ja' answer this question:***<br>Hoeveel pijn heeft u wanneer u staat met steunzolen of een brace?	(0.00) (100.00)
9.9	Heeft u pijn aan het einde van de dag?	◯ Ja<br>◯ Nee<br>◯ Niet van toepassing
9.9.1	***If 'Heeft u pijn aan het einde van de dag?' is equal to 'Ja' answer this question:***<br>Hoeveel pijn heeft u aan het einde van de dag?	(0.00) (100.00)
	Instructies:De lijn naast elke vraag staat voor hoeveel MOEITE u heeft met verschillende activiteiten. De linker kant (0) is “Niet moeilijk” en de rechter kant (100) is “Te moeilijk, niet uitvoerbaar”. Geef voor de onderstaande activiteiten op de lijn aan hoeveel MOEITE u in de afgelopen week door enkelklachten had met onderstaande activiteiten. Als een situatie niet van toepassing is, kies dan de optie "niet van toepassing".	
9.10	Heeft u moeite met door het huis lopen?	◯ Ja<br>◯ Nee<br>◯ Niet van toepassing
9.10.1	***If 'Heeft u moeite met door het huis lopen?' is equal to 'Ja' answer this question:***<br>Hoeveel moeite heeft u met door het huis lopen?	(0.00) (100.00)
9.11	Heeft u moeite met buiten lopen op oneven ondergrond?	◯ Ja<br>◯ Nee<br>◯ Niet van toepassing
9.11.1	***If 'Heeft u moeite met buiten lopen op oneven ondergrond?' is equal to 'Ja' answer this question:***<br>Hoeveel moeite heeft u met buiten lopen op oneven ondergrond?	(0.00) (100.00)
9.12	Heeft u moeite met een paar honderd meter lopen?	◯ Ja<br>◯ Nee<br>◯ Niet van toepassing
9.12.1	***If 'Heeft u moeite met een paar honderd meter lopen?' is equal to 'Ja' answer this question:***<br>Hoeveel moeite heeft u met een paar honderd meter lopen?	(0.00) (100.00)

Paget LDA, *et al*. *BMJ Open* 2019; 9:e030961. doi: 10.1136/bmjopen-2019-030961

9.13	Heeft u moeite met een trap oplopen?	◯ Ja<br>◯ Nee<br>◯ Niet van toepassing	
9.13.1	***If 'Heeft u moeite met een trap oplopen?' is equal to 'Ja' answer this question:***<br>Hoeveel moeite heeft u met een trap oplopen?	(0.00)	(100.00)
9.14	Heeft u moeite met een trap aflopen?	◯ Ja<br>◯ Nee<br>◯ Niet van toepassing	
9.14.1	***If 'Heeft u moeite met een trap aflopen?' is equal to 'Ja' answer this question:***<br>Hoeveel moeite heeft u met een trap aflopen?	(0.00)	(100.00)
9.15	Heeft u moeite met op de tenen staan?	◯ Ja<br>◯ Nee<br>◯ Niet van toepassing	
9.15.1	***If 'Heeft u moeite met op de tenen staan?' is equal to 'Ja' answer this question:***<br>Hoeveel moeite heeft u met op de tenen staan?	(0.00)	(100.00)
9.16	Heeft u moeite met opstaan uit de stoel?	◯ Ja<br>◯ Nee<br>◯ Niet van toepassing	
9.16.1	***If 'Heeft u moeite met opstaan uit de stoel?' is equal to 'Ja' answer this question:***<br>Hoeveel moeite heeft u met opstaan uit de stoel?	(0.00)	(100.00)
9.17	Heeft u moeite met het op- of afstappen van stoepranden?	◯ Ja<br>◯ Nee<br>◯ Niet van toepassing	
9.17.1	***If 'Heeft u moeite met het op- of afstappen van stoepranden?' is equal to 'Ja' answer this question:***<br>Hoeveel moeite heeft u met het op- of afstappen van stoepranden?	(0.00)	(100.00)
9.18	Heeft u moeite met snel lopen of rennen?	◯ Ja<br>◯ Nee<br>◯ Niet van toepassing	

Paget LDA, *et al. BMJ Open* 2019; 9:e030961. doi: 10.1136/bmjopen-2019-030961

9.18.1	***If 'Heeft u moeite met snel lopen of rennen?' is equal to 'Ja' answer this question:***<br>Hoeveel moeite heeft u met snel lopen of rennen?	(0.00)	(100.00)

Vragenlijst PRIMA studie 52 weken - FAOS

Number	Question	Answers
	Deze lijst vraagt naar uw mening over uw voet/enkel. Uw antwoorden geven ons een beeld van uw voet/enkel klachten en hoe u in staat bent om alledaagse activiteiten uit te voeren in uw huidige situatie. Beantwoorden van een vraag doet u door het aanklikken van een vakje met het volgens u meest juiste antwoord (één vakje per vraag). Als u niet zeker weet hoe u een vraag moet beantwoorden, geeft u dan het antwoord dat volgens u het meest op uw situatie van toepassing is. Deze vraag heeft betrekking op het voorkomen van voet/enkel klachten in de afgelopen week.	
10.1	Is uw voet/enkel gezwollen?	○ Nooit ○ Zelden ○ Soms ○ Vaak ○ Altijd
10.2	Voelt u gekraak of hoort u klikken of een ander vreemd geluid wanneer u de voet/enkel beweegt?	○ Nooit ○ Zelden ○ Soms ○ Vaak ○ Altijd
10.3	Hapert uw enkel of blokkeert uw enkel ('op slot' gaan zitten) wanneer u deze beweegt?	○ Nooit ○ Zelden ○ Soms ○ Vaak ○ Altijd
10.4	Kunt u de voet/enkel volledig strekken?	○ Altijd ○ Vaak ○ Soms ○ Zelden ○ Nooit
10.5	Kunt u de voet volledig naar u toe buigen?	○ Altijd ○ Vaak ○ Soms ○ Zelden ○ Nooit
10.6	In welke mate heeft u een stijf gevoel in de voet/enkel 's ochtends bij het wakker worden?	○ Niet ○ Mild ○ Matig ○ Ernstig ○ Zeer ernstig
10.7	In welke mate heeft u een stijf gevoel in de voet/enkel na zitten, liggen of rusten later op de dag?	○ Niet ○ Mild ○ Matig ○ Ernstig ○ Zeer ernstig
10.8	Hoe vaak heeft u pijn in uw voet/enkel?	○ Nooit ○ Maandelijks ○ Wekelijks ○ Dagelijks ○ Altijd

Paget LDA, *et al. BMJ Open* 2019; 9:e030961. doi: 10.1136/bmjopen-2019-030961

10.9	Hoeveel voet/enkel pijn heeft u gehad in de afgelopen week bij draaien als uw voet/enkel op de grond staat?	◯ Geen<br>◯ Mild<br>◯ Matig<br>◯ Ernstig<br>◯ Zeer ernstig
10.10	Hoeveel voet/enkel pijn heeft u gehad in de afgelopen week bij het volledig uitstrekken van de voet/enkel?	◯ Geen<br>◯ Mild<br>◯ Matig<br>◯ Ernstig<br>◯ Zeer ernstig
10.11	Hoeveel voet/enkel pijn heeft u gehad in de afgelopen week bij het volledig naar u toe buigen/optrekken van de voet/enkel	◯ Geen<br>◯ Mild<br>◯ Matig<br>◯ Ernstig<br>◯ Zeer ernstig
10.12	Hoeveel voet/enkel pijn heeft u gehad in de afgelopen week bij het lopen op een vlakke ondergrond	◯ Geen<br>◯ Mild<br>◯ Matig<br>◯ Ernstig<br>◯ Zeer ernstig
10.13	Hoeveel voet/enkel pijn heeft u gehad in de afgelopen week bij het trap op- en trap af lopen?	◯ Geen<br>◯ Mild<br>◯ Matig<br>◯ Ernstig<br>◯ Zeer ernstig
10.14	Hoeveel voet/enkel pijn heeft u gehad in de afgelopen week 's nachts in bed?	◯ Geen<br>◯ Mild<br>◯ Matig<br>◯ Ernstig<br>◯ Zeer ernstig
10.15	Hoeveel voet/enkel pijn heeft u gehad in de afgelopen week bij het zitten of liggen?	◯ Geen<br>◯ Mild<br>◯ Matig<br>◯ Ernstig<br>◯ Zeer ernstig

Paget LDA, *et al. BMJ Open* 2019; 9:e030961. doi: 10.1136/bmjopen-2019-030961

10.16	Hoeveel voet/enkel pijn heeft u gehad in de afgelopen week bij het rechtop staan?	◯ Geen<br>◯ Mild<br>◯ Matig<br>◯ Ernstig<br>◯ Zeer ernstig
10.17	In welke mate werd u gehinderd bij het trap aflopen?	◯ Niet ◯ Mild ◯ Matig ◯ Ernstig ◯ Zeer ernstig
10.18	In welke mate werd u gehinderd bij het trap op lopen?	◯ Niet ◯ Mild ◯ Matig ◯ Ernstig ◯ Zeer ernstig
10.19	In welke mate werd u gehinderd als u vanuit een zittende positie ging staan?	◯ Niet ◯ Mild ◯ Matig ◯ Ernstig ◯ Zeer ernstig
10.20	In welke mate werd u gehinderd bij het staan?	◯ Niet ◯ Mild ◯ Matig ◯ Ernstig ◯ Zeer ernstig
10.21	In welke mate werd u gehinderd bij het naar de grond buigen/iets oprapen?	◯ Niet ◯ Mild ◯ Matig ◯ Ernstig ◯ Zeer ernstig
10.22	In welke mate werd u gehinderd bij het lopen op een vlakke ondergrond?	◯ Niet ◯ Mild ◯ Matig ◯ Ernstig ◯ Zeer ernstig
10.23	In welke mate werd u gehinderd bij het in- en uit de auto stappen?	◯ Niet ◯ Mild ◯ Matig ◯ Ernstig ◯ Zeer ernstig
10.24	In welke mate werd u gehinderd bij het boodschappen doen?	◯ Niet ◯ Mild ◯ Matig ◯ Ernstig ◯ Zeer ernstig
10.25	In welke mate werd u gehinderd bij sokken/panty's aantrekken?	◯ Niet ◯ Mild ◯ Matig ◯ Ernstig ◯ Zeer ernstig
10.26	In welke mate werd u gehinderd bij het opstaan uit bed?	◯ Niet ◯ Mild ◯ Matig ◯ Ernstig ◯ Zeer ernstig
10.27	In welke mate werd u gehinderd bij het sokken uittrekken?	◯ Niet ◯ Mild ◯ Matig ◯ Ernstig ◯ Zeer ernstig
10.28	In welke mate werd u gehinderd bij het in bed liggen (omdraaien, lange tijd uw voet/enkel in dezelfde positie houden)?	◯ Niet ◯ Mild ◯ Matig ◯ Ernstig ◯ Zeer ernstig

Paget LDA, *et al. BMJ Open* 2019; 9:e030961. doi: 10.1136/bmjopen-2019-030961

10.29	In welke mate werd u gehinderd bij het in/uit bad stappen?	◯ Niet ◯ Mild ◯ Matig ◯ Ernstig ◯ Zeer ernstig
10.30	In welke mate werd u gehinderd bij zitten?	◯ Niet ◯ Mild ◯ Matig ◯ Ernstig ◯ Zeer ernstig
10.31	In welke mate werd u gehinderd bij het toilet op en af gaan?	◯ Niet ◯ Mild ◯ Matig ◯ Ernstig ◯ Zeer ernstig
10.32	In welke mate werd u gehinderd bij zwaar huishoudelijk werk (bijvoorbeeld zware dozen sjouwen, vloer schrobben)?	◯ Niet ◯ Mild ◯ Matig ◯ Ernstig ◯ Zeer ernstig
10.33	In welke mate werd u gehinderd bij licht huishoudeijk werk (bijvoorbeeld koken, afstoffen)?	◯ Niet ◯ Mild ◯ Matig ◯ Ernstig ◯ Zeer ernstig
10.34	In welke mate werd u gehinderd bij hurken?	◯ Niet ◯ Mild ◯ Matig ◯ Ernstig ◯ Zeer ernstig
10.35	In welke mate werd u gehinderd bij hardlopen?	◯ Niet ◯ Mild ◯ Matig ◯ Ernstig ◯ Zeer ernstig
10.36	In welke mate werd u gehinderd bij springen?	◯ Niet ◯ Mild ◯ Matig ◯ Ernstig ◯ Zeer ernstig
10.37	In welke mate werd u gehinderd bij ronddraaien op uw aangedane voet/enkel?	◯ Niet ◯ Mild ◯ Matig ◯ Ernstig ◯ Zeer ernstig
10.38	In welke mate werd u gehinderd bij knielen?	◯ Niet ◯ Mild ◯ Matig ◯ Ernstig ◯ Zeer ernstig
10.39	Hoe vaak bent u zich bewust van uw voet/enkel probleem?	◯ Nooit ◯ Maandelijks ◯ Wekelijks ◯ Dagelijks ◯ Altijd
10.40	Heeft u uw leven veranderd om activiteiten te vermijden die schadelijk kunnen zijn voor uw voet/enkel?	◯ Niet<br>◯ Enigszins<br>◯ Matig<br>◯ Behoorlijk<br>◯ Volledig

Paget LDA, *et al. BMJ Open* 2019; 9:e030961. doi: 10.1136/bmjopen-2019-030961

10.41	In hoeverre kunt u op uw voet/enkel vertrouwen?	◯ Volledig<br>◯ Behoorlijk<br>◯ Matig<br>◯ Enigzins<br>◯ Niet
10.42	In het algemeen, in welke mate ondervindt u hinder van uw voet/enkel	◯ Geen<br>◯ Mild<br>◯ Matig<br>◯ Ernstig<br>◯ Zeer ernstig

Vragenlijst PRIMA studie 52 weken - PRODISQ - Gezondheid en werk

Number	Question	Answers
11.1	Wat is uw leeftijd?	[ ] jaar
11.2	Wat is uw geslacht?	◯ Man<br>◯ Vrouw
11.3	Wat is de hoogste opleiding die u heeft afgemaakt (Zoek uw hoogste opleiding en kruis het hokje daarvoor aan)?	☐ Ik heb geen school of opleiding afgemaakt<br>☐ Lagere school of basisschool<br>☐ Huishoudschool, vbo, lbo, lts, leao of lhno<br>☐ Mavo, mulo, ivo of vmbo<br>☐ Mbo, mts, meao, mhno, inas of intas<br>☐ Havo, vwo, hbs, mms, atheneum of gymnasium<br>☐ Hbo, hts, heao of hhno<br>☐ Universiteit<br>☐ Ik heb een andere opleiding afgemaakt, namelijk
11.3.1	***If 'Wat is de hoogste opleiding die u heeft afgemaakt (Zoek uw hoogste opleiding en kruis het hokje daarvoor aan)?' is equal to 'Ik heb een andere opleiding afgemaakt, namelijk' answer this question:***<br>Wat is de hoogste opleiding die u heeft afgemaakt?	[ ]

Paget LDA, *et al. BMJ Open* 2019; 9:e030961. doi: 10.1136/bmjopen-2019-030961

11.4	Wat doet u in het dagelijks leven?	☐ Ik zit op school, ik studeer<br>☐ Ik werk in loondienst<br>☐ Ik ben zelfstandig ondernemer<br>☐ Ik ben huisvrouw, huisman<br>☐ Ik ben werkloos<br>☐ Ik ben arbeidsongeschikt<br>☐ Ik ben met pensioen of prepensioen<br>☐ Ik doe iets anders, namelijk
11.4.1	***If 'Wat doet u in het dagelijks leven?' is equal to 'Ik ben arbeidsongeschikt' answer this question:***<br>Wat doet u in het dagelijks leven?	
11.4.2	***If 'Wat doet u in het dagelijks leven?' is equal to 'Ik doe iets anders, namelijk' answer this question:***<br>Wat doet u in het dagelijks leven?	
11.5	Hebt u betaald werk?	○ Nee<br>○ Ja
11.5.1	***If 'Hebt u betaald werk?' is equal to 'Ja' answer this question:***<br>Wat is uw beroep?	
11.5.2	***If 'Hebt u betaald werk?' is equal to 'Ja' answer this question:***<br>Hoeveel uur per week werkt u (Tel alleen de uren waarvoor u betaald wordt)?	uren
11.5.3	***If 'Hebt u betaald werk?' is equal to 'Ja' answer this question:***<br>Hoeveel dagen in de week werkt u?	Dagen
11.5.4	***If 'Hebt u betaald werk?' is equal to 'Ja' answer this question:***<br>Bent u in de afgelopen 4 weken afwezig geweest van uw werk omdat u ziek was?	○ Nee<br>○ Ja
11.5.4.1	***If 'Bent u in de afgelopen 4 weken afwezig geweest van uw werk omdat u ziek was?' is equal to 'Ja' answer this question:***<br>Bent u in de afgelopen 4 weken afwezig geweest van uw werk omdat u ziek was (Tel alleen de werkdagen in de afgelopen 4 weken)?	dagen afwezig geweest
11.5.4.2	***If 'Bent u in de afgelopen 4 weken afwezig geweest van uw werk omdat u ziek was?' is equal to 'Ja' answer this question:***<br>Was u langer dan de gehele periode van 4 weken afwezig van uw werk doordat u ziek was?	○ Nee<br>○ Ja

Paget LDA, *et al. BMJ Open* 2019; 9:e030961. doi: 10.1136/bmjopen-2019-030961

11.5.4.2.1	***If 'Was u langer dan de gehele periode van 4 weken afwezig van uw werk doordat u ziek was?' is equal to 'Ja' answer this question:*** Wanneer heeft u zich ziek gemeld?	☐ ☐ ☐ *(dd-mm-yyyy)*
11.5.5	***If 'Hebt u betaald werk?' is equal to 'Ja' answer this question:*** Waren er in de afgelopen 4 weken, dagen waarop u wel gewerkt heeft, maar tijdens uw werk last had van lichamelijke of psychische problemen?	○ Ja<br>○ Nee
11.5.5.1	***If 'Waren er in de afgelopen 4 weken, dagen waarop u wel gewerkt heeft, maar tijdens uw werk last had van lichamelijke of psychische problemen?' is equal to 'Ja' answer this question:*** Op hoeveel werkdagen had u tijdens uw werk last van uw lichamelijke of psychische problemen?	☐ werkdagen
11.5.5.2	***If 'Waren er in de afgelopen 4 weken, dagen waarop u wel gewerkt heeft, maar tijdens uw werk last had van lichamelijke of psychische problemen?' is equal to 'Ja' answer this question:*** Op de dagen dat u last had, kon u misschien niet zoveel werk doen als normaal. Hoeveel werk kon u op deze dagen gemiddeld doen?	Ik kon op deze dagen niks doen (0.00) — Ik kon net zoveel doen als normaal (10.00)
11.6	Waren er dagen waarop u minder onbetaald werk kon doen door uw lichamelijke of psychische problemen?	○ Nee<br>○ Ja
11.6.1	***If 'Waren er dagen waarop u minder onbetaald werk kon doen door uw lichamelijke of psychische problemen?' is equal to 'Ja' answer this question:*** Op hoeveel dagen was dit zo?	☐ Dagen
11.6.2	***If 'Waren er dagen waarop u minder onbetaald werk kon doen door uw lichamelijke of psychische problemen?' is equal to 'Ja' answer this question:*** Stel dat iemand, bijvoorbeeld uw partner, familielid of een bekende, u op deze dagen had geholpen. En al het onbetaalde werk wat u niet kon doen, voor u had gedaan. Hoeveel uur was die persoon hier op deze dagen dan gemiddeld mee bezig geweest?	☐ uur op deze dagen

Vragenlijst PRIMA studie 52 weken - PRODISQ - Zorggebruik

Number	Question	Answers
	Wij willen graag weten met welke dokters u in de afgelopen 3 maanden een afspraak had. Het gaat om afspraken voor uzelf. Ook andere zorgverleners tellen mee. Bijvoorbeeld de fysiotherapeut.	

Paget LDA, *et al. BMJ Open* 2019; 9:e030961. doi: 10.1136/bmjopen-2019-030961

12.1	Bent u in de afgelopen 3 maanden naar uw huisarts geweest?	◯ Nee<br>◯ Ja
12.1.1	***If 'Bent u in de afgelopen 3 maanden naar uw huisarts geweest?' is equal to 'Ja' answer this question:***<br>Hoeveel afspraken had u?	[ ] afspraken
12.2	Bent u in de afgelopen 3 maanden in contact geweest met een maatschappelijk werker?	◯ Nee<br>◯ Ja
12.2.1	***If 'Bent u in de afgelopen 3 maanden in contact geweest met een maatschappelijk werker?' is equal to 'Ja' answer this question:***<br>Hoeveel afspraken had u?	[ ] afspraken
12.3	Bent u in de afgelopen 3 maanden naar een fysiotherapeut geweest? Of een caesartherapeut, therapeut mensendieck of een manueel therapeut?	◯ Nee<br>◯ Ja
12.3.1	***If 'Bent u in de afgelopen 3 maanden naar een fysiotherapeut geweest? Of een caesartherapeut, therapeut mensendieck of een manueel therapeut?' is equal to 'Ja' answer this question:***<br>Hoeveel afspraken had u?	[ ] afspraken
12.4	Bent u in de afgelopen 3 maanden naar een ergotherapeut geweest?	◯ Nee<br>◯ Ja
12.4.1	***If 'Bent u in de afgelopen 3 maanden naar een ergotherapeut geweest?' is equal to 'Ja' answer this question:***<br>Hoeveel afspraken had u?	[ ] afspraken
12.5	Bent u in de afgelopen 3 maanden naar een logopedist geweest?	◯ Nee<br>◯ Ja
12.5.1	***If 'Bent u in de afgelopen 3 maanden naar een logopedist geweest?' is equal to 'Ja' answer this question:***<br>Hoeveel afspraken had u?	[ ] afspraken
12.6	Bent u in de afgelopen 3 maanden naar een diëtist geweest?	◯ Nee<br>◯ Ja
12.6.1	***If 'Bent u in de afgelopen 3 maanden naar een diëtist geweest?' is equal to 'Ja' answer this question:***<br>Hoeveel afspraken had u?	[ ] afspraken

Paget LDA, *et al. BMJ Open* 2019; 9:e030961. doi: 10.1136/bmjopen-2019-030961

12.7	Bent u in de afgelopen 3 maanden naar een homeopaat geweest? Of een acupuncturist?	◯ Nee<br>◯ Ja
12.7.1	***If 'Bent u in de afgelopen 3 maanden naar een homeopaat geweest? Of een acupuncturist?' is equal to 'Ja' answer this question:***<br>Hoeveel afspraken had u?	[ ] afspraken
12.8	Bent u in de afgelopen 3 maanden naar een psycholoog geweest? Of een psychotherapeut of psychiater?	◯ Nee<br>◯ Ja
12.8.1	***If 'Bent u in de afgelopen 3 maanden naar een psycholoog geweest? Of een psychotherapeut of psychiater?' is equal to 'Ja' answer this question:***<br>Hoeveel afspraken had u?	[ ] afspraken
12.9	Heeft u in de afgelopen 3 maanden afspraken gehad met de bedrijfsarts?	◯ Nee<br>◯ Ja
12.9.1	***If 'Heeft u in de afgelopen 3 maanden afspraken gehad met de bedrijfsarts?' is equal to 'Ja' answer this question:***<br>Hoeveel afspraken had u?	[ ] afspraken
12.10	Heeft u in de afgelopen 3 maanden hulp van de thuiszorg gehad?	◯ Ja<br>◯ Nee
12.10.1	***If 'Heeft u in de afgelopen 3 maanden hulp van de thuiszorg gehad?' is equal to 'Ja' answer this question:***<br>Wat voor hulp van de thuiszorg heeft u gehad in de afgelopen 3 maanden?	☐ Huishoudelijke hulp; voorbeeld: stofzuigen, bed opmaken, boodschappen doen<br>☐ Verzorging van uzelf; voorbeeld: hulp bij douchen of aankleden<br>☐ Verpleging; voorbeeld: verband omdoen, medicijnen geven, bloeddruk meten
12.10.1.1	***If 'Wat voor hulp van de thuiszorg heeft u gehad in de afgelopen 3 maanden?' is equal to 'Huishoudelijke hulp; voorbeeld: stofzuigen, bed opmaken, boodschappen doen' answer this question:***<br>Hoeveel weken heeft u deze thuiszorg gehad? Huishoudelijke hulp:	[ ] weken in de afgelopen 3 maanden
12.10.1.2	***If 'Wat voor hulp van de thuiszorg heeft u gehad in de afgelopen 3 maanden?' is equal to 'Verzorging van uzelf; voorbeeld: hulp bij douchen of aankleden' answer this question:***<br>Hoeveel weken heeft u deze thuiszorg gehad? Verzorging van uzelf:	[ ] weken in de afgelopen 3 maanden

Paget LDA, *et al*. *BMJ Open* 2019; 9:e030961. doi: 10.1136/bmjopen-2019-030961

12.10.1.3	***If 'Wat voor hulp van de thuiszorg heeft u gehad in de afgelopen 3 maanden?' is equal to 'Verpleging; voorbeeld: verband omdoen, medicijnen geven, bloeddruk meten' answer this question:*** Hoeveel weken heeft u deze thuiszorg gehad? Verpleging:	[ ] weken in de afgelopen 3 maanden
12.10.1.4	***If 'Wat voor hulp van de thuiszorg heeft u gehad in de afgelopen 3 maanden?' is equal to 'Huishoudelijke hulp; voorbeeld: stofzuigen, bed opmaken, boodschappen doen' answer this question:*** Hoeveel uur thuiszorg kreeg u in deze weken gemiddeld? Huishoudelijke hulp: gemiddeld	[ ] uur in de week
12.10.1.5	***If 'Wat voor hulp van de thuiszorg heeft u gehad in de afgelopen 3 maanden?' is equal to 'Verzorging van uzelf; voorbeeld: hulp bij douchen of aankleden' answer this question:*** Hoeveel uur thuiszorg kreeg u in deze weken gemiddeld? Verzorging van uzelf: gemiddeld	[ ] uur in de week
12.10.1.6	***If 'Wat voor hulp van de thuiszorg heeft u gehad in de afgelopen 3 maanden?' is equal to 'Verpleging; voorbeeld: verband omdoen, medicijnen geven, bloeddruk meten' answer this question:*** Hoeveel uur thuiszorg kreeg u in deze weken gemiddeld? Verpleging: gemiddeld	[ ] uur in de week
12.11	Heeft u in de afgelopen 3 maanden medicijnen gebruikt?	○ Ja<br>○ Nee
12.11.1	***If 'Heeft u in de afgelopen 3 maanden medicijnen gebruikt?' is equal to 'Ja' answer this question:*** Welke medicijnen heeft u in de afgelopen 3 maanden gebruikt?	[ ]
12.12	Bent u in de afgelopen 3 maanden op de spoedeisende eerste hulp van een ziekenhuis geweest? (Een andere naam voor spoedeisende eerste hulp is EHBO)	○ Nee<br>○ Ja
12.12.1	***If 'Bent u in de afgelopen 3 maanden op de spoedeisende eerste hulp van een ziekenhuis geweest? (Een andere naam voor spoedeisende eerste hulp is EHBO)' is equal to 'Ja' answer this question:*** Hoevaak bent u geweest?	[ ] keer
12.13	Bent u in de afgelopen 3 maanden met een ambulance naar het ziekenhuis gebracht? (Een andere naam voor ambulance is ziekenauto)	○ Nee<br>○ Ja

Paget LDA, *et al. BMJ Open* 2019; 9:e030961. doi: 10.1136/bmjopen-2019-030961

12.13.1	***If 'Bent u in de afgelopen 3 maanden met een ambulance naar het ziekenhuis gebracht? (Een andere naam voor ambulance is ziekenauto)' is equal to 'Ja' answer this question:*** Hoe vaak bent u naar het ziekenhuis gebracht?	[ ] keer
12.14	Had u in de afgelopen 3 maanden een afspraak bij de polikliniek van het ziekenhuis? (Het gaat om afspraken voor uzelf met een dokter. Bijvoorbeeld met de cardioloog, reumatoloog of neuroloog)	◯ Ja<br>◯ Nee
12.14.1	***If 'Had u in de afgelopen 3 maanden een afspraak bij de polikliniek van het ziekenhuis? (Het gaat om afspraken voor uzelf met een dokter. Bijvoorbeeld met de cardioloog, reumatoloog of neuroloog)' is equal to 'Ja' answer this question:*** Bij welke soorten dokters bent u in de afgelopen 3 maanden in het ziekenhuis geweest? En hoe vaak?	[ ]
12.15	Bent u in de afgelopen 3 maanden overdag in het ziekenhuis geweest voor een behandeling? (U bleef dus niet slapen. U kwam bijvoorbeeld voor een bloedtransfusie, nierdialyse of chemokuur)	◯ Ja<br>◯ Nee
12.15.1	***If 'Bent u in de afgelopen 3 maanden overdag in het ziekenhuis geweest voor een behandeling? (U bleef dus niet slapen. U kwam bijvoorbeeld voor een bloedtransfusie, nierdialyse of chemokuur)' is equal to 'Ja' answer this question:*** Voor welke soort behandeling was dit?	[ ]
12.15.2	***If 'Bent u in de afgelopen 3 maanden overdag in het ziekenhuis geweest voor een behandeling? (U bleef dus niet slapen. U kwam bijvoorbeeld voor een bloedtransfusie, nierdialyse of chemokuur)' is equal to 'Ja' answer this question:*** Hoeveel keer moest u in de afgelopen 3 maanden voor deze behandelingen naar het ziekenhuis?	[ ]
12.16	Bent u in de afgelopen 3 maanden ergens anders geweest voor een behandeling overdag? (U bleef dus niet slapen. U ging bijvoorbeeld naar de dagopvang van een woon-/zorgcentrum of een psychiatrische instelling. Of naar de dagbehandeling van een revalidatiecentrum)	◯ Ja<br>◯ Nee
12.16.1	***If 'Bent u in de afgelopen 3 maanden ergens anders geweest voor een behandeling overdag? (U bleef dus niet slapen. U ging bijvoorbeeld naar de dagopvang van een woon-/zorgcentrum of een psychiatrische instelling. Of naar de dagbehandeling van een revalidatiecentrum)' is equal to 'Ja' answer this question:*** Wat voor instelling was dit?	☐ Woon-/zorgcentrum<br>☐ Revalidatiecentrum<br>☐ Psychiatrische instelling<br>☐ Een andere instelling, namelijk

Paget LDA, *et al. BMJ Open* 2019; 9:e030961. doi: 10.1136/bmjopen-2019-030961

12.16.1.1	***If 'Wat voor instelling was dit?' is equal to 'Een andere instelling, namelijk' answer this question:*** Wat voor instelling was dit?	
12.16.1.2	***If 'Wat voor instelling was dit?' is equal to 'Woon-/zorgcentrum' answer this question:*** Hoe vaak moest u hier in de afgelopen 3 maanden naartoe? Naar het woon-/zorgcentrum:	keer in de afgelopen 3 maanden
12.16.1.3	***If 'Wat voor instelling was dit?' is equal to 'Revalidatiecentrum' answer this question:*** Hoe vaak moest u hier in de afgelopen 3 maanden naartoe? Naar het revalidatiecentrum:	keer in de afgelopen 3 maanden
12.16.1.4	***If 'Wat voor instelling was dit?' is equal to 'Psychiatrische instelling' answer this question:*** Hoe vaak moest u hier in de afgelopen 3 maanden naartoe? Naar de psychiatrische instelling:	keer in de afgelopen 3 maanden
12.16.1.5	***If 'Wat voor instelling was dit?' is equal to 'Een andere instelling, namelijk' answer this question:*** Hoe vaak moest u hier in de afgelopen 3 maanden naartoe? Naar de andere instelling:	keer in de afgelopen 3 maanden
12.17	Heeft u in de afgelopen 3 maanden weleens in het ziekenhuis gelegen? (U moest dus blijven slapen. Bijvoorbeeld omdat u geopereerd was en niet meteen naar huis kon)	○ Ja<br>○ Nee
12.17.1	***If 'Heeft u in de afgelopen 3 maanden weleens in het ziekenhuis gelegen? (U moest dus blijven slapen. Bijvoorbeeld omdat u geopereerd was en niet meteen naar huis kon)' is equal to 'Ja' answer this question:*** Hoe vaak heeft u in de afgelopen 3 maanden in het ziekenhuis gelegen?	keer in de afgelopen 3 maanden
12.17.2	***If 'Heeft u in de afgelopen 3 maanden weleens in het ziekenhuis gelegen? (U moest dus blijven slapen. Bijvoorbeeld omdat u geopereerd was en niet meteen naar huis kon)' is equal to 'Ja' answer this question:*** Hoe lang heeft u in het ziekenhuis gelegen?	dagen in de afgelopen 3 maanden
12.18	Moest u in de afgelopen 3 maanden ergens anders blijven slapen voor uw gezondheid? (Bijvoorbeeld in een woon-/zorgcentrum, psychiatrische instelling of revalidatiecentrum)	○ Ja<br>○ Nee

Paget LDA, *et al*. *BMJ Open* 2019; 9:e030961. doi: 10.1136/bmjopen-2019-030961

12.18.1	***If 'Moest u in de afgelopen 3 maanden ergens anders blijven slapen voor uw gezondheid? (Bijvoorbeeld in een woon-/zorgcentrum, psychiatrische instelling of revalidatiecentrum)' is equal to 'Ja' answer this question:***<br>Wat voor instelling was dit?	☐ Woon-/zorgcentrum<br>☐ Revalidatiecentrum<br>☐ Psychiatrische instelling<br>☐ Een andere instelling, namelijk
12.18.1.1	***If 'Wat voor instelling was dit?' is equal to 'Een andere instelling, namelijk' answer this question:***<br>Een andere instelling, namelijk	
12.18.1.2	***If 'Wat voor instelling was dit?' is equal to 'Woon-/zorgcentrum' answer this question:***<br>Hoe lang bent u in deze instelling geweest? In het woon-/zorgcentrum:	____ dagen in de afgelopen 3 maanden
12.18.1.3	***If 'Wat voor instelling was dit?' is equal to 'Revalidatiecentrum' answer this question:***<br>Hoe lang bent u in deze instelling geweest? In het revalidatiecentrum:	____ dagen in de afgelopen 3 maanden
12.18.1.4	***If 'Wat voor instelling was dit?' is equal to 'Psychiatrische instelling' answer this question:***<br>Hoe lang bent u in deze instelling geweest? In de psychiatrische instelling:	____ dagen in de afgelopen 3 maanden
12.18.1.5	***If 'Wat voor instelling was dit?' is equal to 'Een andere instelling, namelijk' answer this question:***<br>Hoe lang bent u in deze instelling geweest? In de andere instelling:	____ dagen in de afgelopen 3 maanden
12.19	Heeft u in de afgelopen 3 maanden hulp gekregen van een familielid of een bekende vanwege uw lichamelijke of psychische problemen?	◯ Ja<br>◯ Nee
12.19.1	***If 'Heeft u in de afgelopen 3 maanden hulp gekregen van een familielid of een bekende vanwege uw lichamelijke of psychische problemen?' is equal to 'Ja' answer this question:***<br>Wat voor hulp van familieleden of bekenden heeft u gehad in de afgelopen 3 maanden?	☐ Huishoudelijke hulp - voorbeeld: stofzuigen, bed opmaken, boodschappen doen, klaarmaken van eten en drinken, verzorgen van kinderen<br>☐ Verzorging van uzelf - voorbeeld: hulp bij douchen of aankleden, hulp bij het eten en drinken of het geven van medicijnen<br>☐ Praktische hulp - voorbeeld: ondersteuning bij wandelen, het maken van uitstapjes of bezoekjes aan bekenden, bezoeken aan de huisarts of het ziekenhuis, het regelen van hulp of het regelen van financiële zaken

Paget LDA, *et al*. *BMJ Open* 2019; 9:e030961. doi: 10.1136/bmjopen-2019-030961

12.19.1.1	***If 'Wat voor hulp van familieleden of bekenden heeft u gehad in de afgelopen 3 maanden?' is equal to 'Huishoudelijke hulp - voorbeeld: stofzuigen, bed opmaken, boodschappen doen, klaarmaken van eten en drinken, verzorgen van kinderen' answer this question:*** Hoeveel weken heeft u deze hulp gehad? Huishoudelijke hulp:	[ ] weken in de afgelopen 3 maanden
12.19.1.2	***If 'Wat voor hulp van familieleden of bekenden heeft u gehad in de afgelopen 3 maanden?' is equal to 'Verzorging van uzelf - voorbeeld: hulp bij douchen of aankleden, hulp bij het eten en drinken of het geven van medicijnen' answer this question:*** Hoeveel weken heeft u deze hulp gehad? Verzorging van uzelf:	[ ] weken in de afgelopen 3 maanden
12.19.1.3	***If 'Wat voor hulp van familieleden of bekenden heeft u gehad in de afgelopen 3 maanden?' is equal to 'Praktische hulp - voorbeeld: ondersteuning bij wandelen, het maken van uitstapjes of bezoekjes aan bekenden, bezoeken aan de huisarts of het ziekenhuis, het regelen van hulp of het regelen van financiële zaken' answer this question:*** Hoeveel uur hulp kreeg u in deze weken gemiddeld? Praktische hulp:	[ ] weken in de afgelopen 3 maanden
12.19.1.4	***If 'Wat voor hulp van familieleden of bekenden heeft u gehad in de afgelopen 3 maanden?' is equal to 'Huishoudelijke hulp - voorbeeld: stofzuigen, bed opmaken, boodschappen doen, klaarmaken van eten en drinken, verzorgen van kinderen' answer this question:*** Hoeveel uur hulp kreeg u in deze weken gemiddeld? Huishoudelijke hulp: gemiddeld	[ ] uur in de week
12.19.1.5	***If 'Wat voor hulp van familieleden of bekenden heeft u gehad in de afgelopen 3 maanden?' is equal to 'Verzorging van uzelf - voorbeeld: hulp bij douchen of aankleden, hulp bij het eten en drinken of het geven van medicijnen' answer this question:*** Hoeveel uur hulp kreeg u in deze weken gemiddeld? Verzorging van uzelf: gemiddeld	[ ] uur in de week
12.19.1.6	***If 'Wat voor hulp van familieleden of bekenden heeft u gehad in de afgelopen 3 maanden?' is equal to 'Praktische hulp - voorbeeld: ondersteuning bij wandelen, het maken van uitstapjes of bezoekjes aan bekenden, bezoeken aan de huisarts of het ziekenhuis, het regelen van hulp of het regelen van financiële zaken' answer this question:*** Hoeveel uur hulp kreeg u in deze weken gemiddeld? Praktische hulp: gemiddeld	[ ] uur in de week

Paget LDA, *et al*. *BMJ Open* 2019; 9:e030961. doi: 10.1136/bmjopen-2019-030961

Vragenlijst PRIMA studie 52 weken - Dank

Number	Question	Answers
13.1	Hartelijk dank voor de tijd die u heeft genomen om de vragenlijsten in te vullen. Indien u nog opmerkingen heeft kunt u deze hierin plaatsen.	

Survey 'Vragenlijst PRIMA studie 6 weken'

Vragenlijst PRIMA studie 6 weken - AOFAS - Pijn

Number	Question	Answers
1.1	Hoeveel pijn ervaart u aan uw voet?	☐ Geen<br>☐ Milde pijnklachten of af en toe pijn<br>☐ Matige pijnklachten, dagelijks<br>☐ Vrijwel continu erge pijn

Vragenlijst PRIMA studie 6 weken - AOFAS resterende gedeelte

Number	Question	Answers
2.1	Hoeveel beperkingen in het dagelijks leven ervaart u?	○ Geen beperkingen, geen hulpmiddelen nodig<br>○ Geen beperkingen in algemeen dagelijkse bezigheden, wel beperkingen in recreatieve activiteiten (sport, hobbies, etc) geen ondersteuning<br>○ Beperkingen bij dagelijkse- en recreatieve activiteiten, stok<br>○ Ernstige beperkingen bij dagelijkse- en recreatieactiviteiten, walker, krukken, rolstoel, brace

Paget LDA, *et al. BMJ Open* 2019; 9:e030961. doi: 10.1136/bmjopen-2019-030961

2.2	Wat is de afstand die u in één keer kunt lopen?	◯ Meer dan 600 meter<br>◯ 400 - 600 meter<br>◯ 100 - 300 meter<br>◯ Minder dan 100 meter
2.3	Heeft u moeite met lopen op een bepaalde ondergrond?	◯ Geen problemen bij lopen<br>◯ Enig problemen op ongelijke ondergrond, trap, helling, ladders<br>◯ Ernstige problemen bij ongelijke ondergrond, trap, helling, ladders
2.4	Hoe zou u uw looppatroon beoordelen?	◯ Normaal of minimaal afwijkend<br>◯ Duidelijk<br>◯ Ernstig
2.5	Hoe zou u de beweeglijkheid van uw voet beoordelen ten opzicht van de andere zijde of ten opzicte van vóór het ongeval?	◯ Hetzelfde of licht beperkt t.o.v. de niet aangedane zijde of zoals voor het ongeval<br>◯ Ongeveer de helft beperkt<br>◯ Sterk verminderd, bijna geen bewegingen mogelijk
2.6	Hoe zou u de beweeglijkheid van uw enkel beoordelen ten opzichte van de andere zijde of ten opzichte van voor het ongeval?	◯ Hetzelfde of licht beperkt t.o.v. de niet aangedane zijde of zoals voor het ongeval<br>◯ Ongeveer de helft beperkt<br>◯ Sterk verminderd, bijna geen bewegingen mogelijk
2.7	Hoe zou u de stabiliteit van uw voet en enkel beoordelen	◯ Stabiel, ik verzwik mijn enkel hooguit incidenteel<br>◯ Niet stabiel, ik verzwik mijn enkel vaak
2.8	Hoe zou u de stand van uw voet beoordelen ten opzichte van de andere zijde of ten opzichte van voor het ongeval?	◯ Goed, ik kan mijn voet goed plat op de grond zetten, waarbij de stand niet afwijkend is<br>◯ Ik sta vrijwel alleen op de buitenzijde van mijn voet<br>◯ Ik kan mijn voet niet plat op de grond zetten en loop vrijwel alleen op mijn voorvoet

Vragenlijst PRIMA studie 6 weken - VAS Pain score

Paget LDA, *et al*. *BMJ Open* 2019; 9:e030961. doi: 10.1136/bmjopen-2019-030961

Number	Question	Answers	
3.1	VAS pijnscore (Geef uw minimaal pijnscore aan tijdens dagelijkse activiteiten op een schaal van 0 tot 100, waarbij 0 geen pijn is en 100 de ergste pijn denkbaar is)	(0.00)	100 (100.00)

Vragenlijst PRIMA studie 6 weken - AAS

Number	Question	Answers

Paget LDA, *et al*. *BMJ Open* 2019; 9:e030961. doi: 10.1136/bmjopen-2019-030961

4.1	Wat is het meest bij u van toepassing ten aanzien van uw acitviteit?	☐ American Football<br>☐ Basketbal<br>☐ Gymnastiek/turnen<br>☐ Handbal<br>☐ Rugby<br>☐ Voetbal<br>☐ Hockey<br>☐ Korfbal<br>☐ Gevechtssporten: judo, karate, kung fu, taekwando, jiu jitsu, etc.<br>☐ Oriëntatielopen<br>☐ Rhytmische gymnastiek<br>☐ Volleybal<br>☐ Boxen<br>☐ Freestyle snowboarden<br>☐ Ijshockey<br>☐ Tennis<br>☐ Worstelen<br>☐ Fitness, aerobics<br>☐ Badminton<br>☐ Baseball<br>☐ Cross-country hardlopen<br>☐ Moderne pentathlon<br>☐ Squash<br>☐ Surfen, windsurfen<br>☐ Tafel tennis<br>☐ Atletiek: spring-, werponderdelen<br>☐ Waterskieën<br>☐ Dans<br>☐ Schermen<br>☐ Zaalhockey<br>☐ Bergbeklimmen<br>☐ Langlauf<br>☐ Parachute springen<br>☐ Softball<br>☐ Speciale beroepen en werkactiviteiten; speciale beroepen: ballet, professioneel soldaat, speciale reddingswerker, stuntman, etc.<br>☐ Duiken<br>☐ Scubaduiken<br>☐ Skaten, in-line skaten<br>☐ Atletiek: looponderdelen<br>☐ Triatlon<br>☐ Gewichtsheffen, body-building

		☐ Alle competitieve sporten hieronder met 'seasonal' conditioning<br>☐ Zwaar fysiek werk<br>☐ Alpine skiën en snowboarden<br>☐ Bowlen/curlen<br>☐ Golf<br>☐ Mountainbike/BMX<br>☐ Powerliften<br>☐ Zeilen<br>☐ Fysiek werk<br>☐ Wielrennen<br>☐ Paardrijden<br>☐ Motorsporten/technische sporten<br>☐ Roeien, kayakken<br>☐ Boogschieten<br>☐ Water polo en zwemmen<br>☐ Kunnen lopen op oneven grond<br>☐ Geen sport, echter geen limitatie in dagelijkse activiteiten<br>☐ Kunnen lopen op even grond, maar de dagelijkste activiteiten zijn gelimiteerd<br>☐ Niet kunnen lopen, fysieke handicap wegens enkelproblemen
4.1.1	***If 'Wat is het meest bij u van toepassing ten aanzien van uw acitviteit?' is equal to 'American Football' answer this question:***<br>American Football	○ Professioneel<br>○ Competitief<br>○ Recreatief
4.1.2	***If 'Wat is het meest bij u van toepassing ten aanzien van uw acitviteit?' is equal to 'Basketbal' answer this question:***<br>Basketbal	○ Professioneel<br>○ Competitief<br>○ Recreatief
4.1.3	***If 'Wat is het meest bij u van toepassing ten aanzien van uw acitviteit?' is equal to 'Gymnastiek/turnen' answer this question:***<br>Gymnastiek/turnen	○ Professioneel<br>○ Competitief<br>○ Recreatief
4.1.4	***If 'Wat is het meest bij u van toepassing ten aanzien van uw acitviteit?' is equal to 'Handbal' answer this question:***<br>Handbal	○ Professioneel<br>○ Competitief<br>○ Recreatief
4.1.5	***If 'Wat is het meest bij u van toepassing ten aanzien van uw acitviteit?' is equal to 'Rugby' answer this question:***<br>Rugby	○ Professioneel<br>○ Competitief<br>○ Recreatief

Paget LDA, *et al. BMJ Open* 2019; 9:e030961. doi: 10.1136/bmjopen-2019-030961

4.1.6	***If 'Wat is het meest bij u van toepassing ten aanzien van uw acitviteit?' is equal to 'Voetbal' answer this question:***<br>Voetbal	◯ Professioneel<br>◯ Competitief<br>◯ Recreatief
4.1.7	***If 'Wat is het meest bij u van toepassing ten aanzien van uw acitviteit?' is equal to 'Hockey' answer this question:***<br>Hockey	◯ Professioneel<br>◯ Competitief<br>◯ Recreatief
4.1.8	***If 'Wat is het meest bij u van toepassing ten aanzien van uw acitviteit?' is equal to 'Korfbal' answer this question:***<br>Korfbal	◯ Professioneel<br>◯ Competitief<br>◯ Recreatief
4.1.9	***If 'Wat is het meest bij u van toepassing ten aanzien van uw acitviteit?' is equal to 'Gevechtssporten: judo, karate, kung fu, taekwando, jiu jitsu, etc.' answer this question:***<br>Gevechtssporten: judo, karate, kung fu, taekwando, jiu jitsu, etc.	◯ Professioneel<br>◯ Competitief<br>◯ Recreatief
4.1.10	***If 'Wat is het meest bij u van toepassing ten aanzien van uw acitviteit?' is equal to 'Oriëntatielopen' answer this question:***<br>Oriëntatielopen	◯ Professioneel<br>◯ Competitief<br>◯ Recreatief
4.1.11	***If 'Wat is het meest bij u van toepassing ten aanzien van uw acitviteit?' is equal to 'Rhytmische gymnastiek' answer this question:***<br>Rhytmisch gymnastiek	◯ Professioneel<br>◯ Competitief<br>◯ Recreatief
4.1.12	***If 'Wat is het meest bij u van toepassing ten aanzien van uw acitviteit?' is equal to 'Volleybal' answer this question:***<br>Volleybal	◯ Professioneel<br>◯ Competitief<br>◯ Recreatief
4.1.13	***If 'Wat is het meest bij u van toepassing ten aanzien van uw acitviteit?' is equal to 'Boxen' answer this question:***<br>Boxen	◯ Professioneel<br>◯ Competitief<br>◯ Recreatief
4.1.14	***If 'Wat is het meest bij u van toepassing ten aanzien van uw acitviteit?' is equal to 'Freestyle snowboarden' answer this question:***<br>Freestyle snowboarden	◯ Professioneel<br>◯ Competitief<br>◯ Recreatief
4.1.15	***If 'Wat is het meest bij u van toepassing ten aanzien van uw acitviteit?' is equal to 'Freestyle snowboarden' answer this question:***<br>IJshockey	◯ Professioneel<br>◯ Competitief<br>◯ Recreatief

Paget LDA, *et al. BMJ Open* 2019; 9:e030961. doi: 10.1136/bmjopen-2019-030961

4.1.16	***If 'Wat is het meest bij u van toepassing ten aanzien van uw acitviteit?' is equal to 'Tennis' answer this question:***<br>Tennis	◯ Professioneel<br>◯ Competitief<br>◯ Recreatief
4.1.17	***If 'Wat is het meest bij u van toepassing ten aanzien van uw acitviteit?' is equal to 'Worstelen' answer this question:***<br>Worstelen	◯ Professioneel<br>◯ Competitief<br>◯ Recreatief
4.1.18	***If 'Wat is het meest bij u van toepassing ten aanzien van uw acitviteit?' is equal to 'Fitness, aerobics' answer this question:***<br>Fitness, aerobics	◯ Professioneel<br>◯ Competitief<br>◯ Recreatief
4.1.19	***If 'Wat is het meest bij u van toepassing ten aanzien van uw acitviteit?' is equal to 'Badminton' answer this question:***<br>Badminton	◯ Professioneel<br>◯ Competitief<br>◯ Recreatief
4.1.20	***If 'Wat is het meest bij u van toepassing ten aanzien van uw acitviteit?' is equal to 'Baseball' answer this question:***<br>Baseball	◯ Professioneel<br>◯ Competitief<br>◯ Recreatief
4.1.21	***If 'Wat is het meest bij u van toepassing ten aanzien van uw acitviteit?' is equal to 'Cross-country hardlopen' answer this question:***<br>Cross-country hardlopen	◯ Professioneel<br>◯ Competitief<br>◯ Recreatief
4.1.22	***If 'Wat is het meest bij u van toepassing ten aanzien van uw acitviteit?' is equal to 'Moderne pentathlon' answer this question:***<br>Moderne pentathlon	◯ Professioneel<br>◯ Competitief<br>◯ Recreatief
4.1.23	***If 'Wat is het meest bij u van toepassing ten aanzien van uw acitviteit?' is equal to 'Squash' answer this question:***<br>Squash	◯ Professioneel<br>◯ Competitief<br>◯ Recreatief
4.1.24	***If 'Wat is het meest bij u van toepassing ten aanzien van uw acitviteit?' is equal to 'Surfen, windsurfen' answer this question:***<br>Surfen, windsurfen	◯ Professioneel<br>◯ Competitief<br>◯ Recreatief
4.1.25	***If 'Wat is het meest bij u van toepassing ten aanzien van uw acitviteit?' is equal to 'Tafel tennis' answer this question:***<br>Tafel tennis	◯ Professioneel<br>◯ Competitief<br>◯ Recreatief

Paget LDA, *et al. BMJ Open* 2019; 9:e030961. doi: 10.1136/bmjopen-2019-030961

4.1.26	***If 'Wat is het meest bij u van toepassing ten aanzien van uw acitviteit?' is equal to 'Atletiek: spring-, werponderdelen' answer this question:***<br>Atletiek: spring-, werponderdelen	◯ Professioneel<br>◯ Competitief<br>◯ Recreatief
4.1.27	***If 'Wat is het meest bij u van toepassing ten aanzien van uw acitviteit?' is equal to 'Waterskieën' answer this question:***<br>Waterskien	◯ Professioneel<br>◯ Competitief<br>◯ Recreatief
4.1.28	***If 'Wat is het meest bij u van toepassing ten aanzien van uw acitviteit?' is equal to 'Dans' answer this question:***<br>Dans	◯ Professioneel<br>◯ Competitief<br>◯ Recreatief
4.1.29	***If 'Wat is het meest bij u van toepassing ten aanzien van uw acitviteit?' is equal to 'Schermen' answer this question:***<br>Schermen	◯ Professioneel<br>◯ Competitief<br>◯ Recreatief
4.1.30	***If 'Wat is het meest bij u van toepassing ten aanzien van uw acitviteit?' is equal to 'Zaalhockey' answer this question:***<br>Zaalhockey	◯ Professioneel<br>◯ Competitief<br>◯ Recreatief
4.1.31	***If 'Wat is het meest bij u van toepassing ten aanzien van uw acitviteit?' is equal to 'Bergbeklimmen' answer this question:***<br>Bergbeklimmen	◯ Professioneel<br>◯ Competitief<br>◯ Recreatief
4.1.32	***If 'Wat is het meest bij u van toepassing ten aanzien van uw acitviteit?' is equal to 'Langlauf' answer this question:***<br>Langlauf	◯ Professioneel<br>◯ Competitief<br>◯ Recreatief
4.1.33	***If 'Wat is het meest bij u van toepassing ten aanzien van uw acitviteit?' is equal to 'Parachute springen' answer this question:***<br>Parachute springen	◯ Professioneel<br>◯ Competitief<br>◯ Recreatief
4.1.34	***If 'Wat is het meest bij u van toepassing ten aanzien van uw acitviteit?' is equal to 'Softball' answer this question:***<br>Softball	◯ Professioneel<br>◯ Competitief<br>◯ Recreatief

Paget LDA, *et al. BMJ Open* 2019; 9:e030961. doi: 10.1136/bmjopen-2019-030961

4.1.35	***If 'Wat is het meest bij u van toepassing ten aanzien van uw acitviteit?' is equal to 'Speciale beroepen en werkactiviteiten; speciale beroepen: ballet, professioneel soldaat, speciale reddingswerker, stuntman, etc.' answer this question:***<br>Speciale beroepen en werkactiviteiten speciale beroepen: ballet, professioneel soldaat, speciale reddingswerker, stuntman, etc.	○ ballet, professioneel soldaat, speciale reddingswerker, stuntman, etc.
4.1.36	***If 'Wat is het meest bij u van toepassing ten aanzien van uw acitviteit?' is equal to 'Duiken' answer this question:***<br>Duiken	○ Professioneel/competitief<br>○ Recreatief
4.1.37	***If 'Wat is het meest bij u van toepassing ten aanzien van uw acitviteit?' is equal to 'Scubaduiken' answer this question:***<br>Scubaduiken	○ Professioneel/competitief<br>○ Recreatief
4.1.38	***If 'Wat is het meest bij u van toepassing ten aanzien van uw acitviteit?' is equal to 'Skaten, in-line skaten' answer this question:***<br>skaten, in-linen skaten	○ Professioneel/competitief<br>○ Recreatief
4.1.39	***If 'Wat is het meest bij u van toepassing ten aanzien van uw acitviteit?' is equal to 'Atletiek: looponderdelen' answer this question:***<br>Atletiek: looponderdelen	○ Professioneel/competitief<br>○ Recreatief
4.1.40	***If 'Wat is het meest bij u van toepassing ten aanzien van uw acitviteit?' is equal to 'Triatlon' answer this question:***<br>triatlon	○ Professioneel/competitief<br>○ Recreatief
4.1.41	***If 'Wat is het meest bij u van toepassing ten aanzien van uw acitviteit?' is equal to 'Gewichtsheffen, body-building' answer this question:***<br>Gewichtsheffen, body-building	○ Professioneel/competitief<br>○ Recreatief
4.1.42	***If 'Wat is het meest bij u van toepassing ten aanzien van uw acitviteit?' is equal to 'Alle competitieve sporten hieronder met ‘seasonal’ conditioning' answer this question:***<br>Alle competitieve sporten hieronder met ‘seasonal’ conditioning	○ Alle competitieve sporten hieronder met ‘seasonal’ conditioning
4.1.43	***If 'Wat is het meest bij u van toepassing ten aanzien van uw acitviteit?' is equal to 'Zwaar fysiek werk' answer this question:***<br>Zwaar fysiek werk	○ Alle competitieve sporten hieronder met ‘seasonal’ conditioning

Paget LDA, *et al. BMJ Open* 2019; 9:e030961. doi: 10.1136/bmjopen-2019-030961

4.1.44	***If 'Wat is het meest bij u van toepassing ten aanzien van uw acitviteit?' is equal to 'Alpine skiën en snowboarden' answer this question:***<br>Alpine skien en snowboarden	◯ Alpine skien en snowboarden
4.1.45	***If 'Wat is het meest bij u van toepassing ten aanzien van uw acitviteit?' is equal to 'Bowlen/curlen' answer this question:***<br>Bowlen/curlen	◯ Bowlen/curlen
4.1.46	***If 'Wat is het meest bij u van toepassing ten aanzien van uw acitviteit?' is equal to 'Golf' answer this question:***<br>Golf	◯ Golf
4.1.47	***If 'Wat is het meest bij u van toepassing ten aanzien van uw acitviteit?' is equal to 'Mountainbike/BMX' answer this question:***<br>Mountainbike/BMX	◯ Mountainbike/BMX
4.1.48	***If 'Wat is het meest bij u van toepassing ten aanzien van uw acitviteit?' is equal to 'Powerliften' answer this question:***<br>Powerliften	◯ Powerliften
4.1.49	***If 'Wat is het meest bij u van toepassing ten aanzien van uw acitviteit?' is equal to 'Zeilen' answer this question:***<br>Zeilen	◯ Zeilen
4.1.50	***If 'Wat is het meest bij u van toepassing ten aanzien van uw acitviteit?' is equal to 'Fysiek werk' answer this question:***<br>Fysiek werk	◯ Fysiek werk
4.1.51	***If 'Wat is het meest bij u van toepassing ten aanzien van uw acitviteit?' is equal to 'Wielrennen' answer this question:***<br>Wielrennen	◯ Wielrennen
4.1.52	***If 'Wat is het meest bij u van toepassing ten aanzien van uw acitviteit?' is equal to 'Paardrijden' answer this question:***<br>Paardrijden	◯ Paardrijden
4.1.53	***If 'Wat is het meest bij u van toepassing ten aanzien van uw acitviteit?' is equal to 'Motorsporten/technische sporten' answer this question:***<br>Motorsporten/technische sporten	◯ Motorsporten/technische sporten
4.1.54	***If 'Wat is het meest bij u van toepassing ten aanzien van uw acitviteit?' is equal to 'Roeien, kayakken' answer this question:***<br>Roeien, kayakken	◯ Roeien, kayakken

Paget LDA, *et al. BMJ Open* 2019; 9:e030961. doi: 10.1136/bmjopen-2019-030961

4.1.55	***If 'Wat is het meest bij u van toepassing ten aanzien van uw acitviteit?' is equal to 'Boogschieten' answer this question:*** Boogschieten	◯ Boogschieten
4.1.56	***If 'Wat is het meest bij u van toepassing ten aanzien van uw acitviteit?' is equal to 'Water polo en zwemmen' answer this question:*** Water polo en zwemmen	◯ Water polo en zwemmen
4.1.57	***If 'Wat is het meest bij u van toepassing ten aanzien van uw acitviteit?' is equal to 'Kunnen lopen op oneven grond' answer this question:*** Kunnen lopen op oneven grond	◯ Kunnen lopen op oneven grond
4.1.58	***If 'Wat is het meest bij u van toepassing ten aanzien van uw acitviteit?' is equal to 'Geen sport, echter geen limitatie in dagelijkse activiteiten' answer this question:*** Geen sport, echter geen limitatie in dagelijkse activiteiten	◯ Geen sport, echter geen limitatie in dagelijkse activiteiten
4.1.59	***If 'Wat is het meest bij u van toepassing ten aanzien van uw acitviteit?' is equal to 'Kunnen lopen op even grond, maar de dagelijkste activiteiten zijn gelimiteerd' answer this question:*** Kunnen lopen op even grond, maar de dagelijkste activiteiten zijn gelimiteerd	◯ Kunnen lopen op even grond, maar de dagelijkste activiteiten zijn gelimiteerd
4.1.60	***If 'Wat is het meest bij u van toepassing ten aanzien van uw acitviteit?' is equal to 'Niet kunnen lopen, fysieke handicap wegens enkelproblemen' answer this question:*** Niet kunnen lopen, fysieke handicap wegens enkelproblemen	◯ Niet kunnen lopen, fysieke handicap wegens enkelproblemen

Vragenlijst PRIMA studie 6 weken - Hoe tevreden bent u over de enkelklachten?

Number	Question	Answers
5.1	Hoe tevreden bent u over de enkelklachten?	◯ Slecht<br>◯ Matig<br>◯ Goed<br>◯ Uitstekend

Paget LDA, *et al. BMJ Open* 2019; 9:e030961. doi: 10.1136/bmjopen-2019-030961

Vragenlijst PRIMA studie 6 weken - SF-36

Number	Question	Answers
	Deze vragenlijst gaat over uw standpunten t.a.v. uw gezondheid. Met behulp van deze gegevens kan worden bijgehouden hoe u zich voelt en hoe goed u in staat bent uw gebruikelijke bezigheden uit te voeren. Beantwoord elke vraag door een antwoord aan te klikken. Als u niet zeker weet hoe u een vraag moet beantwoorden, geef dan het best mogelijke antwoord.	
6.1	Hoe zou u over het algemeen uw gezondheid noemen?	◯ Uitstekend ◯ Zeer goed ◯ Goed ◯ Matig ◯ Slecht
6.2	Hoe beoordeelt u nu uw gezondheid over het algemeen vergeleken met een jaar geleden?	◯ Veel beter dan een jaar geleden ◯ Wat beter dan een jaar geleden ◯ Ongeveer hetzelfde als een jaar geleden ◯ Wat slechter dan een jaar geleden ◯ Veel slechter dan een jaar geleden
6.3	Wordt u door uw gezondheid op dit moment beperkt bij forse inspanning, zoals hardlopen, tillen van zware voorwerpen of een veeleisende sport beoefenen?	◯ Ja, ernstig beperkt ◯ Ja, een beetje beperkt ◯ Nee, helemaal niet beperkt
6.4	Wordt u door uw gezondheid op dit moment beperkt bij matige inspanning zoals een tafel verplaatsen, stofzuigen, zwemmen of fietsen?	◯ Ja, ernstig beperkt ◯ Ja, een beetje beperkt ◯ Nee, helemaal niet beperkt
6.5	Wordt u door uw gezondheid op dit moment beperkt bij boodschappen tillen of dragen?	◯ Ja, ernstig beperkt ◯ Ja, een beetje beperkt ◯ Nee, helemaal niet beperkt
6.6	Wordt u door uw gezondheid op dit moment beperkt bij een paar trappen oplopen?	◯ Ja, ernstig beperkt ◯ Ja, een beetje beperkt ◯ Nee, helemaal niet beperkt
6.7	Wordt u door uw gezondheid op dit moment beperkt bij één trap oplopen?	◯ Ja, ernstig beperkt ◯ Ja, een beetje beperkt ◯ Nee, helemaal niet beperkt
6.8	Wordt u door uw gezondheid op dit moment beperkt bij bukken, knielen of hurken?	◯ Ja, ernstig beperkt ◯ Ja, een beetje beperkt ◯ Nee, helemaal niet beperkt
6.9	Wordt u door uw gezondheid op dit moment beperkt bij meer dan één kilometer lopen?	◯ Ja, ernstig beperkt ◯ Ja, een beetje beperkt ◯ Nee, helemaal niet beperkt
6.10	Wordt u door uw gezondheid op dit moment beperkt bij een paar honderd meter lopen?	◯ Ja, ernstig beperkt ◯ Ja, een beetje beperkt ◯ Nee, helemaal niet beperkt

Paget LDA, *et al. BMJ Open* 2019; 9:e030961. doi: 10.1136/bmjopen-2019-030961

6.11	Wordt u door uw gezondheid op dit moment beperkt bij ongeveer honderd meter lopen?	◯ Ja, ernstig beperkt ◯ Ja, een beetje beperkt ◯ Nee, helemaal niet beperkt
6.12	Wordt u door uw gezondheid op dit moment beperkt bij uzelf wassen of aankleden?	◯ Ja, ernstig beperkt ◯ Ja, een beetje beperkt ◯ Nee, helemaal niet beperkt
6.13	U besteedde in de afgelopen 4 weken minder tijd aan werk of andere bezigheden	◯ Ja ◯ Nee
6.14	U heeft in de afgelopen 4 weken minder bereikt dan u zou willen	◯ Ja ◯ Nee
6.15	U was in de afgelopen 4 weken beperkt in het soort werk of andere bezigheden.	◯ Ja ◯ Nee
6.16	U had de afgelopen 4 weken moeite om uw werk of andere bezigheden uit te voeren (het kostte u bijvoorbeeld extra inspanning).	◯ Ja ◯ Nee
6.17	U besteedde in de afgelopen 4 weken minder tijd aan werk of andere bezigheden ten gevolge van emotionele problemen	◯ Ja ◯ Nee
6.18	U heeft in de afgelopen 4 weken minder bereikt dan u zou willen ten gevolge van emotionele problemen.	◯ Ja ◯ Nee
6.19	U deed de afgelopen 4 weken uw werk of andere bezigheden niet zo zorgvuldig als gewoonlijk ten gevolge van emotionele problemen.	◯ Ja ◯ Nee
6.20	In hoeverre hebben uw lichamelijke gezondheid of emotionele problemen u gedurende de afgelopen 4 weken gehinderd in uw normale omgang met familie, vrienden of buren, of bij activiteiten in groepsverband?	◯ Helemaal niet ◯ Enigszins ◯ Nogal ◯ Veel ◯ Heel erg veel
6.21	Hoeveel lichamelijke pijn heeft u de afgelopen 4 weken gehad?	◯ Geen<br>◯ Heel licht<br>◯ Licht<br>◯ Nogal<br>◯ Ernstig<br>◯ Heel ernstig
6.22	In welke mate bent u de afgelopen 4 weken door de pijn gehinderd in uw normale werk (zowel werk buitenshuis als huishoudelijk werk)?	◯ Helemaal niet<br>◯ Een klein beetje<br>◯ Nogal<br>◯ Veel<br>◯ Heel erg veel

Paget LDA, *et al*. *BMJ Open* 2019; 9:e030961. doi: 10.1136/bmjopen-2019-030961

6.23	Voelde u zich levenslustig?	◯ Altijd<br>◯ Meestal<br>◯ Vaak<br>◯ Soms<br>◯ Zelden<br>◯ Nooit
6.24	Was u erg zenuwachtig?	◯ Altijd<br>◯ Meestal<br>◯ Vaak<br>◯ Soms<br>◯ Zelden<br>◯ Nooit
6.25	Zat u zo in de put dat u niets kon?	◯ Altijd<br>◯ Meestal<br>◯ Vaak<br>◯ Soms<br>◯ Zelden<br>◯ Nooit
6.26	Voelde u zich rustig en tevreden?	◯ Altijd<br>◯ Meestal<br>◯ Vaak<br>◯ Soms<br>◯ Zelden<br>◯ Nooit
6.27	Had u veel energie?	◯ Altijd<br>◯ Meestal<br>◯ Vaak<br>◯ Soms<br>◯ Zelden<br>◯ Nooit
6.28	Voelde u zich somber en neerslachtig?	◯ Altijd<br>◯ Meestal<br>◯ Vaak<br>◯ Soms<br>◯ Zelden<br>◯ Nooit

Paget LDA, *et al. BMJ Open* 2019; 9:e030961. doi: 10.1136/bmjopen-2019-030961

6.29	Voelde u zich uitgeput?	◯ Altijd<br>◯ Meestal<br>◯ Vaak<br>◯ Soms<br>◯ Zelden<br>◯ Nooit
6.30	Was u een gelukkig mens?	◯ Altijd<br>◯ Meestal<br>◯ Vaak<br>◯ Soms<br>◯ Zelden<br>◯ Nooit
6.31	Voelde u zich moe?	◯ Altijd<br>◯ Meestal<br>◯ Vaak<br>◯ Soms<br>◯ Zelden<br>◯ Nooit
6.32	Hoe vaak hebben uw lichamelijke gezondheid of emotionele problemen u gedurende de afgelopen 4 weken gehinderd bij uw sociale activiteiten (zoals vrienden of familie bezoeken etc.)?	◯ Altijd<br>◯ Meestal<br>◯ Soms<br>◯ Zelden<br>◯ Nooit
6.33	Ik lijk wat gemakkelijker ziek te worden dan andere mensen	◯ Volkomen juist<br>◯ Grotendeels juist<br>◯ Weet ik niet<br>◯ Grotendeels onjuist<br>◯ Volkomen onjuist
6.34	Ik ben even gezond als andere mensen die ik ken	◯ Volkomen juist<br>◯ Grotendeels juist<br>◯ Weet ik niet<br>◯ Grotendeels onjuist<br>◯ Volkomen onjuist

Paget LDA, *et al. BMJ Open* 2019; 9:e030961. doi: 10.1136/bmjopen-2019-030961

6.35	Ik verwacht dat mijn gezondheid achteruit zal gaan	◯ Volkomen juist<br>◯ Grotendeels juist<br>◯ Weet ik niet<br>◯ Grotendeels onjuist<br>◯ Volkomen onjuist
6.36	Mijn gezondheid is uitstekend	◯ Volkomen juist<br>◯ Grotendeels juist<br>◯ Weet ik niet<br>◯ Grotendeels onjuist<br>◯ Volkomen onjuist

Vragenlijst PRIMA studie 6 weken - GAS

Number	Question	Answers
	Tijdens de eerste afspraak werd een duidelijk doel met u afgesproken. Dit werd tevens per email naar u gestuurd. Hoe is de situatie nu ten aanzien van de destijds gestelde doelstelling?	
7.1	Wat is nu de situatie ten aanzien van het tijdens de eerste afspraak afgesproken doelstelling (Goal attainment Scaling)?	◯ Achteruitgang (minder dan de utigangssituatie)<br>◯ Uitgangssituatie<br>◯ Minder dan het doel<br>◯ Doel<br>◯ Meer dan het doel<br>◯ Veel meer dan het doel

Vragenlijst PRIMA studie 6 weken - EQ-5D-3L

Number	Question	Answers
	Deze vragenlijst gaat over uw standpunten t.a.v. uw gezondheid. Met behulp van deze gegevens kan worden bijgehouden hoe u zich voelt en hoe goed u in staat bent uw gebruikelijke bezigheden uit te voeren. Beantwoord elke vraag door een antwoord aan te klikken. Als u niet zeker weet hoe u een vraag moet beantwoorden, geef dan het best mogelijke antwoord.	
8.1	Hoe is het met uw Mobiliteit gesteld?	◯ Ik heb geen problemen met lopen<br>◯ Ik heb enige problemen met lopen<br>◯ Ik ben bedlegerig

Paget LDA, *et al*. *BMJ Open* 2019; 9:e030961. doi: 10.1136/bmjopen-2019-030961

8.2	Hoe is het met uw Zelfzorg gesteld?	◯ Ik heb geen problemen om mijzelf te wassen of aan te kleden<br>◯ Ik heb enige problemen om mijzelf te wassen of aan te kleden<br>◯ Ik ben niet in staat om mijzelf te wassen of aan te kleden
8.3	Hoe is het met de Dagelijkse activiteiten (werk, studie, huishouden, gezins- en vrijetijdsactiviteiten) gesteld?	◯ Ik heb geen problemen met mijn dagelijkse activiteiten<br>◯ Ik heb enige problemen met mijn dagelijkse activiteiten<br>◯ Ik ben niet in staat om mijn dagelijkse activiteiten uit te voeren
8.4	Hoe is het met de Pijn/klachten gesteld?	◯ Ik heb geen pijn of andere klachten<br>◯ Ik heb matige pijn of andere klachten<br>◯ Ik heb zeer ernstige pijn of andere klachten
8.5	Hoe is het met de Stemming gesteld?	◯ Ik ben niet angstig of somber<br>◯ Ik ben matig angstig of somber<br>◯ Ik ben erg angstig of somber
	We willen weten hoe goed of slecht uw gezondheid VANDAAG is. Deze meetschaal loopt van 0 tot 100: 100 staat voor de BESTE gezondheid die u zich kunt voorstellen - 0 staat voor de SLECHTSTE gezondheid die u zich kunt voorstellen	
8.6	Uw Gezondheid vandaag	(0.00) (100.00)

Vragenlijst PRIMA studie 6 weken - AOS

Number	Question	Answers
	Instructies: De lijn naast elke vraag staat voor hoeveel PIJN u heeft in verschillende situaties. De linker kant (0) is “geen pijn” en de rechter kant (100) is “ergste pijn denkbaar”. Geef voor de onderstaande situaties op de lijn aan hoeveel PIJN u in de afgelopen week in de enkel had. Als een of meerdere van deze situaties niet van toepassing waren, dan kiest u de "niet van toepassing" optie.	
9.1	Wat was de hoogte van de ergste pijn in de afgelopen week?	(0.00) (100.00)
9.2	Heeft u pijn als u 's ochtends opstaat?	◯ Ja<br>◯ Nee<br>◯ Niet van toepassing

Paget LDA, *et al. BMJ Open* 2019; 9:e030961. doi: 10.1136/bmjopen-2019-030961

9.2.1	***If 'Heeft u pijn als u 's ochtends opstaat?' is equal to 'Ja' answer this question:***<br>Hoeveel pijn heeft u voordat u 's ochtends opstaat?	(0.00)	(100.00)
9.3	Heeft u pijn wanneer u op blote voeten loopt?	◯ Ja<br>◯ Nee<br>◯ Niet van toepassing	
9.3.1	***If 'Heeft u pijn wanneer u op blote voeten loopt?' is equal to 'Ja' answer this question:***<br>Hoeveel pijn heeft u als u op blote voeten loopt?	(0.00)	(100.00)
9.4	Heeft u pijn wanneer u op blote voeten staat?	◯ Ja<br>◯ Nee<br>◯ Niet van toepassing	
9.4.1	***If 'Heeft u pijn wanneer u op blote voeten staat?' is equal to 'Ja' answer this question:***<br>Hoeveel pijn heeft u als u op blote voeten staat?	(0.00)	(100.00)
9.5	Heeft u pijn wanneer u met schoenen loopt?	◯ Ja<br>◯ Nee<br>◯ Niet van toepassing	
9.5.1	***If 'Heeft u pijn wanneer u met schoenen loopt?' is equal to 'Ja' answer this question:***<br>Hoeveel pijn heeft u wanneer u met schoenen loopt?	(0.00)	(100.00)
9.6	Heeft u pijn wanneer u met schoenen staat?	◯ Ja<br>◯ Nee<br>◯ Niet van toepassing	
9.6.1	***If 'Heeft u pijn wanneer u met schoenen staat?' is equal to 'Ja' answer this question:***<br>Hoeveel pijn heeft u wanneer u met schoenen staat?	(0.00)	(100.00)
9.7	Heeft u pijn wanneer u loopt met steunzolen of een brace?	◯ Ja<br>◯ Nee<br>◯ Niet van toepassing	
9.7.1	***If 'Heeft u pijn wanneer u loopt met steunzolen of een brace?' is equal to 'Ja' answer this question:***<br>Hoeveel pijn heeft u wanneer u loopt met steunzolen of een brace?	(0.00)	(100.00)

Paget LDA, *et al. BMJ Open* 2019; 9:e030961. doi: 10.1136/bmjopen-2019-030961

9.8	Heeft u pijn wanneer u staat met steunzolen of een brace?	◯ Ja<br>◯ Nee<br>◯ Niet van toepassing	
9.8.1	***If 'Heeft u pijn wanneer u staat met steunzolen of een brace?' is equal to 'Ja' answer this question:***<br>Hoeveel pijn heeft u wanneer u staat met steunzolen of een brace?	(0.00)	(100.00)
9.9	Heeft u pijn aan het einde van de dag?	◯ Ja<br>◯ Nee<br>◯ Niet van toepassing	
9.9.1	***If 'Heeft u pijn aan het einde van de dag?' is equal to 'Ja' answer this question:***<br>Hoeveel pijn heeft u aan het einde van de dag?	(0.00)	(100.00)
	Instructies:De lijn naast elke vraag staat voor hoeveel MOEITE u heeft met verschillende activiteiten. De linker kant (0) is “Niet moeilijk” en de rechter kant (100) is “Te moeilijk, niet uitvoerbaar”. Geef voor de onderstaande activiteiten op de lijn aan hoeveel MOEITE u in de afgelopen week door enkelklachten had met onderstaande activiteiten. Als een situatie niet van toepassing is, kies dan de optie "niet van toepassing".		
9.10	Heeft u moeite met door het huis lopen?	◯ Ja<br>◯ Nee<br>◯ Niet van toepassing	
9.10.1	***If 'Heeft u moeite met door het huis lopen?' is equal to 'Ja' answer this question:***<br>Hoeveel moeite heeft u met door het huis lopen?	(0.00)	(100.00)
9.11	Heeft u moeite met buiten lopen op oneven ondergrond?	◯ Ja<br>◯ Nee<br>◯ Niet van toepassing	
9.11.1	***If 'Heeft u moeite met buiten lopen op oneven ondergrond?' is equal to 'Ja' answer this question:***<br>Hoeveel moeite heeft u met buiten lopen op oneven ondergrond?	(0.00)	(100.00)
9.12	Heeft u moeite met een paar honderd meter lopen?	◯ Ja<br>◯ Nee<br>◯ Niet van toepassing	
9.12.1	***If 'Heeft u moeite met een paar honderd meter lopen?' is equal to 'Ja' answer this question:***<br>Hoeveel moeite heeft u met een paar honderd meter lopen?	(0.00)	(100.00)

Paget LDA, *et al. BMJ Open* 2019; 9:e030961. doi: 10.1136/bmjopen-2019-030961

9.13	Heeft u moeite met een trap oplopen?	◯ Ja<br>◯ Nee<br>◯ Niet van toepassing	
9.13.1	***If 'Heeft u moeite met een trap oplopen?' is equal to 'Ja' answer this question:***<br>Hoeveel moeite heeft u met een trap oplopen?	(0.00)	(100.00)
9.14	Heeft u moeite met een trap aflopen?	◯ Ja<br>◯ Nee<br>◯ Niet van toepassing	
9.14.1	***If 'Heeft u moeite met een trap aflopen?' is equal to 'Ja' answer this question:***<br>Hoeveel moeite heeft u met een trap aflopen?	(0.00)	(100.00)
9.15	Heeft u moeite met op de tenen staan?	◯ Ja<br>◯ Nee<br>◯ Niet van toepassing	
9.15.1	***If 'Heeft u moeite met op de tenen staan?' is equal to 'Ja' answer this question:***<br>Hoeveel moeite heeft u met op de tenen staan?	(0.00)	(100.00)
9.16	Heeft u moeite met opstaan uit de stoel?	◯ Ja<br>◯ Nee<br>◯ Niet van toepassing	
9.16.1	***If 'Heeft u moeite met opstaan uit de stoel?' is equal to 'Ja' answer this question:***<br>Hoeveel moeite heeft u met opstaan uit de stoel?	(0.00)	(100.00)
9.17	Heeft u moeite met het op- of afstappen van stoepranden?	◯ Ja<br>◯ Nee<br>◯ Niet van toepassing	
9.17.1	***If 'Heeft u moeite met het op- of afstappen van stoepranden?' is equal to 'Ja' answer this question:***<br>Hoeveel moeite heeft u met het op- of afstappen van stoepranden?	(0.00)	(100.00)
9.18	Heeft u moeite met snel lopen of rennen?	◯ Ja<br>◯ Nee<br>◯ Niet van toepassing	

Paget LDA, *et al. BMJ Open* 2019; 9:e030961. doi: 10.1136/bmjopen-2019-030961

9.18.1	***If 'Heeft u moeite met snel lopen of rennen?' is equal to 'Ja' answer this question:*** Hoeveel moeite heeft u met snel lopen of rennen?	(0.00)	(100.00)

Vragenlijst PRIMA studie 6 weken - FAOS

Number	Question	Answers
	Deze lijst vraagt naar uw mening over uw voet/enkel. Uw antwoorden geven ons een beeld van uw voet/enkel klachten en hoe u in staat bent om alledaagse activiteiten uit te voeren in uw huidige situatie. Beantwoorden van een vraag doet u door het aanklikken van een vakje met het volgens u meest juiste antwoord (één vakje per vraag). Als u niet zeker weet hoe u een vraag moet beantwoorden, geeft u dan het antwoord dat volgens u het meest op uw situatie van toepassing is. Deze vraag heeft betrekking op het voorkomen van voet/enkel klachten in de afgelopen week.	
10.1	Is uw voet/enkel gezwollen?	○ Nooit ○ Zelden ○ Soms ○ Vaak ○ Altijd
10.2	Voelt u gekraak of hoort u klikken of een ander vreemd geluid wanneer u de voet/enkel beweegt?	○ Nooit ○ Zelden ○ Soms ○ Vaak ○ Altijd
10.3	Hapert uw enkel of blokkeert uw enkel ('op slot' gaan zitten) wanneer u deze beweegt?	○ Nooit ○ Zelden ○ Soms ○ Vaak ○ Altijd
10.4	Kunt u de voet/enkel volledig strekken?	○ Altijd ○ Vaak ○ Soms ○ Zelden ○ Nooit
10.5	Kunt u de voet volledig naar u toe buigen?	○ Altijd ○ Vaak ○ Soms ○ Zelden ○ Nooit
10.6	In welke mate heeft u een stijf gevoel in de voet/enkel 's ochtends bij het wakker worden?	○ Niet ○ Mild ○ Matig ○ Ernstig ○ Zeer ernstig
10.7	In welke mate heeft u een stijf gevoel in de voet/enkel na zitten, liggen of rusten later op de dag?	○ Niet ○ Mild ○ Matig ○ Ernstig ○ Zeer ernstig
10.8	Hoe vaak heeft u pijn in uw voet/enkel?	○ Nooit ○ Maandelijks ○ Wekelijks ○ Dagelijks ○ Altijd

Paget LDA, *et al. BMJ Open* 2019; 9:e030961. doi: 10.1136/bmjopen-2019-030961

10.9	Hoeveel voet/enkel pijn heeft u gehad in de afgelopen week bij draaien als uw voet/enkel op de grond staat?	◯ Geen<br>◯ Mild<br>◯ Matig<br>◯ Ernstig<br>◯ Zeer ernstig
10.10	Hoeveel voet/enkel pijn heeft u gehad in de afgelopen week bij het volledig uitstrekken van de voet/enkel?	◯ Geen<br>◯ Mild<br>◯ Matig<br>◯ Ernstig<br>◯ Zeer ernstig
10.11	Hoeveel voet/enkel pijn heeft u gehad in de afgelopen week bij het volledig naar u toe buigen/optrekken van de voet/enkel	◯ Geen<br>◯ Mild<br>◯ Matig<br>◯ Ernstig<br>◯ Zeer ernstig
10.12	Hoeveel voet/enkel pijn heeft u gehad in de afgelopen week bij het lopen op een vlakke ondergrond	◯ Geen<br>◯ Mild<br>◯ Matig<br>◯ Ernstig<br>◯ Zeer ernstig
10.13	Hoeveel voet/enkel pijn heeft u gehad in de afgelopen week bij het trap op- en trap af lopen?	◯ Geen<br>◯ Mild<br>◯ Matig<br>◯ Ernstig<br>◯ Zeer ernstig
10.14	Hoeveel voet/enkel pijn heeft u gehad in de afgelopen week 's nachts in bed?	◯ Geen<br>◯ Mild<br>◯ Matig<br>◯ Ernstig<br>◯ Zeer ernstig
10.15	Hoeveel voet/enkel pijn heeft u gehad in de afgelopen week bij het zitten of liggen?	◯ Geen<br>◯ Mild<br>◯ Matig<br>◯ Ernstig<br>◯ Zeer ernstig

Paget LDA, *et al. BMJ Open* 2019; 9:e030961. doi: 10.1136/bmjopen-2019-030961

10.16	Hoeveel voet/enkel pijn heeft u gehad in de afgelopen week bij het rechtop staan?	◯ Geen<br>◯ Mild<br>◯ Matig<br>◯ Ernstig<br>◯ Zeer ernstig
10.17	In welke mate werd u gehinderd bij het trap aflopen?	◯ Niet ◯ Mild ◯ Matig ◯ Ernstig ◯ Zeer ernstig
10.18	In welke mate werd u gehinderd bij het trap op lopen?	◯ Niet ◯ Mild ◯ Matig ◯ Ernstig ◯ Zeer ernstig
10.19	In welke mate werd u gehinderd als u vanuit een zittende positie ging staan?	◯ Niet ◯ Mild ◯ Matig ◯ Ernstig ◯ Zeer ernstig
10.20	In welke mate werd u gehinderd bij het staan?	◯ Niet ◯ Mild ◯ Matig ◯ Ernstig ◯ Zeer ernstig
10.21	In welke mate werd u gehinderd bij het naar de grond buigen/iets oprapen?	◯ Niet ◯ Mild ◯ Matig ◯ Ernstig ◯ Zeer ernstig
10.22	In welke mate werd u gehinderd bij het lopen op een vlakke ondergrond?	◯ Niet ◯ Mild ◯ Matig ◯ Ernstig ◯ Zeer ernstig
10.23	In welke mate werd u gehinderd bij het in- en uit de auto stappen?	◯ Niet ◯ Mild ◯ Matig ◯ Ernstig ◯ Zeer ernstig
10.24	In welke mate werd u gehinderd bij het boodschappen doen?	◯ Niet ◯ Mild ◯ Matig ◯ Ernstig ◯ Zeer ernstig
10.25	In welke mate werd u gehinderd bij sokken/panty's aantrekken?	◯ Niet ◯ Mild ◯ Matig ◯ Ernstig ◯ Zeer ernstig
10.26	In welke mate werd u gehinderd bij het opstaan uit bed?	◯ Niet ◯ Mild ◯ Matig ◯ Ernstig ◯ Zeer ernstig
10.27	In welke mate werd u gehinderd bij het sokken uittrekken?	◯ Niet ◯ Mild ◯ Matig ◯ Ernstig ◯ Zeer ernstig
10.28	In welke mate werd u gehinderd bij het in bed liggen (omdraaien, lange tijd uw voet/enkel in dezelfde positie houden)?	◯ Niet ◯ Mild ◯ Matig ◯ Ernstig ◯ Zeer ernstig

Paget LDA, *et al. BMJ Open* 2019; 9:e030961. doi: 10.1136/bmjopen-2019-030961

10.29	In welke mate werd u gehinderd bij het in/uit bad stappen?	◯ Niet ◯ Mild ◯ Matig ◯ Ernstig ◯ Zeer ernstig
10.30	In welke mate werd u gehinderd bij zitten?	◯ Niet ◯ Mild ◯ Matig ◯ Ernstig ◯ Zeer ernstig
10.31	In welke mate werd u gehinderd bij het toilet op en af gaan?	◯ Niet ◯ Mild ◯ Matig ◯ Ernstig ◯ Zeer ernstig
10.32	In welke mate werd u gehinderd bij zwaar huishoudelijk werk (bijvoorbeeld zware dozen sjouwen, vloer schrobben)?	◯ Niet ◯ Mild ◯ Matig ◯ Ernstig ◯ Zeer ernstig
10.33	In welke mate werd u gehinderd bij licht huishoudeijk werk (bijvoorbeeld koken, afstoffen)?	◯ Niet ◯ Mild ◯ Matig ◯ Ernstig ◯ Zeer ernstig
10.34	In welke mate werd u gehinderd bij hurken?	◯ Niet ◯ Mild ◯ Matig ◯ Ernstig ◯ Zeer ernstig
10.35	In welke mate werd u gehinderd bij hardlopen?	◯ Niet ◯ Mild ◯ Matig ◯ Ernstig ◯ Zeer ernstig
10.36	In welke mate werd u gehinderd bij springen?	◯ Niet ◯ Mild ◯ Matig ◯ Ernstig ◯ Zeer ernstig
10.37	In welke mate werd u gehinderd bij ronddraaien op uw aangedane voet/enkel?	◯ Niet ◯ Mild ◯ Matig ◯ Ernstig ◯ Zeer ernstig
10.38	In welke mate werd u gehinderd bij knielen?	◯ Niet ◯ Mild ◯ Matig ◯ Ernstig ◯ Zeer ernstig
10.39	Hoe vaak bent u zich bewust van uw voet/enkel probleem?	◯ Nooit ◯ Maandelijks ◯ Wekelijks ◯ Dagelijks ◯ Altijd
10.40	Heeft u uw leven veranderd om activiteiten te vermijden die schadelijk kunnen zijn voor uw voet/enkel?	◯ Niet<br>◯ Enigszins<br>◯ Matig<br>◯ Behoorlijk<br>◯ Volledig

Paget LDA, *et al. BMJ Open* 2019; 9:e030961. doi: 10.1136/bmjopen-2019-030961

10.41	In hoeverre kunt u op uw voet/enkel vertrouwen?	◯ Volledig<br>◯ Behoorlijk<br>◯ Matig<br>◯ Enigzins<br>◯ Niet
10.42	In het algemeen, in welke mate ondervindt u hinder van uw voet/enkel	◯ Geen<br>◯ Mild<br>◯ Matig<br>◯ Ernstig<br>◯ Zeer ernstig

Vragenlijst PRIMA studie 6 weken - Dank

Number	Question	Answers
11.1	Hartelijk dank voor de tijd die u heeft genomen om de vragenlijsten in te vullen. Indien u nog opmerkingen heeft kunt u deze hierin plaatsen.	

Paget LDA, *et al. BMJ Open* 2019; 9:e030961. doi: 10.1136/bmjopen-2019-030961
