# Supplementary material for: Platelet-Rich plasma Injection Management for Ankle osteoarthritis study (PRIMA): protocol of a Dutch multicentre, stratified, block-randomised, double-blind, placebo-controlled trial
Source: BMJ Open. 2019 Oct 7;9(10):e030961. doi: 10.1136/bmjopen-2019-030961 (PMC6797250; doi:10.1136/bmjopen-2019-030961)
Supplement: Author's manuscript [file bmjopen-2019-030961.draft_revisions.pdf]

BMJ Open is committed to open peer review. As part of this commitment we make the peer review history of every article we publish publicly available.

When an article is published we post the peer reviewers' comments and the authors' responses online. We also post the versions of the paper that were used during peer review. These are the versions that the peer review comments apply to.

The versions of the paper that follow are the versions that were submitted during the peer review process. They are not the versions of record or the final published versions. They should not be cited or distributed as the published version of this manuscript.

BMJ Open is an open access journal and the full, final, typeset and author-corrected version of record of the manuscript is available on our site with no access controls, subscription charges or pay-per-view fees (http://bmjopen.bmj.com).

If you have any questions on BMJ Open's open peer review process please email info.bmjopen@bmj.com

BMJ Open

Platelet Rich plasma Injection Management for Ankle osteoarthritis study (PRIMA) Protocol of a multi-center, stratified, block-randomized, double-blind, placebo-controlled trial

Journal:	*BMJ Open*
Manuscript ID	bmjopen-2019-030961
Article Type:	Protocol
Date Submitted by the Author:	14-Apr-2019

Complete List of Authors:	Paget, Liam; Amsterdam UMC - Locatie AMC, Orthopaedic Surgery<br>Bierma-Zeinstra, Sita; Erasmus University Medical Centre, Department of General Practice<br>Goedegebuure, S; OLVG, The Sport Physician Group, department of Sports Medicine<br>Kerkhoffs, G; Amsterdam UMC - Locatie AMC, Orthopaedic surgery<br>Krips, R; Flevoziekenhuis, Orthopaedic Surgery<br>Maas, Mario; Amsterdam UMC - Locatie AMC<br>Moen, Maarten; The Bergman Clinics, Sports Medicine<br>Reurink, G; Amsterdam UMC - Locatie AMC<br>Stufkens, S; Amsterdam UMC - Locatie AMC<br>de Vos, Robert-Jan; Erasmus University Medical Centre, Department of Orthopaedics and Sports Medicine<br>Weir, A; Erasmus University Medical Centre, 5. Department of Orthopaedics and sports medicine; Aspetar Qatar Orthopaedic and Sports Medicine Hospital, Orthopaedics and Sport Medicine<br>Tol, J; Amsterdam UMC - Locatie AMC, Orthopaedic Surgery

Keywords:	Foot & ankle < ORTHOPAEDIC & TRAUMA SURGERY, Adult orthopaedics < ORTHOPAEDIC & TRAUMA SURGERY, SPORTS MEDICINE, ORTHOPAEDIC & TRAUMA SURGERY, Platelet-rich Plasma

SCHOLARONE™
Manuscripts

Platelet Rich plasma Injection Management for Ankle osteoarthritis study (PRIMA)

Protocol of a multi-center, stratified, block-randomized, double-blind, placebo-controlled trial

Authors: L.D.A. Paget[1,2,3], S.M.A. Bierma – Zeinstra[4,5], S. Goedegebuure[2,6], G.M.M.J. Kerkhoffs[1,2,3], R. Krips[7], M. Maas[3,8,9], M. H. Moen[6,10], G. Reurink[1,2,3,6], S. A. S. Stufkens[1,2,3], R.J. de Vos[5], A. Weir[5,12,13], J.L. Tol[1,2,3,6,12]

1. Department of Orthopaedic Surgery, Amsterdam UMC, University of Amsterdam, Amsterdam Movement Sciences, Amsterdam, the Netherlands.
2. Academic Center for Evidence-based Sports medicine (ACES), Amsterdam UMC, Amsterdam, the Netherlands
3. Amsterdam Collaboration for Health and Safety in Sports (ACHSS), AMC/VUmc IOC Research Center, Amsterdam, the Netherlands
4. Department of General Practice, Erasmus University Medical Center, Rotterdam, The Netherlands
5. Department of Orthopaedics and sports medicine, Erasmus University Medical Center, Rotterdam, The Netherlands
6. The Sport Physician Group, Department of Sports Medicine, OLVG, Amsterdam, The Netherlands
7. Department of Orthopaedic Surgery, Flevoziekenhuis, Almere, The Netherlands
8. Department of Musculoskeletal Radiology, The Netherlands
9. Musculoskeletal Imaging Quantification Center (MIQC), The Netherlands.
10. Department of Sports Medicine , Bergman Clinics , Naarden , the Netherlands
11. Aspetar, Orthopaedic and Sports Medicine Hospital, Doha, Qatar
12. Aspetar Sports Groin Pain Centre, Aspetar Orthopaedic and Sports Hospital, Doha, Qatar.
13. Sport medicine and exercise clinic Haarlem (SBK). Haarlem, The Netherlands.

Correspondence

Liam. D.A. Paget

Department of Orthopaedic Surgery,

Amsterdam UMC

PO Box 22660, 1100 DD Amsterdam

The Netherlands

L.d.paget@amsterdamumc.nl

For peer review only

ABSTRACT

Introduction

The onset of ankle osteoarthritis (OA) occurs at a relatively young age in contrast to knee OA and hip OA, with a reported incidence of 1% to 4% in the general adult population. Systematic reviews showed that platelet-rich plasma (PRP) injections significantly decrease pain and improve function in knee OA patients.

In case series PRP injections in ankle OA are shown to be safe and improve quality of life over time, but no randomised controlled trial has been conducted. The objective of the present study is to evaluate the efficacy of PRP injections for symptom reduction and functional improvement, compared to placebo, in the treatment of ankle (talocrural) OA.

Methods and analysis

We will conduct the Platelet Rich plasma Injection Management for Ankle osteoarthritis (PRIMA) study: a multicentre, randomised, placebo-controlled trial. One hundred patients suffering from ankle OA will be randomised into two treatment groups: PRP injection or placebo (saline) injection. Both groups will receive two injections of PRP or placebo at an interval of 6 weeks. Primary outcome is the American Orthopaedic Foot and Ankle Society (AOFAS) score at 26 weeks . Secondary outcomes determined at several follow-up moments up to 5 years, include Ankle Osteoarthritis Score (AOS), Foot and Ankle Outcome Score (FAOS), pain sub-scale of AOFAS (0-40), VAS score (VAS 0-100), Ankle activity score (AAS 0-10), Subjective patient satisfactionShort form Survey-36 (SF-36), Global Attainment Scaling (GAS) and the EQ-5D-3L utility score. A cost-effectiveness analysis will be performed at 1 year.

Ethics and dissemination

The study is approved by the local Medical Ethics Committee (ABR 2018-042, approved 23-7-2018) and registered in the Dutch trial register (NTR 7261). Results and new knowledge will be

For peer review only

disseminated through the Dutch Arthritis Association (ReumaNederland), the Dutch patient federation, conferences and published in a scientific peer-reviewed journal.

Trial registration number

(NTR7261)

For peer review only

STRENGTH AND LIMITATIONS

- This is the first study that evaluates the efficacy and cost-effectiveness of ankle (talocrural) OA in a multi-center, stratified, block-randomized, double-blind, placebo-controlled trial design.
- A positive outcome of this study will have a significant effect on widespread availability and nationwide implementation of an intra articular injection treatment for ankle OA with PRP. Similarly, a negative outcome (no effect of PRP), will prevent the widespread use of a non-efficacious treatment on patients.
- The study population consists only of subjects with isolated ankle OA and no concomitant OA of one or more other major joints of the lower extremities that negatively affects their daily activity level
- The outcome concerning the PRP product researched in this randomised controlled trial is prepared using one of the available commercial PRP products. Therefore it has limited generalisability. This is similarly the case for alternative treatment regimens using a different dose or a different number of intra-articular injections.
- The composition of PRP will not be analysed. This decision was based on the fact that PRP preparation and composition has frequently been analysed.[9]

For peer review only

BACKGROUND AND RATIONALE

PRP is a potentially efficacious treatment for ankle OA but its use has not been examined in high quality studies. Ankle OA is more common than hip or knee OA in the young active population. [10] The incidence of symptomatic ankle OA is estimated at 1% to 4% in the general adult population.[2] Patients with ankle OA have a quality of life and physical functioning which is comparable with hip OA and end-stage kidney disease or congestive heart failure.[11] These relatively young (predominantly female) patients are increased risk for decreased work participation and family care. The available surgical intervention (arthrodesis) is associated with significant functional limitations. In contrast to hip and knee OA, where joint replacement is an excellent surgical alternative for severe cases, there is a clear need for effective non-surgical interventions in ankle OA.

Recent systematic reviews on the intra-articular injection therapy in ankle OA found a lack of evidence from high quality studies to assist in clinical decision-making.[12,13] When compared to placebo injections, hyaluronic acid or corticosteroid injections, PRP injections might significantly decrease pain and improve function in knee OA patients.[14–16] Given the clinical effect on pain reduction in knee OA and a good safety profile, PRP is a promising non-surgical therapy for ankle OA.[3,4] PRP might delay the irreversible surgical options like arthrodesis. No significant adverse events have been reported in any PRP trials regarding acute hamstring injuries, achilles tendinopathy, knee OA or ankle OA. [6,7,15,17–20]. Clinical studies on PRP in ankle OA are limited to a single report of 5 cases and a prospective case series of 20 subjects, which both showed safety and significant reduction of the VAS-FA score at a mean of 16 months and 12 weeks follow-up.[5,6] Well-designed prospective Randomised controlled trials (RCT) have not yet been performed.

For peer review only

METHODS AND DESIGN

Objectives

With the PRIMA study we aim to determine the efficacy of PRP injections in the management of ankle OA by comparing 2 groups, both receiving 2 injections of either: PRP or a placebo solution. We hypothesize that PRP injections are efficacious for symptom reduction and functional improvement compared to placebo in the treatment of ankle (talocrural) OA.

Study Design

The PRIMA study is a multi-center, stratified, block-randomized, double-blind, placebo-controlled trial design will be conducted in order to compare two treatment groups: PRP vs Placebo (saline). After the 26 weeks follow-up of the last patient in the study, the principal investigator and coordinating researcher will be unblinded only after the analysis of the primary outcome. A flow chart of the design and follow-up is shown in figure 1.

Patient involvement

Active patient involvement through reviewing the proposal (Reviewer level):

Active involvement of patients during the design phase is already secured by active involvement of 2 patients with ankle OA. Both reviewed the grant proposal.

Execution phase – *patients will actively contribute:*

Active patient involvement will be secured by invitation of at least 2 patients for the annual trial monitoring and evaluation meetings (Informant level).

Analysis phase – *Patient will review and interpret the results (reviewer level):*

For peer review only

Using the blinded codes of the randomisation groups and after breaking the randomisation code, patients (also active in the design phase) will be given the opportunity to interpret the results from a patient perspective.

Dissemination phase – *Patient oriented approach* (Advisor level)*:*

Dissemination activities will comprise a social media strategy with active patient involvement to share results with online patient communities and an events attendance strategy enabling patient participation in seminars, conferences and workshops.

Fig.1 illustrating the PRIMA multicentre randomized controlled trial design and follow-up procedure

Study Population

Population (base)

Patients with ankle OA in University Medical Centres, teaching hospitals, general hospitals and private specialist clinics will be informed about the study. In order to participate, patients must meet the eligibility criteria documented below.

Inclusion criteria

1. Severity of Ankle OA pain on a visual analogue scale (VAS) (0–100 mm) ≥ 40 mm during daily activities
2. X-rays (AP and lateral view) indicating ≥ grade 2 talocrural OA on the Van Dijk classification (clarified under section Radiographs)[8]
3. Age ≥ 18 years

For peer review only

Exclusion criteria

1. Patient has received injection therapy for ankle OA in the previous 6 months
2. Patient does not want to receive one of the two therapies
3. Patient has clinical signs of concomitant OA of one or more other major joints of the lower extremities that negatively affects their daily activity level
4. Previous ankle surgery for OA or Osteochondral defects (OCD) < 1 year (not including surgery for an ankle fracture in the past)

Radiographs

AP and lateral X-rays of the talocrural joints will be scored according to the Van Dijk classification:[8]

0) Normal joint or subchondral sclerosis
1) Osteophytes without joint space narrowing
2) Joint space narrowing with or without osteophytes
3) (Sub)total disappearance or deformation of the joint space

Randomisation, Blinding and Treatment Allocation

We will include patients at the centre of their first outpatient clinic appointment. For each patient the coordinating researcher will prepare a syringe with PRP and a syringe with placebo (isotonic saline: 0.9% sodium chloride). A Good Clinical Practice (GCP) approved data management system (Castor EDC, based in Amsterdam, the Netherlands) will be used to perform a computer generated block randomisation scheme with patients stratified to centre with a variable block size of 2,4 or 6. This procedure will ensure treatment allocation concealment. The coordinating researcher, treating physician and patient remain blinded to the allocated intervention. An independent researcher from the coordinating location will have access to the randomization result and the allocated intervention. This will be relayed to a GCP-certified research assistant at the centre. The research assistant then

For peer review only

selects one of the two syringes based on the allocated intervention and blinds the syringe with a covering sheath, ensuring concealment of the content of the syringe. The patients, treating physicians, and coordinating researcher will all be blinded to the allocation of the intervention and to the contents of the syringe. The success of blinding will be assessed by asking participants which injection they think they have received just after the injection procedure, this will then be registered accordingly.

After the 26 weeks follow-up of the last patient in the study, the principal investigator and coordinating researcher will be unblinded only after the analysis of the primary outcome. The patients will remain blinded to the therapy until the 52 weeks follow-up (online questionnaire) of the last patient in the study.

Intervention

In this study, patients will be randomised into two treatment groups: PRP injection or placebo saline injection. For each patient the coordinating researcher will prepare a PRP and a placebo injection (isotonic saline: 0.9% sodium chloride). The PRP syringe will be prepared according to the instructions of the manufacturer.[17] Prior to commencement of the study, the coordinating researcher was trained by a representative of Arthrex, as well as two experienced members of the PRIMA trial research group (dr. G Reurink en dr. RJ de Vos) with a vast experience regarding PRP preparation and injection. The PRP will be prepared using a widely used and commercially available system (Arthrex double syringe PRP system, Arthrex Medizinische Instrumente GmbH, Garching, Germany).

One syringe of 15ml autologous blood will be collected twice from the cubital vein: at inclusion and at a time interval of 6 weeks. After blood collection the syringe will undergo 5 minutes of centrifugation and the injection will be given within 30 minutes following venepuncture to prevent formation of blood clots. No additional substances (calcium, thrombin or citrate) will be added to the PRP solution. For each procedure, 2 ml of PRP or placebo will be prepared and injected into the affected ankle joint under ultrasonographic guidance. All subjects will receive a second injection at 6

For peer review only

weeks, regardless of the effect of the first injection. To guarantee blinding for the allocated treatment of the patient, treatment assessor and treating physician, blood will be drawn and both PRP and placebo will be prepared according to exactly the same procedure for each patient (both at inclusion and at a time interval of 6 weeks after the first injection). However, in the control group, subjects will be injected with 2ml of physiological saline instead of PRP. An unblinded research assistant will blind one of the two identical syringes based on the allocated intervention using a specially manufactured covering sheath in order to conceal randomization. Following the intra-articular injection, the syringe covered by the sheath (containing either the remnants of the PRP or saline), will be directly handed to the unblinded research assistant, who will immediately dispose of the syringe, therefore keeping the treating physician and coordinating researcher blinded. After the intervention, patients are advised to avoid heavy or repetitive stress to the ankle joint for a period of 48 hours. Furthermore, patients receive a leaflet containing usual-care healthy life style advice beneficial for ankle OA. This includes losing weight and exercise (avoiding heavy or repetitive ankle loading exercises) such as walking, cycling or swimming.

For peer review only

Use of co-intervention

Patients are instructed to avoid the use of co-interventions and Non Steroidal Anti-Inflammatory Drugs if possible, 24 hours prior to the intervention and if possible up to a year after the first injection. Throughout the study, key treatment (including usual care: exercise therapy and healthy life style advice) and co-interventions used by participants will be registered, such as Non Steroidal Anti-Inflammatory Drugs (NSAID's), other analgesic drugs, intra-articular injections or inlays.

Study Procedures

In the event the subject meets the criteria for inclusion and exclusion, the subject will be informed in more detail about the study procedure. At that time the patient can ask questions about the study and decide whether they will participate and sign the informed consent form. Subsequently the patient will proceed to inclusion and the randomization procedure.

Inclusion

Patients are recruited for inclusion by their treating physicians at location. Following inclusion PRP will be prepared according to the PRP system instructions of the manufacturer. During the first two consultations a total of two intra-articular injections will be documented with a time interval of 6 weeks. The patient will have no additional costs to usual care as a result of taking part in this study.

Physical examination will be performed on three occasions up to 26 weeks from baseline. Patient Reported Outcome Measures (PROMs) will be used to evaluate the treatment effect up to five years. A cost-effectivity analysis will be performed at one year using the PRODISQ questionnaire. The time-frame of the follow-up questionnaires is further illustrated in table 1. Questionnaires will be managed and distributed digitally using a GCP approved data management system (Castor EDC)

For peer review only

Follow-up	
Baseline	- 1st intervention injections<br>- Physical Examination<br>- AOFAS<br>- PROMs<br>- PRODISQ cost-effectivity
6 weeks	- 2nd intervention injection<br>- Physical Examination<br>- AOFAS<br>- PROMs
12 weeks	- AOFAS<br>- PROMs<br>- PRODISQ cost-effectivity
26 weeks	- AOFAS<br>- PROMs<br>- PRODISQ cost-effectivity
39 weeks	- PRODISQ cost-effectivity
52 weeks	- AOFAS<br>- PROMs<br>- PRODISQ cost-effectivity
5 years	- AOFAS<br>- PROMs

Table 1. In addition to the American Orthopaedic Foot and Ankle Society (AOFAS) score, the following PROMs will be taken: Foot and Ankle Outcome Score (FAOS), Ankle Osteoarthritis Score (AOS), Visual Analogue Scale (VAS), Ankle activity score (AAS), Subjective patient satisfaction, Short Form Health Survey (SF-36), The Global Attainment Scaling (GAS), EuroQol-5 dimensions-3 levels (EQ-5D-3L). These PROMs will be elaborated on further on. Furthermore the PROductivity and DISease Questionnaire (PRODISQ) will be used to perform a cost-effectivity analysis.

For peer review only

Replacement of individual subjects after withdrawal

In the sample size calculation we compensated for an expected loss of 10% of patients to follow-up. No patients will be replaced after withdrawal.

Safety

All adverse events reported spontaneously by the subject or observed by the investigator or his staff will be recorded. A variety of conditions have been treated with PRP ranging from muscle and tendon

injuries to intra-articular injections of the knee and ankle. No serious adverse events have been documented to date concerning PRP intra-articular injections of the ankle.

Outcome Measures

Main study parameter/endpoint

The primary objective of this study will be to quantify pain or function improvement using the American Orthopaedic Foot and Ankle Society (AOFAS) score at 26 weeks follow-up. Studies evaluating the efficacy of PRP in knee OA maintained a follow-up between 3 and 12 months. We have therefore opted to take 26 weeks as our primary outcome measure, fitting within the aforementioned range.[3] The AOFAS is a validated scale for ankle OA (0-100) measuring three subdomains (pain, function and alignment) which together total nine items.[21–24] Subdomain pain is measured by one item where a maximal score of 40 indicates no pain. Function consists of 7 items where full function is indicated by the maximal score of 50 points. Similar to the pain subdomain, alignment has a potential maximum score of 10 points using one item, indicating good alignment.[21,22] The AOFAS questionnaire, having undergone forward and backward translation to Dutch by de Boer et al. 2017, has an excellent internal consistency (Cronbach's α 0.947) and an excellent test-retest reliability (ICC 0.93).[21]

Secondary study parameters/endpoints

Secondary outcome measures are a number of other Patient Reported Outcome Measures (PROMs). These will be taken at baseline, 6, 12, 26 and 52 weeks as well as 5 years

1. Ankle Osteoarthritis Score (AOS) is a visual analog scale from 0 – 100 mm for 18 questions, with 9 relating to pain and 9 relating to disability.
2. Foot and Ankle Outcome Score (FAOS). Each question gets assigned 0 – 4 points based on the answer given. The scale runs from 0 (extreme symptoms) to 100 points (no symptoms).
3. In order to evaluate pain, the pain sub-scale of AOFAS (0-40 points) will be analysed. On this scale the lower the score the more pain the patient has. Additionally a VAS score (VAS 0-100

For peer review only

mm) is measured during activities of daily living, with 0 mm being no pain and 100 mm the worst pain imaginable.

4. The total AOFAS score at the other time points than the primary one
5. Ankle activity score (0-10 points) is scored according to chart based on the performable activity level
6. Subjective patient satisfaction (4 categories) Poor, Fair, Good, Excellent
7. Short Form Health Survey SF-36 is a health-related quality of life score (0-100 points). The higher the patient scores, the higher the disability.
8. The Global Attainment Scaling (GAS) is a method of scoring based on achievement related to pre-determined goals in agreement with the patient. Points are subtracted for not achieving the pre-defined goals or vice versa. Scores range from 100 (high functioning) to 0 (severely impaired)
9. EuroQol-5 dimensions-3 levels (EQ-5D-3L) utility score allows a patient's health to be defined by a 5-digit number.
10. PROductivity and DISease Questionnaire (PRODISQ) will be used to determine indirect costs and direct costs cost effectivity. The PRODISQ questionnaire is taken at baseline and every 3 months thereafter up until 1 year. This will be done in conjunction with the EQ-5D-3L.

Loss to follow-up

The coordinating researcher will attempt to limit loss to follow-up as much as possible by contacting every subject and being present at every subject visit. All digital questionnaires will be constantly monitored to ensure they are being filled in and otherwise followed-up by the coordinating researcher. In the event of subject withdrawal, an analysis of demographic and prognostic characteristics will be done on these cases and the remaining patients. As previously described by Järvinen et al. 2014, we will document the participant eligible for and compliant with each follow-up.[25]

For peer review only

For peer review only

Missing items

Missing items of a score will be handled according to the instructions of the specific scales. In the event of no instructions, we will calculate the percentage of missing items on a scale. Due to the potential impact on trial conclusions, multiple imputation (if more than 10% missing items on a scale) will be applied.

Sample Size

Based on previous and ongoing studies, the study protocol of the randomised controlled trial is designed to detect a difference of 12 points (0-100) on the AOFAS score. There is no official agreement on the minimal clinical important difference for the AOFAS score regarding ankle OA. However in relatable musculoskeletal literature, 10% – 15% of the used scale was reported. [18,26,27] Our pre-defined minimal clinical important difference of 12 % is located within this range.[18,26,27] Based on a previous placebo controlled RCT on injection therapy (hyaluronic acid) in ankle OA of De Groot et al. a standard deviation of 16.3 can be expected.[13] Taking into account a two-sided level of significance of 5%, a power of 90% and a dropout rate of 10%, approximately 50 (45 plus 10% drop out) patients per group will be needed (N=100).

Data Management

After giving permission for participating in this study, participants will receive a link to fill in digital surveys. All data gained outside Castor EDC will be stored on the AMC secured hard drive. All data will be coded and stored in the Castor EDC online database which meets the AMC safety criteria and good clinical practice guidelines. The primary investigator and project leader will safeguard the coded data through password secured access. All participant's data will be archived for at least 15 years and handled with in accordance with the Dutch Personal Data Protection Act (Wbp). Data protections is provided through the safety protocol of Castor EDC with automated backups and SSL security.

Statistical analysis

A standard operating procedure will be available to logically recode and clean the data. The data will be interpreted according to a blinded data interpretation scheme described by Järvinen et al, 2014.[25]

For peer review only

The authors will interpret the statistical results until a consensus is reached. Once the authors are in agreement the two groups will be unblinded and no changes will be applied to the interpretation of the results.

Baseline characteristics

Baseline characteristics will be analyzed between groups using descriptive statistics.

Primary outcome measure

Analysis will be performed using an intention to treat approach. To test for the effect of treatment on the between-group difference in primary outcome, we will use the repeated measurement general linear model. Changes from baseline to all follow-up time points will be included in the model. Adjustments will be made for those baseline variables that influenced the primary outcome with $p < 0.10$.

Secondary outcome measures

To test for the effect of treatment on the between-group difference in the secondary outcomes, we will use the repeated measurement general linear model. Changes from baseline to all follow-up time points will be included in the model.

Economic analysis

In the event of a positive significant outcome, an economic analysis is needed to support a possible change of practice. An economic analysis (costs) will be performed in order to determine cost-effectiveness.

We will assess the differences in mean quality-adjusted life years (QALYs), costs, and net benefits between the PRP injection group and the placebo group using linear models. We express the cost-effectiveness by using cost-effectiveness acceptability curves from both a healthcare perspective and a societal perspective. With multiple bootstrap replicates of the average difference in costs and

For peer review only

effects in the incremental cost-effectiveness plane we will express the uncertainty of our cost-effectiveness analysis.

The cost-effectivity analysis will be performed with a 1-year time horizon. We use the three-level EQ-5D questionnaire (Euroqol, Rotterdam, the Netherlands) to calculate QALYs as the area under the curve of the utility scores measured over 12 months, according to the Dutch tariff. The analysis will be based on indirect costs and direct costs and will be determined using the PRODISQ questionnaire. The PRODISQ questionnaire is taken at baseline and every 3 months thereafter up until 1 year.

Monitoring committee

The PRIMA trial will be monitored by the clinical research unit (CRU) of the coordination study centre. The CRU aims to improve the quality of clinical research and ultimately, patient care. Before the PRIMA trial commenced, a monitoring plan was set-up. This Monitoring plan facilitates compliance with the Human Research Act (WMO), Good Clinical Practice (ICH-GCP) guidelines (5.18.1) and/ or ISO14155, which require monitors to verify that (a) the rights and well-being of human subjects are protected,

(b) recorded study data is accurate, complete, and verifiable with the source documents,

(c) the conduct of the study is compliant with the currently approved protocol and with applicable laws regulatory requirements, e.g. WMO, ICH-GCP, ISO14155.

Throughout the study, monitoring will be performed at initiation of the trial, at each participating centre once approximately 3 subjects have been enrolled, after approximately 10 – 15 enrolled subjects have completed the 26 week follow-up, after 70 subjects have been enrolled and after database lock.

For peer review only

Ethics and Dissemination

The study is approved by the local Medical Ethics Committee (ABR 2018-042, approved 23-7-2018) and is registered in the Dutch trial register (NTR 7261). The results of this project study and new knowledge will be disseminated through the Dutch Arthritis Association (ReumaNederland), presentations, news publications, blogs, websites social media and professional organisations (rheumatology, orthopaedics, primary care medicine, sports medicine, public health) and the Dutch patient federation (Patiëntenfederatie Nederland). The participants will also be informed about the results of the study. The study will be conducted according to the principles of the Declaration of Helsinki, GCP and the Medical Research Involving Human Subjects Act (WMO). The board of each participating hospital reviewed and approved the local feasibility. Monitoring and auditing will be carried out throughout the study. In the event of Serious Adverse Events, these will be documented and reported to the monitor and the Medical Ethics board. Eligible patients will be informed of the study and will sign an informed consent form before participating.

Discussion

This is the first study that evaluates the efficacy and cost-effectiveness of Platelet-rich Plasma injections in the treatment of ankle OA in a multi-center, stratified, block-randomized, double-blind, placebo-controlled trial design. The primary outcome is the AOFAS score at 26 weeks. This study will also evaluate the long term efficacy of PRP for up to five years.

Platelet-rich Plasma has been shown to alleviate symptoms of patients suffering from knee OA.[4] In the absence of effective evidence-based non-surgical interventions for ankle OA, PRP would fulfil a clear clinical need. This will be the first randomized controlled trial to evaluate the efficacy of PRP in ankle OA. Previous trials concerned with the efficacy of PRP for multiple indications are usually of low

For peer review only

quality and adhere to a follow-up of 26 weeks or shorter. Furthermore a cost-effectiveness analysis has never been performed in previous trials on PRP in the treatment of OA.

A positive study outcome would have a significant impact leading to change in clinical practice, where PRP could be offered as a non-surgical intervention, in the management of ankle OA, if available. A negative outcome, will prevent the widespread use of a non-efficacious treatment on patients.

This study has potential limitations. First of all, due to financial constraints, (semi-quantitative) MRI-defined cartilage degenerative changes could not be used as a secondary outcome score. Furthermore, the composition of PRP was not analysed. This decision was based on the fact that PRP preparation and composition has frequently been analysed.[9] Lastly, the outcome concerning the PRP product researched in this randomised controlled trial is prepared using a Hettich Rotofix32 A centrifuge and Arthrex syringes. Therefore it has limited generalisability compared to other PRP products. This is similarly the case for alternative treatment regimens using a different dose or a different number of intra-articular injections.

Conclusion

This will be the first randomized controlled trial to assess both the efficacy and cost-effectiveness of PRP in ankle OA.

Contributor ship statement

All authors have been actively involved in creating and writing the study protocol, and all authors critically commented on the paper and gave their final approval of the version that we submitted.

Competing interests

For peer review only

All authors have completed the ICMJE uniform disclosure form at www.icmje.org/coi_disclosure.pdf and declare: no support from any organisation for the submitted work; no financial relationships with any organisations that might have an interest in the submitted work in the previous three years; no other relationships or activities that could appear to have influenced the submitted work.

Funding

This multicenter Randomized Controlled Trial was supported by a grant from the Dutch Arthritis Foundation (ReumaNederland)

Figure legends/captions

Fig.1 illustrating the PRIMA multicentre randomized controlled trial design and follow-up procedure

For peer review only

References

1. Saltzman CL, Salamon ML, Blanchard GM, et al. Epidemiology of ankle arthritis: report of a consecutive series of 639 patients from a tertiary orthopaedic center. *Iowa Orthop J*. 2005.

2. Cushnaghan J, Dieppe P. Study of 500 patients with limb joint osteoarthritis. I. Analysis by age, sex, and distribution of symptomatic joint sites. *Ann Rheum Dis*. 1991;50(1):8-13. doi:10.1136/ard.50.1.8

3. Xing D, Wang B, Zhang W, et al. Intra-articular platelet-rich plasma injections for knee osteoarthritis: An overview of systematic reviews and risk of bias considerations. *Int J Rheum Dis*. 2017;20(11):1612-1630. doi:10.1111/1756-185X.13233

4. Dai WL, Zhou AG, Zhang H, Zhang J. Efficacy of Platelet-Rich Plasma in the Treatment of Knee Osteoarthritis: A Meta-analysis of Randomized Controlled Trials. *Arthrosc - J Arthrosc Relat Surg*. 2017. doi:10.1016/j.arthro.2016.09.024

5. Angthong C, Khadsongkram A, Angthong W. Outcomes and quality of life after platelet-rich plasma therapy in patients with recalcitrant hindfoot and ankle diseases: A preliminary report of 12 patients. *J Foot Ankle Surg*. 2013;52(4):475-480. doi:10.1053/j.jfas.2013.04.005

6. Fukawa T, Yamaguchi S, Akatsu Y, Yamamoto Y, Akagi R, Sasho T. Safety and Efficacy of Intra-articular Injection of Platelet-Rich Plasma in Patients With Ankle Osteoarthritis. *Foot Ankle Int*. 2017;38(6):596-604. doi:10.1177/1071100717700377

7. Repetto I, Biti B, Cerruti P, Trentini R, Felli L. Conservative Treatment of Ankle Osteoarthritis: Can Platelet-Rich Plasma Effectively Postpone Surgery? *J Foot Ankle Surg*. 2017;56(2):362-365. doi:10.1053/j.jfas.2016.11.015

8. van Dijk CN, Verhagen R a, Tol JL. Arthroscopy for problems after ankle fracture. *J Bone Jt Surg*. 1997;79-B(2):280-284. doi:10.1302/0301-620X.79B2.7153

9. Oudelaar BW, Peerbooms JC, Huis in 't Veld R, Vochteloo AJH. Concentrations of Blood Components in Commercial Platelet-Rich Plasma Separation Systems: A Review of the Literature. *Am J Sports Med*. 2018. doi:10.1177/0363546517746112

10. Murray C, Marshall M, Rathod T, Bowen CJ, Menz HB, Roddy E. Population prevalence and distribution of ankle pain and symptomatic radiographic ankle osteoarthritis in community dwelling older adults: A systematic review and cross-sectional study. *PLoS One*. 2018. doi:10.1371/journal.pone.0193662

11. Saltzman CL. Impact of Comorbidities on the Measurement of Health in Patients with Ankle Osteoarthritis. *J Bone Jt Surg*. 2006;88(11):2366. doi:10.2106/JBJS.F.00295

12. Vannabouathong C, Del Fabbro G, Sales B, et al. Intra-articular Injections in the Treatment of Symptoms from Ankle Arthritis: A Systematic Review. *Foot Ankle Int*.

For peer review only

2018:107110071877937. doi:10.1177/1071100718779375

13. DeGroot H, Uzunishvili S, Weir R, Al-omari A, Gomes B. Intra-articular injection of hyaluronic acid is not superior to saline solution injection for ankle arthritis: a randomized, double-blind, placebo-controlled study. *J Bone Joint Surg Am*. 2012;94(1):2-8. doi:10.2106/JBJS.J.01763

14. Meheux CJ, McCulloch PC, Lintner DM, Varner KE, Harris JD. Efficacy of Intra-articular Platelet-Rich Plasma Injections in Knee Osteoarthritis: A Systematic Review. *Arthrosc - J Arthrosc Relat Surg*. 2016;32(3):495-505. doi:10.1016/j.arthro.2015.08.005

15. Laudy ABM, Bakker EWP, Rekers M, Moen MH. Efficacy of platelet-rich plasma injections in osteoarthritis of the knee: a systematic review and meta-analysis. *Br J Sports Med*. 2015;49(10):657-672. doi:10.1136/bjsports-2014-094036

16. Moen M, Weir A, Bakker E, Rekers M, Laudy G. Efficacy of platelet-rich plasma injections in osteoarthritis of the knee: an updated systematic review and meta-analysis. *Osteoarthr Cartil*. 2016;24:S520-S521. doi:10.1016/j.joca.2016.01.953

17. Reurink G, Goudswaard GJ, Moen MH, et al. Platelet-Rich Plasma Injections in Acute Muscle Injury. *N Engl J Med*. 2014;370(26):2546-2547. doi:10.1056/NEJMc1402340

18. de Vos RJ, Weir A, van Schie HTM, et al. Platelet-rich plasma injection for chronic Achilles tendinopathy: a randomized controlled trial. *JAMA*. 2010;303(2):144-149. doi:10.1001/jama.2009.1986

19. Pas HI, Reurink G, Tol JL, Weir A, Winters M, Moen MH. Efficacy of rehabilitation (lengthening) exercises, platelet-rich plasma injections, and other conservative interventions in acute hamstring injuries: an updated systematic review and meta-analysis. *Br J Sports Med*. 2015;49(18):1197-1205. doi:10.1136/bjsports-2015-094879

20. Hamilton B, Tol JL, Almusa E, et al. Platelet-rich plasma does not enhance return to play in hamstring injuries: a randomised controlled trial. *Br J Sports Med*. 2015;49(14):943-950. doi:10.1136/bjsports-2015-094603

21. De Boer AS, Tjioe RJC, Van Der Sijde F, et al. The American Orthopaedic Foot and Ankle Society Ankle-Hindfoot Scale; Translation and validation of the Dutch language version for ankle fractures. *BMJ Open*. 2017;7(8):1-12. doi:10.1136/bmjopen-2017-017040

22. Kitaoka HB, Alexander IJ, Adelaar RS, Nunley JA, Myerson MS, Sanders M. Clinical rating systems for the ankle-hindfoot, midfoot, hallux, and lesser toes. *Foot ankle Int*. 1994;15(7):349-353. doi:10.1177/107110079401500701

23. Sayyed-Hosseinian S-H, Hassankhani GG, Bagheri F, Alavi N, Shojaie B, Mousavian A. Validation of the Persian Version of the American Orthopedic Foot and Ankle Society Score (AOFAS) Questionnaire. *Arch bone Jt Surg*. 2018;6(3):233-239. doi:10.1097/PHM.0000000000000234

For peer review only

24. Vosoughi AR, Roustaei N, Mahdaviazad H. American Orthopaedic Foot and Ankle Society ankle–hindfoot scale: A cross-cultural adaptation and validation study from Iran. *Foot Ankle Surg*. 2018. doi:10.1016/j.fas.2017.02.007

25. Järvinen TLN, Sihvonen R, Bhandari M, et al. Blinded interpretation of study results can feasibly and effectively diminish interpretation bias. *J Clin Epidemiol*. 2014;67(7):769-772. doi:10.1016/j.jclinepi.2013.11.011

26. Pham T, van der Heijde D, Altman RD, et al. OMERACT-OARSI initiative: Osteoarthritis research society international set of responder criteria for osteoarthritis clinical trials revisited. *Osteoarthr Cartil*. 2004. doi:10.1016/j.joca.2004.02.001

27. Rozendaal RM, Koes BW, Van Osch GJVM, et al. Effect of glucosamine sulfate on hip osteoarthritis: A randomized trial. *Ann Intern Med*. 2008. doi:10.7326/0003-4819-148-4-200802190-00005

For peer review only

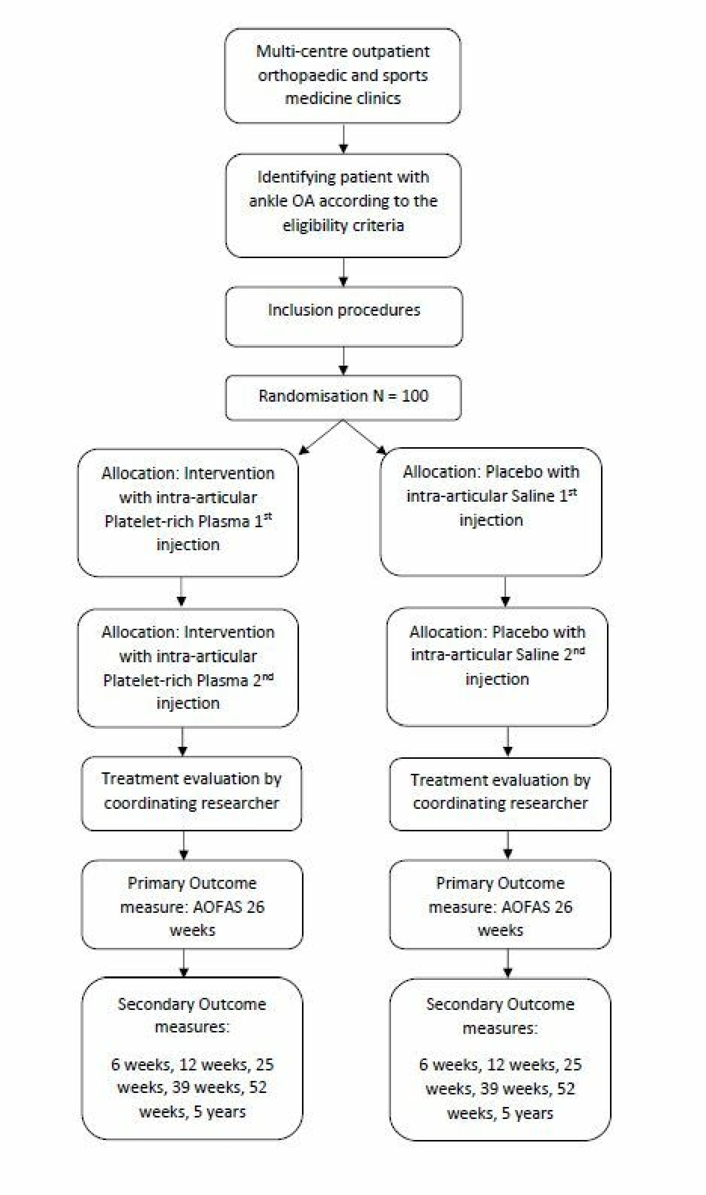

Fig.1 illustrating the PRIMA multicentre randomized controlled trial design and follow-up procedure

39x67mm (300 x 300 DPI)

BMJ Open

Platelet Rich plasma Injection Management for Ankle osteoarthritis study (PRIMA) Protocol of a Dutch multi-center, stratified, block-randomized, double-blind, placebo-controlled trial

Journal:	*BMJ Open*
Manuscript ID	bmjopen-2019-030961.R1
Article Type:	Protocol
Date Submitted by the Author:	16-Sep-2019
Complete List of Authors:	Paget, Liam; Amsterdam UMC - Locatie AMC, Orthopaedic Surgery; Amsterdam Collaboration for Health and Safety in Sports (ACHSS), AMC/VUmc IOC Research Center<br>Bierma-Zeinstra, Sita; Erasmus University Medical Centre, Department of General Practice; Erasmus Medical Center, Orthopaedic Surgery<br>Goedegebuure, S; OLVG, the Sport Physician Group, Sports Medicine; Academic Center for Evidence-based Sports medicine (ACES), Amsterdam UMC<br>Kerkhoffs, Gino; Amsterdam UMC - Locatie AMC, Orthopaedic Surgery; Amsterdam Collaboration for Health and Safety in Sports (ACHSS), AMC/VUmc IOC Research Center<br>Krips, R; Flevoziekenhuis, Orthopaedic Surgery<br>Maas, Mario; Amsterdam UMC - Locatie AMC, Radiology; Academic Center for Evidence-based Sports medicine (ACES), AmsterdamUMC<br>Moen, Maarten; Bergman Clinics, Sports Medicine; OLVG, the Sport Physician Group<br>Reurink, G; Academic Center for Evidence-based Sports medicine (ACES), Amsterdam UMC; OLVG, the Sport Physician Group, Sports Medicine<br>Stufkens, Sjoerd; Amsterdam UMC - Locatie AMC, Orthopaedic Surgery; Amsterdam Collaboration for Health and Safety in Sports (ACHSS), AMC/VUmc IOC Research Center<br>de Vos, Robert-Jan; Erasmus University Medical Centre, Orthopaedics and Sports Medicine<br>Weir, A; Erasmus University Medical Centre, Orthopaedics and sports medicine; Sports medicine and exercise clinic Haarlem (SBK)<br>Tol, J; Academic Center for Evidence-based Sports medicine (ACES), Amsterdam UMC; Aspetar Orthopaedic and Sports Medicine Hospital
**Primary Subject Heading**:	Sports and exercise medicine
Secondary Subject Heading:	Surgery, Sports and exercise medicine
Keywords:	Foot & ankle < ORTHOPAEDIC & TRAUMA SURGERY, SPORTS MEDICINE, ORTHOPAEDIC & TRAUMA SURGERY, Platelet-rich Plasma, Osteoarthritis, Ankle Osteoarthritis

For peer review only - http://bmjopen.bmj.com/site/about/guidelines.xhtml

SCHOLARONE™
Manuscripts

Platelet Rich plasma Injection Management for Ankle osteoarthritis study (PRIMA)

Protocol of a Dutch multi-center, stratified, block-randomized, double-blind, placebo-controlled trial

Authors: L.D.A. Paget[1,2,3], S.M.A. Bierma – Zeinstra[4,5], S. Goedegebuure[2,6], G.M.M.J. Kerkhoffs[1,2,3], R. Krips[7], M. Maas[3,8,9], M. H. Moen[6,10], G. Reurink[1,2,3,6], S. A. S. Stufkens[1,2,3], R.J. de Vos[5], A. Weir[5,12,13], J.L. Tol[1,2,3,6,11]

1. Department of Orthopaedic Surgery, Amsterdam UMC, University of Amsterdam, Amsterdam Movement Sciences, Amsterdam, the Netherlands.
2. Academic Center for Evidence-based Sports medicine (ACES), Amsterdam UMC, Amsterdam, the Netherlands
3. Amsterdam Collaboration for Health and Safety in Sports (ACHSS), AMC/VUmc IOC Research Center, Amsterdam, the Netherlands
4. Department of General Practice, Erasmus University Medical Center, Rotterdam, The Netherlands
5. Department of Orthopaedics and Sports Medicine, Erasmus University Medical Center, Rotterdam, The Netherlands
6. The Sport Physician Group, Department of Sports Medicine, OLVG, Amsterdam, The Netherlands
7. Department of Orthopaedic Surgery, Flevoziekenhuis, Almere, The Netherlands
8. Department of Musculoskeletal Radiology, The Netherlands
9. Musculoskeletal Imaging Quantification Center (MIQC), The Netherlands.
10. Department of Sports Medicine, Bergman Clinics, Naarden, the Netherlands
11. Aspetar, Orthopaedic and Sports Medicine Hospital, Doha, Qatar
12. Aspetar Sports Groin Pain Centre, Orthopaedic and Sports Medicine Hospital, Doha, Qatar
13. Sport medicine and exercise clinic Haarlem (SBK). Haarlem, The Netherlands.

Correspondence

Liam. D.A. Paget

Department of Orthopaedic Surgery,

Amsterdam UMC

PO Box 22660, 1100 DD Amsterdam

The Netherlands

L.d.paget@amsterdamumc.nl

For peer review only - http://bmjopen.bmj.com/site/about/guidelines.xhtml

ABSTRACT

Introduction

PRP is a potentially efficacious treatment for ankle OA but its use has not been examined in high quality studies. Systematic reviews show that platelet-rich plasma (PRP) injections significantly decrease pain and improve function in knee OA patients. Ankle OA is more common than hip or knee OA in the young active population; with a prevalence of 3.4%.

PRP injections in ankle OA are shown to be safe and improve quality of life over time, but no randomised controlled trial has been conducted. Our randomised controlled trial will evaluate the efficacy of PRP injections for symptom reduction and functional improvement, compared to placebo, in the treatment of ankle (talocrural) OA.

Methods and analysis

We will conduct the Platelet Rich plasma Injection Management for Ankle osteoarthritis (PRIMA) study: a multicentre, randomised, placebo-controlled trial. One hundred patients suffering from ankle OA will be randomised into two treatment groups: PRP injection or placebo (saline) injection. Both groups will receive two injections of PRP or placebo at an interval of 6 weeks. Primary outcome is the American Orthopaedic Foot and Ankle Society (AOFAS) score at 26 weeks. Secondary outcomes determined at several follow-up moments up to 5 years, include Ankle Osteoarthritis Score (AOS), Foot and Ankle Outcome Score (FAOS), pain sub-scale of AOFAS (0-40), VAS score (VAS 0-100), Ankle activity score (AAS 0-10), Subjective patient satisfaction Short form Survey-36 (SF-36), Global Attainment Scaling (GAS) and the EQ-5D-3L utility score. A cost-effectiveness analysis will be performed at 1 year.

Ethics and dissemination

The study is approved by the Medical Ethics Review Committee Amsterdam Medical Center, the Netherlands (ABR 2018-042, approved 23-7-2018) and registered in the Netherlands trial register (NTR 7261). Results and new knowledge will be disseminated through the Dutch Arthritis Association

For peer review only - http://bmjopen.bmj.com/site/about/guidelines.xhtml

(ReumaNederland), Dutch patient federation, conferences and published in a scientific peer-reviewed journal.

Trial registration number

(NTR7261)

For peer review only

For peer review only - http://bmjopen.bmj.com/site/about/guidelines.xhtml

STRENGTH AND LIMITATIONS

- This is the first study to evaluate the efficacy and cost-effectiveness of ankle (talocrural) OA in a multi-center, stratified, block-randomized, double-blind, placebo-controlled trial design.
- A positive study outcome will have a significant effect on widespread availability and nationwide implementation of an intra articular injection treatment for ankle OA with PRP. A negative outcome (no effect of PRP), will prevent the widespread use of a non-efficacious treatment on patients.
- The study population consists only of patients with isolated ankle OA and no concomitant OA in other major joints of the lower extremities that negatively affects their daily activity level
- The PRP used in this randomised controlled trial is an available PRP product that is used in clinical practise. However, there is a multitude of commercial PRP products available that differ in their production method and content, and different treatment regimens regarding dose, timing and number of injections are used in clinical practise. The generalizability to other PRP products and treatment regimens remains unknown.
- The composition of PRP will not be analysed. This decision was based on the fact that PRP preparation and composition has frequently been analysed and is typically not performed in clinical practice prior to injection.

For peer review only - http://bmjopen.bmj.com/site/about/guidelines.xhtml

BACKGROUND AND RATIONALE

PRP is a potentially efficacious treatment for ankle OA but its use has not been examined in high quality studies. Ankle OA is more common than hip or knee OA in the young active population. [1] The incidence of symptomatic ankle OA is estimated to be 3.4% in the general adult population.[1] Patients with ankle OA have a quality of life and physical functioning comparable with hip OA, end-stage kidney disease or congestive heart failure.[2] These relatively young (predominantly female) patients have an increased risk for decreased work participation and family care. The available surgical intervention (arthrodesis) is associated with significant functional limitations. In contrast to hip and knee OA, where joint replacement is an excellent surgical alternative for severe cases, there is a clear need for effective non-surgical interventions in ankle OA.

Platelet Rich Plasma (PRP) is defined as plasma containing a concentration of at least 1,000,000 platelets/µl.[3] Growth factors are stored in α-granules within platelets, and are released in a selective manner upon activation. Growth factors released from the α-granules of platelets are assumed to provide the regenerative benefits of PRP.

Recent reviews concluded that in animal models PRP can diminish multiple inflammatory IL-1 mediated effects.[4] Due to this local anti-inflammatory response, PRP might have an indirect analgesic effect. The second suggested effect might be protection of the cartilage from destructive pro-inflammatory interleukines. This is mediated due to an increased mRNA expression of proteoglycan core protein in the articular cartilage and decreased chondrocyte apoptosis.[4] Consequently, PRP could positively influence the collagen network of the cartilage.

Recent systematic reviews on the intra-articular injection therapy in ankle OA found a lack of evidence from high quality studies to assist in clinical decision-making.[5,6] When compared to placebo injections, hyaluronic acid or corticosteroid injections, PRP injections might significantly decrease

For peer review only - http://bmjopen.bmj.com/site/about/guidelines.xhtml

114 pain and improve function in knee OA patients.[7–9] Given the clinical effect on pain reduction in knee

115 OA and a good safety profile, PRP is a promising non-surgical therapy for ankle OA.[10,11] PRP might

116 delay the irreversible surgical options like arthrodesis. No significant adverse events have been

117 reported in any PRP trials regarding ankle OA, knee OA, acute hamstring injuries or Achilles

118 tendinopathy. [8,12–17]. Clinical studies on PRP in ankle OA are limited to a single report of 5 cases and a

119 prospective case series of 20 subjects, which both showed safety and significant reduction of pain at

120 a mean of 16 months and 12 weeks follow-up.[14,18] Well-designed prospective Randomised controlled

121 trials (RCT) have not yet been performed.

122

For peer review only - http://bmjopen.bmj.com/site/about/guidelines.xhtml

For peer review only

METHODS AND DESIGN

Objectives

We aim to determine the efficacy of PRP injections in the management of ankle OA by comparing 2 groups, both receiving 2 injections of either: PRP or placebo solution. We hypothesize that PRP injections are efficacious for symptom reduction and functional improvement compared to placebo in the treatment of ankle (talocrural) OA.

Study Design

The PRIMA study is a multi-center, stratified, block-randomized, double-blind, placebo-controlled trial design will be conducted in order to compare two treatment groups: PRP vs Placebo (saline). After the 26 weeks follow-up of the last patient in the study, the principal investigator and coordinating researcher will be unblinded only after the analysis of the primary outcome. A flow chart of the design and follow-up is shown in figure 1. The study included its first patient in August 2018 and aims to include the last patient by January 2021, consequently allowing analysis and then deblinding to commence after the last follow-up (26 weeks) of the last patient by July 2021.

Patient involvement

Active patient involvement through reviewing the proposal (Reviewer level):

Active involvement of patients during the design phase was performed by 2 patients with ankle OA. Both reviewed the grant proposal and provided feedback.

Execution phase – patients will actively contribute:

Active patient involvement will be secured by invitation of at least 2 patients for the annual trial monitoring and evaluation meetings (Informant level).

For peer review only - http://bmjopen.bmj.com/site/about/guidelines.xhtml
For peer review only

Analysis phase – Patient will review and interpret the results (reviewer level):

Using the blinded codes of the randomisation groups and after breaking the randomisation code, patients (also active in the design phase) will be given the opportunity to interpret the results from a patient perspective.

Dissemination phase – Patient oriented approach (Advisor level)*:*

Results and new knowledge will be disseminated through the Dutch Arthritis Association (ReumaNederland), the Dutch patient federation, conferences and published in a scientific peer-reviewed journal. Dissemination activities will comprise a social media strategy with active patient involvement to share results with online patient communities and an events attendance strategy enabling patient participation in seminars, conferences and workshops.

Fig.1 illustrating the PRIMA multicentre randomized controlled trial design and follow-up procedure

Study Population

Population (base)

Patients with ankle OA in University Medical Centres, teaching hospitals, general hospitals and private specialist clinics will be informed about the study. In order to participate, patients must meet the eligibility criteria documented below.

Inclusion criteria

1. Severity of Ankle OA pain on a visual analogue scale (VAS) (0–100 mm) ≥ 40 mm during daily activities

For peer review only - http://bmjopen.bmj.com/site/about/guidelines.xhtml

2. X-rays (AP and lateral view) indicating ≥ grade 2 talocrural OA on the Van Dijk classification (clarified under section Radiographs)[19]
3. Age ≥ 18 years

Exclusion criteria

1. Patient has received injection therapy for ankle OA in the previous 6 months
2. Patient does not want to receive one of the two therapies
3. Patient has clinical signs of concomitant OA of one or more other major joints of the lower extremities that negatively affects their daily activity level
4. Previous ankle surgery for OA or Osteochondral defects (OCD) < 1 year (not including surgery for an ankle fracture in the past)

Radiographs

AP and lateral X-rays of the talocrural joints will be scored according to the Van Dijk classification:[19]

0) Normal joint or subchondral sclerosis
1) Osteophytes without joint space narrowing
2) Joint space narrowing with or without osteophytes
3) (Sub)total disappearance or deformation of the joint space

Randomisation, Blinding and Treatment Allocation

We will include patients at the centre of their first outpatient clinic appointment. For each patient the coordinating researcher will prepare a syringe with PRP and a syringe with placebo (isotonic saline: 0.9% sodium chloride). A Good Clinical Practice (GCP) approved data management system (Castor EDC, based in Amsterdam, the Netherlands) will be used to perform a computer generated

For peer review only - http://bmjopen.bmj.com/site/about/guidelines.xhtml
For peer review only

block randomisation scheme with patients stratified to centre with a variable block size of 2,4 or 6. This procedure will ensure treatment allocation concealment. The coordinating researcher, treating physician and patient all remain blinded to the allocated intervention. An independent researcher from the coordinating location will have access to the randomization result and the allocated intervention. This will be relayed to a GCP-certified research assistant at the centre. The research assistant then selects one of the two syringes based on the allocated intervention and blinds the syringe with a covering sheath, ensuring concealment of the content of the syringe. The patients, treating physicians, and coordinating researcher will all be blinded to the allocation of the intervention and to the contents of the syringe. The success of blinding will be assessed by asking patients which injection they think they have received just after the injection procedure, this will then be registered accordingly.

For peer review only

After the 26 weeks follow-up of the last patient in the study, the principal investigator and coordinating researcher will be unblinded only after the analysis of the primary outcome. The patients will remain blinded to the therapy until the 52 weeks follow-up (online questionnaire- appendix 1) of the last patient in the study.

Intervention

In this study, patients will be randomised into two treatment groups: PRP injection or placebo saline injection. For each patient the coordinating researcher will prepare a PRP and a placebo injection (isotonic saline: 0.9% sodium chloride). The PRP syringe will be prepared according to the instructions of the manufacturer.[12] Prior to commencement of the study, the coordinating researcher was trained by a representative of Arthrex, as well as two experienced members of the PRIMA trial research group (GR and RJdV) with a vast scientific experience regarding PRP preparation and injection.[12,13] The PRP will be prepared using a widely used and commercially available system (Arthrex double syringe PRP system, Arthrex Medizinische Instrumente GmbH, Garching, Germany).

For peer review only - http://bmjopen.bmj.com/site/about/guidelines.xhtml

One syringe of 15ml autologous blood will be collected twice from the cubital vein: at inclusion and at a time interval of 6 weeks. After blood collection the syringe will undergo 5 minutes of centrifugation and the injection will be given within 30 minutes following venepuncture to prevent formation of blood clots. No additional substances (calcium, thrombin or citrate) will be added to the PRP solution. For each procedure, 2 ml of PRP or placebo will be prepared and injected into the affected ankle joint under ultrasonographic guidance using a sterile technique. All patients will receive a second injection at 6 weeks, regardless of the effect of the first injection. To guarantee blinding for the allocated treatment of the patient, treatment assessor and treating physician, blood will be drawn and both PRP and placebo will be prepared according to exactly the same procedure for each patient (both at inclusion and at a time interval of 6 weeks after the first injection). However, in the control group, patients will be injected with 2ml of physiological saline instead of PRP. An unblinded research assistant will blind one of the two identical syringes based on the allocated intervention using a specially manufactured covering sheath in order to conceal randomization. Following the intra-articular injection, the syringe covered by the sheath (containing either the remnants of the PRP or saline), is handed to the unblinded research assistant, who will dispose of the syringe, therefore keeping the treating physician and coordinating researcher blinded. After the intervention, patients are advised to avoid heavy or repetitive stress to the ankle joint for a period of 48 hours. Furthermore, patients receive a leaflet containing usual-care healthy lifestyle advice beneficial for ankle OA. This includes losing weight and exercise (avoiding heavy or repetitive ankle loading exercises) such as walking, cycling or swimming.

For peer review only

For peer review only - http://bmjopen.bmj.com/site/about/guidelines.xhtml

Use of co-intervention

Patients are instructed to avoid the use of co-interventions and non-steroidal anti-inflammatory drugs if possible, 24 hours prior to the intervention and if possible up to a year after the first injection. Throughout the study, key treatment (including usual care: exercise therapy and healthy life style advice) and co-interventions used by patients will be registered, such as non-steroidal anti-Inflammatory drugs, other analgesic drugs, intra-articular injections or inlays.

Study Procedures

In the event the patient meets the criteria for inclusion and exclusion, the patient will be informed in more detail about the study procedure. At that time the patient can ask questions about the study and decide whether they will participate and sign the informed consent form. Subsequently the patient will proceed to inclusion and the randomization procedure.

Inclusion

Patients are recruited for inclusion by their treating physicians at location. Following inclusion PRP will be prepared according to the PRP system instructions of the manufacturer. During the first two consultations a total of two intra-articular injections will be documented with a time interval of 6 weeks. The patient will have no additional costs to usual care as a result of taking part in this study.

Physical examination will be performed on three occasions up to 26 weeks from baseline. Patient Reported Outcome Measures (PROMs) will be used to evaluate the treatment effect up to five years. A cost-effectivity analysis will be performed at one year using the PRODISQ questionnaire. The time-frame of the follow-up questionnaires is further illustrated in table 1. Questionnaires will be managed and distributed digitally using a GCP approved data management system (Castor EDC)

For peer review only - http://bmjopen.bmj.com/site/about/guidelines.xhtml

Follow-up	
Baseline	- 1st intervention injections<br>- Physical Examination<br>- AOFAS<br>- PROMs<br>- PRODISQ cost-effectivity
6 weeks	- 2nd intervention injection<br>- Physical Examination<br>- AOFAS<br>- PROMs
12 weeks	- AOFAS<br>- PROMs<br>- PRODISQ cost-effectivity
26 weeks	- AOFAS<br>- PROMs<br>- PRODISQ cost-effectivity
39 weeks	- PRODISQ cost-effectivity
52 weeks	- AOFAS<br>- PROMs<br>- PRODISQ cost-effectivity
5 years	- AOFAS<br>- PROMs

Table 1. In addition to the American Orthopaedic Foot and Ankle Society (AOFAS) score, the following PROMs will be taken: Foot and Ankle Outcome Score (FAOS), Ankle Osteoarthritis Score (AOS), Visual Analogue Scale (VAS), Ankle activity score (AAS), Subjective patient satisfaction, Short Form Health Survey (SF-36), The Global Attainment Scaling (GAS), EuroQol-5 dimensions-3 levels (EQ-5D-3L). These PROMs will be elaborated on further on. Furthermore the PROductivity and DISease Questionnaire (PRODISQ) will be used to perform a cost-effectivity analysis. These questionnaires can be found in appendix 1

Replacement of individual patients after withdrawal

In the sample size calculation we compensated for an expected loss of 10% of patients to follow-up. No patients will be replaced after withdrawal.

Safety

For peer review only - http://bmjopen.bmj.com/site/about/guidelines.xhtml

All adverse events reported spontaneously by the patient or observed by the investigator or his staff will be recorded. A variety of conditions have been treated with PRP ranging from muscle and tendon injuries to intra-articular injections of the knee and ankle. To date, no serious adverse events have been documented in the literature, concerning PRP intra-articular injections of the ankle. In accordance with the Central Committee on Research Involving Human Subjects (CCMO) guidelines, this study was classified as low-risk for adverse events. Therefore, the local Medical Ethical Commission will be notified of any serious adverse events. In the event this happens, the advice of the Medical Ethical Commission will be followed accordingly.

Outcome Measures

Primary study parameter/endpoint

The primary objective of this study will be to quantify pain or functional improvement using the American Orthopaedic Foot and Ankle Society (AOFAS) score at 26 weeks follow-up. Studies evaluating the efficacy of PRP in knee OA maintained a follow-up between 3 and 12 months. We therefore opted to take 26 weeks for our primary outcome measure.[10] The AOFAS is a validated scale for ankle OA (0-100) measuring three subdomains (pain, function and alignment) which together total nine items.[20–23] The subdomain of pain is measured by one item where a maximal score of 40 indicates no pain. Function consists of 7 items where full function is indicated by the maximal score of 50 points. Similar to the pain subdomain, alignment has a potential maximum score of 10 points using one item, indicating good alignment.[20,21] The AOFAS questionnaire, having undergone forward and backward translation to Dutch by de Boer et al. 2017, has an excellent internal consistency (Cronbach's α 0.947) and an excellent test-retest reliability (ICC 0.93).[20]

Secondary study parameters/endpoints

Secondary outcome measures are a number of other Patient Reported Outcome Measures (PROMs). Specific time points of the secondary outcome measures can be found in Table 1.

For peer review only - http://bmjopen.bmj.com/site/about/guidelines.xhtml

1. Ankle Osteoarthritis Score (AOS) is a visual analog scale from 0 – 100 mm with 18 questions; 9 relating to pain and 9 relating to disability.
2. Foot and Ankle Outcome Score (FAOS). Each question is assigned 0 – 4 points based on the answer given. The scale runs from 0 (extreme symptoms) to 100 points (no symptoms).
3. In order to evaluate pain, the pain sub-scale of AOFAS (0-40 points) will be analysed. On this scale the lower the score the more pain the patient has. Additionally a VAS score (VAS 0-100 mm) is measured during activities of daily living, with 0 mm being no pain and 100 mm the worst pain imaginable.
4. Total AOFAS score at the other time points than the primary one (at 6, 12 and 52 weeks as well as 5 years).
5. Ankle activity score (0-10 points) is scored according to chart based on the performable activity level
6. Subjective patient satisfaction (4 categories) Poor, Fair, Good, Excellent
7. Short Form Health Survey SF-36 is a health-related quality of life score (0-100 points). The higher the patient scores, the higher the disability.
8. The Global Attainment Scaling (GAS) is a method of scoring based on achievement related to pre-determined goals in agreement with the patient. Points are subtracted for not achieving the pre-defined goals or vice versa. Scores range from 100 (high functioning) to 0 (severely impaired)
9. EuroQol-5 dimensions-3 levels (EQ-5D-3L) utility score allows a patient's health to be defined by a 5-digit number.
10. PROductivity and DISease Questionnaire (PRODISQ) will be used to determine indirect costs and direct costs cost effectivity. The PRODISQ questionnaire is taken at baseline and every 3 months thereafter up until 1 year. This will be done in conjunction with the EQ-5D-3L.

Loss to follow-up

For peer review only

For peer review only - http://bmjopen.bmj.com/site/about/guidelines.xhtml

331 The coordinating researcher will attempt to limit loss to follow-up as much as possible by contacting every patient and being present at every patient visit. All digital questionnaires will be constantly monitored to ensure they are being filled in and otherwise followed-up by the coordinating researcher. In the event of patient withdrawal, an analysis of demographic and prognostic characteristics will be done on these cases and the remaining patients. As previously described by Järvinen et al. 2014, we will document the patient eligible for and compliant with each follow-up.[24]

For peer review only

For peer review only - http://bmjopen.bmj.com/site/about/guidelines.xhtml

Missing items

Missing items of a score will be handled according to the instructions of the specific scales. In the event of no instructions, we will calculate the percentage of missing items on a scale. Due to the potential impact on trial conclusions, multiple imputation (if more than 10% missing items on a scale) will be applied.

Sample Size

Based on previous and ongoing studies, the study protocol of the randomised controlled trial is designed to detect a difference of 12 points (0-100) on the AOFAS score. There is no official agreement on the minimal clinical important difference for the AOFAS score regarding ankle OA. However in relatable musculoskeletal literature, 10% – 15% of the used scale was reported. [13,25,26] Our pre-defined minimal clinical important difference of 12 % is located within this range.[13,25,26] Based on a previous placebo controlled RCT on injection therapy (hyaluronic acid) in ankle OA of De Groot et al. a standard deviation of 16.3 can be expected.[6] Taking into account a two-sided level of significance of 5%, a power of 90% and a dropout rate of 10%, approximately 50 (45 plus 10% drop out) patients per group will be needed (N=100 in total).

Data Management

After giving permission for participating in this study, patients will receive a link to fill in digital surveys. All data gained outside Castor EDC will be stored on the AMC secured hard drive. All data will be coded and stored in the Castor EDC online database which meets the AMC safety criteria and good clinical practice guidelines. The primary investigator and project leader will safeguard the coded data through password secured access. All patient's data will be archived for at least 15 years and handled with in accordance with the Dutch Personal Data Protection Act (Wbp). Data protections is provided through the safety protocol of Castor EDC with automated backups and SSL security.

Statistical analysis

A standard operating procedure will be available to logically recode and clean the data. The data will be interpreted according to a blinded data interpretation scheme described by Järvinen et al, 2014.[24]

For peer review only - http://bmjopen.bmj.com/site/about/guidelines.xhtml
For peer review only

A statistical expert (SB) is present among the authors. The authors will interpret the statistical results until a consensus is reached. Once the authors are in agreement the two groups will be unblinded and no changes will be made to the interpretation of the results.

Baseline characteristics

Baseline characteristics will be analyzed between groups using descriptive statistics.

Primary outcome measure

Analysis will be performed using an intention to treat approach. To test for the effect of treatment on the between-group difference in primary outcome, we will use a repeated measurement general linear model. Changes from baseline to all follow-up time points will be included in the model. Adjustments will be made for those baseline variables that influenced the primary outcome with $p < 0.10$.

Secondary outcome measures

To test for the effect of treatment on between-group differences in secondary outcomes, we will use the repeated measurement general linear model. Changes from baseline to all follow-up time points will be included in the model.

Economic analysis

In the event of a positive significant outcome, an economic analysis is needed to support a possible change of practice. An economic analysis (costs) will be performed in order to determine cost-effectiveness.

We will assess the differences in mean quality-adjusted life years (QALYs), costs, and net benefits between the PRP injection group and the placebo group using linear models. We express the cost-effectiveness by using cost-effectiveness acceptability curves from both a healthcare perspective and a societal perspective. With multiple bootstrap replicates of the average difference in costs and

For peer review only

For peer review only - http://bmjopen.bmj.com/site/about/guidelines.xhtml

effects in the incremental cost-effectiveness plane we will express the uncertainty of our cost-effectiveness analysis.

The cost-effectivity analysis will be performed with a 1-year time horizon. We use the three-level EQ-5D questionnaire (Euroqol, Rotterdam, the Netherlands) to calculate QALYs as the area under the curve of the utility scores measured over 12 months, according to the Dutch pricing system. The analysis will be based on indirect costs and direct costs and will be determined using the PRODISQ questionnaire. The PRODISQ questionnaire is taken at baseline and every 3 months thereafter up until 1 year.

Monitoring committee

The PRIMA trial will be monitored by the clinical research unit (CRU) of the coordination study centre. The CRU aims to improve the quality of clinical research and ultimately, patient care. Before the PRIMA trial commenced, a monitoring plan was set-up. This Monitoring plan facilitates compliance with the Human Research Act (WMO), Good Clinical Practice (ICH-GCP) guidelines (5.18.1) and/ or ISO14155, which require monitors to verify that (a) the rights and well-being of human subjects are protected,

(b) recorded study data is accurate, complete, and verifiable with the source documents,

(c) the conduct of the study is compliant with the currently approved protocol and with applicable laws regulatory requirements, e.g. WMO, ICH-GCP, ISO14155.

Throughout the study, monitoring will be performed at initiation of the trial, at each participating centre once approximately 3 patients have been enrolled, after approximately 10 – 15 enrolled patients have completed the 26 week follow-up, after 70 patients have been enrolled and after database lock.

For peer review only - http://bmjopen.bmj.com/site/about/guidelines.xhtml

Ethics and Dissemination

The study is approved by the local Medical Ethics Review Committee Amsterdam Medical Center, Amsterdam, the Netherlands (ABR 2018-042, approved 23-7-2018) and is registered in the Netherlands trial register (NTR 7261) in accordance with the World Health Organization Data Set (WHO Data Set). In the events of amendments or other changes, co-researchers will be notified. If relevant patients participating will also be notified. Intellectual data will be the property of the PRIMA study group. Participant data that underline the results reported in this article following de-identification will be shared anonymously on request following publication. Data will be shared, wherever legally and ethically possible and in line with ICMJE guidelines, with researchers who provide a methodologically sound proposal. Data will be stored in a repository (Figshare) under management of the medical library of the AMC. Any proposal should contain: Title, Background, Rationale, Objective, Methods (eligibility criteria, variables and timeframe of interest, statistical plan), Information regarding publication including authorship.

The results of this project study and new knowledge will be disseminated through the Dutch Arthritis Association (ReumaNederland), presentations, news publications, blogs, websites social media and professional organisations (rheumatology, orthopaedics, primary care medicine, sports medicine, public health) and the Dutch patient federation (Patiëntenfederatie Nederland). The patients will also be informed about the results of the study. The study will be conducted according to the principles of the Declaration of Helsinki, GCP and the Medical Research Involving Human Subjects Act (WMO). The board of each participating hospital reviewed and approved the local feasibility. Monitoring and auditing will be carried out throughout the study. In the event of Serious Adverse Events, these will be documented and reported to the monitor and the Medical Ethics board. Eligible patients will be informed of the study and will sign an informed consent form before participating (acquired by researchers associated with the study). Provisions for post-trial care as compensation for those who suffer harm from trial participation is documented in the patient information.

For peer review only - http://bmjopen.bmj.com/site/about/guidelines.xhtml

Discussion

This is the first study evaluating the efficacy and cost-effectiveness of Platelet-rich Plasma injections in the treatment of ankle OA with a multi-center, stratified, block-randomized, double-blind, placebo-controlled design. The primary outcome is the AOFAS score at 26 weeks. Our study will also evaluate the long term efficacy of PRP for up to five years.

Platelet-rich Plasma has been shown to alleviate symptoms of patients suffering from knee OA.[11] In the absence of effective evidence-based non-surgical interventions for ankle OA, PRP would fulfil a clear clinical need. Previous trials concerned with the efficacy of PRP for multiple indications are usually of low quality and adhere to a follow-up of 26 weeks or shorter. Furthermore a cost-effectiveness analysis has never been performed in previous trials on PRP in the treatment of OA.

A positive study outcome would have a significant impact leading to change in clinical practice, where PRP could be offered as a non-surgical intervention. A negative outcome will prevent the widespread use of a non-efficacious treatment.

This study has potential limitations. First of all, due to financial constraints, (semi-quantitative) MRI-defined cartilage degenerative changes will not be used as a secondary outcome score. Furthermore, the composition of PRP isnot analysed. This decision is based on the fact that PRP preparation and composition has frequently been analysed and is typically not performed in clinical practice prior to injection.[27] The PRP we use (Hettich Rotofix32 A centrifuge and Arthrex syringes) is one out of a multitude of commercially available PRP products. All these products differ in their production method and content, and different treatment regimens regarding dose, timing and number of injections are used in clinical practise. The generalizability to other PRP products and treatment regimens remains unknown.

Conclusion

For peer review only

For peer review only - http://bmjopen.bmj.com/site/about/guidelines.xhtml

This will be the first randomized controlled trial to assess the efficacy and cost-effectiveness of PRP in ankle OA.

Contributor ship statement

All authors have been actively involved in creating and writing the study protocol, and all authors critically commented on the paper and gave their final approval of the version that we submitted. *Authorship was and for future publications will be, determined according to the ICMJE authorship recommendations:*

“Substantial contributions to the conception or design of the work; the acquisition, analysis, or interpretation of data for the work;

L.D.A. Paget, S.M.A. Bierma – Zeinstra, S. Goedegebuure, G.M.M.J. Kerkhoffs, R. Krips, M. Maas, M. H. Moen, G. Reurink, S. A. S. Stufkens, R.J. de Vos, A. Weir, J.L. Tol

Drafting the work or revising it critically for important intellectual content;

L.D.A. Paget S.M.A. Bierma – Zeinstra, G.M.M.J. Kerkhoffs, G. Reurink, R.J. de Vos, A. Weir, J.L. Tol

Final approval of the version to be published;

L.D.A. Paget, S.M.A. Bierma – Zeinstra, S. Goedegebuure, G.M.M.J. Kerkhoffs, R. Krips, M. Maas, M. H. Moen, G. Reurink, S. A. S. Stufkens, R.J. de Vos, A. Weir, J.L. Tol

Agreement to be accountable for all aspects of the work in ensuring that questions related to the accuracy or integrity of any part of the work are appropriately investigated and resolved.

L.D.A. Paget, S.M.A. Bierma – Zeinstra, S. Goedegebuure, G.M.M.J. Kerkhoffs, R. Krips, M. Maas, M. H. Moen, G. Reurink, S. A. S. Stufkens, R.J. de Vos, A. Weir, J.L. Tol

For peer review only

For peer review only - http://bmjopen.bmj.com/site/about/guidelines.xhtml

Competing interests

All authors have completed the ICMJE uniform disclosure form at www.icmje.org/coi_disclosure.pdf and declare: no support from any organisation for the submitted work; no financial relationships with any organisations that might have an interest in the submitted work in the previous three years; no other relationships or activities that could appear to have influenced the submitted work.

Funding

This multicenter Randomized Controlled Trial was supported by a grant from the Dutch Arthritis Foundation (ReumaNederland). The Dutch Arthritis Foundation will not, and has not had any role regarding study design, collection, management, analysis, data interpretation or writing of the report. Furthermore, the Dutch Arthritis Foundation does not have authority over report submission or publication.

Figure legends/captions

Fig.1 illustrating the PRIMA multicentre randomized controlled trial design and follow-up procedure

For peer review only

For peer review only - http://bmjopen.bmj.com/site/about/guidelines.xhtml

References

1. Murray C, Marshall M, Rathod T, Bowen CJ, Menz HB, Roddy E. Population prevalence and distribution of ankle pain and symptomatic radiographic ankle osteoarthritis in community dwelling older adults: A systematic review and cross-sectional study. *PLoS One*. 2018. doi:10.1371/journal.pone.0193662

2. Saltzman CL. Impact of Comorbidities on the Measurement of Health in Patients with Ankle Osteoarthritis. *J Bone Jt Surg*. 2006;88(11):2366. doi:10.2106/JBJS.F.00295

3. Marx RE. Platelet-rich plasma (PRP): What is PRP and what is not PRP? *Implant Dent*. 2001. doi:10.1097/00008505-200110000-00002

4. Zhu Y, Yuan M, Meng HY, et al. Basic science and clinical application of platelet-rich plasma forcartilage defects and osteoarthritis: A review. *Osteoarthr Cartil*. 2013;21(11):1627-1637. doi:10.1016/j.joca.2013.07.017

5. Vannabouathong C, Del Fabbro G, Sales B, et al. Intra-articular Injections in the Treatment of Symptoms from Ankle Arthritis: A Systematic Review. *Foot Ankle Int*. 2018:107110071877937. doi:10.1177/1071100718779375

6. DeGroot H, Uzunishvili S, Weir R, Al-omari A, Gomes B. Intra-articular injection of hyaluronic acid is not superior to saline solution injection for ankle arthritis: a randomized, double-blind, placebo-controlled study. *J Bone Joint Surg Am*. 2012;94(1):2-8. doi:10.2106/JBJS.J.01763

7. Meheux CJ, McCulloch PC, Lintner DM, Varner KE, Harris JD. Efficacy of Intra-articular Platelet-Rich Plasma Injections in Knee Osteoarthritis: A Systematic Review. *Arthrosc - J Arthrosc Relat Surg*. 2016;32(3):495-505. doi:10.1016/j.arthro.2015.08.005

8. Laudy ABM, Bakker EWP, Rekers M, Moen MH. Efficacy of platelet-rich plasma injections in osteoarthritis of the knee: a systematic review and meta-analysis. *Br J Sports Med*. 2015;49(10):657-672. doi:10.1136/bjsports-2014-094036

9. Moen M, Weir A, Bakker E, Rekers M, Laudy G. Efficacy of platelet-rich plasma injections in osteoarthritis of the knee: an updated systematic review and meta-analysis. *Osteoarthr Cartil*. 2016;24:S520-S521. doi:10.1016/j.joca.2016.01.953

10. Xing D, Wang B, Zhang W, et al. Intra-articular platelet-rich plasma injections for knee osteoarthritis: An overview of systematic reviews and risk of bias considerations. *Int J Rheum Dis*. 2017;20(11):1612-1630. doi:10.1111/1756-185X.13233

11. Dai WL, Zhou AG, Zhang H, Zhang J. Efficacy of Platelet-Rich Plasma in the Treatment of Knee Osteoarthritis: A Meta-analysis of Randomized Controlled Trials. *Arthrosc - J Arthrosc Relat Surg*. 2017. doi:10.1016/j.arthro.2016.09.024

12. Reurink G, Goudswaard GJ, Moen MH, et al. Platelet-Rich Plasma Injections in Acute

For peer review only

For peer review only - http://bmjopen.bmj.com/site/about/guidelines.xhtml

Muscle Injury. *N Engl J Med*. 2014;370(26):2546-2547. doi:10.1056/NEJMc1402340

13. de Vos RJ, Weir A, van Schie HTM, et al. Platelet-rich plasma injection for chronic Achilles tendinopathy: a randomized controlled trial. *JAMA*. 2010;303(2):144-149. doi:10.1001/jama.2009.1986

14. Fukawa T, Yamaguchi S, Akatsu Y, Yamamoto Y, Akagi R, Sasho T. Safety and Efficacy of Intra-articular Injection of Platelet-Rich Plasma in Patients With Ankle Osteoarthritis. *Foot Ankle Int*. 2017;38(6):596-604. doi:10.1177/1071100717700377

15. Repetto I, Biti B, Cerruti P, Trentini R, Felli L. Conservative Treatment of Ankle Osteoarthritis: Can Platelet-Rich Plasma Effectively Postpone Surgery? *J Foot Ankle Surg*. 2017;56(2):362-365. doi:10.1053/j.jfas.2016.11.015

16. Pas HI, Reurink G, Tol JL, Weir A, Winters M, Moen MH. Efficacy of rehabilitation (lengthening) exercises, platelet-rich plasma injections, and other conservative interventions in acute hamstring injuries: an updated systematic review and meta-analysis. *Br J Sports Med*. 2015;49(18):1197-1205. doi:10.1136/bjsports-2015-094879

17. Hamilton B, Tol JL, Almusa E, et al. Platelet-rich plasma does not enhance return to play in hamstring injuries: a randomised controlled trial. *Br J Sports Med*. 2015;49(14):943-950. doi:10.1136/bjsports-2015-094603

18. Angthong C, Khadsongkram A, Angthong W. Outcomes and quality of life after platelet-rich plasma therapy in patients with recalcitrant hindfoot and ankle diseases: A preliminary report of 12 patients. *J Foot Ankle Surg*. 2013;52(4):475-480. doi:10.1053/j.jfas.2013.04.005

19. van Dijk CN, Verhagen R a, Tol JL. Arthroscopy for problems after ankle fracture. *J Bone Jt Surg*. 1997;79-B(2):280-284. doi:10.1302/0301-620X.79B2.7153

20. De Boer AS, Tjioe RJC, Van Der Sijde F, et al. The American Orthopaedic Foot and Ankle Society Ankle-Hindfoot Scale; Translation and validation of the Dutch language version for ankle fractures. *BMJ Open*. 2017;7(8):1-12. doi:10.1136/bmjopen-2017-017040

21. Kitaoka HB, Alexander IJ, Adelaar RS, Nunley JA, Myerson MS, Sanders M. Clinical rating systems for the ankle-hindfoot, midfoot, hallux, and lesser toes. *Foot ankle Int*. 1994;15(7):349-353. doi:10.1177/107110079401500701

22. Sayyed-Hosseinian S-H, Hassankhani GG, Bagheri F, Alavi N, Shojaie B, Mousavian A. Validation of the Persian Version of the American Orthopedic Foot and Ankle Society Score (AOFAS) Questionnaire. *Arch bone Jt Surg*. 2018;6(3):233-239. doi:10.1097/PHM.0000000000000234

23. Vosoughi AR, Roustaei N, Mahdaviazad H. American Orthopaedic Foot and Ankle Society ankle–hindfoot scale: A cross-cultural adaptation and validation study from Iran. *Foot Ankle Surg*. 2018. doi:10.1016/j.fas.2017.02.007

For peer review only

For peer review only - http://bmjopen.bmj.com/site/about/guidelines.xhtml

24. Järvinen TLN, Sihvonen R, Bhandari M, et al. Blinded interpretation of study results can feasibly and effectively diminish interpretation bias. *J Clin Epidemiol*. 2014;67(7):769-772. doi:10.1016/j.jclinepi.2013.11.011

25. Pham T, van der Heijde D, Altman RD, et al. OMERACT-OARSI initiative: Osteoarthritis research society international set of responder criteria for osteoarthritis clinical trials revisited. *Osteoarthr Cartil*. 2004. doi:10.1016/j.joca.2004.02.001

26. Rozendaal RM, Koes BW, Van Osch GJVM, et al. Effect of glucosamine sulfate on hip osteoarthritis: A randomized trial. *Ann Intern Med*. 2008. doi:10.7326/0003-4819-148-4-200802190-00005

27. Oudelaar BW, Peerbooms JC, Huis in 't Veld R, Vochteloo AJH. Concentrations of Blood Components in Commercial Platelet-Rich Plasma Separation Systems: A Review of the Literature. *Am J Sports Med*. 2018. doi:10.1177/0363546517746112

For peer review only

For peer review only - http://bmjopen.bmj.com/site/about/guidelines.xhtml

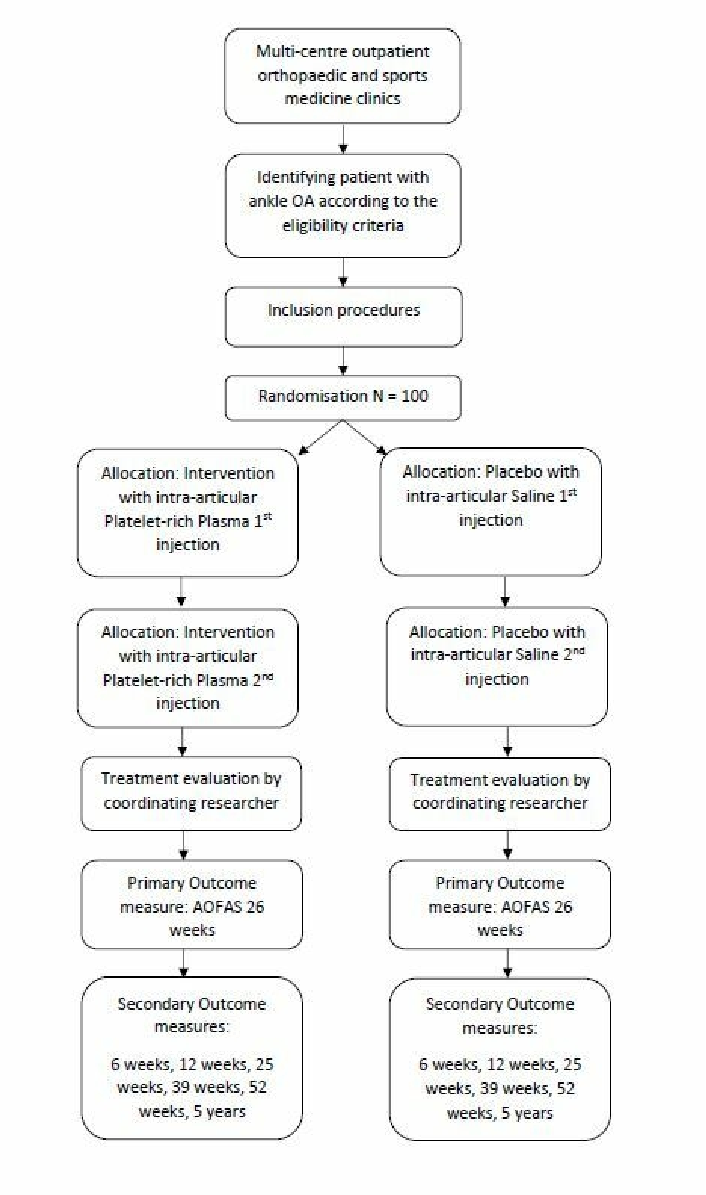

Fig.1 illustrating the PRIMA multicentre randomized controlled trial design and follow-up procedure

39x67mm (300 x 300 DPI)

Surveys of PRIMA trial - version 83.01

Printed on 16-09-2019 11:34:13 by Liam Paget

Survey 'Vragenlijst PRIMA studie'

Vragenlijst PRIMA studie - AOFAS - Pijn

Number	Question	Answers
1.1	Hoeveel pijn ervaart u aan uw voet??	☐ Geen<br>☐ Milde pijnklachten of af en toe pijn<br>☐ Matige pijnklachten, dagelijks<br>☐ Vrijwel continu erge pijn

Vragenlijst PRIMA studie - AOFAS resterende gedeelte

Number	Question	Answers
2.1	Hoeveel beperkingen in het dagelijks leven ervaart u?	○ Geen beperkingen, geen hulpmiddelen nodig<br>○ Geen beperkingen in algemeen dagelijkse bezigheden, wel beperkingen in recreatieve activiteiten (sport, hobbies, etc) geen ondersteuning<br>○ Beperkingen bij dagelijkse- en recreatieve activiteiten, stok<br>○ Ernstige beperkingen bij dagelijkse- en recreatieactiviteiten, walker, krukken, rolstoel, brace
2.2	Wat is de afstand die u in één keer kunt lopen?	○ Meer dan 600 meter<br>○ 400 - 600 meter<br>○ 100 - 300 meter<br>○ Minder dan 100 meter
2.3	Heeft u moeite met lopen op een bepaalde ondergrond?	○ Geen problemen bij lopen<br>○ Enig problemen op ongelijke ondergrond, trap, helling, ladders<br>○ Ernstige problemen bij ongelijke ondergrond, trap, helling, ladders

2.4	Hoe zou u uw looppatroon beoordelen?	◯ Normaal of minimaal afwijkend<br>◯ Duidelijk<br>◯ Ernstig
2.5	Hoe zou u de beweeglijkheid van uw voet beoordelen ten opzicht van de andere zijde of ten opzicte van vóór het ongeval?	◯ Hetzelfde of licht beperkt t.o.v. de niet aangedane zijde of zoals voor het ongeval<br>◯ Ongeveer de helft beperkt<br>◯ Sterk verminderd, bijna geen bewegingen mogelijk
2.6	Hoe zou u de beweeglijkheid van uw enkel beoordelen ten opzichte van de andere zijde of ten opzichte van voor het ongeval?	◯ Hetzelfde of licht beperkt t.o.v. de niet aangedane zijde of zoals voor het ongeval<br>◯ Ongeveer de helft beperkt<br>◯ Sterk verminderd, bijna geen bewegingen mogelijk
2.7	Hoe zou u de stabiliteit van uw voet en enkel beoordelen	◯ Stabiel, ik verzwik mijn enkel hooguit incidenteel<br>◯ Niet stabiel, ik verzwik mijn enkel vaak
2.8	Hoe zou u de stand van uw voet beoordelen ten opzichte van de andere zijde of ten opzichte van voor het ongeval?	◯ Goed, ik kan mijn voet goed plat op de grond zetten, waarbij de stand niet afwijkend is<br>◯ Ik sta vrijwel alleen op de buitenzijde van mijn voet<br>◯ Ik kan mijn voet niet plat op de grond zetten en loop vrijwel alleen op mijn voorvoet

Vragenlijst PRIMA studie - VAS Pain score

Number	Question	Answers	
3.1	VAS pijnscore (Geef uw minimaal pijnscore aan tijdens dagelijkse activiteiten op een schaal van 0 tot 100, waarbij 0 geen pijn is en 100 de ergste pijn denkbaar is)	(0.00)	100 (100.00)

Vragenlijst PRIMA studie - AAS

Number	Question	Answers

4.1	Wat is het meest bij u van toepassing ten aanzien van uw acitviteit?	☐ American Football<br>☐ Basketbal<br>☐ Gymnastiek/turnen<br>☐ Handbal<br>☐ Rugby<br>☐ Voetbal<br>☐ Hockey<br>☐ Korfbal<br>☐ Gevechtssporten: judo, karate, kung fu, taekwando, jiu jitsu, etc.<br>☐ Oriëntatielopen<br>☐ Rhytmische gymnastiek<br>☐ Volleybal<br>☐ Boxen<br>☐ Freestyle snowboarden<br>☐ Ijshockey<br>☐ Tennis<br>☐ Worstelen<br>☐ Fitness, aerobics<br>☐ Badminton<br>☐ Baseball<br>☐ Cross-country hardlopen<br>☐ Moderne pentathlon<br>☐ Squash<br>☐ Surfen, windsurfen<br>☐ Tafel tennis<br>☐ Atletiek: spring-, werponderdelen<br>☐ Waterskieën<br>☐ Dans<br>☐ Schermen<br>☐ Zaalhockey<br>☐ Bergbeklimmen<br>☐ Langlauf<br>☐ Parachute springen<br>☐ Softball<br>☐ Speciale beroepen en werkactiviteiten; speciale beroepen: ballet, professioneel soldaat, speciale reddingswerker, stuntman, etc.<br>☐ Duiken<br>☐ Scubaduiken<br>☐ Skaten, in-line skaten<br>☐ Atletiek: looponderdelen<br>☐ Triatlon<br>☐ Gewichtsheffen, body-building

- ☐ Alle competitieve sporten hieronder met ‘seasonal’ conditioning
- ☐ Zwaar fysiek werk
- ☐ Alpine skiën en snowboarden
- ☐ Bowlen/curlen
- ☐ Golf
- ☐ Mountainbike/BMX
- ☐ Powerliften
- ☐ Zeilen
- ☐ Fysiek werk
- ☐ Wielrennen
- ☐ Paardrijden
- ☐ Motorsporten/technische sporten
- ☐ Roeien, kayakken
- ☐ Boogschieten
- ☐ Water polo en zwemmen
- ☐ Kunnen lopen op oneven grond
- ☐ Geen sport, echter geen limitatie in dagelijkse activiteiten
- ☐ Kunnen lopen op even grond, maar de dagelijkste activiteiten zijn gelimiteerd
- ☐ Niet kunnen lopen, fysieke handicap wegens enkelproblemen

---

4.1.1 ***If 'Wat is het meest bij u van toepassing ten aanzien van uw acitviteit?' is equal to 'American Football' answer this question:***
American Football

- ○ Professioneel
- ○ Competitief
- ○ Recreatief

4.1.2 ***If 'Wat is het meest bij u van toepassing ten aanzien van uw acitviteit?' is equal to 'Basketbal' answer this question:***
Basketbal

- ○ Professioneel
- ○ Competitief
- ○ Recreatief

4.1.3 ***If 'Wat is het meest bij u van toepassing ten aanzien van uw acitviteit?' is equal to 'Gymnastiek/turnen' answer this question:***
Gymnastiek/turnen

- ○ Professioneel
- ○ Competitief
- ○ Recreatief

4.1.4 ***If 'Wat is het meest bij u van toepassing ten aanzien van uw acitviteit?' is equal to 'Handbal' answer this question:***
Handbal

- ○ Professioneel
- ○ Competitief
- ○ Recreatief

4.1.5 ***If 'Wat is het meest bij u van toepassing ten aanzien van uw acitviteit?' is equal to 'Rugby' answer this question:***
Rugby

- ○ Professioneel
- ○ Competitief
- ○ Recreatief

For peer review only - http://bmjopen.bmj.com/site/about/guidelines.xhtml

4.1.6	***If 'Wat is het meest bij u van toepassing ten aanzien van uw acitviteit?' is equal to 'Voetbal' answer this question:***<br>Voetbal	◯ Professioneel<br>◯ Competitief<br>◯ Recreatief
4.1.7	***If 'Wat is het meest bij u van toepassing ten aanzien van uw acitviteit?' is equal to 'Hockey' answer this question:***<br>Hockey	◯ Professioneel<br>◯ Competitief<br>◯ Recreatief
4.1.8	***If 'Wat is het meest bij u van toepassing ten aanzien van uw acitviteit?' is equal to 'Korfbal' answer this question:***<br>Korfbal	◯ Professioneel<br>◯ Competitief<br>◯ Recreatief
4.1.9	***If 'Wat is het meest bij u van toepassing ten aanzien van uw acitviteit?' is equal to 'Gevechtssporten: judo, karate, kung fu, taekwando, jiu jitsu, etc.' answer this question:***<br>Gevechtssporten: judo, karate, kung fu, taekwando, jiu jitsu, etc.	◯ Professioneel<br>◯ Competitief<br>◯ Recreatief
4.1.10	***If 'Wat is het meest bij u van toepassing ten aanzien van uw acitviteit?' is equal to 'Oriëntatielopen' answer this question:***<br>Oriëntatielopen	◯ Professioneel<br>◯ Competitief<br>◯ Recreatief
4.1.11	***If 'Wat is het meest bij u van toepassing ten aanzien van uw acitviteit?' is equal to 'Rhytmische gymnastiek' answer this question:***<br>Rhytmisch gymnastiek	◯ Professioneel<br>◯ Competitief<br>◯ Recreatief
4.1.12	***If 'Wat is het meest bij u van toepassing ten aanzien van uw acitviteit?' is equal to 'Volleybal' answer this question:***<br>Volleybal	◯ Professioneel<br>◯ Competitief<br>◯ Recreatief
4.1.13	***If 'Wat is het meest bij u van toepassing ten aanzien van uw acitviteit?' is equal to 'Boxen' answer this question:***<br>Boxen	◯ Professioneel<br>◯ Competitief<br>◯ Recreatief
4.1.14	***If 'Wat is het meest bij u van toepassing ten aanzien van uw acitviteit?' is equal to 'Freestyle snowboarden' answer this question:***<br>Freestyle snowboarden	◯ Professioneel<br>◯ Competitief<br>◯ Recreatief
4.1.15	***If 'Wat is het meest bij u van toepassing ten aanzien van uw acitviteit?' is equal to 'Freestyle snowboarden' answer this question:***<br>IJshockey	◯ Professioneel<br>◯ Competitief<br>◯ Recreatief

4.1.16	***If 'Wat is het meest bij u van toepassing ten aanzien van uw acitviteit?' is equal to 'Tennis' answer this question:***<br>Tennis	◯ Professioneel<br>◯ Competitief<br>◯ Recreatief
4.1.17	***If 'Wat is het meest bij u van toepassing ten aanzien van uw acitviteit?' is equal to 'Worstelen' answer this question:***<br>Worstelen	◯ Professioneel<br>◯ Competitief<br>◯ Recreatief
4.1.18	***If 'Wat is het meest bij u van toepassing ten aanzien van uw acitviteit?' is equal to 'Fitness, aerobics' answer this question:***<br>Fitness, aerobics	◯ Professioneel<br>◯ Competitief<br>◯ Recreatief
4.1.19	***If 'Wat is het meest bij u van toepassing ten aanzien van uw acitviteit?' is equal to 'Badminton' answer this question:***<br>Badminton	◯ Professioneel<br>◯ Competitief<br>◯ Recreatief
4.1.20	***If 'Wat is het meest bij u van toepassing ten aanzien van uw acitviteit?' is equal to 'Baseball' answer this question:***<br>Baseball	◯ Professioneel<br>◯ Competitief<br>◯ Recreatief
4.1.21	***If 'Wat is het meest bij u van toepassing ten aanzien van uw acitviteit?' is equal to 'Cross-country hardlopen' answer this question:***<br>Cross-country hardlopen	◯ Professioneel<br>◯ Competitief<br>◯ Recreatief
4.1.22	***If 'Wat is het meest bij u van toepassing ten aanzien van uw acitviteit?' is equal to 'Moderne pentathlon' answer this question:***<br>Moderne pentathlon	◯ Professioneel<br>◯ Competitief<br>◯ Recreatief
4.1.23	***If 'Wat is het meest bij u van toepassing ten aanzien van uw acitviteit?' is equal to 'Squash' answer this question:***<br>Squash	◯ Professioneel<br>◯ Competitief<br>◯ Recreatief
4.1.24	***If 'Wat is het meest bij u van toepassing ten aanzien van uw acitviteit?' is equal to 'Surfen, windsurfen' answer this question:***<br>Surfen, windsurfen	◯ Professioneel<br>◯ Competitief<br>◯ Recreatief
4.1.25	***If 'Wat is het meest bij u van toepassing ten aanzien van uw acitviteit?' is equal to 'Tafel tennis' answer this question:***<br>Tafel tennis	◯ Professioneel<br>◯ Competitief<br>◯ Recreatief

4.1.26	***If 'Wat is het meest bij u van toepassing ten aanzien van uw acitviteit?' is equal to 'Atletiek: spring-, werponderdelen' answer this question:***<br>Atletiek: spring-, werponderdelen	◯ Professioneel<br>◯ Competitief<br>◯ Recreatief
4.1.27	***If 'Wat is het meest bij u van toepassing ten aanzien van uw acitviteit?' is equal to 'Waterskieën' answer this question:***<br>Waterskien	◯ Professioneel<br>◯ Competitief<br>◯ Recreatief
4.1.28	***If 'Wat is het meest bij u van toepassing ten aanzien van uw acitviteit?' is equal to 'Dans' answer this question:***<br>Dans	◯ Professioneel<br>◯ Competitief<br>◯ Recreatief
4.1.29	***If 'Wat is het meest bij u van toepassing ten aanzien van uw acitviteit?' is equal to 'Schermen' answer this question:***<br>Schermen	◯ Professioneel<br>◯ Competitief<br>◯ Recreatief
4.1.30	***If 'Wat is het meest bij u van toepassing ten aanzien van uw acitviteit?' is equal to 'Zaalhockey' answer this question:***<br>Zaalhockey	◯ Professioneel<br>◯ Competitief<br>◯ Recreatief
4.1.31	***If 'Wat is het meest bij u van toepassing ten aanzien van uw acitviteit?' is equal to 'Bergbeklimmen' answer this question:***<br>Bergbeklimmen	◯ Professioneel<br>◯ Competitief<br>◯ Recreatief
4.1.32	***If 'Wat is het meest bij u van toepassing ten aanzien van uw acitviteit?' is equal to 'Langlauf' answer this question:***<br>Langlauf	◯ Professioneel<br>◯ Competitief<br>◯ Recreatief
4.1.33	***If 'Wat is het meest bij u van toepassing ten aanzien van uw acitviteit?' is equal to 'Parachute springen' answer this question:***<br>Parachute springen	◯ Professioneel<br>◯ Competitief<br>◯ Recreatief
4.1.34	***If 'Wat is het meest bij u van toepassing ten aanzien van uw acitviteit?' is equal to 'Softball' answer this question:***<br>Softball	◯ Professioneel<br>◯ Competitief<br>◯ Recreatief

4.1.35	***If 'Wat is het meest bij u van toepassing ten aanzien van uw acitviteit?' is equal to 'Speciale beroepen en werkactiviteiten; speciale beroepen: ballet, professioneel soldaat, speciale reddingswerker, stuntman, etc.' answer this question:***<br>Speciale beroepen en werkactiviteiten speciale beroepen: ballet, professioneel soldaat, speciale reddingswerker, stuntman, etc.	◯ ballet, professioneel soldaat, speciale reddingswerker, stuntman, etc.
4.1.36	***If 'Wat is het meest bij u van toepassing ten aanzien van uw acitviteit?' is equal to 'Duiken' answer this question:***<br>Duiken	◯ Professioneel/competitief<br>◯ Recreatief
4.1.37	***If 'Wat is het meest bij u van toepassing ten aanzien van uw acitviteit?' is equal to 'Scubaduiken' answer this question:***<br>Scubaduiken	◯ Professioneel/competitief<br>◯ Recreatief
4.1.38	***If 'Wat is het meest bij u van toepassing ten aanzien van uw acitviteit?' is equal to 'Skaten, in-line skaten' answer this question:***<br>skaten, in-linen skaten	◯ Professioneel/competitief<br>◯ Recreatief
4.1.39	***If 'Wat is het meest bij u van toepassing ten aanzien van uw acitviteit?' is equal to 'Atletiek: looponderdelen' answer this question:***<br>Atletiek: looponderdelen	◯ Professioneel/competitief<br>◯ Recreatief
4.1.40	***If 'Wat is het meest bij u van toepassing ten aanzien van uw acitviteit?' is equal to 'Triatlon' answer this question:***<br>triatlon	◯ Professioneel/competitief<br>◯ Recreatief
4.1.41	***If 'Wat is het meest bij u van toepassing ten aanzien van uw acitviteit?' is equal to 'Gewichtsheffen, body-building' answer this question:***<br>Gewichtsheffen, body-building	◯ Professioneel/competitief<br>◯ Recreatief
4.1.42	***If 'Wat is het meest bij u van toepassing ten aanzien van uw acitviteit?' is equal to 'Alle competitieve sporten hieronder met ‘seasonal’ conditioning' answer this question:***<br>Alle competitieve sporten hieronder met ‘seasonal’ conditioning	◯ Alle competitieve sporten hieronder met ‘seasonal’ conditioning
4.1.43	***If 'Wat is het meest bij u van toepassing ten aanzien van uw acitviteit?' is equal to 'Zwaar fysiek werk' answer this question:***<br>Zwaar fysiek werk	◯ Alle competitieve sporten hieronder met ‘seasonal’ conditioning

4.1.44	***If 'Wat is het meest bij u van toepassing ten aanzien van uw acitviteit?' is equal to 'Alpine skiën en snowboarden' answer this question:*** Alpine skien en snowboarden	◯ Alpine skien en snowboarden
4.1.45	***If 'Wat is het meest bij u van toepassing ten aanzien van uw acitviteit?' is equal to 'Bowlen/curlen' answer this question:*** Bowlen/curlen	◯ Bowlen/curlen
4.1.46	***If 'Wat is het meest bij u van toepassing ten aanzien van uw acitviteit?' is equal to 'Golf' answer this question:*** Golf	◯ Golf
4.1.47	***If 'Wat is het meest bij u van toepassing ten aanzien van uw acitviteit?' is equal to 'Mountainbike/BMX' answer this question:*** Mountainbike/BMX	◯ Mountainbike/BMX
4.1.48	***If 'Wat is het meest bij u van toepassing ten aanzien van uw acitviteit?' is equal to 'Powerliften' answer this question:*** Powerliften	◯ Powerliften
4.1.49	***If 'Wat is het meest bij u van toepassing ten aanzien van uw acitviteit?' is equal to 'Zeilen' answer this question:*** Zeilen	◯ Zeilen
4.1.50	***If 'Wat is het meest bij u van toepassing ten aanzien van uw acitviteit?' is equal to 'Fysiek werk' answer this question:*** Fysiek werk	◯ Fysiek werk
4.1.51	***If 'Wat is het meest bij u van toepassing ten aanzien van uw acitviteit?' is equal to 'Wielrennen' answer this question:*** Wielrennen	◯ Wielrennen
4.1.52	***If 'Wat is het meest bij u van toepassing ten aanzien van uw acitviteit?' is equal to 'Paardrijden' answer this question:*** Paardrijden	◯ Paardrijden
4.1.53	***If 'Wat is het meest bij u van toepassing ten aanzien van uw acitviteit?' is equal to 'Motorsporten/technische sporten' answer this question:*** Motorsporten/technische sporten	◯ Motorsporten/technische sporten
4.1.54	***If 'Wat is het meest bij u van toepassing ten aanzien van uw acitviteit?' is equal to 'Roeien, kayakken' answer this question:*** Roeien, kayakken	◯ Roeien, kayakken

4.1.55	***If 'Wat is het meest bij u van toepassing ten aanzien van uw acitviteit?' is equal to 'Boogschieten' answer this question:*** Boogschieten	○ Boogschieten
4.1.56	***If 'Wat is het meest bij u van toepassing ten aanzien van uw acitviteit?' is equal to 'Water polo en zwemmen' answer this question:*** Water polo en zwemmen	○ Water polo en zwemmen
4.1.57	***If 'Wat is het meest bij u van toepassing ten aanzien van uw acitviteit?' is equal to 'Kunnen lopen op oneven grond' answer this question:*** Kunnen lopen op oneven grond	○ Kunnen lopen op oneven grond
4.1.58	***If 'Wat is het meest bij u van toepassing ten aanzien van uw acitviteit?' is equal to 'Geen sport, echter geen limitatie in dagelijkse activiteiten' answer this question:*** Geen sport, echter geen limitatie in dagelijkse activiteiten	○ Geen sport, echter geen limitatie in dagelijkse activiteiten
4.1.59	***If 'Wat is het meest bij u van toepassing ten aanzien van uw acitviteit?' is equal to 'Kunnen lopen op even grond, maar de dagelijkste activiteiten zijn gelimiteerd' answer this question:*** Kunnen lopen op even grond, maar de dagelijkste activiteiten zijn gelimiteerd	○ Kunnen lopen op even grond, maar de dagelijkste activiteiten zijn gelimiteerd
4.1.60	***If 'Wat is het meest bij u van toepassing ten aanzien van uw acitviteit?' is equal to 'Niet kunnen lopen, fysieke handicap wegens enkelproblemen' answer this question:*** Niet kunnen lopen, fysieke handicap wegens enkelproblemen	○ Niet kunnen lopen, fysieke handicap wegens enkelproblemen

Vragenlijst PRIMA studie - Hoe tevreden bent u over de enkelklachten?

Number	Question	Answers
5.1	Hoe tevreden bent u over de enkelklachten?	○ Slecht<br>○ Matig<br>○ Goed<br>○ Uitstekend

Vragenlijst PRIMA studie - SF-36

Number	Question	Answers
	Deze vragenlijst gaat over uw standpunten t.a.v. uw gezondheid. Met behulp van deze gegevens kan worden bijgehouden hoe u zich voelt en hoe goed u in staat bent uw gebruikelijke bezigheden uit te voeren. Beantwoord elke vraag door een antwoord aan te klikken. Als u niet zeker weet hoe u een vraag moet beantwoorden, geef dan het best mogelijke antwoord.	
6.1	Hoe zou u over het algemeen uw gezondheid noemen?	◯ Uitstekend ◯ Zeer goed ◯ Goed ◯ Matig ◯ Slecht
6.2	Hoe beoordeelt u nu uw gezondheid over het algemeen vergeleken met een jaar geleden?	◯ Veel beter dan een jaar geleden ◯ Wat beter dan een jaar geleden ◯ Ongeveer hetzelfde als een jaar geleden ◯ Wat slechter dan een jaar geleden ◯ Veel slechter dan een jaar geleden
6.3	Wordt u door uw gezondheid op dit moment beperkt bij forse inspanning, zoals hardlopen, tillen van zware voorwerpen of een veeleisende sport beoefenen?	◯ Ja, ernstig beperkt ◯ Ja, een beetje beperkt ◯ Nee, helemaal niet beperkt
6.4	Wordt u door uw gezondheid op dit moment beperkt bij matige inspanning zoals een tafel verplaatsen, stofzuigen, zwemmen of fietsen?	◯ Ja, ernstig beperkt ◯ Ja, een beetje beperkt ◯ Nee, helemaal niet beperkt
6.5	Wordt u door uw gezondheid op dit moment beperkt bij boodschappen tillen of dragen?	◯ Ja, ernstig beperkt ◯ Ja, een beetje beperkt ◯ Nee, helemaal niet beperkt
6.6	Wordt u door uw gezondheid op dit moment beperkt bij een paar trappen oplopen?	◯ Ja, ernstig beperkt ◯ Ja, een beetje beperkt ◯ Nee, helemaal niet beperkt
6.7	Wordt u door uw gezondheid op dit moment beperkt bij één trap oplopen?	◯ Ja, ernstig beperkt ◯ Ja, een beetje beperkt ◯ Nee, helemaal niet beperkt
6.8	Wordt u door uw gezondheid op dit moment beperkt bij bukken, knielen of hurken?	◯ Ja, ernstig beperkt ◯ Ja, een beetje beperkt ◯ Nee, helemaal niet beperkt
6.9	Wordt u door uw gezondheid op dit moment beperkt bij meer dan één kilometer lopen?	◯ Ja, ernstig beperkt ◯ Ja, een beetje beperkt ◯ Nee, helemaal niet beperkt
6.10	Wordt u door uw gezondheid op dit moment beperkt bij een paar honderd meter lopen?	◯ Ja, ernstig beperkt ◯ Ja, een beetje beperkt ◯ Nee, helemaal niet beperkt

6.11	Wordt u door uw gezondheid op dit moment beperkt bij ongeveer honderd meter lopen?	◯ Ja, ernstig beperkt ◯ Ja, een beetje beperkt ◯ Nee, helemaal niet beperkt
6.12	Wordt u door uw gezondheid op dit moment beperkt bij uzelf wassen of aankleden?	◯ Ja, ernstig beperkt ◯ Ja, een beetje beperkt ◯ Nee, helemaal niet beperkt
6.13	U besteedde in de afgelopen 4 weken minder tijd aan werk of andere bezigheden	◯ Ja ◯ Nee
6.14	U heeft in de afgelopen 4 weken minder bereikt dan u zou willen	◯ Ja ◯ Nee
6.15	U was in de afgelopen 4 weken beperkt in het soort werk of andere bezigheden.	◯ Ja ◯ Nee
6.16	U had de afgelopen 4 weken moeite om uw werk of andere bezigheden uit te voeren (het kostte u bijvoorbeeld extra inspanning).	◯ Ja ◯ Nee
6.17	U besteedde in de afgelopen 4 weken minder tijd aan werk of andere bezigheden ten gevolge van emotionele problemen	◯ Ja ◯ Nee
6.18	U heeft in de afgelopen 4 weken minder bereikt dan u zou willen ten gevolge van emotionele problemen.	◯ Ja ◯ Nee
6.19	U deed de afgelopen 4 weken uw werk of andere bezigheden niet zo zorgvuldig als gewoonlijk ten gevolge van emotionele problemen.	◯ Ja ◯ Nee
6.20	In hoeverre hebben uw lichamelijke gezondheid of emotionele problemen u gedurende de afgelopen 4 weken gehinderd in uw normale omgang met familie, vrienden of buren, of bij activiteiten in groepsverband?	◯ Helemaal niet ◯ Enigszins ◯ Nogal ◯ Veel ◯ Heel erg veel
6.21	Hoeveel lichamelijke pijn heeft u de afgelopen 4 weken gehad?	◯ Geen<br>◯ Heel licht<br>◯ Licht<br>◯ Nogal<br>◯ Ernstig<br>◯ Heel ernstig
6.22	In welke mate bent u de afgelopen 4 weken door de pijn gehinderd in uw normale werk (zowel werk buitenshuis als huishoudelijk werk)?	◯ Helemaal niet<br>◯ Een klein beetje<br>◯ Nogal<br>◯ Veel<br>◯ Heel erg veel

6.23	Voelde u zich levenslustig?	◯ Altijd<br>◯ Meestal<br>◯ Vaak<br>◯ Soms<br>◯ Zelden<br>◯ Nooit
6.24	Was u erg zenuwachtig?	◯ Altijd<br>◯ Meestal<br>◯ Vaak<br>◯ Soms<br>◯ Zelden<br>◯ Nooit
6.25	Zat u zo in de put dat u niets kon?	◯ Altijd<br>◯ Meestal<br>◯ Vaak<br>◯ Soms<br>◯ Zelden<br>◯ Nooit
6.26	Voelde u zich rustig en tevreden?	◯ Altijd<br>◯ Meestal<br>◯ Vaak<br>◯ Soms<br>◯ Zelden<br>◯ Nooit
6.27	Had u veel energie?	◯ Altijd<br>◯ Meestal<br>◯ Vaak<br>◯ Soms<br>◯ Zelden<br>◯ Nooit
6.28	Voelde u zich somber en neerslachtig?	◯ Altijd<br>◯ Meestal<br>◯ Vaak<br>◯ Soms<br>◯ Zelden<br>◯ Nooit

6.29	Voelde u zich uitgeput?	◯ Altijd<br>◯ Meestal<br>◯ Vaak<br>◯ Soms<br>◯ Zelden<br>◯ Nooit
6.30	Was u een gelukkig mens?	◯ Altijd<br>◯ Meestal<br>◯ Vaak<br>◯ Soms<br>◯ Zelden<br>◯ Nooit
6.31	Voelde u zich moe?	◯ Altijd<br>◯ Meestal<br>◯ Vaak<br>◯ Soms<br>◯ Zelden<br>◯ Nooit
6.32	Hoe vaak hebben uw lichamelijke gezondheid of emotionele problemen u gedurende de afgelopen 4 weken gehinderd bij uw sociale activiteiten (zoals vrienden of familie bezoeken etc.)?	◯ Altijd<br>◯ Meestal<br>◯ Soms<br>◯ Zelden<br>◯ Nooit
6.33	Ik lijk wat gemakkelijker ziek te worden dan andere mensen	◯ Volkomen juist<br>◯ Grotendeels juist<br>◯ Weet ik niet<br>◯ Grotendeels onjuist<br>◯ Volkomen onjuist
6.34	Ik ben even gezond als andere mensen die ik ken	◯ Volkomen juist<br>◯ Grotendeels juist<br>◯ Weet ik niet<br>◯ Grotendeels onjuist<br>◯ Volkomen onjuist

6.35	Ik verwacht dat mijn gezondheid achteruit zal gaan	◯ Volkomen juist<br>◯ Grotendeels juist<br>◯ Weet ik niet<br>◯ Grotendeels onjuist<br>◯ Volkomen onjuist
6.36	Mijn gezondheid is uitstekend	◯ Volkomen juist<br>◯ Grotendeels juist<br>◯ Weet ik niet<br>◯ Grotendeels onjuist<br>◯ Volkomen onjuist

Vragenlijst PRIMA studie - EQ-5D-3L

Number	Question	Answers
	Deze vragenlijst gaat over uw standpunten t.a.v. uw gezondheid. Met behulp van deze gegevens kan worden bijgehouden hoe u zich voelt en hoe goed u in staat bent uw gebruikelijke bezigheden uit te voeren. Beantwoord elke vraag door een antwoord aan te klikken. Als u niet zeker weet hoe u een vraag moet beantwoorden, geef dan het best mogelijke antwoord.	
7.1	Hoe is het met uw Mobiliteit gesteld?	◯ Ik heb geen problemen met lopen<br>◯ Ik heb enige problemen met lopen<br>◯ Ik ben bedlegerig
7.2	Hoe is het met uw Zelfzorg gesteld?	◯ Ik heb geen problemen om mijzelf te wassen of aan te kleden<br>◯ Ik heb enige problemen om mijzelf te wassen of aan te kleden<br>◯ Ik ben niet in staat om mijzelf te wassen of aan te kleden
7.3	Hoe is het met de Dagelijkse activiteiten (werk, studie, huishouden, gezins- en vrijetijdsactiviteiten) gesteld?	◯ Ik heb geen problemen met mijn dagelijkse activiteiten<br>◯ Ik heb enige problemen met mijn dagelijkse activiteiten<br>◯ Ik ben niet in staat om mijn dagelijkse activiteiten uit te voeren

7.4	Hoe is het met de Pijn/klachten gesteld?	◯ Ik heb geen pijn of andere klachten<br>◯ Ik heb matige pijn of andere klachten<br>◯ Ik heb zeer ernstige pijn of andere klachten
7.5	Hoe is het met de Stemming gesteld?	◯ Ik ben niet angstig of somber<br>◯ Ik ben matig angstig of somber<br>◯ Ik ben erg angstig of somber
	We willen weten hoe goed of slecht uw gezondheid VANDAAG is. Deze meetschaal loopt van 0 tot 100: 100 staat voor de BESTE gezondheid die u zich kunt voorstellen - 0 staat voor de SLECHTSTE gezondheid die u zich kunt voorstellen	
7.6	Uw Gezondheid vandaag	(0.00) (100.00)

Vragenlijst PRIMA studie - AOS

Number	Question	Answers
	Instructies: De lijn naast elke vraag staat voor hoeveel PIJN u heeft in verschillende situaties. De linker kant (0) is “geen pijn” en de rechter kant (100) is “ergste pijn denkbaar”. Geef voor de onderstaande situaties op de lijn aan hoeveel PIJN u in de afgelopen week in de enkel had. Als een of meerdere van deze situaties niet van toepassing waren, dan kiest u de "niet van toepassing" optie.	
8.1	Wat was de hoogte van de ergste pijn in de afgelopen week?	(0.00) (100.00)
8.2	Heeft u pijn als u 's ochtends opstaat?	◯ Ja<br>◯ Nee<br>◯ Niet van toepassing
8.2.1	***If 'Heeft u pijn als u 's ochtends opstaat?' is equal to 'Ja' answer this question:***<br>Hoeveel pijn heeft u voordat u 's ochtends opstaat?	(0.00) (100.00)
8.3	Heeft u pijn wanneer u op blote voeten loopt?	◯ Ja<br>◯ Nee<br>◯ Niet van toepassing
8.3.1	***If 'Heeft u pijn wanneer u op blote voeten loopt?' is equal to 'Ja' answer this question:***<br>Hoeveel pijn heeft u als u op blote voeten loopt?	(0.00) (100.00)

8.4	Heeft u pijn wanneer u op blote voeten staat?	◯ Ja<br>◯ Nee<br>◯ Niet van toepassing	
8.4.1	***If 'Heeft u pijn wanneer u op blote voeten staat?' is equal to 'Ja' answer this question:***<br>Hoeveel pijn heeft u als u op blote voeten staat?	(0.00)	(100.00)
8.5	Heeft u pijn wanneer u met schoenen loopt?	◯ Ja<br>◯ Nee<br>◯ Niet van toepassing	
8.5.1	***If 'Heeft u pijn wanneer u met schoenen loopt?' is equal to 'Ja' answer this question:***<br>Hoeveel pijn heeft u wanneer u met schoenen loopt?	(0.00)	(100.00)
8.6	Heeft u pijn wanneer u met schoenen staat?	◯ Ja<br>◯ Nee<br>◯ Niet van toepassing	
8.6.1	***If 'Heeft u pijn wanneer u met schoenen staat?' is equal to 'Ja' answer this question:***<br>Hoeveel pijn heeft u wanneer u met schoenen staat?	(0.00)	(100.00)
8.7	Heeft u pijn wanneer u loopt met steunzolen of een brace?	◯ Ja<br>◯ Nee<br>◯ Niet van toepassing	
8.7.1	***If 'Heeft u pijn wanneer u loopt met steunzolen of een brace?' is equal to 'Ja' answer this question:***<br>Hoeveel pijn heeft u wanneer u loopt met steunzolen of een brace?	(0.00)	(100.00)
8.8	Heeft u pijn wanneer u staat met steunzolen of een brace?	◯ Ja<br>◯ Nee<br>◯ Niet van toepassing	
8.8.1	***If 'Heeft u pijn wanneer u staat met steunzolen of een brace?' is equal to 'Ja' answer this question:***<br>Hoeveel pijn heeft u wanneer u staat met steunzolen of een brace?	(0.00)	(100.00)
8.9	Heeft u pijn aan het einde van de dag?	◯ Ja<br>◯ Nee<br>◯ Niet van toepassing	

8.9.1	***If 'Heeft u pijn aan het einde van de dag?' is equal to 'Ja' answer this question:***<br>Hoeveel pijn heeft u aan het einde van de dag?	(0.00)	(100.00)

Instructies:De lijn naast elke vraag staat voor hoeveel MOEITE u heeft met verschillende activiteiten. De linker kant (0) is “Niet moeilijk” en de rechter kant (100) is “Te moeilijk, niet uitvoerbaar”. Geef voor de onderstaande activiteiten op de lijn aan hoeveel MOEITE u in de afgelopen week door enkelklachten had met onderstaande activiteiten. Als een situatie niet van toepassing is, kies dan de optie "niet van toepassing".

8.10	Heeft u moeite met door het huis lopen?	◯ Ja<br>◯ Nee<br>◯ Niet van toepassing	
8.10.1	***If 'Heeft u moeite met door het huis lopen?' is equal to 'Ja' answer this question:***<br>Hoeveel moeite heeft u met door het huis lopen?	(0.00)	(100.00)
8.11	Heeft u moeite met buiten lopen op oneven ondergrond?	◯ Ja<br>◯ Nee<br>◯ Niet van toepassing	
8.11.1	***If 'Heeft u moeite met buiten lopen op oneven ondergrond?' is equal to 'Ja' answer this question:***<br>Hoeveel moeite heeft u met buiten lopen op oneven ondergrond?	(0.00)	(100.00)
8.12	Heeft u moeite met een paar honderd meter lopen?	◯ Ja<br>◯ Nee<br>◯ Niet van toepassing	
8.12.1	***If 'Heeft u moeite met een paar honderd meter lopen?' is equal to 'Ja' answer this question:***<br>Hoeveel moeite heeft u met een paar honderd meter lopen?	(0.00)	(100.00)
8.13	Heeft u moeite met een trap oplopen?	◯ Ja<br>◯ Nee<br>◯ Niet van toepassing	
8.13.1	***If 'Heeft u moeite met een trap oplopen?' is equal to 'Ja' answer this question:***<br>Hoeveel moeite heeft u met een trap oplopen?	(0.00)	(100.00)
8.14	Heeft u moeite met een trap aflopen?	◯ Ja<br>◯ Nee<br>◯ Niet van toepassing	

8.14.1	***If 'Heeft u moeite met een trap aflopen?' is equal to 'Ja' answer this question:***<br>Hoeveel moeite heeft u met een trap aflopen?	(0.00)	(100.00)
8.15	Heeft u moeite met op de tenen staan?	◯ Ja<br>◯ Nee<br>◯ Niet van toepassing	
8.15.1	***If 'Heeft u moeite met op de tenen staan?' is equal to 'Ja' answer this question:***<br>Hoeveel moeite heeft u met op de tenen staan?	(0.00)	(100.00)
8.16	Heeft u moeite met opstaan uit de stoel?	◯ Ja<br>◯ Nee<br>◯ Niet van toepassing	
8.16.1	***If 'Heeft u moeite met opstaan uit de stoel?' is equal to 'Ja' answer this question:***<br>Hoeveel moeite heeft u met opstaan uit de stoel?	(0.00)	(100.00)
8.17	Heeft u moeite met het op- of afstappen van stoepranden?	◯ Ja<br>◯ Nee<br>◯ Niet van toepassing	
8.17.1	***If 'Heeft u moeite met het op- of afstappen van stoepranden?' is equal to 'Ja' answer this question:***<br>Hoeveel moeite heeft u met het op- of afstappen van stoepranden?	(0.00)	(100.00)
8.18	Heeft u moeite met snel lopen of rennen?	◯ Ja<br>◯ Nee<br>◯ Niet van toepassing	
8.18.1	***If 'Heeft u moeite met snel lopen of rennen?' is equal to 'Ja' answer this question:***<br>Hoeveel moeite heeft u met snel lopen of rennen?	(0.00)	(100.00)

Vragenlijst PRIMA studie - FAOS

Number	Question	Answers

Deze lijst vraagt naar uw mening over uw voet/enkel. Uw antwoorden geven ons een beeld van uw voet/enkel klachten en hoe u in staat bent om alledaagse activiteiten uit te voeren in uw huidige situatie. Beantwoorden van een vraag doet u door het aanklikken van een vakje met het volgens u meest juiste antwoord (één vakje per vraag). Als u niet zeker weet hoe u een vraag moet beantwoorden, geeft u dan het antwoord dat volgens u het meest op uw situatie van toepassing is. Deze vraag heeft betrekking op het voorkomen van voet/enkel klachten in de afgelopen week.

9.1	Is uw voet/enkel gezwollen?	○ Nooit ○ Zelden ○ Soms ○ Vaak ○ Altijd
9.2	Voelt u gekraak of hoort u klikken of een ander vreemd geluid wanneer u de voet/enkel beweegt?	○ Nooit ○ Zelden ○ Soms ○ Vaak ○ Altijd
9.3	Hapert uw enkel of blokkeert uw enkel ('op slot' gaan zitten) wanneer u deze beweegt?	○ Nooit ○ Zelden ○ Soms ○ Vaak ○ Altijd
9.4	Kunt u de voet/enkel volledig strekken?	○ Altijd ○ Vaak ○ Soms ○ Zelden ○ Nooit
9.5	Kunt u de voet volledig naar u toe buigen?	○ Altijd ○ Vaak ○ Soms ○ Zelden ○ Nooit
9.6	In welke mate heeft u een stijf gevoel in de voet/enkel 's ochtends bij het wakker worden?	○ Niet ○ Mild ○ Matig ○ Ernstig ○ Zeer ernstig
9.7	In welke mate heeft u een stijf gevoel in de voet/enkel na zitten, liggen of rusten later op de dag?	○ Niet ○ Mild ○ Matig ○ Ernstig ○ Zeer ernstig
9.8	Hoe vaak heeft u pijn in uw voet/enkel?	○ Nooit ○ Maandelijks ○ Wekelijks ○ Dagelijks ○ Altijd
9.9	Hoeveel voet/enkel pijn heeft u gehad in de afgelopen week bij draaien als uw voet/enkel op de grond staat?	○ Geen<br>○ Mild<br>○ Matig<br>○ Ernstig<br>○ Zeer ernstig
9.10	Hoeveel voet/enkel pijn heeft u gehad in de afgelopen week bij het volledig uitstrekken van de voet/enkel?	○ Geen<br>○ Mild<br>○ Matig<br>○ Ernstig<br>○ Zeer ernstig

9.11	Hoeveel voet/enkel pijn heeft u gehad in de afgelopen week bij het volledig naar u toe buigen/optrekken van de voet/enkel	○ Geen<br>○ Mild<br>○ Matig<br>○ Ernstig<br>○ Zeer ernstig
9.12	Hoeveel voet/enkel pijn heeft u gehad in de afgelopen week bij het lopen op een vlakke ondergrond	○ Geen<br>○ Mild<br>○ Matig<br>○ Ernstig<br>○ Zeer ernstig
9.13	Hoeveel voet/enkel pijn heeft u gehad in de afgelopen week bij het trap op- en trap af lopen?	○ Geen<br>○ Mild<br>○ Matig<br>○ Ernstig<br>○ Zeer ernstig
9.14	Hoeveel voet/enkel pijn heeft u gehad in de afgelopen week 's nachts in bed?	○ Geen<br>○ Mild<br>○ Matig<br>○ Ernstig<br>○ Zeer ernstig
9.15	Hoeveel voet/enkel pijn heeft u gehad in de afgelopen week bij het zitten of liggen?	○ Geen<br>○ Mild<br>○ Matig<br>○ Ernstig<br>○ Zeer ernstig
9.16	Hoeveel voet/enkel pijn heeft u gehad in de afgelopen week bij het rechtop staan?	○ Geen<br>○ Mild<br>○ Matig<br>○ Ernstig<br>○ Zeer ernstig
9.17	In welke mate werd u gehinderd bij het trap aflopen?	○ Niet ○ Mild ○ Matig ○ Ernstig ○ Zeer ernstig
9.18	In welke mate werd u gehinderd bij het trap op lopen?	○ Niet ○ Mild ○ Matig ○ Ernstig ○ Zeer ernstig

9.19	In welke mate werd u gehinderd als u vanuit een zittende positie ging staan?	◯ Niet ◯ Mild ◯ Matig ◯ Ernstig ◯ Zeer ernstig
9.20	In welke mate werd u gehinderd bij het staan?	◯ Niet ◯ Mild ◯ Matig ◯ Ernstig ◯ Zeer ernstig
9.21	In welke mate werd u gehinderd bij het naar de grond buigen/iets oprapen?	◯ Niet ◯ Mild ◯ Matig ◯ Ernstig ◯ Zeer ernstig
9.22	In welke mate werd u gehinderd bij het lopen op een vlakke ondergrond?	◯ Niet ◯ Mild ◯ Matig ◯ Ernstig ◯ Zeer ernstig
9.23	In welke mate werd u gehinderd bij het in- en uit de auto stappen?	◯ Niet ◯ Mild ◯ Matig ◯ Ernstig ◯ Zeer ernstig
9.24	In welke mate werd u gehinderd bij het boodschappen doen?	◯ Niet ◯ Mild ◯ Matig ◯ Ernstig ◯ Zeer ernstig
9.25	In welke mate werd u gehinderd bij sokken/panty's aantrekken?	◯ Niet ◯ Mild ◯ Matig ◯ Ernstig ◯ Zeer ernstig
9.26	In welke mate werd u gehinderd bij het opstaan uit bed?	◯ Niet ◯ Mild ◯ Matig ◯ Ernstig ◯ Zeer ernstig
9.27	In welke mate werd u gehinderd bij het sokken uittrekken?	◯ Niet ◯ Mild ◯ Matig ◯ Ernstig ◯ Zeer ernstig
9.28	In welke mate werd u gehinderd bij het in bed liggen (omdraaien, lange tijd uw voet/enkel in dezelfde positie houden)?	◯ Niet ◯ Mild ◯ Matig ◯ Ernstig ◯ Zeer ernstig
9.29	In welke mate werd u gehinderd bij het in/uit bad stappen?	◯ Niet ◯ Mild ◯ Matig ◯ Ernstig ◯ Zeer ernstig
9.30	In welke mate werd u gehinderd bij zitten?	◯ Niet ◯ Mild ◯ Matig ◯ Ernstig ◯ Zeer ernstig
9.31	In welke mate werd u gehinderd bij het toilet op en af gaan?	◯ Niet ◯ Mild ◯ Matig ◯ Ernstig ◯ Zeer ernstig

9.32	In welke mate werd u gehinderd bij zwaar huishoudelijk werk (bijvoorbeeld zware dozen sjouwen, vloer schrobben)?	◯ Niet ◯ Mild ◯ Matig ◯ Ernstig ◯ Zeer ernstig
9.33	In welke mate werd u gehinderd bij licht huishoudeijk werk (bijvoorbeeld koken, afstoffen)?	◯ Niet ◯ Mild ◯ Matig ◯ Ernstig ◯ Zeer ernstig
9.34	In welke mate werd u gehinderd bij hurken?	◯ Niet ◯ Mild ◯ Matig ◯ Ernstig ◯ Zeer ernstig
9.35	In welke mate werd u gehinderd bij hardlopen?	◯ Niet ◯ Mild ◯ Matig ◯ Ernstig ◯ Zeer ernstig
9.36	In welke mate werd u gehinderd bij springen?	◯ Niet ◯ Mild ◯ Matig ◯ Ernstig ◯ Zeer ernstig
9.37	In welke mate werd u gehinderd bij ronddraaien op uw aangedane voet/enkel?	◯ Niet ◯ Mild ◯ Matig ◯ Ernstig ◯ Zeer ernstig
9.38	In welke mate werd u gehinderd bij knielen?	◯ Niet ◯ Mild ◯ Matig ◯ Ernstig ◯ Zeer ernstig
9.39	Hoe vaak bent u zich bewust van uw voet/enkel probleem?	◯ Nooit ◯ Maandelijks ◯ Wekelijks ◯ Dagelijks ◯ Altijd
9.40	Heeft u uw leven veranderd om activiteiten te vermijden die schadelijk kunnen zijn voor uw voet/enkel?	◯ Niet<br>◯ Enigszins<br>◯ Matig<br>◯ Behoorlijk<br>◯ Volledig
9.41	In hoeverre kunt u op uw voet/enkel vertrouwen?	◯ Volledig<br>◯ Behoorlijk<br>◯ Matig<br>◯ Enigzins<br>◯ Niet
9.42	In het algemeen, in welke mate ondervindt u hinder van uw voet/enkel	◯ Geen<br>◯ Mild<br>◯ Matig<br>◯ Ernstig<br>◯ Zeer ernstig

Vragenlijst PRIMA studie - PRODISQ - Gezondheid en werk

Number	Question	Answers
10.1	Wat is uw leeftijd?	[ ] jaar
10.2	Wat is uw geslacht?	○ Man<br>○ Vrouw
10.3	Wat is de hoogste opleiding die u heeft afgemaakt (Zoek uw hoogste opleiding en kruis het hokje daarvoor aan)?	☐ Ik heb geen school of opleiding afgemaakt<br>☐ Lagere school of basisschool<br>☐ Huishoudschool, vbo, lbo, lts, leao of lhno<br>☐ Mavo, mulo, ivo of vmbo<br>☐ Mbo, mts, meao, mhno, inas of intas<br>☐ Havo, vwo, hbs, mms, atheneum of gymnasium<br>☐ Hbo, hts, heao of hhno<br>☐ Universiteit<br>☐ Ik heb een andere opleiding afgemaakt, namelijk
10.3.1	***If 'Wat is de hoogste opleiding die u heeft afgemaakt (Zoek uw hoogste opleiding en kruis het hokje daarvoor aan)?' is equal to 'Ik heb een andere opleiding afgemaakt, namelijk' answer this question:***<br>Wat is de hoogste opleiding die u heeft afgemaakt?	[ ]
10.4	Wat doet u in het dagelijks leven?	☐ Ik zit op school, ik studeer<br>☐ Ik werk in loondienst<br>☐ Ik ben zelfstandig ondernemer<br>☐ Ik ben huisvrouw, huisman<br>☐ Ik ben werkloos<br>☐ Ik ben arbeidsongeschikt<br>☐ Ik ben met pensioen of prepensioen<br>☐ Ik doe iets anders, namelijk
10.4.1	***If 'Wat doet u in het dagelijks leven?' is equal to 'Ik ben arbeidsongeschikt' answer this question:***<br>Wat doet u in het dagelijks leven?	[ ]

10.4.2	***If 'Wat doet u in het dagelijks leven?' is equal to 'Ik doe iets anders, namelijk' answer this question:***<br>Wat doet u in het dagelijks leven?	
10.5	Hebt u betaald werk?	◯ Nee<br>◯ Ja
10.5.1	***If 'Hebt u betaald werk?' is equal to 'Ja' answer this question:***<br>Wat is uw beroep?	
10.5.2	***If 'Hebt u betaald werk?' is equal to 'Ja' answer this question:***<br>Hoeveel uur per week werkt u (Tel alleen de uren waarvoor u betaald wordt)?	uren
10.5.3	***If 'Hebt u betaald werk?' is equal to 'Ja' answer this question:***<br>Hoeveel dagen in de week werkt u?	Dagen
10.5.4	***If 'Hebt u betaald werk?' is equal to 'Ja' answer this question:***<br>Bent u in de afgelopen 4 weken afwezig geweest van uw werk omdat u ziek was?	◯ Nee<br>◯ Ja
10.5.4.1	***If 'Bent u in de afgelopen 4 weken afwezig geweest van uw werk omdat u ziek was?' is equal to 'Ja' answer this question:***<br>Bent u in de afgelopen 4 weken afwezig geweest van uw werk omdat u ziek was (Tel alleen de werkdagen in de afgelopen 4 weken)?	dagen afwezig geweest
10.5.4.2	***If 'Bent u in de afgelopen 4 weken afwezig geweest van uw werk omdat u ziek was?' is equal to 'Ja' answer this question:***<br>Was u langer dan de gehele periode van 4 weken afwezig van uw werk doordat u ziek was?	◯ Nee<br>◯ Ja
10.5.4.2.1	***If 'Was u langer dan de gehele periode van 4 weken afwezig van uw werk doordat u ziek was?' is equal to 'Ja' answer this question:***<br>Wanneer heeft u zich ziek gemeld?	*(dd-mm-yyyy)*
10.5.5	***If 'Hebt u betaald werk?' is equal to 'Ja' answer this question:***<br>Waren er in de afgelopen 4 weken, dagen waarop u wel gewerkt heeft, maar tijdens uw werk last had van lichamelijke of psychische problemen?	◯ Ja<br>◯ Nee

10.5.5.1	***If 'Waren er in de afgelopen 4 weken, dagen waarop u wel gewerkt heeft, maar tijdens uw werk last had van lichamelijke of psychische problemen?' is equal to 'Ja' answer this question:***<br>Op hoeveel werkdagen had u tijdens uw werk last van uw lichamelijke of psychische problemen?	[ ] werkdagen
10.5.5.2	***If 'Waren er in de afgelopen 4 weken, dagen waarop u wel gewerkt heeft, maar tijdens uw werk last had van lichamelijke of psychische problemen?' is equal to 'Ja' answer this question:***<br>Op de dagen dat u last had, kon u misschien niet zoveel werk doen als normaal. Hoeveel werk kon u op deze dagen gemiddeld doen?	Ik kon op deze dagen niks doen (0.00) … Ik kon net zoveel doen als normaal (10.00)
10.6	Waren er dagen waarop u minder onbetaald werk kon doen door uw lichamelijke of psychische problemen?	◯ Nee<br>◯ Ja
10.6.1	***If 'Waren er dagen waarop u minder onbetaald werk kon doen door uw lichamelijke of psychische problemen?' is equal to 'Ja' answer this question:***<br>Op hoeveel dagen was dit zo?	[ ] Dagen
10.6.2	***If 'Waren er dagen waarop u minder onbetaald werk kon doen door uw lichamelijke of psychische problemen?' is equal to 'Ja' answer this question:***<br>Stel dat iemand, bijvoorbeeld uw partner, familielid of een bekende, u op deze dagen had geholpen. En al het onbetaalde werk wat u niet kon doen, voor u had gedaan. Hoeveel uur was die persoon hier op deze dagen dan gemiddeld mee bezig geweest?	[ ] uur op deze dagen

Vragenlijst PRIMA studie - PRODISQ - Zorggebruik

Number	Question	Answers
	Wij willen graag weten met welke dokters u in de afgelopen 3 maanden een afspraak had. Het gaat om afspraken voor uzelf. Ook andere zorgverleners tellen mee. Bijvoorbeeld de fysiotherapeut.	
11.1	Bent u in de afgelopen 3 maanden naar uw huisarts geweest?	◯ Nee<br>◯ Ja
11.1.1	***If 'Bent u in de afgelopen 3 maanden naar uw huisarts geweest?' is equal to 'Ja' answer this question:***<br>Hoeveel afspraken had u?	[ ] afspraken

11.2	Bent u in de afgelopen 3 maanden in contact geweest met een maatschappelijk werker?	◯ Nee<br>◯ Ja
11.2.1	***If 'Bent u in de afgelopen 3 maanden in contact geweest met een maatschappelijk werker?' is equal to 'Ja' answer this question:***<br>Hoeveel afspraken had u?	[ ] afspraken
11.3	Bent u in de afgelopen 3 maanden naar een fysiotherapeut geweest? Of een caesartherapeut, therapeut mensendieck of een manueel therapeut?	◯ Nee<br>◯ Ja
11.3.1	***If 'Bent u in de afgelopen 3 maanden naar een fysiotherapeut geweest? Of een caesartherapeut, therapeut mensendieck of een manueel therapeut?' is equal to 'Ja' answer this question:***<br>Hoeveel afspraken had u?	[ ] afspraken
11.4	Bent u in de afgelopen 3 maanden naar een ergotherapeut geweest?	◯ Nee<br>◯ Ja
11.4.1	***If 'Bent u in de afgelopen 3 maanden naar een ergotherapeut geweest?' is equal to 'Ja' answer this question:***<br>Hoeveel afspraken had u?	[ ] afspraken
11.5	Bent u in de afgelopen 3 maanden naar een logopedist geweest?	◯ Nee<br>◯ Ja
11.5.1	***If 'Bent u in de afgelopen 3 maanden naar een logopedist geweest?' is equal to 'Ja' answer this question:***<br>Hoeveel afspraken had u?	[ ] afspraken
11.6	Bent u in de afgelopen 3 maanden naar een diëtist geweest?	◯ Nee<br>◯ Ja
11.6.1	***If 'Bent u in de afgelopen 3 maanden naar een diëtist geweest?' is equal to 'Ja' answer this question:***<br>Hoeveel afspraken had u?	[ ] afspraken
11.7	Bent u in de afgelopen 3 maanden naar een homeopaat geweest? Of een acupuncturist?	◯ Nee<br>◯ Ja
11.7.1	***If 'Bent u in de afgelopen 3 maanden naar een homeopaat geweest? Of een acupuncturist?' is equal to 'Ja' answer this question:***<br>Hoeveel afspraken had u?	[ ] afspraken

11.8	Bent u in de afgelopen 3 maanden naar een psycholoog geweest? Of een psychotherapeut of psychiater?	◯ Nee<br>◯ Ja
11.8.1	***If 'Bent u in de afgelopen 3 maanden naar een psycholoog geweest? Of een psychotherapeut of psychiater?' is equal to 'Ja' answer this question:***<br>Hoeveel afspraken had u?	[ ] afspraken
11.9	Heeft u in de afgelopen 3 maanden afspraken gehad met de bedrijfsarts?	◯ Nee<br>◯ Ja
11.9.1	***If 'Heeft u in de afgelopen 3 maanden afspraken gehad met de bedrijfsarts?' is equal to 'Ja' answer this question:***<br>Hoeveel afspraken had u?	[ ] afspraken
11.10	Heeft u in de afgelopen 3 maanden hulp van de thuiszorg gehad?	◯ Ja<br>◯ Nee
11.10.1	***If 'Heeft u in de afgelopen 3 maanden hulp van de thuiszorg gehad?' is equal to 'Ja' answer this question:***<br>Wat voor hulp van de thuiszorg heeft u gehad in de afgelopen 3 maanden?	☐ Huishoudelijke hulp; voorbeeld: stofzuigen, bed opmaken, boodschappen doen<br>☐ Verzorging van uzelf; voorbeeld: hulp bij douchen of aankleden<br>☐ Verpleging; voorbeeld: verband omdoen, medicijnen geven, bloeddruk meten
11.10.1.1	***If 'Wat voor hulp van de thuiszorg heeft u gehad in de afgelopen 3 maanden?' is equal to 'Huishoudelijke hulp; voorbeeld: stofzuigen, bed opmaken, boodschappen doen' answer this question:***<br>Hoeveel weken heeft u deze thuiszorg gehad? Huishoudelijke hulp:	[ ] weken in de afgelopen 3 maanden
11.10.1.2	***If 'Wat voor hulp van de thuiszorg heeft u gehad in de afgelopen 3 maanden?' is equal to 'Verzorging van uzelf; voorbeeld: hulp bij douchen of aankleden' answer this question:***<br>Hoeveel weken heeft u deze thuiszorg gehad? Verzorging van uzelf:	[ ] weken in de afgelopen 3 maanden
11.10.1.3	***If 'Wat voor hulp van de thuiszorg heeft u gehad in de afgelopen 3 maanden?' is equal to 'Verpleging; voorbeeld: verband omdoen, medicijnen geven, bloeddruk meten' answer this question:***<br>Hoeveel weken heeft u deze thuiszorg gehad? Verpleging:	[ ] weken in de afgelopen 3 maanden

11.10.1.4	***If 'Wat voor hulp van de thuiszorg heeft u gehad in de afgelopen 3 maanden?' is equal to 'Huishoudelijke hulp; voorbeeld: stofzuigen, bed opmaken, boodschappen doen' answer this question:*** Hoeveel uur thuiszorg kreeg u in deze weken gemiddeld? Huishoudelijke hulp: gemiddeld	[ ] uur in de week
11.10.1.5	***If 'Wat voor hulp van de thuiszorg heeft u gehad in de afgelopen 3 maanden?' is equal to 'Verzorging van uzelf; voorbeeld: hulp bij douchen of aankleden' answer this question:*** Hoeveel uur thuiszorg kreeg u in deze weken gemiddeld? Verzorging van uzelf: gemiddeld	[ ] uur in de week
11.10.1.6	***If 'Wat voor hulp van de thuiszorg heeft u gehad in de afgelopen 3 maanden?' is equal to 'Verpleging; voorbeeld: verband omdoen, medicijnen geven, bloeddruk meten' answer this question:*** Hoeveel uur thuiszorg kreeg u in deze weken gemiddeld? Verpleging: gemiddeld	[ ] uur in de week
11.11	Heeft u in de afgelopen 3 maanden medicijnen gebruikt?	○ Ja<br>○ Nee
11.11.1	***If 'Heeft u in de afgelopen 3 maanden medicijnen gebruikt?' is equal to 'Ja' answer this question:*** Welke medicijnen heeft u in de afgelopen 3 maanden gebruikt?	[ ]
11.12	Bent u in de afgelopen 3 maanden op de spoedeisende eerste hulp van een ziekenhuis geweest? (Een andere naam voor spoedeisende eerste hulp is EHBO)	○ Nee<br>○ Ja
11.12.1	***If 'Bent u in de afgelopen 3 maanden op de spoedeisende eerste hulp van een ziekenhuis geweest? (Een andere naam voor spoedeisende eerste hulp is EHBO)' is equal to 'Ja' answer this question:*** Hoevaak bent u geweest?	[ ] keer
11.13	Bent u in de afgelopen 3 maanden met een ambulance naar het ziekenhuis gebracht? (Een andere naam voor ambulance is ziekenauto)	○ Nee<br>○ Ja
11.13.1	***If 'Bent u in de afgelopen 3 maanden met een ambulance naar het ziekenhuis gebracht? (Een andere naam voor ambulance is ziekenauto)' is equal to 'Ja' answer this question:*** Hoe vaak bent u naar het ziekenhuis gebracht?	[ ] keer

11.14	Had u in de afgelopen 3 maanden een afspraak bij de polikliniek van het ziekenhuis? (Het gaat om afspraken voor uzelf met een dokter. Bijvoorbeeld met de cardioloog, reumatoloog of neuroloog)	◯ Ja<br>◯ Nee
11.14.1	***If 'Had u in de afgelopen 3 maanden een afspraak bij de polikliniek van het ziekenhuis? (Het gaat om afspraken voor uzelf met een dokter. Bijvoorbeeld met de cardioloog, reumatoloog of neuroloog)' is equal to 'Ja' answer this question:***<br>Bij welke soorten dokters bent u in de afgelopen 3 maanden in het ziekenhuis geweest? En hoe vaak?	
11.15	Bent u in de afgelopen 3 maanden overdag in het ziekenhuis geweest voor een behandeling? (U bleef dus niet slapen. U kwam bijvoorbeeld voor een bloedtransfusie, nierdialyse of chemokuur)	◯ Ja<br>◯ Nee
11.15.1	***If 'Bent u in de afgelopen 3 maanden overdag in het ziekenhuis geweest voor een behandeling? (U bleef dus niet slapen. U kwam bijvoorbeeld voor een bloedtransfusie, nierdialyse of chemokuur)' is equal to 'Ja' answer this question:***<br>Voor welke soort behandeling was dit?	
11.15.2	***If 'Bent u in de afgelopen 3 maanden overdag in het ziekenhuis geweest voor een behandeling? (U bleef dus niet slapen. U kwam bijvoorbeeld voor een bloedtransfusie, nierdialyse of chemokuur)' is equal to 'Ja' answer this question:***<br>Hoeveel keer moest u in de afgelopen 3 maanden voor deze behandelingen naar het ziekenhuis?	
11.16	Bent u in de afgelopen 3 maanden ergens anders geweest voor een behandeling overdag? (U bleef dus niet slapen. U ging bijvoorbeeld naar de dagopvang van een woon-/zorgcentrum of een psychiatrische instelling. Of naar de dagbehandeling van een revalidatiecentrum)	◯ Ja<br>◯ Nee
11.16.1	***If 'Bent u in de afgelopen 3 maanden ergens anders geweest voor een behandeling overdag? (U bleef dus niet slapen. U ging bijvoorbeeld naar de dagopvang van een woon-/zorgcentrum of een psychiatrische instelling. Of naar de dagbehandeling van een revalidatiecentrum)' is equal to 'Ja' answer this question:***<br>Wat voor instelling was dit?	☐ Woon-/zorgcentrum<br>☐ Revalidatiecentrum<br>☐ Psychiatrische instelling<br>☐ Een andere instelling, namelijk
11.16.1.1	***If 'Wat voor instelling was dit?' is equal to 'Een andere instelling, namelijk' answer this question:***<br>Wat voor instelling was dit?	

11.16.1.2	***If 'Wat voor instelling was dit?' is equal to 'Woon-/zorgcentrum' answer this question:***<br>Hoe vaak moest u hier in de afgelopen 3 maanden naartoe? Naar het woon-/zorgcentrum:	[ ] keer in de afgelopen 3 maanden
11.16.1.3	***If 'Wat voor instelling was dit?' is equal to 'Revalidatiecentrum' answer this question:***<br>Hoe vaak moest u hier in de afgelopen 3 maanden naartoe? Naar het revalidatiecentrum:	[ ] keer in de afgelopen 3 maanden
11.16.1.4	***If 'Wat voor instelling was dit?' is equal to 'Psychiatrische instelling' answer this question:***<br>Hoe vaak moest u hier in de afgelopen 3 maanden naartoe? Naar de psychiatrische instelling:	[ ] keer in de afgelopen 3 maanden
11.16.1.5	***If 'Wat voor instelling was dit?' is equal to 'Een andere instelling, namelijk' answer this question:***<br>Hoe vaak moest u hier in de afgelopen 3 maanden naartoe? Naar de andere instelling:	[ ] keer in de afgelopen 3 maanden
11.17	Heeft u in de afgelopen 3 maanden weleens in het ziekenhuis gelegen? (U moest dus blijven slapen. Bijvoorbeeld omdat u geopereerd was en niet meteen naar huis kon)	○ Ja<br>○ Nee
11.17.1	***If 'Heeft u in de afgelopen 3 maanden weleens in het ziekenhuis gelegen? (U moest dus blijven slapen. Bijvoorbeeld omdat u geopereerd was en niet meteen naar huis kon)' is equal to 'Ja' answer this question:***<br>Hoe vaak heeft u in de afgelopen 3 maanden in het ziekenhuis gelegen?	[ ] keer in de afgelopen 3 maanden
11.17.2	***If 'Heeft u in de afgelopen 3 maanden weleens in het ziekenhuis gelegen? (U moest dus blijven slapen. Bijvoorbeeld omdat u geopereerd was en niet meteen naar huis kon)' is equal to 'Ja' answer this question:***<br>Hoe lang heeft u in het ziekenhuis gelegen?	[ ] dagen in de afgelopen 3 maanden
11.18	Moest u in de afgelopen 3 maanden ergens anders blijven slapen voor uw gezondheid? (Bijvoorbeeld in een woon-/zorgcentrum, psychiatrische instelling of revalidatiecentrum)	○ Ja<br>○ Nee
11.18.1	***If 'Moest u in de afgelopen 3 maanden ergens anders blijven slapen voor uw gezondheid? (Bijvoorbeeld in een woon-/zorgcentrum, psychiatrische instelling of revalidatiecentrum)' is equal to 'Ja' answer this question:***<br>Wat voor instelling was dit?	☐ Woon-/zorgcentrum<br>☐ Revalidatiecentrum<br>☐ Psychiatrische instelling<br>☐ Een andere instelling, namelijk

11.18.1.1	***If 'Wat voor instelling was dit?' is equal to 'Een andere instelling, namelijk' answer this question:*** Een andere instelling, namelijk	
11.18.1.2	***If 'Wat voor instelling was dit?' is equal to 'Woon-/zorgcentrum' answer this question:*** Hoe lang bent u in deze instelling geweest? In het woon-/zorgcentrum:	dagen in de afgelopen 3 maanden
11.18.1.3	***If 'Wat voor instelling was dit?' is equal to 'Revalidatiecentrum' answer this question:*** Hoe lang bent u in deze instelling geweest? In het revalidatiecentrum:	dagen in de afgelopen 3 maanden
11.18.1.4	***If 'Wat voor instelling was dit?' is equal to 'Psychiatrische instelling' answer this question:*** Hoe lang bent u in deze instelling geweest? In de psychiatrische instelling:	dagen in de afgelopen 3 maanden
11.18.1.5	***If 'Wat voor instelling was dit?' is equal to 'Een andere instelling, namelijk' answer this question:*** Hoe lang bent u in deze instelling geweest? In de andere instelling:	dagen in de afgelopen 3 maanden
11.19	Heeft u in de afgelopen 3 maanden hulp gekregen van een familielid of een bekende vanwege uw lichamelijke of psychische problemen?	◯ Ja<br>◯ Nee
11.19.1	***If 'Heeft u in de afgelopen 3 maanden hulp gekregen van een familielid of een bekende vanwege uw lichamelijke of psychische problemen?' is equal to 'Ja' answer this question:*** Wat voor hulp van familieleden of bekenden heeft u gehad in de afgelopen 3 maanden?	☐ Huishoudelijke hulp - voorbeeld: stofzuigen, bed opmaken, boodschappen doen, klaarmaken van eten en drinken, verzorgen van kinderen<br>☐ Verzorging van uzelf - voorbeeld: hulp bij douchen of aankleden, hulp bij het eten en drinken of het geven van medicijnen<br>☐ Praktische hulp - voorbeeld: ondersteuning bij wandelen, het maken van uitstapjes of bezoekjes aan bekenden, bezoeken aan de huisarts of het ziekenhuis, het regelen van hulp of het regelen van financiële zaken
11.19.1.1	***If 'Wat voor hulp van familieleden of bekenden heeft u gehad in de afgelopen 3 maanden?' is equal to 'Huishoudelijke hulp - voorbeeld: stofzuigen, bed opmaken, boodschappen doen, klaarmaken van eten en drinken, verzorgen van kinderen' answer this question:*** Hoeveel weken heeft u deze hulp gehad? Huishoudelijke hulp:	weken in de afgelopen 3 maanden

11.19.1.2	***If 'Wat voor hulp van familieleden of bekenden heeft u gehad in de afgelopen 3 maanden?' is equal to 'Verzorging van uzelf - voorbeeld: hulp bij douchen of aankleden, hulp bij het eten en drinken of het geven van medicijnen' answer this question:*** Hoeveel weken heeft u deze hulp gehad? Verzorging van uzelf:	[ ] weken in de afgelopen 3 maanden
11.19.1.3	***If 'Wat voor hulp van familieleden of bekenden heeft u gehad in de afgelopen 3 maanden?' is equal to 'Praktische hulp - voorbeeld: ondersteuning bij wandelen, het maken van uitstapjes of bezoekjes aan bekenden, bezoeken aan de huisarts of het ziekenhuis, het regelen van hulp of het regelen van financiële zaken' answer this question:*** Hoeveel uur hulp kreeg u in deze weken gemiddeld? Praktische hulp:	[ ] weken in de afgelopen 3 maanden
11.19.1.4	***If 'Wat voor hulp van familieleden of bekenden heeft u gehad in de afgelopen 3 maanden?' is equal to 'Huishoudelijke hulp - voorbeeld: stofzuigen, bed opmaken, boodschappen doen, klaarmaken van eten en drinken, verzorgen van kinderen' answer this question:*** Hoeveel uur hulp kreeg u in deze weken gemiddeld? Huishoudelijke hulp: gemiddeld	[ ] uur in de week
11.19.1.5	***If 'Wat voor hulp van familieleden of bekenden heeft u gehad in de afgelopen 3 maanden?' is equal to 'Verzorging van uzelf - voorbeeld: hulp bij douchen of aankleden, hulp bij het eten en drinken of het geven van medicijnen' answer this question:*** Hoeveel uur hulp kreeg u in deze weken gemiddeld? Verzorging van uzelf: gemiddeld	[ ] uur in de week
11.19.1.6	***If 'Wat voor hulp van familieleden of bekenden heeft u gehad in de afgelopen 3 maanden?' is equal to 'Praktische hulp - voorbeeld: ondersteuning bij wandelen, het maken van uitstapjes of bezoekjes aan bekenden, bezoeken aan de huisarts of het ziekenhuis, het regelen van hulp of het regelen van financiële zaken' answer this question:*** Hoeveel uur hulp kreeg u in deze weken gemiddeld? Praktische hulp: gemiddeld	[ ] uur in de week

Vragenlijst PRIMA studie - Dank

Number	Question	Answers

12.1	Hartelijk dank voor de tijd die u heeft genomen om de vragenlijsten in te vullen. Indien u nog opmerkingen heeft kunt u deze hierin plaatsen.	

Survey 'Vragenlijst PRIMA studie 12 weken'

Vragenlijst PRIMA studie 12 weken - AOFAS - Pijn

Number	Question	Answers
1.1	Hoeveel pijn ervaart u aan uw voet?	☐ Geen<br>☐ Milde pijnklachten of af en toe pijn<br>☐ Matige pijnklachten, dagelijks<br>☐ Vrijwel continu erge pijn

Vragenlijst PRIMA studie 12 weken - AOFAS resterende gedeelte

Number	Question	Answers
2.1	Hoeveel beperkingen in het dagelijks leven ervaart u?	○ Geen beperkingen, geen hulpmiddelen nodig<br>○ Geen beperkingen in algemeen dagelijkse bezigheden, wel beperkingen in recreatieve activiteiten (sport, hobbies, etc) geen ondersteuning<br>○ Beperkingen bij dagelijkse- en recreatieve activiteiten, stok<br>○ Ernstige beperkingen bij dagelijkse- en recreatieactiviteiten, walker, krukken, rolstoel, brace
2.2	Wat is de afstand die u in één keer kunt lopen?	○ Meer dan 600 meter<br>○ 400 - 600 meter<br>○ 100 - 300 meter<br>○ Minder dan 100 meter

2.3	Heeft u moeite met lopen op een bepaalde ondergrond?	◯ Geen problemen bij lopen<br>◯ Enig problemen op ongelijke ondergrond, trap, helling, ladders<br>◯ Ernstige problemen bij ongelijke ondergrond, trap, helling, ladders
2.4	Hoe zou u uw looppatroon beoordelen?	◯ Normaal of minimaal afwijkend<br>◯ Duidelijk<br>◯ Ernstig
2.5	Hoe zou u de beweeglijkheid van uw voet beoordelen ten opzicht van de andere zijde of ten opzicte van vóór het ongeval?	◯ Hetzelfde of licht beperkt t.o.v. de niet aangedane zijde of zoals voor het ongeval<br>◯ Ongeveer de helft beperkt<br>◯ Sterk verminderd, bijna geen bewegingen mogelijk
2.6	Hoe zou u de beweeglijkheid van uw enkel beoordelen ten opzichte van de andere zijde of ten opzichte van voor het ongeval?	◯ Hetzelfde of licht beperkt t.o.v. de niet aangedane zijde of zoals voor het ongeval<br>◯ Ongeveer de helft beperkt<br>◯ Sterk verminderd, bijna geen bewegingen mogelijk
2.7	Hoe zou u de stabiliteit van uw voet en enkel beoordelen	◯ Stabiel, ik verzwik mijn enkel hooguit incidenteel<br>◯ Niet stabiel, ik verzwik mijn enkel vaak
2.8	Hoe zou u de stand van uw voet beoordelen ten opzichte van de andere zijde of ten opzichte van voor het ongeval?	◯ Goed, ik kan mijn voet goed plat op de grond zetten, waarbij de stand niet afwijkend is<br>◯ Ik sta vrijwel alleen op de buitenzijde van mijn voet<br>◯ Ik kan mijn voet niet plat op de grond zetten en loop vrijwel alleen op mijn voorvoet

Vragenlijst PRIMA studie 12 weken - VAS Pain score

Number	Question	Answers	
3.1	VAS pijnscore (Geef uw minimaal pijnscore aan tijdens dagelijkse activiteiten op een schaal van 0 tot 100, waarbij 0 geen pijn is en 100 de ergste pijn denkbaar is)	(0.00)	100 (100.00)

Vragenlijst PRIMA studie 12 weken - AAS

Number	Question	Answers

4.1	Wat is het meest bij u van toepassing ten aanzien van uw acitviteit?	☐ American Football<br>☐ Basketbal<br>☐ Gymnastiek/turnen<br>☐ Handbal<br>☐ Rugby<br>☐ Voetbal<br>☐ Hockey<br>☐ Korfbal<br>☐ Gevechtssporten: judo, karate, kung fu, taekwando, jiu jitsu, etc.<br>☐ Oriëntatielopen<br>☐ Rhytmische gymnastiek<br>☐ Volleybal<br>☐ Boxen<br>☐ Freestyle snowboarden<br>☐ Ijshockey<br>☐ Tennis<br>☐ Worstelen<br>☐ Fitness, aerobics<br>☐ Badminton<br>☐ Baseball<br>☐ Cross-country hardlopen<br>☐ Moderne pentathlon<br>☐ Squash<br>☐ Surfen, windsurfen<br>☐ Tafel tennis<br>☐ Atletiek: spring-, werponderdelen<br>☐ Waterskieën<br>☐ Dans<br>☐ Schermen<br>☐ Zaalhockey<br>☐ Bergbeklimmen<br>☐ Langlauf<br>☐ Parachute springen<br>☐ Softball<br>☐ Speciale beroepen en werkactiviteiten; speciale beroepen: ballet, professioneel soldaat, speciale reddingswerker, stuntman, etc.<br>☐ Duiken<br>☐ Scubaduiken<br>☐ Skaten, in-line skaten<br>☐ Atletiek: looponderdelen<br>☐ Triatlon<br>☐ Gewichtsheffen, body-building

☐ Alle competitieve sporten hieronder met 'seasonal' conditioning
☐ Zwaar fysiek werk
☐ Alpine skiën en snowboarden
☐ Bowlen/curlen
☐ Golf
☐ Mountainbike/BMX
☐ Powerliften
☐ Zeilen
☐ Fysiek werk
☐ Wielrennen
☐ Paardrijden
☐ Motorsporten/technische sporten
☐ Roeien, kayakken
☐ Boogschieten
☐ Water polo en zwemmen
☐ Kunnen lopen op oneven grond
☐ Geen sport, echter geen limitatie in dagelijkse activiteiten
☐ Kunnen lopen op even grond, maar de dagelijkste activiteiten zijn gelimiteerd
☐ Niet kunnen lopen, fysieke handicap wegens enkelproblemen

4.1.1 ***If 'Wat is het meest bij u van toepassing ten aanzien van uw acitviteit?' is equal to 'American Football' answer this question:***
American Football

◯ Professioneel
◯ Competitief
◯ Recreatief

4.1.2 ***If 'Wat is het meest bij u van toepassing ten aanzien van uw acitviteit?' is equal to 'Basketbal' answer this question:***
Basketbal

◯ Professioneel
◯ Competitief
◯ Recreatief

4.1.3 ***If 'Wat is het meest bij u van toepassing ten aanzien van uw acitviteit?' is equal to 'Gymnastiek/turnen' answer this question:***
Gymnastiek/turnen

◯ Professioneel
◯ Competitief
◯ Recreatief

4.1.4 ***If 'Wat is het meest bij u van toepassing ten aanzien van uw acitviteit?' is equal to 'Handbal' answer this question:***
Handbal

◯ Professioneel
◯ Competitief
◯ Recreatief

4.1.5 ***If 'Wat is het meest bij u van toepassing ten aanzien van uw acitviteit?' is equal to 'Rugby' answer this question:***
Rugby

◯ Professioneel
◯ Competitief
◯ Recreatief

4.1.6	***If 'Wat is het meest bij u van toepassing ten aanzien van uw acitviteit?' is equal to 'Voetbal' answer this question:***<br>Voetbal	◯ Professioneel<br>◯ Competitief<br>◯ Recreatief
4.1.7	***If 'Wat is het meest bij u van toepassing ten aanzien van uw acitviteit?' is equal to 'Hockey' answer this question:***<br>Hockey	◯ Professioneel<br>◯ Competitief<br>◯ Recreatief
4.1.8	***If 'Wat is het meest bij u van toepassing ten aanzien van uw acitviteit?' is equal to 'Korfbal' answer this question:***<br>Korfbal	◯ Professioneel<br>◯ Competitief<br>◯ Recreatief
4.1.9	***If 'Wat is het meest bij u van toepassing ten aanzien van uw acitviteit?' is equal to 'Gevechtssporten: judo, karate, kung fu, taekwando, jiu jitsu, etc.' answer this question:***<br>Gevechtssporten: judo, karate, kung fu, taekwando, jiu jitsu, etc.	◯ Professioneel<br>◯ Competitief<br>◯ Recreatief
4.1.10	***If 'Wat is het meest bij u van toepassing ten aanzien van uw acitviteit?' is equal to 'Oriëntatielopen' answer this question:***<br>Oriëntatielopen	◯ Professioneel<br>◯ Competitief<br>◯ Recreatief
4.1.11	***If 'Wat is het meest bij u van toepassing ten aanzien van uw acitviteit?' is equal to 'Rhytmische gymnastiek' answer this question:***<br>Rhytmisch gymnastiek	◯ Professioneel<br>◯ Competitief<br>◯ Recreatief
4.1.12	***If 'Wat is het meest bij u van toepassing ten aanzien van uw acitviteit?' is equal to 'Volleybal' answer this question:***<br>Volleybal	◯ Professioneel<br>◯ Competitief<br>◯ Recreatief
4.1.13	***If 'Wat is het meest bij u van toepassing ten aanzien van uw acitviteit?' is equal to 'Boxen' answer this question:***<br>Boxen	◯ Professioneel<br>◯ Competitief<br>◯ Recreatief
4.1.14	***If 'Wat is het meest bij u van toepassing ten aanzien van uw acitviteit?' is equal to 'Freestyle snowboarden' answer this question:***<br>Freestyle snowboarden	◯ Professioneel<br>◯ Competitief<br>◯ Recreatief
4.1.15	***If 'Wat is het meest bij u van toepassing ten aanzien van uw acitviteit?' is equal to 'Freestyle snowboarden' answer this question:***<br>IJshockey	◯ Professioneel<br>◯ Competitief<br>◯ Recreatief

4.1.16	***If 'Wat is het meest bij u van toepassing ten aanzien van uw acitviteit?' is equal to 'Tennis' answer this question:*** Tennis	◯ Professioneel ◯ Competitief ◯ Recreatief
4.1.17	***If 'Wat is het meest bij u van toepassing ten aanzien van uw acitviteit?' is equal to 'Worstelen' answer this question:*** Worstelen	◯ Professioneel ◯ Competitief ◯ Recreatief
4.1.18	***If 'Wat is het meest bij u van toepassing ten aanzien van uw acitviteit?' is equal to 'Fitness, aerobics' answer this question:*** Fitness, aerobics	◯ Professioneel ◯ Competitief ◯ Recreatief
4.1.19	***If 'Wat is het meest bij u van toepassing ten aanzien van uw acitviteit?' is equal to 'Badminton' answer this question:*** Badminton	◯ Professioneel ◯ Competitief ◯ Recreatief
4.1.20	***If 'Wat is het meest bij u van toepassing ten aanzien van uw acitviteit?' is equal to 'Baseball' answer this question:*** Baseball	◯ Professioneel ◯ Competitief ◯ Recreatief
4.1.21	***If 'Wat is het meest bij u van toepassing ten aanzien van uw acitviteit?' is equal to 'Cross-country hardlopen' answer this question:*** Cross-country hardlopen	◯ Professioneel ◯ Competitief ◯ Recreatief
4.1.22	***If 'Wat is het meest bij u van toepassing ten aanzien van uw acitviteit?' is equal to 'Moderne pentathlon' answer this question:*** Moderne pentathlon	◯ Professioneel ◯ Competitief ◯ Recreatief
4.1.23	***If 'Wat is het meest bij u van toepassing ten aanzien van uw acitviteit?' is equal to 'Squash' answer this question:*** Squash	◯ Professioneel ◯ Competitief ◯ Recreatief
4.1.24	***If 'Wat is het meest bij u van toepassing ten aanzien van uw acitviteit?' is equal to 'Surfen, windsurfen' answer this question:*** Surfen, windsurfen	◯ Professioneel ◯ Competitief ◯ Recreatief
4.1.25	***If 'Wat is het meest bij u van toepassing ten aanzien van uw acitviteit?' is equal to 'Tafel tennis' answer this question:*** Tafel tennis	◯ Professioneel ◯ Competitief ◯ Recreatief

4.1.26	***If 'Wat is het meest bij u van toepassing ten aanzien van uw acitviteit?' is equal to 'Atletiek: spring-, werponderdelen' answer this question:***<br>Atletiek: spring-, werponderdelen	◯ Professioneel<br>◯ Competitief<br>◯ Recreatief
4.1.27	***If 'Wat is het meest bij u van toepassing ten aanzien van uw acitviteit?' is equal to 'Waterskieën' answer this question:***<br>Waterskien	◯ Professioneel<br>◯ Competitief<br>◯ Recreatief
4.1.28	***If 'Wat is het meest bij u van toepassing ten aanzien van uw acitviteit?' is equal to 'Dans' answer this question:***<br>Dans	◯ Professioneel<br>◯ Competitief<br>◯ Recreatief
4.1.29	***If 'Wat is het meest bij u van toepassing ten aanzien van uw acitviteit?' is equal to 'Schermen' answer this question:***<br>Schermen	◯ Professioneel<br>◯ Competitief<br>◯ Recreatief
4.1.30	***If 'Wat is het meest bij u van toepassing ten aanzien van uw acitviteit?' is equal to 'Zaalhockey' answer this question:***<br>Zaalhockey	◯ Professioneel<br>◯ Competitief<br>◯ Recreatief
4.1.31	***If 'Wat is het meest bij u van toepassing ten aanzien van uw acitviteit?' is equal to 'Bergbeklimmen' answer this question:***<br>Bergbeklimmen	◯ Professioneel<br>◯ Competitief<br>◯ Recreatief
4.1.32	***If 'Wat is het meest bij u van toepassing ten aanzien van uw acitviteit?' is equal to 'Langlauf' answer this question:***<br>Langlauf	◯ Professioneel<br>◯ Competitief<br>◯ Recreatief
4.1.33	***If 'Wat is het meest bij u van toepassing ten aanzien van uw acitviteit?' is equal to 'Parachute springen' answer this question:***<br>Parachute springen	◯ Professioneel<br>◯ Competitief<br>◯ Recreatief
4.1.34	***If 'Wat is het meest bij u van toepassing ten aanzien van uw acitviteit?' is equal to 'Softball' answer this question:***<br>Softball	◯ Professioneel<br>◯ Competitief<br>◯ Recreatief

4.1.35	***If 'Wat is het meest bij u van toepassing ten aanzien van uw acitviteit?' is equal to 'Speciale beroepen en werkactiviteiten; speciale beroepen: ballet, professioneel soldaat, speciale reddingswerker, stuntman, etc.' answer this question:*** Speciale beroepen en werkactiviteiten speciale beroepen: ballet, professioneel soldaat, speciale reddingswerker, stuntman, etc.	◯ ballet, professioneel soldaat, speciale reddingswerker, stuntman, etc.
4.1.36	***If 'Wat is het meest bij u van toepassing ten aanzien van uw acitviteit?' is equal to 'Duiken' answer this question:*** Duiken	◯ Professioneel/competitief<br>◯ Recreatief
4.1.37	***If 'Wat is het meest bij u van toepassing ten aanzien van uw acitviteit?' is equal to 'Scubaduiken' answer this question:*** Scubaduiken	◯ Professioneel/competitief<br>◯ Recreatief
4.1.38	***If 'Wat is het meest bij u van toepassing ten aanzien van uw acitviteit?' is equal to 'Skaten, in-line skaten' answer this question:*** skaten, in-linen skaten	◯ Professioneel/competitief<br>◯ Recreatief
4.1.39	***If 'Wat is het meest bij u van toepassing ten aanzien van uw acitviteit?' is equal to 'Atletiek: looponderdelen' answer this question:*** Atletiek: looponderdelen	◯ Professioneel/competitief<br>◯ Recreatief
4.1.40	***If 'Wat is het meest bij u van toepassing ten aanzien van uw acitviteit?' is equal to 'Triatlon' answer this question:*** triatlon	◯ Professioneel/competitief<br>◯ Recreatief
4.1.41	***If 'Wat is het meest bij u van toepassing ten aanzien van uw acitviteit?' is equal to 'Gewichtsheffen, body-building' answer this question:*** Gewichtsheffen, body-building	◯ Professioneel/competitief<br>◯ Recreatief
4.1.42	***If 'Wat is het meest bij u van toepassing ten aanzien van uw acitviteit?' is equal to 'Alle competitieve sporten hieronder met 'seasonal' conditioning' answer this question:*** Alle competitieve sporten hieronder met 'seasonal' conditioning	◯ Alle competitieve sporten hieronder met 'seasonal' conditioning
4.1.43	***If 'Wat is het meest bij u van toepassing ten aanzien van uw acitviteit?' is equal to 'Zwaar fysiek werk' answer this question:*** Zwaar fysiek werk	◯ Alle competitieve sporten hieronder met 'seasonal' conditioning

4.1.44	***If 'Wat is het meest bij u van toepassing ten aanzien van uw acitviteit?' is equal to 'Alpine skiën en snowboarden' answer this question:*** Alpine skien en snowboarden	○ Alpine skien en snowboarden
4.1.45	***If 'Wat is het meest bij u van toepassing ten aanzien van uw acitviteit?' is equal to 'Bowlen/curlen' answer this question:*** Bowlen/curlen	○ Bowlen/curlen
4.1.46	***If 'Wat is het meest bij u van toepassing ten aanzien van uw acitviteit?' is equal to 'Golf' answer this question:*** Golf	○ Golf
4.1.47	***If 'Wat is het meest bij u van toepassing ten aanzien van uw acitviteit?' is equal to 'Mountainbike/BMX' answer this question:*** Mountainbike/BMX	○ Mountainbike/BMX
4.1.48	***If 'Wat is het meest bij u van toepassing ten aanzien van uw acitviteit?' is equal to 'Powerliften' answer this question:*** Powerliften	○ Powerliften
4.1.49	***If 'Wat is het meest bij u van toepassing ten aanzien van uw acitviteit?' is equal to 'Zeilen' answer this question:*** Zeilen	○ Zeilen
4.1.50	***If 'Wat is het meest bij u van toepassing ten aanzien van uw acitviteit?' is equal to 'Fysiek werk' answer this question:*** Fysiek werk	○ Fysiek werk
4.1.51	***If 'Wat is het meest bij u van toepassing ten aanzien van uw acitviteit?' is equal to 'Wielrennen' answer this question:*** Wielrennen	○ Wielrennen
4.1.52	***If 'Wat is het meest bij u van toepassing ten aanzien van uw acitviteit?' is equal to 'Paardrijden' answer this question:*** Paardrijden	○ Paardrijden
4.1.53	***If 'Wat is het meest bij u van toepassing ten aanzien van uw acitviteit?' is equal to 'Motorsporten/technische sporten' answer this question:*** Motorsporten/technische sporten	○ Motorsporten/technische sporten
4.1.54	***If 'Wat is het meest bij u van toepassing ten aanzien van uw acitviteit?' is equal to 'Roeien, kayakken' answer this question:*** Roeien, kayakken	○ Roeien, kayakken

4.1.55	***If 'Wat is het meest bij u van toepassing ten aanzien van uw acitviteit?' is equal to 'Boogschieten' answer this question:*** Boogschieten	○ Boogschieten
4.1.56	***If 'Wat is het meest bij u van toepassing ten aanzien van uw acitviteit?' is equal to 'Water polo en zwemmen' answer this question:*** Water polo en zwemmen	○ Water polo en zwemmen
4.1.57	***If 'Wat is het meest bij u van toepassing ten aanzien van uw acitviteit?' is equal to 'Kunnen lopen op oneven grond' answer this question:*** Kunnen lopen op oneven grond	○ Kunnen lopen op oneven grond
4.1.58	***If 'Wat is het meest bij u van toepassing ten aanzien van uw acitviteit?' is equal to 'Geen sport, echter geen limitatie in dagelijkse activiteiten' answer this question:*** Geen sport, echter geen limitatie in dagelijkse activiteiten	○ Geen sport, echter geen limitatie in dagelijkse activiteiten
4.1.59	***If 'Wat is het meest bij u van toepassing ten aanzien van uw acitviteit?' is equal to 'Kunnen lopen op even grond, maar de dagelijkste activiteiten zijn gelimiteerd' answer this question:*** Kunnen lopen op even grond, maar de dagelijkste activiteiten zijn gelimiteerd	○ Kunnen lopen op even grond, maar de dagelijkste activiteiten zijn gelimiteerd
4.1.60	***If 'Wat is het meest bij u van toepassing ten aanzien van uw acitviteit?' is equal to 'Niet kunnen lopen, fysieke handicap wegens enkelproblemen' answer this question:*** Niet kunnen lopen, fysieke handicap wegens enkelproblemen	○ Niet kunnen lopen, fysieke handicap wegens enkelproblemen

Vragenlijst PRIMA studie 12 weken - Hoe tevreden bent u over de enkelklachten?

Number	Question	Answers
5.1	Hoe tevreden bent u over de enkelklachten?	○ Slecht<br>○ Matig<br>○ Goed<br>○ Uitstekend

Vragenlijst PRIMA studie 12 weken - SF-36

Number	Question	Answers
	Deze vragenlijst gaat over uw standpunten t.a.v. uw gezondheid. Met behulp van deze gegevens kan worden bijgehouden hoe u zich voelt en hoe goed u in staat bent uw gebruikelijke bezigheden uit te voeren. Beantwoord elke vraag door een antwoord aan te klikken. Als u niet zeker weet hoe u een vraag moet beantwoorden, geef dan het best mogelijke antwoord.	
6.1	Hoe zou u over het algemeen uw gezondheid noemen?	◯ Uitstekend ◯ Zeer goed ◯ Goed ◯ Matig ◯ Slecht
6.2	Hoe beoordeelt u nu uw gezondheid over het algemeen vergeleken met een jaar geleden?	◯ Veel beter dan een jaar geleden ◯ Wat beter dan een jaar geleden ◯ Ongeveer hetzelfde als een jaar geleden ◯ Wat slechter dan een jaar geleden ◯ Veel slechter dan een jaar geleden
6.3	Wordt u door uw gezondheid op dit moment beperkt bij forse inspanning, zoals hardlopen, tillen van zware voorwerpen of een veeleisende sport beoefenen?	◯ Ja, ernstig beperkt ◯ Ja, een beetje beperkt ◯ Nee, helemaal niet beperkt
6.4	Wordt u door uw gezondheid op dit moment beperkt bij matige inspanning zoals een tafel verplaatsen, stofzuigen, zwemmen of fietsen?	◯ Ja, ernstig beperkt ◯ Ja, een beetje beperkt ◯ Nee, helemaal niet beperkt
6.5	Wordt u door uw gezondheid op dit moment beperkt bij boodschappen tillen of dragen?	◯ Ja, ernstig beperkt ◯ Ja, een beetje beperkt ◯ Nee, helemaal niet beperkt
6.6	Wordt u door uw gezondheid op dit moment beperkt bij een paar trappen oplopen?	◯ Ja, ernstig beperkt ◯ Ja, een beetje beperkt ◯ Nee, helemaal niet beperkt
6.7	Wordt u door uw gezondheid op dit moment beperkt bij één trap oplopen?	◯ Ja, ernstig beperkt ◯ Ja, een beetje beperkt ◯ Nee, helemaal niet beperkt
6.8	Wordt u door uw gezondheid op dit moment beperkt bij bukken, knielen of hurken?	◯ Ja, ernstig beperkt ◯ Ja, een beetje beperkt ◯ Nee, helemaal niet beperkt
6.9	Wordt u door uw gezondheid op dit moment beperkt bij meer dan één kilometer lopen?	◯ Ja, ernstig beperkt ◯ Ja, een beetje beperkt ◯ Nee, helemaal niet beperkt
6.10	Wordt u door uw gezondheid op dit moment beperkt bij een paar honderd meter lopen?	◯ Ja, ernstig beperkt ◯ Ja, een beetje beperkt ◯ Nee, helemaal niet beperkt

6.11	Wordt u door uw gezondheid op dit moment beperkt bij ongeveer honderd meter lopen?	◯ Ja, ernstig beperkt ◯ Ja, een beetje beperkt ◯ Nee, helemaal niet beperkt
6.12	Wordt u door uw gezondheid op dit moment beperkt bij uzelf wassen of aankleden?	◯ Ja, ernstig beperkt ◯ Ja, een beetje beperkt ◯ Nee, helemaal niet beperkt
6.13	U besteedde in de afgelopen 4 weken minder tijd aan werk of andere bezigheden	◯ Ja ◯ Nee
6.14	U heeft in de afgelopen 4 weken minder bereikt dan u zou willen	◯ Ja ◯ Nee
6.15	U was in de afgelopen 4 weken beperkt in het soort werk of andere bezigheden.	◯ Ja ◯ Nee
6.16	U had de afgelopen 4 weken moeite om uw werk of andere bezigheden uit te voeren (het kostte u bijvoorbeeld extra inspanning).	◯ Ja ◯ Nee
6.17	U besteedde in de afgelopen 4 weken minder tijd aan werk of andere bezigheden ten gevolge van emotionele problemen	◯ Ja ◯ Nee
6.18	U heeft in de afgelopen 4 weken minder bereikt dan u zou willen ten gevolge van emotionele problemen.	◯ Ja ◯ Nee
6.19	U deed de afgelopen 4 weken uw werk of andere bezigheden niet zo zorgvuldig als gewoonlijk ten gevolge van emotionele problemen.	◯ Ja ◯ Nee
6.20	In hoeverre hebben uw lichamelijke gezondheid of emotionele problemen u gedurende de afgelopen 4 weken gehinderd in uw normale omgang met familie, vrienden of buren, of bij activiteiten in groepsverband?	◯ Helemaal niet ◯ Enigszins ◯ Nogal ◯ Veel ◯ Heel erg veel
6.21	Hoeveel lichamelijke pijn heeft u de afgelopen 4 weken gehad?	◯ Geen<br>◯ Heel licht<br>◯ Licht<br>◯ Nogal<br>◯ Ernstig<br>◯ Heel ernstig
6.22	In welke mate bent u de afgelopen 4 weken door de pijn gehinderd in uw normale werk (zowel werk buitenshuis als huishoudelijk werk)?	◯ Helemaal niet<br>◯ Een klein beetje<br>◯ Nogal<br>◯ Veel<br>◯ Heel erg veel

6.23	Voelde u zich levenslustig?	◯ Altijd<br>◯ Meestal<br>◯ Vaak<br>◯ Soms<br>◯ Zelden<br>◯ Nooit
6.24	Was u erg zenuwachtig?	◯ Altijd<br>◯ Meestal<br>◯ Vaak<br>◯ Soms<br>◯ Zelden<br>◯ Nooit
6.25	Zat u zo in de put dat u niets kon?	◯ Altijd<br>◯ Meestal<br>◯ Vaak<br>◯ Soms<br>◯ Zelden<br>◯ Nooit
6.26	Voelde u zich rustig en tevreden?	◯ Altijd<br>◯ Meestal<br>◯ Vaak<br>◯ Soms<br>◯ Zelden<br>◯ Nooit
6.27	Had u veel energie?	◯ Altijd<br>◯ Meestal<br>◯ Vaak<br>◯ Soms<br>◯ Zelden<br>◯ Nooit
6.28	Voelde u zich somber en neerslachtig?	◯ Altijd<br>◯ Meestal<br>◯ Vaak<br>◯ Soms<br>◯ Zelden<br>◯ Nooit

6.29	Voelde u zich uitgeput?	◯ Altijd<br>◯ Meestal<br>◯ Vaak<br>◯ Soms<br>◯ Zelden<br>◯ Nooit
6.30	Was u een gelukkig mens?	◯ Altijd<br>◯ Meestal<br>◯ Vaak<br>◯ Soms<br>◯ Zelden<br>◯ Nooit
6.31	Voelde u zich moe?	◯ Altijd<br>◯ Meestal<br>◯ Vaak<br>◯ Soms<br>◯ Zelden<br>◯ Nooit
6.32	Hoe vaak hebben uw lichamelijke gezondheid of emotionele problemen u gedurende de afgelopen 4 weken gehinderd bij uw sociale activiteiten (zoals vrienden of familie bezoeken etc.)?	◯ Altijd<br>◯ Meestal<br>◯ Soms<br>◯ Zelden<br>◯ Nooit
6.33	Ik lijk wat gemakkelijker ziek te worden dan andere mensen	◯ Volkomen juist<br>◯ Grotendeels juist<br>◯ Weet ik niet<br>◯ Grotendeels onjuist<br>◯ Volkomen onjuist
6.34	Ik ben even gezond als andere mensen die ik ken	◯ Volkomen juist<br>◯ Grotendeels juist<br>◯ Weet ik niet<br>◯ Grotendeels onjuist<br>◯ Volkomen onjuist

6.35	Ik verwacht dat mijn gezondheid achteruit zal gaan	◯ Volkomen juist<br>◯ Grotendeels juist<br>◯ Weet ik niet<br>◯ Grotendeels onjuist<br>◯ Volkomen onjuist
6.36	Mijn gezondheid is uitstekend	◯ Volkomen juist<br>◯ Grotendeels juist<br>◯ Weet ik niet<br>◯ Grotendeels onjuist<br>◯ Volkomen onjuist

Vragenlijst PRIMA studie 12 weken - GAS

Number	Question	Answers
	Tijdens de eerste afspraak werd een duidelijk doel met u afgesproken. Dit werd tevens per email naar u gestuurd. Hoe is de situatie nu ten aanzien van de destijds gestelde doelstelling?	
7.1	Wat is nu de situatie ten aanzien van het tijdens de eerste afspraak afgesproken doelstelling (Goal attainment Scaling)?	◯ Achteruitgang (minder dan de utigangssituatie)<br>◯ Uitgangssituatie<br>◯ Minder dan het doel<br>◯ Doel<br>◯ Meer dan het doel<br>◯ Veel meer dan het doel

Vragenlijst PRIMA studie 12 weken - EQ-5D-3L

Number	Question	Answers
	Deze vragenlijst gaat over uw standpunten t.a.v. uw gezondheid. Met behulp van deze gegevens kan worden bijgehouden hoe u zich voelt en hoe goed u in staat bent uw gebruikelijke bezigheden uit te voeren. Beantwoord elke vraag door een antwoord aan te klikken. Als u niet zeker weet hoe u een vraag moet beantwoorden, geef dan het best mogelijke antwoord.	
8.1	Hoe is het met uw Mobiliteit gesteld?	◯ Ik heb geen problemen met lopen<br>◯ Ik heb enige problemen met lopen<br>◯ Ik ben bedlegerig

For peer review only - http://bmjopen.bmj.com/site/about/guidelines.xhtml

8.2	Hoe is het met uw Zelfzorg gesteld?	◯ Ik heb geen problemen om mijzelf te wassen of aan te kleden<br>◯ Ik heb enige problemen om mijzelf te wassen of aan te kleden<br>◯ Ik ben niet in staat om mijzelf te wassen of aan te kleden
8.3	Hoe is het met de Dagelijkse activiteiten (werk, studie, huishouden, gezins- en vrijetijdsactiviteiten) gesteld?	◯ Ik heb geen problemen met mijn dagelijkse activiteiten<br>◯ Ik heb enige problemen met mijn dagelijkse activiteiten<br>◯ Ik ben niet in staat om mijn dagelijkse activiteiten uit te voeren
8.4	Hoe is het met de Pijn/klachten gesteld?	◯ Ik heb geen pijn of andere klachten<br>◯ Ik heb matige pijn of andere klachten<br>◯ Ik heb zeer ernstige pijn of andere klachten
8.5	Hoe is het met de Stemming gesteld?	◯ Ik ben niet angstig of somber<br>◯ Ik ben matig angstig of somber<br>◯ Ik ben erg angstig of somber
	We willen weten hoe goed of slecht uw gezondheid VANDAAG is. Deze meetschaal loopt van 0 tot 100: 100 staat voor de BESTE gezondheid die u zich kunt voorstellen - 0 staat voor de SLECHTSTE gezondheid die u zich kunt voorstellen	
8.6	Uw Gezondheid vandaag	(0.00) (100.00)

Vragenlijst PRIMA studie 12 weken - AOS

Number	Question	Answers
	Instructies: De lijn naast elke vraag staat voor hoeveel PIJN u heeft in verschillende situaties. De linker kant (0) is “geen pijn” en de rechter kant (100) is “ergste pijn denkbaar”. Geef voor de onderstaande situaties op de lijn aan hoeveel PIJN u in de afgelopen week in de enkel had. Als een of meerdere van deze situaties niet van toepassing waren, dan kiest u de "niet van toepassing" optie.	
9.1	Wat was de hoogte van de ergste pijn in de afgelopen week?	(0.00) (100.00)
9.2	Heeft u pijn als u 's ochtends opstaat?	◯ Ja<br>◯ Nee<br>◯ Niet van toepassing

9.2.1	***If 'Heeft u pijn als u 's ochtends opstaat?' is equal to 'Ja' answer this question:***<br>Hoeveel pijn heeft u voordat u 's ochtends opstaat?	(0.00)	(100.00)
9.3	Heeft u pijn wanneer u op blote voeten loopt?	◯ Ja<br>◯ Nee<br>◯ Niet van toepassing	
9.3.1	***If 'Heeft u pijn wanneer u op blote voeten loopt?' is equal to 'Ja' answer this question:***<br>Hoeveel pijn heeft u als u op blote voeten loopt?	(0.00)	(100.00)
9.4	Heeft u pijn wanneer u op blote voeten staat?	◯ Ja<br>◯ Nee<br>◯ Niet van toepassing	
9.4.1	***If 'Heeft u pijn wanneer u op blote voeten staat?' is equal to 'Ja' answer this question:***<br>Hoeveel pijn heeft u als u op blote voeten staat?	(0.00)	(100.00)
9.5	Heeft u pijn wanneer u met schoenen loopt?	◯ Ja<br>◯ Nee<br>◯ Niet van toepassing	
9.5.1	***If 'Heeft u pijn wanneer u met schoenen loopt?' is equal to 'Ja' answer this question:***<br>Hoeveel pijn heeft u wanneer u met schoenen loopt?	(0.00)	(100.00)
9.6	Heeft u pijn wanneer u met schoenen staat?	◯ Ja<br>◯ Nee<br>◯ Niet van toepassing	
9.6.1	***If 'Heeft u pijn wanneer u met schoenen staat?' is equal to 'Ja' answer this question:***<br>Hoeveel pijn heeft u wanneer u met schoenen staat?	(0.00)	(100.00)
9.7	Heeft u pijn wanneer u loopt met steunzolen of een brace?	◯ Ja<br>◯ Nee<br>◯ Niet van toepassing	
9.7.1	***If 'Heeft u pijn wanneer u loopt met steunzolen of een brace?' is equal to 'Ja' answer this question:***<br>Hoeveel pijn heeft u wanneer u loopt met steunzolen of een brace?	(0.00)	(100.00)

9.8	Heeft u pijn wanneer u staat met steunzolen of een brace?	◯ Ja<br>◯ Nee<br>◯ Niet van toepassing	
9.8.1	***If 'Heeft u pijn wanneer u staat met steunzolen of een brace?' is equal to 'Ja' answer this question:***<br>Hoeveel pijn heeft u wanneer u staat met steunzolen of een brace?	(0.00)	(100.00)
9.9	Heeft u pijn aan het einde van de dag?	◯ Ja<br>◯ Nee<br>◯ Niet van toepassing	
9.9.1	***If 'Heeft u pijn aan het einde van de dag?' is equal to 'Ja' answer this question:***<br>Hoeveel pijn heeft u aan het einde van de dag?	(0.00)	(100.00)
	Instructies:De lijn naast elke vraag staat voor hoeveel MOEITE u heeft met verschillende activiteiten. De linker kant (0) is "Niet moeilijk" en de rechter kant (100) is "Te moeilijk, niet uitvoerbaar". Geef voor de onderstaande activiteiten op de lijn aan hoeveel MOEITE u in de afgelopen week door enkelklachten had met onderstaande activiteiten. Als een situatie niet van toepassing is, kies dan de optie "niet van toepassing".		
9.10	Heeft u moeite met door het huis lopen?	◯ Ja<br>◯ Nee<br>◯ Niet van toepassing	
9.10.1	***If 'Heeft u moeite met door het huis lopen?' is equal to 'Ja' answer this question:***<br>Hoeveel moeite heeft u met door het huis lopen?	(0.00)	(100.00)
9.11	Heeft u moeite met buiten lopen op oneven ondergrond?	◯ Ja<br>◯ Nee<br>◯ Niet van toepassing	
9.11.1	***If 'Heeft u moeite met buiten lopen op oneven ondergrond?' is equal to 'Ja' answer this question:***<br>Hoeveel moeite heeft u met buiten lopen op oneven ondergrond?	(0.00)	(100.00)
9.12	Heeft u moeite met een paar honderd meter lopen?	◯ Ja<br>◯ Nee<br>◯ Niet van toepassing	
9.12.1	***If 'Heeft u moeite met een paar honderd meter lopen?' is equal to 'Ja' answer this question:***<br>Hoeveel moeite heeft u met een paar honderd meter lopen?	(0.00)	(100.00)

9.13	Heeft u moeite met een trap oplopen?	◯ Ja<br>◯ Nee<br>◯ Niet van toepassing	
9.13.1	***If 'Heeft u moeite met een trap oplopen?' is equal to 'Ja' answer this question:***<br>Hoeveel moeite heeft u met een trap oplopen?	(0.00)	(100.00)
9.14	Heeft u moeite met een trap aflopen?	◯ Ja<br>◯ Nee<br>◯ Niet van toepassing	
9.14.1	***If 'Heeft u moeite met een trap aflopen?' is equal to 'Ja' answer this question:***<br>Hoeveel moeite heeft u met een trap aflopen?	(0.00)	(100.00)
9.15	Heeft u moeite met op de tenen staan?	◯ Ja<br>◯ Nee<br>◯ Niet van toepassing	
9.15.1	***If 'Heeft u moeite met op de tenen staan?' is equal to 'Ja' answer this question:***<br>Hoeveel moeite heeft u met op de tenen staan?	(0.00)	(100.00)
9.16	Heeft u moeite met opstaan uit de stoel?	◯ Ja<br>◯ Nee<br>◯ Niet van toepassing	
9.16.1	***If 'Heeft u moeite met opstaan uit de stoel?' is equal to 'Ja' answer this question:***<br>Hoeveel moeite heeft u met opstaan uit de stoel?	(0.00)	(100.00)
9.17	Heeft u moeite met het op- of afstappen van stoepranden?	◯ Ja<br>◯ Nee<br>◯ Niet van toepassing	
9.17.1	***If 'Heeft u moeite met het op- of afstappen van stoepranden?' is equal to 'Ja' answer this question:***<br>Hoeveel moeite heeft u met het op- of afstappen van stoepranden?	(0.00)	(100.00)
9.18	Heeft u moeite met snel lopen of rennen?	◯ Ja<br>◯ Nee<br>◯ Niet van toepassing	

9.18.1	***If 'Heeft u moeite met snel lopen of rennen?' is equal to 'Ja' answer this question:*** Hoeveel moeite heeft u met snel lopen of rennen?	(0.00)	(100.00)

Vragenlijst PRIMA studie 12 weken - FAOS

Number	Question	Answers
	Deze lijst vraagt naar uw mening over uw voet/enkel. Uw antwoorden geven ons een beeld van uw voet/enkel klachten en hoe u in staat bent om alledaagse activiteiten uit te voeren in uw huidige situatie. Beantwoorden van een vraag doet u door het aanklikken van een vakje met het volgens u meest juiste antwoord (één vakje per vraag). Als u niet zeker weet hoe u een vraag moet beantwoorden, geeft u dan het antwoord dat volgens u het meest op uw situatie van toepassing is. Deze vraag heeft betrekking op het voorkomen van voet/enkel klachten in de afgelopen week.	
10.1	Is uw voet/enkel gezwollen?	◯ Nooit ◯ Zelden ◯ Soms ◯ Vaak ◯ Altijd
10.2	Voelt u gekraak of hoort u klikken of een ander vreemd geluid wanneer u de voet/enkel beweegt?	◯ Nooit ◯ Zelden ◯ Soms ◯ Vaak ◯ Altijd
10.3	Hapert uw enkel of blokkeert uw enkel ('op slot' gaan zitten) wanneer u deze beweegt?	◯ Nooit ◯ Zelden ◯ Soms ◯ Vaak ◯ Altijd
10.4	Kunt u de voet/enkel volledig strekken?	◯ Altijd ◯ Vaak ◯ Soms ◯ Zelden ◯ Nooit
10.5	Kunt u de voet volledig naar u toe buigen?	◯ Altijd ◯ Vaak ◯ Soms ◯ Zelden ◯ Nooit
10.6	In welke mate heeft u een stijf gevoel in de voet/enkel 's ochtends bij het wakker worden?	◯ Niet ◯ Mild ◯ Matig ◯ Ernstig ◯ Zeer ernstig
10.7	In welke mate heeft u een stijf gevoel in de voet/enkel na zitten, liggen of rusten later op de dag?	◯ Niet ◯ Mild ◯ Matig ◯ Ernstig ◯ Zeer ernstig
10.8	Hoe vaak heeft u pijn in uw voet/enkel?	◯ Nooit ◯ Maandelijks ◯ Wekelijks ◯ Dagelijks ◯ Altijd

10.9	Hoeveel voet/enkel pijn heeft u gehad in de afgelopen week bij draaien als uw voet/enkel op de grond staat?	◯ Geen<br>◯ Mild<br>◯ Matig<br>◯ Ernstig<br>◯ Zeer ernstig
10.10	Hoeveel voet/enkel pijn heeft u gehad in de afgelopen week bij het volledig uitstrekken van de voet/enkel?	◯ Geen<br>◯ Mild<br>◯ Matig<br>◯ Ernstig<br>◯ Zeer ernstig
10.11	Hoeveel voet/enkel pijn heeft u gehad in de afgelopen week bij het volledig naar u toe buigen/optrekken van de voet/enkel	◯ Geen<br>◯ Mild<br>◯ Matig<br>◯ Ernstig<br>◯ Zeer ernstig
10.12	Hoeveel voet/enkel pijn heeft u gehad in de afgelopen week bij het lopen op een vlakke ondergrond	◯ Geen<br>◯ Mild<br>◯ Matig<br>◯ Ernstig<br>◯ Zeer ernstig
10.13	Hoeveel voet/enkel pijn heeft u gehad in de afgelopen week bij het trap op- en trap af lopen?	◯ Geen<br>◯ Mild<br>◯ Matig<br>◯ Ernstig<br>◯ Zeer ernstig
10.14	Hoeveel voet/enkel pijn heeft u gehad in de afgelopen week 's nachts in bed?	◯ Geen<br>◯ Mild<br>◯ Matig<br>◯ Ernstig<br>◯ Zeer ernstig
10.15	Hoeveel voet/enkel pijn heeft u gehad in de afgelopen week bij het zitten of liggen?	◯ Geen<br>◯ Mild<br>◯ Matig<br>◯ Ernstig<br>◯ Zeer ernstig

For peer review only - http://bmjopen.bmj.com/site/about/guidelines.xhtml

10.16	Hoeveel voet/enkel pijn heeft u gehad in de afgelopen week bij het rechtop staan?	◯ Geen<br>◯ Mild<br>◯ Matig<br>◯ Ernstig<br>◯ Zeer ernstig
10.17	In welke mate werd u gehinderd bij het trap aflopen?	◯ Niet ◯ Mild ◯ Matig ◯ Ernstig ◯ Zeer ernstig
10.18	In welke mate werd u gehinderd bij het trap op lopen?	◯ Niet ◯ Mild ◯ Matig ◯ Ernstig ◯ Zeer ernstig
10.19	In welke mate werd u gehinderd als u vanuit een zittende positie ging staan?	◯ Niet ◯ Mild ◯ Matig ◯ Ernstig ◯ Zeer ernstig
10.20	In welke mate werd u gehinderd bij het staan?	◯ Niet ◯ Mild ◯ Matig ◯ Ernstig ◯ Zeer ernstig
10.21	In welke mate werd u gehinderd bij het naar de grond buigen/iets oprapen?	◯ Niet ◯ Mild ◯ Matig ◯ Ernstig ◯ Zeer ernstig
10.22	In welke mate werd u gehinderd bij het lopen op een vlakke ondergrond?	◯ Niet ◯ Mild ◯ Matig ◯ Ernstig ◯ Zeer ernstig
10.23	In welke mate werd u gehinderd bij het in- en uit de auto stappen?	◯ Niet ◯ Mild ◯ Matig ◯ Ernstig ◯ Zeer ernstig
10.24	In welke mate werd u gehinderd bij het boodschappen doen?	◯ Niet ◯ Mild ◯ Matig ◯ Ernstig ◯ Zeer ernstig
10.25	In welke mate werd u gehinderd bij sokken/panty's aantrekken?	◯ Niet ◯ Mild ◯ Matig ◯ Ernstig ◯ Zeer ernstig
10.26	In welke mate werd u gehinderd bij het opstaan uit bed?	◯ Niet ◯ Mild ◯ Matig ◯ Ernstig ◯ Zeer ernstig
10.27	In welke mate werd u gehinderd bij het sokken uittrekken?	◯ Niet ◯ Mild ◯ Matig ◯ Ernstig ◯ Zeer ernstig
10.28	In welke mate werd u gehinderd bij het in bed liggen (omdraaien, lange tijd uw voet/enkel in dezelfde positie houden)?	◯ Niet ◯ Mild ◯ Matig ◯ Ernstig ◯ Zeer ernstig

10.29	In welke mate werd u gehinderd bij het in/uit bad stappen?	◯ Niet ◯ Mild ◯ Matig ◯ Ernstig ◯ Zeer ernstig
10.30	In welke mate werd u gehinderd bij zitten?	◯ Niet ◯ Mild ◯ Matig ◯ Ernstig ◯ Zeer ernstig
10.31	In welke mate werd u gehinderd bij het toilet op en af gaan?	◯ Niet ◯ Mild ◯ Matig ◯ Ernstig ◯ Zeer ernstig
10.32	In welke mate werd u gehinderd bij zwaar huishoudelijk werk (bijvoorbeeld zware dozen sjouwen, vloer schrobben)?	◯ Niet ◯ Mild ◯ Matig ◯ Ernstig ◯ Zeer ernstig
10.33	In welke mate werd u gehinderd bij licht huishoudeijk werk (bijvoorbeeld koken, afstoffen)?	◯ Niet ◯ Mild ◯ Matig ◯ Ernstig ◯ Zeer ernstig
10.34	In welke mate werd u gehinderd bij hurken?	◯ Niet ◯ Mild ◯ Matig ◯ Ernstig ◯ Zeer ernstig
10.35	In welke mate werd u gehinderd bij hardlopen?	◯ Niet ◯ Mild ◯ Matig ◯ Ernstig ◯ Zeer ernstig
10.36	In welke mate werd u gehinderd bij springen?	◯ Niet ◯ Mild ◯ Matig ◯ Ernstig ◯ Zeer ernstig
10.37	In welke mate werd u gehinderd bij ronddraaien op uw aangedane voet/enkel?	◯ Niet ◯ Mild ◯ Matig ◯ Ernstig ◯ Zeer ernstig
10.38	In welke mate werd u gehinderd bij knielen?	◯ Niet ◯ Mild ◯ Matig ◯ Ernstig ◯ Zeer ernstig
10.39	Hoe vaak bent u zich bewust van uw voet/enkel probleem?	◯ Nooit ◯ Maandelijks ◯ Wekelijks ◯ Dagelijks ◯ Altijd
10.40	Heeft u uw leven veranderd om activiteiten te vermijden die schadelijk kunnen zijn voor uw voet/enkel?	◯ Niet<br>◯ Enigszins<br>◯ Matig<br>◯ Behoorlijk<br>◯ Volledig

10.41	In hoeverre kunt u op uw voet/enkel vertrouwen?	◯ Volledig<br>◯ Behoorlijk<br>◯ Matig<br>◯ Enigzins<br>◯ Niet
10.42	In het algemeen, in welke mate ondervindt u hinder van uw voet/enkel	◯ Geen<br>◯ Mild<br>◯ Matig<br>◯ Ernstig<br>◯ Zeer ernstig

Vragenlijst PRIMA studie 12 weken - PRODISQ - Gezondheid en werk

Number	Question	Answers
11.1	Wat is uw leeftijd?	[ ] jaar
11.2	Wat is uw geslacht?	◯ Man<br>◯ Vrouw
11.3	Wat is de hoogste opleiding die u heeft afgemaakt (Zoek uw hoogste opleiding en kruis het hokje daarvoor aan)?	☐ Ik heb geen school of opleiding afgemaakt<br>☐ Lagere school of basisschool<br>☐ Huishoudschool, vbo, lbo, lts, leao of lhno<br>☐ Mavo, mulo, ivo of vmbo<br>☐ Mbo, mts, meao, mhno, inas of intas<br>☐ Havo, vwo, hbs, mms, atheneum of gymnasium<br>☐ Hbo, hts, heao of hhno<br>☐ Universiteit<br>☐ Ik heb een andere opleiding afgemaakt, namelijk
11.3.1	***If 'Wat is de hoogste opleiding die u heeft afgemaakt (Zoek uw hoogste opleiding en kruis het hokje daarvoor aan)?' is equal to 'Ik heb een andere opleiding afgemaakt, namelijk' answer this question:***<br>Wat is de hoogste opleiding die u heeft afgemaakt?	[ ]

11.4	Wat doet u in het dagelijks leven?	☐ Ik zit op school, ik studeer<br>☐ Ik werk in loondienst<br>☐ Ik ben zelfstandig ondernemer<br>☐ Ik ben huisvrouw, huisman<br>☐ Ik ben werkloos<br>☐ Ik ben arbeidsongeschikt<br>☐ Ik ben met pensioen of prepensioen<br>☐ Ik doe iets anders, namelijk
11.4.1	***If 'Wat doet u in het dagelijks leven?' is equal to 'Ik ben arbeidsongeschikt' answer this question:***<br>Wat doet u in het dagelijks leven?	
11.4.2	***If 'Wat doet u in het dagelijks leven?' is equal to 'Ik doe iets anders, namelijk' answer this question:***<br>Wat doet u in het dagelijks leven?	
11.5	Hebt u betaald werk?	◯ Nee<br>◯ Ja
11.5.1	***If 'Hebt u betaald werk?' is equal to 'Ja' answer this question:***<br>Wat is uw beroep?	
11.5.2	***If 'Hebt u betaald werk?' is equal to 'Ja' answer this question:***<br>Hoeveel uur per week werkt u (Tel alleen de uren waarvoor u betaald wordt)?	uren
11.5.3	***If 'Hebt u betaald werk?' is equal to 'Ja' answer this question:***<br>Hoeveel dagen in de week werkt u?	Dagen
11.5.4	***If 'Hebt u betaald werk?' is equal to 'Ja' answer this question:***<br>Bent u in de afgelopen 4 weken afwezig geweest van uw werk omdat u ziek was?	◯ Nee<br>◯ Ja
11.5.4.1	***If 'Bent u in de afgelopen 4 weken afwezig geweest van uw werk omdat u ziek was?' is equal to 'Ja' answer this question:***<br>Bent u in de afgelopen 4 weken afwezig geweest van uw werk omdat u ziek was (Tel alleen de werkdagen in de afgelopen 4 weken)?	dagen afwezig geweest
11.5.4.2	***If 'Bent u in de afgelopen 4 weken afwezig geweest van uw werk omdat u ziek was?' is equal to 'Ja' answer this question:***<br>Was u langer dan de gehele periode van 4 weken afwezig van uw werk doordat u ziek was?	◯ Nee<br>◯ Ja

11.5.4.2.1	***If 'Was u langer dan de gehele periode van 4 weken afwezig van uw werk doordat u ziek was?' is equal to 'Ja' answer this question:***<br>Wanneer heeft u zich ziek gemeld?	☐ ☐ ☐ *(dd-mm-yyyy)*
11.5.5	***If 'Hebt u betaald werk?' is equal to 'Ja' answer this question:***<br>Waren er in de afgelopen 4 weken, dagen waarop u wel gewerkt heeft, maar tijdens uw werk last had van lichamelijke of psychische problemen?	◯ Ja<br>◯ Nee
11.5.5.1	***If 'Waren er in de afgelopen 4 weken, dagen waarop u wel gewerkt heeft, maar tijdens uw werk last had van lichamelijke of psychische problemen?' is equal to 'Ja' answer this question:***<br>Op hoeveel werkdagen had u tijdens uw werk last van uw lichamelijke of psychische problemen?	☐ werkdagen
11.5.5.2	***If 'Waren er in de afgelopen 4 weken, dagen waarop u wel gewerkt heeft, maar tijdens uw werk last had van lichamelijke of psychische problemen?' is equal to 'Ja' answer this question:***<br>Op de dagen dat u last had, kon u misschien niet zoveel werk doen als normaal. Hoeveel werk kon u op deze dagen gemiddeld doen?	Ik kon op deze dagen niks doen (0.00) — Ik kon net zoveel doen als normaal (10.00)
11.6	Waren er dagen waarop u minder onbetaald werk kon doen door uw lichamelijke of psychische problemen?	◯ Nee<br>◯ Ja
11.6.1	***If 'Waren er dagen waarop u minder onbetaald werk kon doen door uw lichamelijke of psychische problemen?' is equal to 'Ja' answer this question:***<br>Op hoeveel dagen was dit zo?	☐ Dagen
11.6.2	***If 'Waren er dagen waarop u minder onbetaald werk kon doen door uw lichamelijke of psychische problemen?' is equal to 'Ja' answer this question:***<br>Stel dat iemand, bijvoorbeeld uw partner, familielid of een bekende, u op deze dagen had geholpen. En al het onbetaalde werk wat u niet kon doen, voor u had gedaan. Hoeveel uur was die persoon hier op deze dagen dan gemiddeld mee bezig geweest?	☐ uur op deze dagen

Vragenlijst PRIMA studie 12 weken - PRODISQ - Zorggebruik

Number	Question	Answers
	Wij willen graag weten met welke dokters u in de afgelopen 3 maanden een afspraak had. Het gaat om afspraken voor uzelf. Ook andere zorgverleners tellen mee. Bijvoorbeeld de fysiotherapeut.	

12.1	Bent u in de afgelopen 3 maanden naar uw huisarts geweest?	◯ Nee<br>◯ Ja
12.1.1	***If 'Bent u in de afgelopen 3 maanden naar uw huisarts geweest?' is equal to 'Ja' answer this question:***<br>Hoeveel afspraken had u?	[ ] afspraken
12.2	Bent u in de afgelopen 3 maanden in contact geweest met een maatschappelijk werker?	◯ Nee<br>◯ Ja
12.2.1	***If 'Bent u in de afgelopen 3 maanden in contact geweest met een maatschappelijk werker?' is equal to 'Ja' answer this question:***<br>Hoeveel afspraken had u?	[ ] afspraken
12.3	Bent u in de afgelopen 3 maanden naar een fysiotherapeut geweest? Of een caesartherapeut, therapeut mensendieck of een manueel therapeut?	◯ Nee<br>◯ Ja
12.3.1	***If 'Bent u in de afgelopen 3 maanden naar een fysiotherapeut geweest? Of een caesartherapeut, therapeut mensendieck of een manueel therapeut?' is equal to 'Ja' answer this question:***<br>Hoeveel afspraken had u?	[ ] afspraken
12.4	Bent u in de afgelopen 3 maanden naar een ergotherapeut geweest?	◯ Nee<br>◯ Ja
12.4.1	***If 'Bent u in de afgelopen 3 maanden naar een ergotherapeut geweest?' is equal to 'Ja' answer this question:***<br>Hoeveel afspraken had u?	[ ] afspraken
12.5	Bent u in de afgelopen 3 maanden naar een logopedist geweest?	◯ Nee<br>◯ Ja
12.5.1	***If 'Bent u in de afgelopen 3 maanden naar een logopedist geweest?' is equal to 'Ja' answer this question:***<br>Hoeveel afspraken had u?	[ ] afspraken
12.6	Bent u in de afgelopen 3 maanden naar een diëtist geweest?	◯ Nee<br>◯ Ja
12.6.1	***If 'Bent u in de afgelopen 3 maanden naar een diëtist geweest?' is equal to 'Ja' answer this question:***<br>Hoeveel afspraken had u?	[ ] afspraken

12.7	Bent u in de afgelopen 3 maanden naar een homeopaat geweest? Of een acupuncturist?	◯ Nee<br>◯ Ja
12.7.1	***If 'Bent u in de afgelopen 3 maanden naar een homeopaat geweest? Of een acupuncturist?' is equal to 'Ja' answer this question:***<br>Hoeveel afspraken had u?	[ ] afspraken
12.8	Bent u in de afgelopen 3 maanden naar een psycholoog geweest? Of een psychotherapeut of psychiater?	◯ Nee<br>◯ Ja
12.8.1	***If 'Bent u in de afgelopen 3 maanden naar een psycholoog geweest? Of een psychotherapeut of psychiater?' is equal to 'Ja' answer this question:***<br>Hoeveel afspraken had u?	[ ] afspraken
12.9	Heeft u in de afgelopen 3 maanden afspraken gehad met de bedrijfsarts?	◯ Nee<br>◯ Ja
12.9.1	***If 'Heeft u in de afgelopen 3 maanden afspraken gehad met de bedrijfsarts?' is equal to 'Ja' answer this question:***<br>Hoeveel afspraken had u?	[ ] afspraken
12.10	Heeft u in de afgelopen 3 maanden hulp van de thuiszorg gehad?	◯ Ja<br>◯ Nee
12.10.1	***If 'Heeft u in de afgelopen 3 maanden hulp van de thuiszorg gehad?' is equal to 'Ja' answer this question:***<br>Wat voor hulp van de thuiszorg heeft u gehad in de afgelopen 3 maanden?	☐ Huishoudelijke hulp; voorbeeld: stofzuigen, bed opmaken, boodschappen doen<br>☐ Verzorging van uzelf; voorbeeld: hulp bij douchen of aankleden<br>☐ Verpleging; voorbeeld: verband omdoen, medicijnen geven, bloeddruk meten
12.10.1.1	***If 'Wat voor hulp van de thuiszorg heeft u gehad in de afgelopen 3 maanden?' is equal to 'Huishoudelijke hulp; voorbeeld: stofzuigen, bed opmaken, boodschappen doen' answer this question:***<br>Hoeveel weken heeft u deze thuiszorg gehad? Huishoudelijke hulp:	[ ] weken in de afgelopen 3 maanden
12.10.1.2	***If 'Wat voor hulp van de thuiszorg heeft u gehad in de afgelopen 3 maanden?' is equal to 'Verzorging van uzelf; voorbeeld: hulp bij douchen of aankleden' answer this question:***<br>Hoeveel weken heeft u deze thuiszorg gehad? Verzorging van uzelf:	[ ] weken in de afgelopen 3 maanden

12.10.1.3	***If 'Wat voor hulp van de thuiszorg heeft u gehad in de afgelopen 3 maanden?' is equal to 'Verpleging; voorbeeld: verband omdoen, medicijnen geven, bloeddruk meten' answer this question:***<br>Hoeveel weken heeft u deze thuiszorg gehad? Verpleging:	______ weken in de afgelopen 3 maanden
12.10.1.4	***If 'Wat voor hulp van de thuiszorg heeft u gehad in de afgelopen 3 maanden?' is equal to 'Huishoudelijke hulp; voorbeeld: stofzuigen, bed opmaken, boodschappen doen' answer this question:***<br>Hoeveel uur thuiszorg kreeg u in deze weken gemiddeld? Huishoudelijke hulp: gemiddeld	______ uur in de week
12.10.1.5	***If 'Wat voor hulp van de thuiszorg heeft u gehad in de afgelopen 3 maanden?' is equal to 'Verzorging van uzelf; voorbeeld: hulp bij douchen of aankleden' answer this question:***<br>Hoeveel uur thuiszorg kreeg u in deze weken gemiddeld? Verzorging van uzelf: gemiddeld	______ uur in de week
12.10.1.6	***If 'Wat voor hulp van de thuiszorg heeft u gehad in de afgelopen 3 maanden?' is equal to 'Verpleging; voorbeeld: verband omdoen, medicijnen geven, bloeddruk meten' answer this question:***<br>Hoeveel uur thuiszorg kreeg u in deze weken gemiddeld? Verpleging: gemiddeld	______ uur in de week
12.11	Heeft u in de afgelopen 3 maanden medicijnen gebruikt?	○ Ja<br>○ Nee
12.11.1	***If 'Heeft u in de afgelopen 3 maanden medicijnen gebruikt?' is equal to 'Ja' answer this question:***<br>Welke medicijnen heeft u in de afgelopen 3 maanden gebruikt?	______
12.12	Bent u in de afgelopen 3 maanden op de spoedeisende eerste hulp van een ziekenhuis geweest? (Een andere naam voor spoedeisende eerste hulp is EHBO)	○ Nee<br>○ Ja
12.12.1	***If 'Bent u in de afgelopen 3 maanden op de spoedeisende eerste hulp van een ziekenhuis geweest? (Een andere naam voor spoedeisende eerste hulp is EHBO)' is equal to 'Ja' answer this question:***<br>Hoevaak bent u geweest?	______ keer
12.13	Bent u in de afgelopen 3 maanden met een ambulance naar het ziekenhuis gebracht? (Een andere naam voor ambulance is ziekenauto)	○ Nee<br>○ Ja

12.13.1	***If 'Bent u in de afgelopen 3 maanden met een ambulance naar het ziekenhuis gebracht? (Een andere naam voor ambulance is ziekenauto)' is equal to 'Ja' answer this question:***<br>Hoe vaak bent u naar het ziekenhuis gebracht?	______ keer
12.14	Had u in de afgelopen 3 maanden een afspraak bij de polikliniek van het ziekenhuis? (Het gaat om afspraken voor uzelf met een dokter. Bijvoorbeeld met de cardioloog, reumatoloog of neuroloog)	◯ Ja<br>◯ Nee
12.14.1	***If 'Had u in de afgelopen 3 maanden een afspraak bij de polikliniek van het ziekenhuis? (Het gaat om afspraken voor uzelf met een dokter. Bijvoorbeeld met de cardioloog, reumatoloog of neuroloog)' is equal to 'Ja' answer this question:***<br>Bij welke soorten dokters bent u in de afgelopen 3 maanden in het ziekenhuis geweest? En hoe vaak?	
12.15	Bent u in de afgelopen 3 maanden overdag in het ziekenhuis geweest voor een behandeling? (U bleef dus niet slapen. U kwam bijvoorbeeld voor een bloedtransfusie, nierdialyse of chemokuur)	◯ Ja<br>◯ Nee
12.15.1	***If 'Bent u in de afgelopen 3 maanden overdag in het ziekenhuis geweest voor een behandeling? (U bleef dus niet slapen. U kwam bijvoorbeeld voor een bloedtransfusie, nierdialyse of chemokuur)' is equal to 'Ja' answer this question:***<br>Voor welke soort behandeling was dit?	
12.15.2	***If 'Bent u in de afgelopen 3 maanden overdag in het ziekenhuis geweest voor een behandeling? (U bleef dus niet slapen. U kwam bijvoorbeeld voor een bloedtransfusie, nierdialyse of chemokuur)' is equal to 'Ja' answer this question:***<br>Hoeveel keer moest u in de afgelopen 3 maanden voor deze behandelingen naar het ziekenhuis?	
12.16	Bent u in de afgelopen 3 maanden ergens anders geweest voor een behandeling overdag? (U bleef dus niet slapen. U ging bijvoorbeeld naar de dagopvang van een woon-/zorgcentrum of een psychiatrische instelling. Of naar de dagbehandeling van een revalidatiecentrum)	◯ Ja<br>◯ Nee
12.16.1	***If 'Bent u in de afgelopen 3 maanden ergens anders geweest voor een behandeling overdag? (U bleef dus niet slapen. U ging bijvoorbeeld naar de dagopvang van een woon-/zorgcentrum of een psychiatrische instelling. Of naar de dagbehandeling van een revalidatiecentrum)' is equal to 'Ja' answer this question:***<br>Wat voor instelling was dit?	☐ Woon-/zorgcentrum<br>☐ Revalidatiecentrum<br>☐ Psychiatrische instelling<br>☐ Een andere instelling, namelijk

12.16.1.1	***If 'Wat voor instelling was dit?' is equal to 'Een andere instelling, namelijk' answer this question:*** Wat voor instelling was dit?	
12.16.1.2	***If 'Wat voor instelling was dit?' is equal to 'Woon-/zorgcentrum' answer this question:*** Hoe vaak moest u hier in de afgelopen 3 maanden naartoe? Naar het woon-/zorgcentrum:	keer in de afgelopen 3 maanden
12.16.1.3	***If 'Wat voor instelling was dit?' is equal to 'Revalidatiecentrum' answer this question:*** Hoe vaak moest u hier in de afgelopen 3 maanden naartoe? Naar het revalidatiecentrum:	keer in de afgelopen 3 maanden
12.16.1.4	***If 'Wat voor instelling was dit?' is equal to 'Psychiatrische instelling' answer this question:*** Hoe vaak moest u hier in de afgelopen 3 maanden naartoe? Naar de psychiatrische instelling:	keer in de afgelopen 3 maanden
12.16.1.5	***If 'Wat voor instelling was dit?' is equal to 'Een andere instelling, namelijk' answer this question:*** Hoe vaak moest u hier in de afgelopen 3 maanden naartoe? Naar de andere instelling:	keer in de afgelopen 3 maanden
12.17	Heeft u in de afgelopen 3 maanden weleens in het ziekenhuis gelegen? (U moest dus blijven slapen. Bijvoorbeeld omdat u geopereerd was en niet meteen naar huis kon)	◯ Ja<br>◯ Nee
12.17.1	***If 'Heeft u in de afgelopen 3 maanden weleens in het ziekenhuis gelegen? (U moest dus blijven slapen. Bijvoorbeeld omdat u geopereerd was en niet meteen naar huis kon)' is equal to 'Ja' answer this question:*** Hoe vaak heeft u in de afgelopen 3 maanden in het ziekenhuis gelegen?	keer in de afgelopen 3 maanden
12.17.2	***If 'Heeft u in de afgelopen 3 maanden weleens in het ziekenhuis gelegen? (U moest dus blijven slapen. Bijvoorbeeld omdat u geopereerd was en niet meteen naar huis kon)' is equal to 'Ja' answer this question:*** Hoe lang heeft u in het ziekenhuis gelegen?	dagen in de afgelopen 3 maanden
12.18	Moest u in de afgelopen 3 maanden ergens anders blijven slapen voor uw gezondheid? (Bijvoorbeeld in een woon-/zorgcentrum, psychiatrische instelling of revalidatiecentrum)	◯ Ja<br>◯ Nee

12.18.1	***If 'Moest u in de afgelopen 3 maanden ergens anders blijven slapen voor uw gezondheid? (Bijvoorbeeld in een woon-/zorgcentrum, psychiatrische instelling of revalidatiecentrum)' is equal to 'Ja' answer this question:*** Wat voor instelling was dit?	☐ Woon-/zorgcentrum<br>☐ Revalidatiecentrum<br>☐ Psychiatrische instelling<br>☐ Een andere instelling, namelijk
12.18.1.1	***If 'Wat voor instelling was dit?' is equal to 'Een andere instelling, namelijk' answer this question:*** Een andere instelling, namelijk	
12.18.1.2	***If 'Wat voor instelling was dit?' is equal to 'Woon-/zorgcentrum' answer this question:*** Hoe lang bent u in deze instelling geweest? In het woon-/zorgcentrum:	____ dagen in de afgelopen 3 maanden
12.18.1.3	***If 'Wat voor instelling was dit?' is equal to 'Revalidatiecentrum' answer this question:*** Hoe lang bent u in deze instelling geweest? In het revalidatiecentrum:	____ dagen in de afgelopen 3 maanden
12.18.1.4	***If 'Wat voor instelling was dit?' is equal to 'Psychiatrische instelling' answer this question:*** Hoe lang bent u in deze instelling geweest? In de psychiatrische instelling:	____ dagen in de afgelopen 3 maanden
12.18.1.5	***If 'Wat voor instelling was dit?' is equal to 'Een andere instelling, namelijk' answer this question:*** Hoe lang bent u in deze instelling geweest? In de andere instelling:	____ dagen in de afgelopen 3 maanden
12.19	Heeft u in de afgelopen 3 maanden hulp gekregen van een familielid of een bekende vanwege uw lichamelijke of psychische problemen?	○ Ja<br>○ Nee
12.19.1	***If 'Heeft u in de afgelopen 3 maanden hulp gekregen van een familielid of een bekende vanwege uw lichamelijke of psychische problemen?' is equal to 'Ja' answer this question:*** Wat voor hulp van familieleden of bekenden heeft u gehad in de afgelopen 3 maanden?	☐ Huishoudelijke hulp - voorbeeld: stofzuigen, bed opmaken, boodschappen doen, klaarmaken van eten en drinken, verzorgen van kinderen<br>☐ Verzorging van uzelf - voorbeeld: hulp bij douchen of aankleden, hulp bij het eten en drinken of het geven van medicijnen<br>☐ Praktische hulp - voorbeeld: ondersteuning bij wandelen, het maken van uitstapjes of bezoekjes aan bekenden, bezoeken aan de huisarts of het ziekenhuis, het regelen van hulp of het regelen van financiële zaken

12.19.1.1	***If 'Wat voor hulp van familieleden of bekenden heeft u gehad in de afgelopen 3 maanden?' is equal to 'Huishoudelijke hulp - voorbeeld: stofzuigen, bed opmaken, boodschappen doen, klaarmaken van eten en drinken, verzorgen van kinderen' answer this question:*** Hoeveel weken heeft u deze hulp gehad? Huishoudelijke hulp:	______ weken in de afgelopen 3 maanden
12.19.1.2	***If 'Wat voor hulp van familieleden of bekenden heeft u gehad in de afgelopen 3 maanden?' is equal to 'Verzorging van uzelf - voorbeeld: hulp bij douchen of aankleden, hulp bij het eten en drinken of het geven van medicijnen' answer this question:*** Hoeveel weken heeft u deze hulp gehad? Verzorging van uzelf:	______ weken in de afgelopen 3 maanden
12.19.1.3	***If 'Wat voor hulp van familieleden of bekenden heeft u gehad in de afgelopen 3 maanden?' is equal to 'Praktische hulp - voorbeeld: ondersteuning bij wandelen, het maken van uitstapjes of bezoekjes aan bekenden, bezoeken aan de huisarts of het ziekenhuis, het regelen van hulp of het regelen van financiële zaken' answer this question:*** Hoeveel uur hulp kreeg u in deze weken gemiddeld? Praktische hulp:	______ weken in de afgelopen 3 maanden
12.19.1.4	***If 'Wat voor hulp van familieleden of bekenden heeft u gehad in de afgelopen 3 maanden?' is equal to 'Huishoudelijke hulp - voorbeeld: stofzuigen, bed opmaken, boodschappen doen, klaarmaken van eten en drinken, verzorgen van kinderen' answer this question:*** Hoeveel uur hulp kreeg u in deze weken gemiddeld? Huishoudelijke hulp: gemiddeld	______ uur in de week
12.19.1.5	***If 'Wat voor hulp van familieleden of bekenden heeft u gehad in de afgelopen 3 maanden?' is equal to 'Verzorging van uzelf - voorbeeld: hulp bij douchen of aankleden, hulp bij het eten en drinken of het geven van medicijnen' answer this question:*** Hoeveel uur hulp kreeg u in deze weken gemiddeld? Verzorging van uzelf: gemiddeld	______ uur in de week
12.19.1.6	***If 'Wat voor hulp van familieleden of bekenden heeft u gehad in de afgelopen 3 maanden?' is equal to 'Praktische hulp - voorbeeld: ondersteuning bij wandelen, het maken van uitstapjes of bezoekjes aan bekenden, bezoeken aan de huisarts of het ziekenhuis, het regelen van hulp of het regelen van financiële zaken' answer this question:*** Hoeveel uur hulp kreeg u in deze weken gemiddeld? Praktische hulp: gemiddeld	______ uur in de week

Vragenlijst PRIMA studie 12 weken - Dank

Number	Question	Answers
13.1	Hartelijk dank voor de tijd die u heeft genomen om de vragenlijsten in te vullen. Indien u nog opmerkingen heeft kunt u deze hierin plaatsen.	

Survey 'Vragenlijst PRIMA studie 26 weken'

Vragenlijst PRIMA studie 26 weken - AOFAS - Pijn

Number	Question	Answers
1.1	Hoeveel pijn ervaart u aan uw voet?	☐ Geen<br>☐ Milde pijnklachten of af en toe pijn<br>☐ Matige pijnklachten, dagelijks<br>☐ Vrijwel continu erge pijn

Vragenlijst PRIMA studie 26 weken - AOFAS resterende gedeelte

Number	Question	Answers
2.1	Hoeveel beperkingen in het dagelijks leven ervaart u?	○ Geen beperkingen, geen hulpmiddelen nodig<br>○ Geen beperkingen in algemeen dagelijkse bezigheden, wel beperkingen in recreatieve activiteiten (sport, hobbies, etc) geen ondersteuning<br>○ Beperkingen bij dagelijkse- en recreatieve activiteiten, stok<br>○ Ernstige beperkingen bij dagelijkse- en recreatieactiviteiten, walker, krukken, rolstoel, brace

2.2	Wat is de afstand die u in één keer kunt lopen?	◯ Meer dan 600 meter<br>◯ 400 - 600 meter<br>◯ 100 - 300 meter<br>◯ Minder dan 100 meter
2.3	Heeft u moeite met lopen op een bepaalde ondergrond?	◯ Geen problemen bij lopen<br>◯ Enig problemen op ongelijke ondergrond, trap, helling, ladders<br>◯ Ernstige problemen bij ongelijke ondergrond, trap, helling, ladders
2.4	Hoe zou u uw looppatroon beoordelen?	◯ Normaal of minimaal afwijkend<br>◯ Duidelijk<br>◯ Ernstig
2.5	Hoe zou u de beweeglijkheid van uw voet beoordelen ten opzicht van de andere zijde of ten opzicte van vóór het ongeval?	◯ Hetzelfde of licht beperkt t.o.v. de niet aangedane zijde of zoals voor het ongeval<br>◯ Ongeveer de helft beperkt<br>◯ Sterk verminderd, bijna geen bewegingen mogelijk
2.6	Hoe zou u de beweeglijkheid van uw enkel beoordelen ten opzichte van de andere zijde of ten opzichte van voor het ongeval?	◯ Hetzelfde of licht beperkt t.o.v. de niet aangedane zijde of zoals voor het ongeval<br>◯ Ongeveer de helft beperkt<br>◯ Sterk verminderd, bijna geen bewegingen mogelijk
2.7	Hoe zou u de stabiliteit van uw voet en enkel beoordelen	◯ Stabiel, ik verzwik mijn enkel hooguit incidenteel<br>◯ Niet stabiel, ik verzwik mijn enkel vaak
2.8	Hoe zou u de stand van uw voet beoordelen ten opzichte van de andere zijde of ten opzichte van voor het ongeval?	◯ Goed, ik kan mijn voet goed plat op de grond zetten, waarbij de stand niet afwijkend is<br>◯ Ik sta vrijwel alleen op de buitenzijde van mijn voet<br>◯ Ik kan mijn voet niet plat op de grond zetten en loop vrijwel alleen op mijn voorvoet

Vragenlijst PRIMA studie 26 weken - VAS Pain score

Number	Question	Answers	
3.1	VAS pijnscore (Geef uw minimaal pijnscore aan tijdens dagelijkse activiteiten op een schaal van 0 tot 100, waarbij 0 geen pijn is en 100 de ergste pijn denkbaar is)	(0.00)	100 (100.00)

Vragenlijst PRIMA studie 26 weken - AAS

Number	Question	Answers

4.1	Wat is het meest bij u van toepassing ten aanzien van uw acitviteit?	☐ American Football<br>☐ Basketbal<br>☐ Gymnastiek/turnen<br>☐ Handbal<br>☐ Rugby<br>☐ Voetbal<br>☐ Hockey<br>☐ Korfbal<br>☐ Gevechtssporten: judo, karate, kung fu, taekwando, jiu jitsu, etc.<br>☐ Oriëntatielopen<br>☐ Rhytmische gymnastiek<br>☐ Volleybal<br>☐ Boxen<br>☐ Freestyle snowboarden<br>☐ Ijshockey<br>☐ Tennis<br>☐ Worstelen<br>☐ Fitness, aerobics<br>☐ Badminton<br>☐ Baseball<br>☐ Cross-country hardlopen<br>☐ Moderne pentathlon<br>☐ Squash<br>☐ Surfen, windsurfen<br>☐ Tafel tennis<br>☐ Atletiek: spring-, werponderdelen<br>☐ Waterskieën<br>☐ Dans<br>☐ Schermen<br>☐ Zaalhockey<br>☐ Bergbeklimmen<br>☐ Langlauf<br>☐ Parachute springen<br>☐ Softball<br>☐ Speciale beroepen en werkactiviteiten; speciale beroepen: ballet, professioneel soldaat, speciale reddingswerker, stuntman, etc.<br>☐ Duiken<br>☐ Scubaduiken<br>☐ Skaten, in-line skaten<br>☐ Atletiek: looponderdelen<br>☐ Triatlon<br>☐ Gewichtsheffen, body-building

☐ Alle competitieve sporten hieronder met 'seasonal' conditioning
☐ Zwaar fysiek werk
☐ Alpine skiën en snowboarden
☐ Bowlen/curlen
☐ Golf
☐ Mountainbike/BMX
☐ Powerliften
☐ Zeilen
☐ Fysiek werk
☐ Wielrennen
☐ Paardrijden
☐ Motorsporten/technische sporten
☐ Roeien, kayakken
☐ Boogschieten
☐ Water polo en zwemmen
☐ Kunnen lopen op oneven grond
☐ Geen sport, echter geen limitatie in dagelijkse activiteiten
☐ Kunnen lopen op even grond, maar de dagelijkste activiteiten zijn gelimiteerd
☐ Niet kunnen lopen, fysieke handicap wegens enkelproblemen

4.1.1	***If 'Wat is het meest bij u van toepassing ten aanzien van uw acitviteit?' is equal to 'American Football' answer this question:***<br>American Football	○ Professioneel<br>○ Competitief<br>○ Recreatief
4.1.2	***If 'Wat is het meest bij u van toepassing ten aanzien van uw acitviteit?' is equal to 'Basketbal' answer this question:***<br>Basketbal	○ Professioneel<br>○ Competitief<br>○ Recreatief
4.1.3	***If 'Wat is het meest bij u van toepassing ten aanzien van uw acitviteit?' is equal to 'Gymnastiek/turnen' answer this question:***<br>Gymnastiek/turnen	○ Professioneel<br>○ Competitief<br>○ Recreatief
4.1.4	***If 'Wat is het meest bij u van toepassing ten aanzien van uw acitviteit?' is equal to 'Handbal' answer this question:***<br>Handbal	○ Professioneel<br>○ Competitief<br>○ Recreatief
4.1.5	***If 'Wat is het meest bij u van toepassing ten aanzien van uw acitviteit?' is equal to 'Rugby' answer this question:***<br>Rugby	○ Professioneel<br>○ Competitief<br>○ Recreatief

4.1.6	***If 'Wat is het meest bij u van toepassing ten aanzien van uw acitviteit?' is equal to 'Voetbal' answer this question:*** Voetbal	○ Professioneel ○ Competitief ○ Recreatief
4.1.7	***If 'Wat is het meest bij u van toepassing ten aanzien van uw acitviteit?' is equal to 'Hockey' answer this question:*** Hockey	○ Professioneel ○ Competitief ○ Recreatief
4.1.8	***If 'Wat is het meest bij u van toepassing ten aanzien van uw acitviteit?' is equal to 'Korfbal' answer this question:*** Korfbal	○ Professioneel ○ Competitief ○ Recreatief
4.1.9	***If 'Wat is het meest bij u van toepassing ten aanzien van uw acitviteit?' is equal to 'Gevechtssporten: judo, karate, kung fu, taekwando, jiu jitsu, etc.' answer this question:*** Gevechtssporten: judo, karate, kung fu, taekwando, jiu jitsu, etc.	○ Professioneel ○ Competitief ○ Recreatief
4.1.10	***If 'Wat is het meest bij u van toepassing ten aanzien van uw acitviteit?' is equal to 'Oriëntatielopen' answer this question:*** Oriëntatielopen	○ Professioneel ○ Competitief ○ Recreatief
4.1.11	***If 'Wat is het meest bij u van toepassing ten aanzien van uw acitviteit?' is equal to 'Rhytmische gymnastiek' answer this question:*** Rhytmisch gymnastiek	○ Professioneel ○ Competitief ○ Recreatief
4.1.12	***If 'Wat is het meest bij u van toepassing ten aanzien van uw acitviteit?' is equal to 'Volleybal' answer this question:*** Volleybal	○ Professioneel ○ Competitief ○ Recreatief
4.1.13	***If 'Wat is het meest bij u van toepassing ten aanzien van uw acitviteit?' is equal to 'Boxen' answer this question:*** Boxen	○ Professioneel ○ Competitief ○ Recreatief
4.1.14	***If 'Wat is het meest bij u van toepassing ten aanzien van uw acitviteit?' is equal to 'Freestyle snowboarden' answer this question:*** Freestyle snowboarden	○ Professioneel ○ Competitief ○ Recreatief
4.1.15	***If 'Wat is het meest bij u van toepassing ten aanzien van uw acitviteit?' is equal to 'Freestyle snowboarden' answer this question:*** IJshockey	○ Professioneel ○ Competitief ○ Recreatief

4.1.16	***If 'Wat is het meest bij u van toepassing ten aanzien van uw acitviteit?' is equal to 'Tennis' answer this question:*** Tennis	◯ Professioneel ◯ Competitief ◯ Recreatief
4.1.17	***If 'Wat is het meest bij u van toepassing ten aanzien van uw acitviteit?' is equal to 'Worstelen' answer this question:*** Worstelen	◯ Professioneel ◯ Competitief ◯ Recreatief
4.1.18	***If 'Wat is het meest bij u van toepassing ten aanzien van uw acitviteit?' is equal to 'Fitness, aerobics' answer this question:*** Fitness, aerobics	◯ Professioneel ◯ Competitief ◯ Recreatief
4.1.19	***If 'Wat is het meest bij u van toepassing ten aanzien van uw acitviteit?' is equal to 'Badminton' answer this question:*** Badminton	◯ Professioneel ◯ Competitief ◯ Recreatief
4.1.20	***If 'Wat is het meest bij u van toepassing ten aanzien van uw acitviteit?' is equal to 'Baseball' answer this question:*** Baseball	◯ Professioneel ◯ Competitief ◯ Recreatief
4.1.21	***If 'Wat is het meest bij u van toepassing ten aanzien van uw acitviteit?' is equal to 'Cross-country hardlopen' answer this question:*** Cross-country hardlopen	◯ Professioneel ◯ Competitief ◯ Recreatief
4.1.22	***If 'Wat is het meest bij u van toepassing ten aanzien van uw acitviteit?' is equal to 'Moderne pentathlon' answer this question:*** Moderne pentathlon	◯ Professioneel ◯ Competitief ◯ Recreatief
4.1.23	***If 'Wat is het meest bij u van toepassing ten aanzien van uw acitviteit?' is equal to 'Squash' answer this question:*** Squash	◯ Professioneel ◯ Competitief ◯ Recreatief
4.1.24	***If 'Wat is het meest bij u van toepassing ten aanzien van uw acitviteit?' is equal to 'Surfen, windsurfen' answer this question:*** Surfen, windsurfen	◯ Professioneel ◯ Competitief ◯ Recreatief
4.1.25	***If 'Wat is het meest bij u van toepassing ten aanzien van uw acitviteit?' is equal to 'Tafel tennis' answer this question:*** Tafel tennis	◯ Professioneel ◯ Competitief ◯ Recreatief

4.1.26	***If 'Wat is het meest bij u van toepassing ten aanzien van uw acitviteit?' is equal to 'Atletiek: spring-, werponderdelen' answer this question:***<br>Atletiek: spring-, werponderdelen	◯ Professioneel<br>◯ Competitief<br>◯ Recreatief
4.1.27	***If 'Wat is het meest bij u van toepassing ten aanzien van uw acitviteit?' is equal to 'Waterskieën' answer this question:***<br>Waterskien	◯ Professioneel<br>◯ Competitief<br>◯ Recreatief
4.1.28	***If 'Wat is het meest bij u van toepassing ten aanzien van uw acitviteit?' is equal to 'Dans' answer this question:***<br>Dans	◯ Professioneel<br>◯ Competitief<br>◯ Recreatief
4.1.29	***If 'Wat is het meest bij u van toepassing ten aanzien van uw acitviteit?' is equal to 'Schermen' answer this question:***<br>Schermen	◯ Professioneel<br>◯ Competitief<br>◯ Recreatief
4.1.30	***If 'Wat is het meest bij u van toepassing ten aanzien van uw acitviteit?' is equal to 'Zaalhockey' answer this question:***<br>Zaalhockey	◯ Professioneel<br>◯ Competitief<br>◯ Recreatief
4.1.31	***If 'Wat is het meest bij u van toepassing ten aanzien van uw acitviteit?' is equal to 'Bergbeklimmen' answer this question:***<br>Bergbeklimmen	◯ Professioneel<br>◯ Competitief<br>◯ Recreatief
4.1.32	***If 'Wat is het meest bij u van toepassing ten aanzien van uw acitviteit?' is equal to 'Langlauf' answer this question:***<br>Langlauf	◯ Professioneel<br>◯ Competitief<br>◯ Recreatief
4.1.33	***If 'Wat is het meest bij u van toepassing ten aanzien van uw acitviteit?' is equal to 'Parachute springen' answer this question:***<br>Parachute springen	◯ Professioneel<br>◯ Competitief<br>◯ Recreatief
4.1.34	***If 'Wat is het meest bij u van toepassing ten aanzien van uw acitviteit?' is equal to 'Softball' answer this question:***<br>Softball	◯ Professioneel<br>◯ Competitief<br>◯ Recreatief

4.1.35	***If 'Wat is het meest bij u van toepassing ten aanzien van uw acitviteit?' is equal to 'Speciale beroepen en werkactiviteiten; speciale beroepen: ballet, professioneel soldaat, speciale reddingswerker, stuntman, etc.' answer this question:***<br>Speciale beroepen en werkactiviteiten speciale beroepen: ballet, professioneel soldaat, speciale reddingswerker, stuntman, etc.	◯ ballet, professioneel soldaat, speciale reddingswerker, stuntman, etc.
4.1.36	***If 'Wat is het meest bij u van toepassing ten aanzien van uw acitviteit?' is equal to 'Duiken' answer this question:***<br>Duiken	◯ Professioneel/competitief<br>◯ Recreatief
4.1.37	***If 'Wat is het meest bij u van toepassing ten aanzien van uw acitviteit?' is equal to 'Scubaduiken' answer this question:***<br>Scubaduiken	◯ Professioneel/competitief<br>◯ Recreatief
4.1.38	***If 'Wat is het meest bij u van toepassing ten aanzien van uw acitviteit?' is equal to 'Skaten, in-line skaten' answer this question:***<br>skaten, in-linen skaten	◯ Professioneel/competitief<br>◯ Recreatief
4.1.39	***If 'Wat is het meest bij u van toepassing ten aanzien van uw acitviteit?' is equal to 'Atletiek: looponderdelen' answer this question:***<br>Atletiek: looponderdelen	◯ Professioneel/competitief<br>◯ Recreatief
4.1.40	***If 'Wat is het meest bij u van toepassing ten aanzien van uw acitviteit?' is equal to 'Triatlon' answer this question:***<br>triatlon	◯ Professioneel/competitief<br>◯ Recreatief
4.1.41	***If 'Wat is het meest bij u van toepassing ten aanzien van uw acitviteit?' is equal to 'Gewichtsheffen, body-building' answer this question:***<br>Gewichtsheffen, body-building	◯ Professioneel/competitief<br>◯ Recreatief
4.1.42	***If 'Wat is het meest bij u van toepassing ten aanzien van uw acitviteit?' is equal to 'Alle competitieve sporten hieronder met 'seasonal' conditioning' answer this question:***<br>Alle competitieve sporten hieronder met 'seasonal' conditioning	◯ Alle competitieve sporten hieronder met 'seasonal' conditioning
4.1.43	***If 'Wat is het meest bij u van toepassing ten aanzien van uw acitviteit?' is equal to 'Zwaar fysiek werk' answer this question:***<br>Zwaar fysiek werk	◯ Alle competitieve sporten hieronder met 'seasonal' conditioning

4.1.44	***If 'Wat is het meest bij u van toepassing ten aanzien van uw acitviteit?' is equal to 'Alpine skiën en snowboarden' answer this question:*** Alpine skien en snowboarden	○ Alpine skien en snowboarden
4.1.45	***If 'Wat is het meest bij u van toepassing ten aanzien van uw acitviteit?' is equal to 'Bowlen/curlen' answer this question:*** Bowlen/curlen	○ Bowlen/curlen
4.1.46	***If 'Wat is het meest bij u van toepassing ten aanzien van uw acitviteit?' is equal to 'Golf' answer this question:*** Golf	○ Golf
4.1.47	***If 'Wat is het meest bij u van toepassing ten aanzien van uw acitviteit?' is equal to 'Mountainbike/BMX' answer this question:*** Mountainbike/BMX	○ Mountainbike/BMX
4.1.48	***If 'Wat is het meest bij u van toepassing ten aanzien van uw acitviteit?' is equal to 'Powerliften' answer this question:*** Powerliften	○ Powerliften
4.1.49	***If 'Wat is het meest bij u van toepassing ten aanzien van uw acitviteit?' is equal to 'Zeilen' answer this question:*** Zeilen	○ Zeilen
4.1.50	***If 'Wat is het meest bij u van toepassing ten aanzien van uw acitviteit?' is equal to 'Fysiek werk' answer this question:*** Fysiek werk	○ Fysiek werk
4.1.51	***If 'Wat is het meest bij u van toepassing ten aanzien van uw acitviteit?' is equal to 'Wielrennen' answer this question:*** Wielrennen	○ Wielrennen
4.1.52	***If 'Wat is het meest bij u van toepassing ten aanzien van uw acitviteit?' is equal to 'Paardrijden' answer this question:*** Paardrijden	○ Paardrijden
4.1.53	***If 'Wat is het meest bij u van toepassing ten aanzien van uw acitviteit?' is equal to 'Motorsporten/technische sporten' answer this question:*** Motorsporten/technische sporten	○ Motorsporten/technische sporten
4.1.54	***If 'Wat is het meest bij u van toepassing ten aanzien van uw acitviteit?' is equal to 'Roeien, kayakken' answer this question:*** Roeien, kayakken	○ Roeien, kayakken

4.1.55	***If 'Wat is het meest bij u van toepassing ten aanzien van uw acitviteit?' is equal to 'Boogschieten' answer this question:*** Boogschieten	◯ Boogschieten
4.1.56	***If 'Wat is het meest bij u van toepassing ten aanzien van uw acitviteit?' is equal to 'Water polo en zwemmen' answer this question:*** Water polo en zwemmen	◯ Water polo en zwemmen
4.1.57	***If 'Wat is het meest bij u van toepassing ten aanzien van uw acitviteit?' is equal to 'Kunnen lopen op oneven grond' answer this question:*** Kunnen lopen op oneven grond	◯ Kunnen lopen op oneven grond
4.1.58	***If 'Wat is het meest bij u van toepassing ten aanzien van uw acitviteit?' is equal to 'Geen sport, echter geen limitatie in dagelijkse activiteiten' answer this question:*** Geen sport, echter geen limitatie in dagelijkse activiteiten	◯ Geen sport, echter geen limitatie in dagelijkse activiteiten
4.1.59	***If 'Wat is het meest bij u van toepassing ten aanzien van uw acitviteit?' is equal to 'Kunnen lopen op even grond, maar de dagelijkste activiteiten zijn gelimiteerd' answer this question:*** Kunnen lopen op even grond, maar de dagelijkste activiteiten zijn gelimiteerd	◯ Kunnen lopen op even grond, maar de dagelijkste activiteiten zijn gelimiteerd
4.1.60	***If 'Wat is het meest bij u van toepassing ten aanzien van uw acitviteit?' is equal to 'Niet kunnen lopen, fysieke handicap wegens enkelproblemen' answer this question:*** Niet kunnen lopen, fysieke handicap wegens enkelproblemen	◯ Niet kunnen lopen, fysieke handicap wegens enkelproblemen

Vragenlijst PRIMA studie 26 weken - Hoe tevreden bent u over de enkelklachten?

Number	Question	Answers
5.1	Hoe tevreden bent u over de enkelklachten?	◯ Slecht<br>◯ Matig<br>◯ Goed<br>◯ Uitstekend

Vragenlijst PRIMA studie 26 weken - SF-36

Number	Question	Answers
	Deze vragenlijst gaat over uw standpunten t.a.v. uw gezondheid. Met behulp van deze gegevens kan worden bijgehouden hoe u zich voelt en hoe goed u in staat bent uw gebruikelijke bezigheden uit te voeren. Beantwoord elke vraag door een antwoord aan te klikken. Als u niet zeker weet hoe u een vraag moet beantwoorden, geef dan het best mogelijke antwoord.	
6.1	Hoe zou u over het algemeen uw gezondheid noemen?	◯ Uitstekend ◯ Zeer goed ◯ Goed ◯ Matig ◯ Slecht
6.2	Hoe beoordeelt u nu uw gezondheid over het algemeen vergeleken met een jaar geleden?	◯ Veel beter dan een jaar geleden ◯ Wat beter dan een jaar geleden ◯ Ongeveer hetzelfde als een jaar geleden ◯ Wat slechter dan een jaar geleden ◯ Veel slechter dan een jaar geleden
6.3	Wordt u door uw gezondheid op dit moment beperkt bij forse inspanning, zoals hardlopen, tillen van zware voorwerpen of een veeleisende sport beoefenen?	◯ Ja, ernstig beperkt ◯ Ja, een beetje beperkt ◯ Nee, helemaal niet beperkt
6.4	Wordt u door uw gezondheid op dit moment beperkt bij matige inspanning zoals een tafel verplaatsen, stofzuigen, zwemmen of fietsen?	◯ Ja, ernstig beperkt ◯ Ja, een beetje beperkt ◯ Nee, helemaal niet beperkt
6.5	Wordt u door uw gezondheid op dit moment beperkt bij boodschappen tillen of dragen?	◯ Ja, ernstig beperkt ◯ Ja, een beetje beperkt ◯ Nee, helemaal niet beperkt
6.6	Wordt u door uw gezondheid op dit moment beperkt bij een paar trappen oplopen?	◯ Ja, ernstig beperkt ◯ Ja, een beetje beperkt ◯ Nee, helemaal niet beperkt
6.7	Wordt u door uw gezondheid op dit moment beperkt bij één trap oplopen?	◯ Ja, ernstig beperkt ◯ Ja, een beetje beperkt ◯ Nee, helemaal niet beperkt
6.8	Wordt u door uw gezondheid op dit moment beperkt bij bukken, knielen of hurken?	◯ Ja, ernstig beperkt ◯ Ja, een beetje beperkt ◯ Nee, helemaal niet beperkt
6.9	Wordt u door uw gezondheid op dit moment beperkt bij meer dan één kilometer lopen?	◯ Ja, ernstig beperkt ◯ Ja, een beetje beperkt ◯ Nee, helemaal niet beperkt
6.10	Wordt u door uw gezondheid op dit moment beperkt bij een paar honderd meter lopen?	◯ Ja, ernstig beperkt ◯ Ja, een beetje beperkt ◯ Nee, helemaal niet beperkt

6.11	Wordt u door uw gezondheid op dit moment beperkt bij ongeveer honderd meter lopen?	◯ Ja, ernstig beperkt ◯ Ja, een beetje beperkt ◯ Nee, helemaal niet beperkt
6.12	Wordt u door uw gezondheid op dit moment beperkt bij uzelf wassen of aankleden?	◯ Ja, ernstig beperkt ◯ Ja, een beetje beperkt ◯ Nee, helemaal niet beperkt
6.13	U besteedde in de afgelopen 4 weken minder tijd aan werk of andere bezigheden	◯ Ja ◯ Nee
6.14	U heeft in de afgelopen 4 weken minder bereikt dan u zou willen	◯ Ja ◯ Nee
6.15	U was in de afgelopen 4 weken beperkt in het soort werk of andere bezigheden.	◯ Ja ◯ Nee
6.16	U had de afgelopen 4 weken moeite om uw werk of andere bezigheden uit te voeren (het kostte u bijvoorbeeld extra inspanning).	◯ Ja ◯ Nee
6.17	U besteedde in de afgelopen 4 weken minder tijd aan werk of andere bezigheden ten gevolge van emotionele problemen	◯ Ja ◯ Nee
6.18	U heeft in de afgelopen 4 weken minder bereikt dan u zou willen ten gevolge van emotionele problemen.	◯ Ja ◯ Nee
6.19	U deed de afgelopen 4 weken uw werk of andere bezigheden niet zo zorgvuldig als gewoonlijk ten gevolge van emotionele problemen.	◯ Ja ◯ Nee
6.20	In hoeverre hebben uw lichamelijke gezondheid of emotionele problemen u gedurende de afgelopen 4 weken gehinderd in uw normale omgang met familie, vrienden of buren, of bij activiteiten in groepsverband?	◯ Helemaal niet ◯ Enigszins ◯ Nogal ◯ Veel ◯ Heel erg veel
6.21	Hoeveel lichamelijke pijn heeft u de afgelopen 4 weken gehad?	◯ Geen<br>◯ Heel licht<br>◯ Licht<br>◯ Nogal<br>◯ Ernstig<br>◯ Heel ernstig
6.22	In welke mate bent u de afgelopen 4 weken door de pijn gehinderd in uw normale werk (zowel werk buitenshuis als huishoudelijk werk)?	◯ Helemaal niet<br>◯ Een klein beetje<br>◯ Nogal<br>◯ Veel<br>◯ Heel erg veel

6.23	Voelde u zich levenslustig?	◯ Altijd<br>◯ Meestal<br>◯ Vaak<br>◯ Soms<br>◯ Zelden<br>◯ Nooit
6.24	Was u erg zenuwachtig?	◯ Altijd<br>◯ Meestal<br>◯ Vaak<br>◯ Soms<br>◯ Zelden<br>◯ Nooit
6.25	Zat u zo in de put dat u niets kon?	◯ Altijd<br>◯ Meestal<br>◯ Vaak<br>◯ Soms<br>◯ Zelden<br>◯ Nooit
6.26	Voelde u zich rustig en tevreden?	◯ Altijd<br>◯ Meestal<br>◯ Vaak<br>◯ Soms<br>◯ Zelden<br>◯ Nooit
6.27	Had u veel energie?	◯ Altijd<br>◯ Meestal<br>◯ Vaak<br>◯ Soms<br>◯ Zelden<br>◯ Nooit
6.28	Voelde u zich somber en neerslachtig?	◯ Altijd<br>◯ Meestal<br>◯ Vaak<br>◯ Soms<br>◯ Zelden<br>◯ Nooit

6.29	Voelde u zich uitgeput?	○ Altijd<br>○ Meestal<br>○ Vaak<br>○ Soms<br>○ Zelden<br>○ Nooit
6.30	Was u een gelukkig mens?	○ Altijd<br>○ Meestal<br>○ Vaak<br>○ Soms<br>○ Zelden<br>○ Nooit
6.31	Voelde u zich moe?	○ Altijd<br>○ Meestal<br>○ Vaak<br>○ Soms<br>○ Zelden<br>○ Nooit
6.32	Hoe vaak hebben uw lichamelijke gezondheid of emotionele problemen u gedurende de afgelopen 4 weken gehinderd bij uw sociale activiteiten (zoals vrienden of familie bezoeken etc.)?	○ Altijd<br>○ Meestal<br>○ Soms<br>○ Zelden<br>○ Nooit
6.33	Ik lijk wat gemakkelijker ziek te worden dan andere mensen	○ Volkomen juist<br>○ Grotendeels juist<br>○ Weet ik niet<br>○ Grotendeels onjuist<br>○ Volkomen onjuist
6.34	Ik ben even gezond als andere mensen die ik ken	○ Volkomen juist<br>○ Grotendeels juist<br>○ Weet ik niet<br>○ Grotendeels onjuist<br>○ Volkomen onjuist

6.35	Ik verwacht dat mijn gezondheid achteruit zal gaan	◯ Volkomen juist<br>◯ Grotendeels juist<br>◯ Weet ik niet<br>◯ Grotendeels onjuist<br>◯ Volkomen onjuist
6.36	Mijn gezondheid is uitstekend	◯ Volkomen juist<br>◯ Grotendeels juist<br>◯ Weet ik niet<br>◯ Grotendeels onjuist<br>◯ Volkomen onjuist

Vragenlijst PRIMA studie 26 weken - GAS

Number	Question	Answers
	Tijdens de eerste afspraak werd een duidelijk doel met u afgesproken. Dit werd tevens per email naar u gestuurd. Hoe is de situatie nu ten aanzien van de destijds gestelde doelstelling?	
7.1	Wat is nu de situatie ten aanzien van het tijdens de eerste afspraak afgesproken doelstelling (Goal attainment Scaling)?	◯ Achteruitgang (minder dan de utigangssituatie)<br>◯ Uitgangssituatie<br>◯ Minder dan het doel<br>◯ Doel<br>◯ Meer dan het doel<br>◯ Veel meer dan het doel

Vragenlijst PRIMA studie 26 weken - EQ-5D-3L

Number	Question	Answers
	Deze vragenlijst gaat over uw standpunten t.a.v. uw gezondheid. Met behulp van deze gegevens kan worden bijgehouden hoe u zich voelt en hoe goed u in staat bent uw gebruikelijke bezigheden uit te voeren. Beantwoord elke vraag door een antwoord aan te klikken. Als u niet zeker weet hoe u een vraag moet beantwoorden, geef dan het best mogelijke antwoord.	
8.1	Hoe is het met uw Mobiliteit gesteld?	◯ Ik heb geen problemen met lopen<br>◯ Ik heb enige problemen met lopen<br>◯ Ik ben bedlegerig

8.2	Hoe is het met uw Zelfzorg gesteld?	◯ Ik heb geen problemen om mijzelf te wassen of aan te kleden<br>◯ Ik heb enige problemen om mijzelf te wassen of aan te kleden<br>◯ Ik ben niet in staat om mijzelf te wassen of aan te kleden
8.3	Hoe is het met de Dagelijkse activiteiten (werk, studie, huishouden, gezins- en vrijetijdsactiviteiten) gesteld?	◯ Ik heb geen problemen met mijn dagelijkse activiteiten<br>◯ Ik heb enige problemen met mijn dagelijkse activiteiten<br>◯ Ik ben niet in staat om mijn dagelijkse activiteiten uit te voeren
8.4	Hoe is het met de Pijn/klachten gesteld?	◯ Ik heb geen pijn of andere klachten<br>◯ Ik heb matige pijn of andere klachten<br>◯ Ik heb zeer ernstige pijn of andere klachten
8.5	Hoe is het met de Stemming gesteld?	◯ Ik ben niet angstig of somber<br>◯ Ik ben matig angstig of somber<br>◯ Ik ben erg angstig of somber
	We willen weten hoe goed of slecht uw gezondheid VANDAAG is. Deze meetschaal loopt van 0 tot 100: 100 staat voor de BESTE gezondheid die u zich kunt voorstellen - 0 staat voor de SLECHTSTE gezondheid die u zich kunt voorstellen	
8.6	Uw Gezondheid vandaag	(0.00) (100.00)

Vragenlijst PRIMA studie 26 weken - AOS

Number	Question	Answers
	Instructies: De lijn naast elke vraag staat voor hoeveel PIJN u heeft in verschillende situaties. De linker kant (0) is “geen pijn” en de rechter kant (100) is “ergste pijn denkbaar”. Geef voor de onderstaande situaties op de lijn aan hoeveel PIJN u in de afgelopen week in de enkel had. Als een of meerdere van deze situaties niet van toepassing waren, dan kiest u de "niet van toepassing" optie.	
9.1	Wat was de hoogte van de ergste pijn in de afgelopen week?	(0.00) (100.00)
9.2	Heeft u pijn als u 's ochtends opstaat?	◯ Ja<br>◯ Nee<br>◯ Niet van toepassing

9.2.1	***If 'Heeft u pijn als u 's ochtends opstaat?' is equal to 'Ja' answer this question:***<br>Hoeveel pijn heeft u voordat u 's ochtends opstaat?	(0.00)	(100.00)
9.3	Heeft u pijn wanneer u op blote voeten loopt?	◯ Ja<br>◯ Nee<br>◯ Niet van toepassing	
9.3.1	***If 'Heeft u pijn wanneer u op blote voeten loopt?' is equal to 'Ja' answer this question:***<br>Hoeveel pijn heeft u als u op blote voeten loopt?	(0.00)	(100.00)
9.4	Heeft u pijn wanneer u op blote voeten staat?	◯ Ja<br>◯ Nee<br>◯ Niet van toepassing	
9.4.1	***If 'Heeft u pijn wanneer u op blote voeten staat?' is equal to 'Ja' answer this question:***<br>Hoeveel pijn heeft u als u op blote voeten staat?	(0.00)	(100.00)
9.5	Heeft u pijn wanneer u met schoenen loopt?	◯ Ja<br>◯ Nee<br>◯ Niet van toepassing	
9.5.1	***If 'Heeft u pijn wanneer u met schoenen loopt?' is equal to 'Ja' answer this question:***<br>Hoeveel pijn heeft u wanneer u met schoenen loopt?	(0.00)	(100.00)
9.6	Heeft u pijn wanneer u met schoenen staat?	◯ Ja<br>◯ Nee<br>◯ Niet van toepassing	
9.6.1	***If 'Heeft u pijn wanneer u met schoenen staat?' is equal to 'Ja' answer this question:***<br>Hoeveel pijn heeft u wanneer u met schoenen staat?	(0.00)	(100.00)
9.7	Heeft u pijn wanneer u loopt met steunzolen of een brace?	◯ Ja<br>◯ Nee<br>◯ Niet van toepassing	
9.7.1	***If 'Heeft u pijn wanneer u loopt met steunzolen of een brace?' is equal to 'Ja' answer this question:***<br>Hoeveel pijn heeft u wanneer u loopt met steunzolen of een brace?	(0.00)	(100.00)

9.8	Heeft u pijn wanneer u staat met steunzolen of een brace?	◯ Ja<br>◯ Nee<br>◯ Niet van toepassing	
9.8.1	***If 'Heeft u pijn wanneer u staat met steunzolen of een brace?' is equal to 'Ja' answer this question:***<br>Hoeveel pijn heeft u wanneer u staat met steunzolen of een brace?	(0.00)	(100.00)
9.9	Heeft u pijn aan het einde van de dag?	◯ Ja<br>◯ Nee<br>◯ Niet van toepassing	
9.9.1	***If 'Heeft u pijn aan het einde van de dag?' is equal to 'Ja' answer this question:***<br>Hoeveel pijn heeft u aan het einde van de dag?	(0.00)	(100.00)
	Instructies:De lijn naast elke vraag staat voor hoeveel MOEITE u heeft met verschillende activiteiten. De linker kant (0) is “Niet moeilijk” en de rechter kant (100) is “Te moeilijk, niet uitvoerbaar”. Geef voor de onderstaande activiteiten op de lijn aan hoeveel MOEITE u in de afgelopen week door enkelklachten had met onderstaande activiteiten. Als een situatie niet van toepassing is, kies dan de optie "niet van toepassing".		
9.10	Heeft u moeite met door het huis lopen?	◯ Ja<br>◯ Nee<br>◯ Niet van toepassing	
9.10.1	***If 'Heeft u moeite met door het huis lopen?' is equal to 'Ja' answer this question:***<br>Hoeveel moeite heeft u met door het huis lopen?	(0.00)	(100.00)
9.11	Heeft u moeite met buiten lopen op oneven ondergrond?	◯ Ja<br>◯ Nee<br>◯ Niet van toepassing	
9.11.1	***If 'Heeft u moeite met buiten lopen op oneven ondergrond?' is equal to 'Ja' answer this question:***<br>Hoeveel moeite heeft u met buiten lopen op oneven ondergrond?	(0.00)	(100.00)
9.12	Heeft u moeite met een paar honderd meter lopen?	◯ Ja<br>◯ Nee<br>◯ Niet van toepassing	
9.12.1	***If 'Heeft u moeite met een paar honderd meter lopen?' is equal to 'Ja' answer this question:***<br>Hoeveel moeite heeft u met een paar honderd meter lopen?	(0.00)	(100.00)

9.13	Heeft u moeite met een trap oplopen?	◯ Ja<br>◯ Nee<br>◯ Niet van toepassing	
9.13.1	***If 'Heeft u moeite met een trap oplopen?' is equal to 'Ja' answer this question:***<br>Hoeveel moeite heeft u met een trap oplopen?	(0.00)	(100.00)
9.14	Heeft u moeite met een trap aflopen?	◯ Ja<br>◯ Nee<br>◯ Niet van toepassing	
9.14.1	***If 'Heeft u moeite met een trap aflopen?' is equal to 'Ja' answer this question:***<br>Hoeveel moeite heeft u met een trap aflopen?	(0.00)	(100.00)
9.15	Heeft u moeite met op de tenen staan?	◯ Ja<br>◯ Nee<br>◯ Niet van toepassing	
9.15.1	***If 'Heeft u moeite met op de tenen staan?' is equal to 'Ja' answer this question:***<br>Hoeveel moeite heeft u met op de tenen staan?	(0.00)	(100.00)
9.16	Heeft u moeite met opstaan uit de stoel?	◯ Ja<br>◯ Nee<br>◯ Niet van toepassing	
9.16.1	***If 'Heeft u moeite met opstaan uit de stoel?' is equal to 'Ja' answer this question:***<br>Hoeveel moeite heeft u met opstaan uit de stoel?	(0.00)	(100.00)
9.17	Heeft u moeite met het op- of afstappen van stoepranden?	◯ Ja<br>◯ Nee<br>◯ Niet van toepassing	
9.17.1	***If 'Heeft u moeite met het op- of afstappen van stoepranden?' is equal to 'Ja' answer this question:***<br>Hoeveel moeite heeft u met het op- of afstappen van stoepranden?	(0.00)	(100.00)
9.18	Heeft u moeite met snel lopen of rennen?	◯ Ja<br>◯ Nee<br>◯ Niet van toepassing	

9.18.1	***If 'Heeft u moeite met snel lopen of rennen?' is equal to 'Ja' answer this question:***<br>Hoeveel moeite heeft u met snel lopen of rennen?	(0.00)	(100.00)

Vragenlijst PRIMA studie 26 weken - FAOS

Number	Question	Answers
	Deze lijst vraagt naar uw mening over uw voet/enkel. Uw antwoorden geven ons een beeld van uw voet/enkel klachten en hoe u in staat bent om alledaagse activiteiten uit te voeren in uw huidige situatie. Beantwoorden van een vraag doet u door het aanklikken van een vakje met het volgens u meest juiste antwoord (één vakje per vraag). Als u niet zeker weet hoe u een vraag moet beantwoorden, geeft u dan het antwoord dat volgens u het meest op uw situatie van toepassing is. Deze vraag heeft betrekking op het voorkomen van voet/enkel klachten in de afgelopen week.	
10.1	Is uw voet/enkel gezwollen?	○ Nooit ○ Zelden ○ Soms ○ Vaak ○ Altijd
10.2	Voelt u gekraak of hoort u klikken of een ander vreemd geluid wanneer u de voet/enkel beweegt?	○ Nooit ○ Zelden ○ Soms ○ Vaak ○ Altijd
10.3	Hapert uw enkel of blokkeert uw enkel ('op slot' gaan zitten) wanneer u deze beweegt?	○ Nooit ○ Zelden ○ Soms ○ Vaak ○ Altijd
10.4	Kunt u de voet/enkel volledig strekken?	○ Altijd ○ Vaak ○ Soms ○ Zelden ○ Nooit
10.5	Kunt u de voet volledig naar u toe buigen?	○ Altijd ○ Vaak ○ Soms ○ Zelden ○ Nooit
10.6	In welke mate heeft u een stijf gevoel in de voet/enkel 's ochtends bij het wakker worden?	○ Niet ○ Mild ○ Matig ○ Ernstig ○ Zeer ernstig
10.7	In welke mate heeft u een stijf gevoel in de voet/enkel na zitten, liggen of rusten later op de dag?	○ Niet ○ Mild ○ Matig ○ Ernstig ○ Zeer ernstig
10.8	Hoe vaak heeft u pijn in uw voet/enkel?	○ Nooit ○ Maandelijks ○ Wekelijks ○ Dagelijks ○ Altijd

10.9	Hoeveel voet/enkel pijn heeft u gehad in de afgelopen week bij draaien als uw voet/enkel op de grond staat?	◯ Geen<br>◯ Mild<br>◯ Matig<br>◯ Ernstig<br>◯ Zeer ernstig
10.10	Hoeveel voet/enkel pijn heeft u gehad in de afgelopen week bij het volledig uitstrekken van de voet/enkel?	◯ Geen<br>◯ Mild<br>◯ Matig<br>◯ Ernstig<br>◯ Zeer ernstig
10.11	Hoeveel voet/enkel pijn heeft u gehad in de afgelopen week bij het volledig naar u toe buigen/optrekken van de voet/enkel	◯ Geen<br>◯ Mild<br>◯ Matig<br>◯ Ernstig<br>◯ Zeer ernstig
10.12	Hoeveel voet/enkel pijn heeft u gehad in de afgelopen week bij het lopen op een vlakke ondergrond	◯ Geen<br>◯ Mild<br>◯ Matig<br>◯ Ernstig<br>◯ Zeer ernstig
10.13	Hoeveel voet/enkel pijn heeft u gehad in de afgelopen week bij het trap op- en trap af lopen?	◯ Geen<br>◯ Mild<br>◯ Matig<br>◯ Ernstig<br>◯ Zeer ernstig
10.14	Hoeveel voet/enkel pijn heeft u gehad in de afgelopen week 's nachts in bed?	◯ Geen<br>◯ Mild<br>◯ Matig<br>◯ Ernstig<br>◯ Zeer ernstig
10.15	Hoeveel voet/enkel pijn heeft u gehad in de afgelopen week bij het zitten of liggen?	◯ Geen<br>◯ Mild<br>◯ Matig<br>◯ Ernstig<br>◯ Zeer ernstig

10.16	Hoeveel voet/enkel pijn heeft u gehad in de afgelopen week bij het rechtop staan?	◯ Geen<br>◯ Mild<br>◯ Matig<br>◯ Ernstig<br>◯ Zeer ernstig
10.17	In welke mate werd u gehinderd bij het trap aflopen?	◯ Niet ◯ Mild ◯ Matig ◯ Ernstig ◯ Zeer ernstig
10.18	In welke mate werd u gehinderd bij het trap op lopen?	◯ Niet ◯ Mild ◯ Matig ◯ Ernstig ◯ Zeer ernstig
10.19	In welke mate werd u gehinderd als u vanuit een zittende positie ging staan?	◯ Niet ◯ Mild ◯ Matig ◯ Ernstig ◯ Zeer ernstig
10.20	In welke mate werd u gehinderd bij het staan?	◯ Niet ◯ Mild ◯ Matig ◯ Ernstig ◯ Zeer ernstig
10.21	In welke mate werd u gehinderd bij het naar de grond buigen/iets oprapen?	◯ Niet ◯ Mild ◯ Matig ◯ Ernstig ◯ Zeer ernstig
10.22	In welke mate werd u gehinderd bij het lopen op een vlakke ondergrond?	◯ Niet ◯ Mild ◯ Matig ◯ Ernstig ◯ Zeer ernstig
10.23	In welke mate werd u gehinderd bij het in- en uit de auto stappen?	◯ Niet ◯ Mild ◯ Matig ◯ Ernstig ◯ Zeer ernstig
10.24	In welke mate werd u gehinderd bij het boodschappen doen?	◯ Niet ◯ Mild ◯ Matig ◯ Ernstig ◯ Zeer ernstig
10.25	In welke mate werd u gehinderd bij sokken/panty's aantrekken?	◯ Niet ◯ Mild ◯ Matig ◯ Ernstig ◯ Zeer ernstig
10.26	In welke mate werd u gehinderd bij het opstaan uit bed?	◯ Niet ◯ Mild ◯ Matig ◯ Ernstig ◯ Zeer ernstig
10.27	In welke mate werd u gehinderd bij het sokken uittrekken?	◯ Niet ◯ Mild ◯ Matig ◯ Ernstig ◯ Zeer ernstig
10.28	In welke mate werd u gehinderd bij het in bed liggen (omdraaien, lange tijd uw voet/enkel in dezelfde positie houden)?	◯ Niet ◯ Mild ◯ Matig ◯ Ernstig ◯ Zeer ernstig

10.29	In welke mate werd u gehinderd bij het in/uit bad stappen?	◯ Niet ◯ Mild ◯ Matig ◯ Ernstig ◯ Zeer ernstig
10.30	In welke mate werd u gehinderd bij zitten?	◯ Niet ◯ Mild ◯ Matig ◯ Ernstig ◯ Zeer ernstig
10.31	In welke mate werd u gehinderd bij het toilet op en af gaan?	◯ Niet ◯ Mild ◯ Matig ◯ Ernstig ◯ Zeer ernstig
10.32	In welke mate werd u gehinderd bij zwaar huishoudelijk werk (bijvoorbeeld zware dozen sjouwen, vloer schrobben)?	◯ Niet ◯ Mild ◯ Matig ◯ Ernstig ◯ Zeer ernstig
10.33	In welke mate werd u gehinderd bij licht huishoudeijk werk (bijvoorbeeld koken, afstoffen)?	◯ Niet ◯ Mild ◯ Matig ◯ Ernstig ◯ Zeer ernstig
10.34	In welke mate werd u gehinderd bij hurken?	◯ Niet ◯ Mild ◯ Matig ◯ Ernstig ◯ Zeer ernstig
10.35	In welke mate werd u gehinderd bij hardlopen?	◯ Niet ◯ Mild ◯ Matig ◯ Ernstig ◯ Zeer ernstig
10.36	In welke mate werd u gehinderd bij springen?	◯ Niet ◯ Mild ◯ Matig ◯ Ernstig ◯ Zeer ernstig
10.37	In welke mate werd u gehinderd bij ronddraaien op uw aangedane voet/enkel?	◯ Niet ◯ Mild ◯ Matig ◯ Ernstig ◯ Zeer ernstig
10.38	In welke mate werd u gehinderd bij knielen?	◯ Niet ◯ Mild ◯ Matig ◯ Ernstig ◯ Zeer ernstig
10.39	Hoe vaak bent u zich bewust van uw voet/enkel probleem?	◯ Nooit ◯ Maandelijks ◯ Wekelijks ◯ Dagelijks ◯ Altijd
10.40	Heeft u uw leven veranderd om activiteiten te vermijden die schadelijk kunnen zijn voor uw voet/enkel?	◯ Niet<br>◯ Enigszins<br>◯ Matig<br>◯ Behoorlijk<br>◯ Volledig

10.41	In hoeverre kunt u op uw voet/enkel vertrouwen?	◯ Volledig<br>◯ Behoorlijk<br>◯ Matig<br>◯ Enigzins<br>◯ Niet
10.42	In het algemeen, in welke mate ondervindt u hinder van uw voet/enkel	◯ Geen<br>◯ Mild<br>◯ Matig<br>◯ Ernstig<br>◯ Zeer ernstig

Vragenlijst PRIMA studie 26 weken - PRODISQ - Gezondheid en werk

Number	Question	Answers
11.1	Wat is uw leeftijd?	[ ] jaar
11.2	Wat is uw geslacht?	◯ Man<br>◯ Vrouw
11.3	Wat is de hoogste opleiding die u heeft afgemaakt (Zoek uw hoogste opleiding en kruis het hokje daarvoor aan)?	☐ Ik heb geen school of opleiding afgemaakt<br>☐ Lagere school of basisschool<br>☐ Huishoudschool, vbo, lbo, lts, leao of lhno<br>☐ Mavo, mulo, ivo of vmbo<br>☐ Mbo, mts, meao, mhno, inas of intas<br>☐ Havo, vwo, hbs, mms, atheneum of gymnasium<br>☐ Hbo, hts, heao of hhno<br>☐ Universiteit<br>☐ Ik heb een andere opleiding afgemaakt, namelijk
11.3.1	***If 'Wat is de hoogste opleiding die u heeft afgemaakt (Zoek uw hoogste opleiding en kruis het hokje daarvoor aan)?' is equal to 'Ik heb een andere opleiding afgemaakt, namelijk' answer this question:***<br>Wat is de hoogste opleiding die u heeft afgemaakt?	[ ]

11.4	Wat doet u in het dagelijks leven?	☐ Ik zit op school, ik studeer<br>☐ Ik werk in loondienst<br>☐ Ik ben zelfstandig ondernemer<br>☐ Ik ben huisvrouw, huisman<br>☐ Ik ben werkloos<br>☐ Ik ben arbeidsongeschikt<br>☐ Ik ben met pensioen of prepensioen<br>☐ Ik doe iets anders, namelijk
11.4.1	***If 'Wat doet u in het dagelijks leven?' is equal to 'Ik ben arbeidsongeschikt' answer this question:***<br>Wat doet u in het dagelijks leven?	
11.4.2	***If 'Wat doet u in het dagelijks leven?' is equal to 'Ik doe iets anders, namelijk' answer this question:***<br>Wat doet u in het dagelijks leven?	
11.5	Hebt u betaald werk?	○ Nee<br>○ Ja
11.5.1	***If 'Hebt u betaald werk?' is equal to 'Ja' answer this question:***<br>Wat is uw beroep?	
11.5.2	***If 'Hebt u betaald werk?' is equal to 'Ja' answer this question:***<br>Hoeveel uur per week werkt u (Tel alleen de uren waarvoor u betaald wordt)?	uren
11.5.3	***If 'Hebt u betaald werk?' is equal to 'Ja' answer this question:***<br>Hoeveel dagen in de week werkt u?	Dagen
11.5.4	***If 'Hebt u betaald werk?' is equal to 'Ja' answer this question:***<br>Bent u in de afgelopen 4 weken afwezig geweest van uw werk omdat u ziek was?	○ Nee<br>○ Ja
11.5.4.1	***If 'Bent u in de afgelopen 4 weken afwezig geweest van uw werk omdat u ziek was?' is equal to 'Ja' answer this question:***<br>Bent u in de afgelopen 4 weken afwezig geweest van uw werk omdat u ziek was (Tel alleen de werkdagen in de afgelopen 4 weken)?	dagen afwezig geweest
11.5.4.2	***If 'Bent u in de afgelopen 4 weken afwezig geweest van uw werk omdat u ziek was?' is equal to 'Ja' answer this question:***<br>Was u langer dan de gehele periode van 4 weken afwezig van uw werk doordat u ziek was?	○ Nee<br>○ Ja

11.5.4.2.1	***If 'Was u langer dan de gehele periode van 4 weken afwezig van uw werk doordat u ziek was?' is equal to 'Ja' answer this question:*** Wanneer heeft u zich ziek gemeld?	[ ] [ ] [ ] *(dd-mm-yyyy)*
11.5.5	***If 'Hebt u betaald werk?' is equal to 'Ja' answer this question:*** Waren er in de afgelopen 4 weken, dagen waarop u wel gewerkt heeft, maar tijdens uw werk last had van lichamelijke of psychische problemen?	◯ Ja<br>◯ Nee
11.5.5.1	***If 'Waren er in de afgelopen 4 weken, dagen waarop u wel gewerkt heeft, maar tijdens uw werk last had van lichamelijke of psychische problemen?' is equal to 'Ja' answer this question:*** Op hoeveel werkdagen had u tijdens uw werk last van uw lichamelijke of psychische problemen?	[ ] werkdagen
11.5.5.2	***If 'Waren er in de afgelopen 4 weken, dagen waarop u wel gewerkt heeft, maar tijdens uw werk last had van lichamelijke of psychische problemen?' is equal to 'Ja' answer this question:*** Op de dagen dat u last had, kon u misschien niet zoveel werk doen als normaal. Hoeveel werk kon u op deze dagen gemiddeld doen?	Ik kon op deze dagen niks doen (0.00) — Ik kon net zoveel doen als normaal (10.00)
11.6	Waren er dagen waarop u minder onbetaald werk kon doen door uw lichamelijke of psychische problemen?	◯ Nee<br>◯ Ja
11.6.1	***If 'Waren er dagen waarop u minder onbetaald werk kon doen door uw lichamelijke of psychische problemen?' is equal to 'Ja' answer this question:*** Op hoeveel dagen was dit zo?	[ ] Dagen
11.6.2	***If 'Waren er dagen waarop u minder onbetaald werk kon doen door uw lichamelijke of psychische problemen?' is equal to 'Ja' answer this question:*** Stel dat iemand, bijvoorbeeld uw partner, familielid of een bekende, u op deze dagen had geholpen. En al het onbetaalde werk wat u niet kon doen, voor u had gedaan. Hoeveel uur was die persoon hier op deze dagen dan gemiddeld mee bezig geweest?	[ ] uur op deze dagen

Vragenlijst PRIMA studie 26 weken - PRODISQ - Zorggebruik

Number	Question	Answers
	Wij willen graag weten met welke dokters u in de afgelopen 3 maanden een afspraak had. Het gaat om afspraken voor uzelf. Ook andere zorgverleners tellen mee. Bijvoorbeeld de fysiotherapeut.	

12.1	Bent u in de afgelopen 3 maanden naar uw huisarts geweest?	◯ Nee<br>◯ Ja
12.1.1	***If 'Bent u in de afgelopen 3 maanden naar uw huisarts geweest?' is equal to 'Ja' answer this question:***<br>Hoeveel afspraken had u?	[ ] afspraken
12.2	Bent u in de afgelopen 3 maanden in contact geweest met een maatschappelijk werker?	◯ Nee<br>◯ Ja
12.2.1	***If 'Bent u in de afgelopen 3 maanden in contact geweest met een maatschappelijk werker?' is equal to 'Ja' answer this question:***<br>Hoeveel afspraken had u?	[ ] afspraken
12.3	Bent u in de afgelopen 3 maanden naar een fysiotherapeut geweest? Of een caesartherapeut, therapeut mensendieck of een manueel therapeut?	◯ Nee<br>◯ Ja
12.3.1	***If 'Bent u in de afgelopen 3 maanden naar een fysiotherapeut geweest? Of een caesartherapeut, therapeut mensendieck of een manueel therapeut?' is equal to 'Ja' answer this question:***<br>Hoeveel afspraken had u?	[ ] afspraken
12.4	Bent u in de afgelopen 3 maanden naar een ergotherapeut geweest?	◯ Nee<br>◯ Ja
12.4.1	***If 'Bent u in de afgelopen 3 maanden naar een ergotherapeut geweest?' is equal to 'Ja' answer this question:***<br>Hoeveel afspraken had u?	[ ] afspraken
12.5	Bent u in de afgelopen 3 maanden naar een logopedist geweest?	◯ Nee<br>◯ Ja
12.5.1	***If 'Bent u in de afgelopen 3 maanden naar een logopedist geweest?' is equal to 'Ja' answer this question:***<br>Hoeveel afspraken had u?	[ ] afspraken
12.6	Bent u in de afgelopen 3 maanden naar een diëtist geweest?	◯ Nee<br>◯ Ja
12.6.1	***If 'Bent u in de afgelopen 3 maanden naar een diëtist geweest?' is equal to 'Ja' answer this question:***<br>Hoeveel afspraken had u?	[ ] afspraken

12.7	Bent u in de afgelopen 3 maanden naar een homeopaat geweest? Of een acupuncturist?	◯ Nee<br>◯ Ja
12.7.1	***If 'Bent u in de afgelopen 3 maanden naar een homeopaat geweest? Of een acupuncturist?' is equal to 'Ja' answer this question:***<br>Hoeveel afspraken had u?	[ ] afspraken
12.8	Bent u in de afgelopen 3 maanden naar een psycholoog geweest? Of een psychotherapeut of psychiater?	◯ Nee<br>◯ Ja
12.8.1	***If 'Bent u in de afgelopen 3 maanden naar een psycholoog geweest? Of een psychotherapeut of psychiater?' is equal to 'Ja' answer this question:***<br>Hoeveel afspraken had u?	[ ] afspraken
12.9	Heeft u in de afgelopen 3 maanden afspraken gehad met de bedrijfsarts?	◯ Nee<br>◯ Ja
12.9.1	***If 'Heeft u in de afgelopen 3 maanden afspraken gehad met de bedrijfsarts?' is equal to 'Ja' answer this question:***<br>Hoeveel afspraken had u?	[ ] afspraken
12.10	Heeft u in de afgelopen 3 maanden hulp van de thuiszorg gehad?	◯ Ja<br>◯ Nee
12.10.1	***If 'Heeft u in de afgelopen 3 maanden hulp van de thuiszorg gehad?' is equal to 'Ja' answer this question:***<br>Wat voor hulp van de thuiszorg heeft u gehad in de afgelopen 3 maanden?	☐ Huishoudelijke hulp; voorbeeld: stofzuigen, bed opmaken, boodschappen doen<br>☐ Verzorging van uzelf; voorbeeld: hulp bij douchen of aankleden<br>☐ Verpleging; voorbeeld: verband omdoen, medicijnen geven, bloeddruk meten
12.10.1.1	***If 'Wat voor hulp van de thuiszorg heeft u gehad in de afgelopen 3 maanden?' is equal to 'Huishoudelijke hulp; voorbeeld: stofzuigen, bed opmaken, boodschappen doen' answer this question:***<br>Hoeveel weken heeft u deze thuiszorg gehad? Huishoudelijke hulp:	[ ] weken in de afgelopen 3 maanden
12.10.1.2	***If 'Wat voor hulp van de thuiszorg heeft u gehad in de afgelopen 3 maanden?' is equal to 'Verzorging van uzelf; voorbeeld: hulp bij douchen of aankleden' answer this question:***<br>Hoeveel weken heeft u deze thuiszorg gehad? Verzorging van uzelf:	[ ] weken in de afgelopen 3 maanden

12.10.1.3	***If 'Wat voor hulp van de thuiszorg heeft u gehad in de afgelopen 3 maanden?' is equal to 'Verpleging; voorbeeld: verband omdoen, medicijnen geven, bloeddruk meten' answer this question:*** Hoeveel weken heeft u deze thuiszorg gehad? Verpleging:	____ weken in de afgelopen 3 maanden
12.10.1.4	***If 'Wat voor hulp van de thuiszorg heeft u gehad in de afgelopen 3 maanden?' is equal to 'Huishoudelijke hulp; voorbeeld: stofzuigen, bed opmaken, boodschappen doen' answer this question:*** Hoeveel uur thuiszorg kreeg u in deze weken gemiddeld? Huishoudelijke hulp: gemiddeld	____ uur in de week
12.10.1.5	***If 'Wat voor hulp van de thuiszorg heeft u gehad in de afgelopen 3 maanden?' is equal to 'Verzorging van uzelf; voorbeeld: hulp bij douchen of aankleden' answer this question:*** Hoeveel uur thuiszorg kreeg u in deze weken gemiddeld? Verzorging van uzelf: gemiddeld	____ uur in de week
12.10.1.6	***If 'Wat voor hulp van de thuiszorg heeft u gehad in de afgelopen 3 maanden?' is equal to 'Verpleging; voorbeeld: verband omdoen, medicijnen geven, bloeddruk meten' answer this question:*** Hoeveel uur thuiszorg kreeg u in deze weken gemiddeld? Verpleging: gemiddeld	____ uur in de week
12.11	Heeft u in de afgelopen 3 maanden medicijnen gebruikt?	○ Ja<br>○ Nee
12.11.1	***If 'Heeft u in de afgelopen 3 maanden medicijnen gebruikt?' is equal to 'Ja' answer this question:*** Welke medicijnen heeft u in de afgelopen 3 maanden gebruikt?	____
12.12	Bent u in de afgelopen 3 maanden op de spoedeisende eerste hulp van een ziekenhuis geweest? (Een andere naam voor spoedeisende eerste hulp is EHBO)	○ Nee<br>○ Ja
12.12.1	***If 'Bent u in de afgelopen 3 maanden op de spoedeisende eerste hulp van een ziekenhuis geweest? (Een andere naam voor spoedeisende eerste hulp is EHBO)' is equal to 'Ja' answer this question:*** Hoevaak bent u geweest?	____ keer
12.13	Bent u in de afgelopen 3 maanden met een ambulance naar het ziekenhuis gebracht? (Een andere naam voor ambulance is ziekenauto)	○ Nee<br>○ Ja

12.13.1	***If 'Bent u in de afgelopen 3 maanden met een ambulance naar het ziekenhuis gebracht? (Een andere naam voor ambulance is ziekenauto)' is equal to 'Ja' answer this question:*** Hoe vaak bent u naar het ziekenhuis gebracht?	______ keer
12.14	Had u in de afgelopen 3 maanden een afspraak bij de polikliniek van het ziekenhuis? (Het gaat om afspraken voor uzelf met een dokter. Bijvoorbeeld met de cardioloog, reumatoloog of neuroloog)	◯ Ja<br>◯ Nee
12.14.1	***If 'Had u in de afgelopen 3 maanden een afspraak bij de polikliniek van het ziekenhuis? (Het gaat om afspraken voor uzelf met een dokter. Bijvoorbeeld met de cardioloog, reumatoloog of neuroloog)' is equal to 'Ja' answer this question:*** Bij welke soorten dokters bent u in de afgelopen 3 maanden in het ziekenhuis geweest? En hoe vaak?	
12.15	Bent u in de afgelopen 3 maanden overdag in het ziekenhuis geweest voor een behandeling? (U bleef dus niet slapen. U kwam bijvoorbeeld voor een bloedtransfusie, nierdialyse of chemokuur)	◯ Ja<br>◯ Nee
12.15.1	***If 'Bent u in de afgelopen 3 maanden overdag in het ziekenhuis geweest voor een behandeling? (U bleef dus niet slapen. U kwam bijvoorbeeld voor een bloedtransfusie, nierdialyse of chemokuur)' is equal to 'Ja' answer this question:*** Voor welke soort behandeling was dit?	
12.15.2	***If 'Bent u in de afgelopen 3 maanden overdag in het ziekenhuis geweest voor een behandeling? (U bleef dus niet slapen. U kwam bijvoorbeeld voor een bloedtransfusie, nierdialyse of chemokuur)' is equal to 'Ja' answer this question:*** Hoeveel keer moest u in de afgelopen 3 maanden voor deze behandelingen naar het ziekenhuis?	
12.16	Bent u in de afgelopen 3 maanden ergens anders geweest voor een behandeling overdag? (U bleef dus niet slapen. U ging bijvoorbeeld naar de dagopvang van een woon-/zorgcentrum of een psychiatrische instelling. Of naar de dagbehandeling van een revalidatiecentrum)	◯ Ja<br>◯ Nee
12.16.1	***If 'Bent u in de afgelopen 3 maanden ergens anders geweest voor een behandeling overdag? (U bleef dus niet slapen. U ging bijvoorbeeld naar de dagopvang van een woon-/zorgcentrum of een psychiatrische instelling. Of naar de dagbehandeling van een revalidatiecentrum)' is equal to 'Ja' answer this question:*** Wat voor instelling was dit?	☐ Woon-/zorgcentrum<br>☐ Revalidatiecentrum<br>☐ Psychiatrische instelling<br>☐ Een andere instelling, namelijk

12.16.1.1	***If 'Wat voor instelling was dit?' is equal to 'Een andere instelling, namelijk' answer this question:*** Wat voor instelling was dit?	
12.16.1.2	***If 'Wat voor instelling was dit?' is equal to 'Woon-/zorgcentrum' answer this question:*** Hoe vaak moest u hier in de afgelopen 3 maanden naartoe? Naar het woon-/zorgcentrum:	keer in de afgelopen 3 maanden
12.16.1.3	***If 'Wat voor instelling was dit?' is equal to 'Revalidatiecentrum' answer this question:*** Hoe vaak moest u hier in de afgelopen 3 maanden naartoe? Naar het revalidatiecentrum:	keer in de afgelopen 3 maanden
12.16.1.4	***If 'Wat voor instelling was dit?' is equal to 'Psychiatrische instelling' answer this question:*** Hoe vaak moest u hier in de afgelopen 3 maanden naartoe? Naar de psychiatrische instelling:	keer in de afgelopen 3 maanden
12.16.1.5	***If 'Wat voor instelling was dit?' is equal to 'Een andere instelling, namelijk' answer this question:*** Hoe vaak moest u hier in de afgelopen 3 maanden naartoe? Naar de andere instelling:	keer in de afgelopen 3 maanden
12.17	Heeft u in de afgelopen 3 maanden weleens in het ziekenhuis gelegen? (U moest dus blijven slapen. Bijvoorbeeld omdat u geopereerd was en niet meteen naar huis kon)	◯ Ja<br>◯ Nee
12.17.1	***If 'Heeft u in de afgelopen 3 maanden weleens in het ziekenhuis gelegen? (U moest dus blijven slapen. Bijvoorbeeld omdat u geopereerd was en niet meteen naar huis kon)' is equal to 'Ja' answer this question:*** Hoe vaak heeft u in de afgelopen 3 maanden in het ziekenhuis gelegen?	keer in de afgelopen 3 maanden
12.17.2	***If 'Heeft u in de afgelopen 3 maanden weleens in het ziekenhuis gelegen? (U moest dus blijven slapen. Bijvoorbeeld omdat u geopereerd was en niet meteen naar huis kon)' is equal to 'Ja' answer this question:*** Hoe lang heeft u in het ziekenhuis gelegen?	dagen in de afgelopen 3 maanden
12.18	Moest u in de afgelopen 3 maanden ergens anders blijven slapen voor uw gezondheid? (Bijvoorbeeld in een woon-/zorgcentrum, psychiatrische instelling of revalidatiecentrum)	◯ Ja<br>◯ Nee

12.18.1	***If 'Moest u in de afgelopen 3 maanden ergens anders blijven slapen voor uw gezondheid? (Bijvoorbeeld in een woon-/zorgcentrum, psychiatrische instelling of revalidatiecentrum)' is equal to 'Ja' answer this question:*** Wat voor instelling was dit?	☐ Woon-/zorgcentrum<br>☐ Revalidatiecentrum<br>☐ Psychiatrische instelling<br>☐ Een andere instelling, namelijk
12.18.1.1	***If 'Wat voor instelling was dit?' is equal to 'Een andere instelling, namelijk' answer this question:*** Een andere instelling, namelijk	
12.18.1.2	***If 'Wat voor instelling was dit?' is equal to 'Woon-/zorgcentrum' answer this question:*** Hoe lang bent u in deze instelling geweest? In het woon-/zorgcentrum:	______ dagen in de afgelopen 3 maanden
12.18.1.3	***If 'Wat voor instelling was dit?' is equal to 'Revalidatiecentrum' answer this question:*** Hoe lang bent u in deze instelling geweest? In het revalidatiecentrum:	______ dagen in de afgelopen 3 maanden
12.18.1.4	***If 'Wat voor instelling was dit?' is equal to 'Psychiatrische instelling' answer this question:*** Hoe lang bent u in deze instelling geweest? In de psychiatrische instelling:	______ dagen in de afgelopen 3 maanden
12.18.1.5	***If 'Wat voor instelling was dit?' is equal to 'Een andere instelling, namelijk' answer this question:*** Hoe lang bent u in deze instelling geweest? In de andere instelling:	______ dagen in de afgelopen 3 maanden
12.19	Heeft u in de afgelopen 3 maanden hulp gekregen van een familielid of een bekende vanwege uw lichamelijke of psychische problemen?	○ Ja<br>○ Nee
12.19.1	***If 'Heeft u in de afgelopen 3 maanden hulp gekregen van een familielid of een bekende vanwege uw lichamelijke of psychische problemen?' is equal to 'Ja' answer this question:*** Wat voor hulp van familieleden of bekenden heeft u gehad in de afgelopen 3 maanden?	☐ Huishoudelijke hulp - voorbeeld: stofzuigen, bed opmaken, boodschappen doen, klaarmaken van eten en drinken, verzorgen van kinderen<br>☐ Verzorging van uzelf - voorbeeld: hulp bij douchen of aankleden, hulp bij het eten en drinken of het geven van medicijnen<br>☐ Praktische hulp - voorbeeld: ondersteuning bij wandelen, het maken van uitstapjes of bezoekjes aan bekenden, bezoeken aan de huisarts of het ziekenhuis, het regelen van hulp of het regelen van financiële zaken

12.19.1.1	***If 'Wat voor hulp van familieleden of bekenden heeft u gehad in de afgelopen 3 maanden?' is equal to 'Huishoudelijke hulp - voorbeeld: stofzuigen, bed opmaken, boodschappen doen, klaarmaken van eten en drinken, verzorgen van kinderen' answer this question:*** Hoeveel weken heeft u deze hulp gehad? Huishoudelijke hulp:	______ weken in de afgelopen 3 maanden
12.19.1.2	***If 'Wat voor hulp van familieleden of bekenden heeft u gehad in de afgelopen 3 maanden?' is equal to 'Verzorging van uzelf - voorbeeld: hulp bij douchen of aankleden, hulp bij het eten en drinken of het geven van medicijnen' answer this question:*** Hoeveel weken heeft u deze hulp gehad? Verzorging van uzelf:	______ weken in de afgelopen 3 maanden
12.19.1.3	***If 'Wat voor hulp van familieleden of bekenden heeft u gehad in de afgelopen 3 maanden?' is equal to 'Praktische hulp - voorbeeld: ondersteuning bij wandelen, het maken van uitstapjes of bezoekjes aan bekenden, bezoeken aan de huisarts of het ziekenhuis, het regelen van hulp of het regelen van financiële zaken' answer this question:*** Hoeveel uur hulp kreeg u in deze weken gemiddeld? Praktische hulp:	______ weken in de afgelopen 3 maanden
12.19.1.4	***If 'Wat voor hulp van familieleden of bekenden heeft u gehad in de afgelopen 3 maanden?' is equal to 'Huishoudelijke hulp - voorbeeld: stofzuigen, bed opmaken, boodschappen doen, klaarmaken van eten en drinken, verzorgen van kinderen' answer this question:*** Hoeveel uur hulp kreeg u in deze weken gemiddeld? Huishoudelijke hulp: gemiddeld	______ uur in de week
12.19.1.5	***If 'Wat voor hulp van familieleden of bekenden heeft u gehad in de afgelopen 3 maanden?' is equal to 'Verzorging van uzelf - voorbeeld: hulp bij douchen of aankleden, hulp bij het eten en drinken of het geven van medicijnen' answer this question:*** Hoeveel uur hulp kreeg u in deze weken gemiddeld? Verzorging van uzelf: gemiddeld	______ uur in de week
12.19.1.6	***If 'Wat voor hulp van familieleden of bekenden heeft u gehad in de afgelopen 3 maanden?' is equal to 'Praktische hulp - voorbeeld: ondersteuning bij wandelen, het maken van uitstapjes of bezoekjes aan bekenden, bezoeken aan de huisarts of het ziekenhuis, het regelen van hulp of het regelen van financiële zaken' answer this question:*** Hoeveel uur hulp kreeg u in deze weken gemiddeld? Praktische hulp: gemiddeld	______ uur in de week

Vragenlijst PRIMA studie 26 weken - Dank

Number	Question	Answers
13.1	Hartelijk dank voor de tijd die u heeft genomen om de vragenlijsten in te vullen. Indien u nog opmerkingen heeft kunt u deze hierin plaatsen.	

Survey 'Vragenlijst PRIMA studie 39 weken'

Vragenlijst PRIMA studie 39 weken - PRODISQ - Gezondheid en werk

Number	Question	Answers
1.1	Wat is uw leeftijd?	jaar
1.2	Wat is uw geslacht?	◯ Man<br>◯ Vrouw
1.3	Wat is de hoogste opleiding die u heeft afgemaakt (Zoek uw hoogste opleiding en kruis het hokje daarvoor aan)?	☐ Ik heb geen school of opleiding afgemaakt<br>☐ Lagere school of basisschool<br>☐ Huishoudschool, vbo, lbo, lts, leao of lhno<br>☐ Mavo, mulo, ivo of vmbo<br>☐ Mbo, mts, meao, mhno, inas of intas<br>☐ Havo, vwo, hbs, mms, atheneum of gymnasium<br>☐ Hbo, hts, heao of hhno<br>☐ Universiteit<br>☐ Ik heb een andere opleiding afgemaakt, namelijk
1.3.1	***If 'Wat is de hoogste opleiding die u heeft afgemaakt (Zoek uw hoogste opleiding en kruis het hokje daarvoor aan)?' is equal to 'Ik heb een andere opleiding afgemaakt, namelijk' answer this question:***<br>Wat is de hoogste opleiding die u heeft afgemaakt?	

1.4	Wat doet u in het dagelijks leven?	☐ Ik zit op school, ik studeer<br>☐ Ik werk in loondienst<br>☐ Ik ben zelfstandig ondernemer<br>☐ Ik ben huisvrouw, huisman<br>☐ Ik ben werkloos<br>☐ Ik ben arbeidsongeschikt<br>☐ Ik ben met pensioen of prepensioen<br>☐ Ik doe iets anders, namelijk
1.4.1	***If 'Wat doet u in het dagelijks leven?' is equal to 'Ik ben arbeidsongeschikt' answer this question:***<br>Wat doet u in het dagelijks leven?	
1.4.2	***If 'Wat doet u in het dagelijks leven?' is equal to 'Ik doe iets anders, namelijk' answer this question:***<br>Wat doet u in het dagelijks leven?	
1.5	Hebt u betaald werk?	◯ Nee<br>◯ Ja
1.5.1	***If 'Hebt u betaald werk?' is equal to 'Ja' answer this question:***<br>Wat is uw beroep?	
1.5.2	***If 'Hebt u betaald werk?' is equal to 'Ja' answer this question:***<br>Hoeveel uur per week werkt u (Tel alleen de uren waarvoor u betaald wordt)?	uren
1.5.3	***If 'Hebt u betaald werk?' is equal to 'Ja' answer this question:***<br>Hoeveel dagen in de week werkt u?	Dagen
1.5.4	***If 'Hebt u betaald werk?' is equal to 'Ja' answer this question:***<br>Bent u in de afgelopen 4 weken afwezig geweest van uw werk omdat u ziek was?	◯ Nee<br>◯ Ja
1.5.4.1	***If 'Bent u in de afgelopen 4 weken afwezig geweest van uw werk omdat u ziek was?' is equal to 'Ja' answer this question:***<br>Bent u in de afgelopen 4 weken afwezig geweest van uw werk omdat u ziek was (Tel alleen de werkdagen in de afgelopen 4 weken)?	dagen afwezig geweest
1.5.4.2	***If 'Bent u in de afgelopen 4 weken afwezig geweest van uw werk omdat u ziek was?' is equal to 'Ja' answer this question:***<br>Was u langer dan de gehele periode van 4 weken afwezig van uw werk doordat u ziek was?	◯ Nee<br>◯ Ja

1.5.4.2.1	***If 'Was u langer dan de gehele periode van 4 weken afwezig van uw werk doordat u ziek was?' is equal to 'Ja' answer this question:***<br>Wanneer heeft u zich ziek gemeld?	☐ ☐ ☐ *(dd-mm-yyyy)*
1.5.5	***If 'Hebt u betaald werk?' is equal to 'Ja' answer this question:***<br>Waren er in de afgelopen 4 weken, dagen waarop u wel gewerkt heeft, maar tijdens uw werk last had van lichamelijke of psychische problemen?	○ Ja<br>○ Nee
1.5.5.1	***If 'Waren er in de afgelopen 4 weken, dagen waarop u wel gewerkt heeft, maar tijdens uw werk last had van lichamelijke of psychische problemen?' is equal to 'Ja' answer this question:***<br>Op hoeveel werkdagen had u tijdens uw werk last van uw lichamelijke of psychische problemen?	☐ werkdagen
1.5.5.2	***If 'Waren er in de afgelopen 4 weken, dagen waarop u wel gewerkt heeft, maar tijdens uw werk last had van lichamelijke of psychische problemen?' is equal to 'Ja' answer this question:***<br>Op de dagen dat u last had, kon u misschien niet zoveel werk doen als normaal. Hoeveel werk kon u op deze dagen gemiddeld doen?	Ik kon op deze dagen niks doen (0.00) — Ik kon net zoveel doen als normaal (10.00)
1.6	Waren er dagen waarop u minder onbetaald werk kon doen door uw lichamelijke of psychische problemen?	○ Nee<br>○ Ja
1.6.1	***If 'Waren er dagen waarop u minder onbetaald werk kon doen door uw lichamelijke of psychische problemen?' is equal to 'Ja' answer this question:***<br>Op hoeveel dagen was dit zo?	☐ Dagen
1.6.2	***If 'Waren er dagen waarop u minder onbetaald werk kon doen door uw lichamelijke of psychische problemen?' is equal to 'Ja' answer this question:***<br>Stel dat iemand, bijvoorbeeld uw partner, familielid of een bekende, u op deze dagen had geholpen. En al het onbetaalde werk wat u niet kon doen, voor u had gedaan. Hoeveel uur was die persoon hier op deze dagen dan gemiddeld mee bezig geweest?	☐ uur op deze dagen

Vragenlijst PRIMA studie 39 weken - PRODISQ - Zorggebruik

Number	Question	Answers
	Wij willen graag weten met welke dokters u in de afgelopen 3 maanden een afspraak had. Het gaat om afspraken voor uzelf. Ook andere zorgverleners tellen mee. Bijvoorbeeld de fysiotherapeut.	

2.1	Bent u in de afgelopen 3 maanden naar uw huisarts geweest?	◯ Nee<br>◯ Ja
2.1.1	***If 'Bent u in de afgelopen 3 maanden naar uw huisarts geweest?' is equal to 'Ja' answer this question:***<br>Hoeveel afspraken had u?	[ ] afspraken
2.2	Bent u in de afgelopen 3 maanden in contact geweest met een maatschappelijk werker?	◯ Nee<br>◯ Ja
2.2.1	***If 'Bent u in de afgelopen 3 maanden in contact geweest met een maatschappelijk werker?' is equal to 'Ja' answer this question:***<br>Hoeveel afspraken had u?	[ ] afspraken
2.3	Bent u in de afgelopen 3 maanden naar een fysiotherapeut geweest? Of een caesartherapeut, therapeut mensendieck of een manueel therapeut?	◯ Nee<br>◯ Ja
2.3.1	***If 'Bent u in de afgelopen 3 maanden naar een fysiotherapeut geweest? Of een caesartherapeut, therapeut mensendieck of een manueel therapeut?' is equal to 'Ja' answer this question:***<br>Hoeveel afspraken had u?	[ ] afspraken
2.4	Bent u in de afgelopen 3 maanden naar een ergotherapeut geweest?	◯ Nee<br>◯ Ja
2.4.1	***If 'Bent u in de afgelopen 3 maanden naar een ergotherapeut geweest?' is equal to 'Ja' answer this question:***<br>Hoeveel afspraken had u?	[ ] afspraken
2.5	Bent u in de afgelopen 3 maanden naar een logopedist geweest?	◯ Nee<br>◯ Ja
2.5.1	***If 'Bent u in de afgelopen 3 maanden naar een logopedist geweest?' is equal to 'Ja' answer this question:***<br>Hoeveel afspraken had u?	[ ] afspraken
2.6	Bent u in de afgelopen 3 maanden naar een diëtist geweest?	◯ Nee<br>◯ Ja
2.6.1	***If 'Bent u in de afgelopen 3 maanden naar een diëtist geweest?' is equal to 'Ja' answer this question:***<br>Hoeveel afspraken had u?	[ ] afspraken

For peer review only - http://bmjopen.bmj.com/site/about/guidelines.xhtml

2.7	Bent u in de afgelopen 3 maanden naar een homeopaat geweest? Of een acupuncturist?	○ Nee<br>○ Ja
2.7.1	***If 'Bent u in de afgelopen 3 maanden naar een homeopaat geweest? Of een acupuncturist?' is equal to 'Ja' answer this question:***<br>Hoeveel afspraken had u?	[ ] afspraken
2.8	Bent u in de afgelopen 3 maanden naar een psycholoog geweest? Of een psychotherapeut of psychiater?	○ Nee<br>○ Ja
2.8.1	***If 'Bent u in de afgelopen 3 maanden naar een psycholoog geweest? Of een psychotherapeut of psychiater?' is equal to 'Ja' answer this question:***<br>Hoeveel afspraken had u?	[ ] afspraken
2.9	Heeft u in de afgelopen 3 maanden afspraken gehad met de bedrijfsarts?	○ Nee<br>○ Ja
2.9.1	***If 'Heeft u in de afgelopen 3 maanden afspraken gehad met de bedrijfsarts?' is equal to 'Ja' answer this question:***<br>Hoeveel afspraken had u?	[ ] afspraken
2.10	Heeft u in de afgelopen 3 maanden hulp van de thuiszorg gehad?	○ Ja<br>○ Nee
2.10.1	***If 'Heeft u in de afgelopen 3 maanden hulp van de thuiszorg gehad?' is equal to 'Ja' answer this question:***<br>Wat voor hulp van de thuiszorg heeft u gehad in de afgelopen 3 maanden?	☐ Huishoudelijke hulp; voorbeeld: stofzuigen, bed opmaken, boodschappen doen<br>☐ Verzorging van uzelf; voorbeeld: hulp bij douchen of aankleden<br>☐ Verpleging; voorbeeld: verband omdoen, medicijnen geven, bloeddruk meten
2.10.1.1	***If 'Wat voor hulp van de thuiszorg heeft u gehad in de afgelopen 3 maanden?' is equal to 'Huishoudelijke hulp; voorbeeld: stofzuigen, bed opmaken, boodschappen doen' answer this question:***<br>Hoeveel weken heeft u deze thuiszorg gehad? Huishoudelijke hulp:	[ ] weken in de afgelopen 3 maanden
2.10.1.2	***If 'Wat voor hulp van de thuiszorg heeft u gehad in de afgelopen 3 maanden?' is equal to 'Verzorging van uzelf; voorbeeld: hulp bij douchen of aankleden' answer this question:***<br>Hoeveel weken heeft u deze thuiszorg gehad? Verzorging van uzelf:	[ ] weken in de afgelopen 3 maanden

2.10.1.3	***If 'Wat voor hulp van de thuiszorg heeft u gehad in de afgelopen 3 maanden?' is equal to 'Verpleging; voorbeeld: verband omdoen, medicijnen geven, bloeddruk meten' answer this question:*** Hoeveel weken heeft u deze thuiszorg gehad? Verpleging:	[ ] weken in de afgelopen 3 maanden
2.10.1.4	***If 'Wat voor hulp van de thuiszorg heeft u gehad in de afgelopen 3 maanden?' is equal to 'Huishoudelijke hulp; voorbeeld: stofzuigen, bed opmaken, boodschappen doen' answer this question:*** Hoeveel uur thuiszorg kreeg u in deze weken gemiddeld? Huishoudelijke hulp: gemiddeld	[ ] uur in de week
2.10.1.5	***If 'Wat voor hulp van de thuiszorg heeft u gehad in de afgelopen 3 maanden?' is equal to 'Verzorging van uzelf; voorbeeld: hulp bij douchen of aankleden' answer this question:*** Hoeveel uur thuiszorg kreeg u in deze weken gemiddeld? Verzorging van uzelf: gemiddeld	[ ] uur in de week
2.10.1.6	***If 'Wat voor hulp van de thuiszorg heeft u gehad in de afgelopen 3 maanden?' is equal to 'Verpleging; voorbeeld: verband omdoen, medicijnen geven, bloeddruk meten' answer this question:*** Hoeveel uur thuiszorg kreeg u in deze weken gemiddeld? Verpleging: gemiddeld	[ ] uur in de week
2.11	Heeft u in de afgelopen 3 maanden medicijnen gebruikt?	◯ Ja<br>◯ Nee
2.11.1	***If 'Heeft u in de afgelopen 3 maanden medicijnen gebruikt?' is equal to 'Ja' answer this question:*** Welke medicijnen heeft u in de afgelopen 3 maanden gebruikt?	[ ]
2.12	Bent u in de afgelopen 3 maanden op de spoedeisende eerste hulp van een ziekenhuis geweest? (Een andere naam voor spoedeisende eerste hulp is EHBO)	◯ Nee<br>◯ Ja
2.12.1	***If 'Bent u in de afgelopen 3 maanden op de spoedeisende eerste hulp van een ziekenhuis geweest? (Een andere naam voor spoedeisende eerste hulp is EHBO)' is equal to 'Ja' answer this question:*** Hoevaak bent u geweest?	[ ] keer
2.13	Bent u in de afgelopen 3 maanden met een ambulance naar het ziekenhuis gebracht? (Een andere naam voor ambulance is ziekenauto)	◯ Nee<br>◯ Ja

2.13.1	***If 'Bent u in de afgelopen 3 maanden met een ambulance naar het ziekenhuis gebracht? (Een andere naam voor ambulance is ziekenauto)' is equal to 'Ja' answer this question:***<br>Hoe vaak bent u naar het ziekenhuis gebracht?	☐ keer
2.14	Had u in de afgelopen 3 maanden een afspraak bij de polikliniek van het ziekenhuis? (Het gaat om afspraken voor uzelf met een dokter. Bijvoorbeeld met de cardioloog, reumatoloog of neuroloog)	○ Ja<br>○ Nee
2.14.1	***If 'Had u in de afgelopen 3 maanden een afspraak bij de polikliniek van het ziekenhuis? (Het gaat om afspraken voor uzelf met een dokter. Bijvoorbeeld met de cardioloog, reumatoloog of neuroloog)' is equal to 'Ja' answer this question:***<br>Bij welke soorten dokters bent u in de afgelopen 3 maanden in het ziekenhuis geweest? En hoe vaak?	
2.15	Bent u in de afgelopen 3 maanden overdag in het ziekenhuis geweest voor een behandeling? (U bleef dus niet slapen. U kwam bijvoorbeeld voor een bloedtransfusie, nierdialyse of chemokuur)	○ Ja<br>○ Nee
2.15.1	***If 'Bent u in de afgelopen 3 maanden overdag in het ziekenhuis geweest voor een behandeling? (U bleef dus niet slapen. U kwam bijvoorbeeld voor een bloedtransfusie, nierdialyse of chemokuur)' is equal to 'Ja' answer this question:***<br>Voor welke soort behandeling was dit?	
2.15.2	***If 'Bent u in de afgelopen 3 maanden overdag in het ziekenhuis geweest voor een behandeling? (U bleef dus niet slapen. U kwam bijvoorbeeld voor een bloedtransfusie, nierdialyse of chemokuur)' is equal to 'Ja' answer this question:***<br>Hoeveel keer moest u in de afgelopen 3 maanden voor deze behandelingen naar het ziekenhuis?	
2.16	Bent u in de afgelopen 3 maanden ergens anders geweest voor een behandeling overdag? (U bleef dus niet slapen. U ging bijvoorbeeld naar de dagopvang van een woon-/zorgcentrum of een psychiatrische instelling. Of naar de dagbehandeling van een revalidatiecentrum)	○ Ja<br>○ Nee
2.16.1	***If 'Bent u in de afgelopen 3 maanden ergens anders geweest voor een behandeling overdag? (U bleef dus niet slapen. U ging bijvoorbeeld naar de dagopvang van een woon-/zorgcentrum of een psychiatrische instelling. Of naar de dagbehandeling van een revalidatiecentrum)' is equal to 'Ja' answer this question:***<br>Wat voor instelling was dit?	☐ Woon-/zorgcentrum<br>☐ Revalidatiecentrum<br>☐ Psychiatrische instelling<br>☐ Een andere instelling, namelijk

2.16.1.1	***If 'Wat voor instelling was dit?' is equal to 'Een andere instelling, namelijk' answer this question:*** Wat voor instelling was dit?	
2.16.1.2	***If 'Wat voor instelling was dit?' is equal to 'Woon-/zorgcentrum' answer this question:*** Hoe vaak moest u hier in de afgelopen 3 maanden naartoe? Naar het woon-/zorgcentrum:	keer in de afgelopen 3 maanden
2.16.1.3	***If 'Wat voor instelling was dit?' is equal to 'Revalidatiecentrum' answer this question:*** Hoe vaak moest u hier in de afgelopen 3 maanden naartoe? Naar het revalidatiecentrum:	keer in de afgelopen 3 maanden
2.16.1.4	***If 'Wat voor instelling was dit?' is equal to 'Psychiatrische instelling' answer this question:*** Hoe vaak moest u hier in de afgelopen 3 maanden naartoe? Naar de psychiatrische instelling:	keer in de afgelopen 3 maanden
2.16.1.5	***If 'Wat voor instelling was dit?' is equal to 'Een andere instelling, namelijk' answer this question:*** Hoe vaak moest u hier in de afgelopen 3 maanden naartoe? Naar de andere instelling:	keer in de afgelopen 3 maanden
2.17	Heeft u in de afgelopen 3 maanden weleens in het ziekenhuis gelegen? (U moest dus blijven slapen. Bijvoorbeeld omdat u geopereerd was en niet meteen naar huis kon)	○ Ja<br>○ Nee
2.17.1	***If 'Heeft u in de afgelopen 3 maanden weleens in het ziekenhuis gelegen? (U moest dus blijven slapen. Bijvoorbeeld omdat u geopereerd was en niet meteen naar huis kon)' is equal to 'Ja' answer this question:*** Hoe vaak heeft u in de afgelopen 3 maanden in het ziekenhuis gelegen?	keer in de afgelopen 3 maanden
2.17.2	***If 'Heeft u in de afgelopen 3 maanden weleens in het ziekenhuis gelegen? (U moest dus blijven slapen. Bijvoorbeeld omdat u geopereerd was en niet meteen naar huis kon)' is equal to 'Ja' answer this question:*** Hoe lang heeft u in het ziekenhuis gelegen?	dagen in de afgelopen 3 maanden
2.18	Moest u in de afgelopen 3 maanden ergens anders blijven slapen voor uw gezondheid? (Bijvoorbeeld in een woon-/zorgcentrum, psychiatrische instelling of revalidatiecentrum)	○ Ja<br>○ Nee

2.18.1	***If 'Moest u in de afgelopen 3 maanden ergens anders blijven slapen voor uw gezondheid? (Bijvoorbeeld in een woon-/zorgcentrum, psychiatrische instelling of revalidatiecentrum)' is equal to 'Ja' answer this question:*** Wat voor instelling was dit?	☐ Woon-/zorgcentrum<br>☐ Revalidatiecentrum<br>☐ Psychiatrische instelling<br>☐ Een andere instelling, namelijk
2.18.1.1	***If 'Wat voor instelling was dit?' is equal to 'Een andere instelling, namelijk' answer this question:*** Een andere instelling, namelijk	
2.18.1.2	***If 'Wat voor instelling was dit?' is equal to 'Woon-/zorgcentrum' answer this question:*** Hoe lang bent u in deze instelling geweest? In het woon-/zorgcentrum:	______ dagen in de afgelopen 3 maanden
2.18.1.3	***If 'Wat voor instelling was dit?' is equal to 'Revalidatiecentrum' answer this question:*** Hoe lang bent u in deze instelling geweest? In het revalidatiecentrum:	______ dagen in de afgelopen 3 maanden
2.18.1.4	***If 'Wat voor instelling was dit?' is equal to 'Psychiatrische instelling' answer this question:*** Hoe lang bent u in deze instelling geweest? In de psychiatrische instelling:	______ dagen in de afgelopen 3 maanden
2.18.1.5	***If 'Wat voor instelling was dit?' is equal to 'Een andere instelling, namelijk' answer this question:*** Hoe lang bent u in deze instelling geweest? In de andere instelling:	______ dagen in de afgelopen 3 maanden
2.19	Heeft u in de afgelopen 3 maanden hulp gekregen van een familielid of een bekende vanwege uw lichamelijke of psychische problemen?	○ Ja<br>○ Nee
2.19.1	***If 'Heeft u in de afgelopen 3 maanden hulp gekregen van een familielid of een bekende vanwege uw lichamelijke of psychische problemen?' is equal to 'Ja' answer this question:*** Wat voor hulp van familieleden of bekenden heeft u gehad in de afgelopen 3 maanden?	☐ Huishoudelijke hulp - voorbeeld: stofzuigen, bed opmaken, boodschappen doen, klaarmaken van eten en drinken, verzorgen van kinderen<br>☐ Verzorging van uzelf - voorbeeld: hulp bij douchen of aankleden, hulp bij het eten en drinken of het geven van medicijnen<br>☐ Praktische hulp - voorbeeld: ondersteuning bij wandelen, het maken van uitstapjes of bezoekjes aan bekenden, bezoeken aan de huisarts of het ziekenhuis, het regelen van hulp of het regelen van financiële zaken

2.19.1.1	***If 'Wat voor hulp van familieleden of bekenden heeft u gehad in de afgelopen 3 maanden?' is equal to 'Huishoudelijke hulp - voorbeeld: stofzuigen, bed opmaken, boodschappen doen, klaarmaken van eten en drinken, verzorgen van kinderen' answer this question:*** Hoeveel weken heeft u deze hulp gehad? Huishoudelijke hulp:	______ weken in de afgelopen 3 maanden
2.19.1.2	***If 'Wat voor hulp van familieleden of bekenden heeft u gehad in de afgelopen 3 maanden?' is equal to 'Verzorging van uzelf - voorbeeld: hulp bij douchen of aankleden, hulp bij het eten en drinken of het geven van medicijnen' answer this question:*** Hoeveel weken heeft u deze hulp gehad? Verzorging van uzelf:	______ weken in de afgelopen 3 maanden
2.19.1.3	***If 'Wat voor hulp van familieleden of bekenden heeft u gehad in de afgelopen 3 maanden?' is equal to 'Praktische hulp - voorbeeld: ondersteuning bij wandelen, het maken van uitstapjes of bezoekjes aan bekenden, bezoeken aan de huisarts of het ziekenhuis, het regelen van hulp of het regelen van financiële zaken' answer this question:*** Hoeveel uur hulp kreeg u in deze weken gemiddeld? Praktische hulp:	______ weken in de afgelopen 3 maanden
2.19.1.4	***If 'Wat voor hulp van familieleden of bekenden heeft u gehad in de afgelopen 3 maanden?' is equal to 'Huishoudelijke hulp - voorbeeld: stofzuigen, bed opmaken, boodschappen doen, klaarmaken van eten en drinken, verzorgen van kinderen' answer this question:*** Hoeveel uur hulp kreeg u in deze weken gemiddeld? Huishoudelijke hulp: gemiddeld	______ uur in de week
2.19.1.5	***If 'Wat voor hulp van familieleden of bekenden heeft u gehad in de afgelopen 3 maanden?' is equal to 'Verzorging van uzelf - voorbeeld: hulp bij douchen of aankleden, hulp bij het eten en drinken of het geven van medicijnen' answer this question:*** Hoeveel uur hulp kreeg u in deze weken gemiddeld? Verzorging van uzelf: gemiddeld	______ uur in de week
2.19.1.6	***If 'Wat voor hulp van familieleden of bekenden heeft u gehad in de afgelopen 3 maanden?' is equal to 'Praktische hulp - voorbeeld: ondersteuning bij wandelen, het maken van uitstapjes of bezoekjes aan bekenden, bezoeken aan de huisarts of het ziekenhuis, het regelen van hulp of het regelen van financiële zaken' answer this question:*** Hoeveel uur hulp kreeg u in deze weken gemiddeld? Praktische hulp: gemiddeld	______ uur in de week

For peer review only - http://bmjopen.bmj.com/site/about/guidelines.xhtml

Vragenlijst PRIMA studie 39 weken - Dank

Number	Question	Answers
3.1	Hartelijk dank voor de tijd die u heeft genomen om de vragenlijsten in te vullen. Indien u nog opmerkingen heeft kunt u deze hierin plaatsen.	

Survey 'Vragenlijst PRIMA studie 5 jaar'

Vragenlijst PRIMA studie 5 jaar - AOFAS - Pijn

Number	Question	Answers
1.1	Hoeveel pijn ervaart u aan uw voet?	☐ Geen<br>☐ Milde pijnklachten of af en toe pijn<br>☐ Matige pijnklachten, dagelijks<br>☐ Vrijwel continu erge pijn

Vragenlijst PRIMA studie 5 jaar - AOFAS resterende gedeelte

Number	Question	Answers
2.1	Hoeveel beperkingen in het dagelijks leven ervaart u?	○ Geen beperkingen, geen hulpmiddelen nodig<br>○ Geen beperkingen in algemeen dagelijkse bezigheden, wel beperkingen in recreatieve activiteiten (sport, hobbies, etc) geen ondersteuning<br>○ Beperkingen bij dagelijkse- en recreatieve activiteiten, stok<br>○ Ernstige beperkingen bij dagelijkse- en recreatieactiviteiten, walker, krukken, rolstoel, brace

2.2	Wat is de afstand die u in één keer kunt lopen?	◯ Meer dan 600 meter<br>◯ 400 - 600 meter<br>◯ 100 - 300 meter<br>◯ Minder dan 100 meter
2.3	Heeft u moeite met lopen op een bepaalde ondergrond?	◯ Geen problemen bij lopen<br>◯ Enig problemen op ongelijke ondergrond, trap, helling, ladders<br>◯ Ernstige problemen bij ongelijke ondergrond, trap, helling, ladders
2.4	Hoe zou u uw looppatroon beoordelen?	◯ Normaal of minimaal afwijkend<br>◯ Duidelijk<br>◯ Ernstig
2.5	Hoe zou u de beweeglijkheid van uw voet beoordelen ten opzicht van de andere zijde of ten opzicte van vóór het ongeval?	◯ Hetzelfde of licht beperkt t.o.v. de niet aangedane zijde of zoals voor het ongeval<br>◯ Ongeveer de helft beperkt<br>◯ Sterk verminderd, bijna geen bewegingen mogelijk
2.6	Hoe zou u de beweeglijkheid van uw enkel beoordelen ten opzichte van de andere zijde of ten opzichte van voor het ongeval?	◯ Hetzelfde of licht beperkt t.o.v. de niet aangedane zijde of zoals voor het ongeval<br>◯ Ongeveer de helft beperkt<br>◯ Sterk verminderd, bijna geen bewegingen mogelijk
2.7	Hoe zou u de stabiliteit van uw voet en enkel beoordelen	◯ Stabiel, ik verzwik mijn enkel hooguit incidenteel<br>◯ Niet stabiel, ik verzwik mijn enkel vaak
2.8	Hoe zou u de stand van uw voet beoordelen ten opzichte van de andere zijde of ten opzichte van voor het ongeval?	◯ Goed, ik kan mijn voet goed plat op de grond zetten, waarbij de stand niet afwijkend is<br>◯ Ik sta vrijwel alleen op de buitenzijde van mijn voet<br>◯ Ik kan mijn voet niet plat op de grond zetten en loop vrijwel alleen op mijn voorvoet

Vragenlijst PRIMA studie 5 jaar - VAS Pain score

Number	Question	Answers	
3.1	VAS pijnscore (Geef uw minimaal pijnscore aan tijdens dagelijkse activiteiten op een schaal van 0 tot 100, waarbij 0 geen pijn is en 100 de ergste pijn denkbaar is)	(0.00)	100 (100.00)

Vragenlijst PRIMA studie 5 jaar - AAS

Number	Question	Answers

4.1	Wat is het meest bij u van toepassing ten aanzien van uw acitviteit?	☐ American Football<br>☐ Basketbal<br>☐ Gymnastiek/turnen<br>☐ Handbal<br>☐ Rugby<br>☐ Voetbal<br>☐ Hockey<br>☐ Korfbal<br>☐ Gevechtssporten: judo, karate, kung fu, taekwando, jiu jitsu, etc.<br>☐ Oriëntatielopen<br>☐ Rhytmische gymnastiek<br>☐ Volleybal<br>☐ Boxen<br>☐ Freestyle snowboarden<br>☐ Ijshockey<br>☐ Tennis<br>☐ Worstelen<br>☐ Fitness, aerobics<br>☐ Badminton<br>☐ Baseball<br>☐ Cross-country hardlopen<br>☐ Moderne pentathlon<br>☐ Squash<br>☐ Surfen, windsurfen<br>☐ Tafel tennis<br>☐ Atletiek: spring-, werponderdelen<br>☐ Waterskieën<br>☐ Dans<br>☐ Schermen<br>☐ Zaalhockey<br>☐ Bergbeklimmen<br>☐ Langlauf<br>☐ Parachute springen<br>☐ Softball<br>☐ Speciale beroepen en werkactiviteiten; speciale beroepen: ballet, professioneel soldaat, speciale reddingswerker, stuntman, etc.<br>☐ Duiken<br>☐ Scubaduiken<br>☐ Skaten, in-line skaten<br>☐ Atletiek: looponderdelen<br>☐ Triatlon<br>☐ Gewichtsheffen, body-building

☐ Alle competitieve sporten hieronder met 'seasonal' conditioning
☐ Zwaar fysiek werk
☐ Alpine skiën en snowboarden
☐ Bowlen/curlen
☐ Golf
☐ Mountainbike/BMX
☐ Powerliften
☐ Zeilen
☐ Fysiek werk
☐ Wielrennen
☐ Paardrijden
☐ Motorsporten/technische sporten
☐ Roeien, kayakken
☐ Boogschieten
☐ Water polo en zwemmen
☐ Kunnen lopen op oneven grond
☐ Geen sport, echter geen limitatie in dagelijkse activiteiten
☐ Kunnen lopen op even grond, maar de dagelijkste activiteiten zijn gelimiteerd
☐ Niet kunnen lopen, fysieke handicap wegens enkelproblemen

4.1.1	***If 'Wat is het meest bij u van toepassing ten aanzien van uw acitviteit?' is equal to 'American Football' answer this question:*** American Football	○ Professioneel<br>○ Competitief<br>○ Recreatief
4.1.2	***If 'Wat is het meest bij u van toepassing ten aanzien van uw acitviteit?' is equal to 'Basketbal' answer this question:*** Basketbal	○ Professioneel<br>○ Competitief<br>○ Recreatief
4.1.3	***If 'Wat is het meest bij u van toepassing ten aanzien van uw acitviteit?' is equal to 'Gymnastiek/turnen' answer this question:*** Gymnastiek/turnen	○ Professioneel<br>○ Competitief<br>○ Recreatief
4.1.4	***If 'Wat is het meest bij u van toepassing ten aanzien van uw acitviteit?' is equal to 'Handbal' answer this question:*** Handbal	○ Professioneel<br>○ Competitief<br>○ Recreatief
4.1.5	***If 'Wat is het meest bij u van toepassing ten aanzien van uw acitviteit?' is equal to 'Rugby' answer this question:*** Rugby	○ Professioneel<br>○ Competitief<br>○ Recreatief

For peer review only - http://bmjopen.bmj.com/site/about/guidelines.xhtml

4.1.6	***If 'Wat is het meest bij u van toepassing ten aanzien van uw acitviteit?' is equal to 'Voetbal' answer this question:***<br>Voetbal	○ Professioneel<br>○ Competitief<br>○ Recreatief
4.1.7	***If 'Wat is het meest bij u van toepassing ten aanzien van uw acitviteit?' is equal to 'Hockey' answer this question:***<br>Hockey	○ Professioneel<br>○ Competitief<br>○ Recreatief
4.1.8	***If 'Wat is het meest bij u van toepassing ten aanzien van uw acitviteit?' is equal to 'Korfbal' answer this question:***<br>Korfbal	○ Professioneel<br>○ Competitief<br>○ Recreatief
4.1.9	***If 'Wat is het meest bij u van toepassing ten aanzien van uw acitviteit?' is equal to 'Gevechtssporten: judo, karate, kung fu, taekwando, jiu jitsu, etc.' answer this question:***<br>Gevechtssporten: judo, karate, kung fu, taekwando, jiu jitsu, etc.	○ Professioneel<br>○ Competitief<br>○ Recreatief
4.1.10	***If 'Wat is het meest bij u van toepassing ten aanzien van uw acitviteit?' is equal to 'Oriëntatielopen' answer this question:***<br>Oriëntatielopen	○ Professioneel<br>○ Competitief<br>○ Recreatief
4.1.11	***If 'Wat is het meest bij u van toepassing ten aanzien van uw acitviteit?' is equal to 'Rhytmische gymnastiek' answer this question:***<br>Rhytmisch gymnastiek	○ Professioneel<br>○ Competitief<br>○ Recreatief
4.1.12	***If 'Wat is het meest bij u van toepassing ten aanzien van uw acitviteit?' is equal to 'Volleybal' answer this question:***<br>Volleybal	○ Professioneel<br>○ Competitief<br>○ Recreatief
4.1.13	***If 'Wat is het meest bij u van toepassing ten aanzien van uw acitviteit?' is equal to 'Boxen' answer this question:***<br>Boxen	○ Professioneel<br>○ Competitief<br>○ Recreatief
4.1.14	***If 'Wat is het meest bij u van toepassing ten aanzien van uw acitviteit?' is equal to 'Freestyle snowboarden' answer this question:***<br>Freestyle snowboarden	○ Professioneel<br>○ Competitief<br>○ Recreatief
4.1.15	***If 'Wat is het meest bij u van toepassing ten aanzien van uw acitviteit?' is equal to 'Freestyle snowboarden' answer this question:***<br>IJshockey	○ Professioneel<br>○ Competitief<br>○ Recreatief

4.1.16	***If 'Wat is het meest bij u van toepassing ten aanzien van uw acitviteit?' is equal to 'Tennis' answer this question:***<br>Tennis	◯ Professioneel<br>◯ Competitief<br>◯ Recreatief
4.1.17	***If 'Wat is het meest bij u van toepassing ten aanzien van uw acitviteit?' is equal to 'Worstelen' answer this question:***<br>Worstelen	◯ Professioneel<br>◯ Competitief<br>◯ Recreatief
4.1.18	***If 'Wat is het meest bij u van toepassing ten aanzien van uw acitviteit?' is equal to 'Fitness, aerobics' answer this question:***<br>Fitness, aerobics	◯ Professioneel<br>◯ Competitief<br>◯ Recreatief
4.1.19	***If 'Wat is het meest bij u van toepassing ten aanzien van uw acitviteit?' is equal to 'Badminton' answer this question:***<br>Badminton	◯ Professioneel<br>◯ Competitief<br>◯ Recreatief
4.1.20	***If 'Wat is het meest bij u van toepassing ten aanzien van uw acitviteit?' is equal to 'Baseball' answer this question:***<br>Baseball	◯ Professioneel<br>◯ Competitief<br>◯ Recreatief
4.1.21	***If 'Wat is het meest bij u van toepassing ten aanzien van uw acitviteit?' is equal to 'Cross-country hardlopen' answer this question:***<br>Cross-country hardlopen	◯ Professioneel<br>◯ Competitief<br>◯ Recreatief
4.1.22	***If 'Wat is het meest bij u van toepassing ten aanzien van uw acitviteit?' is equal to 'Moderne pentathlon' answer this question:***<br>Moderne pentathlon	◯ Professioneel<br>◯ Competitief<br>◯ Recreatief
4.1.23	***If 'Wat is het meest bij u van toepassing ten aanzien van uw acitviteit?' is equal to 'Squash' answer this question:***<br>Squash	◯ Professioneel<br>◯ Competitief<br>◯ Recreatief
4.1.24	***If 'Wat is het meest bij u van toepassing ten aanzien van uw acitviteit?' is equal to 'Surfen, windsurfen' answer this question:***<br>Surfen, windsurfen	◯ Professioneel<br>◯ Competitief<br>◯ Recreatief
4.1.25	***If 'Wat is het meest bij u van toepassing ten aanzien van uw acitviteit?' is equal to 'Tafel tennis' answer this question:***<br>Tafel tennis	◯ Professioneel<br>◯ Competitief<br>◯ Recreatief

4.1.26	***If 'Wat is het meest bij u van toepassing ten aanzien van uw acitviteit?' is equal to 'Atletiek: spring-, werponderdelen' answer this question:***<br>Atletiek: spring-, werponderdelen	◯ Professioneel<br>◯ Competitief<br>◯ Recreatief
4.1.27	***If 'Wat is het meest bij u van toepassing ten aanzien van uw acitviteit?' is equal to 'Waterskieën' answer this question:***<br>Waterskien	◯ Professioneel<br>◯ Competitief<br>◯ Recreatief
4.1.28	***If 'Wat is het meest bij u van toepassing ten aanzien van uw acitviteit?' is equal to 'Dans' answer this question:***<br>Dans	◯ Professioneel<br>◯ Competitief<br>◯ Recreatief
4.1.29	***If 'Wat is het meest bij u van toepassing ten aanzien van uw acitviteit?' is equal to 'Schermen' answer this question:***<br>Schermen	◯ Professioneel<br>◯ Competitief<br>◯ Recreatief
4.1.30	***If 'Wat is het meest bij u van toepassing ten aanzien van uw acitviteit?' is equal to 'Zaalhockey' answer this question:***<br>Zaalhockey	◯ Professioneel<br>◯ Competitief<br>◯ Recreatief
4.1.31	***If 'Wat is het meest bij u van toepassing ten aanzien van uw acitviteit?' is equal to 'Bergbeklimmen' answer this question:***<br>Bergbeklimmen	◯ Professioneel<br>◯ Competitief<br>◯ Recreatief
4.1.32	***If 'Wat is het meest bij u van toepassing ten aanzien van uw acitviteit?' is equal to 'Langlauf' answer this question:***<br>Langlauf	◯ Professioneel<br>◯ Competitief<br>◯ Recreatief
4.1.33	***If 'Wat is het meest bij u van toepassing ten aanzien van uw acitviteit?' is equal to 'Parachute springen' answer this question:***<br>Parachute springen	◯ Professioneel<br>◯ Competitief<br>◯ Recreatief
4.1.34	***If 'Wat is het meest bij u van toepassing ten aanzien van uw acitviteit?' is equal to 'Softball' answer this question:***<br>Softball	◯ Professioneel<br>◯ Competitief<br>◯ Recreatief

4.1.35	***If 'Wat is het meest bij u van toepassing ten aanzien van uw acitviteit?' is equal to 'Speciale beroepen en werkactiviteiten; speciale beroepen: ballet, professioneel soldaat, speciale reddingswerker, stuntman, etc.' answer this question:***<br>Speciale beroepen en werkactiviteiten speciale beroepen: ballet, professioneel soldaat, speciale reddingswerker, stuntman, etc.	◯ ballet, professioneel soldaat, speciale reddingswerker, stuntman, etc.
4.1.36	***If 'Wat is het meest bij u van toepassing ten aanzien van uw acitviteit?' is equal to 'Duiken' answer this question:***<br>Duiken	◯ Professioneel/competitief<br>◯ Recreatief
4.1.37	***If 'Wat is het meest bij u van toepassing ten aanzien van uw acitviteit?' is equal to 'Scubaduiken' answer this question:***<br>Scubaduiken	◯ Professioneel/competitief<br>◯ Recreatief
4.1.38	***If 'Wat is het meest bij u van toepassing ten aanzien van uw acitviteit?' is equal to 'Skaten, in-line skaten' answer this question:***<br>skaten, in-linen skaten	◯ Professioneel/competitief<br>◯ Recreatief
4.1.39	***If 'Wat is het meest bij u van toepassing ten aanzien van uw acitviteit?' is equal to 'Atletiek: looponderdelen' answer this question:***<br>Atletiek: looponderdelen	◯ Professioneel/competitief<br>◯ Recreatief
4.1.40	***If 'Wat is het meest bij u van toepassing ten aanzien van uw acitviteit?' is equal to 'Triatlon' answer this question:***<br>triatlon	◯ Professioneel/competitief<br>◯ Recreatief
4.1.41	***If 'Wat is het meest bij u van toepassing ten aanzien van uw acitviteit?' is equal to 'Gewichtsheffen, body-building' answer this question:***<br>Gewichtsheffen, body-building	◯ Professioneel/competitief<br>◯ Recreatief
4.1.42	***If 'Wat is het meest bij u van toepassing ten aanzien van uw acitviteit?' is equal to 'Alle competitieve sporten hieronder met 'seasonal' conditioning' answer this question:***<br>Alle competitieve sporten hieronder met 'seasonal' conditioning	◯ Alle competitieve sporten hieronder met 'seasonal' conditioning
4.1.43	***If 'Wat is het meest bij u van toepassing ten aanzien van uw acitviteit?' is equal to 'Zwaar fysiek werk' answer this question:***<br>Zwaar fysiek werk	◯ Alle competitieve sporten hieronder met 'seasonal' conditioning

4.1.44	***If 'Wat is het meest bij u van toepassing ten aanzien van uw acitviteit?' is equal to 'Alpine skiën en snowboarden' answer this question:***<br>Alpine skien en snowboarden	○ Alpine skien en snowboarden
4.1.45	***If 'Wat is het meest bij u van toepassing ten aanzien van uw acitviteit?' is equal to 'Bowlen/curlen' answer this question:***<br>Bowlen/curlen	○ Bowlen/curlen
4.1.46	***If 'Wat is het meest bij u van toepassing ten aanzien van uw acitviteit?' is equal to 'Golf' answer this question:***<br>Golf	○ Golf
4.1.47	***If 'Wat is het meest bij u van toepassing ten aanzien van uw acitviteit?' is equal to 'Mountainbike/BMX' answer this question:***<br>Mountainbike/BMX	○ Mountainbike/BMX
4.1.48	***If 'Wat is het meest bij u van toepassing ten aanzien van uw acitviteit?' is equal to 'Powerliften' answer this question:***<br>Powerliften	○ Powerliften
4.1.49	***If 'Wat is het meest bij u van toepassing ten aanzien van uw acitviteit?' is equal to 'Zeilen' answer this question:***<br>Zeilen	○ Zeilen
4.1.50	***If 'Wat is het meest bij u van toepassing ten aanzien van uw acitviteit?' is equal to 'Fysiek werk' answer this question:***<br>Fysiek werk	○ Fysiek werk
4.1.51	***If 'Wat is het meest bij u van toepassing ten aanzien van uw acitviteit?' is equal to 'Wielrennen' answer this question:***<br>Wielrennen	○ Wielrennen
4.1.52	***If 'Wat is het meest bij u van toepassing ten aanzien van uw acitviteit?' is equal to 'Paardrijden' answer this question:***<br>Paardrijden	○ Paardrijden
4.1.53	***If 'Wat is het meest bij u van toepassing ten aanzien van uw acitviteit?' is equal to 'Motorsporten/technische sporten' answer this question:***<br>Motorsporten/technische sporten	○ Motorsporten/technische sporten
4.1.54	***If 'Wat is het meest bij u van toepassing ten aanzien van uw acitviteit?' is equal to 'Roeien, kayakken' answer this question:***<br>Roeien, kayakken	○ Roeien, kayakken

For peer review only - http://bmjopen.bmj.com/site/about/guidelines.xhtml

4.1.55	***If 'Wat is het meest bij u van toepassing ten aanzien van uw acitviteit?' is equal to 'Boogschieten' answer this question:*** Boogschieten	○ Boogschieten
4.1.56	***If 'Wat is het meest bij u van toepassing ten aanzien van uw acitviteit?' is equal to 'Water polo en zwemmen' answer this question:*** Water polo en zwemmen	○ Water polo en zwemmen
4.1.57	***If 'Wat is het meest bij u van toepassing ten aanzien van uw acitviteit?' is equal to 'Kunnen lopen op oneven grond' answer this question:*** Kunnen lopen op oneven grond	○ Kunnen lopen op oneven grond
4.1.58	***If 'Wat is het meest bij u van toepassing ten aanzien van uw acitviteit?' is equal to 'Geen sport, echter geen limitatie in dagelijkse activiteiten' answer this question:*** Geen sport, echter geen limitatie in dagelijkse activiteiten	○ Geen sport, echter geen limitatie in dagelijkse activiteiten
4.1.59	***If 'Wat is het meest bij u van toepassing ten aanzien van uw acitviteit?' is equal to 'Kunnen lopen op even grond, maar de dagelijkste activiteiten zijn gelimiteerd' answer this question:*** Kunnen lopen op even grond, maar de dagelijkste activiteiten zijn gelimiteerd	○ Kunnen lopen op even grond, maar de dagelijkste activiteiten zijn gelimiteerd
4.1.60	***If 'Wat is het meest bij u van toepassing ten aanzien van uw acitviteit?' is equal to 'Niet kunnen lopen, fysieke handicap wegens enkelproblemen' answer this question:*** Niet kunnen lopen, fysieke handicap wegens enkelproblemen	○ Niet kunnen lopen, fysieke handicap wegens enkelproblemen

Vragenlijst PRIMA studie 5 jaar - Hoe tevreden bent u over de enkelklachten?

Number	Question	Answers
5.1	Hoe tevreden bent u over de enkelklachten?	○ Slecht<br>○ Matig<br>○ Goed<br>○ Uitstekend

Vragenlijst PRIMA studie 5 jaar - SF-36

Number	Question	Answers
	Deze vragenlijst gaat over uw standpunten t.a.v. uw gezondheid. Met behulp van deze gegevens kan worden bijgehouden hoe u zich voelt en hoe goed u in staat bent uw gebruikelijke bezigheden uit te voeren. Beantwoord elke vraag door een antwoord aan te klikken. Als u niet zeker weet hoe u een vraag moet beantwoorden, geef dan het best mogelijke antwoord.	
6.1	Hoe zou u over het algemeen uw gezondheid noemen?	◯ Uitstekend ◯ Zeer goed ◯ Goed ◯ Matig ◯ Slecht
6.2	Hoe beoordeelt u nu uw gezondheid over het algemeen vergeleken met een jaar geleden?	◯ Veel beter dan een jaar geleden ◯ Wat beter dan een jaar geleden ◯ Ongeveer hetzelfde als een jaar geleden ◯ Wat slechter dan een jaar geleden ◯ Veel slechter dan een jaar geleden
6.3	Wordt u door uw gezondheid op dit moment beperkt bij forse inspanning, zoals hardlopen, tillen van zware voorwerpen of een veeleisende sport beoefenen?	◯ Ja, ernstig beperkt ◯ Ja, een beetje beperkt ◯ Nee, helemaal niet beperkt
6.4	Wordt u door uw gezondheid op dit moment beperkt bij matige inspanning zoals een tafel verplaatsen, stofzuigen, zwemmen of fietsen?	◯ Ja, ernstig beperkt ◯ Ja, een beetje beperkt ◯ Nee, helemaal niet beperkt
6.5	Wordt u door uw gezondheid op dit moment beperkt bij boodschappen tillen of dragen?	◯ Ja, ernstig beperkt ◯ Ja, een beetje beperkt ◯ Nee, helemaal niet beperkt
6.6	Wordt u door uw gezondheid op dit moment beperkt bij een paar trappen oplopen?	◯ Ja, ernstig beperkt ◯ Ja, een beetje beperkt ◯ Nee, helemaal niet beperkt
6.7	Wordt u door uw gezondheid op dit moment beperkt bij één trap oplopen?	◯ Ja, ernstig beperkt ◯ Ja, een beetje beperkt ◯ Nee, helemaal niet beperkt
6.8	Wordt u door uw gezondheid op dit moment beperkt bij bukken, knielen of hurken?	◯ Ja, ernstig beperkt ◯ Ja, een beetje beperkt ◯ Nee, helemaal niet beperkt
6.9	Wordt u door uw gezondheid op dit moment beperkt bij meer dan één kilometer lopen?	◯ Ja, ernstig beperkt ◯ Ja, een beetje beperkt ◯ Nee, helemaal niet beperkt
6.10	Wordt u door uw gezondheid op dit moment beperkt bij een paar honderd meter lopen?	◯ Ja, ernstig beperkt ◯ Ja, een beetje beperkt ◯ Nee, helemaal niet beperkt

For peer review only - http://bmjopen.bmj.com/site/about/guidelines.xhtml

6.11	Wordt u door uw gezondheid op dit moment beperkt bij ongeveer honderd meter lopen?	◯ Ja, ernstig beperkt ◯ Ja, een beetje beperkt ◯ Nee, helemaal niet beperkt
6.12	Wordt u door uw gezondheid op dit moment beperkt bij uzelf wassen of aankleden?	◯ Ja, ernstig beperkt ◯ Ja, een beetje beperkt ◯ Nee, helemaal niet beperkt
6.13	U besteedde in de afgelopen 4 weken minder tijd aan werk of andere bezigheden	◯ Ja ◯ Nee
6.14	U heeft in de afgelopen 4 weken minder bereikt dan u zou willen	◯ Ja ◯ Nee
6.15	U was in de afgelopen 4 weken beperkt in het soort werk of andere bezigheden.	◯ Ja ◯ Nee
6.16	U had de afgelopen 4 weken moeite om uw werk of andere bezigheden uit te voeren (het kostte u bijvoorbeeld extra inspanning).	◯ Ja ◯ Nee
6.17	U besteedde in de afgelopen 4 weken minder tijd aan werk of andere bezigheden ten gevolge van emotionele problemen	◯ Ja ◯ Nee
6.18	U heeft in de afgelopen 4 weken minder bereikt dan u zou willen ten gevolge van emotionele problemen.	◯ Ja ◯ Nee
6.19	U deed de afgelopen 4 weken uw werk of andere bezigheden niet zo zorgvuldig als gewoonlijk ten gevolge van emotionele problemen.	◯ Ja ◯ Nee
6.20	In hoeverre hebben uw lichamelijke gezondheid of emotionele problemen u gedurende de afgelopen 4 weken gehinderd in uw normale omgang met familie, vrienden of buren, of bij activiteiten in groepsverband?	◯ Helemaal niet ◯ Enigszins ◯ Nogal ◯ Veel ◯ Heel erg veel
6.21	Hoeveel lichamelijke pijn heeft u de afgelopen 4 weken gehad?	◯ Geen<br>◯ Heel licht<br>◯ Licht<br>◯ Nogal<br>◯ Ernstig<br>◯ Heel ernstig
6.22	In welke mate bent u de afgelopen 4 weken door de pijn gehinderd in uw normale werk (zowel werk buitenshuis als huishoudelijk werk)?	◯ Helemaal niet<br>◯ Een klein beetje<br>◯ Nogal<br>◯ Veel<br>◯ Heel erg veel

6.23	Voelde u zich levenslustig?	◯ Altijd<br>◯ Meestal<br>◯ Vaak<br>◯ Soms<br>◯ Zelden<br>◯ Nooit
6.24	Was u erg zenuwachtig?	◯ Altijd<br>◯ Meestal<br>◯ Vaak<br>◯ Soms<br>◯ Zelden<br>◯ Nooit
6.25	Zat u zo in de put dat u niets kon?	◯ Altijd<br>◯ Meestal<br>◯ Vaak<br>◯ Soms<br>◯ Zelden<br>◯ Nooit
6.26	Voelde u zich rustig en tevreden?	◯ Altijd<br>◯ Meestal<br>◯ Vaak<br>◯ Soms<br>◯ Zelden<br>◯ Nooit
6.27	Had u veel energie?	◯ Altijd<br>◯ Meestal<br>◯ Vaak<br>◯ Soms<br>◯ Zelden<br>◯ Nooit
6.28	Voelde u zich somber en neerslachtig?	◯ Altijd<br>◯ Meestal<br>◯ Vaak<br>◯ Soms<br>◯ Zelden<br>◯ Nooit

6.29	Voelde u zich uitgeput?	◯ Altijd<br>◯ Meestal<br>◯ Vaak<br>◯ Soms<br>◯ Zelden<br>◯ Nooit
6.30	Was u een gelukkig mens?	◯ Altijd<br>◯ Meestal<br>◯ Vaak<br>◯ Soms<br>◯ Zelden<br>◯ Nooit
6.31	Voelde u zich moe?	◯ Altijd<br>◯ Meestal<br>◯ Vaak<br>◯ Soms<br>◯ Zelden<br>◯ Nooit
6.32	Hoe vaak hebben uw lichamelijke gezondheid of emotionele problemen u gedurende de afgelopen 4 weken gehinderd bij uw sociale activiteiten (zoals vrienden of familie bezoeken etc.)?	◯ Altijd<br>◯ Meestal<br>◯ Soms<br>◯ Zelden<br>◯ Nooit
6.33	Ik lijk wat gemakkelijker ziek te worden dan andere mensen	◯ Volkomen juist<br>◯ Grotendeels juist<br>◯ Weet ik niet<br>◯ Grotendeels onjuist<br>◯ Volkomen onjuist
6.34	Ik ben even gezond als andere mensen die ik ken	◯ Volkomen juist<br>◯ Grotendeels juist<br>◯ Weet ik niet<br>◯ Grotendeels onjuist<br>◯ Volkomen onjuist

6.35	Ik verwacht dat mijn gezondheid achteruit zal gaan	◯ Volkomen juist<br>◯ Grotendeels juist<br>◯ Weet ik niet<br>◯ Grotendeels onjuist<br>◯ Volkomen onjuist
6.36	Mijn gezondheid is uitstekend	◯ Volkomen juist<br>◯ Grotendeels juist<br>◯ Weet ik niet<br>◯ Grotendeels onjuist<br>◯ Volkomen onjuist

Vragenlijst PRIMA studie 5 jaar - GAS

Number	Question	Answers
	Tijdens de eerste afspraak werd een duidelijk doel met u afgesproken. Dit werd tevens per email naar u gestuurd. Hoe is de situatie nu ten aanzien van de destijds gestelde doelstelling?	
7.1	Wat is nu de situatie ten aanzien van het tijdens de eerste afspraak afgesproken doelstelling (Goal attainment Scaling)?	◯ Achteruitgang (minder dan de utigangssituatie)<br>◯ Uitgangssituatie<br>◯ Minder dan het doel<br>◯ Doel<br>◯ Meer dan het doel<br>◯ Veel meer dan het doel

Vragenlijst PRIMA studie 5 jaar - EQ-5D-3L

Number	Question	Answers
	Deze vragenlijst gaat over uw standpunten t.a.v. uw gezondheid. Met behulp van deze gegevens kan worden bijgehouden hoe u zich voelt en hoe goed u in staat bent uw gebruikelijke bezigheden uit te voeren. Beantwoord elke vraag door een antwoord aan te klikken. Als u niet zeker weet hoe u een vraag moet beantwoorden, geef dan het best mogelijke antwoord.	
8.1	Hoe is het met uw Mobiliteit gesteld?	◯ Ik heb geen problemen met lopen<br>◯ Ik heb enige problemen met lopen<br>◯ Ik ben bedlegerig

8.2	Hoe is het met uw Zelfzorg gesteld?	◯ Ik heb geen problemen om mijzelf te wassen of aan te kleden<br>◯ Ik heb enige problemen om mijzelf te wassen of aan te kleden<br>◯ Ik ben niet in staat om mijzelf te wassen of aan te kleden
8.3	Hoe is het met de Dagelijkse activiteiten (werk, studie, huishouden, gezins- en vrijetijdsactiviteiten) gesteld?	◯ Ik heb geen problemen met mijn dagelijkse activiteiten<br>◯ Ik heb enige problemen met mijn dagelijkse activiteiten<br>◯ Ik ben niet in staat om mijn dagelijkse activiteiten uit te voeren
8.4	Hoe is het met de Pijn/klachten gesteld?	◯ Ik heb geen pijn of andere klachten<br>◯ Ik heb matige pijn of andere klachten<br>◯ Ik heb zeer ernstige pijn of andere klachten
8.5	Hoe is het met de Stemming gesteld?	◯ Ik ben niet angstig of somber<br>◯ Ik ben matig angstig of somber<br>◯ Ik ben erg angstig of somber
	We willen weten hoe goed of slecht uw gezondheid VANDAAG is. Deze meetschaal loopt van 0 tot 100: 100 staat voor de BESTE gezondheid die u zich kunt voorstellen - 0 staat voor de SLECHTSTE gezondheid die u zich kunt voorstellen	
8.6	Uw Gezondheid vandaag	(0.00) (100.00)

Vragenlijst PRIMA studie 5 jaar - AOS

Number	Question	Answers
	Instructies: De lijn naast elke vraag staat voor hoeveel PIJN u heeft in verschillende situaties. De linker kant (0) is “geen pijn” en de rechter kant (100) is “ergste pijn denkbaar”. Geef voor de onderstaande situaties op de lijn aan hoeveel PIJN u in de afgelopen week in de enkel had. Als een of meerdere van deze situaties niet van toepassing waren, dan kiest u de "niet van toepassing" optie.	
9.1	Wat was de hoogte van de ergste pijn in de afgelopen week?	(0.00) (100.00)
9.2	Heeft u pijn als u 's ochtends opstaat?	◯ Ja<br>◯ Nee<br>◯ Niet van toepassing

9.2.1	***If 'Heeft u pijn als u 's ochtends opstaat?' is equal to 'Ja' answer this question:***<br>Hoeveel pijn heeft u voordat u 's ochtends opstaat?	(0.00)	(100.00)
9.3	Heeft u pijn wanneer u op blote voeten loopt?	◯ Ja<br>◯ Nee<br>◯ Niet van toepassing	
9.3.1	***If 'Heeft u pijn wanneer u op blote voeten loopt?' is equal to 'Ja' answer this question:***<br>Hoeveel pijn heeft u als u op blote voeten loopt?	(0.00)	(100.00)
9.4	Heeft u pijn wanneer u op blote voeten staat?	◯ Ja<br>◯ Nee<br>◯ Niet van toepassing	
9.4.1	***If 'Heeft u pijn wanneer u op blote voeten staat?' is equal to 'Ja' answer this question:***<br>Hoeveel pijn heeft u als u op blote voeten staat?	(0.00)	(100.00)
9.5	Heeft u pijn wanneer u met schoenen loopt?	◯ Ja<br>◯ Nee<br>◯ Niet van toepassing	
9.5.1	***If 'Heeft u pijn wanneer u met schoenen loopt?' is equal to 'Ja' answer this question:***<br>Hoeveel pijn heeft u wanneer u met schoenen loopt?	(0.00)	(100.00)
9.6	Heeft u pijn wanneer u met schoenen staat?	◯ Ja<br>◯ Nee<br>◯ Niet van toepassing	
9.6.1	***If 'Heeft u pijn wanneer u met schoenen staat?' is equal to 'Ja' answer this question:***<br>Hoeveel pijn heeft u wanneer u met schoenen staat?	(0.00)	(100.00)
9.7	Heeft u pijn wanneer u loopt met steunzolen of een brace?	◯ Ja<br>◯ Nee<br>◯ Niet van toepassing	
9.7.1	***If 'Heeft u pijn wanneer u loopt met steunzolen of een brace?' is equal to 'Ja' answer this question:***<br>Hoeveel pijn heeft u wanneer u loopt met steunzolen of een brace?	(0.00)	(100.00)

9.8	Heeft u pijn wanneer u staat met steunzolen of een brace?	◯ Ja<br>◯ Nee<br>◯ Niet van toepassing	
9.8.1	***If 'Heeft u pijn wanneer u staat met steunzolen of een brace?' is equal to 'Ja' answer this question:***<br>Hoeveel pijn heeft u wanneer u staat met steunzolen of een brace?	(0.00)	(100.00)
9.9	Heeft u pijn aan het einde van de dag?	◯ Ja<br>◯ Nee<br>◯ Niet van toepassing	
9.9.1	***If 'Heeft u pijn aan het einde van de dag?' is equal to 'Ja' answer this question:***<br>Hoeveel pijn heeft u aan het einde van de dag?	(0.00)	(100.00)
	Instructies:De lijn naast elke vraag staat voor hoeveel MOEITE u heeft met verschillende activiteiten. De linker kant (0) is “Niet moeilijk” en de rechter kant (100) is “Te moeilijk, niet uitvoerbaar”. Geef voor de onderstaande activiteiten op de lijn aan hoeveel MOEITE u in de afgelopen week door enkelklachten had met onderstaande activiteiten. Als een situatie niet van toepassing is, kies dan de optie "niet van toepassing".		
9.10	Heeft u moeite met door het huis lopen?	◯ Ja<br>◯ Nee<br>◯ Niet van toepassing	
9.10.1	***If 'Heeft u moeite met door het huis lopen?' is equal to 'Ja' answer this question:***<br>Hoeveel moeite heeft u met door het huis lopen?	(0.00)	(100.00)
9.11	Heeft u moeite met buiten lopen op oneven ondergrond?	◯ Ja<br>◯ Nee<br>◯ Niet van toepassing	
9.11.1	***If 'Heeft u moeite met buiten lopen op oneven ondergrond?' is equal to 'Ja' answer this question:***<br>Hoeveel moeite heeft u met buiten lopen op oneven ondergrond?	(0.00)	(100.00)
9.12	Heeft u moeite met een paar honderd meter lopen?	◯ Ja<br>◯ Nee<br>◯ Niet van toepassing	
9.12.1	***If 'Heeft u moeite met een paar honderd meter lopen?' is equal to 'Ja' answer this question:***<br>Hoeveel moeite heeft u met een paar honderd meter lopen?	(0.00)	(100.00)

9.13	Heeft u moeite met een trap oplopen?	◯ Ja<br>◯ Nee<br>◯ Niet van toepassing	
9.13.1	***If 'Heeft u moeite met een trap oplopen?' is equal to 'Ja' answer this question:***<br>Hoeveel moeite heeft u met een trap oplopen?	(0.00)	(100.00)
9.14	Heeft u moeite met een trap aflopen?	◯ Ja<br>◯ Nee<br>◯ Niet van toepassing	
9.14.1	***If 'Heeft u moeite met een trap aflopen?' is equal to 'Ja' answer this question:***<br>Hoeveel moeite heeft u met een trap aflopen?	(0.00)	(100.00)
9.15	Heeft u moeite met op de tenen staan?	◯ Ja<br>◯ Nee<br>◯ Niet van toepassing	
9.15.1	***If 'Heeft u moeite met op de tenen staan?' is equal to 'Ja' answer this question:***<br>Hoeveel moeite heeft u met op de tenen staan?	(0.00)	(100.00)
9.16	Heeft u moeite met opstaan uit de stoel?	◯ Ja<br>◯ Nee<br>◯ Niet van toepassing	
9.16.1	***If 'Heeft u moeite met opstaan uit de stoel?' is equal to 'Ja' answer this question:***<br>Hoeveel moeite heeft u met opstaan uit de stoel?	(0.00)	(100.00)
9.17	Heeft u moeite met het op- of afstappen van stoepranden?	◯ Ja<br>◯ Nee<br>◯ Niet van toepassing	
9.17.1	***If 'Heeft u moeite met het op- of afstappen van stoepranden?' is equal to 'Ja' answer this question:***<br>Hoeveel moeite heeft u met het op- of afstappen van stoepranden?	(0.00)	(100.00)
9.18	Heeft u moeite met snel lopen of rennen?	◯ Ja<br>◯ Nee<br>◯ Niet van toepassing	

For peer review only - http://bmjopen.bmj.com/site/about/guidelines.xhtml

9.18.1	***If 'Heeft u moeite met snel lopen of rennen?' is equal to 'Ja' answer this question:*** Hoeveel moeite heeft u met snel lopen of rennen?	(0.00)	(100.00)

Vragenlijst PRIMA studie 5 jaar - FAOS

Number	Question	Answers
	Deze lijst vraagt naar uw mening over uw voet/enkel. Uw antwoorden geven ons een beeld van uw voet/enkel klachten en hoe u in staat bent om alledaagse activiteiten uit te voeren in uw huidige situatie. Beantwoorden van een vraag doet u door het aanklikken van een vakje met het volgens u meest juiste antwoord (één vakje per vraag). Als u niet zeker weet hoe u een vraag moet beantwoorden, geeft u dan het antwoord dat volgens u het meest op uw situatie van toepassing is. Deze vraag heeft betrekking op het voorkomen van voet/enkel klachten in de afgelopen week.	
10.1	Is uw voet/enkel gezwollen?	◯ Nooit ◯ Zelden ◯ Soms ◯ Vaak ◯ Altijd
10.2	Voelt u gekraak of hoort u klikken of een ander vreemd geluid wanneer u de voet/enkel beweegt?	◯ Nooit ◯ Zelden ◯ Soms ◯ Vaak ◯ Altijd
10.3	Hapert uw enkel of blokkeert uw enkel ('op slot' gaan zitten) wanneer u deze beweegt?	◯ Nooit ◯ Zelden ◯ Soms ◯ Vaak ◯ Altijd
10.4	Kunt u de voet/enkel volledig strekken?	◯ Altijd ◯ Vaak ◯ Soms ◯ Zelden ◯ Nooit
10.5	Kunt u de voet volledig naar u toe buigen?	◯ Altijd ◯ Vaak ◯ Soms ◯ Zelden ◯ Nooit
10.6	In welke mate heeft u een stijf gevoel in de voet/enkel 's ochtends bij het wakker worden?	◯ Niet ◯ Mild ◯ Matig ◯ Ernstig ◯ Zeer ernstig
10.7	In welke mate heeft u een stijf gevoel in de voet/enkel na zitten, liggen of rusten later op de dag?	◯ Niet ◯ Mild ◯ Matig ◯ Ernstig ◯ Zeer ernstig
10.8	Hoe vaak heeft u pijn in uw voet/enkel?	◯ Nooit ◯ Maandelijks ◯ Wekelijks ◯ Dagelijks ◯ Altijd

10.9	Hoeveel voet/enkel pijn heeft u gehad in de afgelopen week bij draaien als uw voet/enkel op de grond staat?	◯ Geen<br>◯ Mild<br>◯ Matig<br>◯ Ernstig<br>◯ Zeer ernstig
10.10	Hoeveel voet/enkel pijn heeft u gehad in de afgelopen week bij het volledig uitstrekken van de voet/enkel?	◯ Geen<br>◯ Mild<br>◯ Matig<br>◯ Ernstig<br>◯ Zeer ernstig
10.11	Hoeveel voet/enkel pijn heeft u gehad in de afgelopen week bij het volledig naar u toe buigen/optrekken van de voet/enkel	◯ Geen<br>◯ Mild<br>◯ Matig<br>◯ Ernstig<br>◯ Zeer ernstig
10.12	Hoeveel voet/enkel pijn heeft u gehad in de afgelopen week bij het lopen op een vlakke ondergrond	◯ Geen<br>◯ Mild<br>◯ Matig<br>◯ Ernstig<br>◯ Zeer ernstig
10.13	Hoeveel voet/enkel pijn heeft u gehad in de afgelopen week bij het trap op- en trap af lopen?	◯ Geen<br>◯ Mild<br>◯ Matig<br>◯ Ernstig<br>◯ Zeer ernstig
10.14	Hoeveel voet/enkel pijn heeft u gehad in de afgelopen week 's nachts in bed?	◯ Geen<br>◯ Mild<br>◯ Matig<br>◯ Ernstig<br>◯ Zeer ernstig
10.15	Hoeveel voet/enkel pijn heeft u gehad in de afgelopen week bij het zitten of liggen?	◯ Geen<br>◯ Mild<br>◯ Matig<br>◯ Ernstig<br>◯ Zeer ernstig

For peer review only - http://bmjopen.bmj.com/site/about/guidelines.xhtml

10.16	Hoeveel voet/enkel pijn heeft u gehad in de afgelopen week bij het rechtop staan?	◯ Geen<br>◯ Mild<br>◯ Matig<br>◯ Ernstig<br>◯ Zeer ernstig
10.17	In welke mate werd u gehinderd bij het trap aflopen?	◯ Niet ◯ Mild ◯ Matig ◯ Ernstig ◯ Zeer ernstig
10.18	In welke mate werd u gehinderd bij het trap op lopen?	◯ Niet ◯ Mild ◯ Matig ◯ Ernstig ◯ Zeer ernstig
10.19	In welke mate werd u gehinderd als u vanuit een zittende positie ging staan?	◯ Niet ◯ Mild ◯ Matig ◯ Ernstig ◯ Zeer ernstig
10.20	In welke mate werd u gehinderd bij het staan?	◯ Niet ◯ Mild ◯ Matig ◯ Ernstig ◯ Zeer ernstig
10.21	In welke mate werd u gehinderd bij het naar de grond buigen/iets oprapen?	◯ Niet ◯ Mild ◯ Matig ◯ Ernstig ◯ Zeer ernstig
10.22	In welke mate werd u gehinderd bij het lopen op een vlakke ondergrond?	◯ Niet ◯ Mild ◯ Matig ◯ Ernstig ◯ Zeer ernstig
10.23	In welke mate werd u gehinderd bij het in- en uit de auto stappen?	◯ Niet ◯ Mild ◯ Matig ◯ Ernstig ◯ Zeer ernstig
10.24	In welke mate werd u gehinderd bij het boodschappen doen?	◯ Niet ◯ Mild ◯ Matig ◯ Ernstig ◯ Zeer ernstig
10.25	In welke mate werd u gehinderd bij sokken/panty's aantrekken?	◯ Niet ◯ Mild ◯ Matig ◯ Ernstig ◯ Zeer ernstig
10.26	In welke mate werd u gehinderd bij het opstaan uit bed?	◯ Niet ◯ Mild ◯ Matig ◯ Ernstig ◯ Zeer ernstig
10.27	In welke mate werd u gehinderd bij het sokken uittrekken?	◯ Niet ◯ Mild ◯ Matig ◯ Ernstig ◯ Zeer ernstig
10.28	In welke mate werd u gehinderd bij het in bed liggen (omdraaien, lange tijd uw voet/enkel in dezelfde positie houden)?	◯ Niet ◯ Mild ◯ Matig ◯ Ernstig ◯ Zeer ernstig

10.29	In welke mate werd u gehinderd bij het in/uit bad stappen?	○ Niet ○ Mild ○ Matig ○ Ernstig ○ Zeer ernstig
10.30	In welke mate werd u gehinderd bij zitten?	○ Niet ○ Mild ○ Matig ○ Ernstig ○ Zeer ernstig
10.31	In welke mate werd u gehinderd bij het toilet op en af gaan?	○ Niet ○ Mild ○ Matig ○ Ernstig ○ Zeer ernstig
10.32	In welke mate werd u gehinderd bij zwaar huishoudelijk werk (bijvoorbeeld zware dozen sjouwen, vloer schrobben)?	○ Niet ○ Mild ○ Matig ○ Ernstig ○ Zeer ernstig
10.33	In welke mate werd u gehinderd bij licht huishoudeijk werk (bijvoorbeeld koken, afstoffen)?	○ Niet ○ Mild ○ Matig ○ Ernstig ○ Zeer ernstig
10.34	In welke mate werd u gehinderd bij hurken?	○ Niet ○ Mild ○ Matig ○ Ernstig ○ Zeer ernstig
10.35	In welke mate werd u gehinderd bij hardlopen?	○ Niet ○ Mild ○ Matig ○ Ernstig ○ Zeer ernstig
10.36	In welke mate werd u gehinderd bij springen?	○ Niet ○ Mild ○ Matig ○ Ernstig ○ Zeer ernstig
10.37	In welke mate werd u gehinderd bij ronddraaien op uw aangedane voet/enkel?	○ Niet ○ Mild ○ Matig ○ Ernstig ○ Zeer ernstig
10.38	In welke mate werd u gehinderd bij knielen?	○ Niet ○ Mild ○ Matig ○ Ernstig ○ Zeer ernstig
10.39	Hoe vaak bent u zich bewust van uw voet/enkel probleem?	○ Nooit ○ Maandelijks ○ Wekelijks ○ Dagelijks ○ Altijd
10.40	Heeft u uw leven veranderd om activiteiten te vermijden die schadelijk kunnen zijn voor uw voet/enkel?	○ Niet<br>○ Enigszins<br>○ Matig<br>○ Behoorlijk<br>○ Volledig

10.41	In hoeverre kunt u op uw voet/enkel vertrouwen?	◯ Volledig<br>◯ Behoorlijk<br>◯ Matig<br>◯ Enigzins<br>◯ Niet
10.42	In het algemeen, in welke mate ondervindt u hinder van uw voet/enkel	◯ Geen<br>◯ Mild<br>◯ Matig<br>◯ Ernstig<br>◯ Zeer ernstig

Vragenlijst PRIMA studie 5 jaar - Dank

Number	Question	Answers
11.1	Hartelijk dank voor de tijd die u heeft genomen om de vragenlijsten in te vullen. Indien u nog opmerkingen heeft kunt u deze hierin plaatsen.	

Survey 'Vragenlijst PRIMA studie 52 weken'

Vragenlijst PRIMA studie 52 weken - AOFAS - Pijn

Number	Question	Answers
1.1	Hoeveel pijn ervaart u aan uw voet?	☐ Geen<br>☐ Milde pijnklachten of af en toe pijn<br>☐ Matige pijnklachten, dagelijks<br>☐ Vrijwel continu erge pijn

Vragenlijst PRIMA studie 52 weken - AOFAS resterende gedeelte

Number	Question	Answers
2.1	Hoeveel beperkingen in het dagelijks leven ervaart u?	◯ Geen beperkingen, geen hulpmiddelen nodig<br>◯ Geen beperkingen in algemeen dagelijkse bezigheden, wel beperkingen in recreatieve activiteiten (sport, hobbies, etc) geen ondersteuning<br>◯ Beperkingen bij dagelijkse- en recreatieve activiteiten, stok<br>◯ Ernstige beperkingen bij dagelijkse- en recreatieactiviteiten, walker, krukken, rolstoel, brace
2.2	Wat is de afstand die u in één keer kunt lopen?	◯ Meer dan 600 meter<br>◯ 400 - 600 meter<br>◯ 100 - 300 meter<br>◯ Minder dan 100 meter
2.3	Heeft u moeite met lopen op een bepaalde ondergrond?	◯ Geen problemen bij lopen<br>◯ Enig problemen op ongelijke ondergrond, trap, helling, ladders<br>◯ Ernstige problemen bij ongelijke ondergrond, trap, helling, ladders
2.4	Hoe zou u uw looppatroon beoordelen?	◯ Normaal of minimaal afwijkend<br>◯ Duidelijk<br>◯ Ernstig
2.5	Hoe zou u de beweeglijkheid van uw voet beoordelen ten opzicht van de andere zijde of ten opzicte van vóór het ongeval?	◯ Hetzelfde of licht beperkt t.o.v. de niet aangedane zijde of zoals voor het ongeval<br>◯ Ongeveer de helft beperkt<br>◯ Sterk verminderd, bijna geen bewegingen mogelijk
2.6	Hoe zou u de beweeglijkheid van uw enkel beoordelen ten opzichte van de andere zijde of ten opzichte van voor het ongeval?	◯ Hetzelfde of licht beperkt t.o.v. de niet aangedane zijde of zoals voor het ongeval<br>◯ Ongeveer de helft beperkt<br>◯ Sterk verminderd, bijna geen bewegingen mogelijk
2.7	Hoe zou u de stabiliteit van uw voet en enkel beoordelen	◯ Stabiel, ik verzwik mijn enkel hooguit incidenteel<br>◯ Niet stabiel, ik verzwik mijn enkel vaak

2.8	Hoe zou u de stand van uw voet beoordelen ten opzichte van de andere zijde of ten opzichte van voor het ongeval?	◯ Goed, ik kan mijn voet goed plat op de grond zetten, waarbij de stand niet afwijkend is<br>◯ Ik sta vrijwel alleen op de buitenzijde van mijn voet<br>◯ Ik kan mijn voet niet plat op de grond zetten en loop vrijwel alleen op mijn voorvoet

Vragenlijst PRIMA studie 52 weken - VAS Pain score

Number	Question	Answers	
3.1	VAS pijnscore (Geef uw minimaal pijnscore aan tijdens dagelijkse activiteiten op een schaal van 0 tot 100, waarbij 0 geen pijn is en 100 de ergste pijn denkbaar is)	(0.00)	100 (100.00)

Vragenlijst PRIMA studie 52 weken - AAS

Number	Question	Answers

4.1	Wat is het meest bij u van toepassing ten aanzien van uw acitviteit?	☐ American Football<br>☐ Basketbal<br>☐ Gymnastiek/turnen<br>☐ Handbal<br>☐ Rugby<br>☐ Voetbal<br>☐ Hockey<br>☐ Korfbal<br>☐ Gevechtssporten: judo, karate, kung fu, taekwando, jiu jitsu, etc.<br>☐ Oriëntatielopen<br>☐ Rhytmische gymnastiek<br>☐ Volleybal<br>☐ Boxen<br>☐ Freestyle snowboarden<br>☐ Ijshockey<br>☐ Tennis<br>☐ Worstelen<br>☐ Fitness, aerobics<br>☐ Badminton<br>☐ Baseball<br>☐ Cross-country hardlopen<br>☐ Moderne pentathlon<br>☐ Squash<br>☐ Surfen, windsurfen<br>☐ Tafel tennis<br>☐ Atletiek: spring-, werponderdelen<br>☐ Waterskieën<br>☐ Dans<br>☐ Schermen<br>☐ Zaalhockey<br>☐ Bergbeklimmen<br>☐ Langlauf<br>☐ Parachute springen<br>☐ Softball<br>☐ Speciale beroepen en werkactiviteiten; speciale beroepen: ballet, professioneel soldaat, speciale reddingswerker, stuntman, etc.<br>☐ Duiken<br>☐ Scubaduiken<br>☐ Skaten, in-line skaten<br>☐ Atletiek: looponderdelen<br>☐ Triatlon<br>☐ Gewichtsheffen, body-building

- ☐ Alle competitieve sporten hieronder met 'seasonal' conditioning
- ☐ Zwaar fysiek werk
- ☐ Alpine skiën en snowboarden
- ☐ Bowlen/curlen
- ☐ Golf
- ☐ Mountainbike/BMX
- ☐ Powerliften
- ☐ Zeilen
- ☐ Fysiek werk
- ☐ Wielrennen
- ☐ Paardrijden
- ☐ Motorsporten/technische sporten
- ☐ Roeien, kayakken
- ☐ Boogschieten
- ☐ Water polo en zwemmen
- ☐ Kunnen lopen op oneven grond
- ☐ Geen sport, echter geen limitatie in dagelijkse activiteiten
- ☐ Kunnen lopen op even grond, maar de dagelijkste activiteiten zijn gelimiteerd
- ☐ Niet kunnen lopen, fysieke handicap wegens enkelproblemen

4.1.1	***If 'Wat is het meest bij u van toepassing ten aanzien van uw acitviteit?' is equal to 'American Football' answer this question:***<br>American Football	○ Professioneel<br>○ Competitief<br>○ Recreatief
4.1.2	***If 'Wat is het meest bij u van toepassing ten aanzien van uw acitviteit?' is equal to 'Basketbal' answer this question:***<br>Basketbal	○ Professioneel<br>○ Competitief<br>○ Recreatief
4.1.3	***If 'Wat is het meest bij u van toepassing ten aanzien van uw acitviteit?' is equal to 'Gymnastiek/turnen' answer this question:***<br>Gymnastiek/turnen	○ Professioneel<br>○ Competitief<br>○ Recreatief
4.1.4	***If 'Wat is het meest bij u van toepassing ten aanzien van uw acitviteit?' is equal to 'Handbal' answer this question:***<br>Handbal	○ Professioneel<br>○ Competitief<br>○ Recreatief
4.1.5	***If 'Wat is het meest bij u van toepassing ten aanzien van uw acitviteit?' is equal to 'Rugby' answer this question:***<br>Rugby	○ Professioneel<br>○ Competitief<br>○ Recreatief

4.1.6	***If 'Wat is het meest bij u van toepassing ten aanzien van uw acitviteit?' is equal to 'Voetbal' answer this question:*** Voetbal	○ Professioneel ○ Competitief ○ Recreatief
4.1.7	***If 'Wat is het meest bij u van toepassing ten aanzien van uw acitviteit?' is equal to 'Hockey' answer this question:*** Hockey	○ Professioneel ○ Competitief ○ Recreatief
4.1.8	***If 'Wat is het meest bij u van toepassing ten aanzien van uw acitviteit?' is equal to 'Korfbal' answer this question:*** Korfbal	○ Professioneel ○ Competitief ○ Recreatief
4.1.9	***If 'Wat is het meest bij u van toepassing ten aanzien van uw acitviteit?' is equal to 'Gevechtssporten: judo, karate, kung fu, taekwando, jiu jitsu, etc.' answer this question:*** Gevechtssporten: judo, karate, kung fu, taekwando, jiu jitsu, etc.	○ Professioneel ○ Competitief ○ Recreatief
4.1.10	***If 'Wat is het meest bij u van toepassing ten aanzien van uw acitviteit?' is equal to 'Oriëntatielopen' answer this question:*** Oriëntatielopen	○ Professioneel ○ Competitief ○ Recreatief
4.1.11	***If 'Wat is het meest bij u van toepassing ten aanzien van uw acitviteit?' is equal to 'Rhytmische gymnastiek' answer this question:*** Rhytmisch gymnastiek	○ Professioneel ○ Competitief ○ Recreatief
4.1.12	***If 'Wat is het meest bij u van toepassing ten aanzien van uw acitviteit?' is equal to 'Volleybal' answer this question:*** Volleybal	○ Professioneel ○ Competitief ○ Recreatief
4.1.13	***If 'Wat is het meest bij u van toepassing ten aanzien van uw acitviteit?' is equal to 'Boxen' answer this question:*** Boxen	○ Professioneel ○ Competitief ○ Recreatief
4.1.14	***If 'Wat is het meest bij u van toepassing ten aanzien van uw acitviteit?' is equal to 'Freestyle snowboarden' answer this question:*** Freestyle snowboarden	○ Professioneel ○ Competitief ○ Recreatief
4.1.15	***If 'Wat is het meest bij u van toepassing ten aanzien van uw acitviteit?' is equal to 'Freestyle snowboarden' answer this question:*** IJshockey	○ Professioneel ○ Competitief ○ Recreatief

4.1.16	***If 'Wat is het meest bij u van toepassing ten aanzien van uw acitviteit?' is equal to 'Tennis' answer this question:***<br>Tennis	◯ Professioneel<br>◯ Competitief<br>◯ Recreatief
4.1.17	***If 'Wat is het meest bij u van toepassing ten aanzien van uw acitviteit?' is equal to 'Worstelen' answer this question:***<br>Worstelen	◯ Professioneel<br>◯ Competitief<br>◯ Recreatief
4.1.18	***If 'Wat is het meest bij u van toepassing ten aanzien van uw acitviteit?' is equal to 'Fitness, aerobics' answer this question:***<br>Fitness, aerobics	◯ Professioneel<br>◯ Competitief<br>◯ Recreatief
4.1.19	***If 'Wat is het meest bij u van toepassing ten aanzien van uw acitviteit?' is equal to 'Badminton' answer this question:***<br>Badminton	◯ Professioneel<br>◯ Competitief<br>◯ Recreatief
4.1.20	***If 'Wat is het meest bij u van toepassing ten aanzien van uw acitviteit?' is equal to 'Baseball' answer this question:***<br>Baseball	◯ Professioneel<br>◯ Competitief<br>◯ Recreatief
4.1.21	***If 'Wat is het meest bij u van toepassing ten aanzien van uw acitviteit?' is equal to 'Cross-country hardlopen' answer this question:***<br>Cross-country hardlopen	◯ Professioneel<br>◯ Competitief<br>◯ Recreatief
4.1.22	***If 'Wat is het meest bij u van toepassing ten aanzien van uw acitviteit?' is equal to 'Moderne pentathlon' answer this question:***<br>Moderne pentathlon	◯ Professioneel<br>◯ Competitief<br>◯ Recreatief
4.1.23	***If 'Wat is het meest bij u van toepassing ten aanzien van uw acitviteit?' is equal to 'Squash' answer this question:***<br>Squash	◯ Professioneel<br>◯ Competitief<br>◯ Recreatief
4.1.24	***If 'Wat is het meest bij u van toepassing ten aanzien van uw acitviteit?' is equal to 'Surfen, windsurfen' answer this question:***<br>Surfen, windsurfen	◯ Professioneel<br>◯ Competitief<br>◯ Recreatief
4.1.25	***If 'Wat is het meest bij u van toepassing ten aanzien van uw acitviteit?' is equal to 'Tafel tennis' answer this question:***<br>Tafel tennis	◯ Professioneel<br>◯ Competitief<br>◯ Recreatief

4.1.26	***If 'Wat is het meest bij u van toepassing ten aanzien van uw acitviteit?' is equal to 'Atletiek: spring-, werponderdelen' answer this question:*** Atletiek: spring-, werponderdelen	○ Professioneel ○ Competitief ○ Recreatief
4.1.27	***If 'Wat is het meest bij u van toepassing ten aanzien van uw acitviteit?' is equal to 'Waterskieën' answer this question:*** Waterskien	○ Professioneel ○ Competitief ○ Recreatief
4.1.28	***If 'Wat is het meest bij u van toepassing ten aanzien van uw acitviteit?' is equal to 'Dans' answer this question:*** Dans	○ Professioneel ○ Competitief ○ Recreatief
4.1.29	***If 'Wat is het meest bij u van toepassing ten aanzien van uw acitviteit?' is equal to 'Schermen' answer this question:*** Schermen	○ Professioneel ○ Competitief ○ Recreatief
4.1.30	***If 'Wat is het meest bij u van toepassing ten aanzien van uw acitviteit?' is equal to 'Zaalhockey' answer this question:*** Zaalhockey	○ Professioneel ○ Competitief ○ Recreatief
4.1.31	***If 'Wat is het meest bij u van toepassing ten aanzien van uw acitviteit?' is equal to 'Bergbeklimmen' answer this question:*** Bergbeklimmen	○ Professioneel ○ Competitief ○ Recreatief
4.1.32	***If 'Wat is het meest bij u van toepassing ten aanzien van uw acitviteit?' is equal to 'Langlauf' answer this question:*** Langlauf	○ Professioneel ○ Competitief ○ Recreatief
4.1.33	***If 'Wat is het meest bij u van toepassing ten aanzien van uw acitviteit?' is equal to 'Parachute springen' answer this question:*** Parachute springen	○ Professioneel ○ Competitief ○ Recreatief
4.1.34	***If 'Wat is het meest bij u van toepassing ten aanzien van uw acitviteit?' is equal to 'Softball' answer this question:*** Softball	○ Professioneel ○ Competitief ○ Recreatief

4.1.35	***If 'Wat is het meest bij u van toepassing ten aanzien van uw acitviteit?' is equal to 'Speciale beroepen en werkactiviteiten; speciale beroepen: ballet, professioneel soldaat, speciale reddingswerker, stuntman, etc.' answer this question:***<br>Speciale beroepen en werkactiviteiten speciale beroepen: ballet, professioneel soldaat, speciale reddingswerker, stuntman, etc.	◯ ballet, professioneel soldaat, speciale reddingswerker, stuntman, etc.
4.1.36	***If 'Wat is het meest bij u van toepassing ten aanzien van uw acitviteit?' is equal to 'Duiken' answer this question:***<br>Duiken	◯ Professioneel/competitief<br>◯ Recreatief
4.1.37	***If 'Wat is het meest bij u van toepassing ten aanzien van uw acitviteit?' is equal to 'Scubaduiken' answer this question:***<br>Scubaduiken	◯ Professioneel/competitief<br>◯ Recreatief
4.1.38	***If 'Wat is het meest bij u van toepassing ten aanzien van uw acitviteit?' is equal to 'Skaten, in-line skaten' answer this question:***<br>skaten, in-linen skaten	◯ Professioneel/competitief<br>◯ Recreatief
4.1.39	***If 'Wat is het meest bij u van toepassing ten aanzien van uw acitviteit?' is equal to 'Atletiek: looponderdelen' answer this question:***<br>Atletiek: looponderdelen	◯ Professioneel/competitief<br>◯ Recreatief
4.1.40	***If 'Wat is het meest bij u van toepassing ten aanzien van uw acitviteit?' is equal to 'Triatlon' answer this question:***<br>triatlon	◯ Professioneel/competitief<br>◯ Recreatief
4.1.41	***If 'Wat is het meest bij u van toepassing ten aanzien van uw acitviteit?' is equal to 'Gewichtsheffen, body-building' answer this question:***<br>Gewichtsheffen, body-building	◯ Professioneel/competitief<br>◯ Recreatief
4.1.42	***If 'Wat is het meest bij u van toepassing ten aanzien van uw acitviteit?' is equal to 'Alle competitieve sporten hieronder met 'seasonal' conditioning' answer this question:***<br>Alle competitieve sporten hieronder met 'seasonal' conditioning	◯ Alle competitieve sporten hieronder met 'seasonal' conditioning
4.1.43	***If 'Wat is het meest bij u van toepassing ten aanzien van uw acitviteit?' is equal to 'Zwaar fysiek werk' answer this question:***<br>Zwaar fysiek werk	◯ Alle competitieve sporten hieronder met 'seasonal' conditioning

4.1.44	***If 'Wat is het meest bij u van toepassing ten aanzien van uw acitviteit?' is equal to 'Alpine skiën en snowboarden' answer this question:*** Alpine skien en snowboarden	◯ Alpine skien en snowboarden
4.1.45	***If 'Wat is het meest bij u van toepassing ten aanzien van uw acitviteit?' is equal to 'Bowlen/curlen' answer this question:*** Bowlen/curlen	◯ Bowlen/curlen
4.1.46	***If 'Wat is het meest bij u van toepassing ten aanzien van uw acitviteit?' is equal to 'Golf' answer this question:*** Golf	◯ Golf
4.1.47	***If 'Wat is het meest bij u van toepassing ten aanzien van uw acitviteit?' is equal to 'Mountainbike/BMX' answer this question:*** Mountainbike/BMX	◯ Mountainbike/BMX
4.1.48	***If 'Wat is het meest bij u van toepassing ten aanzien van uw acitviteit?' is equal to 'Powerliften' answer this question:*** Powerliften	◯ Powerliften
4.1.49	***If 'Wat is het meest bij u van toepassing ten aanzien van uw acitviteit?' is equal to 'Zeilen' answer this question:*** Zeilen	◯ Zeilen
4.1.50	***If 'Wat is het meest bij u van toepassing ten aanzien van uw acitviteit?' is equal to 'Fysiek werk' answer this question:*** Fysiek werk	◯ Fysiek werk
4.1.51	***If 'Wat is het meest bij u van toepassing ten aanzien van uw acitviteit?' is equal to 'Wielrennen' answer this question:*** Wielrennen	◯ Wielrennen
4.1.52	***If 'Wat is het meest bij u van toepassing ten aanzien van uw acitviteit?' is equal to 'Paardrijden' answer this question:*** Paardrijden	◯ Paardrijden
4.1.53	***If 'Wat is het meest bij u van toepassing ten aanzien van uw acitviteit?' is equal to 'Motorsporten/technische sporten' answer this question:*** Motorsporten/technische sporten	◯ Motorsporten/technische sporten
4.1.54	***If 'Wat is het meest bij u van toepassing ten aanzien van uw acitviteit?' is equal to 'Roeien, kayakken' answer this question:*** Roeien, kayakken	◯ Roeien, kayakken

4.1.55	***If 'Wat is het meest bij u van toepassing ten aanzien van uw acitviteit?' is equal to 'Boogschieten' answer this question:*** Boogschieten	◯ Boogschieten
4.1.56	***If 'Wat is het meest bij u van toepassing ten aanzien van uw acitviteit?' is equal to 'Water polo en zwemmen' answer this question:*** Water polo en zwemmen	◯ Water polo en zwemmen
4.1.57	***If 'Wat is het meest bij u van toepassing ten aanzien van uw acitviteit?' is equal to 'Kunnen lopen op oneven grond' answer this question:*** Kunnen lopen op oneven grond	◯ Kunnen lopen op oneven grond
4.1.58	***If 'Wat is het meest bij u van toepassing ten aanzien van uw acitviteit?' is equal to 'Geen sport, echter geen limitatie in dagelijkse activiteiten' answer this question:*** Geen sport, echter geen limitatie in dagelijkse activiteiten	◯ Geen sport, echter geen limitatie in dagelijkse activiteiten
4.1.59	***If 'Wat is het meest bij u van toepassing ten aanzien van uw acitviteit?' is equal to 'Kunnen lopen op even grond, maar de dagelijkste activiteiten zijn gelimiteerd' answer this question:*** Kunnen lopen op even grond, maar de dagelijkste activiteiten zijn gelimiteerd	◯ Kunnen lopen op even grond, maar de dagelijkste activiteiten zijn gelimiteerd
4.1.60	***If 'Wat is het meest bij u van toepassing ten aanzien van uw acitviteit?' is equal to 'Niet kunnen lopen, fysieke handicap wegens enkelproblemen' answer this question:*** Niet kunnen lopen, fysieke handicap wegens enkelproblemen	◯ Niet kunnen lopen, fysieke handicap wegens enkelproblemen

Vragenlijst PRIMA studie 52 weken - Hoe tevreden bent u over de enkelklachten?

Number	Question	Answers
5.1	Hoe tevreden bent u over de enkelklachten?	◯ Slecht<br>◯ Matig<br>◯ Goed<br>◯ Uitstekend

Vragenlijst PRIMA studie 52 weken - SF-36

Number	Question	Answers
	Deze vragenlijst gaat over uw standpunten t.a.v. uw gezondheid. Met behulp van deze gegevens kan worden bijgehouden hoe u zich voelt en hoe goed u in staat bent uw gebruikelijke bezigheden uit te voeren. Beantwoord elke vraag door een antwoord aan te klikken. Als u niet zeker weet hoe u een vraag moet beantwoorden, geef dan het best mogelijke antwoord.	
6.1	Hoe zou u over het algemeen uw gezondheid noemen?	○ Uitstekend ○ Zeer goed ○ Goed ○ Matig ○ Slecht
6.2	Hoe beoordeelt u nu uw gezondheid over het algemeen vergeleken met een jaar geleden?	○ Veel beter dan een jaar geleden ○ Wat beter dan een jaar geleden ○ Ongeveer hetzelfde als een jaar geleden ○ Wat slechter dan een jaar geleden ○ Veel slechter dan een jaar geleden
6.3	Wordt u door uw gezondheid op dit moment beperkt bij forse inspanning, zoals hardlopen, tillen van zware voorwerpen of een veeleisende sport beoefenen?	○ Ja, ernstig beperkt ○ Ja, een beetje beperkt ○ Nee, helemaal niet beperkt
6.4	Wordt u door uw gezondheid op dit moment beperkt bij matige inspanning zoals een tafel verplaatsen, stofzuigen, zwemmen of fietsen?	○ Ja, ernstig beperkt ○ Ja, een beetje beperkt ○ Nee, helemaal niet beperkt
6.5	Wordt u door uw gezondheid op dit moment beperkt bij boodschappen tillen of dragen?	○ Ja, ernstig beperkt ○ Ja, een beetje beperkt ○ Nee, helemaal niet beperkt
6.6	Wordt u door uw gezondheid op dit moment beperkt bij een paar trappen oplopen?	○ Ja, ernstig beperkt ○ Ja, een beetje beperkt ○ Nee, helemaal niet beperkt
6.7	Wordt u door uw gezondheid op dit moment beperkt bij één trap oplopen?	○ Ja, ernstig beperkt ○ Ja, een beetje beperkt ○ Nee, helemaal niet beperkt
6.8	Wordt u door uw gezondheid op dit moment beperkt bij bukken, knielen of hurken?	○ Ja, ernstig beperkt ○ Ja, een beetje beperkt ○ Nee, helemaal niet beperkt
6.9	Wordt u door uw gezondheid op dit moment beperkt bij meer dan één kilometer lopen?	○ Ja, ernstig beperkt ○ Ja, een beetje beperkt ○ Nee, helemaal niet beperkt
6.10	Wordt u door uw gezondheid op dit moment beperkt bij een paar honderd meter lopen?	○ Ja, ernstig beperkt ○ Ja, een beetje beperkt ○ Nee, helemaal niet beperkt

For peer review only - http://bmjopen.bmj.com/site/about/guidelines.xhtml

6.11	Wordt u door uw gezondheid op dit moment beperkt bij ongeveer honderd meter lopen?	◯ Ja, ernstig beperkt ◯ Ja, een beetje beperkt ◯ Nee, helemaal niet beperkt
6.12	Wordt u door uw gezondheid op dit moment beperkt bij uzelf wassen of aankleden?	◯ Ja, ernstig beperkt ◯ Ja, een beetje beperkt ◯ Nee, helemaal niet beperkt
6.13	U besteedde in de afgelopen 4 weken minder tijd aan werk of andere bezigheden	◯ Ja ◯ Nee
6.14	U heeft in de afgelopen 4 weken minder bereikt dan u zou willen	◯ Ja ◯ Nee
6.15	U was in de afgelopen 4 weken beperkt in het soort werk of andere bezigheden.	◯ Ja ◯ Nee
6.16	U had de afgelopen 4 weken moeite om uw werk of andere bezigheden uit te voeren (het kostte u bijvoorbeeld extra inspanning).	◯ Ja ◯ Nee
6.17	U besteedde in de afgelopen 4 weken minder tijd aan werk of andere bezigheden ten gevolge van emotionele problemen	◯ Ja ◯ Nee
6.18	U heeft in de afgelopen 4 weken minder bereikt dan u zou willen ten gevolge van emotionele problemen.	◯ Ja ◯ Nee
6.19	U deed de afgelopen 4 weken uw werk of andere bezigheden niet zo zorgvuldig als gewoonlijk ten gevolge van emotionele problemen.	◯ Ja ◯ Nee
6.20	In hoeverre hebben uw lichamelijke gezondheid of emotionele problemen u gedurende de afgelopen 4 weken gehinderd in uw normale omgang met familie, vrienden of buren, of bij activiteiten in groepsverband?	◯ Helemaal niet ◯ Enigszins ◯ Nogal ◯ Veel ◯ Heel erg veel
6.21	Hoeveel lichamelijke pijn heeft u de afgelopen 4 weken gehad?	◯ Geen<br>◯ Heel licht<br>◯ Licht<br>◯ Nogal<br>◯ Ernstig<br>◯ Heel ernstig
6.22	In welke mate bent u de afgelopen 4 weken door de pijn gehinderd in uw normale werk (zowel werk buitenshuis als huishoudelijk werk)?	◯ Helemaal niet<br>◯ Een klein beetje<br>◯ Nogal<br>◯ Veel<br>◯ Heel erg veel

6.23	Voelde u zich levenslustig?	◯ Altijd<br>◯ Meestal<br>◯ Vaak<br>◯ Soms<br>◯ Zelden<br>◯ Nooit
6.24	Was u erg zenuwachtig?	◯ Altijd<br>◯ Meestal<br>◯ Vaak<br>◯ Soms<br>◯ Zelden<br>◯ Nooit
6.25	Zat u zo in de put dat u niets kon?	◯ Altijd<br>◯ Meestal<br>◯ Vaak<br>◯ Soms<br>◯ Zelden<br>◯ Nooit
6.26	Voelde u zich rustig en tevreden?	◯ Altijd<br>◯ Meestal<br>◯ Vaak<br>◯ Soms<br>◯ Zelden<br>◯ Nooit
6.27	Had u veel energie?	◯ Altijd<br>◯ Meestal<br>◯ Vaak<br>◯ Soms<br>◯ Zelden<br>◯ Nooit
6.28	Voelde u zich somber en neerslachtig?	◯ Altijd<br>◯ Meestal<br>◯ Vaak<br>◯ Soms<br>◯ Zelden<br>◯ Nooit

6.29	Voelde u zich uitgeput?	◯ Altijd<br>◯ Meestal<br>◯ Vaak<br>◯ Soms<br>◯ Zelden<br>◯ Nooit
6.30	Was u een gelukkig mens?	◯ Altijd<br>◯ Meestal<br>◯ Vaak<br>◯ Soms<br>◯ Zelden<br>◯ Nooit
6.31	Voelde u zich moe?	◯ Altijd<br>◯ Meestal<br>◯ Vaak<br>◯ Soms<br>◯ Zelden<br>◯ Nooit
6.32	Hoe vaak hebben uw lichamelijke gezondheid of emotionele problemen u gedurende de afgelopen 4 weken gehinderd bij uw sociale activiteiten (zoals vrienden of familie bezoeken etc.)?	◯ Altijd<br>◯ Meestal<br>◯ Soms<br>◯ Zelden<br>◯ Nooit
6.33	Ik lijk wat gemakkelijker ziek te worden dan andere mensen	◯ Volkomen juist<br>◯ Grotendeels juist<br>◯ Weet ik niet<br>◯ Grotendeels onjuist<br>◯ Volkomen onjuist
6.34	Ik ben even gezond als andere mensen die ik ken	◯ Volkomen juist<br>◯ Grotendeels juist<br>◯ Weet ik niet<br>◯ Grotendeels onjuist<br>◯ Volkomen onjuist

6.35	Ik verwacht dat mijn gezondheid achteruit zal gaan	◯ Volkomen juist<br>◯ Grotendeels juist<br>◯ Weet ik niet<br>◯ Grotendeels onjuist<br>◯ Volkomen onjuist
6.36	Mijn gezondheid is uitstekend	◯ Volkomen juist<br>◯ Grotendeels juist<br>◯ Weet ik niet<br>◯ Grotendeels onjuist<br>◯ Volkomen onjuist

Vragenlijst PRIMA studie 52 weken - GAS

Number	Question	Answers
	Tijdens de eerste afspraak werd een duidelijk doel met u afgesproken. Dit werd tevens per email naar u gestuurd. Hoe is de situatie nu ten aanzien van de destijds gestelde doelstelling?	
7.1	Wat is nu de situatie ten aanzien van het tijdens de eerste afspraak afgesproken doelstelling (Goal attainment Scaling)?	◯ Achteruitgang (minder dan de utigangssituatie)<br>◯ Uitgangssituatie<br>◯ Minder dan het doel<br>◯ Doel<br>◯ Meer dan het doel<br>◯ Veel meer dan het doel

Vragenlijst PRIMA studie 52 weken - EQ-5D-3L

Number	Question	Answers
	Deze vragenlijst gaat over uw standpunten t.a.v. uw gezondheid. Met behulp van deze gegevens kan worden bijgehouden hoe u zich voelt en hoe goed u in staat bent uw gebruikelijke bezigheden uit te voeren. Beantwoord elke vraag door een antwoord aan te klikken. Als u niet zeker weet hoe u een vraag moet beantwoorden, geef dan het best mogelijke antwoord.	
8.1	Hoe is het met uw Mobiliteit gesteld?	◯ Ik heb geen problemen met lopen<br>◯ Ik heb enige problemen met lopen<br>◯ Ik ben bedlegerig

8.2	Hoe is het met uw Zelfzorg gesteld?	◯ Ik heb geen problemen om mijzelf te wassen of aan te kleden<br>◯ Ik heb enige problemen om mijzelf te wassen of aan te kleden<br>◯ Ik ben niet in staat om mijzelf te wassen of aan te kleden
8.3	Hoe is het met de Dagelijkse activiteiten (werk, studie, huishouden, gezins- en vrijetijdsactiviteiten) gesteld?	◯ Ik heb geen problemen met mijn dagelijkse activiteiten<br>◯ Ik heb enige problemen met mijn dagelijkse activiteiten<br>◯ Ik ben niet in staat om mijn dagelijkse activiteiten uit te voeren
8.4	Hoe is het met de Pijn/klachten gesteld?	◯ Ik heb geen pijn of andere klachten<br>◯ Ik heb matige pijn of andere klachten<br>◯ Ik heb zeer ernstige pijn of andere klachten
8.5	Hoe is het met de Stemming gesteld?	◯ Ik ben niet angstig of somber<br>◯ Ik ben matig angstig of somber<br>◯ Ik ben erg angstig of somber
	We willen weten hoe goed of slecht uw gezondheid VANDAAG is. Deze meetschaal loopt van 0 tot 100: 100 staat voor de BESTE gezondheid die u zich kunt voorstellen - 0 staat voor de SLECHTSTE gezondheid die u zich kunt voorstellen	
8.6	Uw Gezondheid vandaag	(0.00) (100.00)

Vragenlijst PRIMA studie 52 weken - AOS

Number	Question	Answers
	Instructies: De lijn naast elke vraag staat voor hoeveel PIJN u heeft in verschillende situaties. De linker kant (0) is “geen pijn” en de rechter kant (100) is “ergste pijn denkbaar”. Geef voor de onderstaande situaties op de lijn aan hoeveel PIJN u in de afgelopen week in de enkel had. Als een of meerdere van deze situaties niet van toepassing waren, dan kiest u de "niet van toepassing" optie.	
9.1	Wat was de hoogte van de ergste pijn in de afgelopen week?	(0.00) (100.00)
9.2	Heeft u pijn als u 's ochtends opstaat?	◯ Ja<br>◯ Nee<br>◯ Niet van toepassing

9.2.1	***If 'Heeft u pijn als u 's ochtends opstaat?' is equal to 'Ja' answer this question:***<br>Hoeveel pijn heeft u voordat u 's ochtends opstaat?	(0.00)	(100.00)
9.3	Heeft u pijn wanneer u op blote voeten loopt?	◯ Ja<br>◯ Nee<br>◯ Niet van toepassing	
9.3.1	***If 'Heeft u pijn wanneer u op blote voeten loopt?' is equal to 'Ja' answer this question:***<br>Hoeveel pijn heeft u als u op blote voeten loopt?	(0.00)	(100.00)
9.4	Heeft u pijn wanneer u op blote voeten staat?	◯ Ja<br>◯ Nee<br>◯ Niet van toepassing	
9.4.1	***If 'Heeft u pijn wanneer u op blote voeten staat?' is equal to 'Ja' answer this question:***<br>Hoeveel pijn heeft u als u op blote voeten staat?	(0.00)	(100.00)
9.5	Heeft u pijn wanneer u met schoenen loopt?	◯ Ja<br>◯ Nee<br>◯ Niet van toepassing	
9.5.1	***If 'Heeft u pijn wanneer u met schoenen loopt?' is equal to 'Ja' answer this question:***<br>Hoeveel pijn heeft u wanneer u met schoenen loopt?	(0.00)	(100.00)
9.6	Heeft u pijn wanneer u met schoenen staat?	◯ Ja<br>◯ Nee<br>◯ Niet van toepassing	
9.6.1	***If 'Heeft u pijn wanneer u met schoenen staat?' is equal to 'Ja' answer this question:***<br>Hoeveel pijn heeft u wanneer u met schoenen staat?	(0.00)	(100.00)
9.7	Heeft u pijn wanneer u loopt met steunzolen of een brace?	◯ Ja<br>◯ Nee<br>◯ Niet van toepassing	
9.7.1	***If 'Heeft u pijn wanneer u loopt met steunzolen of een brace?' is equal to 'Ja' answer this question:***<br>Hoeveel pijn heeft u wanneer u loopt met steunzolen of een brace?	(0.00)	(100.00)

For peer review only - http://bmjopen.bmj.com/site/about/guidelines.xhtml

9.8	Heeft u pijn wanneer u staat met steunzolen of een brace?	◯ Ja<br>◯ Nee<br>◯ Niet van toepassing
9.8.1	***If 'Heeft u pijn wanneer u staat met steunzolen of een brace?' is equal to 'Ja' answer this question:***<br>Hoeveel pijn heeft u wanneer u staat met steunzolen of een brace?	(0.00) (100.00)
9.9	Heeft u pijn aan het einde van de dag?	◯ Ja<br>◯ Nee<br>◯ Niet van toepassing
9.9.1	***If 'Heeft u pijn aan het einde van de dag?' is equal to 'Ja' answer this question:***<br>Hoeveel pijn heeft u aan het einde van de dag?	(0.00) (100.00)
	Instructies:De lijn naast elke vraag staat voor hoeveel MOEITE u heeft met verschillende activiteiten. De linker kant (0) is “Niet moeilijk” en de rechter kant (100) is “Te moeilijk, niet uitvoerbaar”. Geef voor de onderstaande activiteiten op de lijn aan hoeveel MOEITE u in de afgelopen week door enkelklachten had met onderstaande activiteiten. Als een situatie niet van toepassing is, kies dan de optie "niet van toepassing".	
9.10	Heeft u moeite met door het huis lopen?	◯ Ja<br>◯ Nee<br>◯ Niet van toepassing
9.10.1	***If 'Heeft u moeite met door het huis lopen?' is equal to 'Ja' answer this question:***<br>Hoeveel moeite heeft u met door het huis lopen?	(0.00) (100.00)
9.11	Heeft u moeite met buiten lopen op oneven ondergrond?	◯ Ja<br>◯ Nee<br>◯ Niet van toepassing
9.11.1	***If 'Heeft u moeite met buiten lopen op oneven ondergrond?' is equal to 'Ja' answer this question:***<br>Hoeveel moeite heeft u met buiten lopen op oneven ondergrond?	(0.00) (100.00)
9.12	Heeft u moeite met een paar honderd meter lopen?	◯ Ja<br>◯ Nee<br>◯ Niet van toepassing
9.12.1	***If 'Heeft u moeite met een paar honderd meter lopen?' is equal to 'Ja' answer this question:***<br>Hoeveel moeite heeft u met een paar honderd meter lopen?	(0.00) (100.00)

9.13	Heeft u moeite met een trap oplopen?	◯ Ja<br>◯ Nee<br>◯ Niet van toepassing	
9.13.1	***If 'Heeft u moeite met een trap oplopen?' is equal to 'Ja' answer this question:***<br>Hoeveel moeite heeft u met een trap oplopen?	(0.00)	(100.00)
9.14	Heeft u moeite met een trap aflopen?	◯ Ja<br>◯ Nee<br>◯ Niet van toepassing	
9.14.1	***If 'Heeft u moeite met een trap aflopen?' is equal to 'Ja' answer this question:***<br>Hoeveel moeite heeft u met een trap aflopen?	(0.00)	(100.00)
9.15	Heeft u moeite met op de tenen staan?	◯ Ja<br>◯ Nee<br>◯ Niet van toepassing	
9.15.1	***If 'Heeft u moeite met op de tenen staan?' is equal to 'Ja' answer this question:***<br>Hoeveel moeite heeft u met op de tenen staan?	(0.00)	(100.00)
9.16	Heeft u moeite met opstaan uit de stoel?	◯ Ja<br>◯ Nee<br>◯ Niet van toepassing	
9.16.1	***If 'Heeft u moeite met opstaan uit de stoel?' is equal to 'Ja' answer this question:***<br>Hoeveel moeite heeft u met opstaan uit de stoel?	(0.00)	(100.00)
9.17	Heeft u moeite met het op- of afstappen van stoepranden?	◯ Ja<br>◯ Nee<br>◯ Niet van toepassing	
9.17.1	***If 'Heeft u moeite met het op- of afstappen van stoepranden?' is equal to 'Ja' answer this question:***<br>Hoeveel moeite heeft u met het op- of afstappen van stoepranden?	(0.00)	(100.00)
9.18	Heeft u moeite met snel lopen of rennen?	◯ Ja<br>◯ Nee<br>◯ Niet van toepassing	

9.18.1	***If 'Heeft u moeite met snel lopen of rennen?' is equal to 'Ja' answer this question:*** Hoeveel moeite heeft u met snel lopen of rennen?	(0.00)	(100.00)

Vragenlijst PRIMA studie 52 weken - FAOS

Number	Question	Answers
	Deze lijst vraagt naar uw mening over uw voet/enkel. Uw antwoorden geven ons een beeld van uw voet/enkel klachten en hoe u in staat bent om alledaagse activiteiten uit te voeren in uw huidige situatie. Beantwoorden van een vraag doet u door het aanklikken van een vakje met het volgens u meest juiste antwoord (één vakje per vraag). Als u niet zeker weet hoe u een vraag moet beantwoorden, geeft u dan het antwoord dat volgens u het meest op uw situatie van toepassing is. Deze vraag heeft betrekking op het voorkomen van voet/enkel klachten in de afgelopen week.	
10.1	Is uw voet/enkel gezwollen?	◯ Nooit ◯ Zelden ◯ Soms ◯ Vaak ◯ Altijd
10.2	Voelt u gekraak of hoort u klikken of een ander vreemd geluid wanneer u de voet/enkel beweegt?	◯ Nooit ◯ Zelden ◯ Soms ◯ Vaak ◯ Altijd
10.3	Hapert uw enkel of blokkeert uw enkel ('op slot' gaan zitten) wanneer u deze beweegt?	◯ Nooit ◯ Zelden ◯ Soms ◯ Vaak ◯ Altijd
10.4	Kunt u de voet/enkel volledig strekken?	◯ Altijd ◯ Vaak ◯ Soms ◯ Zelden ◯ Nooit
10.5	Kunt u de voet volledig naar u toe buigen?	◯ Altijd ◯ Vaak ◯ Soms ◯ Zelden ◯ Nooit
10.6	In welke mate heeft u een stijf gevoel in de voet/enkel 's ochtends bij het wakker worden?	◯ Niet ◯ Mild ◯ Matig ◯ Ernstig ◯ Zeer ernstig
10.7	In welke mate heeft u een stijf gevoel in de voet/enkel na zitten, liggen of rusten later op de dag?	◯ Niet ◯ Mild ◯ Matig ◯ Ernstig ◯ Zeer ernstig
10.8	Hoe vaak heeft u pijn in uw voet/enkel?	◯ Nooit ◯ Maandelijks ◯ Wekelijks ◯ Dagelijks ◯ Altijd

10.9	Hoeveel voet/enkel pijn heeft u gehad in de afgelopen week bij draaien als uw voet/enkel op de grond staat?	◯ Geen<br>◯ Mild<br>◯ Matig<br>◯ Ernstig<br>◯ Zeer ernstig
10.10	Hoeveel voet/enkel pijn heeft u gehad in de afgelopen week bij het volledig uitstrekken van de voet/enkel?	◯ Geen<br>◯ Mild<br>◯ Matig<br>◯ Ernstig<br>◯ Zeer ernstig
10.11	Hoeveel voet/enkel pijn heeft u gehad in de afgelopen week bij het volledig naar u toe buigen/optrekken van de voet/enkel	◯ Geen<br>◯ Mild<br>◯ Matig<br>◯ Ernstig<br>◯ Zeer ernstig
10.12	Hoeveel voet/enkel pijn heeft u gehad in de afgelopen week bij het lopen op een vlakke ondergrond	◯ Geen<br>◯ Mild<br>◯ Matig<br>◯ Ernstig<br>◯ Zeer ernstig
10.13	Hoeveel voet/enkel pijn heeft u gehad in de afgelopen week bij het trap op- en trap af lopen?	◯ Geen<br>◯ Mild<br>◯ Matig<br>◯ Ernstig<br>◯ Zeer ernstig
10.14	Hoeveel voet/enkel pijn heeft u gehad in de afgelopen week 's nachts in bed?	◯ Geen<br>◯ Mild<br>◯ Matig<br>◯ Ernstig<br>◯ Zeer ernstig
10.15	Hoeveel voet/enkel pijn heeft u gehad in de afgelopen week bij het zitten of liggen?	◯ Geen<br>◯ Mild<br>◯ Matig<br>◯ Ernstig<br>◯ Zeer ernstig

For peer review only - http://bmjopen.bmj.com/site/about/guidelines.xhtml

10.16	Hoeveel voet/enkel pijn heeft u gehad in de afgelopen week bij het rechtop staan?	◯ Geen<br>◯ Mild<br>◯ Matig<br>◯ Ernstig<br>◯ Zeer ernstig
10.17	In welke mate werd u gehinderd bij het trap aflopen?	◯ Niet ◯ Mild ◯ Matig ◯ Ernstig ◯ Zeer ernstig
10.18	In welke mate werd u gehinderd bij het trap op lopen?	◯ Niet ◯ Mild ◯ Matig ◯ Ernstig ◯ Zeer ernstig
10.19	In welke mate werd u gehinderd als u vanuit een zittende positie ging staan?	◯ Niet ◯ Mild ◯ Matig ◯ Ernstig ◯ Zeer ernstig
10.20	In welke mate werd u gehinderd bij het staan?	◯ Niet ◯ Mild ◯ Matig ◯ Ernstig ◯ Zeer ernstig
10.21	In welke mate werd u gehinderd bij het naar de grond buigen/iets oprapen?	◯ Niet ◯ Mild ◯ Matig ◯ Ernstig ◯ Zeer ernstig
10.22	In welke mate werd u gehinderd bij het lopen op een vlakke ondergrond?	◯ Niet ◯ Mild ◯ Matig ◯ Ernstig ◯ Zeer ernstig
10.23	In welke mate werd u gehinderd bij het in- en uit de auto stappen?	◯ Niet ◯ Mild ◯ Matig ◯ Ernstig ◯ Zeer ernstig
10.24	In welke mate werd u gehinderd bij het boodschappen doen?	◯ Niet ◯ Mild ◯ Matig ◯ Ernstig ◯ Zeer ernstig
10.25	In welke mate werd u gehinderd bij sokken/panty's aantrekken?	◯ Niet ◯ Mild ◯ Matig ◯ Ernstig ◯ Zeer ernstig
10.26	In welke mate werd u gehinderd bij het opstaan uit bed?	◯ Niet ◯ Mild ◯ Matig ◯ Ernstig ◯ Zeer ernstig
10.27	In welke mate werd u gehinderd bij het sokken uittrekken?	◯ Niet ◯ Mild ◯ Matig ◯ Ernstig ◯ Zeer ernstig
10.28	In welke mate werd u gehinderd bij het in bed liggen (omdraaien, lange tijd uw voet/enkel in dezelfde positie houden)?	◯ Niet ◯ Mild ◯ Matig ◯ Ernstig ◯ Zeer ernstig

10.29	In welke mate werd u gehinderd bij het in/uit bad stappen?	◯ Niet ◯ Mild ◯ Matig ◯ Ernstig ◯ Zeer ernstig
10.30	In welke mate werd u gehinderd bij zitten?	◯ Niet ◯ Mild ◯ Matig ◯ Ernstig ◯ Zeer ernstig
10.31	In welke mate werd u gehinderd bij het toilet op en af gaan?	◯ Niet ◯ Mild ◯ Matig ◯ Ernstig ◯ Zeer ernstig
10.32	In welke mate werd u gehinderd bij zwaar huishoudelijk werk (bijvoorbeeld zware dozen sjouwen, vloer schrobben)?	◯ Niet ◯ Mild ◯ Matig ◯ Ernstig ◯ Zeer ernstig
10.33	In welke mate werd u gehinderd bij licht huishoudeijk werk (bijvoorbeeld koken, afstoffen)?	◯ Niet ◯ Mild ◯ Matig ◯ Ernstig ◯ Zeer ernstig
10.34	In welke mate werd u gehinderd bij hurken?	◯ Niet ◯ Mild ◯ Matig ◯ Ernstig ◯ Zeer ernstig
10.35	In welke mate werd u gehinderd bij hardlopen?	◯ Niet ◯ Mild ◯ Matig ◯ Ernstig ◯ Zeer ernstig
10.36	In welke mate werd u gehinderd bij springen?	◯ Niet ◯ Mild ◯ Matig ◯ Ernstig ◯ Zeer ernstig
10.37	In welke mate werd u gehinderd bij ronddraaien op uw aangedane voet/enkel?	◯ Niet ◯ Mild ◯ Matig ◯ Ernstig ◯ Zeer ernstig
10.38	In welke mate werd u gehinderd bij knielen?	◯ Niet ◯ Mild ◯ Matig ◯ Ernstig ◯ Zeer ernstig
10.39	Hoe vaak bent u zich bewust van uw voet/enkel probleem?	◯ Nooit ◯ Maandelijks ◯ Wekelijks ◯ Dagelijks ◯ Altijd
10.40	Heeft u uw leven veranderd om activiteiten te vermijden die schadelijk kunnen zijn voor uw voet/enkel?	◯ Niet<br>◯ Enigszins<br>◯ Matig<br>◯ Behoorlijk<br>◯ Volledig

10.41	In hoeverre kunt u op uw voet/enkel vertrouwen?	◯ Volledig<br>◯ Behoorlijk<br>◯ Matig<br>◯ Enigzins<br>◯ Niet
10.42	In het algemeen, in welke mate ondervindt u hinder van uw voet/enkel	◯ Geen<br>◯ Mild<br>◯ Matig<br>◯ Ernstig<br>◯ Zeer ernstig

Vragenlijst PRIMA studie 52 weken - PRODISQ - Gezondheid en werk

Number	Question	Answers
11.1	Wat is uw leeftijd?	[ ] jaar
11.2	Wat is uw geslacht?	◯ Man<br>◯ Vrouw
11.3	Wat is de hoogste opleiding die u heeft afgemaakt (Zoek uw hoogste opleiding en kruis het hokje daarvoor aan)?	☐ Ik heb geen school of opleiding afgemaakt<br>☐ Lagere school of basisschool<br>☐ Huishoudschool, vbo, lbo, lts, leao of lhno<br>☐ Mavo, mulo, ivo of vmbo<br>☐ Mbo, mts, meao, mhno, inas of intas<br>☐ Havo, vwo, hbs, mms, atheneum of gymnasium<br>☐ Hbo, hts, heao of hhno<br>☐ Universiteit<br>☐ Ik heb een andere opleiding afgemaakt, namelijk
11.3.1	***If 'Wat is de hoogste opleiding die u heeft afgemaakt (Zoek uw hoogste opleiding en kruis het hokje daarvoor aan)?' is equal to 'Ik heb een andere opleiding afgemaakt, namelijk' answer this question:***<br>Wat is de hoogste opleiding die u heeft afgemaakt?	[ ]

11.4	Wat doet u in het dagelijks leven?	☐ Ik zit op school, ik studeer<br>☐ Ik werk in loondienst<br>☐ Ik ben zelfstandig ondernemer<br>☐ Ik ben huisvrouw, huisman<br>☐ Ik ben werkloos<br>☐ Ik ben arbeidsongeschikt<br>☐ Ik ben met pensioen of prepensioen<br>☐ Ik doe iets anders, namelijk
11.4.1	***If 'Wat doet u in het dagelijks leven?' is equal to 'Ik ben arbeidsongeschikt' answer this question:***<br>Wat doet u in het dagelijks leven?	
11.4.2	***If 'Wat doet u in het dagelijks leven?' is equal to 'Ik doe iets anders, namelijk' answer this question:***<br>Wat doet u in het dagelijks leven?	
11.5	Hebt u betaald werk?	○ Nee<br>○ Ja
11.5.1	***If 'Hebt u betaald werk?' is equal to 'Ja' answer this question:***<br>Wat is uw beroep?	
11.5.2	***If 'Hebt u betaald werk?' is equal to 'Ja' answer this question:***<br>Hoeveel uur per week werkt u (Tel alleen de uren waarvoor u betaald wordt)?	uren
11.5.3	***If 'Hebt u betaald werk?' is equal to 'Ja' answer this question:***<br>Hoeveel dagen in de week werkt u?	Dagen
11.5.4	***If 'Hebt u betaald werk?' is equal to 'Ja' answer this question:***<br>Bent u in de afgelopen 4 weken afwezig geweest van uw werk omdat u ziek was?	○ Nee<br>○ Ja
11.5.4.1	***If 'Bent u in de afgelopen 4 weken afwezig geweest van uw werk omdat u ziek was?' is equal to 'Ja' answer this question:***<br>Bent u in de afgelopen 4 weken afwezig geweest van uw werk omdat u ziek was (Tel alleen de werkdagen in de afgelopen 4 weken)?	dagen afwezig geweest
11.5.4.2	***If 'Bent u in de afgelopen 4 weken afwezig geweest van uw werk omdat u ziek was?' is equal to 'Ja' answer this question:***<br>Was u langer dan de gehele periode van 4 weken afwezig van uw werk doordat u ziek was?	○ Nee<br>○ Ja

11.5.4.2.1	***If 'Was u langer dan de gehele periode van 4 weken afwezig van uw werk doordat u ziek was?' is equal to 'Ja' answer this question:***<br>Wanneer heeft u zich ziek gemeld?	☐ ☐ ☐ *(dd-mm-yyyy)*
11.5.5	***If 'Hebt u betaald werk?' is equal to 'Ja' answer this question:***<br>Waren er in de afgelopen 4 weken, dagen waarop u wel gewerkt heeft, maar tijdens uw werk last had van lichamelijke of psychische problemen?	○ Ja<br>○ Nee
11.5.5.1	***If 'Waren er in de afgelopen 4 weken, dagen waarop u wel gewerkt heeft, maar tijdens uw werk last had van lichamelijke of psychische problemen?' is equal to 'Ja' answer this question:***<br>Op hoeveel werkdagen had u tijdens uw werk last van uw lichamelijke of psychische problemen?	☐ werkdagen
11.5.5.2	***If 'Waren er in de afgelopen 4 weken, dagen waarop u wel gewerkt heeft, maar tijdens uw werk last had van lichamelijke of psychische problemen?' is equal to 'Ja' answer this question:***<br>Op de dagen dat u last had, kon u misschien niet zoveel werk doen als normaal. Hoeveel werk kon u op deze dagen gemiddeld doen?	Ik kon op deze dagen niks doen (0.00) — Ik kon net zoveel doen als normaal (10.00)
11.6	Waren er dagen waarop u minder onbetaald werk kon doen door uw lichamelijke of psychische problemen?	○ Nee<br>○ Ja
11.6.1	***If 'Waren er dagen waarop u minder onbetaald werk kon doen door uw lichamelijke of psychische problemen?' is equal to 'Ja' answer this question:***<br>Op hoeveel dagen was dit zo?	☐ Dagen
11.6.2	***If 'Waren er dagen waarop u minder onbetaald werk kon doen door uw lichamelijke of psychische problemen?' is equal to 'Ja' answer this question:***<br>Stel dat iemand, bijvoorbeeld uw partner, familielid of een bekende, u op deze dagen had geholpen. En al het onbetaalde werk wat u niet kon doen, voor u had gedaan. Hoeveel uur was die persoon hier op deze dagen dan gemiddeld mee bezig geweest?	☐ uur op deze dagen

Vragenlijst PRIMA studie 52 weken - PRODISQ - Zorggebruik

Number	Question	Answers
	Wij willen graag weten met welke dokters u in de afgelopen 3 maanden een afspraak had. Het gaat om afspraken voor uzelf. Ook andere zorgverleners tellen mee. Bijvoorbeeld de fysiotherapeut.	

12.1	Bent u in de afgelopen 3 maanden naar uw huisarts geweest?	◯ Nee<br>◯ Ja
12.1.1	***If 'Bent u in de afgelopen 3 maanden naar uw huisarts geweest?' is equal to 'Ja' answer this question:***<br>Hoeveel afspraken had u?	[ ] afspraken
12.2	Bent u in de afgelopen 3 maanden in contact geweest met een maatschappelijk werker?	◯ Nee<br>◯ Ja
12.2.1	***If 'Bent u in de afgelopen 3 maanden in contact geweest met een maatschappelijk werker?' is equal to 'Ja' answer this question:***<br>Hoeveel afspraken had u?	[ ] afspraken
12.3	Bent u in de afgelopen 3 maanden naar een fysiotherapeut geweest? Of een caesartherapeut, therapeut mensendieck of een manueel therapeut?	◯ Nee<br>◯ Ja
12.3.1	***If 'Bent u in de afgelopen 3 maanden naar een fysiotherapeut geweest? Of een caesartherapeut, therapeut mensendieck of een manueel therapeut?' is equal to 'Ja' answer this question:***<br>Hoeveel afspraken had u?	[ ] afspraken
12.4	Bent u in de afgelopen 3 maanden naar een ergotherapeut geweest?	◯ Nee<br>◯ Ja
12.4.1	***If 'Bent u in de afgelopen 3 maanden naar een ergotherapeut geweest?' is equal to 'Ja' answer this question:***<br>Hoeveel afspraken had u?	[ ] afspraken
12.5	Bent u in de afgelopen 3 maanden naar een logopedist geweest?	◯ Nee<br>◯ Ja
12.5.1	***If 'Bent u in de afgelopen 3 maanden naar een logopedist geweest?' is equal to 'Ja' answer this question:***<br>Hoeveel afspraken had u?	[ ] afspraken
12.6	Bent u in de afgelopen 3 maanden naar een diëtist geweest?	◯ Nee<br>◯ Ja
12.6.1	***If 'Bent u in de afgelopen 3 maanden naar een diëtist geweest?' is equal to 'Ja' answer this question:***<br>Hoeveel afspraken had u?	[ ] afspraken

12.7	Bent u in de afgelopen 3 maanden naar een homeopaat geweest? Of een acupuncturist?	◯ Nee<br>◯ Ja
12.7.1	***If 'Bent u in de afgelopen 3 maanden naar een homeopaat geweest? Of een acupuncturist?' is equal to 'Ja' answer this question:***<br>Hoeveel afspraken had u?	[ ] afspraken
12.8	Bent u in de afgelopen 3 maanden naar een psycholoog geweest? Of een psychotherapeut of psychiater?	◯ Nee<br>◯ Ja
12.8.1	***If 'Bent u in de afgelopen 3 maanden naar een psycholoog geweest? Of een psychotherapeut of psychiater?' is equal to 'Ja' answer this question:***<br>Hoeveel afspraken had u?	[ ] afspraken
12.9	Heeft u in de afgelopen 3 maanden afspraken gehad met de bedrijfsarts?	◯ Nee<br>◯ Ja
12.9.1	***If 'Heeft u in de afgelopen 3 maanden afspraken gehad met de bedrijfsarts?' is equal to 'Ja' answer this question:***<br>Hoeveel afspraken had u?	[ ] afspraken
12.10	Heeft u in de afgelopen 3 maanden hulp van de thuiszorg gehad?	◯ Ja<br>◯ Nee
12.10.1	***If 'Heeft u in de afgelopen 3 maanden hulp van de thuiszorg gehad?' is equal to 'Ja' answer this question:***<br>Wat voor hulp van de thuiszorg heeft u gehad in de afgelopen 3 maanden?	☐ Huishoudelijke hulp; voorbeeld: stofzuigen, bed opmaken, boodschappen doen<br>☐ Verzorging van uzelf; voorbeeld: hulp bij douchen of aankleden<br>☐ Verpleging; voorbeeld: verband omdoen, medicijnen geven, bloeddruk meten
12.10.1.1	***If 'Wat voor hulp van de thuiszorg heeft u gehad in de afgelopen 3 maanden?' is equal to 'Huishoudelijke hulp; voorbeeld: stofzuigen, bed opmaken, boodschappen doen' answer this question:***<br>Hoeveel weken heeft u deze thuiszorg gehad? Huishoudelijke hulp:	[ ] weken in de afgelopen 3 maanden
12.10.1.2	***If 'Wat voor hulp van de thuiszorg heeft u gehad in de afgelopen 3 maanden?' is equal to 'Verzorging van uzelf; voorbeeld: hulp bij douchen of aankleden' answer this question:***<br>Hoeveel weken heeft u deze thuiszorg gehad? Verzorging van uzelf:	[ ] weken in de afgelopen 3 maanden

For peer review only - http://bmjopen.bmj.com/site/about/guidelines.xhtml

12.10.1.3	***If 'Wat voor hulp van de thuiszorg heeft u gehad in de afgelopen 3 maanden?' is equal to 'Verpleging; voorbeeld: verband omdoen, medicijnen geven, bloeddruk meten' answer this question:*** Hoeveel weken heeft u deze thuiszorg gehad? Verpleging:	______ weken in de afgelopen 3 maanden
12.10.1.4	***If 'Wat voor hulp van de thuiszorg heeft u gehad in de afgelopen 3 maanden?' is equal to 'Huishoudelijke hulp; voorbeeld: stofzuigen, bed opmaken, boodschappen doen' answer this question:*** Hoeveel uur thuiszorg kreeg u in deze weken gemiddeld? Huishoudelijke hulp: gemiddeld	______ uur in de week
12.10.1.5	***If 'Wat voor hulp van de thuiszorg heeft u gehad in de afgelopen 3 maanden?' is equal to 'Verzorging van uzelf; voorbeeld: hulp bij douchen of aankleden' answer this question:*** Hoeveel uur thuiszorg kreeg u in deze weken gemiddeld? Verzorging van uzelf: gemiddeld	______ uur in de week
12.10.1.6	***If 'Wat voor hulp van de thuiszorg heeft u gehad in de afgelopen 3 maanden?' is equal to 'Verpleging; voorbeeld: verband omdoen, medicijnen geven, bloeddruk meten' answer this question:*** Hoeveel uur thuiszorg kreeg u in deze weken gemiddeld? Verpleging: gemiddeld	______ uur in de week
12.11	Heeft u in de afgelopen 3 maanden medicijnen gebruikt?	◯ Ja<br>◯ Nee
12.11.1	***If 'Heeft u in de afgelopen 3 maanden medicijnen gebruikt?' is equal to 'Ja' answer this question:*** Welke medicijnen heeft u in de afgelopen 3 maanden gebruikt?	______
12.12	Bent u in de afgelopen 3 maanden op de spoedeisende eerste hulp van een ziekenhuis geweest? (Een andere naam voor spoedeisende eerste hulp is EHBO)	◯ Nee<br>◯ Ja
12.12.1	***If 'Bent u in de afgelopen 3 maanden op de spoedeisende eerste hulp van een ziekenhuis geweest? (Een andere naam voor spoedeisende eerste hulp is EHBO)' is equal to 'Ja' answer this question:*** Hoevaak bent u geweest?	______ keer
12.13	Bent u in de afgelopen 3 maanden met een ambulance naar het ziekenhuis gebracht? (Een andere naam voor ambulance is ziekenauto)	◯ Nee<br>◯ Ja

12.13.1	***If 'Bent u in de afgelopen 3 maanden met een ambulance naar het ziekenhuis gebracht? (Een andere naam voor ambulance is ziekenauto)' is equal to 'Ja' answer this question:*** Hoe vaak bent u naar het ziekenhuis gebracht?	keer
12.14	Had u in de afgelopen 3 maanden een afspraak bij de polikliniek van het ziekenhuis? (Het gaat om afspraken voor uzelf met een dokter. Bijvoorbeeld met de cardioloog, reumatoloog of neuroloog)	◯ Ja<br>◯ Nee
12.14.1	***If 'Had u in de afgelopen 3 maanden een afspraak bij de polikliniek van het ziekenhuis? (Het gaat om afspraken voor uzelf met een dokter. Bijvoorbeeld met de cardioloog, reumatoloog of neuroloog)' is equal to 'Ja' answer this question:*** Bij welke soorten dokters bent u in de afgelopen 3 maanden in het ziekenhuis geweest? En hoe vaak?	
12.15	Bent u in de afgelopen 3 maanden overdag in het ziekenhuis geweest voor een behandeling? (U bleef dus niet slapen. U kwam bijvoorbeeld voor een bloedtransfusie, nierdialyse of chemokuur)	◯ Ja<br>◯ Nee
12.15.1	***If 'Bent u in de afgelopen 3 maanden overdag in het ziekenhuis geweest voor een behandeling? (U bleef dus niet slapen. U kwam bijvoorbeeld voor een bloedtransfusie, nierdialyse of chemokuur)' is equal to 'Ja' answer this question:*** Voor welke soort behandeling was dit?	
12.15.2	***If 'Bent u in de afgelopen 3 maanden overdag in het ziekenhuis geweest voor een behandeling? (U bleef dus niet slapen. U kwam bijvoorbeeld voor een bloedtransfusie, nierdialyse of chemokuur)' is equal to 'Ja' answer this question:*** Hoeveel keer moest u in de afgelopen 3 maanden voor deze behandelingen naar het ziekenhuis?	
12.16	Bent u in de afgelopen 3 maanden ergens anders geweest voor een behandeling overdag? (U bleef dus niet slapen. U ging bijvoorbeeld naar de dagopvang van een woon-/zorgcentrum of een psychiatrische instelling. Of naar de dagbehandeling van een revalidatiecentrum)	◯ Ja<br>◯ Nee
12.16.1	***If 'Bent u in de afgelopen 3 maanden ergens anders geweest voor een behandeling overdag? (U bleef dus niet slapen. U ging bijvoorbeeld naar de dagopvang van een woon-/zorgcentrum of een psychiatrische instelling. Of naar de dagbehandeling van een revalidatiecentrum)' is equal to 'Ja' answer this question:*** Wat voor instelling was dit?	☐ Woon-/zorgcentrum<br>☐ Revalidatiecentrum<br>☐ Psychiatrische instelling<br>☐ Een andere instelling, namelijk

12.16.1.1	***If 'Wat voor instelling was dit?' is equal to 'Een andere instelling, namelijk' answer this question:*** Wat voor instelling was dit?	
12.16.1.2	***If 'Wat voor instelling was dit?' is equal to 'Woon-/zorgcentrum' answer this question:*** Hoe vaak moest u hier in de afgelopen 3 maanden naartoe? Naar het woon-/zorgcentrum:	keer in de afgelopen 3 maanden
12.16.1.3	***If 'Wat voor instelling was dit?' is equal to 'Revalidatiecentrum' answer this question:*** Hoe vaak moest u hier in de afgelopen 3 maanden naartoe? Naar het revalidatiecentrum:	keer in de afgelopen 3 maanden
12.16.1.4	***If 'Wat voor instelling was dit?' is equal to 'Psychiatrische instelling' answer this question:*** Hoe vaak moest u hier in de afgelopen 3 maanden naartoe? Naar de psychiatrische instelling:	keer in de afgelopen 3 maanden
12.16.1.5	***If 'Wat voor instelling was dit?' is equal to 'Een andere instelling, namelijk' answer this question:*** Hoe vaak moest u hier in de afgelopen 3 maanden naartoe? Naar de andere instelling:	keer in de afgelopen 3 maanden
12.17	Heeft u in de afgelopen 3 maanden weleens in het ziekenhuis gelegen? (U moest dus blijven slapen. Bijvoorbeeld omdat u geopereerd was en niet meteen naar huis kon)	◯ Ja<br>◯ Nee
12.17.1	***If 'Heeft u in de afgelopen 3 maanden weleens in het ziekenhuis gelegen? (U moest dus blijven slapen. Bijvoorbeeld omdat u geopereerd was en niet meteen naar huis kon)' is equal to 'Ja' answer this question:*** Hoe vaak heeft u in de afgelopen 3 maanden in het ziekenhuis gelegen?	keer in de afgelopen 3 maanden
12.17.2	***If 'Heeft u in de afgelopen 3 maanden weleens in het ziekenhuis gelegen? (U moest dus blijven slapen. Bijvoorbeeld omdat u geopereerd was en niet meteen naar huis kon)' is equal to 'Ja' answer this question:*** Hoe lang heeft u in het ziekenhuis gelegen?	dagen in de afgelopen 3 maanden
12.18	Moest u in de afgelopen 3 maanden ergens anders blijven slapen voor uw gezondheid? (Bijvoorbeeld in een woon-/zorgcentrum, psychiatrische instelling of revalidatiecentrum)	◯ Ja<br>◯ Nee

12.18.1	***If 'Moest u in de afgelopen 3 maanden ergens anders blijven slapen voor uw gezondheid? (Bijvoorbeeld in een woon-/zorgcentrum, psychiatrische instelling of revalidatiecentrum)' is equal to 'Ja' answer this question:*** Wat voor instelling was dit?	☐ Woon-/zorgcentrum<br>☐ Revalidatiecentrum<br>☐ Psychiatrische instelling<br>☐ Een andere instelling, namelijk
12.18.1.1	***If 'Wat voor instelling was dit?' is equal to 'Een andere instelling, namelijk' answer this question:*** Een andere instelling, namelijk	
12.18.1.2	***If 'Wat voor instelling was dit?' is equal to 'Woon-/zorgcentrum' answer this question:*** Hoe lang bent u in deze instelling geweest? In het woon-/zorgcentrum:	____ dagen in de afgelopen 3 maanden
12.18.1.3	***If 'Wat voor instelling was dit?' is equal to 'Revalidatiecentrum' answer this question:*** Hoe lang bent u in deze instelling geweest? In het revalidatiecentrum:	____ dagen in de afgelopen 3 maanden
12.18.1.4	***If 'Wat voor instelling was dit?' is equal to 'Psychiatrische instelling' answer this question:*** Hoe lang bent u in deze instelling geweest? In de psychiatrische instelling:	____ dagen in de afgelopen 3 maanden
12.18.1.5	***If 'Wat voor instelling was dit?' is equal to 'Een andere instelling, namelijk' answer this question:*** Hoe lang bent u in deze instelling geweest? In de andere instelling:	____ dagen in de afgelopen 3 maanden
12.19	Heeft u in de afgelopen 3 maanden hulp gekregen van een familielid of een bekende vanwege uw lichamelijke of psychische problemen?	◯ Ja<br>◯ Nee
12.19.1	***If 'Heeft u in de afgelopen 3 maanden hulp gekregen van een familielid of een bekende vanwege uw lichamelijke of psychische problemen?' is equal to 'Ja' answer this question:*** Wat voor hulp van familieleden of bekenden heeft u gehad in de afgelopen 3 maanden?	☐ Huishoudelijke hulp - voorbeeld: stofzuigen, bed opmaken, boodschappen doen, klaarmaken van eten en drinken, verzorgen van kinderen<br>☐ Verzorging van uzelf - voorbeeld: hulp bij douchen of aankleden, hulp bij het eten en drinken of het geven van medicijnen<br>☐ Praktische hulp - voorbeeld: ondersteuning bij wandelen, het maken van uitstapjes of bezoekjes aan bekenden, bezoeken aan de huisarts of het ziekenhuis, het regelen van hulp of het regelen van financiële zaken

12.19.1.1	***If 'Wat voor hulp van familieleden of bekenden heeft u gehad in de afgelopen 3 maanden?' is equal to 'Huishoudelijke hulp - voorbeeld: stofzuigen, bed opmaken, boodschappen doen, klaarmaken van eten en drinken, verzorgen van kinderen' answer this question:*** Hoeveel weken heeft u deze hulp gehad? Huishoudelijke hulp:	weken in de afgelopen 3 maanden
12.19.1.2	***If 'Wat voor hulp van familieleden of bekenden heeft u gehad in de afgelopen 3 maanden?' is equal to 'Verzorging van uzelf - voorbeeld: hulp bij douchen of aankleden, hulp bij het eten en drinken of het geven van medicijnen' answer this question:*** Hoeveel weken heeft u deze hulp gehad? Verzorging van uzelf:	weken in de afgelopen 3 maanden
12.19.1.3	***If 'Wat voor hulp van familieleden of bekenden heeft u gehad in de afgelopen 3 maanden?' is equal to 'Praktische hulp - voorbeeld: ondersteuning bij wandelen, het maken van uitstapjes of bezoekjes aan bekenden, bezoeken aan de huisarts of het ziekenhuis, het regelen van hulp of het regelen van financiële zaken' answer this question:*** Hoeveel uur hulp kreeg u in deze weken gemiddeld? Praktische hulp:	weken in de afgelopen 3 maanden
12.19.1.4	***If 'Wat voor hulp van familieleden of bekenden heeft u gehad in de afgelopen 3 maanden?' is equal to 'Huishoudelijke hulp - voorbeeld: stofzuigen, bed opmaken, boodschappen doen, klaarmaken van eten en drinken, verzorgen van kinderen' answer this question:*** Hoeveel uur hulp kreeg u in deze weken gemiddeld? Huishoudelijke hulp: gemiddeld	uur in de week
12.19.1.5	***If 'Wat voor hulp van familieleden of bekenden heeft u gehad in de afgelopen 3 maanden?' is equal to 'Verzorging van uzelf - voorbeeld: hulp bij douchen of aankleden, hulp bij het eten en drinken of het geven van medicijnen' answer this question:*** Hoeveel uur hulp kreeg u in deze weken gemiddeld? Verzorging van uzelf: gemiddeld	uur in de week
12.19.1.6	***If 'Wat voor hulp van familieleden of bekenden heeft u gehad in de afgelopen 3 maanden?' is equal to 'Praktische hulp - voorbeeld: ondersteuning bij wandelen, het maken van uitstapjes of bezoekjes aan bekenden, bezoeken aan de huisarts of het ziekenhuis, het regelen van hulp of het regelen van financiële zaken' answer this question:*** Hoeveel uur hulp kreeg u in deze weken gemiddeld? Praktische hulp: gemiddeld	uur in de week

Vragenlijst PRIMA studie 52 weken - Dank

Number	Question	Answers
13.1	Hartelijk dank voor de tijd die u heeft genomen om de vragenlijsten in te vullen. Indien u nog opmerkingen heeft kunt u deze hierin plaatsen.	

Survey 'Vragenlijst PRIMA studie 6 weken'

Vragenlijst PRIMA studie 6 weken - AOFAS - Pijn

Number	Question	Answers
1.1	Hoeveel pijn ervaart u aan uw voet?	☐ Geen<br>☐ Milde pijnklachten of af en toe pijn<br>☐ Matige pijnklachten, dagelijks<br>☐ Vrijwel continu erge pijn

Vragenlijst PRIMA studie 6 weken - AOFAS resterende gedeelte

Number	Question	Answers
2.1	Hoeveel beperkingen in het dagelijks leven ervaart u?	○ Geen beperkingen, geen hulpmiddelen nodig<br>○ Geen beperkingen in algemeen dagelijkse bezigheden, wel beperkingen in recreatieve activiteiten (sport, hobbies, etc) geen ondersteuning<br>○ Beperkingen bij dagelijkse- en recreatieve activiteiten, stok<br>○ Ernstige beperkingen bij dagelijkse- en recreatieactiviteiten, walker, krukken, rolstoel, brace

2.2	Wat is de afstand die u in één keer kunt lopen?	◯ Meer dan 600 meter<br>◯ 400 - 600 meter<br>◯ 100 - 300 meter<br>◯ Minder dan 100 meter
2.3	Heeft u moeite met lopen op een bepaalde ondergrond?	◯ Geen problemen bij lopen<br>◯ Enig problemen op ongelijke ondergrond, trap, helling, ladders<br>◯ Ernstige problemen bij ongelijke ondergrond, trap, helling, ladders
2.4	Hoe zou u uw looppatroon beoordelen?	◯ Normaal of minimaal afwijkend<br>◯ Duidelijk<br>◯ Ernstig
2.5	Hoe zou u de beweeglijkheid van uw voet beoordelen ten opzicht van de andere zijde of ten opzicte van vóór het ongeval?	◯ Hetzelfde of licht beperkt t.o.v. de niet aangedane zijde of zoals voor het ongeval<br>◯ Ongeveer de helft beperkt<br>◯ Sterk verminderd, bijna geen bewegingen mogelijk
2.6	Hoe zou u de beweeglijkheid van uw enkel beoordelen ten opzichte van de andere zijde of ten opzichte van voor het ongeval?	◯ Hetzelfde of licht beperkt t.o.v. de niet aangedane zijde of zoals voor het ongeval<br>◯ Ongeveer de helft beperkt<br>◯ Sterk verminderd, bijna geen bewegingen mogelijk
2.7	Hoe zou u de stabiliteit van uw voet en enkel beoordelen	◯ Stabiel, ik verzwik mijn enkel hooguit incidenteel<br>◯ Niet stabiel, ik verzwik mijn enkel vaak
2.8	Hoe zou u de stand van uw voet beoordelen ten opzichte van de andere zijde of ten opzichte van voor het ongeval?	◯ Goed, ik kan mijn voet goed plat op de grond zetten, waarbij de stand niet afwijkend is<br>◯ Ik sta vrijwel alleen op de buitenzijde van mijn voet<br>◯ Ik kan mijn voet niet plat op de grond zetten en loop vrijwel alleen op mijn voorvoet

Vragenlijst PRIMA studie 6 weken - VAS Pain score

Number	Question	Answers	
3.1	VAS pijnscore (Geef uw minimaal pijnscore aan tijdens dagelijkse activiteiten op een schaal van 0 tot 100, waarbij 0 geen pijn is en 100 de ergste pijn denkbaar is)	(0.00)	100 (100.00)

Vragenlijst PRIMA studie 6 weken - AAS

Number	Question	Answers

4.1	Wat is het meest bij u van toepassing ten aanzien van uw acitviteit?	☐ American Football<br>☐ Basketbal<br>☐ Gymnastiek/turnen<br>☐ Handbal<br>☐ Rugby<br>☐ Voetbal<br>☐ Hockey<br>☐ Korfbal<br>☐ Gevechtssporten: judo, karate, kung fu, taekwando, jiu jitsu, etc.<br>☐ Oriëntatielopen<br>☐ Rhytmische gymnastiek<br>☐ Volleybal<br>☐ Boxen<br>☐ Freestyle snowboarden<br>☐ Ijshockey<br>☐ Tennis<br>☐ Worstelen<br>☐ Fitness, aerobics<br>☐ Badminton<br>☐ Baseball<br>☐ Cross-country hardlopen<br>☐ Moderne pentathlon<br>☐ Squash<br>☐ Surfen, windsurfen<br>☐ Tafel tennis<br>☐ Atletiek: spring-, werponderdelen<br>☐ Waterskieën<br>☐ Dans<br>☐ Schermen<br>☐ Zaalhockey<br>☐ Bergbeklimmen<br>☐ Langlauf<br>☐ Parachute springen<br>☐ Softball<br>☐ Speciale beroepen en werkactiviteiten; speciale beroepen: ballet, professioneel soldaat, speciale reddingswerker, stuntman, etc.<br>☐ Duiken<br>☐ Scubaduiken<br>☐ Skaten, in-line skaten<br>☐ Atletiek: looponderdelen<br>☐ Triatlon<br>☐ Gewichtsheffen, body-building

☐ Alle competitieve sporten hieronder met ‘seasonal’ conditioning
☐ Zwaar fysiek werk
☐ Alpine skiën en snowboarden
☐ Bowlen/curlen
☐ Golf
☐ Mountainbike/BMX
☐ Powerliften
☐ Zeilen
☐ Fysiek werk
☐ Wielrennen
☐ Paardrijden
☐ Motorsporten/technische sporten
☐ Roeien, kayakken
☐ Boogschieten
☐ Water polo en zwemmen
☐ Kunnen lopen op oneven grond
☐ Geen sport, echter geen limitatie in dagelijkse activiteiten
☐ Kunnen lopen op even grond, maar de dagelijkste activiteiten zijn gelimiteerd
☐ Niet kunnen lopen, fysieke handicap wegens enkelproblemen

4.1.1	***If 'Wat is het meest bij u van toepassing ten aanzien van uw acitviteit?' is equal to 'American Football' answer this question:***<br>American Football	◯ Professioneel<br>◯ Competitief<br>◯ Recreatief
4.1.2	***If 'Wat is het meest bij u van toepassing ten aanzien van uw acitviteit?' is equal to 'Basketbal' answer this question:***<br>Basketbal	◯ Professioneel<br>◯ Competitief<br>◯ Recreatief
4.1.3	***If 'Wat is het meest bij u van toepassing ten aanzien van uw acitviteit?' is equal to 'Gymnastiek/turnen' answer this question:***<br>Gymnastiek/turnen	◯ Professioneel<br>◯ Competitief<br>◯ Recreatief
4.1.4	***If 'Wat is het meest bij u van toepassing ten aanzien van uw acitviteit?' is equal to 'Handbal' answer this question:***<br>Handbal	◯ Professioneel<br>◯ Competitief<br>◯ Recreatief
4.1.5	***If 'Wat is het meest bij u van toepassing ten aanzien van uw acitviteit?' is equal to 'Rugby' answer this question:***<br>Rugby	◯ Professioneel<br>◯ Competitief<br>◯ Recreatief

4.1.6	***If 'Wat is het meest bij u van toepassing ten aanzien van uw acitviteit?' is equal to 'Voetbal' answer this question:*** Voetbal	◯ Professioneel<br>◯ Competitief<br>◯ Recreatief
4.1.7	***If 'Wat is het meest bij u van toepassing ten aanzien van uw acitviteit?' is equal to 'Hockey' answer this question:*** Hockey	◯ Professioneel<br>◯ Competitief<br>◯ Recreatief
4.1.8	***If 'Wat is het meest bij u van toepassing ten aanzien van uw acitviteit?' is equal to 'Korfbal' answer this question:*** Korfbal	◯ Professioneel<br>◯ Competitief<br>◯ Recreatief
4.1.9	***If 'Wat is het meest bij u van toepassing ten aanzien van uw acitviteit?' is equal to 'Gevechtssporten: judo, karate, kung fu, taekwando, jiu jitsu, etc.' answer this question:*** Gevechtssporten: judo, karate, kung fu, taekwando, jiu jitsu, etc.	◯ Professioneel<br>◯ Competitief<br>◯ Recreatief
4.1.10	***If 'Wat is het meest bij u van toepassing ten aanzien van uw acitviteit?' is equal to 'Oriëntatielopen' answer this question:*** Oriëntatielopen	◯ Professioneel<br>◯ Competitief<br>◯ Recreatief
4.1.11	***If 'Wat is het meest bij u van toepassing ten aanzien van uw acitviteit?' is equal to 'Rhytmische gymnastiek' answer this question:*** Rhytmisch gymnastiek	◯ Professioneel<br>◯ Competitief<br>◯ Recreatief
4.1.12	***If 'Wat is het meest bij u van toepassing ten aanzien van uw acitviteit?' is equal to 'Volleybal' answer this question:*** Volleybal	◯ Professioneel<br>◯ Competitief<br>◯ Recreatief
4.1.13	***If 'Wat is het meest bij u van toepassing ten aanzien van uw acitviteit?' is equal to 'Boxen' answer this question:*** Boxen	◯ Professioneel<br>◯ Competitief<br>◯ Recreatief
4.1.14	***If 'Wat is het meest bij u van toepassing ten aanzien van uw acitviteit?' is equal to 'Freestyle snowboarden' answer this question:*** Freestyle snowboarden	◯ Professioneel<br>◯ Competitief<br>◯ Recreatief
4.1.15	***If 'Wat is het meest bij u van toepassing ten aanzien van uw acitviteit?' is equal to 'Freestyle snowboarden' answer this question:*** IJshockey	◯ Professioneel<br>◯ Competitief<br>◯ Recreatief

4.1.16	***If 'Wat is het meest bij u van toepassing ten aanzien van uw acitviteit?' is equal to 'Tennis' answer this question:***<br>Tennis	○ Professioneel<br>○ Competitief<br>○ Recreatief
4.1.17	***If 'Wat is het meest bij u van toepassing ten aanzien van uw acitviteit?' is equal to 'Worstelen' answer this question:***<br>Worstelen	○ Professioneel<br>○ Competitief<br>○ Recreatief
4.1.18	***If 'Wat is het meest bij u van toepassing ten aanzien van uw acitviteit?' is equal to 'Fitness, aerobics' answer this question:***<br>Fitness, aerobics	○ Professioneel<br>○ Competitief<br>○ Recreatief
4.1.19	***If 'Wat is het meest bij u van toepassing ten aanzien van uw acitviteit?' is equal to 'Badminton' answer this question:***<br>Badminton	○ Professioneel<br>○ Competitief<br>○ Recreatief
4.1.20	***If 'Wat is het meest bij u van toepassing ten aanzien van uw acitviteit?' is equal to 'Baseball' answer this question:***<br>Baseball	○ Professioneel<br>○ Competitief<br>○ Recreatief
4.1.21	***If 'Wat is het meest bij u van toepassing ten aanzien van uw acitviteit?' is equal to 'Cross-country hardlopen' answer this question:***<br>Cross-country hardlopen	○ Professioneel<br>○ Competitief<br>○ Recreatief
4.1.22	***If 'Wat is het meest bij u van toepassing ten aanzien van uw acitviteit?' is equal to 'Moderne pentathlon' answer this question:***<br>Moderne pentathlon	○ Professioneel<br>○ Competitief<br>○ Recreatief
4.1.23	***If 'Wat is het meest bij u van toepassing ten aanzien van uw acitviteit?' is equal to 'Squash' answer this question:***<br>Squash	○ Professioneel<br>○ Competitief<br>○ Recreatief
4.1.24	***If 'Wat is het meest bij u van toepassing ten aanzien van uw acitviteit?' is equal to 'Surfen, windsurfen' answer this question:***<br>Surfen, windsurfen	○ Professioneel<br>○ Competitief<br>○ Recreatief
4.1.25	***If 'Wat is het meest bij u van toepassing ten aanzien van uw acitviteit?' is equal to 'Tafel tennis' answer this question:***<br>Tafel tennis	○ Professioneel<br>○ Competitief<br>○ Recreatief

4.1.26	***If 'Wat is het meest bij u van toepassing ten aanzien van uw acitviteit?' is equal to 'Atletiek: spring-, werponderdelen' answer this question:***<br>Atletiek: spring-, werponderdelen	◯ Professioneel<br>◯ Competitief<br>◯ Recreatief
4.1.27	***If 'Wat is het meest bij u van toepassing ten aanzien van uw acitviteit?' is equal to 'Waterskieën' answer this question:***<br>Waterskien	◯ Professioneel<br>◯ Competitief<br>◯ Recreatief
4.1.28	***If 'Wat is het meest bij u van toepassing ten aanzien van uw acitviteit?' is equal to 'Dans' answer this question:***<br>Dans	◯ Professioneel<br>◯ Competitief<br>◯ Recreatief
4.1.29	***If 'Wat is het meest bij u van toepassing ten aanzien van uw acitviteit?' is equal to 'Schermen' answer this question:***<br>Schermen	◯ Professioneel<br>◯ Competitief<br>◯ Recreatief
4.1.30	***If 'Wat is het meest bij u van toepassing ten aanzien van uw acitviteit?' is equal to 'Zaalhockey' answer this question:***<br>Zaalhockey	◯ Professioneel<br>◯ Competitief<br>◯ Recreatief
4.1.31	***If 'Wat is het meest bij u van toepassing ten aanzien van uw acitviteit?' is equal to 'Bergbeklimmen' answer this question:***<br>Bergbeklimmen	◯ Professioneel<br>◯ Competitief<br>◯ Recreatief
4.1.32	***If 'Wat is het meest bij u van toepassing ten aanzien van uw acitviteit?' is equal to 'Langlauf' answer this question:***<br>Langlauf	◯ Professioneel<br>◯ Competitief<br>◯ Recreatief
4.1.33	***If 'Wat is het meest bij u van toepassing ten aanzien van uw acitviteit?' is equal to 'Parachute springen' answer this question:***<br>Parachute springen	◯ Professioneel<br>◯ Competitief<br>◯ Recreatief
4.1.34	***If 'Wat is het meest bij u van toepassing ten aanzien van uw acitviteit?' is equal to 'Softball' answer this question:***<br>Softball	◯ Professioneel<br>◯ Competitief<br>◯ Recreatief

4.1.35	***If 'Wat is het meest bij u van toepassing ten aanzien van uw acitviteit?' is equal to 'Speciale beroepen en werkactiviteiten; speciale beroepen: ballet, professioneel soldaat, speciale reddingswerker, stuntman, etc.' answer this question:***<br>Speciale beroepen en werkactiviteiten speciale beroepen: ballet, professioneel soldaat, speciale reddingswerker, stuntman, etc.	○ ballet, professioneel soldaat, speciale reddingswerker, stuntman, etc.
4.1.36	***If 'Wat is het meest bij u van toepassing ten aanzien van uw acitviteit?' is equal to 'Duiken' answer this question:***<br>Duiken	○ Professioneel/competitief<br>○ Recreatief
4.1.37	***If 'Wat is het meest bij u van toepassing ten aanzien van uw acitviteit?' is equal to 'Scubaduiken' answer this question:***<br>Scubaduiken	○ Professioneel/competitief<br>○ Recreatief
4.1.38	***If 'Wat is het meest bij u van toepassing ten aanzien van uw acitviteit?' is equal to 'Skaten, in-line skaten' answer this question:***<br>skaten, in-linen skaten	○ Professioneel/competitief<br>○ Recreatief
4.1.39	***If 'Wat is het meest bij u van toepassing ten aanzien van uw acitviteit?' is equal to 'Atletiek: looponderdelen' answer this question:***<br>Atletiek: looponderdelen	○ Professioneel/competitief<br>○ Recreatief
4.1.40	***If 'Wat is het meest bij u van toepassing ten aanzien van uw acitviteit?' is equal to 'Triatlon' answer this question:***<br>triatlon	○ Professioneel/competitief<br>○ Recreatief
4.1.41	***If 'Wat is het meest bij u van toepassing ten aanzien van uw acitviteit?' is equal to 'Gewichtsheffen, body-building' answer this question:***<br>Gewichtsheffen, body-building	○ Professioneel/competitief<br>○ Recreatief
4.1.42	***If 'Wat is het meest bij u van toepassing ten aanzien van uw acitviteit?' is equal to 'Alle competitieve sporten hieronder met 'seasonal' conditioning' answer this question:***<br>Alle competitieve sporten hieronder met 'seasonal' conditioning	○ Alle competitieve sporten hieronder met 'seasonal' conditioning
4.1.43	***If 'Wat is het meest bij u van toepassing ten aanzien van uw acitviteit?' is equal to 'Zwaar fysiek werk' answer this question:***<br>Zwaar fysiek werk	○ Alle competitieve sporten hieronder met 'seasonal' conditioning

4.1.44	***If 'Wat is het meest bij u van toepassing ten aanzien van uw acitviteit?' is equal to 'Alpine skiën en snowboarden' answer this question:***<br>Alpine skien en snowboarden	○ Alpine skien en snowboarden
4.1.45	***If 'Wat is het meest bij u van toepassing ten aanzien van uw acitviteit?' is equal to 'Bowlen/curlen' answer this question:***<br>Bowlen/curlen	○ Bowlen/curlen
4.1.46	***If 'Wat is het meest bij u van toepassing ten aanzien van uw acitviteit?' is equal to 'Golf' answer this question:***<br>Golf	○ Golf
4.1.47	***If 'Wat is het meest bij u van toepassing ten aanzien van uw acitviteit?' is equal to 'Mountainbike/BMX' answer this question:***<br>Mountainbike/BMX	○ Mountainbike/BMX
4.1.48	***If 'Wat is het meest bij u van toepassing ten aanzien van uw acitviteit?' is equal to 'Powerliften' answer this question:***<br>Powerliften	○ Powerliften
4.1.49	***If 'Wat is het meest bij u van toepassing ten aanzien van uw acitviteit?' is equal to 'Zeilen' answer this question:***<br>Zeilen	○ Zeilen
4.1.50	***If 'Wat is het meest bij u van toepassing ten aanzien van uw acitviteit?' is equal to 'Fysiek werk' answer this question:***<br>Fysiek werk	○ Fysiek werk
4.1.51	***If 'Wat is het meest bij u van toepassing ten aanzien van uw acitviteit?' is equal to 'Wielrennen' answer this question:***<br>Wielrennen	○ Wielrennen
4.1.52	***If 'Wat is het meest bij u van toepassing ten aanzien van uw acitviteit?' is equal to 'Paardrijden' answer this question:***<br>Paardrijden	○ Paardrijden
4.1.53	***If 'Wat is het meest bij u van toepassing ten aanzien van uw acitviteit?' is equal to 'Motorsporten/technische sporten' answer this question:***<br>Motorsporten/technische sporten	○ Motorsporten/technische sporten
4.1.54	***If 'Wat is het meest bij u van toepassing ten aanzien van uw acitviteit?' is equal to 'Roeien, kayakken' answer this question:***<br>Roeien, kayakken	○ Roeien, kayakken

4.1.55	***If 'Wat is het meest bij u van toepassing ten aanzien van uw acitviteit?' is equal to 'Boogschieten' answer this question:*** Boogschieten	◯ Boogschieten
4.1.56	***If 'Wat is het meest bij u van toepassing ten aanzien van uw acitviteit?' is equal to 'Water polo en zwemmen' answer this question:*** Water polo en zwemmen	◯ Water polo en zwemmen
4.1.57	***If 'Wat is het meest bij u van toepassing ten aanzien van uw acitviteit?' is equal to 'Kunnen lopen op oneven grond' answer this question:*** Kunnen lopen op oneven grond	◯ Kunnen lopen op oneven grond
4.1.58	***If 'Wat is het meest bij u van toepassing ten aanzien van uw acitviteit?' is equal to 'Geen sport, echter geen limitatie in dagelijkse activiteiten' answer this question:*** Geen sport, echter geen limitatie in dagelijkse activiteiten	◯ Geen sport, echter geen limitatie in dagelijkse activiteiten
4.1.59	***If 'Wat is het meest bij u van toepassing ten aanzien van uw acitviteit?' is equal to 'Kunnen lopen op even grond, maar de dagelijkste activiteiten zijn gelimiteerd' answer this question:*** Kunnen lopen op even grond, maar de dagelijkste activiteiten zijn gelimiteerd	◯ Kunnen lopen op even grond, maar de dagelijkste activiteiten zijn gelimiteerd
4.1.60	***If 'Wat is het meest bij u van toepassing ten aanzien van uw acitviteit?' is equal to 'Niet kunnen lopen, fysieke handicap wegens enkelproblemen' answer this question:*** Niet kunnen lopen, fysieke handicap wegens enkelproblemen	◯ Niet kunnen lopen, fysieke handicap wegens enkelproblemen

Vragenlijst PRIMA studie 6 weken - Hoe tevreden bent u over de enkelklachten?

Number	Question	Answers
5.1	Hoe tevreden bent u over de enkelklachten?	◯ Slecht<br>◯ Matig<br>◯ Goed<br>◯ Uitstekend

Vragenlijst PRIMA studie 6 weken - SF-36

Number	Question	Answers
	Deze vragenlijst gaat over uw standpunten t.a.v. uw gezondheid. Met behulp van deze gegevens kan worden bijgehouden hoe u zich voelt en hoe goed u in staat bent uw gebruikelijke bezigheden uit te voeren. Beantwoord elke vraag door een antwoord aan te klikken. Als u niet zeker weet hoe u een vraag moet beantwoorden, geef dan het best mogelijke antwoord.	
6.1	Hoe zou u over het algemeen uw gezondheid noemen?	◯ Uitstekend ◯ Zeer goed ◯ Goed ◯ Matig ◯ Slecht
6.2	Hoe beoordeelt u nu uw gezondheid over het algemeen vergeleken met een jaar geleden?	◯ Veel beter dan een jaar geleden ◯ Wat beter dan een jaar geleden ◯ Ongeveer hetzelfde als een jaar geleden ◯ Wat slechter dan een jaar geleden ◯ Veel slechter dan een jaar geleden
6.3	Wordt u door uw gezondheid op dit moment beperkt bij forse inspanning, zoals hardlopen, tillen van zware voorwerpen of een veeleisende sport beoefenen?	◯ Ja, ernstig beperkt ◯ Ja, een beetje beperkt ◯ Nee, helemaal niet beperkt
6.4	Wordt u door uw gezondheid op dit moment beperkt bij matige inspanning zoals een tafel verplaatsen, stofzuigen, zwemmen of fietsen?	◯ Ja, ernstig beperkt ◯ Ja, een beetje beperkt ◯ Nee, helemaal niet beperkt
6.5	Wordt u door uw gezondheid op dit moment beperkt bij boodschappen tillen of dragen?	◯ Ja, ernstig beperkt ◯ Ja, een beetje beperkt ◯ Nee, helemaal niet beperkt
6.6	Wordt u door uw gezondheid op dit moment beperkt bij een paar trappen oplopen?	◯ Ja, ernstig beperkt ◯ Ja, een beetje beperkt ◯ Nee, helemaal niet beperkt
6.7	Wordt u door uw gezondheid op dit moment beperkt bij één trap oplopen?	◯ Ja, ernstig beperkt ◯ Ja, een beetje beperkt ◯ Nee, helemaal niet beperkt
6.8	Wordt u door uw gezondheid op dit moment beperkt bij bukken, knielen of hurken?	◯ Ja, ernstig beperkt ◯ Ja, een beetje beperkt ◯ Nee, helemaal niet beperkt
6.9	Wordt u door uw gezondheid op dit moment beperkt bij meer dan één kilometer lopen?	◯ Ja, ernstig beperkt ◯ Ja, een beetje beperkt ◯ Nee, helemaal niet beperkt
6.10	Wordt u door uw gezondheid op dit moment beperkt bij een paar honderd meter lopen?	◯ Ja, ernstig beperkt ◯ Ja, een beetje beperkt ◯ Nee, helemaal niet beperkt

6.11	Wordt u door uw gezondheid op dit moment beperkt bij ongeveer honderd meter lopen?	◯ Ja, ernstig beperkt ◯ Ja, een beetje beperkt ◯ Nee, helemaal niet beperkt
6.12	Wordt u door uw gezondheid op dit moment beperkt bij uzelf wassen of aankleden?	◯ Ja, ernstig beperkt ◯ Ja, een beetje beperkt ◯ Nee, helemaal niet beperkt
6.13	U besteedde in de afgelopen 4 weken minder tijd aan werk of andere bezigheden	◯ Ja ◯ Nee
6.14	U heeft in de afgelopen 4 weken minder bereikt dan u zou willen	◯ Ja ◯ Nee
6.15	U was in de afgelopen 4 weken beperkt in het soort werk of andere bezigheden.	◯ Ja ◯ Nee
6.16	U had de afgelopen 4 weken moeite om uw werk of andere bezigheden uit te voeren (het kostte u bijvoorbeeld extra inspanning).	◯ Ja ◯ Nee
6.17	U besteedde in de afgelopen 4 weken minder tijd aan werk of andere bezigheden ten gevolge van emotionele problemen	◯ Ja ◯ Nee
6.18	U heeft in de afgelopen 4 weken minder bereikt dan u zou willen ten gevolge van emotionele problemen.	◯ Ja ◯ Nee
6.19	U deed de afgelopen 4 weken uw werk of andere bezigheden niet zo zorgvuldig als gewoonlijk ten gevolge van emotionele problemen.	◯ Ja ◯ Nee
6.20	In hoeverre hebben uw lichamelijke gezondheid of emotionele problemen u gedurende de afgelopen 4 weken gehinderd in uw normale omgang met familie, vrienden of buren, of bij activiteiten in groepsverband?	◯ Helemaal niet ◯ Enigszins ◯ Nogal ◯ Veel ◯ Heel erg veel
6.21	Hoeveel lichamelijke pijn heeft u de afgelopen 4 weken gehad?	◯ Geen<br>◯ Heel licht<br>◯ Licht<br>◯ Nogal<br>◯ Ernstig<br>◯ Heel ernstig
6.22	In welke mate bent u de afgelopen 4 weken door de pijn gehinderd in uw normale werk (zowel werk buitenshuis als huishoudelijk werk)?	◯ Helemaal niet<br>◯ Een klein beetje<br>◯ Nogal<br>◯ Veel<br>◯ Heel erg veel

6.23	Voelde u zich levenslustig?	◯ Altijd<br>◯ Meestal<br>◯ Vaak<br>◯ Soms<br>◯ Zelden<br>◯ Nooit
6.24	Was u erg zenuwachtig?	◯ Altijd<br>◯ Meestal<br>◯ Vaak<br>◯ Soms<br>◯ Zelden<br>◯ Nooit
6.25	Zat u zo in de put dat u niets kon?	◯ Altijd<br>◯ Meestal<br>◯ Vaak<br>◯ Soms<br>◯ Zelden<br>◯ Nooit
6.26	Voelde u zich rustig en tevreden?	◯ Altijd<br>◯ Meestal<br>◯ Vaak<br>◯ Soms<br>◯ Zelden<br>◯ Nooit
6.27	Had u veel energie?	◯ Altijd<br>◯ Meestal<br>◯ Vaak<br>◯ Soms<br>◯ Zelden<br>◯ Nooit
6.28	Voelde u zich somber en neerslachtig?	◯ Altijd<br>◯ Meestal<br>◯ Vaak<br>◯ Soms<br>◯ Zelden<br>◯ Nooit

6.29	Voelde u zich uitgeput?	◯ Altijd<br>◯ Meestal<br>◯ Vaak<br>◯ Soms<br>◯ Zelden<br>◯ Nooit
6.30	Was u een gelukkig mens?	◯ Altijd<br>◯ Meestal<br>◯ Vaak<br>◯ Soms<br>◯ Zelden<br>◯ Nooit
6.31	Voelde u zich moe?	◯ Altijd<br>◯ Meestal<br>◯ Vaak<br>◯ Soms<br>◯ Zelden<br>◯ Nooit
6.32	Hoe vaak hebben uw lichamelijke gezondheid of emotionele problemen u gedurende de afgelopen 4 weken gehinderd bij uw sociale activiteiten (zoals vrienden of familie bezoeken etc.)?	◯ Altijd<br>◯ Meestal<br>◯ Soms<br>◯ Zelden<br>◯ Nooit
6.33	Ik lijk wat gemakkelijker ziek te worden dan andere mensen	◯ Volkomen juist<br>◯ Grotendeels juist<br>◯ Weet ik niet<br>◯ Grotendeels onjuist<br>◯ Volkomen onjuist
6.34	Ik ben even gezond als andere mensen die ik ken	◯ Volkomen juist<br>◯ Grotendeels juist<br>◯ Weet ik niet<br>◯ Grotendeels onjuist<br>◯ Volkomen onjuist

6.35	Ik verwacht dat mijn gezondheid achteruit zal gaan	◯ Volkomen juist<br>◯ Grotendeels juist<br>◯ Weet ik niet<br>◯ Grotendeels onjuist<br>◯ Volkomen onjuist
6.36	Mijn gezondheid is uitstekend	◯ Volkomen juist<br>◯ Grotendeels juist<br>◯ Weet ik niet<br>◯ Grotendeels onjuist<br>◯ Volkomen onjuist

Vragenlijst PRIMA studie 6 weken - GAS

Number	Question	Answers
	Tijdens de eerste afspraak werd een duidelijk doel met u afgesproken. Dit werd tevens per email naar u gestuurd. Hoe is de situatie nu ten aanzien van de destijds gestelde doelstelling?	
7.1	Wat is nu de situatie ten aanzien van het tijdens de eerste afspraak afgesproken doelstelling (Goal attainment Scaling)?	◯ Achteruitgang (minder dan de utigangssituatie)<br>◯ Uitgangssituatie<br>◯ Minder dan het doel<br>◯ Doel<br>◯ Meer dan het doel<br>◯ Veel meer dan het doel

Vragenlijst PRIMA studie 6 weken - EQ-5D-3L

Number	Question	Answers
	Deze vragenlijst gaat over uw standpunten t.a.v. uw gezondheid. Met behulp van deze gegevens kan worden bijgehouden hoe u zich voelt en hoe goed u in staat bent uw gebruikelijke bezigheden uit te voeren. Beantwoord elke vraag door een antwoord aan te klikken. Als u niet zeker weet hoe u een vraag moet beantwoorden, geef dan het best mogelijke antwoord.	
8.1	Hoe is het met uw Mobiliteit gesteld?	◯ Ik heb geen problemen met lopen<br>◯ Ik heb enige problemen met lopen<br>◯ Ik ben bedlegerig

8.2	Hoe is het met uw Zelfzorg gesteld?	◯ Ik heb geen problemen om mijzelf te wassen of aan te kleden<br>◯ Ik heb enige problemen om mijzelf te wassen of aan te kleden<br>◯ Ik ben niet in staat om mijzelf te wassen of aan te kleden
8.3	Hoe is het met de Dagelijkse activiteiten (werk, studie, huishouden, gezins- en vrijetijdsactiviteiten) gesteld?	◯ Ik heb geen problemen met mijn dagelijkse activiteiten<br>◯ Ik heb enige problemen met mijn dagelijkse activiteiten<br>◯ Ik ben niet in staat om mijn dagelijkse activiteiten uit te voeren
8.4	Hoe is het met de Pijn/klachten gesteld?	◯ Ik heb geen pijn of andere klachten<br>◯ Ik heb matige pijn of andere klachten<br>◯ Ik heb zeer ernstige pijn of andere klachten
8.5	Hoe is het met de Stemming gesteld?	◯ Ik ben niet angstig of somber<br>◯ Ik ben matig angstig of somber<br>◯ Ik ben erg angstig of somber
	We willen weten hoe goed of slecht uw gezondheid VANDAAG is. Deze meetschaal loopt van 0 tot 100: 100 staat voor de BESTE gezondheid die u zich kunt voorstellen - 0 staat voor de SLECHTSTE gezondheid die u zich kunt voorstellen	
8.6	Uw Gezondheid vandaag	(0.00) (100.00)

Vragenlijst PRIMA studie 6 weken - AOS

Number	Question	Answers
	Instructies: De lijn naast elke vraag staat voor hoeveel PIJN u heeft in verschillende situaties. De linker kant (0) is “geen pijn” en de rechter kant (100) is “ergste pijn denkbaar”. Geef voor de onderstaande situaties op de lijn aan hoeveel PIJN u in de afgelopen week in de enkel had. Als een of meerdere van deze situaties niet van toepassing waren, dan kiest u de "niet van toepassing" optie.	
9.1	Wat was de hoogte van de ergste pijn in de afgelopen week?	(0.00) (100.00)
9.2	Heeft u pijn als u 's ochtends opstaat?	◯ Ja<br>◯ Nee<br>◯ Niet van toepassing

9.2.1	***If 'Heeft u pijn als u 's ochtends opstaat?' is equal to 'Ja' answer this question:***<br>Hoeveel pijn heeft u voordat u 's ochtends opstaat?	(0.00)	(100.00)
9.3	Heeft u pijn wanneer u op blote voeten loopt?	◯ Ja<br>◯ Nee<br>◯ Niet van toepassing	
9.3.1	***If 'Heeft u pijn wanneer u op blote voeten loopt?' is equal to 'Ja' answer this question:***<br>Hoeveel pijn heeft u als u op blote voeten loopt?	(0.00)	(100.00)
9.4	Heeft u pijn wanneer u op blote voeten staat?	◯ Ja<br>◯ Nee<br>◯ Niet van toepassing	
9.4.1	***If 'Heeft u pijn wanneer u op blote voeten staat?' is equal to 'Ja' answer this question:***<br>Hoeveel pijn heeft u als u op blote voeten staat?	(0.00)	(100.00)
9.5	Heeft u pijn wanneer u met schoenen loopt?	◯ Ja<br>◯ Nee<br>◯ Niet van toepassing	
9.5.1	***If 'Heeft u pijn wanneer u met schoenen loopt?' is equal to 'Ja' answer this question:***<br>Hoeveel pijn heeft u wanneer u met schoenen loopt?	(0.00)	(100.00)
9.6	Heeft u pijn wanneer u met schoenen staat?	◯ Ja<br>◯ Nee<br>◯ Niet van toepassing	
9.6.1	***If 'Heeft u pijn wanneer u met schoenen staat?' is equal to 'Ja' answer this question:***<br>Hoeveel pijn heeft u wanneer u met schoenen staat?	(0.00)	(100.00)
9.7	Heeft u pijn wanneer u loopt met steunzolen of een brace?	◯ Ja<br>◯ Nee<br>◯ Niet van toepassing	
9.7.1	***If 'Heeft u pijn wanneer u loopt met steunzolen of een brace?' is equal to 'Ja' answer this question:***<br>Hoeveel pijn heeft u wanneer u loopt met steunzolen of een brace?	(0.00)	(100.00)

For peer review only - http://bmjopen.bmj.com/site/about/guidelines.xhtml

9.8	Heeft u pijn wanneer u staat met steunzolen of een brace?	◯ Ja<br>◯ Nee<br>◯ Niet van toepassing	
9.8.1	***If 'Heeft u pijn wanneer u staat met steunzolen of een brace?' is equal to 'Ja' answer this question:***<br>Hoeveel pijn heeft u wanneer u staat met steunzolen of een brace?	(0.00)	(100.00)
9.9	Heeft u pijn aan het einde van de dag?	◯ Ja<br>◯ Nee<br>◯ Niet van toepassing	
9.9.1	***If 'Heeft u pijn aan het einde van de dag?' is equal to 'Ja' answer this question:***<br>Hoeveel pijn heeft u aan het einde van de dag?	(0.00)	(100.00)
	Instructies:De lijn naast elke vraag staat voor hoeveel MOEITE u heeft met verschillende activiteiten. De linker kant (0) is “Niet moeilijk” en de rechter kant (100) is “Te moeilijk, niet uitvoerbaar”. Geef voor de onderstaande activiteiten op de lijn aan hoeveel MOEITE u in de afgelopen week door enkelklachten had met onderstaande activiteiten. Als een situatie niet van toepassing is, kies dan de optie "niet van toepassing".		
9.10	Heeft u moeite met door het huis lopen?	◯ Ja<br>◯ Nee<br>◯ Niet van toepassing	
9.10.1	***If 'Heeft u moeite met door het huis lopen?' is equal to 'Ja' answer this question:***<br>Hoeveel moeite heeft u met door het huis lopen?	(0.00)	(100.00)
9.11	Heeft u moeite met buiten lopen op oneven ondergrond?	◯ Ja<br>◯ Nee<br>◯ Niet van toepassing	
9.11.1	***If 'Heeft u moeite met buiten lopen op oneven ondergrond?' is equal to 'Ja' answer this question:***<br>Hoeveel moeite heeft u met buiten lopen op oneven ondergrond?	(0.00)	(100.00)
9.12	Heeft u moeite met een paar honderd meter lopen?	◯ Ja<br>◯ Nee<br>◯ Niet van toepassing	
9.12.1	***If 'Heeft u moeite met een paar honderd meter lopen?' is equal to 'Ja' answer this question:***<br>Hoeveel moeite heeft u met een paar honderd meter lopen?	(0.00)	(100.00)

For peer review only - http://bmjopen.bmj.com/site/about/guidelines.xhtml

9.13	Heeft u moeite met een trap oplopen?	◯ Ja<br>◯ Nee<br>◯ Niet van toepassing	
9.13.1	***If 'Heeft u moeite met een trap oplopen?' is equal to 'Ja' answer this question:***<br>Hoeveel moeite heeft u met een trap oplopen?	(0.00)	(100.00)
9.14	Heeft u moeite met een trap aflopen?	◯ Ja<br>◯ Nee<br>◯ Niet van toepassing	
9.14.1	***If 'Heeft u moeite met een trap aflopen?' is equal to 'Ja' answer this question:***<br>Hoeveel moeite heeft u met een trap aflopen?	(0.00)	(100.00)
9.15	Heeft u moeite met op de tenen staan?	◯ Ja<br>◯ Nee<br>◯ Niet van toepassing	
9.15.1	***If 'Heeft u moeite met op de tenen staan?' is equal to 'Ja' answer this question:***<br>Hoeveel moeite heeft u met op de tenen staan?	(0.00)	(100.00)
9.16	Heeft u moeite met opstaan uit de stoel?	◯ Ja<br>◯ Nee<br>◯ Niet van toepassing	
9.16.1	***If 'Heeft u moeite met opstaan uit de stoel?' is equal to 'Ja' answer this question:***<br>Hoeveel moeite heeft u met opstaan uit de stoel?	(0.00)	(100.00)
9.17	Heeft u moeite met het op- of afstappen van stoepranden?	◯ Ja<br>◯ Nee<br>◯ Niet van toepassing	
9.17.1	***If 'Heeft u moeite met het op- of afstappen van stoepranden?' is equal to 'Ja' answer this question:***<br>Hoeveel moeite heeft u met het op- of afstappen van stoepranden?	(0.00)	(100.00)
9.18	Heeft u moeite met snel lopen of rennen?	◯ Ja<br>◯ Nee<br>◯ Niet van toepassing	

9.18.1	***If 'Heeft u moeite met snel lopen of rennen?' is equal to 'Ja' answer this question:***<br>Hoeveel moeite heeft u met snel lopen of rennen?	(0.00)	(100.00)

Vragenlijst PRIMA studie 6 weken - FAOS

Number	Question	Answers
	Deze lijst vraagt naar uw mening over uw voet/enkel. Uw antwoorden geven ons een beeld van uw voet/enkel klachten en hoe u in staat bent om alledaagse activiteiten uit te voeren in uw huidige situatie. Beantwoorden van een vraag doet u door het aanklikken van een vakje met het volgens u meest juiste antwoord (één vakje per vraag). Als u niet zeker weet hoe u een vraag moet beantwoorden, geeft u dan het antwoord dat volgens u het meest op uw situatie van toepassing is. Deze vraag heeft betrekking op het voorkomen van voet/enkel klachten in de afgelopen week.	
10.1	Is uw voet/enkel gezwollen?	○ Nooit ○ Zelden ○ Soms ○ Vaak ○ Altijd
10.2	Voelt u gekraak of hoort u klikken of een ander vreemd geluid wanneer u de voet/enkel beweegt?	○ Nooit ○ Zelden ○ Soms ○ Vaak ○ Altijd
10.3	Hapert uw enkel of blokkeert uw enkel ('op slot' gaan zitten) wanneer u deze beweegt?	○ Nooit ○ Zelden ○ Soms ○ Vaak ○ Altijd
10.4	Kunt u de voet/enkel volledig strekken?	○ Altijd ○ Vaak ○ Soms ○ Zelden ○ Nooit
10.5	Kunt u de voet volledig naar u toe buigen?	○ Altijd ○ Vaak ○ Soms ○ Zelden ○ Nooit
10.6	In welke mate heeft u een stijf gevoel in de voet/enkel 's ochtends bij het wakker worden?	○ Niet ○ Mild ○ Matig ○ Ernstig ○ Zeer ernstig
10.7	In welke mate heeft u een stijf gevoel in de voet/enkel na zitten, liggen of rusten later op de dag?	○ Niet ○ Mild ○ Matig ○ Ernstig ○ Zeer ernstig
10.8	Hoe vaak heeft u pijn in uw voet/enkel?	○ Nooit ○ Maandelijks ○ Wekelijks ○ Dagelijks ○ Altijd

10.9	Hoeveel voet/enkel pijn heeft u gehad in de afgelopen week bij draaien als uw voet/enkel op de grond staat?	◯ Geen<br>◯ Mild<br>◯ Matig<br>◯ Ernstig<br>◯ Zeer ernstig
10.10	Hoeveel voet/enkel pijn heeft u gehad in de afgelopen week bij het volledig uitstrekken van de voet/enkel?	◯ Geen<br>◯ Mild<br>◯ Matig<br>◯ Ernstig<br>◯ Zeer ernstig
10.11	Hoeveel voet/enkel pijn heeft u gehad in de afgelopen week bij het volledig naar u toe buigen/optrekken van de voet/enkel	◯ Geen<br>◯ Mild<br>◯ Matig<br>◯ Ernstig<br>◯ Zeer ernstig
10.12	Hoeveel voet/enkel pijn heeft u gehad in de afgelopen week bij het lopen op een vlakke ondergrond	◯ Geen<br>◯ Mild<br>◯ Matig<br>◯ Ernstig<br>◯ Zeer ernstig
10.13	Hoeveel voet/enkel pijn heeft u gehad in de afgelopen week bij het trap op- en trap af lopen?	◯ Geen<br>◯ Mild<br>◯ Matig<br>◯ Ernstig<br>◯ Zeer ernstig
10.14	Hoeveel voet/enkel pijn heeft u gehad in de afgelopen week 's nachts in bed?	◯ Geen<br>◯ Mild<br>◯ Matig<br>◯ Ernstig<br>◯ Zeer ernstig
10.15	Hoeveel voet/enkel pijn heeft u gehad in de afgelopen week bij het zitten of liggen?	◯ Geen<br>◯ Mild<br>◯ Matig<br>◯ Ernstig<br>◯ Zeer ernstig

10.16	Hoeveel voet/enkel pijn heeft u gehad in de afgelopen week bij het rechtop staan?	◯ Geen<br>◯ Mild<br>◯ Matig<br>◯ Ernstig<br>◯ Zeer ernstig
10.17	In welke mate werd u gehinderd bij het trap aflopen?	◯ Niet ◯ Mild ◯ Matig ◯ Ernstig ◯ Zeer ernstig
10.18	In welke mate werd u gehinderd bij het trap op lopen?	◯ Niet ◯ Mild ◯ Matig ◯ Ernstig ◯ Zeer ernstig
10.19	In welke mate werd u gehinderd als u vanuit een zittende positie ging staan?	◯ Niet ◯ Mild ◯ Matig ◯ Ernstig ◯ Zeer ernstig
10.20	In welke mate werd u gehinderd bij het staan?	◯ Niet ◯ Mild ◯ Matig ◯ Ernstig ◯ Zeer ernstig
10.21	In welke mate werd u gehinderd bij het naar de grond buigen/iets oprapen?	◯ Niet ◯ Mild ◯ Matig ◯ Ernstig ◯ Zeer ernstig
10.22	In welke mate werd u gehinderd bij het lopen op een vlakke ondergrond?	◯ Niet ◯ Mild ◯ Matig ◯ Ernstig ◯ Zeer ernstig
10.23	In welke mate werd u gehinderd bij het in- en uit de auto stappen?	◯ Niet ◯ Mild ◯ Matig ◯ Ernstig ◯ Zeer ernstig
10.24	In welke mate werd u gehinderd bij het boodschappen doen?	◯ Niet ◯ Mild ◯ Matig ◯ Ernstig ◯ Zeer ernstig
10.25	In welke mate werd u gehinderd bij sokken/panty's aantrekken?	◯ Niet ◯ Mild ◯ Matig ◯ Ernstig ◯ Zeer ernstig
10.26	In welke mate werd u gehinderd bij het opstaan uit bed?	◯ Niet ◯ Mild ◯ Matig ◯ Ernstig ◯ Zeer ernstig
10.27	In welke mate werd u gehinderd bij het sokken uittrekken?	◯ Niet ◯ Mild ◯ Matig ◯ Ernstig ◯ Zeer ernstig
10.28	In welke mate werd u gehinderd bij het in bed liggen (omdraaien, lange tijd uw voet/enkel in dezelfde positie houden)?	◯ Niet ◯ Mild ◯ Matig ◯ Ernstig ◯ Zeer ernstig

10.29	In welke mate werd u gehinderd bij het in/uit bad stappen?	◯ Niet ◯ Mild ◯ Matig ◯ Ernstig ◯ Zeer ernstig
10.30	In welke mate werd u gehinderd bij zitten?	◯ Niet ◯ Mild ◯ Matig ◯ Ernstig ◯ Zeer ernstig
10.31	In welke mate werd u gehinderd bij het toilet op en af gaan?	◯ Niet ◯ Mild ◯ Matig ◯ Ernstig ◯ Zeer ernstig
10.32	In welke mate werd u gehinderd bij zwaar huishoudelijk werk (bijvoorbeeld zware dozen sjouwen, vloer schrobben)?	◯ Niet ◯ Mild ◯ Matig ◯ Ernstig ◯ Zeer ernstig
10.33	In welke mate werd u gehinderd bij licht huishoudeijk werk (bijvoorbeeld koken, afstoffen)?	◯ Niet ◯ Mild ◯ Matig ◯ Ernstig ◯ Zeer ernstig
10.34	In welke mate werd u gehinderd bij hurken?	◯ Niet ◯ Mild ◯ Matig ◯ Ernstig ◯ Zeer ernstig
10.35	In welke mate werd u gehinderd bij hardlopen?	◯ Niet ◯ Mild ◯ Matig ◯ Ernstig ◯ Zeer ernstig
10.36	In welke mate werd u gehinderd bij springen?	◯ Niet ◯ Mild ◯ Matig ◯ Ernstig ◯ Zeer ernstig
10.37	In welke mate werd u gehinderd bij ronddraaien op uw aangedane voet/enkel?	◯ Niet ◯ Mild ◯ Matig ◯ Ernstig ◯ Zeer ernstig
10.38	In welke mate werd u gehinderd bij knielen?	◯ Niet ◯ Mild ◯ Matig ◯ Ernstig ◯ Zeer ernstig
10.39	Hoe vaak bent u zich bewust van uw voet/enkel probleem?	◯ Nooit ◯ Maandelijks ◯ Wekelijks ◯ Dagelijks ◯ Altijd
10.40	Heeft u uw leven veranderd om activiteiten te vermijden die schadelijk kunnen zijn voor uw voet/enkel?	◯ Niet<br>◯ Enigszins<br>◯ Matig<br>◯ Behoorlijk<br>◯ Volledig

10.41	In hoeverre kunt u op uw voet/enkel vertrouwen?	◯ Volledig<br>◯ Behoorlijk<br>◯ Matig<br>◯ Enigzins<br>◯ Niet
10.42	In het algemeen, in welke mate ondervindt u hinder van uw voet/enkel	◯ Geen<br>◯ Mild<br>◯ Matig<br>◯ Ernstig<br>◯ Zeer ernstig

Vragenlijst PRIMA studie 6 weken - Dank

Number	Question	Answers
11.1	Hartelijk dank voor de tijd die u heeft genomen om de vragenlijsten in te vullen. Indien u nog opmerkingen heeft kunt u deze hierin plaatsen.	

BMJ Open

Platelet Rich plasma Injection Management for Ankle osteoarthritis study (PRIMA) Protocol of a Dutch multi-center, stratified, block-randomized, double-blind, placebo-controlled trial

Journal:	*BMJ Open*
Manuscript ID	bmjopen-2019-030961.R2
Article Type:	Protocol
Date Submitted by the Author:	18-Sep-2019
Complete List of Authors:	Paget, Liam; Amsterdam UMC - Locatie AMC, Orthopaedic Surgery; Amsterdam Collaboration for Health and Safety in Sports (ACHSS), AMC/VUmc IOC Research Center<br>Bierma-Zeinstra, Sita; Erasmus University Medical Centre, Department of General Practice; Erasmus Medical Center, Orthopaedic Surgery<br>Goedegebuure, S; OLVG, the Sport Physician Group, Sports Medicine; Academic Center for Evidence-based Sports medicine (ACES), Amsterdam UMC<br>Kerkhoffs, Gino; Amsterdam UMC - Locatie AMC, Orthopaedic Surgery; Amsterdam Collaboration for Health and Safety in Sports (ACHSS), AMC/VUmc IOC Research Center<br>Krips, R; Flevoziekenhuis, Orthopaedic Surgery<br>Maas, Mario; Amsterdam UMC - Locatie AMC, Radiology; Academic Center for Evidence-based Sports medicine (ACES), AmsterdamUMC<br>Moen, Maarten; Bergman Clinics, Sports Medicine; OLVG, the Sport Physician Group<br>Reurink, G; Academic Center for Evidence-based Sports medicine (ACES), Amsterdam UMC; OLVG, the Sport Physician Group, Sports Medicine<br>Stufkens, Sjoerd; Amsterdam UMC - Locatie AMC, Orthopaedic Surgery; Amsterdam Collaboration for Health and Safety in Sports (ACHSS), AMC/VUmc IOC Research Center<br>de Vos, Robert-Jan; Erasmus University Medical Centre, Orthopaedics and Sports Medicine<br>Weir, A; Erasmus University Medical Centre, Orthopaedics and sports medicine; Sports medicine and exercise clinic Haarlem (SBK)<br>Tol, J; Academic Center for Evidence-based Sports medicine (ACES), Amsterdam UMC; Aspetar Orthopaedic and Sports Medicine Hospital
**Primary Subject Heading**:	Sports and exercise medicine
Secondary Subject Heading:	Surgery, Sports and exercise medicine
Keywords:	Foot & ankle < ORTHOPAEDIC & TRAUMA SURGERY, SPORTS MEDICINE, ORTHOPAEDIC & TRAUMA SURGERY, Platelet-rich Plasma, Osteoarthritis, Ankle Osteoarthritis

For peer review only - http://bmjopen.bmj.com/site/about/guidelines.xhtml

SCHOLARONE™
Manuscripts

Platelet Rich plasma Injection Management for Ankle osteoarthritis study (PRIMA)

Protocol of a Dutch multi-center, stratified, block-randomized, double-blind, placebo-controlled trial

Authors: L.D.A. Paget[1,2,3], S.M.A. Bierma – Zeinstra[4,5], S. Goedegebuure[2,6], G.M.M.J. Kerkhoffs[1,2,3], R. Krips[7], M. Maas[3,8,9], M. H. Moen[6,10], G. Reurink[1,2,3,6], S. A. S. Stufkens[1,2,3], R.J. de Vos[5], A. Weir[5,12,13], J.L. Tol[1,2,3,6,11]

1. Department of Orthopaedic Surgery, Amsterdam UMC, University of Amsterdam, Amsterdam Movement Sciences, Amsterdam, the Netherlands.
2. Academic Center for Evidence-based Sports medicine (ACES), Amsterdam UMC, Amsterdam, the Netherlands
3. Amsterdam Collaboration for Health and Safety in Sports (ACHSS), AMC/VUmc IOC Research Center, Amsterdam, the Netherlands
4. Department of General Practice, Erasmus University Medical Center, Rotterdam, The Netherlands
5. Department of Orthopaedics and Sports Medicine, Erasmus University Medical Center, Rotterdam, The Netherlands
6. The Sport Physician Group, Department of Sports Medicine, OLVG, Amsterdam, The Netherlands
7. Department of Orthopaedic Surgery, Flevoziekenhuis, Almere, The Netherlands
8. Department of Musculoskeletal Radiology, The Netherlands
9. Musculoskeletal Imaging Quantification Center (MIQC), The Netherlands.
10. Department of Sports Medicine, Bergman Clinics, Naarden, the Netherlands
11. Aspetar, Orthopaedic and Sports Medicine Hospital, Doha, Qatar
12. Aspetar Sports Groin Pain Centre, Orthopaedic and Sports Medicine Hospital, Doha, Qatar
13. Sport medicine and exercise clinic Haarlem (SBK). Haarlem, The Netherlands.

Correspondence

Liam. D.A. Paget

Department of Orthopaedic Surgery,

Amsterdam UMC

PO Box 22660, 1100 DD Amsterdam

The Netherlands

L.d.paget@amsterdamumc.nl

For peer review only

For peer review only - http://bmjopen.bmj.com/site/about/guidelines.xhtml

ABSTRACT

Introduction

PRP is a potentially efficacious treatment for ankle OA but its use has not been examined in high quality studies. Systematic reviews show that platelet-rich plasma (PRP) injections significantly decrease pain and improve function in knee OA patients. Ankle OA is more common than hip or knee OA in the young active population; with a prevalence of 3.4%.

PRP injections in ankle OA are shown to be safe and improve quality of life over time, but no randomised controlled trial has been conducted. Our randomised controlled trial will evaluate the efficacy of PRP injections for symptom reduction and functional improvement, compared to placebo, in the treatment of ankle (talocrural) OA.

Methods and analysis

We will conduct the Platelet Rich plasma Injection Management for Ankle osteoarthritis (PRIMA) study: a multicentre, randomised, placebo-controlled trial. One hundred patients suffering from ankle OA will be randomised into two treatment groups: PRP injection or placebo (saline) injection. Both groups will receive two injections of PRP or placebo at an interval of 6 weeks. Primary outcome is the American Orthopaedic Foot and Ankle Society (AOFAS) score at 26 weeks. Secondary outcomes determined at several follow-up moments up to 5 years, include Ankle Osteoarthritis Score (AOS), Foot and Ankle Outcome Score (FAOS), pain sub-scale of AOFAS (0-40), VAS score (VAS 0-100), Ankle activity score (AAS 0-10), Subjective patient satisfaction Short form Survey-36 (SF-36), Global Attainment Scaling (GAS) and the EQ-5D-3L utility score. A cost-effectiveness analysis will be performed at 1 year.

Ethics and dissemination

The study is approved by the Medical Ethics Review Committee Amsterdam Medical Center, the Netherlands (ABR 2018-042, approved 23-7-2018) and registered in the Netherlands trial register (NTR 7261). Results and new knowledge will be disseminated through the Dutch Arthritis Association

For peer review only - http://bmjopen.bmj.com/site/about/guidelines.xhtml

(ReumaNederland), Dutch patient federation, conferences and published in a scientific peer-reviewed journal.

Trial registration number

(NTR7261)

For peer review only

For peer review only - http://bmjopen.bmj.com/site/about/guidelines.xhtml

STRENGTH AND LIMITATIONS

- This is the first study to evaluate the efficacy and cost-effectiveness of ankle (talocrural) OA in a multi-center, stratified, block-randomized, double-blind, placebo-controlled trial design.
- A positive study outcome will have a significant effect on widespread availability and nationwide implementation of an intra articular injection treatment for ankle OA with PRP. A negative outcome (no effect of PRP), will prevent the widespread use of a non-efficacious treatment on patients.
- The study population consists only of patients with isolated ankle OA and no concomitant OA in other major joints of the lower extremities that negatively affects their daily activity level
- The PRP used in this randomised controlled trial is an available PRP product that is used in clinical practise. However, there is a multitude of commercial PRP products available that differ in their production method and content, and different treatment regimens regarding dose, timing and number of injections are used in clinical practise. The generalizability to other PRP products and treatment regimens remains unknown.
- The composition of PRP will not be analysed. This decision was based on the fact that PRP preparation and composition has frequently been analysed and is typically not performed in clinical practice prior to injection.

For peer review only - http://bmjopen.bmj.com/site/about/guidelines.xhtml

BACKGROUND AND RATIONALE

PRP is a potentially efficacious treatment for ankle OA but its use has not been examined in high quality studies. Ankle OA is more common than hip or knee OA in the young active population. [1] The incidence of symptomatic ankle OA is estimated to be 3.4% in the general adult population.[1] Patients with ankle OA have a quality of life and physical functioning comparable with hip OA, end-stage kidney disease or congestive heart failure.[2] These relatively young (predominantly female) patients have an increased risk for decreased work participation and family care. The available surgical intervention (arthrodesis) is associated with significant functional limitations. In contrast to hip and knee OA, where joint replacement is an excellent surgical alternative for severe cases, there is a clear need for effective non-surgical interventions in ankle OA.

Platelet Rich Plasma (PRP) is defined as plasma containing a concentration of at least 1,000,000 platelets/µl.[3] Growth factors are stored in α-granules within platelets, and are released in a selective manner upon activation. Growth factors released from the α-granules of platelets are assumed to provide the regenerative benefits of PRP.

Recent reviews concluded that in animal models PRP can diminish multiple inflammatory IL-1 mediated effects.[4] Due to this local anti-inflammatory response, PRP might have an indirect analgesic effect. The second suggested effect might be protection of the cartilage from destructive pro-inflammatory interleukines. This is mediated due to an increased mRNA expression of proteoglycan core protein in the articular cartilage and decreased chondrocyte apoptosis.[4] Consequently, PRP could positively influence the collagen network of the cartilage.

Recent systematic reviews on the intra-articular injection therapy in ankle OA found a lack of evidence from high quality studies to assist in clinical decision-making.[5,6] When compared to placebo injections, hyaluronic acid or corticosteroid injections, PRP injections might significantly decrease

For peer review only - http://bmjopen.bmj.com/site/about/guidelines.xhtml

pain and improve function in knee OA patients.[7–9] Given the clinical effect on pain reduction in knee OA and a good safety profile, PRP is a promising non-surgical therapy for ankle OA.[10,11] PRP might delay the irreversible surgical options like arthrodesis. No significant adverse events have been reported in any PRP trials regarding ankle OA, knee OA, acute hamstring injuries or Achilles tendinopathy. [8,12–17]. Clinical studies on PRP in ankle OA are limited to a single report of 5 cases and a prospective case series of 20 subjects, which both showed safety and significant reduction of pain at a mean of 16 months and 12 weeks follow-up.[14,18] Well-designed prospective Randomised controlled trials (RCT) have not yet been performed.

For peer review only

For peer review only - http://bmjopen.bmj.com/site/about/guidelines.xhtml

METHODS AND DESIGN

Objectives

We aim to determine the efficacy of PRP injections in the management of ankle OA by comparing 2 groups, both receiving 2 injections of either: PRP or placebo solution. We hypothesize that PRP injections are efficacious for symptom reduction and functional improvement compared to placebo in the treatment of ankle (talocrural) OA.

Study Design

The PRIMA study is a multi-center, stratified, block-randomized, double-blind, placebo-controlled trial design will be conducted in order to compare two treatment groups: PRP vs Placebo (saline). After the 26 weeks follow-up of the last patient in the study, the principal investigator and coordinating researcher will be unblinded only after the analysis of the primary outcome. A flow chart of the design and follow-up is shown in figure 1. The study included its first patient in August 2018 and aims to include the last patient by January 2021, consequently allowing analysis and then deblinding to commence after the last follow-up (26 weeks) of the last patient by July 2021.

Patient involvement

Active patient involvement through reviewing the proposal (Reviewer level):

Active involvement of patients during the design phase was performed by 2 patients with ankle OA. Both reviewed the grant proposal and provided feedback.

Execution phase – patients will actively contribute:

Active patient involvement will be secured by invitation of at least 2 patients for the annual trial monitoring and evaluation meetings (Informant level).

For peer review only

For peer review only - http://bmjopen.bmj.com/site/about/guidelines.xhtml

Analysis phase - Patient will review and interpret the results (reviewer level):

Using the blinded codes of the randomisation groups and after breaking the randomisation code, patients (also active in the design phase) will be given the opportunity to interpret the results from a patient perspective.

Dissemination phase - Patient oriented approach (Advisor level)*:*

Results and new knowledge will be disseminated through the Dutch Arthritis Association (ReumaNederland), the Dutch patient federation, conferences and published in a scientific peer-reviewed journal. Dissemination activities will comprise a social media strategy with active patient involvement to share results with online patient communities and an events attendance strategy enabling patient participation in seminars, conferences and workshops.

Fig.1 illustrating the PRIMA multicentre randomized controlled trial design and follow-up procedure

Study Population

Population (base)

Patients with ankle OA in University Medical Centres, teaching hospitals, general hospitals and private specialist clinics will be informed about the study. In order to participate, patients must meet the eligibility criteria documented below.

Inclusion criteria

1. Severity of Ankle OA pain on a visual analogue scale (VAS) (0–100 mm) ≥ 40 mm during daily activities

For peer review only - http://bmjopen.bmj.com/site/about/guidelines.xhtml
For peer review only

2. X-rays (AP and lateral view) indicating ≥ grade 2 talocrural OA on the Van Dijk classification (clarified under section Radiographs)[19]
3. Age ≥ 18 years

Exclusion criteria

1. Patient has received injection therapy for ankle OA in the previous 6 months
2. Patient does not want to receive one of the two therapies
3. Patient has clinical signs of concomitant OA of one or more other major joints of the lower extremities that negatively affects their daily activity level
4. Previous ankle surgery for OA or Osteochondral defects (OCD) < 1 year (not including surgery for an ankle fracture in the past)

Radiographs

AP and lateral X-rays of the talocrural joints will be scored according to the Van Dijk classification:[19]

0) Normal joint or subchondral sclerosis
1) Osteophytes without joint space narrowing
2) Joint space narrowing with or without osteophytes
3) (Sub)total disappearance or deformation of the joint space

Randomisation, Blinding and Treatment Allocation

We will include patients at the centre of their first outpatient clinic appointment. For each patient the coordinating researcher will prepare a syringe with PRP and a syringe with placebo (isotonic saline: 0.9% sodium chloride). A Good Clinical Practice (GCP) approved data management system (Castor EDC, based in Amsterdam, the Netherlands) will be used to perform a computer generated

For peer review only - http://bmjopen.bmj.com/site/about/guidelines.xhtml

block randomisation scheme with patients stratified to centre with a variable block size of 2,4 or 6. This procedure will ensure treatment allocation concealment. The coordinating researcher, treating physician and patient all remain blinded to the allocated intervention. An independent researcher from the coordinating location will have access to the randomization result and the allocated intervention. This will be relayed to a GCP-certified research assistant at the centre. The research assistant then selects one of the two syringes based on the allocated intervention and blinds the syringe with a covering sheath, ensuring concealment of the content of the syringe. The patients, treating physicians, and coordinating researcher will all be blinded to the allocation of the intervention and to the contents of the syringe. The success of blinding will be assessed by asking patients which injection they think they have received just after the injection procedure, this will then be registered accordingly.

For peer review only

After the 26 weeks follow-up of the last patient in the study, the principal investigator and coordinating researcher will be unblinded only after the analysis of the primary outcome. The patients will remain blinded to the therapy until the 52 weeks follow-up (online questionnaire-appendix 1) of the last patient in the study.

Intervention

In this study, patients will be randomised into two treatment groups: PRP injection or placebo saline injection. For each patient the coordinating researcher will prepare a PRP and a placebo injection (isotonic saline: 0.9% sodium chloride). The PRP syringe will be prepared according to the instructions of the manufacturer.[12] Prior to commencement of the study, the coordinating researcher was trained by a representative of Arthrex, as well as two experienced members of the PRIMA trial research group (GR and RJdV) with a vast scientific experience regarding PRP preparation and injection.[12,13] The PRP will be prepared using a widely used and commercially available system (Arthrex double syringe PRP system, Arthrex Medizinische Instrumente GmbH, Garching, Germany).

For peer review only - http://bmjopen.bmj.com/site/about/guidelines.xhtml

One syringe of 15ml autologous blood will be collected twice from the cubital vein: at inclusion and at a time interval of 6 weeks. After blood collection the syringe will undergo 5 minutes of centrifugation and the injection will be given within 30 minutes following venepuncture to prevent formation of blood clots. No additional substances (calcium, thrombin or citrate) will be added to the PRP solution. For each procedure, 2 ml of PRP or placebo will be prepared and injected into the affected ankle joint under ultrasonographic guidance using a sterile technique. All patients will receive a second injection at 6 weeks, regardless of the effect of the first injection. To guarantee blinding for the allocated treatment of the patient, treatment assessor and treating physician, blood will be drawn and both PRP and placebo will be prepared according to exactly the same procedure for each patient (both at inclusion and at a time interval of 6 weeks after the first injection). However, in the control group, patients will be injected with 2ml of physiological saline instead of PRP. An unblinded research assistant will blind one of the two identical syringes based on the allocated intervention using a specially manufactured covering sheath in order to conceal randomization. Following the intra-articular injection, the syringe covered by the sheath (containing either the remnants of the PRP or saline), is handed to the unblinded research assistant, who will dispose of the syringe, therefore keeping the treating physician and coordinating researcher blinded. After the intervention, patients are advised to avoid heavy or repetitive stress to the ankle joint for a period of 48 hours. Furthermore, patients receive a leaflet containing usual-care healthy lifestyle advice beneficial for ankle OA. This includes losing weight and exercise (avoiding heavy or repetitive ankle loading exercises) such as walking, cycling or swimming.

For peer review only

For peer review only - http://bmjopen.bmj.com/site/about/guidelines.xhtml

Use of co-intervention

Patients are instructed to avoid the use of co-interventions and non-steroidal anti-inflammatory drugs if possible, 24 hours prior to the intervention and if possible up to a year after the first injection. Throughout the study, key treatment (including usual care: exercise therapy and healthy life style advice) and co-interventions used by patients will be registered, such as non-steroidal anti-Inflammatory drugs, other analgesic drugs, intra-articular injections or inlays.

Study Procedures

In the event the patient meets the criteria for inclusion and exclusion, the patient will be informed in more detail about the study procedure. At that time the patient can ask questions about the study and decide whether they will participate and sign the informed consent form. Subsequently the patient will proceed to inclusion and the randomization procedure.

Inclusion

Patients are recruited for inclusion by their treating physicians at location. Following inclusion PRP will be prepared according to the PRP system instructions of the manufacturer. During the first two consultations a total of two intra-articular injections will be documented with a time interval of 6 weeks. The patient will have no additional costs to usual care as a result of taking part in this study.

Physical examination will be performed on three occasions up to 26 weeks from baseline. Patient Reported Outcome Measures (PROMs) will be used to evaluate the treatment effect up to five years. A cost-effectivity analysis will be performed at one year using the PRODISQ questionnaire. The time-frame of the follow-up questionnaires is further illustrated in table 1. Questionnaires will be managed and distributed digitally using a GCP approved data management system (Castor EDC)

For peer review only - http://bmjopen.bmj.com/site/about/guidelines.xhtml

Follow-up	
Baseline	- 1st intervention injections<br>- Physical Examination<br>- AOFAS<br>- PROMs<br>- PRODISQ cost-effectivity
6 weeks	- 2nd intervention injection<br>- Physical Examination<br>- AOFAS<br>- PROMs
12 weeks	- AOFAS<br>- PROMs<br>- PRODISQ cost-effectivity
26 weeks	- AOFAS<br>- PROMs<br>- PRODISQ cost-effectivity
39 weeks	- PRODISQ cost-effectivity
52 weeks	- AOFAS<br>- PROMs<br>- PRODISQ cost-effectivity
5 years	- AOFAS<br>- PROMs

Table 1. In addition to the American Orthopaedic Foot and Ankle Society (AOFAS) score, the following PROMs will be taken: Foot and Ankle Outcome Score (FAOS), Ankle Osteoarthritis Score (AOS), Visual Analogue Scale (VAS), Ankle activity score (AAS), Subjective patient satisfaction, Short Form Health Survey (SF-36), The Global Attainment Scaling (GAS), EuroQol-5 dimensions-3 levels (EQ-5D-3L). These PROMs will be elaborated on further on. Furthermore the PROductivity and DISease Questionnaire (PRODISQ) will be used to perform a cost-effectivity analysis. These questionnaires can be found in appendix 1

Replacement of individual patients after withdrawal

In the sample size calculation we compensated for an expected loss of 10% of patients to follow-up. No patients will be replaced after withdrawal.

Safety

For peer review only - http://bmjopen.bmj.com/site/about/guidelines.xhtml
For peer review only

All adverse events reported spontaneously by the patient or observed by the investigator or his staff will be recorded. A variety of conditions have been treated with PRP ranging from muscle and tendon injuries to intra-articular injections of the knee and ankle. To date, no serious adverse events have been documented in the literature, concerning PRP intra-articular injections of the ankle. In accordance with the Central Committee on Research Involving Human Subjects (CCMO) guidelines, this study was classified as low-risk for adverse events. Therefore, the local Medical Ethical Commission will be notified of any serious adverse events. In the event this happens, the advice of the Medical Ethical Commission will be followed accordingly.

Outcome Measures

Primary study parameter/endpoint

The primary objective of this study will be to quantify pain or functional improvement using the American Orthopaedic Foot and Ankle Society (AOFAS) score at 26 weeks follow-up. Studies evaluating the efficacy of PRP in knee OA maintained a follow-up between 3 and 12 months. We therefore opted to take 26 weeks for our primary outcome measure.[10] The AOFAS is a validated scale for ankle OA (0-100) measuring three subdomains (pain, function and alignment) which together total nine items.[20–23] The subdomain of pain is measured by one item where a maximal score of 40 indicates no pain. Function consists of 7 items where full function is indicated by the maximal score of 50 points. Similar to the pain subdomain, alignment has a potential maximum score of 10 points using one item, indicating good alignment.[20,21] The AOFAS questionnaire, having undergone forward and backward translation to Dutch by de Boer et al. 2017, has an excellent internal consistency (Cronbach's α 0.947) and an excellent test-retest reliability (ICC 0.93).[20]

Secondary study parameters/endpoints

Secondary outcome measures are a number of other Patient Reported Outcome Measures (PROMs). Specific time points of the secondary outcome measures can be found in Table 1.

For peer review only - http://bmjopen.bmj.com/site/about/guidelines.xhtml

1. Ankle Osteoarthritis Score (AOS) is a visual analog scale from 0 – 100 mm with 18 questions; 9 relating to pain and 9 relating to disability.
2. Foot and Ankle Outcome Score (FAOS). Each question is assigned 0 – 4 points based on the answer given. The scale runs from 0 (extreme symptoms) to 100 points (no symptoms).
3. In order to evaluate pain, the pain sub-scale of AOFAS (0-40 points) will be analysed. On this scale the lower the score the more pain the patient has. Additionally a VAS score (VAS 0-100 mm) is measured during activities of daily living, with 0 mm being no pain and 100 mm the worst pain imaginable.
4. Total AOFAS score at the other time points than the primary one (at 6, 12 and 52 weeks as well as 5 years).
5. Ankle activity score (0-10 points) is scored according to chart based on the performable activity level
6. Subjective patient satisfaction (4 categories) Poor, Fair, Good, Excellent
7. Short Form Health Survey SF-36 is a health-related quality of life score (0-100 points). The higher the patient scores, the higher the disability.
8. The Global Attainment Scaling (GAS) is a method of scoring based on achievement related to pre-determined goals in agreement with the patient. Points are subtracted for not achieving the pre-defined goals or vice versa. Scores range from 100 (high functioning) to 0 (severely impaired)
9. EuroQol-5 dimensions-3 levels (EQ-5D-3L) utility score allows a patient's health to be defined by a 5-digit number.
10. PROductivity and DISease Questionnaire (PRODISQ) will be used to determine indirect costs and direct costs cost effectivity. The PRODISQ questionnaire is taken at baseline and every 3 months thereafter up until 1 year. This will be done in conjunction with the EQ-5D-3L.

Loss to follow-up

For peer review only - http://bmjopen.bmj.com/site/about/guidelines.xhtml

The coordinating researcher will attempt to limit loss to follow-up as much as possible by contacting every patient and being present at every patient visit. All digital questionnaires will be constantly monitored to ensure they are being filled in and otherwise followed-up by the coordinating researcher. In the event of patient withdrawal, an analysis of demographic and prognostic characteristics will be done on these cases and the remaining patients. As previously described by Järvinen et al. 2014, we will document the patient eligible for and compliant with each follow-up.[24]

For peer review only

For peer review only - http://bmjopen.bmj.com/site/about/guidelines.xhtml

Missing items

Missing items of a score will be handled according to the instructions of the specific scales. In the event of no instructions, we will calculate the percentage of missing items on a scale. Due to the potential impact on trial conclusions, multiple imputation (if more than 10% missing items on a scale) will be applied.

Sample Size

Based on previous and ongoing studies, the study protocol of the randomised controlled trial is designed to detect a difference of 12 points (0-100) on the AOFAS score. There is no official agreement on the minimal clinical important difference for the AOFAS score regarding ankle OA. However in relatable musculoskeletal literature, 10% – 15% of the used scale was reported. [13,25,26] Our pre-defined minimal clinical important difference of 12 % is located within this range.[13,25,26] Based on a previous placebo controlled RCT on injection therapy (hyaluronic acid) in ankle OA of De Groot et al. a standard deviation of 16.3 can be expected.[6] Taking into account a two-sided level of significance of 5%, a power of 90% and a dropout rate of 10%, approximately 50 (45 plus 10% drop out) patients per group will be needed (N=100 in total).

Data Management

After giving permission for participating in this study, patients will receive a link to fill in digital surveys. All data gained outside Castor EDC will be stored on the AMC secured hard drive. All data will be coded and stored in the Castor EDC online database which meets the AMC safety criteria and good clinical practice guidelines. The primary investigator and project leader will safeguard the coded data through password secured access. All patient's data will be archived for at least 15 years and handled with in accordance with the Dutch Personal Data Protection Act (Wbp). Data protections is provided through the safety protocol of Castor EDC with automated backups and SSL security.

Statistical analysis

A standard operating procedure will be available to logically recode and clean the data. The data will be interpreted according to a blinded data interpretation scheme described by Järvinen et al, 2014.[24]

For peer review only - http://bmjopen.bmj.com/site/about/guidelines.xhtml
For peer review only

A statistical expert (SB) is present among the authors. The authors will interpret the statistical results until a consensus is reached. Once the authors are in agreement the two groups will be unblinded and no changes will be made to the interpretation of the results.

Baseline characteristics

Baseline characteristics will be analyzed between groups using descriptive statistics.

Primary outcome measure

Analysis will be performed using an intention to treat approach. To test for the effect of treatment on the between-group difference in primary outcome, we will use a repeated measurement general linear model. Changes from baseline to all follow-up time points will be included in the model. Adjustments will be made for those baseline variables that influenced the primary outcome with $p < 0.10$.

Secondary outcome measures

To test for the effect of treatment on between-group differences in secondary outcomes, we will use the repeated measurement general linear model. Changes from baseline to all follow-up time points will be included in the model.

Economic analysis

In the event of a positive significant outcome, an economic analysis is needed to support a possible change of practice. An economic analysis (costs) will be performed in order to determine cost-effectiveness.

We will assess the differences in mean quality-adjusted life years (QALYs), costs, and net benefits between the PRP injection group and the placebo group using linear models. We express the cost-effectiveness by using cost-effectiveness acceptability curves from both a healthcare perspective and a societal perspective. With multiple bootstrap replicates of the average difference in costs and

For peer review only

For peer review only - http://bmjopen.bmj.com/site/about/guidelines.xhtml

effects in the incremental cost-effectiveness plane we will express the uncertainty of our cost-effectiveness analysis.

The cost-effectivity analysis will be performed with a 1-year time horizon. We use the three-level EQ-5D questionnaire (Euroqol, Rotterdam, the Netherlands) to calculate QALYs as the area under the curve of the utility scores measured over 12 months, according to the Dutch pricing system. The analysis will be based on indirect costs and direct costs and will be determined using the PRODISQ questionnaire. The PRODISQ questionnaire is taken at baseline and every 3 months thereafter up until 1 year.

For peer review only

Monitoring committee

The PRIMA trial will be monitored by the clinical research unit (CRU) of the coordination study centre. The CRU aims to improve the quality of clinical research and ultimately, patient care. Before the PRIMA trial commenced, a monitoring plan was set-up. This Monitoring plan facilitates compliance with the Human Research Act (WMO), Good Clinical Practice (ICH-GCP) guidelines (5.18.1) and/ or ISO14155, which require monitors to verify that (a) the rights and well-being of human subjects are protected,

(b) recorded study data is accurate, complete, and verifiable with the source documents,

(c) the conduct of the study is compliant with the currently approved protocol and with applicable laws regulatory requirements, e.g. WMO, ICH-GCP, ISO14155.

Throughout the study, monitoring will be performed at initiation of the trial, at each participating centre once approximately 3 patients have been enrolled, after approximately 10 – 15 enrolled patients have completed the 26 week follow-up, after 70 patients have been enrolled and after database lock.

For peer review only - http://bmjopen.bmj.com/site/about/guidelines.xhtml

Ethics and Dissemination

The study is approved by the local Medical Ethics Review Committee Amsterdam Medical Center, Amsterdam, the Netherlands (ABR 2018-042, approved 23-7-2018) and is registered in the Netherlands trial register (NTR 7261) in accordance with the World Health Organization Data Set (WHO Data Set). In the events of amendments or other changes, co-researchers will be notified. If relevant patients participating will also be notified. Intellectual data will be the property of the PRIMA study group. Participant data that underline the results reported in this article following de-identification will be shared anonymously on request following publication. Data will be shared, wherever legally and ethically possible and in line with ICMJE guidelines, with researchers who provide a methodologically sound proposal. Data will be stored in a repository (Figshare) under management of the medical library of the AMC. Any proposal should contain: Title, Background, Rationale, Objective, Methods (eligibility criteria, variables and timeframe of interest, statistical plan), Information regarding publication including authorship.

The results of this project study and new knowledge will be disseminated through the Dutch Arthritis Association (ReumaNederland), presentations, news publications, blogs, websites social media and professional organisations (rheumatology, orthopaedics, primary care medicine, sports medicine, public health) and the Dutch patient federation (Patiëntenfederatie Nederland). The patients will also be informed about the results of the study. The study will be conducted according to the principles of the Declaration of Helsinki, GCP and the Medical Research Involving Human Subjects Act (WMO). The board of each participating hospital reviewed and approved the local feasibility. Monitoring and auditing will be carried out throughout the study. In the event of Serious Adverse Events, these will be documented and reported to the monitor and the Medical Ethics board. Eligible patients will be informed of the study and will sign an informed consent form before participating (acquired by researchers associated with the study). Provisions for post-trial care as compensation for those who suffer harm from trial participation is documented in the patient information.

For peer review only

For peer review only - http://bmjopen.bmj.com/site/about/guidelines.xhtml

Discussion

This is the first study evaluating the efficacy and cost-effectiveness of Platelet-rich Plasma injections in the treatment of ankle OA with a multi-center, stratified, block-randomized, double-blind, placebo-controlled design. The primary outcome is the AOFAS score at 26 weeks. Our study will also evaluate the long term efficacy of PRP for up to five years.

Platelet-rich Plasma has been shown to alleviate symptoms of patients suffering from knee OA.[11] In the absence of effective evidence-based non-surgical interventions for ankle OA, PRP would fulfil a clear clinical need. Previous trials concerned with the efficacy of PRP for multiple indications are usually of low quality and adhere to a follow-up of 26 weeks or shorter. Furthermore a cost-effectiveness analysis has never been performed in previous trials on PRP in the treatment of OA.

A positive study outcome would have a significant impact leading to change in clinical practice, where PRP could be offered as a non-surgical intervention. A negative outcome will prevent the widespread use of a non-efficacious treatment.

This study has potential limitations. First of all, due to financial constraints, (semi-quantitative) MRI-defined cartilage degenerative changes will not be used as a secondary outcome score. Furthermore, the composition of PRP isnot analysed. This decision is based on the fact that PRP preparation and composition has frequently been analysed and is typically not performed in clinical practice prior to injection.[27] The PRP we use (Hettich Rotofix32 A centrifuge and Arthrex syringes) is one out of a multitude of commercially available PRP products. All these products differ in their production method and content, and different treatment regimens regarding dose, timing and number of injections are used in clinical practise. The generalizability to other PRP products and treatment regimens remains unknown.

Conclusion

For peer review only - http://bmjopen.bmj.com/site/about/guidelines.xhtml
For peer review only

This will be the first randomized controlled trial to assess the efficacy and cost-effectiveness of PRP in ankle OA.

Contributor ship statement

All authors have been actively involved in creating and writing the study protocol, and all authors critically commented on the paper and gave their final approval of the version that we submitted. *Authorship was and for future publications will be, determined according to the ICMJE authorship recommendations:*

"Substantial contributions to the conception or design of the work; the acquisition, analysis, or interpretation of data for the work;

L.D.A. Paget, S.M.A. Bierma – Zeinstra, S. Goedegebuure, G.M.M.J. Kerkhoffs, R. Krips, M. Maas, M. H. Moen, G. Reurink, S. A. S. Stufkens, R.J. de Vos, A. Weir, J.L. Tol

Drafting the work or revising it critically for important intellectual content;

L.D.A. Paget S.M.A. Bierma – Zeinstra, G.M.M.J. Kerkhoffs, G. Reurink, R.J. de Vos, A. Weir, J.L. Tol

Final approval of the version to be published;

L.D.A. Paget, S.M.A. Bierma – Zeinstra, S. Goedegebuure, G.M.M.J. Kerkhoffs, R. Krips, M. Maas, M. H. Moen, G. Reurink, S. A. S. Stufkens, R.J. de Vos, A. Weir, J.L. Tol

Agreement to be accountable for all aspects of the work in ensuring that questions related to the accuracy or integrity of any part of the work are appropriately investigated and resolved.

L.D.A. Paget, S.M.A. Bierma – Zeinstra, S. Goedegebuure, G.M.M.J. Kerkhoffs, R. Krips, M. Maas, M. H. Moen, G. Reurink, S. A. S. Stufkens, R.J. de Vos, A. Weir, J.L. Tol

For peer review only - http://bmjopen.bmj.com/site/about/guidelines.xhtml
For peer review only

Competing interests

All authors have completed the ICMJE uniform disclosure form at www.icmje.org/coi_disclosure.pdf and declare: no support from any organisation for the submitted work; no financial relationships with any organisations that might have an interest in the submitted work in the previous three years; no other relationships or activities that could appear to have influenced the submitted work.

Funding

This multicenter Randomized Controlled Trial was supported by a grant from the Dutch Arthritis Foundation (ReumaNederland). The Dutch Arthritis Foundation will not, and has not had any role regarding study design, collection, management, analysis, data interpretation or writing of the report. Furthermore, the Dutch Arthritis Foundation does not have authority over report submission or publication.

Figure legends/captions

Fig.1 illustrating the PRIMA multicentre randomized controlled trial design and follow-up procedure

For peer review only

For peer review only - http://bmjopen.bmj.com/site/about/guidelines.xhtml

References

1. Murray C, Marshall M, Rathod T, Bowen CJ, Menz HB, Roddy E. Population prevalence and distribution of ankle pain and symptomatic radiographic ankle osteoarthritis in community dwelling older adults: A systematic review and cross-sectional study. *PLoS One*. 2018. doi:10.1371/journal.pone.0193662

2. Saltzman CL. Impact of Comorbidities on the Measurement of Health in Patients with Ankle Osteoarthritis. *J Bone Jt Surg*. 2006;88(11):2366. doi:10.2106/JBJS.F.00295

3. Marx RE. Platelet-rich plasma (PRP): What is PRP and what is not PRP? *Implant Dent*. 2001. doi:10.1097/00008505-200110000-00002

4. Zhu Y, Yuan M, Meng HY, et al. Basic science and clinical application of platelet-rich plasma forcartilage defects and osteoarthritis: A review. *Osteoarthr Cartil*. 2013;21(11):1627-1637. doi:10.1016/j.joca.2013.07.017

5. Vannabouathong C, Del Fabbro G, Sales B, et al. Intra-articular Injections in the Treatment of Symptoms from Ankle Arthritis: A Systematic Review. *Foot Ankle Int*. 2018:107110071877937. doi:10.1177/1071100718779375

6. DeGroot H, Uzunishvili S, Weir R, Al-omari A, Gomes B. Intra-articular injection of hyaluronic acid is not superior to saline solution injection for ankle arthritis: a randomized, double-blind, placebo-controlled study. *J Bone Joint Surg Am*. 2012;94(1):2-8. doi:10.2106/JBJS.J.01763

7. Meheux CJ, McCulloch PC, Lintner DM, Varner KE, Harris JD. Efficacy of Intra-articular Platelet-Rich Plasma Injections in Knee Osteoarthritis: A Systematic Review. *Arthrosc - J Arthrosc Relat Surg*. 2016;32(3):495-505. doi:10.1016/j.arthro.2015.08.005

8. Laudy ABM, Bakker EWP, Rekers M, Moen MH. Efficacy of platelet-rich plasma injections in osteoarthritis of the knee: a systematic review and meta-analysis. *Br J Sports Med*. 2015;49(10):657-672. doi:10.1136/bjsports-2014-094036

9. Moen M, Weir A, Bakker E, Rekers M, Laudy G. Efficacy of platelet-rich plasma injections in osteoarthritis of the knee: an updated systematic review and meta-analysis. *Osteoarthr Cartil*. 2016;24:S520-S521. doi:10.1016/j.joca.2016.01.953

10. Xing D, Wang B, Zhang W, et al. Intra-articular platelet-rich plasma injections for knee osteoarthritis: An overview of systematic reviews and risk of bias considerations. *Int J Rheum Dis*. 2017;20(11):1612-1630. doi:10.1111/1756-185X.13233

11. Dai WL, Zhou AG, Zhang H, Zhang J. Efficacy of Platelet-Rich Plasma in the Treatment of Knee Osteoarthritis: A Meta-analysis of Randomized Controlled Trials. *Arthrosc - J Arthrosc Relat Surg*. 2017. doi:10.1016/j.arthro.2016.09.024

12. Reurink G, Goudswaard GJ, Moen MH, et al. Platelet-Rich Plasma Injections in Acute

For peer review only

For peer review only - http://bmjopen.bmj.com/site/about/guidelines.xhtml

Muscle Injury. *N Engl J Med*. 2014;370(26):2546-2547. doi:10.1056/NEJMc1402340

13. de Vos RJ, Weir A, van Schie HTM, et al. Platelet-rich plasma injection for chronic Achilles tendinopathy: a randomized controlled trial. *JAMA*. 2010;303(2):144-149. doi:10.1001/jama.2009.1986

14. Fukawa T, Yamaguchi S, Akatsu Y, Yamamoto Y, Akagi R, Sasho T. Safety and Efficacy of Intra-articular Injection of Platelet-Rich Plasma in Patients With Ankle Osteoarthritis. *Foot Ankle Int*. 2017;38(6):596-604. doi:10.1177/1071100717700377

15. Repetto I, Biti B, Cerruti P, Trentini R, Felli L. Conservative Treatment of Ankle Osteoarthritis: Can Platelet-Rich Plasma Effectively Postpone Surgery? *J Foot Ankle Surg*. 2017;56(2):362-365. doi:10.1053/j.jfas.2016.11.015

16. Pas HI, Reurink G, Tol JL, Weir A, Winters M, Moen MH. Efficacy of rehabilitation (lengthening) exercises, platelet-rich plasma injections, and other conservative interventions in acute hamstring injuries: an updated systematic review and meta-analysis. *Br J Sports Med*. 2015;49(18):1197-1205. doi:10.1136/bjsports-2015-094879

17. Hamilton B, Tol JL, Almusa E, et al. Platelet-rich plasma does not enhance return to play in hamstring injuries: a randomised controlled trial. *Br J Sports Med*. 2015;49(14):943-950. doi:10.1136/bjsports-2015-094603

18. Angthong C, Khadsongkram A, Angthong W. Outcomes and quality of life after platelet-rich plasma therapy in patients with recalcitrant hindfoot and ankle diseases: A preliminary report of 12 patients. *J Foot Ankle Surg*. 2013;52(4):475-480. doi:10.1053/j.jfas.2013.04.005

19. van Dijk CN, Verhagen R a, Tol JL. Arthroscopy for problems after ankle fracture. *J Bone Jt Surg*. 1997;79-B(2):280-284. doi:10.1302/0301-620X.79B2.7153

20. De Boer AS, Tjioe RJC, Van Der Sijde F, et al. The American Orthopaedic Foot and Ankle Society Ankle-Hindfoot Scale; Translation and validation of the Dutch language version for ankle fractures. *BMJ Open*. 2017;7(8):1-12. doi:10.1136/bmjopen-2017-017040

21. Kitaoka HB, Alexander IJ, Adelaar RS, Nunley JA, Myerson MS, Sanders M. Clinical rating systems for the ankle-hindfoot, midfoot, hallux, and lesser toes. *Foot ankle Int*. 1994;15(7):349-353. doi:10.1177/107110079401500701

22. Sayyed-Hosseinian S-H, Hassankhani GG, Bagheri F, Alavi N, Shojaie B, Mousavian A. Validation of the Persian Version of the American Orthopedic Foot and Ankle Society Score (AOFAS) Questionnaire. *Arch bone Jt Surg*. 2018;6(3):233-239. doi:10.1097/PHM.0000000000000234

23. Vosoughi AR, Roustaei N, Mahdaviazad H. American Orthopaedic Foot and Ankle Society ankle–hindfoot scale: A cross-cultural adaptation and validation study from Iran. *Foot Ankle Surg*. 2018. doi:10.1016/j.fas.2017.02.007

For peer review only - http://bmjopen.bmj.com/site/about/guidelines.xhtml

For peer review only

573 24. Järvinen TLN, Sihvonen R, Bhandari M, et al. Blinded interpretation of study results can feasibly and effectively diminish interpretation bias. *J Clin Epidemiol*. 2014;67(7):769-772. doi:10.1016/j.jclinepi.2013.11.011

576 25. Pham T, van der Heijde D, Altman RD, et al. OMERACT-OARSI initiative: Osteoarthritis research society international set of responder criteria for osteoarthritis clinical trials revisited. *Osteoarthr Cartil*. 2004. doi:10.1016/j.joca.2004.02.001

579 26. Rozendaal RM, Koes BW, Van Osch GJVM, et al. Effect of glucosamine sulfate on hip osteoarthritis: A randomized trial. *Ann Intern Med*. 2008. doi:10.7326/0003-4819-148-4-200802190-00005

582 27. Oudelaar BW, Peerbooms JC, Huis in 't Veld R, Vochteloo AJH. Concentrations of Blood Components in Commercial Platelet-Rich Plasma Separation Systems: A Review of the Literature. *Am J Sports Med*. 2018. doi:10.1177/0363546517746112

585

For peer review only

For peer review only - http://bmjopen.bmj.com/site/about/guidelines.xhtml

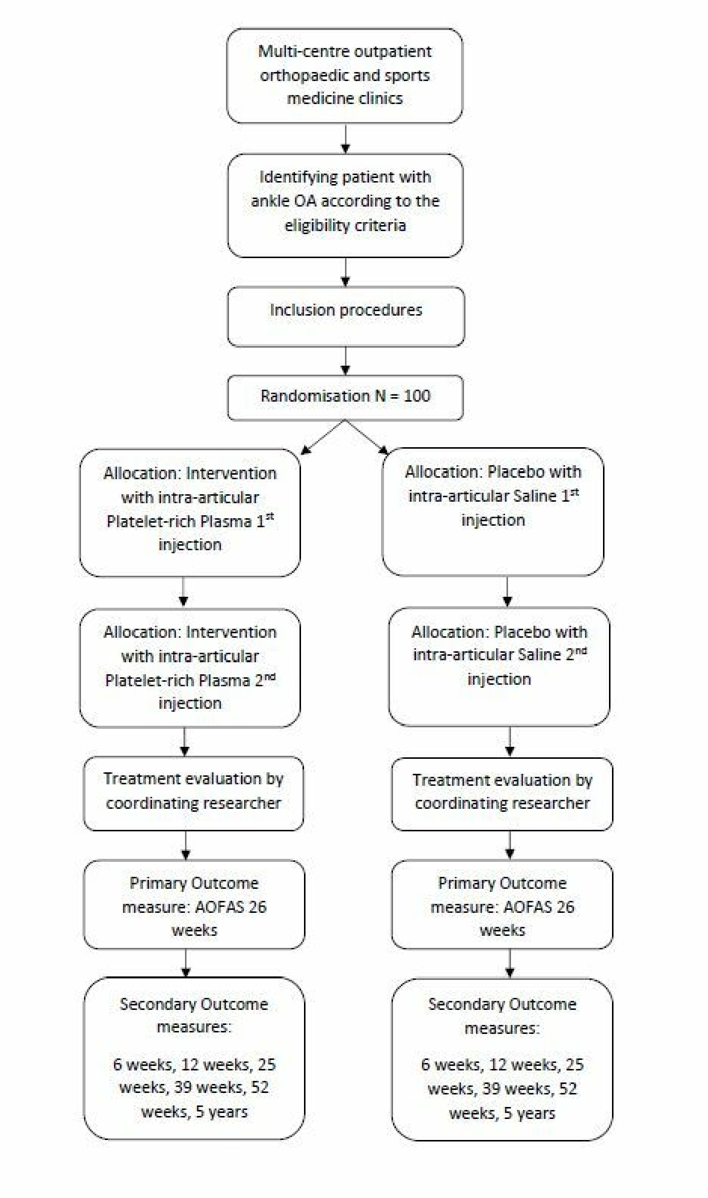

Fig.1 illustrating the PRIMA multicentre randomized controlled trial design and follow-up procedure

39x67mm (300 x 300 DPI)

Surveys of PRIMA trial - version 83.01

Printed on 16-09-2019 11:34:13 by Liam Paget

Survey 'Vragenlijst PRIMA studie'

Vragenlijst PRIMA studie - AOFAS - Pijn

Number	Question	Answers
1.1	Hoeveel pijn ervaart u aan uw voet??	☐ Geen<br>☐ Milde pijnklachten of af en toe pijn<br>☐ Matige pijnklachten, dagelijks<br>☐ Vrijwel continu erge pijn

Vragenlijst PRIMA studie - AOFAS resterende gedeelte

Number	Question	Answers
2.1	Hoeveel beperkingen in het dagelijks leven ervaart u?	○ Geen beperkingen, geen hulpmiddelen nodig<br>○ Geen beperkingen in algemeen dagelijkse bezigheden, wel beperkingen in recreatieve activiteiten (sport, hobbies, etc) geen ondersteuning<br>○ Beperkingen bij dagelijkse- en recreatieve activiteiten, stok<br>○ Ernstige beperkingen bij dagelijkse- en recreatieactiviteiten, walker, krukken, rolstoel, brace
2.2	Wat is de afstand die u in één keer kunt lopen?	○ Meer dan 600 meter<br>○ 400 - 600 meter<br>○ 100 - 300 meter<br>○ Minder dan 100 meter
2.3	Heeft u moeite met lopen op een bepaalde ondergrond?	○ Geen problemen bij lopen<br>○ Enig problemen op ongelijke ondergrond, trap, helling, ladders<br>○ Ernstige problemen bij ongelijke ondergrond, trap, helling, ladders

For peer review only - http://bmjopen.bmj.com/site/about/guidelines.xhtml

2.4	Hoe zou u uw looppatroon beoordelen?	◯ Normaal of minimaal afwijkend<br>◯ Duidelijk<br>◯ Ernstig
2.5	Hoe zou u de beweeglijkheid van uw voet beoordelen ten opzicht van de andere zijde of ten opzicte van vóór het ongeval?	◯ Hetzelfde of licht beperkt t.o.v. de niet aangedane zijde of zoals voor het ongeval<br>◯ Ongeveer de helft beperkt<br>◯ Sterk verminderd, bijna geen bewegingen mogelijk
2.6	Hoe zou u de beweeglijkheid van uw enkel beoordelen ten opzichte van de andere zijde of ten opzichte van voor het ongeval?	◯ Hetzelfde of licht beperkt t.o.v. de niet aangedane zijde of zoals voor het ongeval<br>◯ Ongeveer de helft beperkt<br>◯ Sterk verminderd, bijna geen bewegingen mogelijk
2.7	Hoe zou u de stabiliteit van uw voet en enkel beoordelen	◯ Stabiel, ik verzwik mijn enkel hooguit incidenteel<br>◯ Niet stabiel, ik verzwik mijn enkel vaak
2.8	Hoe zou u de stand van uw voet beoordelen ten opzichte van de andere zijde of ten opzichte van voor het ongeval?	◯ Goed, ik kan mijn voet goed plat op de grond zetten, waarbij de stand niet afwijkend is<br>◯ Ik sta vrijwel alleen op de buitenzijde van mijn voet<br>◯ Ik kan mijn voet niet plat op de grond zetten en loop vrijwel alleen op mijn voorvoet

Vragenlijst PRIMA studie - VAS Pain score

Number	Question	Answers	
3.1	VAS pijnscore (Geef uw minimaal pijnscore aan tijdens dagelijkse activiteiten op een schaal van 0 tot 100, waarbij 0 geen pijn is en 100 de ergste pijn denkbaar is)	(0.00)	100 (100.00)

Vragenlijst PRIMA studie - AAS

Number	Question	Answers

4.1	Wat is het meest bij u van toepassing ten aanzien van uw acitviteit?	☐ American Football<br>☐ Basketbal<br>☐ Gymnastiek/turnen<br>☐ Handbal<br>☐ Rugby<br>☐ Voetbal<br>☐ Hockey<br>☐ Korfbal<br>☐ Gevechtssporten: judo, karate, kung fu, taekwando, jiu jitsu, etc.<br>☐ Oriëntatielopen<br>☐ Rhytmische gymnastiek<br>☐ Volleybal<br>☐ Boxen<br>☐ Freestyle snowboarden<br>☐ Ijshockey<br>☐ Tennis<br>☐ Worstelen<br>☐ Fitness, aerobics<br>☐ Badminton<br>☐ Baseball<br>☐ Cross-country hardlopen<br>☐ Moderne pentathlon<br>☐ Squash<br>☐ Surfen, windsurfen<br>☐ Tafel tennis<br>☐ Atletiek: spring-, werponderdelen<br>☐ Waterskieën<br>☐ Dans<br>☐ Schermen<br>☐ Zaalhockey<br>☐ Bergbeklimmen<br>☐ Langlauf<br>☐ Parachute springen<br>☐ Softball<br>☐ Speciale beroepen en werkactiviteiten; speciale beroepen: ballet, professioneel soldaat, speciale reddingswerker, stuntman, etc.<br>☐ Duiken<br>☐ Scubaduiken<br>☐ Skaten, in-line skaten<br>☐ Atletiek: looponderdelen<br>☐ Triatlon<br>☐ Gewichtsheffen, body-building

- ☐ Alle competitieve sporten hieronder met 'seasonal' conditioning
- ☐ Zwaar fysiek werk
- ☐ Alpine skiën en snowboarden
- ☐ Bowlen/curlen
- ☐ Golf
- ☐ Mountainbike/BMX
- ☐ Powerliften
- ☐ Zeilen
- ☐ Fysiek werk
- ☐ Wielrennen
- ☐ Paardrijden
- ☐ Motorsporten/technische sporten
- ☐ Roeien, kayakken
- ☐ Boogschieten
- ☐ Water polo en zwemmen
- ☐ Kunnen lopen op oneven grond
- ☐ Geen sport, echter geen limitatie in dagelijkse activiteiten
- ☐ Kunnen lopen op even grond, maar de dagelijkste activiteiten zijn gelimiteerd
- ☐ Niet kunnen lopen, fysieke handicap wegens enkelproblemen

4.1.1	***If 'Wat is het meest bij u van toepassing ten aanzien van uw acitviteit?' is equal to 'American Football' answer this question:***<br>American Football	○ Professioneel<br>○ Competitief<br>○ Recreatief
4.1.2	***If 'Wat is het meest bij u van toepassing ten aanzien van uw acitviteit?' is equal to 'Basketbal' answer this question:***<br>Basketbal	○ Professioneel<br>○ Competitief<br>○ Recreatief
4.1.3	***If 'Wat is het meest bij u van toepassing ten aanzien van uw acitviteit?' is equal to 'Gymnastiek/turnen' answer this question:***<br>Gymnastiek/turnen	○ Professioneel<br>○ Competitief<br>○ Recreatief
4.1.4	***If 'Wat is het meest bij u van toepassing ten aanzien van uw acitviteit?' is equal to 'Handbal' answer this question:***<br>Handbal	○ Professioneel<br>○ Competitief<br>○ Recreatief
4.1.5	***If 'Wat is het meest bij u van toepassing ten aanzien van uw acitviteit?' is equal to 'Rugby' answer this question:***<br>Rugby	○ Professioneel<br>○ Competitief<br>○ Recreatief

4.1.6	***If 'Wat is het meest bij u van toepassing ten aanzien van uw acitviteit?' is equal to 'Voetbal' answer this question:***<br>Voetbal	◯ Professioneel<br>◯ Competitief<br>◯ Recreatief
4.1.7	***If 'Wat is het meest bij u van toepassing ten aanzien van uw acitviteit?' is equal to 'Hockey' answer this question:***<br>Hockey	◯ Professioneel<br>◯ Competitief<br>◯ Recreatief
4.1.8	***If 'Wat is het meest bij u van toepassing ten aanzien van uw acitviteit?' is equal to 'Korfbal' answer this question:***<br>Korfbal	◯ Professioneel<br>◯ Competitief<br>◯ Recreatief
4.1.9	***If 'Wat is het meest bij u van toepassing ten aanzien van uw acitviteit?' is equal to 'Gevechtssporten: judo, karate, kung fu, taekwando, jiu jitsu, etc.' answer this question:***<br>Gevechtssporten: judo, karate, kung fu, taekwando, jiu jitsu, etc.	◯ Professioneel<br>◯ Competitief<br>◯ Recreatief
4.1.10	***If 'Wat is het meest bij u van toepassing ten aanzien van uw acitviteit?' is equal to 'Oriëntatielopen' answer this question:***<br>Oriëntatielopen	◯ Professioneel<br>◯ Competitief<br>◯ Recreatief
4.1.11	***If 'Wat is het meest bij u van toepassing ten aanzien van uw acitviteit?' is equal to 'Rhytmische gymnastiek' answer this question:***<br>Rhytmisch gymnastiek	◯ Professioneel<br>◯ Competitief<br>◯ Recreatief
4.1.12	***If 'Wat is het meest bij u van toepassing ten aanzien van uw acitviteit?' is equal to 'Volleybal' answer this question:***<br>Volleybal	◯ Professioneel<br>◯ Competitief<br>◯ Recreatief
4.1.13	***If 'Wat is het meest bij u van toepassing ten aanzien van uw acitviteit?' is equal to 'Boxen' answer this question:***<br>Boxen	◯ Professioneel<br>◯ Competitief<br>◯ Recreatief
4.1.14	***If 'Wat is het meest bij u van toepassing ten aanzien van uw acitviteit?' is equal to 'Freestyle snowboarden' answer this question:***<br>Freestyle snowboarden	◯ Professioneel<br>◯ Competitief<br>◯ Recreatief
4.1.15	***If 'Wat is het meest bij u van toepassing ten aanzien van uw acitviteit?' is equal to 'Freestyle snowboarden' answer this question:***<br>IJshockey	◯ Professioneel<br>◯ Competitief<br>◯ Recreatief

4.1.16	***If 'Wat is het meest bij u van toepassing ten aanzien van uw acitviteit?' is equal to 'Tennis' answer this question:***<br>Tennis	◯ Professioneel<br>◯ Competitief<br>◯ Recreatief
4.1.17	***If 'Wat is het meest bij u van toepassing ten aanzien van uw acitviteit?' is equal to 'Worstelen' answer this question:***<br>Worstelen	◯ Professioneel<br>◯ Competitief<br>◯ Recreatief
4.1.18	***If 'Wat is het meest bij u van toepassing ten aanzien van uw acitviteit?' is equal to 'Fitness, aerobics' answer this question:***<br>Fitness, aerobics	◯ Professioneel<br>◯ Competitief<br>◯ Recreatief
4.1.19	***If 'Wat is het meest bij u van toepassing ten aanzien van uw acitviteit?' is equal to 'Badminton' answer this question:***<br>Badminton	◯ Professioneel<br>◯ Competitief<br>◯ Recreatief
4.1.20	***If 'Wat is het meest bij u van toepassing ten aanzien van uw acitviteit?' is equal to 'Baseball' answer this question:***<br>Baseball	◯ Professioneel<br>◯ Competitief<br>◯ Recreatief
4.1.21	***If 'Wat is het meest bij u van toepassing ten aanzien van uw acitviteit?' is equal to 'Cross-country hardlopen' answer this question:***<br>Cross-country hardlopen	◯ Professioneel<br>◯ Competitief<br>◯ Recreatief
4.1.22	***If 'Wat is het meest bij u van toepassing ten aanzien van uw acitviteit?' is equal to 'Moderne pentathlon' answer this question:***<br>Moderne pentathlon	◯ Professioneel<br>◯ Competitief<br>◯ Recreatief
4.1.23	***If 'Wat is het meest bij u van toepassing ten aanzien van uw acitviteit?' is equal to 'Squash' answer this question:***<br>Squash	◯ Professioneel<br>◯ Competitief<br>◯ Recreatief
4.1.24	***If 'Wat is het meest bij u van toepassing ten aanzien van uw acitviteit?' is equal to 'Surfen, windsurfen' answer this question:***<br>Surfen, windsurfen	◯ Professioneel<br>◯ Competitief<br>◯ Recreatief
4.1.25	***If 'Wat is het meest bij u van toepassing ten aanzien van uw acitviteit?' is equal to 'Tafel tennis' answer this question:***<br>Tafel tennis	◯ Professioneel<br>◯ Competitief<br>◯ Recreatief

4.1.26	***If 'Wat is het meest bij u van toepassing ten aanzien van uw acitviteit?' is equal to 'Atletiek: spring-, werponderdelen' answer this question:*** Atletiek: spring-, werponderdelen	◯ Professioneel ◯ Competitief ◯ Recreatief
4.1.27	***If 'Wat is het meest bij u van toepassing ten aanzien van uw acitviteit?' is equal to 'Waterskieën' answer this question:*** Waterskien	◯ Professioneel ◯ Competitief ◯ Recreatief
4.1.28	***If 'Wat is het meest bij u van toepassing ten aanzien van uw acitviteit?' is equal to 'Dans' answer this question:*** Dans	◯ Professioneel ◯ Competitief ◯ Recreatief
4.1.29	***If 'Wat is het meest bij u van toepassing ten aanzien van uw acitviteit?' is equal to 'Schermen' answer this question:*** Schermen	◯ Professioneel ◯ Competitief ◯ Recreatief
4.1.30	***If 'Wat is het meest bij u van toepassing ten aanzien van uw acitviteit?' is equal to 'Zaalhockey' answer this question:*** Zaalhockey	◯ Professioneel ◯ Competitief ◯ Recreatief
4.1.31	***If 'Wat is het meest bij u van toepassing ten aanzien van uw acitviteit?' is equal to 'Bergbeklimmen' answer this question:*** Bergbeklimmen	◯ Professioneel ◯ Competitief ◯ Recreatief
4.1.32	***If 'Wat is het meest bij u van toepassing ten aanzien van uw acitviteit?' is equal to 'Langlauf' answer this question:*** Langlauf	◯ Professioneel ◯ Competitief ◯ Recreatief
4.1.33	***If 'Wat is het meest bij u van toepassing ten aanzien van uw acitviteit?' is equal to 'Parachute springen' answer this question:*** Parachute springen	◯ Professioneel ◯ Competitief ◯ Recreatief
4.1.34	***If 'Wat is het meest bij u van toepassing ten aanzien van uw acitviteit?' is equal to 'Softball' answer this question:*** Softball	◯ Professioneel ◯ Competitief ◯ Recreatief

4.1.35	***If 'Wat is het meest bij u van toepassing ten aanzien van uw acitviteit?' is equal to 'Speciale beroepen en werkactiviteiten; speciale beroepen: ballet, professioneel soldaat, speciale reddingswerker, stuntman, etc.' answer this question:***<br>Speciale beroepen en werkactiviteiten speciale beroepen: ballet, professioneel soldaat, speciale reddingswerker, stuntman, etc.	○ ballet, professioneel soldaat, speciale reddingswerker, stuntman, etc.
4.1.36	***If 'Wat is het meest bij u van toepassing ten aanzien van uw acitviteit?' is equal to 'Duiken' answer this question:***<br>Duiken	○ Professioneel/competitief<br>○ Recreatief
4.1.37	***If 'Wat is het meest bij u van toepassing ten aanzien van uw acitviteit?' is equal to 'Scubaduiken' answer this question:***<br>Scubaduiken	○ Professioneel/competitief<br>○ Recreatief
4.1.38	***If 'Wat is het meest bij u van toepassing ten aanzien van uw acitviteit?' is equal to 'Skaten, in-line skaten' answer this question:***<br>skaten, in-linen skaten	○ Professioneel/competitief<br>○ Recreatief
4.1.39	***If 'Wat is het meest bij u van toepassing ten aanzien van uw acitviteit?' is equal to 'Atletiek: looponderdelen' answer this question:***<br>Atletiek: looponderdelen	○ Professioneel/competitief<br>○ Recreatief
4.1.40	***If 'Wat is het meest bij u van toepassing ten aanzien van uw acitviteit?' is equal to 'Triatlon' answer this question:***<br>triatlon	○ Professioneel/competitief<br>○ Recreatief
4.1.41	***If 'Wat is het meest bij u van toepassing ten aanzien van uw acitviteit?' is equal to 'Gewichtsheffen, body-building' answer this question:***<br>Gewichtsheffen, body-building	○ Professioneel/competitief<br>○ Recreatief
4.1.42	***If 'Wat is het meest bij u van toepassing ten aanzien van uw acitviteit?' is equal to 'Alle competitieve sporten hieronder met ‘seasonal’ conditioning' answer this question:***<br>Alle competitieve sporten hieronder met ‘seasonal’ conditioning	○ Alle competitieve sporten hieronder met ‘seasonal’ conditioning
4.1.43	***If 'Wat is het meest bij u van toepassing ten aanzien van uw acitviteit?' is equal to 'Zwaar fysiek werk' answer this question:***<br>Zwaar fysiek werk	○ Alle competitieve sporten hieronder met ‘seasonal’ conditioning

For peer review only - http://bmjopen.bmj.com/site/about/guidelines.xhtml

4.1.44	***If 'Wat is het meest bij u van toepassing ten aanzien van uw acitviteit?' is equal to 'Alpine skiën en snowboarden' answer this question:***<br>Alpine skien en snowboarden	◯ Alpine skien en snowboarden
4.1.45	***If 'Wat is het meest bij u van toepassing ten aanzien van uw acitviteit?' is equal to 'Bowlen/curlen' answer this question:***<br>Bowlen/curlen	◯ Bowlen/curlen
4.1.46	***If 'Wat is het meest bij u van toepassing ten aanzien van uw acitviteit?' is equal to 'Golf' answer this question:***<br>Golf	◯ Golf
4.1.47	***If 'Wat is het meest bij u van toepassing ten aanzien van uw acitviteit?' is equal to 'Mountainbike/BMX' answer this question:***<br>Mountainbike/BMX	◯ Mountainbike/BMX
4.1.48	***If 'Wat is het meest bij u van toepassing ten aanzien van uw acitviteit?' is equal to 'Powerliften' answer this question:***<br>Powerliften	◯ Powerliften
4.1.49	***If 'Wat is het meest bij u van toepassing ten aanzien van uw acitviteit?' is equal to 'Zeilen' answer this question:***<br>Zeilen	◯ Zeilen
4.1.50	***If 'Wat is het meest bij u van toepassing ten aanzien van uw acitviteit?' is equal to 'Fysiek werk' answer this question:***<br>Fysiek werk	◯ Fysiek werk
4.1.51	***If 'Wat is het meest bij u van toepassing ten aanzien van uw acitviteit?' is equal to 'Wielrennen' answer this question:***<br>Wielrennen	◯ Wielrennen
4.1.52	***If 'Wat is het meest bij u van toepassing ten aanzien van uw acitviteit?' is equal to 'Paardrijden' answer this question:***<br>Paardrijden	◯ Paardrijden
4.1.53	***If 'Wat is het meest bij u van toepassing ten aanzien van uw acitviteit?' is equal to 'Motorsporten/technische sporten' answer this question:***<br>Motorsporten/technische sporten	◯ Motorsporten/technische sporten
4.1.54	***If 'Wat is het meest bij u van toepassing ten aanzien van uw acitviteit?' is equal to 'Roeien, kayakken' answer this question:***<br>Roeien, kayakken	◯ Roeien, kayakken

For peer review only - http://bmjopen.bmj.com/site/about/guidelines.xhtml

4.1.55	***If 'Wat is het meest bij u van toepassing ten aanzien van uw acitviteit?' is equal to 'Boogschieten' answer this question:*** Boogschieten	◯ Boogschieten
4.1.56	***If 'Wat is het meest bij u van toepassing ten aanzien van uw acitviteit?' is equal to 'Water polo en zwemmen' answer this question:*** Water polo en zwemmen	◯ Water polo en zwemmen
4.1.57	***If 'Wat is het meest bij u van toepassing ten aanzien van uw acitviteit?' is equal to 'Kunnen lopen op oneven grond' answer this question:*** Kunnen lopen op oneven grond	◯ Kunnen lopen op oneven grond
4.1.58	***If 'Wat is het meest bij u van toepassing ten aanzien van uw acitviteit?' is equal to 'Geen sport, echter geen limitatie in dagelijkse activiteiten' answer this question:*** Geen sport, echter geen limitatie in dagelijkse activiteiten	◯ Geen sport, echter geen limitatie in dagelijkse activiteiten
4.1.59	***If 'Wat is het meest bij u van toepassing ten aanzien van uw acitviteit?' is equal to 'Kunnen lopen op even grond, maar de dagelijkste activiteiten zijn gelimiteerd' answer this question:*** Kunnen lopen op even grond, maar de dagelijkste activiteiten zijn gelimiteerd	◯ Kunnen lopen op even grond, maar de dagelijkste activiteiten zijn gelimiteerd
4.1.60	***If 'Wat is het meest bij u van toepassing ten aanzien van uw acitviteit?' is equal to 'Niet kunnen lopen, fysieke handicap wegens enkelproblemen' answer this question:*** Niet kunnen lopen, fysieke handicap wegens enkelproblemen	◯ Niet kunnen lopen, fysieke handicap wegens enkelproblemen

Vragenlijst PRIMA studie - Hoe tevreden bent u over de enkelklachten?

Number	Question	Answers
5.1	Hoe tevreden bent u over de enkelklachten?	◯ Slecht<br>◯ Matig<br>◯ Goed<br>◯ Uitstekend

Vragenlijst PRIMA studie - SF-36

Number	Question	Answers
	Deze vragenlijst gaat over uw standpunten t.a.v. uw gezondheid. Met behulp van deze gegevens kan worden bijgehouden hoe u zich voelt en hoe goed u in staat bent uw gebruikelijke bezigheden uit te voeren. Beantwoord elke vraag door een antwoord aan te klikken. Als u niet zeker weet hoe u een vraag moet beantwoorden, geef dan het best mogelijke antwoord.	
6.1	Hoe zou u over het algemeen uw gezondheid noemen?	◯ Uitstekend ◯ Zeer goed ◯ Goed ◯ Matig ◯ Slecht
6.2	Hoe beoordeelt u nu uw gezondheid over het algemeen vergeleken met een jaar geleden?	◯ Veel beter dan een jaar geleden ◯ Wat beter dan een jaar geleden ◯ Ongeveer hetzelfde als een jaar geleden ◯ Wat slechter dan een jaar geleden ◯ Veel slechter dan een jaar geleden
6.3	Wordt u door uw gezondheid op dit moment beperkt bij forse inspanning, zoals hardlopen, tillen van zware voorwerpen of een veeleisende sport beoefenen?	◯ Ja, ernstig beperkt ◯ Ja, een beetje beperkt ◯ Nee, helemaal niet beperkt
6.4	Wordt u door uw gezondheid op dit moment beperkt bij matige inspanning zoals een tafel verplaatsen, stofzuigen, zwemmen of fietsen?	◯ Ja, ernstig beperkt ◯ Ja, een beetje beperkt ◯ Nee, helemaal niet beperkt
6.5	Wordt u door uw gezondheid op dit moment beperkt bij boodschappen tillen of dragen?	◯ Ja, ernstig beperkt ◯ Ja, een beetje beperkt ◯ Nee, helemaal niet beperkt
6.6	Wordt u door uw gezondheid op dit moment beperkt bij een paar trappen oplopen?	◯ Ja, ernstig beperkt ◯ Ja, een beetje beperkt ◯ Nee, helemaal niet beperkt
6.7	Wordt u door uw gezondheid op dit moment beperkt bij één trap oplopen?	◯ Ja, ernstig beperkt ◯ Ja, een beetje beperkt ◯ Nee, helemaal niet beperkt
6.8	Wordt u door uw gezondheid op dit moment beperkt bij bukken, knielen of hurken?	◯ Ja, ernstig beperkt ◯ Ja, een beetje beperkt ◯ Nee, helemaal niet beperkt
6.9	Wordt u door uw gezondheid op dit moment beperkt bij meer dan één kilometer lopen?	◯ Ja, ernstig beperkt ◯ Ja, een beetje beperkt ◯ Nee, helemaal niet beperkt
6.10	Wordt u door uw gezondheid op dit moment beperkt bij een paar honderd meter lopen?	◯ Ja, ernstig beperkt ◯ Ja, een beetje beperkt ◯ Nee, helemaal niet beperkt

6.11	Wordt u door uw gezondheid op dit moment beperkt bij ongeveer honderd meter lopen?	◯ Ja, ernstig beperkt ◯ Ja, een beetje beperkt ◯ Nee, helemaal niet beperkt
6.12	Wordt u door uw gezondheid op dit moment beperkt bij uzelf wassen of aankleden?	◯ Ja, ernstig beperkt ◯ Ja, een beetje beperkt ◯ Nee, helemaal niet beperkt
6.13	U besteedde in de afgelopen 4 weken minder tijd aan werk of andere bezigheden	◯ Ja ◯ Nee
6.14	U heeft in de afgelopen 4 weken minder bereikt dan u zou willen	◯ Ja ◯ Nee
6.15	U was in de afgelopen 4 weken beperkt in het soort werk of andere bezigheden.	◯ Ja ◯ Nee
6.16	U had de afgelopen 4 weken moeite om uw werk of andere bezigheden uit te voeren (het kostte u bijvoorbeeld extra inspanning).	◯ Ja ◯ Nee
6.17	U besteedde in de afgelopen 4 weken minder tijd aan werk of andere bezigheden ten gevolge van emotionele problemen	◯ Ja ◯ Nee
6.18	U heeft in de afgelopen 4 weken minder bereikt dan u zou willen ten gevolge van emotionele problemen.	◯ Ja ◯ Nee
6.19	U deed de afgelopen 4 weken uw werk of andere bezigheden niet zo zorgvuldig als gewoonlijk ten gevolge van emotionele problemen.	◯ Ja ◯ Nee
6.20	In hoeverre hebben uw lichamelijke gezondheid of emotionele problemen u gedurende de afgelopen 4 weken gehinderd in uw normale omgang met familie, vrienden of buren, of bij activiteiten in groepsverband?	◯ Helemaal niet ◯ Enigszins ◯ Nogal ◯ Veel ◯ Heel erg veel
6.21	Hoeveel lichamelijke pijn heeft u de afgelopen 4 weken gehad?	◯ Geen<br>◯ Heel licht<br>◯ Licht<br>◯ Nogal<br>◯ Ernstig<br>◯ Heel ernstig
6.22	In welke mate bent u de afgelopen 4 weken door de pijn gehinderd in uw normale werk (zowel werk buitenshuis als huishoudelijk werk)?	◯ Helemaal niet<br>◯ Een klein beetje<br>◯ Nogal<br>◯ Veel<br>◯ Heel erg veel

6.23	Voelde u zich levenslustig?	◯ Altijd<br>◯ Meestal<br>◯ Vaak<br>◯ Soms<br>◯ Zelden<br>◯ Nooit
6.24	Was u erg zenuwachtig?	◯ Altijd<br>◯ Meestal<br>◯ Vaak<br>◯ Soms<br>◯ Zelden<br>◯ Nooit
6.25	Zat u zo in de put dat u niets kon?	◯ Altijd<br>◯ Meestal<br>◯ Vaak<br>◯ Soms<br>◯ Zelden<br>◯ Nooit
6.26	Voelde u zich rustig en tevreden?	◯ Altijd<br>◯ Meestal<br>◯ Vaak<br>◯ Soms<br>◯ Zelden<br>◯ Nooit
6.27	Had u veel energie?	◯ Altijd<br>◯ Meestal<br>◯ Vaak<br>◯ Soms<br>◯ Zelden<br>◯ Nooit
6.28	Voelde u zich somber en neerslachtig?	◯ Altijd<br>◯ Meestal<br>◯ Vaak<br>◯ Soms<br>◯ Zelden<br>◯ Nooit

6.29	Voelde u zich uitgeput?	◯ Altijd<br>◯ Meestal<br>◯ Vaak<br>◯ Soms<br>◯ Zelden<br>◯ Nooit
6.30	Was u een gelukkig mens?	◯ Altijd<br>◯ Meestal<br>◯ Vaak<br>◯ Soms<br>◯ Zelden<br>◯ Nooit
6.31	Voelde u zich moe?	◯ Altijd<br>◯ Meestal<br>◯ Vaak<br>◯ Soms<br>◯ Zelden<br>◯ Nooit
6.32	Hoe vaak hebben uw lichamelijke gezondheid of emotionele problemen u gedurende de afgelopen 4 weken gehinderd bij uw sociale activiteiten (zoals vrienden of familie bezoeken etc.)?	◯ Altijd<br>◯ Meestal<br>◯ Soms<br>◯ Zelden<br>◯ Nooit
6.33	Ik lijk wat gemakkelijker ziek te worden dan andere mensen	◯ Volkomen juist<br>◯ Grotendeels juist<br>◯ Weet ik niet<br>◯ Grotendeels onjuist<br>◯ Volkomen onjuist
6.34	Ik ben even gezond als andere mensen die ik ken	◯ Volkomen juist<br>◯ Grotendeels juist<br>◯ Weet ik niet<br>◯ Grotendeels onjuist<br>◯ Volkomen onjuist

6.35	Ik verwacht dat mijn gezondheid achteruit zal gaan	◯ Volkomen juist<br>◯ Grotendeels juist<br>◯ Weet ik niet<br>◯ Grotendeels onjuist<br>◯ Volkomen onjuist
6.36	Mijn gezondheid is uitstekend	◯ Volkomen juist<br>◯ Grotendeels juist<br>◯ Weet ik niet<br>◯ Grotendeels onjuist<br>◯ Volkomen onjuist

Vragenlijst PRIMA studie - EQ-5D-3L

Number	Question	Answers
	Deze vragenlijst gaat over uw standpunten t.a.v. uw gezondheid. Met behulp van deze gegevens kan worden bijgehouden hoe u zich voelt en hoe goed u in staat bent uw gebruikelijke bezigheden uit te voeren. Beantwoord elke vraag door een antwoord aan te klikken. Als u niet zeker weet hoe u een vraag moet beantwoorden, geef dan het best mogelijke antwoord.	
7.1	Hoe is het met uw Mobiliteit gesteld?	◯ Ik heb geen problemen met lopen<br>◯ Ik heb enige problemen met lopen<br>◯ Ik ben bedlegerig
7.2	Hoe is het met uw Zelfzorg gesteld?	◯ Ik heb geen problemen om mijzelf te wassen of aan te kleden<br>◯ Ik heb enige problemen om mijzelf te wassen of aan te kleden<br>◯ Ik ben niet in staat om mijzelf te wassen of aan te kleden
7.3	Hoe is het met de Dagelijkse activiteiten (werk, studie, huishouden, gezins- en vrijetijdsactiviteiten) gesteld?	◯ Ik heb geen problemen met mijn dagelijkse activiteiten<br>◯ Ik heb enige problemen met mijn dagelijkse activiteiten<br>◯ Ik ben niet in staat om mijn dagelijkse activiteiten uit te voeren

7.4	Hoe is het met de Pijn/klachten gesteld?	◯ Ik heb geen pijn of andere klachten<br>◯ Ik heb matige pijn of andere klachten<br>◯ Ik heb zeer ernstige pijn of andere klachten
7.5	Hoe is het met de Stemming gesteld?	◯ Ik ben niet angstig of somber<br>◯ Ik ben matig angstig of somber<br>◯ Ik ben erg angstig of somber
	We willen weten hoe goed of slecht uw gezondheid VANDAAG is. Deze meetschaal loopt van 0 tot 100: 100 staat voor de BESTE gezondheid die u zich kunt voorstellen - 0 staat voor de SLECHTSTE gezondheid die u zich kunt voorstellen	
7.6	Uw Gezondheid vandaag	(0.00) (100.00)

Vragenlijst PRIMA studie - AOS

Number	Question	Answers
	Instructies: De lijn naast elke vraag staat voor hoeveel PIJN u heeft in verschillende situaties. De linker kant (0) is “geen pijn” en de rechter kant (100) is “ergste pijn denkbaar”. Geef voor de onderstaande situaties op de lijn aan hoeveel PIJN u in de afgelopen week in de enkel had. Als een of meerdere van deze situaties niet van toepassing waren, dan kiest u de "niet van toepassing" optie.	
8.1	Wat was de hoogte van de ergste pijn in de afgelopen week?	(0.00) (100.00)
8.2	Heeft u pijn als u 's ochtends opstaat?	◯ Ja<br>◯ Nee<br>◯ Niet van toepassing
8.2.1	***If 'Heeft u pijn als u 's ochtends opstaat?' is equal to 'Ja' answer this question:***<br>Hoeveel pijn heeft u voordat u 's ochtends opstaat?	(0.00) (100.00)
8.3	Heeft u pijn wanneer u op blote voeten loopt?	◯ Ja<br>◯ Nee<br>◯ Niet van toepassing
8.3.1	***If 'Heeft u pijn wanneer u op blote voeten loopt?' is equal to 'Ja' answer this question:***<br>Hoeveel pijn heeft u als u op blote voeten loopt?	(0.00) (100.00)

8.4	Heeft u pijn wanneer u op blote voeten staat?	◯ Ja<br>◯ Nee<br>◯ Niet van toepassing	
8.4.1	***If 'Heeft u pijn wanneer u op blote voeten staat?' is equal to 'Ja' answer this question:***<br>Hoeveel pijn heeft u als u op blote voeten staat?	(0.00)	(100.00)
8.5	Heeft u pijn wanneer u met schoenen loopt?	◯ Ja<br>◯ Nee<br>◯ Niet van toepassing	
8.5.1	***If 'Heeft u pijn wanneer u met schoenen loopt?' is equal to 'Ja' answer this question:***<br>Hoeveel pijn heeft u wanneer u met schoenen loopt?	(0.00)	(100.00)
8.6	Heeft u pijn wanneer u met schoenen staat?	◯ Ja<br>◯ Nee<br>◯ Niet van toepassing	
8.6.1	***If 'Heeft u pijn wanneer u met schoenen staat?' is equal to 'Ja' answer this question:***<br>Hoeveel pijn heeft u wanneer u met schoenen staat?	(0.00)	(100.00)
8.7	Heeft u pijn wanneer u loopt met steunzolen of een brace?	◯ Ja<br>◯ Nee<br>◯ Niet van toepassing	
8.7.1	***If 'Heeft u pijn wanneer u loopt met steunzolen of een brace?' is equal to 'Ja' answer this question:***<br>Hoeveel pijn heeft u wanneer u loopt met steunzolen of een brace?	(0.00)	(100.00)
8.8	Heeft u pijn wanneer u staat met steunzolen of een brace?	◯ Ja<br>◯ Nee<br>◯ Niet van toepassing	
8.8.1	***If 'Heeft u pijn wanneer u staat met steunzolen of een brace?' is equal to 'Ja' answer this question:***<br>Hoeveel pijn heeft u wanneer u staat met steunzolen of een brace?	(0.00)	(100.00)
8.9	Heeft u pijn aan het einde van de dag?	◯ Ja<br>◯ Nee<br>◯ Niet van toepassing	

8.9.1	***If 'Heeft u pijn aan het einde van de dag?' is equal to 'Ja' answer this question:***<br>Hoeveel pijn heeft u aan het einde van de dag?	(0.00)	(100.00)
	Instructies:De lijn naast elke vraag staat voor hoeveel MOEITE u heeft met verschillende activiteiten. De linker kant (0) is “Niet moeilijk” en de rechter kant (100) is “Te moeilijk, niet uitvoerbaar”. Geef voor de onderstaande activiteiten op de lijn aan hoeveel MOEITE u in de afgelopen week door enkelklachten had met onderstaande activiteiten. Als een situatie niet van toepassing is, kies dan de optie "niet van toepassing".		
8.10	Heeft u moeite met door het huis lopen?	◯ Ja<br>◯ Nee<br>◯ Niet van toepassing	
8.10.1	***If 'Heeft u moeite met door het huis lopen?' is equal to 'Ja' answer this question:***<br>Hoeveel moeite heeft u met door het huis lopen?	(0.00)	(100.00)
8.11	Heeft u moeite met buiten lopen op oneven ondergrond?	◯ Ja<br>◯ Nee<br>◯ Niet van toepassing	
8.11.1	***If 'Heeft u moeite met buiten lopen op oneven ondergrond?' is equal to 'Ja' answer this question:***<br>Hoeveel moeite heeft u met buiten lopen op oneven ondergrond?	(0.00)	(100.00)
8.12	Heeft u moeite met een paar honderd meter lopen?	◯ Ja<br>◯ Nee<br>◯ Niet van toepassing	
8.12.1	***If 'Heeft u moeite met een paar honderd meter lopen?' is equal to 'Ja' answer this question:***<br>Hoeveel moeite heeft u met een paar honderd meter lopen?	(0.00)	(100.00)
8.13	Heeft u moeite met een trap oplopen?	◯ Ja<br>◯ Nee<br>◯ Niet van toepassing	
8.13.1	***If 'Heeft u moeite met een trap oplopen?' is equal to 'Ja' answer this question:***<br>Hoeveel moeite heeft u met een trap oplopen?	(0.00)	(100.00)
8.14	Heeft u moeite met een trap aflopen?	◯ Ja<br>◯ Nee<br>◯ Niet van toepassing	

8.14.1	***If 'Heeft u moeite met een trap aflopen?' is equal to 'Ja' answer this question:*** Hoeveel moeite heeft u met een trap aflopen?	(0.00)	(100.00)
8.15	Heeft u moeite met op de tenen staan?	◯ Ja<br>◯ Nee<br>◯ Niet van toepassing	
8.15.1	***If 'Heeft u moeite met op de tenen staan?' is equal to 'Ja' answer this question:*** Hoeveel moeite heeft u met op de tenen staan?	(0.00)	(100.00)
8.16	Heeft u moeite met opstaan uit de stoel?	◯ Ja<br>◯ Nee<br>◯ Niet van toepassing	
8.16.1	***If 'Heeft u moeite met opstaan uit de stoel?' is equal to 'Ja' answer this question:*** Hoeveel moeite heeft u met opstaan uit de stoel?	(0.00)	(100.00)
8.17	Heeft u moeite met het op- of afstappen van stoepranden?	◯ Ja<br>◯ Nee<br>◯ Niet van toepassing	
8.17.1	***If 'Heeft u moeite met het op- of afstappen van stoepranden?' is equal to 'Ja' answer this question:*** Hoeveel moeite heeft u met het op- of afstappen van stoepranden?	(0.00)	(100.00)
8.18	Heeft u moeite met snel lopen of rennen?	◯ Ja<br>◯ Nee<br>◯ Niet van toepassing	
8.18.1	***If 'Heeft u moeite met snel lopen of rennen?' is equal to 'Ja' answer this question:*** Hoeveel moeite heeft u met snel lopen of rennen?	(0.00)	(100.00)

Vragenlijst PRIMA studie - FAOS

Number	Question	Answers

Deze lijst vraagt naar uw mening over uw voet/enkel. Uw antwoorden geven ons een beeld van uw voet/enkel klachten en hoe u in staat bent om alledaagse activiteiten uit te voeren in uw huidige situatie. Beantwoorden van een vraag doet u door het aanklikken van een vakje met het volgens u meest juiste antwoord (één vakje per vraag). Als u niet zeker weet hoe u een vraag moet beantwoorden, geeft u dan het antwoord dat volgens u het meest op uw situatie van toepassing is. Deze vraag heeft betrekking op het voorkomen van voet/enkel klachten in de afgelopen week.

9.1	Is uw voet/enkel gezwollen?	◯ Nooit ◯ Zelden ◯ Soms ◯ Vaak ◯ Altijd
9.2	Voelt u gekraak of hoort u klikken of een ander vreemd geluid wanneer u de voet/enkel beweegt?	◯ Nooit ◯ Zelden ◯ Soms ◯ Vaak ◯ Altijd
9.3	Hapert uw enkel of blokkeert uw enkel ('op slot' gaan zitten) wanneer u deze beweegt?	◯ Nooit ◯ Zelden ◯ Soms ◯ Vaak ◯ Altijd
9.4	Kunt u de voet/enkel volledig strekken?	◯ Altijd ◯ Vaak ◯ Soms ◯ Zelden ◯ Nooit
9.5	Kunt u de voet volledig naar u toe buigen?	◯ Altijd ◯ Vaak ◯ Soms ◯ Zelden ◯ Nooit
9.6	In welke mate heeft u een stijf gevoel in de voet/enkel 's ochtends bij het wakker worden?	◯ Niet ◯ Mild ◯ Matig ◯ Ernstig ◯ Zeer ernstig
9.7	In welke mate heeft u een stijf gevoel in de voet/enkel na zitten, liggen of rusten later op de dag?	◯ Niet ◯ Mild ◯ Matig ◯ Ernstig ◯ Zeer ernstig
9.8	Hoe vaak heeft u pijn in uw voet/enkel?	◯ Nooit ◯ Maandelijks ◯ Wekelijks ◯ Dagelijks ◯ Altijd
9.9	Hoeveel voet/enkel pijn heeft u gehad in de afgelopen week bij draaien als uw voet/enkel op de grond staat?	◯ Geen<br>◯ Mild<br>◯ Matig<br>◯ Ernstig<br>◯ Zeer ernstig
9.10	Hoeveel voet/enkel pijn heeft u gehad in de afgelopen week bij het volledig uitstrekken van de voet/enkel?	◯ Geen<br>◯ Mild<br>◯ Matig<br>◯ Ernstig<br>◯ Zeer ernstig

9.11	Hoeveel voet/enkel pijn heeft u gehad in de afgelopen week bij het volledig naar u toe buigen/optrekken van de voet/enkel	◯ Geen<br>◯ Mild<br>◯ Matig<br>◯ Ernstig<br>◯ Zeer ernstig
9.12	Hoeveel voet/enkel pijn heeft u gehad in de afgelopen week bij het lopen op een vlakke ondergrond	◯ Geen<br>◯ Mild<br>◯ Matig<br>◯ Ernstig<br>◯ Zeer ernstig
9.13	Hoeveel voet/enkel pijn heeft u gehad in de afgelopen week bij het trap op- en trap af lopen?	◯ Geen<br>◯ Mild<br>◯ Matig<br>◯ Ernstig<br>◯ Zeer ernstig
9.14	Hoeveel voet/enkel pijn heeft u gehad in de afgelopen week 's nachts in bed?	◯ Geen<br>◯ Mild<br>◯ Matig<br>◯ Ernstig<br>◯ Zeer ernstig
9.15	Hoeveel voet/enkel pijn heeft u gehad in de afgelopen week bij het zitten of liggen?	◯ Geen<br>◯ Mild<br>◯ Matig<br>◯ Ernstig<br>◯ Zeer ernstig
9.16	Hoeveel voet/enkel pijn heeft u gehad in de afgelopen week bij het rechtop staan?	◯ Geen<br>◯ Mild<br>◯ Matig<br>◯ Ernstig<br>◯ Zeer ernstig
9.17	In welke mate werd u gehinderd bij het trap aflopen?	◯ Niet ◯ Mild ◯ Matig ◯ Ernstig ◯ Zeer ernstig
9.18	In welke mate werd u gehinderd bij het trap op lopen?	◯ Niet ◯ Mild ◯ Matig ◯ Ernstig ◯ Zeer ernstig

9.19	In welke mate werd u gehinderd als u vanuit een zittende positie ging staan?	○ Niet ○ Mild ○ Matig ○ Ernstig ○ Zeer ernstig
9.20	In welke mate werd u gehinderd bij het staan?	○ Niet ○ Mild ○ Matig ○ Ernstig ○ Zeer ernstig
9.21	In welke mate werd u gehinderd bij het naar de grond buigen/iets oprapen?	○ Niet ○ Mild ○ Matig ○ Ernstig ○ Zeer ernstig
9.22	In welke mate werd u gehinderd bij het lopen op een vlakke ondergrond?	○ Niet ○ Mild ○ Matig ○ Ernstig ○ Zeer ernstig
9.23	In welke mate werd u gehinderd bij het in- en uit de auto stappen?	○ Niet ○ Mild ○ Matig ○ Ernstig ○ Zeer ernstig
9.24	In welke mate werd u gehinderd bij het boodschappen doen?	○ Niet ○ Mild ○ Matig ○ Ernstig ○ Zeer ernstig
9.25	In welke mate werd u gehinderd bij sokken/panty's aantrekken?	○ Niet ○ Mild ○ Matig ○ Ernstig ○ Zeer ernstig
9.26	In welke mate werd u gehinderd bij het opstaan uit bed?	○ Niet ○ Mild ○ Matig ○ Ernstig ○ Zeer ernstig
9.27	In welke mate werd u gehinderd bij het sokken uittrekken?	○ Niet ○ Mild ○ Matig ○ Ernstig ○ Zeer ernstig
9.28	In welke mate werd u gehinderd bij het in bed liggen (omdraaien, lange tijd uw voet/enkel in dezelfde positie houden)?	○ Niet ○ Mild ○ Matig ○ Ernstig ○ Zeer ernstig
9.29	In welke mate werd u gehinderd bij het in/uit bad stappen?	○ Niet ○ Mild ○ Matig ○ Ernstig ○ Zeer ernstig
9.30	In welke mate werd u gehinderd bij zitten?	○ Niet ○ Mild ○ Matig ○ Ernstig ○ Zeer ernstig
9.31	In welke mate werd u gehinderd bij het toilet op en af gaan?	○ Niet ○ Mild ○ Matig ○ Ernstig ○ Zeer ernstig

9.32	In welke mate werd u gehinderd bij zwaar huishoudelijk werk (bijvoorbeeld zware dozen sjouwen, vloer schrobben)?	◯ Niet ◯ Mild ◯ Matig ◯ Ernstig ◯ Zeer ernstig
9.33	In welke mate werd u gehinderd bij licht huishoudeijk werk (bijvoorbeeld koken, afstoffen)?	◯ Niet ◯ Mild ◯ Matig ◯ Ernstig ◯ Zeer ernstig
9.34	In welke mate werd u gehinderd bij hurken?	◯ Niet ◯ Mild ◯ Matig ◯ Ernstig ◯ Zeer ernstig
9.35	In welke mate werd u gehinderd bij hardlopen?	◯ Niet ◯ Mild ◯ Matig ◯ Ernstig ◯ Zeer ernstig
9.36	In welke mate werd u gehinderd bij springen?	◯ Niet ◯ Mild ◯ Matig ◯ Ernstig ◯ Zeer ernstig
9.37	In welke mate werd u gehinderd bij ronddraaien op uw aangedane voet/enkel?	◯ Niet ◯ Mild ◯ Matig ◯ Ernstig ◯ Zeer ernstig
9.38	In welke mate werd u gehinderd bij knielen?	◯ Niet ◯ Mild ◯ Matig ◯ Ernstig ◯ Zeer ernstig
9.39	Hoe vaak bent u zich bewust van uw voet/enkel probleem?	◯ Nooit ◯ Maandelijks ◯ Wekelijks ◯ Dagelijks ◯ Altijd
9.40	Heeft u uw leven veranderd om activiteiten te vermijden die schadelijk kunnen zijn voor uw voet/enkel?	◯ Niet<br>◯ Enigszins<br>◯ Matig<br>◯ Behoorlijk<br>◯ Volledig
9.41	In hoeverre kunt u op uw voet/enkel vertrouwen?	◯ Volledig<br>◯ Behoorlijk<br>◯ Matig<br>◯ Enigzins<br>◯ Niet
9.42	In het algemeen, in welke mate ondervindt u hinder van uw voet/enkel	◯ Geen<br>◯ Mild<br>◯ Matig<br>◯ Ernstig<br>◯ Zeer ernstig

Vragenlijst PRIMA studie - PRODISQ - Gezondheid en werk

Number	Question	Answers
10.1	Wat is uw leeftijd?	[ ] jaar
10.2	Wat is uw geslacht?	○ Man<br>○ Vrouw
10.3	Wat is de hoogste opleiding die u heeft afgemaakt (Zoek uw hoogste opleiding en kruis het hokje daarvoor aan)?	☐ Ik heb geen school of opleiding afgemaakt<br>☐ Lagere school of basisschool<br>☐ Huishoudschool, vbo, lbo, lts, leao of lhno<br>☐ Mavo, mulo, ivo of vmbo<br>☐ Mbo, mts, meao, mhno, inas of intas<br>☐ Havo, vwo, hbs, mms, atheneum of gymnasium<br>☐ Hbo, hts, heao of hhno<br>☐ Universiteit<br>☐ Ik heb een andere opleiding afgemaakt, namelijk
10.3.1	***If 'Wat is de hoogste opleiding die u heeft afgemaakt (Zoek uw hoogste opleiding en kruis het hokje daarvoor aan)?' is equal to 'Ik heb een andere opleiding afgemaakt, namelijk' answer this question:***<br>Wat is de hoogste opleiding die u heeft afgemaakt?	[ ]
10.4	Wat doet u in het dagelijks leven?	☐ Ik zit op school, ik studeer<br>☐ Ik werk in loondienst<br>☐ Ik ben zelfstandig ondernemer<br>☐ Ik ben huisvrouw, huisman<br>☐ Ik ben werkloos<br>☐ Ik ben arbeidsongeschikt<br>☐ Ik ben met pensioen of prepensioen<br>☐ Ik doe iets anders, namelijk
10.4.1	***If 'Wat doet u in het dagelijks leven?' is equal to 'Ik ben arbeidsongeschikt' answer this question:***<br>Wat doet u in het dagelijks leven?	[ ]

10.4.2	***If 'Wat doet u in het dagelijks leven?' is equal to 'Ik doe iets anders, namelijk' answer this question:*** Wat doet u in het dagelijks leven?	
10.5	Hebt u betaald werk?	○ Nee<br>○ Ja
10.5.1	***If 'Hebt u betaald werk?' is equal to 'Ja' answer this question:*** Wat is uw beroep?	
10.5.2	***If 'Hebt u betaald werk?' is equal to 'Ja' answer this question:*** Hoeveel uur per week werkt u (Tel alleen de uren waarvoor u betaald wordt)?	uren
10.5.3	***If 'Hebt u betaald werk?' is equal to 'Ja' answer this question:*** Hoeveel dagen in de week werkt u?	Dagen
10.5.4	***If 'Hebt u betaald werk?' is equal to 'Ja' answer this question:*** Bent u in de afgelopen 4 weken afwezig geweest van uw werk omdat u ziek was?	○ Nee<br>○ Ja
10.5.4.1	***If 'Bent u in de afgelopen 4 weken afwezig geweest van uw werk omdat u ziek was?' is equal to 'Ja' answer this question:*** Bent u in de afgelopen 4 weken afwezig geweest van uw werk omdat u ziek was (Tel alleen de werkdagen in de afgelopen 4 weken)?	dagen afwezig geweest
10.5.4.2	***If 'Bent u in de afgelopen 4 weken afwezig geweest van uw werk omdat u ziek was?' is equal to 'Ja' answer this question:*** Was u langer dan de gehele periode van 4 weken afwezig van uw werk doordat u ziek was?	○ Nee<br>○ Ja
10.5.4.2.1	***If 'Was u langer dan de gehele periode van 4 weken afwezig van uw werk doordat u ziek was?' is equal to 'Ja' answer this question:*** Wanneer heeft u zich ziek gemeld?	*(dd-mm-yyyy)*
10.5.5	***If 'Hebt u betaald werk?' is equal to 'Ja' answer this question:*** Waren er in de afgelopen 4 weken, dagen waarop u wel gewerkt heeft, maar tijdens uw werk last had van lichamelijke of psychische problemen?	○ Ja<br>○ Nee

10.5.5.1	***If 'Waren er in de afgelopen 4 weken, dagen waarop u wel gewerkt heeft, maar tijdens uw werk last had van lichamelijke of psychische problemen?' is equal to 'Ja' answer this question:*** Op hoeveel werkdagen had u tijdens uw werk last van uw lichamelijke of psychische problemen?	______ werkdagen
10.5.5.2	***If 'Waren er in de afgelopen 4 weken, dagen waarop u wel gewerkt heeft, maar tijdens uw werk last had van lichamelijke of psychische problemen?' is equal to 'Ja' answer this question:*** Op de dagen dat u last had, kon u misschien niet zoveel werk doen als normaal. Hoeveel werk kon u op deze dagen gemiddeld doen?	Ik kon op deze dagen niks doen (0.00) … Ik kon net zoveel doen als normaal (10.00)
10.6	Waren er dagen waarop u minder onbetaald werk kon doen door uw lichamelijke of psychische problemen?	◯ Nee<br>◯ Ja
10.6.1	***If 'Waren er dagen waarop u minder onbetaald werk kon doen door uw lichamelijke of psychische problemen?' is equal to 'Ja' answer this question:*** Op hoeveel dagen was dit zo?	______ Dagen
10.6.2	***If 'Waren er dagen waarop u minder onbetaald werk kon doen door uw lichamelijke of psychische problemen?' is equal to 'Ja' answer this question:*** Stel dat iemand, bijvoorbeeld uw partner, familielid of een bekende, u op deze dagen had geholpen. En al het onbetaalde werk wat u niet kon doen, voor u had gedaan. Hoeveel uur was die persoon hier op deze dagen dan gemiddeld mee bezig geweest?	______ uur op deze dagen

Vragenlijst PRIMA studie - PRODISQ - Zorggebruik

Number	Question	Answers
	Wij willen graag weten met welke dokters u in de afgelopen 3 maanden een afspraak had. Het gaat om afspraken voor uzelf. Ook andere zorgverleners tellen mee. Bijvoorbeeld de fysiotherapeut.	
11.1	Bent u in de afgelopen 3 maanden naar uw huisarts geweest?	◯ Nee<br>◯ Ja
11.1.1	***If 'Bent u in de afgelopen 3 maanden naar uw huisarts geweest?' is equal to 'Ja' answer this question:*** Hoeveel afspraken had u?	______ afspraken

11.2	Bent u in de afgelopen 3 maanden in contact geweest met een maatschappelijk werker?	◯ Nee<br>◯ Ja
11.2.1	***If 'Bent u in de afgelopen 3 maanden in contact geweest met een maatschappelijk werker?' is equal to 'Ja' answer this question:***<br>Hoeveel afspraken had u?	[ ] afspraken
11.3	Bent u in de afgelopen 3 maanden naar een fysiotherapeut geweest? Of een caesartherapeut, therapeut mensendieck of een manueel therapeut?	◯ Nee<br>◯ Ja
11.3.1	***If 'Bent u in de afgelopen 3 maanden naar een fysiotherapeut geweest? Of een caesartherapeut, therapeut mensendieck of een manueel therapeut?' is equal to 'Ja' answer this question:***<br>Hoeveel afspraken had u?	[ ] afspraken
11.4	Bent u in de afgelopen 3 maanden naar een ergotherapeut geweest?	◯ Nee<br>◯ Ja
11.4.1	***If 'Bent u in de afgelopen 3 maanden naar een ergotherapeut geweest?' is equal to 'Ja' answer this question:***<br>Hoeveel afspraken had u?	[ ] afspraken
11.5	Bent u in de afgelopen 3 maanden naar een logopedist geweest?	◯ Nee<br>◯ Ja
11.5.1	***If 'Bent u in de afgelopen 3 maanden naar een logopedist geweest?' is equal to 'Ja' answer this question:***<br>Hoeveel afspraken had u?	[ ] afspraken
11.6	Bent u in de afgelopen 3 maanden naar een diëtist geweest?	◯ Nee<br>◯ Ja
11.6.1	***If 'Bent u in de afgelopen 3 maanden naar een diëtist geweest?' is equal to 'Ja' answer this question:***<br>Hoeveel afspraken had u?	[ ] afspraken
11.7	Bent u in de afgelopen 3 maanden naar een homeopaat geweest? Of een acupuncturist?	◯ Nee<br>◯ Ja
11.7.1	***If 'Bent u in de afgelopen 3 maanden naar een homeopaat geweest? Of een acupuncturist?' is equal to 'Ja' answer this question:***<br>Hoeveel afspraken had u?	[ ] afspraken

For peer review only - http://bmjopen.bmj.com/site/about/guidelines.xhtml

11.8	Bent u in de afgelopen 3 maanden naar een psycholoog geweest? Of een psychotherapeut of psychiater?	◯ Nee<br>◯ Ja
11.8.1	***If 'Bent u in de afgelopen 3 maanden naar een psycholoog geweest? Of een psychotherapeut of psychiater?' is equal to 'Ja' answer this question:***<br>Hoeveel afspraken had u?	[ ] afspraken
11.9	Heeft u in de afgelopen 3 maanden afspraken gehad met de bedrijfsarts?	◯ Nee<br>◯ Ja
11.9.1	***If 'Heeft u in de afgelopen 3 maanden afspraken gehad met de bedrijfsarts?' is equal to 'Ja' answer this question:***<br>Hoeveel afspraken had u?	[ ] afspraken
11.10	Heeft u in de afgelopen 3 maanden hulp van de thuiszorg gehad?	◯ Ja<br>◯ Nee
11.10.1	***If 'Heeft u in de afgelopen 3 maanden hulp van de thuiszorg gehad?' is equal to 'Ja' answer this question:***<br>Wat voor hulp van de thuiszorg heeft u gehad in de afgelopen 3 maanden?	☐ Huishoudelijke hulp; voorbeeld: stofzuigen, bed opmaken, boodschappen doen<br>☐ Verzorging van uzelf; voorbeeld: hulp bij douchen of aankleden<br>☐ Verpleging; voorbeeld: verband omdoen, medicijnen geven, bloeddruk meten
11.10.1.1	***If 'Wat voor hulp van de thuiszorg heeft u gehad in de afgelopen 3 maanden?' is equal to 'Huishoudelijke hulp; voorbeeld: stofzuigen, bed opmaken, boodschappen doen' answer this question:***<br>Hoeveel weken heeft u deze thuiszorg gehad? Huishoudelijke hulp:	[ ] weken in de afgelopen 3 maanden
11.10.1.2	***If 'Wat voor hulp van de thuiszorg heeft u gehad in de afgelopen 3 maanden?' is equal to 'Verzorging van uzelf; voorbeeld: hulp bij douchen of aankleden' answer this question:***<br>Hoeveel weken heeft u deze thuiszorg gehad? Verzorging van uzelf:	[ ] weken in de afgelopen 3 maanden
11.10.1.3	***If 'Wat voor hulp van de thuiszorg heeft u gehad in de afgelopen 3 maanden?' is equal to 'Verpleging; voorbeeld: verband omdoen, medicijnen geven, bloeddruk meten' answer this question:***<br>Hoeveel weken heeft u deze thuiszorg gehad? Verpleging:	[ ] weken in de afgelopen 3 maanden

11.10.1.4	***If 'Wat voor hulp van de thuiszorg heeft u gehad in de afgelopen 3 maanden?' is equal to 'Huishoudelijke hulp; voorbeeld: stofzuigen, bed opmaken, boodschappen doen' answer this question:*** Hoeveel uur thuiszorg kreeg u in deze weken gemiddeld? Huishoudelijke hulp: gemiddeld	______ uur in de week
11.10.1.5	***If 'Wat voor hulp van de thuiszorg heeft u gehad in de afgelopen 3 maanden?' is equal to 'Verzorging van uzelf; voorbeeld: hulp bij douchen of aankleden' answer this question:*** Hoeveel uur thuiszorg kreeg u in deze weken gemiddeld? Verzorging van uzelf: gemiddeld	______ uur in de week
11.10.1.6	***If 'Wat voor hulp van de thuiszorg heeft u gehad in de afgelopen 3 maanden?' is equal to 'Verpleging; voorbeeld: verband omdoen, medicijnen geven, bloeddruk meten' answer this question:*** Hoeveel uur thuiszorg kreeg u in deze weken gemiddeld? Verpleging: gemiddeld	______ uur in de week
11.11	Heeft u in de afgelopen 3 maanden medicijnen gebruikt?	○ Ja<br>○ Nee
11.11.1	***If 'Heeft u in de afgelopen 3 maanden medicijnen gebruikt?' is equal to 'Ja' answer this question:*** Welke medicijnen heeft u in de afgelopen 3 maanden gebruikt?	
11.12	Bent u in de afgelopen 3 maanden op de spoedeisende eerste hulp van een ziekenhuis geweest? (Een andere naam voor spoedeisende eerste hulp is EHBO)	○ Nee<br>○ Ja
11.12.1	***If 'Bent u in de afgelopen 3 maanden op de spoedeisende eerste hulp van een ziekenhuis geweest? (Een andere naam voor spoedeisende eerste hulp is EHBO)' is equal to 'Ja' answer this question:*** Hoevaak bent u geweest?	______ keer
11.13	Bent u in de afgelopen 3 maanden met een ambulance naar het ziekenhuis gebracht? (Een andere naam voor ambulance is ziekenauto)	○ Nee<br>○ Ja
11.13.1	***If 'Bent u in de afgelopen 3 maanden met een ambulance naar het ziekenhuis gebracht? (Een andere naam voor ambulance is ziekenauto)' is equal to 'Ja' answer this question:*** Hoe vaak bent u naar het ziekenhuis gebracht?	______ keer

For peer review only - http://bmjopen.bmj.com/site/about/guidelines.xhtml

11.14	Had u in de afgelopen 3 maanden een afspraak bij de polikliniek van het ziekenhuis? (Het gaat om afspraken voor uzelf met een dokter. Bijvoorbeeld met de cardioloog, reumatoloog of neuroloog)	◯ Ja<br>◯ Nee
11.14.1	***If 'Had u in de afgelopen 3 maanden een afspraak bij de polikliniek van het ziekenhuis? (Het gaat om afspraken voor uzelf met een dokter. Bijvoorbeeld met de cardioloog, reumatoloog of neuroloog)' is equal to 'Ja' answer this question:***<br>Bij welke soorten dokters bent u in de afgelopen 3 maanden in het ziekenhuis geweest? En hoe vaak?	
11.15	Bent u in de afgelopen 3 maanden overdag in het ziekenhuis geweest voor een behandeling? (U bleef dus niet slapen. U kwam bijvoorbeeld voor een bloedtransfusie, nierdialyse of chemokuur)	◯ Ja<br>◯ Nee
11.15.1	***If 'Bent u in de afgelopen 3 maanden overdag in het ziekenhuis geweest voor een behandeling? (U bleef dus niet slapen. U kwam bijvoorbeeld voor een bloedtransfusie, nierdialyse of chemokuur)' is equal to 'Ja' answer this question:***<br>Voor welke soort behandeling was dit?	
11.15.2	***If 'Bent u in de afgelopen 3 maanden overdag in het ziekenhuis geweest voor een behandeling? (U bleef dus niet slapen. U kwam bijvoorbeeld voor een bloedtransfusie, nierdialyse of chemokuur)' is equal to 'Ja' answer this question:***<br>Hoeveel keer moest u in de afgelopen 3 maanden voor deze behandelingen naar het ziekenhuis?	
11.16	Bent u in de afgelopen 3 maanden ergens anders geweest voor een behandeling overdag? (U bleef dus niet slapen. U ging bijvoorbeeld naar de dagopvang van een woon-/zorgcentrum of een psychiatrische instelling. Of naar de dagbehandeling van een revalidatiecentrum)	◯ Ja<br>◯ Nee
11.16.1	***If 'Bent u in de afgelopen 3 maanden ergens anders geweest voor een behandeling overdag? (U bleef dus niet slapen. U ging bijvoorbeeld naar de dagopvang van een woon-/zorgcentrum of een psychiatrische instelling. Of naar de dagbehandeling van een revalidatiecentrum)' is equal to 'Ja' answer this question:***<br>Wat voor instelling was dit?	☐ Woon-/zorgcentrum<br>☐ Revalidatiecentrum<br>☐ Psychiatrische instelling<br>☐ Een andere instelling, namelijk
11.16.1.1	***If 'Wat voor instelling was dit?' is equal to 'Een andere instelling, namelijk' answer this question:***<br>Wat voor instelling was dit?	

11.16.1.2	***If 'Wat voor instelling was dit?' is equal to 'Woon-/zorgcentrum' answer this question:*** Hoe vaak moest u hier in de afgelopen 3 maanden naartoe? Naar het woon-/zorgcentrum:	______ keer in de afgelopen 3 maanden
11.16.1.3	***If 'Wat voor instelling was dit?' is equal to 'Revalidatiecentrum' answer this question:*** Hoe vaak moest u hier in de afgelopen 3 maanden naartoe? Naar het revalidatiecentrum:	______ keer in de afgelopen 3 maanden
11.16.1.4	***If 'Wat voor instelling was dit?' is equal to 'Psychiatrische instelling' answer this question:*** Hoe vaak moest u hier in de afgelopen 3 maanden naartoe? Naar de psychiatrische instelling:	______ keer in de afgelopen 3 maanden
11.16.1.5	***If 'Wat voor instelling was dit?' is equal to 'Een andere instelling, namelijk' answer this question:*** Hoe vaak moest u hier in de afgelopen 3 maanden naartoe? Naar de andere instelling:	______ keer in de afgelopen 3 maanden
11.17	Heeft u in de afgelopen 3 maanden weleens in het ziekenhuis gelegen? (U moest dus blijven slapen. Bijvoorbeeld omdat u geopereerd was en niet meteen naar huis kon)	○ Ja<br>○ Nee
11.17.1	***If 'Heeft u in de afgelopen 3 maanden weleens in het ziekenhuis gelegen? (U moest dus blijven slapen. Bijvoorbeeld omdat u geopereerd was en niet meteen naar huis kon)' is equal to 'Ja' answer this question:*** Hoe vaak heeft u in de afgelopen 3 maanden in het ziekenhuis gelegen?	______ keer in de afgelopen 3 maanden
11.17.2	***If 'Heeft u in de afgelopen 3 maanden weleens in het ziekenhuis gelegen? (U moest dus blijven slapen. Bijvoorbeeld omdat u geopereerd was en niet meteen naar huis kon)' is equal to 'Ja' answer this question:*** Hoe lang heeft u in het ziekenhuis gelegen?	______ dagen in de afgelopen 3 maanden
11.18	Moest u in de afgelopen 3 maanden ergens anders blijven slapen voor uw gezondheid? (Bijvoorbeeld in een woon-/zorgcentrum, psychiatrische instelling of revalidatiecentrum)	○ Ja<br>○ Nee
11.18.1	***If 'Moest u in de afgelopen 3 maanden ergens anders blijven slapen voor uw gezondheid? (Bijvoorbeeld in een woon-/zorgcentrum, psychiatrische instelling of revalidatiecentrum)' is equal to 'Ja' answer this question:*** Wat voor instelling was dit?	☐ Woon-/zorgcentrum<br>☐ Revalidatiecentrum<br>☐ Psychiatrische instelling<br>☐ Een andere instelling, namelijk

For peer review only - http://bmjopen.bmj.com/site/about/guidelines.xhtml

11.18.1.1	***If 'Wat voor instelling was dit?' is equal to 'Een andere instelling, namelijk' answer this question:*** Een andere instelling, namelijk	
11.18.1.2	***If 'Wat voor instelling was dit?' is equal to 'Woon-/zorgcentrum' answer this question:*** Hoe lang bent u in deze instelling geweest? In het woon-/zorgcentrum:	dagen in de afgelopen 3 maanden
11.18.1.3	***If 'Wat voor instelling was dit?' is equal to 'Revalidatiecentrum' answer this question:*** Hoe lang bent u in deze instelling geweest? In het revalidatiecentrum:	dagen in de afgelopen 3 maanden
11.18.1.4	***If 'Wat voor instelling was dit?' is equal to 'Psychiatrische instelling' answer this question:*** Hoe lang bent u in deze instelling geweest? In de psychiatrische instelling:	dagen in de afgelopen 3 maanden
11.18.1.5	***If 'Wat voor instelling was dit?' is equal to 'Een andere instelling, namelijk' answer this question:*** Hoe lang bent u in deze instelling geweest? In de andere instelling:	dagen in de afgelopen 3 maanden
11.19	Heeft u in de afgelopen 3 maanden hulp gekregen van een familielid of een bekende vanwege uw lichamelijke of psychische problemen?	◯ Ja<br>◯ Nee
11.19.1	***If 'Heeft u in de afgelopen 3 maanden hulp gekregen van een familielid of een bekende vanwege uw lichamelijke of psychische problemen?' is equal to 'Ja' answer this question:*** Wat voor hulp van familieleden of bekenden heeft u gehad in de afgelopen 3 maanden?	☐ Huishoudelijke hulp - voorbeeld: stofzuigen, bed opmaken, boodschappen doen, klaarmaken van eten en drinken, verzorgen van kinderen<br>☐ Verzorging van uzelf - voorbeeld: hulp bij douchen of aankleden, hulp bij het eten en drinken of het geven van medicijnen<br>☐ Praktische hulp - voorbeeld: ondersteuning bij wandelen, het maken van uitstapjes of bezoekjes aan bekenden, bezoeken aan de huisarts of het ziekenhuis, het regelen van hulp of het regelen van financiële zaken
11.19.1.1	***If 'Wat voor hulp van familieleden of bekenden heeft u gehad in de afgelopen 3 maanden?' is equal to 'Huishoudelijke hulp - voorbeeld: stofzuigen, bed opmaken, boodschappen doen, klaarmaken van eten en drinken, verzorgen van kinderen' answer this question:*** Hoeveel weken heeft u deze hulp gehad? Huishoudelijke hulp:	weken in de afgelopen 3 maanden

11.19.1.2	***If 'Wat voor hulp van familieleden of bekenden heeft u gehad in de afgelopen 3 maanden?' is equal to 'Verzorging van uzelf - voorbeeld: hulp bij douchen of aankleden, hulp bij het eten en drinken of het geven van medicijnen' answer this question:*** Hoeveel weken heeft u deze hulp gehad? Verzorging van uzelf:	______ weken in de afgelopen 3 maanden
11.19.1.3	***If 'Wat voor hulp van familieleden of bekenden heeft u gehad in de afgelopen 3 maanden?' is equal to 'Praktische hulp - voorbeeld: ondersteuning bij wandelen, het maken van uitstapjes of bezoekjes aan bekenden, bezoeken aan de huisarts of het ziekenhuis, het regelen van hulp of het regelen van financiële zaken' answer this question:*** Hoeveel uur hulp kreeg u in deze weken gemiddeld? Praktische hulp:	______ weken in de afgelopen 3 maanden
11.19.1.4	***If 'Wat voor hulp van familieleden of bekenden heeft u gehad in de afgelopen 3 maanden?' is equal to 'Huishoudelijke hulp - voorbeeld: stofzuigen, bed opmaken, boodschappen doen, klaarmaken van eten en drinken, verzorgen van kinderen' answer this question:*** Hoeveel uur hulp kreeg u in deze weken gemiddeld? Huishoudelijke hulp: gemiddeld	______ uur in de week
11.19.1.5	***If 'Wat voor hulp van familieleden of bekenden heeft u gehad in de afgelopen 3 maanden?' is equal to 'Verzorging van uzelf - voorbeeld: hulp bij douchen of aankleden, hulp bij het eten en drinken of het geven van medicijnen' answer this question:*** Hoeveel uur hulp kreeg u in deze weken gemiddeld? Verzorging van uzelf: gemiddeld	______ uur in de week
11.19.1.6	***If 'Wat voor hulp van familieleden of bekenden heeft u gehad in de afgelopen 3 maanden?' is equal to 'Praktische hulp - voorbeeld: ondersteuning bij wandelen, het maken van uitstapjes of bezoekjes aan bekenden, bezoeken aan de huisarts of het ziekenhuis, het regelen van hulp of het regelen van financiële zaken' answer this question:*** Hoeveel uur hulp kreeg u in deze weken gemiddeld? Praktische hulp: gemiddeld	______ uur in de week

Vragenlijst PRIMA studie - Dank

Number	Question	Answers

12.1	Hartelijk dank voor de tijd die u heeft genomen om de vragenlijsten in te vullen. Indien u nog opmerkingen heeft kunt u deze hierin plaatsen.	

Survey 'Vragenlijst PRIMA studie 12 weken'

Vragenlijst PRIMA studie 12 weken - AOFAS - Pijn

Number	Question	Answers
1.1	Hoeveel pijn ervaart u aan uw voet?	☐ Geen<br>☐ Milde pijnklachten of af en toe pijn<br>☐ Matige pijnklachten, dagelijks<br>☐ Vrijwel continu erge pijn

Vragenlijst PRIMA studie 12 weken - AOFAS resterende gedeelte

Number	Question	Answers
2.1	Hoeveel beperkingen in het dagelijks leven ervaart u?	○ Geen beperkingen, geen hulpmiddelen nodig<br>○ Geen beperkingen in algemeen dagelijkse bezigheden, wel beperkingen in recreatieve activiteiten (sport, hobbies, etc) geen ondersteuning<br>○ Beperkingen bij dagelijkse- en recreatieve activiteiten, stok<br>○ Ernstige beperkingen bij dagelijkse- en recreatieactiviteiten, walker, krukken, rolstoel, brace
2.2	Wat is de afstand die u in één keer kunt lopen?	○ Meer dan 600 meter<br>○ 400 - 600 meter<br>○ 100 - 300 meter<br>○ Minder dan 100 meter

2.3	Heeft u moeite met lopen op een bepaalde ondergrond?	◯ Geen problemen bij lopen<br>◯ Enig problemen op ongelijke ondergrond, trap, helling, ladders<br>◯ Ernstige problemen bij ongelijke ondergrond, trap, helling, ladders
2.4	Hoe zou u uw looppatroon beoordelen?	◯ Normaal of minimaal afwijkend<br>◯ Duidelijk<br>◯ Ernstig
2.5	Hoe zou u de beweeglijkheid van uw voet beoordelen ten opzicht van de andere zijde of ten opzicte van vóór het ongeval?	◯ Hetzelfde of licht beperkt t.o.v. de niet aangedane zijde of zoals voor het ongeval<br>◯ Ongeveer de helft beperkt<br>◯ Sterk verminderd, bijna geen bewegingen mogelijk
2.6	Hoe zou u de beweeglijkheid van uw enkel beoordelen ten opzichte van de andere zijde of ten opzichte van voor het ongeval?	◯ Hetzelfde of licht beperkt t.o.v. de niet aangedane zijde of zoals voor het ongeval<br>◯ Ongeveer de helft beperkt<br>◯ Sterk verminderd, bijna geen bewegingen mogelijk
2.7	Hoe zou u de stabiliteit van uw voet en enkel beoordelen	◯ Stabiel, ik verzwik mijn enkel hooguit incidenteel<br>◯ Niet stabiel, ik verzwik mijn enkel vaak
2.8	Hoe zou u de stand van uw voet beoordelen ten opzichte van de andere zijde of ten opzichte van voor het ongeval?	◯ Goed, ik kan mijn voet goed plat op de grond zetten, waarbij de stand niet afwijkend is<br>◯ Ik sta vrijwel alleen op de buitenzijde van mijn voet<br>◯ Ik kan mijn voet niet plat op de grond zetten en loop vrijwel alleen op mijn voorvoet

Vragenlijst PRIMA studie 12 weken - VAS Pain score

Number	Question	Answers	
3.1	VAS pijnscore (Geef uw minimaal pijnscore aan tijdens dagelijkse activiteiten op een schaal van 0 tot 100, waarbij 0 geen pijn is en 100 de ergste pijn denkbaar is)	(0.00)	100 (100.00)

Vragenlijst PRIMA studie 12 weken - AAS

Number	Question	Answers

4.1 Wat is het meest bij u van toepassing ten aanzien van uw acitviteit?

- ☐ American Football
- ☐ Basketbal
- ☐ Gymnastiek/turnen
- ☐ Handbal
- ☐ Rugby
- ☐ Voetbal
- ☐ Hockey
- ☐ Korfbal
- ☐ Gevechtssporten: judo, karate, kung fu, taekwando, jiu jitsu, etc.
- ☐ Oriëntatielopen
- ☐ Rhytmische gymnastiek
- ☐ Volleybal
- ☐ Boxen
- ☐ Freestyle snowboarden
- ☐ Ijshockey
- ☐ Tennis
- ☐ Worstelen
- ☐ Fitness, aerobics
- ☐ Badminton
- ☐ Baseball
- ☐ Cross-country hardlopen
- ☐ Moderne pentathlon
- ☐ Squash
- ☐ Surfen, windsurfen
- ☐ Tafel tennis
- ☐ Atletiek: spring-, werponderdelen
- ☐ Waterskieën
- ☐ Dans
- ☐ Schermen
- ☐ Zaalhockey
- ☐ Bergbeklimmen
- ☐ Langlauf
- ☐ Parachute springen
- ☐ Softball
- ☐ Speciale beroepen en werkactiviteiten; speciale beroepen: ballet, professioneel soldaat, speciale reddingswerker, stuntman, etc.
- ☐ Duiken
- ☐ Scubaduiken
- ☐ Skaten, in-line skaten
- ☐ Atletiek: looponderdelen
- ☐ Triatlon
- ☐ Gewichtsheffen, body-building

☐ Alle competitieve sporten hieronder met ‘seasonal’ conditioning
☐ Zwaar fysiek werk
☐ Alpine skiën en snowboarden
☐ Bowlen/curlen
☐ Golf
☐ Mountainbike/BMX
☐ Powerliften
☐ Zeilen
☐ Fysiek werk
☐ Wielrennen
☐ Paardrijden
☐ Motorsporten/technische sporten
☐ Roeien, kayakken
☐ Boogschieten
☐ Water polo en zwemmen
☐ Kunnen lopen op oneven grond
☐ Geen sport, echter geen limitatie in dagelijkse activiteiten
☐ Kunnen lopen op even grond, maar de dagelijkste activiteiten zijn gelimiteerd
☐ Niet kunnen lopen, fysieke handicap wegens enkelproblemen

4.1.1 ***If 'Wat is het meest bij u van toepassing ten aanzien van uw acitviteit?' is equal to 'American Football' answer this question:***
American Football

○ Professioneel
○ Competitief
○ Recreatief

4.1.2 ***If 'Wat is het meest bij u van toepassing ten aanzien van uw acitviteit?' is equal to 'Basketbal' answer this question:***
Basketbal

○ Professioneel
○ Competitief
○ Recreatief

4.1.3 ***If 'Wat is het meest bij u van toepassing ten aanzien van uw acitviteit?' is equal to 'Gymnastiek/turnen' answer this question:***
Gymnastiek/turnen

○ Professioneel
○ Competitief
○ Recreatief

4.1.4 ***If 'Wat is het meest bij u van toepassing ten aanzien van uw acitviteit?' is equal to 'Handbal' answer this question:***
Handbal

○ Professioneel
○ Competitief
○ Recreatief

4.1.5 ***If 'Wat is het meest bij u van toepassing ten aanzien van uw acitviteit?' is equal to 'Rugby' answer this question:***
Rugby

○ Professioneel
○ Competitief
○ Recreatief

4.1.6	***If 'Wat is het meest bij u van toepassing ten aanzien van uw acitviteit?' is equal to 'Voetbal' answer this question:***<br>Voetbal	◯ Professioneel<br>◯ Competitief<br>◯ Recreatief
4.1.7	***If 'Wat is het meest bij u van toepassing ten aanzien van uw acitviteit?' is equal to 'Hockey' answer this question:***<br>Hockey	◯ Professioneel<br>◯ Competitief<br>◯ Recreatief
4.1.8	***If 'Wat is het meest bij u van toepassing ten aanzien van uw acitviteit?' is equal to 'Korfbal' answer this question:***<br>Korfbal	◯ Professioneel<br>◯ Competitief<br>◯ Recreatief
4.1.9	***If 'Wat is het meest bij u van toepassing ten aanzien van uw acitviteit?' is equal to 'Gevechtssporten: judo, karate, kung fu, taekwando, jiu jitsu, etc.' answer this question:***<br>Gevechtssporten: judo, karate, kung fu, taekwando, jiu jitsu, etc.	◯ Professioneel<br>◯ Competitief<br>◯ Recreatief
4.1.10	***If 'Wat is het meest bij u van toepassing ten aanzien van uw acitviteit?' is equal to 'Oriëntatielopen' answer this question:***<br>Oriëntatielopen	◯ Professioneel<br>◯ Competitief<br>◯ Recreatief
4.1.11	***If 'Wat is het meest bij u van toepassing ten aanzien van uw acitviteit?' is equal to 'Rhytmische gymnastiek' answer this question:***<br>Rhytmisch gymnastiek	◯ Professioneel<br>◯ Competitief<br>◯ Recreatief
4.1.12	***If 'Wat is het meest bij u van toepassing ten aanzien van uw acitviteit?' is equal to 'Volleybal' answer this question:***<br>Volleybal	◯ Professioneel<br>◯ Competitief<br>◯ Recreatief
4.1.13	***If 'Wat is het meest bij u van toepassing ten aanzien van uw acitviteit?' is equal to 'Boxen' answer this question:***<br>Boxen	◯ Professioneel<br>◯ Competitief<br>◯ Recreatief
4.1.14	***If 'Wat is het meest bij u van toepassing ten aanzien van uw acitviteit?' is equal to 'Freestyle snowboarden' answer this question:***<br>Freestyle snowboarden	◯ Professioneel<br>◯ Competitief<br>◯ Recreatief
4.1.15	***If 'Wat is het meest bij u van toepassing ten aanzien van uw acitviteit?' is equal to 'Freestyle snowboarden' answer this question:***<br>IJshockey	◯ Professioneel<br>◯ Competitief<br>◯ Recreatief

4.1.16	***If 'Wat is het meest bij u van toepassing ten aanzien van uw acitviteit?' is equal to 'Tennis' answer this question:***<br>Tennis	◯ Professioneel<br>◯ Competitief<br>◯ Recreatief
4.1.17	***If 'Wat is het meest bij u van toepassing ten aanzien van uw acitviteit?' is equal to 'Worstelen' answer this question:***<br>Worstelen	◯ Professioneel<br>◯ Competitief<br>◯ Recreatief
4.1.18	***If 'Wat is het meest bij u van toepassing ten aanzien van uw acitviteit?' is equal to 'Fitness, aerobics' answer this question:***<br>Fitness, aerobics	◯ Professioneel<br>◯ Competitief<br>◯ Recreatief
4.1.19	***If 'Wat is het meest bij u van toepassing ten aanzien van uw acitviteit?' is equal to 'Badminton' answer this question:***<br>Badminton	◯ Professioneel<br>◯ Competitief<br>◯ Recreatief
4.1.20	***If 'Wat is het meest bij u van toepassing ten aanzien van uw acitviteit?' is equal to 'Baseball' answer this question:***<br>Baseball	◯ Professioneel<br>◯ Competitief<br>◯ Recreatief
4.1.21	***If 'Wat is het meest bij u van toepassing ten aanzien van uw acitviteit?' is equal to 'Cross-country hardlopen' answer this question:***<br>Cross-country hardlopen	◯ Professioneel<br>◯ Competitief<br>◯ Recreatief
4.1.22	***If 'Wat is het meest bij u van toepassing ten aanzien van uw acitviteit?' is equal to 'Moderne pentathlon' answer this question:***<br>Moderne pentathlon	◯ Professioneel<br>◯ Competitief<br>◯ Recreatief
4.1.23	***If 'Wat is het meest bij u van toepassing ten aanzien van uw acitviteit?' is equal to 'Squash' answer this question:***<br>Squash	◯ Professioneel<br>◯ Competitief<br>◯ Recreatief
4.1.24	***If 'Wat is het meest bij u van toepassing ten aanzien van uw acitviteit?' is equal to 'Surfen, windsurfen' answer this question:***<br>Surfen, windsurfen	◯ Professioneel<br>◯ Competitief<br>◯ Recreatief
4.1.25	***If 'Wat is het meest bij u van toepassing ten aanzien van uw acitviteit?' is equal to 'Tafel tennis' answer this question:***<br>Tafel tennis	◯ Professioneel<br>◯ Competitief<br>◯ Recreatief

4.1.26	***If 'Wat is het meest bij u van toepassing ten aanzien van uw acitviteit?' is equal to 'Atletiek: spring-, werponderdelen' answer this question:*** Atletiek: spring-, werponderdelen	◯ Professioneel ◯ Competitief ◯ Recreatief
4.1.27	***If 'Wat is het meest bij u van toepassing ten aanzien van uw acitviteit?' is equal to 'Waterskieën' answer this question:*** Waterskien	◯ Professioneel ◯ Competitief ◯ Recreatief
4.1.28	***If 'Wat is het meest bij u van toepassing ten aanzien van uw acitviteit?' is equal to 'Dans' answer this question:*** Dans	◯ Professioneel ◯ Competitief ◯ Recreatief
4.1.29	***If 'Wat is het meest bij u van toepassing ten aanzien van uw acitviteit?' is equal to 'Schermen' answer this question:*** Schermen	◯ Professioneel ◯ Competitief ◯ Recreatief
4.1.30	***If 'Wat is het meest bij u van toepassing ten aanzien van uw acitviteit?' is equal to 'Zaalhockey' answer this question:*** Zaalhockey	◯ Professioneel ◯ Competitief ◯ Recreatief
4.1.31	***If 'Wat is het meest bij u van toepassing ten aanzien van uw acitviteit?' is equal to 'Bergbeklimmen' answer this question:*** Bergbeklimmen	◯ Professioneel ◯ Competitief ◯ Recreatief
4.1.32	***If 'Wat is het meest bij u van toepassing ten aanzien van uw acitviteit?' is equal to 'Langlauf' answer this question:*** Langlauf	◯ Professioneel ◯ Competitief ◯ Recreatief
4.1.33	***If 'Wat is het meest bij u van toepassing ten aanzien van uw acitviteit?' is equal to 'Parachute springen' answer this question:*** Parachute springen	◯ Professioneel ◯ Competitief ◯ Recreatief
4.1.34	***If 'Wat is het meest bij u van toepassing ten aanzien van uw acitviteit?' is equal to 'Softball' answer this question:*** Softball	◯ Professioneel ◯ Competitief ◯ Recreatief

4.1.35	***If 'Wat is het meest bij u van toepassing ten aanzien van uw acitviteit?' is equal to 'Speciale beroepen en werkactiviteiten; speciale beroepen: ballet, professioneel soldaat, speciale reddingswerker, stuntman, etc.' answer this question:***<br>Speciale beroepen en werkactiviteiten speciale beroepen: ballet, professioneel soldaat, speciale reddingswerker, stuntman, etc.	◯ ballet, professioneel soldaat, speciale reddingswerker, stuntman, etc.
4.1.36	***If 'Wat is het meest bij u van toepassing ten aanzien van uw acitviteit?' is equal to 'Duiken' answer this question:***<br>Duiken	◯ Professioneel/competitief<br>◯ Recreatief
4.1.37	***If 'Wat is het meest bij u van toepassing ten aanzien van uw acitviteit?' is equal to 'Scubaduiken' answer this question:***<br>Scubaduiken	◯ Professioneel/competitief<br>◯ Recreatief
4.1.38	***If 'Wat is het meest bij u van toepassing ten aanzien van uw acitviteit?' is equal to 'Skaten, in-line skaten' answer this question:***<br>skaten, in-linen skaten	◯ Professioneel/competitief<br>◯ Recreatief
4.1.39	***If 'Wat is het meest bij u van toepassing ten aanzien van uw acitviteit?' is equal to 'Atletiek: looponderdelen' answer this question:***<br>Atletiek: looponderdelen	◯ Professioneel/competitief<br>◯ Recreatief
4.1.40	***If 'Wat is het meest bij u van toepassing ten aanzien van uw acitviteit?' is equal to 'Triatlon' answer this question:***<br>triatlon	◯ Professioneel/competitief<br>◯ Recreatief
4.1.41	***If 'Wat is het meest bij u van toepassing ten aanzien van uw acitviteit?' is equal to 'Gewichtsheffen, body-building' answer this question:***<br>Gewichtsheffen, body-building	◯ Professioneel/competitief<br>◯ Recreatief
4.1.42	***If 'Wat is het meest bij u van toepassing ten aanzien van uw acitviteit?' is equal to 'Alle competitieve sporten hieronder met ‘seasonal’ conditioning' answer this question:***<br>Alle competitieve sporten hieronder met ‘seasonal’ conditioning	◯ Alle competitieve sporten hieronder met ‘seasonal’ conditioning
4.1.43	***If 'Wat is het meest bij u van toepassing ten aanzien van uw acitviteit?' is equal to 'Zwaar fysiek werk' answer this question:***<br>Zwaar fysiek werk	◯ Alle competitieve sporten hieronder met ‘seasonal’ conditioning

4.1.44	***If 'Wat is het meest bij u van toepassing ten aanzien van uw acitviteit?' is equal to 'Alpine skiën en snowboarden' answer this question:***<br>Alpine skien en snowboarden	○ Alpine skien en snowboarden
4.1.45	***If 'Wat is het meest bij u van toepassing ten aanzien van uw acitviteit?' is equal to 'Bowlen/curlen' answer this question:***<br>Bowlen/curlen	○ Bowlen/curlen
4.1.46	***If 'Wat is het meest bij u van toepassing ten aanzien van uw acitviteit?' is equal to 'Golf' answer this question:***<br>Golf	○ Golf
4.1.47	***If 'Wat is het meest bij u van toepassing ten aanzien van uw acitviteit?' is equal to 'Mountainbike/BMX' answer this question:***<br>Mountainbike/BMX	○ Mountainbike/BMX
4.1.48	***If 'Wat is het meest bij u van toepassing ten aanzien van uw acitviteit?' is equal to 'Powerliften' answer this question:***<br>Powerliften	○ Powerliften
4.1.49	***If 'Wat is het meest bij u van toepassing ten aanzien van uw acitviteit?' is equal to 'Zeilen' answer this question:***<br>Zeilen	○ Zeilen
4.1.50	***If 'Wat is het meest bij u van toepassing ten aanzien van uw acitviteit?' is equal to 'Fysiek werk' answer this question:***<br>Fysiek werk	○ Fysiek werk
4.1.51	***If 'Wat is het meest bij u van toepassing ten aanzien van uw acitviteit?' is equal to 'Wielrennen' answer this question:***<br>Wielrennen	○ Wielrennen
4.1.52	***If 'Wat is het meest bij u van toepassing ten aanzien van uw acitviteit?' is equal to 'Paardrijden' answer this question:***<br>Paardrijden	○ Paardrijden
4.1.53	***If 'Wat is het meest bij u van toepassing ten aanzien van uw acitviteit?' is equal to 'Motorsporten/technische sporten' answer this question:***<br>Motorsporten/technische sporten	○ Motorsporten/technische sporten
4.1.54	***If 'Wat is het meest bij u van toepassing ten aanzien van uw acitviteit?' is equal to 'Roeien, kayakken' answer this question:***<br>Roeien, kayakken	○ Roeien, kayakken

4.1.55	***If 'Wat is het meest bij u van toepassing ten aanzien van uw acitviteit?' is equal to 'Boogschieten' answer this question:*** Boogschieten	◯ Boogschieten
4.1.56	***If 'Wat is het meest bij u van toepassing ten aanzien van uw acitviteit?' is equal to 'Water polo en zwemmen' answer this question:*** Water polo en zwemmen	◯ Water polo en zwemmen
4.1.57	***If 'Wat is het meest bij u van toepassing ten aanzien van uw acitviteit?' is equal to 'Kunnen lopen op oneven grond' answer this question:*** Kunnen lopen op oneven grond	◯ Kunnen lopen op oneven grond
4.1.58	***If 'Wat is het meest bij u van toepassing ten aanzien van uw acitviteit?' is equal to 'Geen sport, echter geen limitatie in dagelijkse activiteiten' answer this question:*** Geen sport, echter geen limitatie in dagelijkse activiteiten	◯ Geen sport, echter geen limitatie in dagelijkse activiteiten
4.1.59	***If 'Wat is het meest bij u van toepassing ten aanzien van uw acitviteit?' is equal to 'Kunnen lopen op even grond, maar de dagelijkste activiteiten zijn gelimiteerd' answer this question:*** Kunnen lopen op even grond, maar de dagelijkste activiteiten zijn gelimiteerd	◯ Kunnen lopen op even grond, maar de dagelijkste activiteiten zijn gelimiteerd
4.1.60	***If 'Wat is het meest bij u van toepassing ten aanzien van uw acitviteit?' is equal to 'Niet kunnen lopen, fysieke handicap wegens enkelproblemen' answer this question:*** Niet kunnen lopen, fysieke handicap wegens enkelproblemen	◯ Niet kunnen lopen, fysieke handicap wegens enkelproblemen

Vragenlijst PRIMA studie 12 weken - Hoe tevreden bent u over de enkelklachten?

Number	Question	Answers
5.1	Hoe tevreden bent u over de enkelklachten?	◯ Slecht<br>◯ Matig<br>◯ Goed<br>◯ Uitstekend

Vragenlijst PRIMA studie 12 weken - SF-36

Number	Question	Answers
	Deze vragenlijst gaat over uw standpunten t.a.v. uw gezondheid. Met behulp van deze gegevens kan worden bijgehouden hoe u zich voelt en hoe goed u in staat bent uw gebruikelijke bezigheden uit te voeren. Beantwoord elke vraag door een antwoord aan te klikken. Als u niet zeker weet hoe u een vraag moet beantwoorden, geef dan het best mogelijke antwoord.	
6.1	Hoe zou u over het algemeen uw gezondheid noemen?	◯ Uitstekend ◯ Zeer goed ◯ Goed ◯ Matig ◯ Slecht
6.2	Hoe beoordeelt u nu uw gezondheid over het algemeen vergeleken met een jaar geleden?	◯ Veel beter dan een jaar geleden ◯ Wat beter dan een jaar geleden ◯ Ongeveer hetzelfde als een jaar geleden ◯ Wat slechter dan een jaar geleden ◯ Veel slechter dan een jaar geleden
6.3	Wordt u door uw gezondheid op dit moment beperkt bij forse inspanning, zoals hardlopen, tillen van zware voorwerpen of een veeleisende sport beoefenen?	◯ Ja, ernstig beperkt ◯ Ja, een beetje beperkt ◯ Nee, helemaal niet beperkt
6.4	Wordt u door uw gezondheid op dit moment beperkt bij matige inspanning zoals een tafel verplaatsen, stofzuigen, zwemmen of fietsen?	◯ Ja, ernstig beperkt ◯ Ja, een beetje beperkt ◯ Nee, helemaal niet beperkt
6.5	Wordt u door uw gezondheid op dit moment beperkt bij boodschappen tillen of dragen?	◯ Ja, ernstig beperkt ◯ Ja, een beetje beperkt ◯ Nee, helemaal niet beperkt
6.6	Wordt u door uw gezondheid op dit moment beperkt bij een paar trappen oplopen?	◯ Ja, ernstig beperkt ◯ Ja, een beetje beperkt ◯ Nee, helemaal niet beperkt
6.7	Wordt u door uw gezondheid op dit moment beperkt bij één trap oplopen?	◯ Ja, ernstig beperkt ◯ Ja, een beetje beperkt ◯ Nee, helemaal niet beperkt
6.8	Wordt u door uw gezondheid op dit moment beperkt bij bukken, knielen of hurken?	◯ Ja, ernstig beperkt ◯ Ja, een beetje beperkt ◯ Nee, helemaal niet beperkt
6.9	Wordt u door uw gezondheid op dit moment beperkt bij meer dan één kilometer lopen?	◯ Ja, ernstig beperkt ◯ Ja, een beetje beperkt ◯ Nee, helemaal niet beperkt
6.10	Wordt u door uw gezondheid op dit moment beperkt bij een paar honderd meter lopen?	◯ Ja, ernstig beperkt ◯ Ja, een beetje beperkt ◯ Nee, helemaal niet beperkt

For peer review only - http://bmjopen.bmj.com/site/about/guidelines.xhtml

6.11	Wordt u door uw gezondheid op dit moment beperkt bij ongeveer honderd meter lopen?	◯ Ja, ernstig beperkt ◯ Ja, een beetje beperkt ◯ Nee, helemaal niet beperkt
6.12	Wordt u door uw gezondheid op dit moment beperkt bij uzelf wassen of aankleden?	◯ Ja, ernstig beperkt ◯ Ja, een beetje beperkt ◯ Nee, helemaal niet beperkt
6.13	U besteedde in de afgelopen 4 weken minder tijd aan werk of andere bezigheden	◯ Ja ◯ Nee
6.14	U heeft in de afgelopen 4 weken minder bereikt dan u zou willen	◯ Ja ◯ Nee
6.15	U was in de afgelopen 4 weken beperkt in het soort werk of andere bezigheden.	◯ Ja ◯ Nee
6.16	U had de afgelopen 4 weken moeite om uw werk of andere bezigheden uit te voeren (het kostte u bijvoorbeeld extra inspanning).	◯ Ja ◯ Nee
6.17	U besteedde in de afgelopen 4 weken minder tijd aan werk of andere bezigheden ten gevolge van emotionele problemen	◯ Ja ◯ Nee
6.18	U heeft in de afgelopen 4 weken minder bereikt dan u zou willen ten gevolge van emotionele problemen.	◯ Ja ◯ Nee
6.19	U deed de afgelopen 4 weken uw werk of andere bezigheden niet zo zorgvuldig als gewoonlijk ten gevolge van emotionele problemen.	◯ Ja ◯ Nee
6.20	In hoeverre hebben uw lichamelijke gezondheid of emotionele problemen u gedurende de afgelopen 4 weken gehinderd in uw normale omgang met familie, vrienden of buren, of bij activiteiten in groepsverband?	◯ Helemaal niet ◯ Enigszins ◯ Nogal ◯ Veel ◯ Heel erg veel
6.21	Hoeveel lichamelijke pijn heeft u de afgelopen 4 weken gehad?	◯ Geen<br>◯ Heel licht<br>◯ Licht<br>◯ Nogal<br>◯ Ernstig<br>◯ Heel ernstig
6.22	In welke mate bent u de afgelopen 4 weken door de pijn gehinderd in uw normale werk (zowel werk buitenshuis als huishoudelijk werk)?	◯ Helemaal niet<br>◯ Een klein beetje<br>◯ Nogal<br>◯ Veel<br>◯ Heel erg veel

6.23	Voelde u zich levenslustig?	◯ Altijd<br>◯ Meestal<br>◯ Vaak<br>◯ Soms<br>◯ Zelden<br>◯ Nooit
6.24	Was u erg zenuwachtig?	◯ Altijd<br>◯ Meestal<br>◯ Vaak<br>◯ Soms<br>◯ Zelden<br>◯ Nooit
6.25	Zat u zo in de put dat u niets kon?	◯ Altijd<br>◯ Meestal<br>◯ Vaak<br>◯ Soms<br>◯ Zelden<br>◯ Nooit
6.26	Voelde u zich rustig en tevreden?	◯ Altijd<br>◯ Meestal<br>◯ Vaak<br>◯ Soms<br>◯ Zelden<br>◯ Nooit
6.27	Had u veel energie?	◯ Altijd<br>◯ Meestal<br>◯ Vaak<br>◯ Soms<br>◯ Zelden<br>◯ Nooit
6.28	Voelde u zich somber en neerslachtig?	◯ Altijd<br>◯ Meestal<br>◯ Vaak<br>◯ Soms<br>◯ Zelden<br>◯ Nooit

6.29	Voelde u zich uitgeput?	◯ Altijd<br>◯ Meestal<br>◯ Vaak<br>◯ Soms<br>◯ Zelden<br>◯ Nooit
6.30	Was u een gelukkig mens?	◯ Altijd<br>◯ Meestal<br>◯ Vaak<br>◯ Soms<br>◯ Zelden<br>◯ Nooit
6.31	Voelde u zich moe?	◯ Altijd<br>◯ Meestal<br>◯ Vaak<br>◯ Soms<br>◯ Zelden<br>◯ Nooit
6.32	Hoe vaak hebben uw lichamelijke gezondheid of emotionele problemen u gedurende de afgelopen 4 weken gehinderd bij uw sociale activiteiten (zoals vrienden of familie bezoeken etc.)?	◯ Altijd<br>◯ Meestal<br>◯ Soms<br>◯ Zelden<br>◯ Nooit
6.33	Ik lijk wat gemakkelijker ziek te worden dan andere mensen	◯ Volkomen juist<br>◯ Grotendeels juist<br>◯ Weet ik niet<br>◯ Grotendeels onjuist<br>◯ Volkomen onjuist
6.34	Ik ben even gezond als andere mensen die ik ken	◯ Volkomen juist<br>◯ Grotendeels juist<br>◯ Weet ik niet<br>◯ Grotendeels onjuist<br>◯ Volkomen onjuist

6.35	Ik verwacht dat mijn gezondheid achteruit zal gaan	◯ Volkomen juist<br>◯ Grotendeels juist<br>◯ Weet ik niet<br>◯ Grotendeels onjuist<br>◯ Volkomen onjuist
6.36	Mijn gezondheid is uitstekend	◯ Volkomen juist<br>◯ Grotendeels juist<br>◯ Weet ik niet<br>◯ Grotendeels onjuist<br>◯ Volkomen onjuist

Vragenlijst PRIMA studie 12 weken - GAS

Number	Question	Answers
	Tijdens de eerste afspraak werd een duidelijk doel met u afgesproken. Dit werd tevens per email naar u gestuurd. Hoe is de situatie nu ten aanzien van de destijds gestelde doelstelling?	
7.1	Wat is nu de situatie ten aanzien van het tijdens de eerste afspraak afgesproken doelstelling (Goal attainment Scaling)?	◯ Achteruitgang (minder dan de utigangssituatie)<br>◯ Uitgangssituatie<br>◯ Minder dan het doel<br>◯ Doel<br>◯ Meer dan het doel<br>◯ Veel meer dan het doel

Vragenlijst PRIMA studie 12 weken - EQ-5D-3L

Number	Question	Answers
	Deze vragenlijst gaat over uw standpunten t.a.v. uw gezondheid. Met behulp van deze gegevens kan worden bijgehouden hoe u zich voelt en hoe goed u in staat bent uw gebruikelijke bezigheden uit te voeren. Beantwoord elke vraag door een antwoord aan te klikken. Als u niet zeker weet hoe u een vraag moet beantwoorden, geef dan het best mogelijke antwoord.	
8.1	Hoe is het met uw Mobiliteit gesteld?	◯ Ik heb geen problemen met lopen<br>◯ Ik heb enige problemen met lopen<br>◯ Ik ben bedlegerig

8.2	Hoe is het met uw Zelfzorg gesteld?	◯ Ik heb geen problemen om mijzelf te wassen of aan te kleden<br>◯ Ik heb enige problemen om mijzelf te wassen of aan te kleden<br>◯ Ik ben niet in staat om mijzelf te wassen of aan te kleden
8.3	Hoe is het met de Dagelijkse activiteiten (werk, studie, huishouden, gezins- en vrijetijdsactiviteiten) gesteld?	◯ Ik heb geen problemen met mijn dagelijkse activiteiten<br>◯ Ik heb enige problemen met mijn dagelijkse activiteiten<br>◯ Ik ben niet in staat om mijn dagelijkse activiteiten uit te voeren
8.4	Hoe is het met de Pijn/klachten gesteld?	◯ Ik heb geen pijn of andere klachten<br>◯ Ik heb matige pijn of andere klachten<br>◯ Ik heb zeer ernstige pijn of andere klachten
8.5	Hoe is het met de Stemming gesteld?	◯ Ik ben niet angstig of somber<br>◯ Ik ben matig angstig of somber<br>◯ Ik ben erg angstig of somber
	We willen weten hoe goed of slecht uw gezondheid VANDAAG is. Deze meetschaal loopt van 0 tot 100: 100 staat voor de BESTE gezondheid die u zich kunt voorstellen - 0 staat voor de SLECHTSTE gezondheid die u zich kunt voorstellen	
8.6	Uw Gezondheid vandaag	(0.00) (100.00)

Vragenlijst PRIMA studie 12 weken - AOS

Number	Question	Answers
	Instructies: De lijn naast elke vraag staat voor hoeveel PIJN u heeft in verschillende situaties. De linker kant (0) is “geen pijn” en de rechter kant (100) is “ergste pijn denkbaar”. Geef voor de onderstaande situaties op de lijn aan hoeveel PIJN u in de afgelopen week in de enkel had. Als een of meerdere van deze situaties niet van toepassing waren, dan kiest u de "niet van toepassing" optie.	
9.1	Wat was de hoogte van de ergste pijn in de afgelopen week?	(0.00) (100.00)
9.2	Heeft u pijn als u 's ochtends opstaat?	◯ Ja<br>◯ Nee<br>◯ Niet van toepassing

9.2.1	***If 'Heeft u pijn als u 's ochtends opstaat?' is equal to 'Ja' answer this question:***<br>Hoeveel pijn heeft u voordat u 's ochtends opstaat?	(0.00)	(100.00)
9.3	Heeft u pijn wanneer u op blote voeten loopt?	◯ Ja<br>◯ Nee<br>◯ Niet van toepassing	
9.3.1	***If 'Heeft u pijn wanneer u op blote voeten loopt?' is equal to 'Ja' answer this question:***<br>Hoeveel pijn heeft u als u op blote voeten loopt?	(0.00)	(100.00)
9.4	Heeft u pijn wanneer u op blote voeten staat?	◯ Ja<br>◯ Nee<br>◯ Niet van toepassing	
9.4.1	***If 'Heeft u pijn wanneer u op blote voeten staat?' is equal to 'Ja' answer this question:***<br>Hoeveel pijn heeft u als u op blote voeten staat?	(0.00)	(100.00)
9.5	Heeft u pijn wanneer u met schoenen loopt?	◯ Ja<br>◯ Nee<br>◯ Niet van toepassing	
9.5.1	***If 'Heeft u pijn wanneer u met schoenen loopt?' is equal to 'Ja' answer this question:***<br>Hoeveel pijn heeft u wanneer u met schoenen loopt?	(0.00)	(100.00)
9.6	Heeft u pijn wanneer u met schoenen staat?	◯ Ja<br>◯ Nee<br>◯ Niet van toepassing	
9.6.1	***If 'Heeft u pijn wanneer u met schoenen staat?' is equal to 'Ja' answer this question:***<br>Hoeveel pijn heeft u wanneer u met schoenen staat?	(0.00)	(100.00)
9.7	Heeft u pijn wanneer u loopt met steunzolen of een brace?	◯ Ja<br>◯ Nee<br>◯ Niet van toepassing	
9.7.1	***If 'Heeft u pijn wanneer u loopt met steunzolen of een brace?' is equal to 'Ja' answer this question:***<br>Hoeveel pijn heeft u wanneer u loopt met steunzolen of een brace?	(0.00)	(100.00)

9.8	Heeft u pijn wanneer u staat met steunzolen of een brace?	◯ Ja<br>◯ Nee<br>◯ Niet van toepassing	
9.8.1	***If 'Heeft u pijn wanneer u staat met steunzolen of een brace?' is equal to 'Ja' answer this question:***<br>Hoeveel pijn heeft u wanneer u staat met steunzolen of een brace?	(0.00)	(100.00)
9.9	Heeft u pijn aan het einde van de dag?	◯ Ja<br>◯ Nee<br>◯ Niet van toepassing	
9.9.1	***If 'Heeft u pijn aan het einde van de dag?' is equal to 'Ja' answer this question:***<br>Hoeveel pijn heeft u aan het einde van de dag?	(0.00)	(100.00)
	Instructies:De lijn naast elke vraag staat voor hoeveel MOEITE u heeft met verschillende activiteiten. De linker kant (0) is “Niet moeilijk” en de rechter kant (100) is “Te moeilijk, niet uitvoerbaar”. Geef voor de onderstaande activiteiten op de lijn aan hoeveel MOEITE u in de afgelopen week door enkelklachten had met onderstaande activiteiten. Als een situatie niet van toepassing is, kies dan de optie "niet van toepassing".		
9.10	Heeft u moeite met door het huis lopen?	◯ Ja<br>◯ Nee<br>◯ Niet van toepassing	
9.10.1	***If 'Heeft u moeite met door het huis lopen?' is equal to 'Ja' answer this question:***<br>Hoeveel moeite heeft u met door het huis lopen?	(0.00)	(100.00)
9.11	Heeft u moeite met buiten lopen op oneven ondergrond?	◯ Ja<br>◯ Nee<br>◯ Niet van toepassing	
9.11.1	***If 'Heeft u moeite met buiten lopen op oneven ondergrond?' is equal to 'Ja' answer this question:***<br>Hoeveel moeite heeft u met buiten lopen op oneven ondergrond?	(0.00)	(100.00)
9.12	Heeft u moeite met een paar honderd meter lopen?	◯ Ja<br>◯ Nee<br>◯ Niet van toepassing	
9.12.1	***If 'Heeft u moeite met een paar honderd meter lopen?' is equal to 'Ja' answer this question:***<br>Hoeveel moeite heeft u met een paar honderd meter lopen?	(0.00)	(100.00)

9.13	Heeft u moeite met een trap oplopen?	◯ Ja<br>◯ Nee<br>◯ Niet van toepassing	
9.13.1	***If 'Heeft u moeite met een trap oplopen?' is equal to 'Ja' answer this question:***<br>Hoeveel moeite heeft u met een trap oplopen?	(0.00)	(100.00)
9.14	Heeft u moeite met een trap aflopen?	◯ Ja<br>◯ Nee<br>◯ Niet van toepassing	
9.14.1	***If 'Heeft u moeite met een trap aflopen?' is equal to 'Ja' answer this question:***<br>Hoeveel moeite heeft u met een trap aflopen?	(0.00)	(100.00)
9.15	Heeft u moeite met op de tenen staan?	◯ Ja<br>◯ Nee<br>◯ Niet van toepassing	
9.15.1	***If 'Heeft u moeite met op de tenen staan?' is equal to 'Ja' answer this question:***<br>Hoeveel moeite heeft u met op de tenen staan?	(0.00)	(100.00)
9.16	Heeft u moeite met opstaan uit de stoel?	◯ Ja<br>◯ Nee<br>◯ Niet van toepassing	
9.16.1	***If 'Heeft u moeite met opstaan uit de stoel?' is equal to 'Ja' answer this question:***<br>Hoeveel moeite heeft u met opstaan uit de stoel?	(0.00)	(100.00)
9.17	Heeft u moeite met het op- of afstappen van stoepranden?	◯ Ja<br>◯ Nee<br>◯ Niet van toepassing	
9.17.1	***If 'Heeft u moeite met het op- of afstappen van stoepranden?' is equal to 'Ja' answer this question:***<br>Hoeveel moeite heeft u met het op- of afstappen van stoepranden?	(0.00)	(100.00)
9.18	Heeft u moeite met snel lopen of rennen?	◯ Ja<br>◯ Nee<br>◯ Niet van toepassing	

9.18.1	***If 'Heeft u moeite met snel lopen of rennen?' is equal to 'Ja' answer this question:*** Hoeveel moeite heeft u met snel lopen of rennen?	(0.00)	(100.00)

Vragenlijst PRIMA studie 12 weken - FAOS

Number	Question	Answers
	Deze lijst vraagt naar uw mening over uw voet/enkel. Uw antwoorden geven ons een beeld van uw voet/enkel klachten en hoe u in staat bent om alledaagse activiteiten uit te voeren in uw huidige situatie. Beantwoorden van een vraag doet u door het aanklikken van een vakje met het volgens u meest juiste antwoord (één vakje per vraag). Als u niet zeker weet hoe u een vraag moet beantwoorden, geeft u dan het antwoord dat volgens u het meest op uw situatie van toepassing is. Deze vraag heeft betrekking op het voorkomen van voet/enkel klachten in de afgelopen week.	
10.1	Is uw voet/enkel gezwollen?	○ Nooit ○ Zelden ○ Soms ○ Vaak ○ Altijd
10.2	Voelt u gekraak of hoort u klikken of een ander vreemd geluid wanneer u de voet/enkel beweegt?	○ Nooit ○ Zelden ○ Soms ○ Vaak ○ Altijd
10.3	Hapert uw enkel of blokkeert uw enkel ('op slot' gaan zitten) wanneer u deze beweegt?	○ Nooit ○ Zelden ○ Soms ○ Vaak ○ Altijd
10.4	Kunt u de voet/enkel volledig strekken?	○ Altijd ○ Vaak ○ Soms ○ Zelden ○ Nooit
10.5	Kunt u de voet volledig naar u toe buigen?	○ Altijd ○ Vaak ○ Soms ○ Zelden ○ Nooit
10.6	In welke mate heeft u een stijf gevoel in de voet/enkel 's ochtends bij het wakker worden?	○ Niet ○ Mild ○ Matig ○ Ernstig ○ Zeer ernstig
10.7	In welke mate heeft u een stijf gevoel in de voet/enkel na zitten, liggen of rusten later op de dag?	○ Niet ○ Mild ○ Matig ○ Ernstig ○ Zeer ernstig
10.8	Hoe vaak heeft u pijn in uw voet/enkel?	○ Nooit ○ Maandelijks ○ Wekelijks ○ Dagelijks ○ Altijd

10.9	Hoeveel voet/enkel pijn heeft u gehad in de afgelopen week bij draaien als uw voet/enkel op de grond staat?	◯ Geen<br>◯ Mild<br>◯ Matig<br>◯ Ernstig<br>◯ Zeer ernstig
10.10	Hoeveel voet/enkel pijn heeft u gehad in de afgelopen week bij het volledig uitstrekken van de voet/enkel?	◯ Geen<br>◯ Mild<br>◯ Matig<br>◯ Ernstig<br>◯ Zeer ernstig
10.11	Hoeveel voet/enkel pijn heeft u gehad in de afgelopen week bij het volledig naar u toe buigen/optrekken van de voet/enkel	◯ Geen<br>◯ Mild<br>◯ Matig<br>◯ Ernstig<br>◯ Zeer ernstig
10.12	Hoeveel voet/enkel pijn heeft u gehad in de afgelopen week bij het lopen op een vlakke ondergrond	◯ Geen<br>◯ Mild<br>◯ Matig<br>◯ Ernstig<br>◯ Zeer ernstig
10.13	Hoeveel voet/enkel pijn heeft u gehad in de afgelopen week bij het trap op- en trap af lopen?	◯ Geen<br>◯ Mild<br>◯ Matig<br>◯ Ernstig<br>◯ Zeer ernstig
10.14	Hoeveel voet/enkel pijn heeft u gehad in de afgelopen week 's nachts in bed?	◯ Geen<br>◯ Mild<br>◯ Matig<br>◯ Ernstig<br>◯ Zeer ernstig
10.15	Hoeveel voet/enkel pijn heeft u gehad in de afgelopen week bij het zitten of liggen?	◯ Geen<br>◯ Mild<br>◯ Matig<br>◯ Ernstig<br>◯ Zeer ernstig

10.16	Hoeveel voet/enkel pijn heeft u gehad in de afgelopen week bij het rechtop staan?	◯ Geen<br>◯ Mild<br>◯ Matig<br>◯ Ernstig<br>◯ Zeer ernstig
10.17	In welke mate werd u gehinderd bij het trap aflopen?	◯ Niet ◯ Mild ◯ Matig ◯ Ernstig ◯ Zeer ernstig
10.18	In welke mate werd u gehinderd bij het trap op lopen?	◯ Niet ◯ Mild ◯ Matig ◯ Ernstig ◯ Zeer ernstig
10.19	In welke mate werd u gehinderd als u vanuit een zittende positie ging staan?	◯ Niet ◯ Mild ◯ Matig ◯ Ernstig ◯ Zeer ernstig
10.20	In welke mate werd u gehinderd bij het staan?	◯ Niet ◯ Mild ◯ Matig ◯ Ernstig ◯ Zeer ernstig
10.21	In welke mate werd u gehinderd bij het naar de grond buigen/iets oprapen?	◯ Niet ◯ Mild ◯ Matig ◯ Ernstig ◯ Zeer ernstig
10.22	In welke mate werd u gehinderd bij het lopen op een vlakke ondergrond?	◯ Niet ◯ Mild ◯ Matig ◯ Ernstig ◯ Zeer ernstig
10.23	In welke mate werd u gehinderd bij het in- en uit de auto stappen?	◯ Niet ◯ Mild ◯ Matig ◯ Ernstig ◯ Zeer ernstig
10.24	In welke mate werd u gehinderd bij het boodschappen doen?	◯ Niet ◯ Mild ◯ Matig ◯ Ernstig ◯ Zeer ernstig
10.25	In welke mate werd u gehinderd bij sokken/panty's aantrekken?	◯ Niet ◯ Mild ◯ Matig ◯ Ernstig ◯ Zeer ernstig
10.26	In welke mate werd u gehinderd bij het opstaan uit bed?	◯ Niet ◯ Mild ◯ Matig ◯ Ernstig ◯ Zeer ernstig
10.27	In welke mate werd u gehinderd bij het sokken uittrekken?	◯ Niet ◯ Mild ◯ Matig ◯ Ernstig ◯ Zeer ernstig
10.28	In welke mate werd u gehinderd bij het in bed liggen (omdraaien, lange tijd uw voet/enkel in dezelfde positie houden)?	◯ Niet ◯ Mild ◯ Matig ◯ Ernstig ◯ Zeer ernstig

10.29	In welke mate werd u gehinderd bij het in/uit bad stappen?	○ Niet ○ Mild ○ Matig ○ Ernstig ○ Zeer ernstig
10.30	In welke mate werd u gehinderd bij zitten?	○ Niet ○ Mild ○ Matig ○ Ernstig ○ Zeer ernstig
10.31	In welke mate werd u gehinderd bij het toilet op en af gaan?	○ Niet ○ Mild ○ Matig ○ Ernstig ○ Zeer ernstig
10.32	In welke mate werd u gehinderd bij zwaar huishoudelijk werk (bijvoorbeeld zware dozen sjouwen, vloer schrobben)?	○ Niet ○ Mild ○ Matig ○ Ernstig ○ Zeer ernstig
10.33	In welke mate werd u gehinderd bij licht huishoudeijk werk (bijvoorbeeld koken, afstoffen)?	○ Niet ○ Mild ○ Matig ○ Ernstig ○ Zeer ernstig
10.34	In welke mate werd u gehinderd bij hurken?	○ Niet ○ Mild ○ Matig ○ Ernstig ○ Zeer ernstig
10.35	In welke mate werd u gehinderd bij hardlopen?	○ Niet ○ Mild ○ Matig ○ Ernstig ○ Zeer ernstig
10.36	In welke mate werd u gehinderd bij springen?	○ Niet ○ Mild ○ Matig ○ Ernstig ○ Zeer ernstig
10.37	In welke mate werd u gehinderd bij ronddraaien op uw aangedane voet/enkel?	○ Niet ○ Mild ○ Matig ○ Ernstig ○ Zeer ernstig
10.38	In welke mate werd u gehinderd bij knielen?	○ Niet ○ Mild ○ Matig ○ Ernstig ○ Zeer ernstig
10.39	Hoe vaak bent u zich bewust van uw voet/enkel probleem?	○ Nooit ○ Maandelijks ○ Wekelijks ○ Dagelijks ○ Altijd
10.40	Heeft u uw leven veranderd om activiteiten te vermijden die schadelijk kunnen zijn voor uw voet/enkel?	○ Niet<br>○ Enigszins<br>○ Matig<br>○ Behoorlijk<br>○ Volledig

10.41	In hoeverre kunt u op uw voet/enkel vertrouwen?	◯ Volledig<br>◯ Behoorlijk<br>◯ Matig<br>◯ Enigzins<br>◯ Niet
10.42	In het algemeen, in welke mate ondervindt u hinder van uw voet/enkel	◯ Geen<br>◯ Mild<br>◯ Matig<br>◯ Ernstig<br>◯ Zeer ernstig

Vragenlijst PRIMA studie 12 weken - PRODISQ - Gezondheid en werk

Number	Question	Answers
11.1	Wat is uw leeftijd?	[ ] jaar
11.2	Wat is uw geslacht?	◯ Man<br>◯ Vrouw
11.3	Wat is de hoogste opleiding die u heeft afgemaakt (Zoek uw hoogste opleiding en kruis het hokje daarvoor aan)?	☐ Ik heb geen school of opleiding afgemaakt<br>☐ Lagere school of basisschool<br>☐ Huishoudschool, vbo, lbo, lts, leao of lhno<br>☐ Mavo, mulo, ivo of vmbo<br>☐ Mbo, mts, meao, mhno, inas of intas<br>☐ Havo, vwo, hbs, mms, atheneum of gymnasium<br>☐ Hbo, hts, heao of hhno<br>☐ Universiteit<br>☐ Ik heb een andere opleiding afgemaakt, namelijk
11.3.1	***If 'Wat is de hoogste opleiding die u heeft afgemaakt (Zoek uw hoogste opleiding en kruis het hokje daarvoor aan)?' is equal to 'Ik heb een andere opleiding afgemaakt, namelijk' answer this question:***<br>Wat is de hoogste opleiding die u heeft afgemaakt?	[ ]

11.4	Wat doet u in het dagelijks leven?	☐ Ik zit op school, ik studeer<br>☐ Ik werk in loondienst<br>☐ Ik ben zelfstandig ondernemer<br>☐ Ik ben huisvrouw, huisman<br>☐ Ik ben werkloos<br>☐ Ik ben arbeidsongeschikt<br>☐ Ik ben met pensioen of prepensioen<br>☐ Ik doe iets anders, namelijk
11.4.1	***If 'Wat doet u in het dagelijks leven?' is equal to 'Ik ben arbeidsongeschikt' answer this question:***<br>Wat doet u in het dagelijks leven?	
11.4.2	***If 'Wat doet u in het dagelijks leven?' is equal to 'Ik doe iets anders, namelijk' answer this question:***<br>Wat doet u in het dagelijks leven?	
11.5	Hebt u betaald werk?	◯ Nee<br>◯ Ja
11.5.1	***If 'Hebt u betaald werk?' is equal to 'Ja' answer this question:***<br>Wat is uw beroep?	
11.5.2	***If 'Hebt u betaald werk?' is equal to 'Ja' answer this question:***<br>Hoeveel uur per week werkt u (Tel alleen de uren waarvoor u betaald wordt)?	uren
11.5.3	***If 'Hebt u betaald werk?' is equal to 'Ja' answer this question:***<br>Hoeveel dagen in de week werkt u?	Dagen
11.5.4	***If 'Hebt u betaald werk?' is equal to 'Ja' answer this question:***<br>Bent u in de afgelopen 4 weken afwezig geweest van uw werk omdat u ziek was?	◯ Nee<br>◯ Ja
11.5.4.1	***If 'Bent u in de afgelopen 4 weken afwezig geweest van uw werk omdat u ziek was?' is equal to 'Ja' answer this question:***<br>Bent u in de afgelopen 4 weken afwezig geweest van uw werk omdat u ziek was (Tel alleen de werkdagen in de afgelopen 4 weken)?	dagen afwezig geweest
11.5.4.2	***If 'Bent u in de afgelopen 4 weken afwezig geweest van uw werk omdat u ziek was?' is equal to 'Ja' answer this question:***<br>Was u langer dan de gehele periode van 4 weken afwezig van uw werk doordat u ziek was?	◯ Nee<br>◯ Ja

11.5.4.2.1	***If 'Was u langer dan de gehele periode van 4 weken afwezig van uw werk doordat u ziek was?' is equal to 'Ja' answer this question:*** Wanneer heeft u zich ziek gemeld?	☐ ☐ ☐ *(dd-mm-yyyy)*
11.5.5	***If 'Hebt u betaald werk?' is equal to 'Ja' answer this question:*** Waren er in de afgelopen 4 weken, dagen waarop u wel gewerkt heeft, maar tijdens uw werk last had van lichamelijke of psychische problemen?	◯ Ja<br>◯ Nee
11.5.5.1	***If 'Waren er in de afgelopen 4 weken, dagen waarop u wel gewerkt heeft, maar tijdens uw werk last had van lichamelijke of psychische problemen?' is equal to 'Ja' answer this question:*** Op hoeveel werkdagen had u tijdens uw werk last van uw lichamelijke of psychische problemen?	☐ werkdagen
11.5.5.2	***If 'Waren er in de afgelopen 4 weken, dagen waarop u wel gewerkt heeft, maar tijdens uw werk last had van lichamelijke of psychische problemen?' is equal to 'Ja' answer this question:*** Op de dagen dat u last had, kon u misschien niet zoveel werk doen als normaal. Hoeveel werk kon u op deze dagen gemiddeld doen?	Ik kon op deze dagen niks doen (0.00) — Ik kon net zoveel doen als normaal (10.00)
11.6	Waren er dagen waarop u minder onbetaald werk kon doen door uw lichamelijke of psychische problemen?	◯ Nee<br>◯ Ja
11.6.1	***If 'Waren er dagen waarop u minder onbetaald werk kon doen door uw lichamelijke of psychische problemen?' is equal to 'Ja' answer this question:*** Op hoeveel dagen was dit zo?	☐ Dagen
11.6.2	***If 'Waren er dagen waarop u minder onbetaald werk kon doen door uw lichamelijke of psychische problemen?' is equal to 'Ja' answer this question:*** Stel dat iemand, bijvoorbeeld uw partner, familielid of een bekende, u op deze dagen had geholpen. En al het onbetaalde werk wat u niet kon doen, voor u had gedaan. Hoeveel uur was die persoon hier op deze dagen dan gemiddeld mee bezig geweest?	☐ uur op deze dagen

Vragenlijst PRIMA studie 12 weken - PRODISQ - Zorggebruik

Number	Question	Answers
	Wij willen graag weten met welke dokters u in de afgelopen 3 maanden een afspraak had. Het gaat om afspraken voor uzelf. Ook andere zorgverleners tellen mee. Bijvoorbeeld de fysiotherapeut.	

12.1	Bent u in de afgelopen 3 maanden naar uw huisarts geweest?	◯ Nee<br>◯ Ja
12.1.1	***If 'Bent u in de afgelopen 3 maanden naar uw huisarts geweest?' is equal to 'Ja' answer this question:***<br>Hoeveel afspraken had u?	[ ] afspraken
12.2	Bent u in de afgelopen 3 maanden in contact geweest met een maatschappelijk werker?	◯ Nee<br>◯ Ja
12.2.1	***If 'Bent u in de afgelopen 3 maanden in contact geweest met een maatschappelijk werker?' is equal to 'Ja' answer this question:***<br>Hoeveel afspraken had u?	[ ] afspraken
12.3	Bent u in de afgelopen 3 maanden naar een fysiotherapeut geweest? Of een caesartherapeut, therapeut mensendieck of een manueel therapeut?	◯ Nee<br>◯ Ja
12.3.1	***If 'Bent u in de afgelopen 3 maanden naar een fysiotherapeut geweest? Of een caesartherapeut, therapeut mensendieck of een manueel therapeut?' is equal to 'Ja' answer this question:***<br>Hoeveel afspraken had u?	[ ] afspraken
12.4	Bent u in de afgelopen 3 maanden naar een ergotherapeut geweest?	◯ Nee<br>◯ Ja
12.4.1	***If 'Bent u in de afgelopen 3 maanden naar een ergotherapeut geweest?' is equal to 'Ja' answer this question:***<br>Hoeveel afspraken had u?	[ ] afspraken
12.5	Bent u in de afgelopen 3 maanden naar een logopedist geweest?	◯ Nee<br>◯ Ja
12.5.1	***If 'Bent u in de afgelopen 3 maanden naar een logopedist geweest?' is equal to 'Ja' answer this question:***<br>Hoeveel afspraken had u?	[ ] afspraken
12.6	Bent u in de afgelopen 3 maanden naar een diëtist geweest?	◯ Nee<br>◯ Ja
12.6.1	***If 'Bent u in de afgelopen 3 maanden naar een diëtist geweest?' is equal to 'Ja' answer this question:***<br>Hoeveel afspraken had u?	[ ] afspraken

12.7	Bent u in de afgelopen 3 maanden naar een homeopaat geweest? Of een acupuncturist?	◯ Nee<br>◯ Ja
12.7.1	***If 'Bent u in de afgelopen 3 maanden naar een homeopaat geweest? Of een acupuncturist?' is equal to 'Ja' answer this question:***<br>Hoeveel afspraken had u?	[ ] afspraken
12.8	Bent u in de afgelopen 3 maanden naar een psycholoog geweest? Of een psychotherapeut of psychiater?	◯ Nee<br>◯ Ja
12.8.1	***If 'Bent u in de afgelopen 3 maanden naar een psycholoog geweest? Of een psychotherapeut of psychiater?' is equal to 'Ja' answer this question:***<br>Hoeveel afspraken had u?	[ ] afspraken
12.9	Heeft u in de afgelopen 3 maanden afspraken gehad met de bedrijfsarts?	◯ Nee<br>◯ Ja
12.9.1	***If 'Heeft u in de afgelopen 3 maanden afspraken gehad met de bedrijfsarts?' is equal to 'Ja' answer this question:***<br>Hoeveel afspraken had u?	[ ] afspraken
12.10	Heeft u in de afgelopen 3 maanden hulp van de thuiszorg gehad?	◯ Ja<br>◯ Nee
12.10.1	***If 'Heeft u in de afgelopen 3 maanden hulp van de thuiszorg gehad?' is equal to 'Ja' answer this question:***<br>Wat voor hulp van de thuiszorg heeft u gehad in de afgelopen 3 maanden?	☐ Huishoudelijke hulp; voorbeeld: stofzuigen, bed opmaken, boodschappen doen<br>☐ Verzorging van uzelf; voorbeeld: hulp bij douchen of aankleden<br>☐ Verpleging; voorbeeld: verband omdoen, medicijnen geven, bloeddruk meten
12.10.1.1	***If 'Wat voor hulp van de thuiszorg heeft u gehad in de afgelopen 3 maanden?' is equal to 'Huishoudelijke hulp; voorbeeld: stofzuigen, bed opmaken, boodschappen doen' answer this question:***<br>Hoeveel weken heeft u deze thuiszorg gehad? Huishoudelijke hulp:	[ ] weken in de afgelopen 3 maanden
12.10.1.2	***If 'Wat voor hulp van de thuiszorg heeft u gehad in de afgelopen 3 maanden?' is equal to 'Verzorging van uzelf; voorbeeld: hulp bij douchen of aankleden' answer this question:***<br>Hoeveel weken heeft u deze thuiszorg gehad? Verzorging van uzelf:	[ ] weken in de afgelopen 3 maanden

12.10.1.3	***If 'Wat voor hulp van de thuiszorg heeft u gehad in de afgelopen 3 maanden?' is equal to 'Verpleging; voorbeeld: verband omdoen, medicijnen geven, bloeddruk meten' answer this question:***<br>Hoeveel weken heeft u deze thuiszorg gehad? Verpleging:	☐ weken in de afgelopen 3 maanden
12.10.1.4	***If 'Wat voor hulp van de thuiszorg heeft u gehad in de afgelopen 3 maanden?' is equal to 'Huishoudelijke hulp; voorbeeld: stofzuigen, bed opmaken, boodschappen doen' answer this question:***<br>Hoeveel uur thuiszorg kreeg u in deze weken gemiddeld? Huishoudelijke hulp: gemiddeld	☐ uur in de week
12.10.1.5	***If 'Wat voor hulp van de thuiszorg heeft u gehad in de afgelopen 3 maanden?' is equal to 'Verzorging van uzelf; voorbeeld: hulp bij douchen of aankleden' answer this question:***<br>Hoeveel uur thuiszorg kreeg u in deze weken gemiddeld? Verzorging van uzelf: gemiddeld	☐ uur in de week
12.10.1.6	***If 'Wat voor hulp van de thuiszorg heeft u gehad in de afgelopen 3 maanden?' is equal to 'Verpleging; voorbeeld: verband omdoen, medicijnen geven, bloeddruk meten' answer this question:***<br>Hoeveel uur thuiszorg kreeg u in deze weken gemiddeld? Verpleging: gemiddeld	☐ uur in de week
12.11	Heeft u in de afgelopen 3 maanden medicijnen gebruikt?	○ Ja<br>○ Nee
12.11.1	***If 'Heeft u in de afgelopen 3 maanden medicijnen gebruikt?' is equal to 'Ja' answer this question:***<br>Welke medicijnen heeft u in de afgelopen 3 maanden gebruikt?	☐
12.12	Bent u in de afgelopen 3 maanden op de spoedeisende eerste hulp van een ziekenhuis geweest? (Een andere naam voor spoedeisende eerste hulp is EHBO)	○ Nee<br>○ Ja
12.12.1	***If 'Bent u in de afgelopen 3 maanden op de spoedeisende eerste hulp van een ziekenhuis geweest? (Een andere naam voor spoedeisende eerste hulp is EHBO)' is equal to 'Ja' answer this question:***<br>Hoevaak bent u geweest?	☐ keer
12.13	Bent u in de afgelopen 3 maanden met een ambulance naar het ziekenhuis gebracht? (Een andere naam voor ambulance is ziekenauto)	○ Nee<br>○ Ja

12.13.1	***If 'Bent u in de afgelopen 3 maanden met een ambulance naar het ziekenhuis gebracht? (Een andere naam voor ambulance is ziekenauto)' is equal to 'Ja' answer this question:***<br>Hoe vaak bent u naar het ziekenhuis gebracht?	[ ] keer
12.14	Had u in de afgelopen 3 maanden een afspraak bij de polikliniek van het ziekenhuis? (Het gaat om afspraken voor uzelf met een dokter. Bijvoorbeeld met de cardioloog, reumatoloog of neuroloog)	◯ Ja<br>◯ Nee
12.14.1	***If 'Had u in de afgelopen 3 maanden een afspraak bij de polikliniek van het ziekenhuis? (Het gaat om afspraken voor uzelf met een dokter. Bijvoorbeeld met de cardioloog, reumatoloog of neuroloog)' is equal to 'Ja' answer this question:***<br>Bij welke soorten dokters bent u in de afgelopen 3 maanden in het ziekenhuis geweest? En hoe vaak?	[ ]
12.15	Bent u in de afgelopen 3 maanden overdag in het ziekenhuis geweest voor een behandeling? (U bleef dus niet slapen. U kwam bijvoorbeeld voor een bloedtransfusie, nierdialyse of chemokuur)	◯ Ja<br>◯ Nee
12.15.1	***If 'Bent u in de afgelopen 3 maanden overdag in het ziekenhuis geweest voor een behandeling? (U bleef dus niet slapen. U kwam bijvoorbeeld voor een bloedtransfusie, nierdialyse of chemokuur)' is equal to 'Ja' answer this question:***<br>Voor welke soort behandeling was dit?	[ ]
12.15.2	***If 'Bent u in de afgelopen 3 maanden overdag in het ziekenhuis geweest voor een behandeling? (U bleef dus niet slapen. U kwam bijvoorbeeld voor een bloedtransfusie, nierdialyse of chemokuur)' is equal to 'Ja' answer this question:***<br>Hoeveel keer moest u in de afgelopen 3 maanden voor deze behandelingen naar het ziekenhuis?	[ ]
12.16	Bent u in de afgelopen 3 maanden ergens anders geweest voor een behandeling overdag? (U bleef dus niet slapen. U ging bijvoorbeeld naar de dagopvang van een woon-/zorgcentrum of een psychiatrische instelling. Of naar de dagbehandeling van een revalidatiecentrum)	◯ Ja<br>◯ Nee
12.16.1	***If 'Bent u in de afgelopen 3 maanden ergens anders geweest voor een behandeling overdag? (U bleef dus niet slapen. U ging bijvoorbeeld naar de dagopvang van een woon-/zorgcentrum of een psychiatrische instelling. Of naar de dagbehandeling van een revalidatiecentrum)' is equal to 'Ja' answer this question:***<br>Wat voor instelling was dit?	☐ Woon-/zorgcentrum<br>☐ Revalidatiecentrum<br>☐ Psychiatrische instelling<br>☐ Een andere instelling, namelijk

12.16.1.1	***If 'Wat voor instelling was dit?' is equal to 'Een andere instelling, namelijk' answer this question:*** Wat voor instelling was dit?	
12.16.1.2	***If 'Wat voor instelling was dit?' is equal to 'Woon-/zorgcentrum' answer this question:*** Hoe vaak moest u hier in de afgelopen 3 maanden naartoe? Naar het woon-/zorgcentrum:	____ keer in de afgelopen 3 maanden
12.16.1.3	***If 'Wat voor instelling was dit?' is equal to 'Revalidatiecentrum' answer this question:*** Hoe vaak moest u hier in de afgelopen 3 maanden naartoe? Naar het revalidatiecentrum:	____ keer in de afgelopen 3 maanden
12.16.1.4	***If 'Wat voor instelling was dit?' is equal to 'Psychiatrische instelling' answer this question:*** Hoe vaak moest u hier in de afgelopen 3 maanden naartoe? Naar de psychiatrische instelling:	____ keer in de afgelopen 3 maanden
12.16.1.5	***If 'Wat voor instelling was dit?' is equal to 'Een andere instelling, namelijk' answer this question:*** Hoe vaak moest u hier in de afgelopen 3 maanden naartoe? Naar de andere instelling:	____ keer in de afgelopen 3 maanden
12.17	Heeft u in de afgelopen 3 maanden weleens in het ziekenhuis gelegen? (U moest dus blijven slapen. Bijvoorbeeld omdat u geopereerd was en niet meteen naar huis kon)	○ Ja<br>○ Nee
12.17.1	***If 'Heeft u in de afgelopen 3 maanden weleens in het ziekenhuis gelegen? (U moest dus blijven slapen. Bijvoorbeeld omdat u geopereerd was en niet meteen naar huis kon)' is equal to 'Ja' answer this question:*** Hoe vaak heeft u in de afgelopen 3 maanden in het ziekenhuis gelegen?	____ keer in de afgelopen 3 maanden
12.17.2	***If 'Heeft u in de afgelopen 3 maanden weleens in het ziekenhuis gelegen? (U moest dus blijven slapen. Bijvoorbeeld omdat u geopereerd was en niet meteen naar huis kon)' is equal to 'Ja' answer this question:*** Hoe lang heeft u in het ziekenhuis gelegen?	____ dagen in de afgelopen 3 maanden
12.18	Moest u in de afgelopen 3 maanden ergens anders blijven slapen voor uw gezondheid? (Bijvoorbeeld in een woon-/zorgcentrum, psychiatrische instelling of revalidatiecentrum)	○ Ja<br>○ Nee

12.18.1	***If 'Moest u in de afgelopen 3 maanden ergens anders blijven slapen voor uw gezondheid? (Bijvoorbeeld in een woon-/zorgcentrum, psychiatrische instelling of revalidatiecentrum)' is equal to 'Ja' answer this question:***<br>Wat voor instelling was dit?	☐ Woon-/zorgcentrum<br>☐ Revalidatiecentrum<br>☐ Psychiatrische instelling<br>☐ Een andere instelling, namelijk
12.18.1.1	***If 'Wat voor instelling was dit?' is equal to 'Een andere instelling, namelijk' answer this question:***<br>Een andere instelling, namelijk	
12.18.1.2	***If 'Wat voor instelling was dit?' is equal to 'Woon-/zorgcentrum' answer this question:***<br>Hoe lang bent u in deze instelling geweest? In het woon-/zorgcentrum:	______ dagen in de afgelopen 3 maanden
12.18.1.3	***If 'Wat voor instelling was dit?' is equal to 'Revalidatiecentrum' answer this question:***<br>Hoe lang bent u in deze instelling geweest? In het revalidatiecentrum:	______ dagen in de afgelopen 3 maanden
12.18.1.4	***If 'Wat voor instelling was dit?' is equal to 'Psychiatrische instelling' answer this question:***<br>Hoe lang bent u in deze instelling geweest? In de psychiatrische instelling:	______ dagen in de afgelopen 3 maanden
12.18.1.5	***If 'Wat voor instelling was dit?' is equal to 'Een andere instelling, namelijk' answer this question:***<br>Hoe lang bent u in deze instelling geweest? In de andere instelling:	______ dagen in de afgelopen 3 maanden
12.19	Heeft u in de afgelopen 3 maanden hulp gekregen van een familielid of een bekende vanwege uw lichamelijke of psychische problemen?	◯ Ja<br>◯ Nee
12.19.1	***If 'Heeft u in de afgelopen 3 maanden hulp gekregen van een familielid of een bekende vanwege uw lichamelijke of psychische problemen?' is equal to 'Ja' answer this question:***<br>Wat voor hulp van familieleden of bekenden heeft u gehad in de afgelopen 3 maanden?	☐ Huishoudelijke hulp - voorbeeld: stofzuigen, bed opmaken, boodschappen doen, klaarmaken van eten en drinken, verzorgen van kinderen<br>☐ Verzorging van uzelf - voorbeeld: hulp bij douchen of aankleden, hulp bij het eten en drinken of het geven van medicijnen<br>☐ Praktische hulp - voorbeeld: ondersteuning bij wandelen, het maken van uitstapjes of bezoekjes aan bekenden, bezoeken aan de huisarts of het ziekenhuis, het regelen van hulp of het regelen van financiële zaken

12.19.1.1	***If 'Wat voor hulp van familieleden of bekenden heeft u gehad in de afgelopen 3 maanden?' is equal to 'Huishoudelijke hulp - voorbeeld: stofzuigen, bed opmaken, boodschappen doen, klaarmaken van eten en drinken, verzorgen van kinderen' answer this question:***<br>Hoeveel weken heeft u deze hulp gehad? Huishoudelijke hulp:	[ ] weken in de afgelopen 3 maanden
12.19.1.2	***If 'Wat voor hulp van familieleden of bekenden heeft u gehad in de afgelopen 3 maanden?' is equal to 'Verzorging van uzelf - voorbeeld: hulp bij douchen of aankleden, hulp bij het eten en drinken of het geven van medicijnen' answer this question:***<br>Hoeveel weken heeft u deze hulp gehad? Verzorging van uzelf:	[ ] weken in de afgelopen 3 maanden
12.19.1.3	***If 'Wat voor hulp van familieleden of bekenden heeft u gehad in de afgelopen 3 maanden?' is equal to 'Praktische hulp - voorbeeld: ondersteuning bij wandelen, het maken van uitstapjes of bezoekjes aan bekenden, bezoeken aan de huisarts of het ziekenhuis, het regelen van hulp of het regelen van financiële zaken' answer this question:***<br>Hoeveel uur hulp kreeg u in deze weken gemiddeld? Praktische hulp:	[ ] weken in de afgelopen 3 maanden
12.19.1.4	***If 'Wat voor hulp van familieleden of bekenden heeft u gehad in de afgelopen 3 maanden?' is equal to 'Huishoudelijke hulp - voorbeeld: stofzuigen, bed opmaken, boodschappen doen, klaarmaken van eten en drinken, verzorgen van kinderen' answer this question:***<br>Hoeveel uur hulp kreeg u in deze weken gemiddeld? Huishoudelijke hulp: gemiddeld	[ ] uur in de week
12.19.1.5	***If 'Wat voor hulp van familieleden of bekenden heeft u gehad in de afgelopen 3 maanden?' is equal to 'Verzorging van uzelf - voorbeeld: hulp bij douchen of aankleden, hulp bij het eten en drinken of het geven van medicijnen' answer this question:***<br>Hoeveel uur hulp kreeg u in deze weken gemiddeld? Verzorging van uzelf: gemiddeld	[ ] uur in de week
12.19.1.6	***If 'Wat voor hulp van familieleden of bekenden heeft u gehad in de afgelopen 3 maanden?' is equal to 'Praktische hulp - voorbeeld: ondersteuning bij wandelen, het maken van uitstapjes of bezoekjes aan bekenden, bezoeken aan de huisarts of het ziekenhuis, het regelen van hulp of het regelen van financiële zaken' answer this question:***<br>Hoeveel uur hulp kreeg u in deze weken gemiddeld? Praktische hulp: gemiddeld	[ ] uur in de week

For peer review only - http://bmjopen.bmj.com/site/about/guidelines.xhtml

Vragenlijst PRIMA studie 12 weken - Dank

Number	Question	Answers
13.1	Hartelijk dank voor de tijd die u heeft genomen om de vragenlijsten in te vullen. Indien u nog opmerkingen heeft kunt u deze hierin plaatsen.	

Survey 'Vragenlijst PRIMA studie 26 weken'

Vragenlijst PRIMA studie 26 weken - AOFAS - Pijn

Number	Question	Answers
1.1	Hoeveel pijn ervaart u aan uw voet?	☐ Geen<br>☐ Milde pijnklachten of af en toe pijn<br>☐ Matige pijnklachten, dagelijks<br>☐ Vrijwel continu erge pijn

Vragenlijst PRIMA studie 26 weken - AOFAS resterende gedeelte

Number	Question	Answers
2.1	Hoeveel beperkingen in het dagelijks leven ervaart u?	○ Geen beperkingen, geen hulpmiddelen nodig<br>○ Geen beperkingen in algemeen dagelijkse bezigheden, wel beperkingen in recreatieve activiteiten (sport, hobbies, etc) geen ondersteuning<br>○ Beperkingen bij dagelijkse- en recreatieve activiteiten, stok<br>○ Ernstige beperkingen bij dagelijkse- en recreatieactiviteiten, walker, krukken, rolstoel, brace

2.2	Wat is de afstand die u in één keer kunt lopen?	◯ Meer dan 600 meter<br>◯ 400 - 600 meter<br>◯ 100 - 300 meter<br>◯ Minder dan 100 meter
2.3	Heeft u moeite met lopen op een bepaalde ondergrond?	◯ Geen problemen bij lopen<br>◯ Enig problemen op ongelijke ondergrond, trap, helling, ladders<br>◯ Ernstige problemen bij ongelijke ondergrond, trap, helling, ladders
2.4	Hoe zou u uw looppatroon beoordelen?	◯ Normaal of minimaal afwijkend<br>◯ Duidelijk<br>◯ Ernstig
2.5	Hoe zou u de beweeglijkheid van uw voet beoordelen ten opzicht van de andere zijde of ten opzicte van vóór het ongeval?	◯ Hetzelfde of licht beperkt t.o.v. de niet aangedane zijde of zoals voor het ongeval<br>◯ Ongeveer de helft beperkt<br>◯ Sterk verminderd, bijna geen bewegingen mogelijk
2.6	Hoe zou u de beweeglijkheid van uw enkel beoordelen ten opzichte van de andere zijde of ten opzichte van voor het ongeval?	◯ Hetzelfde of licht beperkt t.o.v. de niet aangedane zijde of zoals voor het ongeval<br>◯ Ongeveer de helft beperkt<br>◯ Sterk verminderd, bijna geen bewegingen mogelijk
2.7	Hoe zou u de stabiliteit van uw voet en enkel beoordelen	◯ Stabiel, ik verzwik mijn enkel hooguit incidenteel<br>◯ Niet stabiel, ik verzwik mijn enkel vaak
2.8	Hoe zou u de stand van uw voet beoordelen ten opzichte van de andere zijde of ten opzichte van voor het ongeval?	◯ Goed, ik kan mijn voet goed plat op de grond zetten, waarbij de stand niet afwijkend is<br>◯ Ik sta vrijwel alleen op de buitenzijde van mijn voet<br>◯ Ik kan mijn voet niet plat op de grond zetten en loop vrijwel alleen op mijn voorvoet

Vragenlijst PRIMA studie 26 weken - VAS Pain score

Number	Question	Answers	
3.1	VAS pijnscore (Geef uw minimaal pijnscore aan tijdens dagelijkse activiteiten op een schaal van 0 tot 100, waarbij 0 geen pijn is en 100 de ergste pijn denkbaar is)	(0.00)	100 (100.00)

Vragenlijst PRIMA studie 26 weken - AAS

Number	Question	Answers

4.1	Wat is het meest bij u van toepassing ten aanzien van uw acitviteit?	☐ American Football<br>☐ Basketbal<br>☐ Gymnastiek/turnen<br>☐ Handbal<br>☐ Rugby<br>☐ Voetbal<br>☐ Hockey<br>☐ Korfbal<br>☐ Gevechtssporten: judo, karate, kung fu, taekwando, jiu jitsu, etc.<br>☐ Oriëntatielopen<br>☐ Rhytmische gymnastiek<br>☐ Volleybal<br>☐ Boxen<br>☐ Freestyle snowboarden<br>☐ Ijshockey<br>☐ Tennis<br>☐ Worstelen<br>☐ Fitness, aerobics<br>☐ Badminton<br>☐ Baseball<br>☐ Cross-country hardlopen<br>☐ Moderne pentathlon<br>☐ Squash<br>☐ Surfen, windsurfen<br>☐ Tafel tennis<br>☐ Atletiek: spring-, werponderdelen<br>☐ Waterskieën<br>☐ Dans<br>☐ Schermen<br>☐ Zaalhockey<br>☐ Bergbeklimmen<br>☐ Langlauf<br>☐ Parachute springen<br>☐ Softball<br>☐ Speciale beroepen en werkactiviteiten; speciale beroepen: ballet, professioneel soldaat, speciale reddingswerker, stuntman, etc.<br>☐ Duiken<br>☐ Scubaduiken<br>☐ Skaten, in-line skaten<br>☐ Atletiek: looponderdelen<br>☐ Triatlon<br>☐ Gewichtsheffen, body-building

☐ Alle competitieve sporten hieronder met 'seasonal' conditioning
☐ Zwaar fysiek werk
☐ Alpine skiën en snowboarden
☐ Bowlen/curlen
☐ Golf
☐ Mountainbike/BMX
☐ Powerliften
☐ Zeilen
☐ Fysiek werk
☐ Wielrennen
☐ Paardrijden
☐ Motorsporten/technische sporten
☐ Roeien, kayakken
☐ Boogschieten
☐ Water polo en zwemmen
☐ Kunnen lopen op oneven grond
☐ Geen sport, echter geen limitatie in dagelijkse activiteiten
☐ Kunnen lopen op even grond, maar de dagelijkste activiteiten zijn gelimiteerd
☐ Niet kunnen lopen, fysieke handicap wegens enkelproblemen

4.1.1	***If 'Wat is het meest bij u van toepassing ten aanzien van uw acitviteit?' is equal to 'American Football' answer this question:***<br>American Football	◯ Professioneel<br>◯ Competitief<br>◯ Recreatief
4.1.2	***If 'Wat is het meest bij u van toepassing ten aanzien van uw acitviteit?' is equal to 'Basketbal' answer this question:***<br>Basketbal	◯ Professioneel<br>◯ Competitief<br>◯ Recreatief
4.1.3	***If 'Wat is het meest bij u van toepassing ten aanzien van uw acitviteit?' is equal to 'Gymnastiek/turnen' answer this question:***<br>Gymnastiek/turnen	◯ Professioneel<br>◯ Competitief<br>◯ Recreatief
4.1.4	***If 'Wat is het meest bij u van toepassing ten aanzien van uw acitviteit?' is equal to 'Handbal' answer this question:***<br>Handbal	◯ Professioneel<br>◯ Competitief<br>◯ Recreatief
4.1.5	***If 'Wat is het meest bij u van toepassing ten aanzien van uw acitviteit?' is equal to 'Rugby' answer this question:***<br>Rugby	◯ Professioneel<br>◯ Competitief<br>◯ Recreatief

4.1.6	***If 'Wat is het meest bij u van toepassing ten aanzien van uw acitviteit?' is equal to 'Voetbal' answer this question:***<br>Voetbal	◯ Professioneel<br>◯ Competitief<br>◯ Recreatief
4.1.7	***If 'Wat is het meest bij u van toepassing ten aanzien van uw acitviteit?' is equal to 'Hockey' answer this question:***<br>Hockey	◯ Professioneel<br>◯ Competitief<br>◯ Recreatief
4.1.8	***If 'Wat is het meest bij u van toepassing ten aanzien van uw acitviteit?' is equal to 'Korfbal' answer this question:***<br>Korfbal	◯ Professioneel<br>◯ Competitief<br>◯ Recreatief
4.1.9	***If 'Wat is het meest bij u van toepassing ten aanzien van uw acitviteit?' is equal to 'Gevechtssporten: judo, karate, kung fu, taekwando, jiu jitsu, etc.' answer this question:***<br>Gevechtssporten: judo, karate, kung fu, taekwando, jiu jitsu, etc.	◯ Professioneel<br>◯ Competitief<br>◯ Recreatief
4.1.10	***If 'Wat is het meest bij u van toepassing ten aanzien van uw acitviteit?' is equal to 'Oriëntatielopen' answer this question:***<br>Oriëntatielopen	◯ Professioneel<br>◯ Competitief<br>◯ Recreatief
4.1.11	***If 'Wat is het meest bij u van toepassing ten aanzien van uw acitviteit?' is equal to 'Rhytmische gymnastiek' answer this question:***<br>Rhytmisch gymnastiek	◯ Professioneel<br>◯ Competitief<br>◯ Recreatief
4.1.12	***If 'Wat is het meest bij u van toepassing ten aanzien van uw acitviteit?' is equal to 'Volleybal' answer this question:***<br>Volleybal	◯ Professioneel<br>◯ Competitief<br>◯ Recreatief
4.1.13	***If 'Wat is het meest bij u van toepassing ten aanzien van uw acitviteit?' is equal to 'Boxen' answer this question:***<br>Boxen	◯ Professioneel<br>◯ Competitief<br>◯ Recreatief
4.1.14	***If 'Wat is het meest bij u van toepassing ten aanzien van uw acitviteit?' is equal to 'Freestyle snowboarden' answer this question:***<br>Freestyle snowboarden	◯ Professioneel<br>◯ Competitief<br>◯ Recreatief
4.1.15	***If 'Wat is het meest bij u van toepassing ten aanzien van uw acitviteit?' is equal to 'Freestyle snowboarden' answer this question:***<br>IJshockey	◯ Professioneel<br>◯ Competitief<br>◯ Recreatief

4.1.16	***If 'Wat is het meest bij u van toepassing ten aanzien van uw acitviteit?' is equal to 'Tennis' answer this question:***<br>Tennis	◯ Professioneel<br>◯ Competitief<br>◯ Recreatief
4.1.17	***If 'Wat is het meest bij u van toepassing ten aanzien van uw acitviteit?' is equal to 'Worstelen' answer this question:***<br>Worstelen	◯ Professioneel<br>◯ Competitief<br>◯ Recreatief
4.1.18	***If 'Wat is het meest bij u van toepassing ten aanzien van uw acitviteit?' is equal to 'Fitness, aerobics' answer this question:***<br>Fitness, aerobics	◯ Professioneel<br>◯ Competitief<br>◯ Recreatief
4.1.19	***If 'Wat is het meest bij u van toepassing ten aanzien van uw acitviteit?' is equal to 'Badminton' answer this question:***<br>Badminton	◯ Professioneel<br>◯ Competitief<br>◯ Recreatief
4.1.20	***If 'Wat is het meest bij u van toepassing ten aanzien van uw acitviteit?' is equal to 'Baseball' answer this question:***<br>Baseball	◯ Professioneel<br>◯ Competitief<br>◯ Recreatief
4.1.21	***If 'Wat is het meest bij u van toepassing ten aanzien van uw acitviteit?' is equal to 'Cross-country hardlopen' answer this question:***<br>Cross-country hardlopen	◯ Professioneel<br>◯ Competitief<br>◯ Recreatief
4.1.22	***If 'Wat is het meest bij u van toepassing ten aanzien van uw acitviteit?' is equal to 'Moderne pentathlon' answer this question:***<br>Moderne pentathlon	◯ Professioneel<br>◯ Competitief<br>◯ Recreatief
4.1.23	***If 'Wat is het meest bij u van toepassing ten aanzien van uw acitviteit?' is equal to 'Squash' answer this question:***<br>Squash	◯ Professioneel<br>◯ Competitief<br>◯ Recreatief
4.1.24	***If 'Wat is het meest bij u van toepassing ten aanzien van uw acitviteit?' is equal to 'Surfen, windsurfen' answer this question:***<br>Surfen, windsurfen	◯ Professioneel<br>◯ Competitief<br>◯ Recreatief
4.1.25	***If 'Wat is het meest bij u van toepassing ten aanzien van uw acitviteit?' is equal to 'Tafel tennis' answer this question:***<br>Tafel tennis	◯ Professioneel<br>◯ Competitief<br>◯ Recreatief

4.1.26	***If 'Wat is het meest bij u van toepassing ten aanzien van uw acitviteit?' is equal to 'Atletiek: spring-, werponderdelen' answer this question:***<br>Atletiek: spring-, werponderdelen	◯ Professioneel<br>◯ Competitief<br>◯ Recreatief
4.1.27	***If 'Wat is het meest bij u van toepassing ten aanzien van uw acitviteit?' is equal to 'Waterskieën' answer this question:***<br>Waterskien	◯ Professioneel<br>◯ Competitief<br>◯ Recreatief
4.1.28	***If 'Wat is het meest bij u van toepassing ten aanzien van uw acitviteit?' is equal to 'Dans' answer this question:***<br>Dans	◯ Professioneel<br>◯ Competitief<br>◯ Recreatief
4.1.29	***If 'Wat is het meest bij u van toepassing ten aanzien van uw acitviteit?' is equal to 'Schermen' answer this question:***<br>Schermen	◯ Professioneel<br>◯ Competitief<br>◯ Recreatief
4.1.30	***If 'Wat is het meest bij u van toepassing ten aanzien van uw acitviteit?' is equal to 'Zaalhockey' answer this question:***<br>Zaalhockey	◯ Professioneel<br>◯ Competitief<br>◯ Recreatief
4.1.31	***If 'Wat is het meest bij u van toepassing ten aanzien van uw acitviteit?' is equal to 'Bergbeklimmen' answer this question:***<br>Bergbeklimmen	◯ Professioneel<br>◯ Competitief<br>◯ Recreatief
4.1.32	***If 'Wat is het meest bij u van toepassing ten aanzien van uw acitviteit?' is equal to 'Langlauf' answer this question:***<br>Langlauf	◯ Professioneel<br>◯ Competitief<br>◯ Recreatief
4.1.33	***If 'Wat is het meest bij u van toepassing ten aanzien van uw acitviteit?' is equal to 'Parachute springen' answer this question:***<br>Parachute springen	◯ Professioneel<br>◯ Competitief<br>◯ Recreatief
4.1.34	***If 'Wat is het meest bij u van toepassing ten aanzien van uw acitviteit?' is equal to 'Softball' answer this question:***<br>Softball	◯ Professioneel<br>◯ Competitief<br>◯ Recreatief

4.1.35	***If 'Wat is het meest bij u van toepassing ten aanzien van uw acitviteit?' is equal to 'Speciale beroepen en werkactiviteiten; speciale beroepen: ballet, professioneel soldaat, speciale reddingswerker, stuntman, etc.' answer this question:***<br>Speciale beroepen en werkactiviteiten speciale beroepen: ballet, professioneel soldaat, speciale reddingswerker, stuntman, etc.	○ ballet, professioneel soldaat, speciale reddingswerker, stuntman, etc.
4.1.36	***If 'Wat is het meest bij u van toepassing ten aanzien van uw acitviteit?' is equal to 'Duiken' answer this question:***<br>Duiken	○ Professioneel/competitief<br>○ Recreatief
4.1.37	***If 'Wat is het meest bij u van toepassing ten aanzien van uw acitviteit?' is equal to 'Scubaduiken' answer this question:***<br>Scubaduiken	○ Professioneel/competitief<br>○ Recreatief
4.1.38	***If 'Wat is het meest bij u van toepassing ten aanzien van uw acitviteit?' is equal to 'Skaten, in-line skaten' answer this question:***<br>skaten, in-linen skaten	○ Professioneel/competitief<br>○ Recreatief
4.1.39	***If 'Wat is het meest bij u van toepassing ten aanzien van uw acitviteit?' is equal to 'Atletiek: looponderdelen' answer this question:***<br>Atletiek: looponderdelen	○ Professioneel/competitief<br>○ Recreatief
4.1.40	***If 'Wat is het meest bij u van toepassing ten aanzien van uw acitviteit?' is equal to 'Triatlon' answer this question:***<br>triatlon	○ Professioneel/competitief<br>○ Recreatief
4.1.41	***If 'Wat is het meest bij u van toepassing ten aanzien van uw acitviteit?' is equal to 'Gewichtsheffen, body-building' answer this question:***<br>Gewichtsheffen, body-building	○ Professioneel/competitief<br>○ Recreatief
4.1.42	***If 'Wat is het meest bij u van toepassing ten aanzien van uw acitviteit?' is equal to 'Alle competitieve sporten hieronder met ‘seasonal’ conditioning' answer this question:***<br>Alle competitieve sporten hieronder met ‘seasonal’ conditioning	○ Alle competitieve sporten hieronder met ‘seasonal’ conditioning
4.1.43	***If 'Wat is het meest bij u van toepassing ten aanzien van uw acitviteit?' is equal to 'Zwaar fysiek werk' answer this question:***<br>Zwaar fysiek werk	○ Alle competitieve sporten hieronder met ‘seasonal’ conditioning

4.1.44	***If 'Wat is het meest bij u van toepassing ten aanzien van uw acitviteit?' is equal to 'Alpine skiën en snowboarden' answer this question:***<br>Alpine skien en snowboarden	◯ Alpine skien en snowboarden
4.1.45	***If 'Wat is het meest bij u van toepassing ten aanzien van uw acitviteit?' is equal to 'Bowlen/curlen' answer this question:***<br>Bowlen/curlen	◯ Bowlen/curlen
4.1.46	***If 'Wat is het meest bij u van toepassing ten aanzien van uw acitviteit?' is equal to 'Golf' answer this question:***<br>Golf	◯ Golf
4.1.47	***If 'Wat is het meest bij u van toepassing ten aanzien van uw acitviteit?' is equal to 'Mountainbike/BMX' answer this question:***<br>Mountainbike/BMX	◯ Mountainbike/BMX
4.1.48	***If 'Wat is het meest bij u van toepassing ten aanzien van uw acitviteit?' is equal to 'Powerliften' answer this question:***<br>Powerliften	◯ Powerliften
4.1.49	***If 'Wat is het meest bij u van toepassing ten aanzien van uw acitviteit?' is equal to 'Zeilen' answer this question:***<br>Zeilen	◯ Zeilen
4.1.50	***If 'Wat is het meest bij u van toepassing ten aanzien van uw acitviteit?' is equal to 'Fysiek werk' answer this question:***<br>Fysiek werk	◯ Fysiek werk
4.1.51	***If 'Wat is het meest bij u van toepassing ten aanzien van uw acitviteit?' is equal to 'Wielrennen' answer this question:***<br>Wielrennen	◯ Wielrennen
4.1.52	***If 'Wat is het meest bij u van toepassing ten aanzien van uw acitviteit?' is equal to 'Paardrijden' answer this question:***<br>Paardrijden	◯ Paardrijden
4.1.53	***If 'Wat is het meest bij u van toepassing ten aanzien van uw acitviteit?' is equal to 'Motorsporten/technische sporten' answer this question:***<br>Motorsporten/technische sporten	◯ Motorsporten/technische sporten
4.1.54	***If 'Wat is het meest bij u van toepassing ten aanzien van uw acitviteit?' is equal to 'Roeien, kayakken' answer this question:***<br>Roeien, kayakken	◯ Roeien, kayakken

4.1.55	***If 'Wat is het meest bij u van toepassing ten aanzien van uw acitviteit?' is equal to 'Boogschieten' answer this question:*** Boogschieten	◯ Boogschieten
4.1.56	***If 'Wat is het meest bij u van toepassing ten aanzien van uw acitviteit?' is equal to 'Water polo en zwemmen' answer this question:*** Water polo en zwemmen	◯ Water polo en zwemmen
4.1.57	***If 'Wat is het meest bij u van toepassing ten aanzien van uw acitviteit?' is equal to 'Kunnen lopen op oneven grond' answer this question:*** Kunnen lopen op oneven grond	◯ Kunnen lopen op oneven grond
4.1.58	***If 'Wat is het meest bij u van toepassing ten aanzien van uw acitviteit?' is equal to 'Geen sport, echter geen limitatie in dagelijkse activiteiten' answer this question:*** Geen sport, echter geen limitatie in dagelijkse activiteiten	◯ Geen sport, echter geen limitatie in dagelijkse activiteiten
4.1.59	***If 'Wat is het meest bij u van toepassing ten aanzien van uw acitviteit?' is equal to 'Kunnen lopen op even grond, maar de dagelijkste activiteiten zijn gelimiteerd' answer this question:*** Kunnen lopen op even grond, maar de dagelijkste activiteiten zijn gelimiteerd	◯ Kunnen lopen op even grond, maar de dagelijkste activiteiten zijn gelimiteerd
4.1.60	***If 'Wat is het meest bij u van toepassing ten aanzien van uw acitviteit?' is equal to 'Niet kunnen lopen, fysieke handicap wegens enkelproblemen' answer this question:*** Niet kunnen lopen, fysieke handicap wegens enkelproblemen	◯ Niet kunnen lopen, fysieke handicap wegens enkelproblemen

Vragenlijst PRIMA studie 26 weken - Hoe tevreden bent u over de enkelklachten?

Number	Question	Answers
5.1	Hoe tevreden bent u over de enkelklachten?	◯ Slecht<br>◯ Matig<br>◯ Goed<br>◯ Uitstekend

Vragenlijst PRIMA studie 26 weken - SF-36

Number	Question	Answers
	Deze vragenlijst gaat over uw standpunten t.a.v. uw gezondheid. Met behulp van deze gegevens kan worden bijgehouden hoe u zich voelt en hoe goed u in staat bent uw gebruikelijke bezigheden uit te voeren. Beantwoord elke vraag door een antwoord aan te klikken. Als u niet zeker weet hoe u een vraag moet beantwoorden, geef dan het best mogelijke antwoord.	
6.1	Hoe zou u over het algemeen uw gezondheid noemen?	◯ Uitstekend ◯ Zeer goed ◯ Goed ◯ Matig ◯ Slecht
6.2	Hoe beoordeelt u nu uw gezondheid over het algemeen vergeleken met een jaar geleden?	◯ Veel beter dan een jaar geleden ◯ Wat beter dan een jaar geleden ◯ Ongeveer hetzelfde als een jaar geleden ◯ Wat slechter dan een jaar geleden ◯ Veel slechter dan een jaar geleden
6.3	Wordt u door uw gezondheid op dit moment beperkt bij forse inspanning, zoals hardlopen, tillen van zware voorwerpen of een veeleisende sport beoefenen?	◯ Ja, ernstig beperkt ◯ Ja, een beetje beperkt ◯ Nee, helemaal niet beperkt
6.4	Wordt u door uw gezondheid op dit moment beperkt bij matige inspanning zoals een tafel verplaatsen, stofzuigen, zwemmen of fietsen?	◯ Ja, ernstig beperkt ◯ Ja, een beetje beperkt ◯ Nee, helemaal niet beperkt
6.5	Wordt u door uw gezondheid op dit moment beperkt bij boodschappen tillen of dragen?	◯ Ja, ernstig beperkt ◯ Ja, een beetje beperkt ◯ Nee, helemaal niet beperkt
6.6	Wordt u door uw gezondheid op dit moment beperkt bij een paar trappen oplopen?	◯ Ja, ernstig beperkt ◯ Ja, een beetje beperkt ◯ Nee, helemaal niet beperkt
6.7	Wordt u door uw gezondheid op dit moment beperkt bij één trap oplopen?	◯ Ja, ernstig beperkt ◯ Ja, een beetje beperkt ◯ Nee, helemaal niet beperkt
6.8	Wordt u door uw gezondheid op dit moment beperkt bij bukken, knielen of hurken?	◯ Ja, ernstig beperkt ◯ Ja, een beetje beperkt ◯ Nee, helemaal niet beperkt
6.9	Wordt u door uw gezondheid op dit moment beperkt bij meer dan één kilometer lopen?	◯ Ja, ernstig beperkt ◯ Ja, een beetje beperkt ◯ Nee, helemaal niet beperkt
6.10	Wordt u door uw gezondheid op dit moment beperkt bij een paar honderd meter lopen?	◯ Ja, ernstig beperkt ◯ Ja, een beetje beperkt ◯ Nee, helemaal niet beperkt

6.11	Wordt u door uw gezondheid op dit moment beperkt bij ongeveer honderd meter lopen?	◯ Ja, ernstig beperkt ◯ Ja, een beetje beperkt ◯ Nee, helemaal niet beperkt
6.12	Wordt u door uw gezondheid op dit moment beperkt bij uzelf wassen of aankleden?	◯ Ja, ernstig beperkt ◯ Ja, een beetje beperkt ◯ Nee, helemaal niet beperkt
6.13	U besteedde in de afgelopen 4 weken minder tijd aan werk of andere bezigheden	◯ Ja ◯ Nee
6.14	U heeft in de afgelopen 4 weken minder bereikt dan u zou willen	◯ Ja ◯ Nee
6.15	U was in de afgelopen 4 weken beperkt in het soort werk of andere bezigheden.	◯ Ja ◯ Nee
6.16	U had de afgelopen 4 weken moeite om uw werk of andere bezigheden uit te voeren (het kostte u bijvoorbeeld extra inspanning).	◯ Ja ◯ Nee
6.17	U besteedde in de afgelopen 4 weken minder tijd aan werk of andere bezigheden ten gevolge van emotionele problemen	◯ Ja ◯ Nee
6.18	U heeft in de afgelopen 4 weken minder bereikt dan u zou willen ten gevolge van emotionele problemen.	◯ Ja ◯ Nee
6.19	U deed de afgelopen 4 weken uw werk of andere bezigheden niet zo zorgvuldig als gewoonlijk ten gevolge van emotionele problemen.	◯ Ja ◯ Nee
6.20	In hoeverre hebben uw lichamelijke gezondheid of emotionele problemen u gedurende de afgelopen 4 weken gehinderd in uw normale omgang met familie, vrienden of buren, of bij activiteiten in groepsverband?	◯ Helemaal niet ◯ Enigszins ◯ Nogal ◯ Veel ◯ Heel erg veel
6.21	Hoeveel lichamelijke pijn heeft u de afgelopen 4 weken gehad?	◯ Geen<br>◯ Heel licht<br>◯ Licht<br>◯ Nogal<br>◯ Ernstig<br>◯ Heel ernstig
6.22	In welke mate bent u de afgelopen 4 weken door de pijn gehinderd in uw normale werk (zowel werk buitenshuis als huishoudelijk werk)?	◯ Helemaal niet<br>◯ Een klein beetje<br>◯ Nogal<br>◯ Veel<br>◯ Heel erg veel

6.23	Voelde u zich levenslustig?	◯ Altijd<br>◯ Meestal<br>◯ Vaak<br>◯ Soms<br>◯ Zelden<br>◯ Nooit
6.24	Was u erg zenuwachtig?	◯ Altijd<br>◯ Meestal<br>◯ Vaak<br>◯ Soms<br>◯ Zelden<br>◯ Nooit
6.25	Zat u zo in de put dat u niets kon?	◯ Altijd<br>◯ Meestal<br>◯ Vaak<br>◯ Soms<br>◯ Zelden<br>◯ Nooit
6.26	Voelde u zich rustig en tevreden?	◯ Altijd<br>◯ Meestal<br>◯ Vaak<br>◯ Soms<br>◯ Zelden<br>◯ Nooit
6.27	Had u veel energie?	◯ Altijd<br>◯ Meestal<br>◯ Vaak<br>◯ Soms<br>◯ Zelden<br>◯ Nooit
6.28	Voelde u zich somber en neerslachtig?	◯ Altijd<br>◯ Meestal<br>◯ Vaak<br>◯ Soms<br>◯ Zelden<br>◯ Nooit

6.29	Voelde u zich uitgeput?	◯ Altijd<br>◯ Meestal<br>◯ Vaak<br>◯ Soms<br>◯ Zelden<br>◯ Nooit
6.30	Was u een gelukkig mens?	◯ Altijd<br>◯ Meestal<br>◯ Vaak<br>◯ Soms<br>◯ Zelden<br>◯ Nooit
6.31	Voelde u zich moe?	◯ Altijd<br>◯ Meestal<br>◯ Vaak<br>◯ Soms<br>◯ Zelden<br>◯ Nooit
6.32	Hoe vaak hebben uw lichamelijke gezondheid of emotionele problemen u gedurende de afgelopen 4 weken gehinderd bij uw sociale activiteiten (zoals vrienden of familie bezoeken etc.)?	◯ Altijd<br>◯ Meestal<br>◯ Soms<br>◯ Zelden<br>◯ Nooit
6.33	Ik lijk wat gemakkelijker ziek te worden dan andere mensen	◯ Volkomen juist<br>◯ Grotendeels juist<br>◯ Weet ik niet<br>◯ Grotendeels onjuist<br>◯ Volkomen onjuist
6.34	Ik ben even gezond als andere mensen die ik ken	◯ Volkomen juist<br>◯ Grotendeels juist<br>◯ Weet ik niet<br>◯ Grotendeels onjuist<br>◯ Volkomen onjuist

6.35	Ik verwacht dat mijn gezondheid achteruit zal gaan	◯ Volkomen juist<br>◯ Grotendeels juist<br>◯ Weet ik niet<br>◯ Grotendeels onjuist<br>◯ Volkomen onjuist
6.36	Mijn gezondheid is uitstekend	◯ Volkomen juist<br>◯ Grotendeels juist<br>◯ Weet ik niet<br>◯ Grotendeels onjuist<br>◯ Volkomen onjuist

Vragenlijst PRIMA studie 26 weken - GAS

Number	Question	Answers
	Tijdens de eerste afspraak werd een duidelijk doel met u afgesproken. Dit werd tevens per email naar u gestuurd. Hoe is de situatie nu ten aanzien van de destijds gestelde doelstelling?	
7.1	Wat is nu de situatie ten aanzien van het tijdens de eerste afspraak afgesproken doelstelling (Goal attainment Scaling)?	◯ Achteruitgang (minder dan de utigangssituatie)<br>◯ Uitgangssituatie<br>◯ Minder dan het doel<br>◯ Doel<br>◯ Meer dan het doel<br>◯ Veel meer dan het doel

Vragenlijst PRIMA studie 26 weken - EQ-5D-3L

Number	Question	Answers
	Deze vragenlijst gaat over uw standpunten t.a.v. uw gezondheid. Met behulp van deze gegevens kan worden bijgehouden hoe u zich voelt en hoe goed u in staat bent uw gebruikelijke bezigheden uit te voeren. Beantwoord elke vraag door een antwoord aan te klikken. Als u niet zeker weet hoe u een vraag moet beantwoorden, geef dan het best mogelijke antwoord.	
8.1	Hoe is het met uw Mobiliteit gesteld?	◯ Ik heb geen problemen met lopen<br>◯ Ik heb enige problemen met lopen<br>◯ Ik ben bedlegerig

8.2	Hoe is het met uw Zelfzorg gesteld?	◯ Ik heb geen problemen om mijzelf te wassen of aan te kleden<br>◯ Ik heb enige problemen om mijzelf te wassen of aan te kleden<br>◯ Ik ben niet in staat om mijzelf te wassen of aan te kleden
8.3	Hoe is het met de Dagelijkse activiteiten (werk, studie, huishouden, gezins- en vrijetijdsactiviteiten) gesteld?	◯ Ik heb geen problemen met mijn dagelijkse activiteiten<br>◯ Ik heb enige problemen met mijn dagelijkse activiteiten<br>◯ Ik ben niet in staat om mijn dagelijkse activiteiten uit te voeren
8.4	Hoe is het met de Pijn/klachten gesteld?	◯ Ik heb geen pijn of andere klachten<br>◯ Ik heb matige pijn of andere klachten<br>◯ Ik heb zeer ernstige pijn of andere klachten
8.5	Hoe is het met de Stemming gesteld?	◯ Ik ben niet angstig of somber<br>◯ Ik ben matig angstig of somber<br>◯ Ik ben erg angstig of somber
	We willen weten hoe goed of slecht uw gezondheid VANDAAG is. Deze meetschaal loopt van 0 tot 100: 100 staat voor de BESTE gezondheid die u zich kunt voorstellen - 0 staat voor de SLECHTSTE gezondheid die u zich kunt voorstellen	
8.6	Uw Gezondheid vandaag	(0.00) (100.00)

Vragenlijst PRIMA studie 26 weken - AOS

Number	Question	Answers
	Instructies: De lijn naast elke vraag staat voor hoeveel PIJN u heeft in verschillende situaties. De linker kant (0) is “geen pijn” en de rechter kant (100) is “ergste pijn denkbaar”. Geef voor de onderstaande situaties op de lijn aan hoeveel PIJN u in de afgelopen week in de enkel had. Als een of meerdere van deze situaties niet van toepassing waren, dan kiest u de "niet van toepassing" optie.	
9.1	Wat was de hoogte van de ergste pijn in de afgelopen week?	(0.00) (100.00)
9.2	Heeft u pijn als u 's ochtends opstaat?	◯ Ja<br>◯ Nee<br>◯ Niet van toepassing

9.2.1	***If 'Heeft u pijn als u 's ochtends opstaat?' is equal to 'Ja' answer this question:*** Hoeveel pijn heeft u voordat u 's ochtends opstaat?	(0.00)	(100.00)
9.3	Heeft u pijn wanneer u op blote voeten loopt?	◯ Ja<br>◯ Nee<br>◯ Niet van toepassing	
9.3.1	***If 'Heeft u pijn wanneer u op blote voeten loopt?' is equal to 'Ja' answer this question:*** Hoeveel pijn heeft u als u op blote voeten loopt?	(0.00)	(100.00)
9.4	Heeft u pijn wanneer u op blote voeten staat?	◯ Ja<br>◯ Nee<br>◯ Niet van toepassing	
9.4.1	***If 'Heeft u pijn wanneer u op blote voeten staat?' is equal to 'Ja' answer this question:*** Hoeveel pijn heeft u als u op blote voeten staat?	(0.00)	(100.00)
9.5	Heeft u pijn wanneer u met schoenen loopt?	◯ Ja<br>◯ Nee<br>◯ Niet van toepassing	
9.5.1	***If 'Heeft u pijn wanneer u met schoenen loopt?' is equal to 'Ja' answer this question:*** Hoeveel pijn heeft u wanneer u met schoenen loopt?	(0.00)	(100.00)
9.6	Heeft u pijn wanneer u met schoenen staat?	◯ Ja<br>◯ Nee<br>◯ Niet van toepassing	
9.6.1	***If 'Heeft u pijn wanneer u met schoenen staat?' is equal to 'Ja' answer this question:*** Hoeveel pijn heeft u wanneer u met schoenen staat?	(0.00)	(100.00)
9.7	Heeft u pijn wanneer u loopt met steunzolen of een brace?	◯ Ja<br>◯ Nee<br>◯ Niet van toepassing	
9.7.1	***If 'Heeft u pijn wanneer u loopt met steunzolen of een brace?' is equal to 'Ja' answer this question:*** Hoeveel pijn heeft u wanneer u loopt met steunzolen of een brace?	(0.00)	(100.00)

9.8	Heeft u pijn wanneer u staat met steunzolen of een brace?	◯ Ja<br>◯ Nee<br>◯ Niet van toepassing	
9.8.1	***If 'Heeft u pijn wanneer u staat met steunzolen of een brace?' is equal to 'Ja' answer this question:***<br>Hoeveel pijn heeft u wanneer u staat met steunzolen of een brace?	(0.00)	(100.00)
9.9	Heeft u pijn aan het einde van de dag?	◯ Ja<br>◯ Nee<br>◯ Niet van toepassing	
9.9.1	***If 'Heeft u pijn aan het einde van de dag?' is equal to 'Ja' answer this question:***<br>Hoeveel pijn heeft u aan het einde van de dag?	(0.00)	(100.00)
	Instructies:De lijn naast elke vraag staat voor hoeveel MOEITE u heeft met verschillende activiteiten. De linker kant (0) is “Niet moeilijk” en de rechter kant (100) is “Te moeilijk, niet uitvoerbaar”. Geef voor de onderstaande activiteiten op de lijn aan hoeveel MOEITE u in de afgelopen week door enkelklachten had met onderstaande activiteiten. Als een situatie niet van toepassing is, kies dan de optie "niet van toepassing".		
9.10	Heeft u moeite met door het huis lopen?	◯ Ja<br>◯ Nee<br>◯ Niet van toepassing	
9.10.1	***If 'Heeft u moeite met door het huis lopen?' is equal to 'Ja' answer this question:***<br>Hoeveel moeite heeft u met door het huis lopen?	(0.00)	(100.00)
9.11	Heeft u moeite met buiten lopen op oneven ondergrond?	◯ Ja<br>◯ Nee<br>◯ Niet van toepassing	
9.11.1	***If 'Heeft u moeite met buiten lopen op oneven ondergrond?' is equal to 'Ja' answer this question:***<br>Hoeveel moeite heeft u met buiten lopen op oneven ondergrond?	(0.00)	(100.00)
9.12	Heeft u moeite met een paar honderd meter lopen?	◯ Ja<br>◯ Nee<br>◯ Niet van toepassing	
9.12.1	***If 'Heeft u moeite met een paar honderd meter lopen?' is equal to 'Ja' answer this question:***<br>Hoeveel moeite heeft u met een paar honderd meter lopen?	(0.00)	(100.00)

9.13	Heeft u moeite met een trap oplopen?	◯ Ja<br>◯ Nee<br>◯ Niet van toepassing	
9.13.1	***If 'Heeft u moeite met een trap oplopen?' is equal to 'Ja' answer this question:***<br>Hoeveel moeite heeft u met een trap oplopen?	(0.00)	(100.00)
9.14	Heeft u moeite met een trap aflopen?	◯ Ja<br>◯ Nee<br>◯ Niet van toepassing	
9.14.1	***If 'Heeft u moeite met een trap aflopen?' is equal to 'Ja' answer this question:***<br>Hoeveel moeite heeft u met een trap aflopen?	(0.00)	(100.00)
9.15	Heeft u moeite met op de tenen staan?	◯ Ja<br>◯ Nee<br>◯ Niet van toepassing	
9.15.1	***If 'Heeft u moeite met op de tenen staan?' is equal to 'Ja' answer this question:***<br>Hoeveel moeite heeft u met op de tenen staan?	(0.00)	(100.00)
9.16	Heeft u moeite met opstaan uit de stoel?	◯ Ja<br>◯ Nee<br>◯ Niet van toepassing	
9.16.1	***If 'Heeft u moeite met opstaan uit de stoel?' is equal to 'Ja' answer this question:***<br>Hoeveel moeite heeft u met opstaan uit de stoel?	(0.00)	(100.00)
9.17	Heeft u moeite met het op- of afstappen van stoepranden?	◯ Ja<br>◯ Nee<br>◯ Niet van toepassing	
9.17.1	***If 'Heeft u moeite met het op- of afstappen van stoepranden?' is equal to 'Ja' answer this question:***<br>Hoeveel moeite heeft u met het op- of afstappen van stoepranden?	(0.00)	(100.00)
9.18	Heeft u moeite met snel lopen of rennen?	◯ Ja<br>◯ Nee<br>◯ Niet van toepassing	

9.18.1	***If 'Heeft u moeite met snel lopen of rennen?' is equal to 'Ja' answer this question:***<br>Hoeveel moeite heeft u met snel lopen of rennen?	(0.00)	(100.00)

Vragenlijst PRIMA studie 26 weken - FAOS

Number	Question	Answers
	Deze lijst vraagt naar uw mening over uw voet/enkel. Uw antwoorden geven ons een beeld van uw voet/enkel klachten en hoe u in staat bent om alledaagse activiteiten uit te voeren in uw huidige situatie. Beantwoorden van een vraag doet u door het aanklikken van een vakje met het volgens u meest juiste antwoord (één vakje per vraag). Als u niet zeker weet hoe u een vraag moet beantwoorden, geeft u dan het antwoord dat volgens u het meest op uw situatie van toepassing is. Deze vraag heeft betrekking op het voorkomen van voet/enkel klachten in de afgelopen week.	
10.1	Is uw voet/enkel gezwollen?	◯ Nooit ◯ Zelden ◯ Soms ◯ Vaak ◯ Altijd
10.2	Voelt u gekraak of hoort u klikken of een ander vreemd geluid wanneer u de voet/enkel beweegt?	◯ Nooit ◯ Zelden ◯ Soms ◯ Vaak ◯ Altijd
10.3	Hapert uw enkel of blokkeert uw enkel ('op slot' gaan zitten) wanneer u deze beweegt?	◯ Nooit ◯ Zelden ◯ Soms ◯ Vaak ◯ Altijd
10.4	Kunt u de voet/enkel volledig strekken?	◯ Altijd ◯ Vaak ◯ Soms ◯ Zelden ◯ Nooit
10.5	Kunt u de voet volledig naar u toe buigen?	◯ Altijd ◯ Vaak ◯ Soms ◯ Zelden ◯ Nooit
10.6	In welke mate heeft u een stijf gevoel in de voet/enkel 's ochtends bij het wakker worden?	◯ Niet ◯ Mild ◯ Matig ◯ Ernstig ◯ Zeer ernstig
10.7	In welke mate heeft u een stijf gevoel in de voet/enkel na zitten, liggen of rusten later op de dag?	◯ Niet ◯ Mild ◯ Matig ◯ Ernstig ◯ Zeer ernstig
10.8	Hoe vaak heeft u pijn in uw voet/enkel?	◯ Nooit ◯ Maandelijks ◯ Wekelijks ◯ Dagelijks ◯ Altijd

10.9	Hoeveel voet/enkel pijn heeft u gehad in de afgelopen week bij draaien als uw voet/enkel op de grond staat?	◯ Geen<br>◯ Mild<br>◯ Matig<br>◯ Ernstig<br>◯ Zeer ernstig
10.10	Hoeveel voet/enkel pijn heeft u gehad in de afgelopen week bij het volledig uitstrekken van de voet/enkel?	◯ Geen<br>◯ Mild<br>◯ Matig<br>◯ Ernstig<br>◯ Zeer ernstig
10.11	Hoeveel voet/enkel pijn heeft u gehad in de afgelopen week bij het volledig naar u toe buigen/optrekken van de voet/enkel	◯ Geen<br>◯ Mild<br>◯ Matig<br>◯ Ernstig<br>◯ Zeer ernstig
10.12	Hoeveel voet/enkel pijn heeft u gehad in de afgelopen week bij het lopen op een vlakke ondergrond	◯ Geen<br>◯ Mild<br>◯ Matig<br>◯ Ernstig<br>◯ Zeer ernstig
10.13	Hoeveel voet/enkel pijn heeft u gehad in de afgelopen week bij het trap op- en trap af lopen?	◯ Geen<br>◯ Mild<br>◯ Matig<br>◯ Ernstig<br>◯ Zeer ernstig
10.14	Hoeveel voet/enkel pijn heeft u gehad in de afgelopen week 's nachts in bed?	◯ Geen<br>◯ Mild<br>◯ Matig<br>◯ Ernstig<br>◯ Zeer ernstig
10.15	Hoeveel voet/enkel pijn heeft u gehad in de afgelopen week bij het zitten of liggen?	◯ Geen<br>◯ Mild<br>◯ Matig<br>◯ Ernstig<br>◯ Zeer ernstig

10.16	Hoeveel voet/enkel pijn heeft u gehad in de afgelopen week bij het rechtop staan?	◯ Geen<br>◯ Mild<br>◯ Matig<br>◯ Ernstig<br>◯ Zeer ernstig
10.17	In welke mate werd u gehinderd bij het trap aflopen?	◯ Niet ◯ Mild ◯ Matig ◯ Ernstig ◯ Zeer ernstig
10.18	In welke mate werd u gehinderd bij het trap op lopen?	◯ Niet ◯ Mild ◯ Matig ◯ Ernstig ◯ Zeer ernstig
10.19	In welke mate werd u gehinderd als u vanuit een zittende positie ging staan?	◯ Niet ◯ Mild ◯ Matig ◯ Ernstig ◯ Zeer ernstig
10.20	In welke mate werd u gehinderd bij het staan?	◯ Niet ◯ Mild ◯ Matig ◯ Ernstig ◯ Zeer ernstig
10.21	In welke mate werd u gehinderd bij het naar de grond buigen/iets oprapen?	◯ Niet ◯ Mild ◯ Matig ◯ Ernstig ◯ Zeer ernstig
10.22	In welke mate werd u gehinderd bij het lopen op een vlakke ondergrond?	◯ Niet ◯ Mild ◯ Matig ◯ Ernstig ◯ Zeer ernstig
10.23	In welke mate werd u gehinderd bij het in- en uit de auto stappen?	◯ Niet ◯ Mild ◯ Matig ◯ Ernstig ◯ Zeer ernstig
10.24	In welke mate werd u gehinderd bij het boodschappen doen?	◯ Niet ◯ Mild ◯ Matig ◯ Ernstig ◯ Zeer ernstig
10.25	In welke mate werd u gehinderd bij sokken/panty's aantrekken?	◯ Niet ◯ Mild ◯ Matig ◯ Ernstig ◯ Zeer ernstig
10.26	In welke mate werd u gehinderd bij het opstaan uit bed?	◯ Niet ◯ Mild ◯ Matig ◯ Ernstig ◯ Zeer ernstig
10.27	In welke mate werd u gehinderd bij het sokken uittrekken?	◯ Niet ◯ Mild ◯ Matig ◯ Ernstig ◯ Zeer ernstig
10.28	In welke mate werd u gehinderd bij het in bed liggen (omdraaien, lange tijd uw voet/enkel in dezelfde positie houden)?	◯ Niet ◯ Mild ◯ Matig ◯ Ernstig ◯ Zeer ernstig

10.29	In welke mate werd u gehinderd bij het in/uit bad stappen?	◯ Niet ◯ Mild ◯ Matig ◯ Ernstig ◯ Zeer ernstig
10.30	In welke mate werd u gehinderd bij zitten?	◯ Niet ◯ Mild ◯ Matig ◯ Ernstig ◯ Zeer ernstig
10.31	In welke mate werd u gehinderd bij het toilet op en af gaan?	◯ Niet ◯ Mild ◯ Matig ◯ Ernstig ◯ Zeer ernstig
10.32	In welke mate werd u gehinderd bij zwaar huishoudelijk werk (bijvoorbeeld zware dozen sjouwen, vloer schrobben)?	◯ Niet ◯ Mild ◯ Matig ◯ Ernstig ◯ Zeer ernstig
10.33	In welke mate werd u gehinderd bij licht huishoudeijk werk (bijvoorbeeld koken, afstoffen)?	◯ Niet ◯ Mild ◯ Matig ◯ Ernstig ◯ Zeer ernstig
10.34	In welke mate werd u gehinderd bij hurken?	◯ Niet ◯ Mild ◯ Matig ◯ Ernstig ◯ Zeer ernstig
10.35	In welke mate werd u gehinderd bij hardlopen?	◯ Niet ◯ Mild ◯ Matig ◯ Ernstig ◯ Zeer ernstig
10.36	In welke mate werd u gehinderd bij springen?	◯ Niet ◯ Mild ◯ Matig ◯ Ernstig ◯ Zeer ernstig
10.37	In welke mate werd u gehinderd bij ronddraaien op uw aangedane voet/enkel?	◯ Niet ◯ Mild ◯ Matig ◯ Ernstig ◯ Zeer ernstig
10.38	In welke mate werd u gehinderd bij knielen?	◯ Niet ◯ Mild ◯ Matig ◯ Ernstig ◯ Zeer ernstig
10.39	Hoe vaak bent u zich bewust van uw voet/enkel probleem?	◯ Nooit ◯ Maandelijks ◯ Wekelijks ◯ Dagelijks ◯ Altijd
10.40	Heeft u uw leven veranderd om activiteiten te vermijden die schadelijk kunnen zijn voor uw voet/enkel?	◯ Niet<br>◯ Enigszins<br>◯ Matig<br>◯ Behoorlijk<br>◯ Volledig

10.41	In hoeverre kunt u op uw voet/enkel vertrouwen?	◯ Volledig<br>◯ Behoorlijk<br>◯ Matig<br>◯ Enigzins<br>◯ Niet
10.42	In het algemeen, in welke mate ondervindt u hinder van uw voet/enkel	◯ Geen<br>◯ Mild<br>◯ Matig<br>◯ Ernstig<br>◯ Zeer ernstig

Vragenlijst PRIMA studie 26 weken - PRODISQ - Gezondheid en werk

Number	Question	Answers
11.1	Wat is uw leeftijd?	[ ] jaar
11.2	Wat is uw geslacht?	◯ Man<br>◯ Vrouw
11.3	Wat is de hoogste opleiding die u heeft afgemaakt (Zoek uw hoogste opleiding en kruis het hokje daarvoor aan)?	☐ Ik heb geen school of opleiding afgemaakt<br>☐ Lagere school of basisschool<br>☐ Huishoudschool, vbo, lbo, lts, leao of lhno<br>☐ Mavo, mulo, ivo of vmbo<br>☐ Mbo, mts, meao, mhno, inas of intas<br>☐ Havo, vwo, hbs, mms, atheneum of gymnasium<br>☐ Hbo, hts, heao of hhno<br>☐ Universiteit<br>☐ Ik heb een andere opleiding afgemaakt, namelijk
11.3.1	***If 'Wat is de hoogste opleiding die u heeft afgemaakt (Zoek uw hoogste opleiding en kruis het hokje daarvoor aan)?' is equal to 'Ik heb een andere opleiding afgemaakt, namelijk' answer this question:***<br>Wat is de hoogste opleiding die u heeft afgemaakt?	[ ]

11.4	Wat doet u in het dagelijks leven?	☐ Ik zit op school, ik studeer<br>☐ Ik werk in loondienst<br>☐ Ik ben zelfstandig ondernemer<br>☐ Ik ben huisvrouw, huisman<br>☐ Ik ben werkloos<br>☐ Ik ben arbeidsongeschikt<br>☐ Ik ben met pensioen of prepensioen<br>☐ Ik doe iets anders, namelijk
11.4.1	***If 'Wat doet u in het dagelijks leven?' is equal to 'Ik ben arbeidsongeschikt' answer this question:***<br>Wat doet u in het dagelijks leven?	
11.4.2	***If 'Wat doet u in het dagelijks leven?' is equal to 'Ik doe iets anders, namelijk' answer this question:***<br>Wat doet u in het dagelijks leven?	
11.5	Hebt u betaald werk?	○ Nee<br>○ Ja
11.5.1	***If 'Hebt u betaald werk?' is equal to 'Ja' answer this question:***<br>Wat is uw beroep?	
11.5.2	***If 'Hebt u betaald werk?' is equal to 'Ja' answer this question:***<br>Hoeveel uur per week werkt u (Tel alleen de uren waarvoor u betaald wordt)?	______ uren
11.5.3	***If 'Hebt u betaald werk?' is equal to 'Ja' answer this question:***<br>Hoeveel dagen in de week werkt u?	______ Dagen
11.5.4	***If 'Hebt u betaald werk?' is equal to 'Ja' answer this question:***<br>Bent u in de afgelopen 4 weken afwezig geweest van uw werk omdat u ziek was?	○ Nee<br>○ Ja
11.5.4.1	***If 'Bent u in de afgelopen 4 weken afwezig geweest van uw werk omdat u ziek was?' is equal to 'Ja' answer this question:***<br>Bent u in de afgelopen 4 weken afwezig geweest van uw werk omdat u ziek was (Tel alleen de werkdagen in de afgelopen 4 weken)?	______ dagen afwezig geweest
11.5.4.2	***If 'Bent u in de afgelopen 4 weken afwezig geweest van uw werk omdat u ziek was?' is equal to 'Ja' answer this question:***<br>Was u langer dan de gehele periode van 4 weken afwezig van uw werk doordat u ziek was?	○ Nee<br>○ Ja

11.5.4.2.1	***If 'Was u langer dan de gehele periode van 4 weken afwezig van uw werk doordat u ziek was?' is equal to 'Ja' answer this question:*** Wanneer heeft u zich ziek gemeld?	[ ] [ ] [ ] *(dd-mm-yyyy)*
11.5.5	***If 'Hebt u betaald werk?' is equal to 'Ja' answer this question:*** Waren er in de afgelopen 4 weken, dagen waarop u wel gewerkt heeft, maar tijdens uw werk last had van lichamelijke of psychische problemen?	◯ Ja<br>◯ Nee
11.5.5.1	***If 'Waren er in de afgelopen 4 weken, dagen waarop u wel gewerkt heeft, maar tijdens uw werk last had van lichamelijke of psychische problemen?' is equal to 'Ja' answer this question:*** Op hoeveel werkdagen had u tijdens uw werk last van uw lichamelijke of psychische problemen?	[ ] werkdagen
11.5.5.2	***If 'Waren er in de afgelopen 4 weken, dagen waarop u wel gewerkt heeft, maar tijdens uw werk last had van lichamelijke of psychische problemen?' is equal to 'Ja' answer this question:*** Op de dagen dat u last had, kon u misschien niet zoveel werk doen als normaal. Hoeveel werk kon u op deze dagen gemiddeld doen?	Ik kon op deze dagen niks doen (0.00) — Ik kon net zoveel doen als normaal (10.00)
11.6	Waren er dagen waarop u minder onbetaald werk kon doen door uw lichamelijke of psychische problemen?	◯ Nee<br>◯ Ja
11.6.1	***If 'Waren er dagen waarop u minder onbetaald werk kon doen door uw lichamelijke of psychische problemen?' is equal to 'Ja' answer this question:*** Op hoeveel dagen was dit zo?	[ ] Dagen
11.6.2	***If 'Waren er dagen waarop u minder onbetaald werk kon doen door uw lichamelijke of psychische problemen?' is equal to 'Ja' answer this question:*** Stel dat iemand, bijvoorbeeld uw partner, familielid of een bekende, u op deze dagen had geholpen. En al het onbetaalde werk wat u niet kon doen, voor u had gedaan. Hoeveel uur was die persoon hier op deze dagen dan gemiddeld mee bezig geweest?	[ ] uur op deze dagen

Vragenlijst PRIMA studie 26 weken - PRODISQ - Zorggebruik

Number	Question	Answers
	Wij willen graag weten met welke dokters u in de afgelopen 3 maanden een afspraak had. Het gaat om afspraken voor uzelf. Ook andere zorgverleners tellen mee. Bijvoorbeeld de fysiotherapeut.	

12.1	Bent u in de afgelopen 3 maanden naar uw huisarts geweest?	◯ Nee<br>◯ Ja
12.1.1	***If 'Bent u in de afgelopen 3 maanden naar uw huisarts geweest?' is equal to 'Ja' answer this question:***<br>Hoeveel afspraken had u?	[ ] afspraken
12.2	Bent u in de afgelopen 3 maanden in contact geweest met een maatschappelijk werker?	◯ Nee<br>◯ Ja
12.2.1	***If 'Bent u in de afgelopen 3 maanden in contact geweest met een maatschappelijk werker?' is equal to 'Ja' answer this question:***<br>Hoeveel afspraken had u?	[ ] afspraken
12.3	Bent u in de afgelopen 3 maanden naar een fysiotherapeut geweest? Of een caesartherapeut, therapeut mensendieck of een manueel therapeut?	◯ Nee<br>◯ Ja
12.3.1	***If 'Bent u in de afgelopen 3 maanden naar een fysiotherapeut geweest? Of een caesartherapeut, therapeut mensendieck of een manueel therapeut?' is equal to 'Ja' answer this question:***<br>Hoeveel afspraken had u?	[ ] afspraken
12.4	Bent u in de afgelopen 3 maanden naar een ergotherapeut geweest?	◯ Nee<br>◯ Ja
12.4.1	***If 'Bent u in de afgelopen 3 maanden naar een ergotherapeut geweest?' is equal to 'Ja' answer this question:***<br>Hoeveel afspraken had u?	[ ] afspraken
12.5	Bent u in de afgelopen 3 maanden naar een logopedist geweest?	◯ Nee<br>◯ Ja
12.5.1	***If 'Bent u in de afgelopen 3 maanden naar een logopedist geweest?' is equal to 'Ja' answer this question:***<br>Hoeveel afspraken had u?	[ ] afspraken
12.6	Bent u in de afgelopen 3 maanden naar een diëtist geweest?	◯ Nee<br>◯ Ja
12.6.1	***If 'Bent u in de afgelopen 3 maanden naar een diëtist geweest?' is equal to 'Ja' answer this question:***<br>Hoeveel afspraken had u?	[ ] afspraken

12.7	Bent u in de afgelopen 3 maanden naar een homeopaat geweest? Of een acupuncturist?	◯ Nee<br>◯ Ja
12.7.1	***If 'Bent u in de afgelopen 3 maanden naar een homeopaat geweest? Of een acupuncturist?' is equal to 'Ja' answer this question:***<br>Hoeveel afspraken had u?	[ ] afspraken
12.8	Bent u in de afgelopen 3 maanden naar een psycholoog geweest? Of een psychotherapeut of psychiater?	◯ Nee<br>◯ Ja
12.8.1	***If 'Bent u in de afgelopen 3 maanden naar een psycholoog geweest? Of een psychotherapeut of psychiater?' is equal to 'Ja' answer this question:***<br>Hoeveel afspraken had u?	[ ] afspraken
12.9	Heeft u in de afgelopen 3 maanden afspraken gehad met de bedrijfsarts?	◯ Nee<br>◯ Ja
12.9.1	***If 'Heeft u in de afgelopen 3 maanden afspraken gehad met de bedrijfsarts?' is equal to 'Ja' answer this question:***<br>Hoeveel afspraken had u?	[ ] afspraken
12.10	Heeft u in de afgelopen 3 maanden hulp van de thuiszorg gehad?	◯ Ja<br>◯ Nee
12.10.1	***If 'Heeft u in de afgelopen 3 maanden hulp van de thuiszorg gehad?' is equal to 'Ja' answer this question:***<br>Wat voor hulp van de thuiszorg heeft u gehad in de afgelopen 3 maanden?	☐ Huishoudelijke hulp; voorbeeld: stofzuigen, bed opmaken, boodschappen doen<br>☐ Verzorging van uzelf; voorbeeld: hulp bij douchen of aankleden<br>☐ Verpleging; voorbeeld: verband omdoen, medicijnen geven, bloeddruk meten
12.10.1.1	***If 'Wat voor hulp van de thuiszorg heeft u gehad in de afgelopen 3 maanden?' is equal to 'Huishoudelijke hulp; voorbeeld: stofzuigen, bed opmaken, boodschappen doen' answer this question:***<br>Hoeveel weken heeft u deze thuiszorg gehad? Huishoudelijke hulp:	[ ] weken in de afgelopen 3 maanden
12.10.1.2	***If 'Wat voor hulp van de thuiszorg heeft u gehad in de afgelopen 3 maanden?' is equal to 'Verzorging van uzelf; voorbeeld: hulp bij douchen of aankleden' answer this question:***<br>Hoeveel weken heeft u deze thuiszorg gehad? Verzorging van uzelf:	[ ] weken in de afgelopen 3 maanden

12.10.1.3	***If 'Wat voor hulp van de thuiszorg heeft u gehad in de afgelopen 3 maanden?' is equal to 'Verpleging; voorbeeld: verband omdoen, medicijnen geven, bloeddruk meten' answer this question:*** Hoeveel weken heeft u deze thuiszorg gehad? Verpleging:	______ weken in de afgelopen 3 maanden
12.10.1.4	***If 'Wat voor hulp van de thuiszorg heeft u gehad in de afgelopen 3 maanden?' is equal to 'Huishoudelijke hulp; voorbeeld: stofzuigen, bed opmaken, boodschappen doen' answer this question:*** Hoeveel uur thuiszorg kreeg u in deze weken gemiddeld? Huishoudelijke hulp: gemiddeld	______ uur in de week
12.10.1.5	***If 'Wat voor hulp van de thuiszorg heeft u gehad in de afgelopen 3 maanden?' is equal to 'Verzorging van uzelf; voorbeeld: hulp bij douchen of aankleden' answer this question:*** Hoeveel uur thuiszorg kreeg u in deze weken gemiddeld? Verzorging van uzelf: gemiddeld	______ uur in de week
12.10.1.6	***If 'Wat voor hulp van de thuiszorg heeft u gehad in de afgelopen 3 maanden?' is equal to 'Verpleging; voorbeeld: verband omdoen, medicijnen geven, bloeddruk meten' answer this question:*** Hoeveel uur thuiszorg kreeg u in deze weken gemiddeld? Verpleging: gemiddeld	______ uur in de week
12.11	Heeft u in de afgelopen 3 maanden medicijnen gebruikt?	○ Ja<br>○ Nee
12.11.1	***If 'Heeft u in de afgelopen 3 maanden medicijnen gebruikt?' is equal to 'Ja' answer this question:*** Welke medicijnen heeft u in de afgelopen 3 maanden gebruikt?	______
12.12	Bent u in de afgelopen 3 maanden op de spoedeisende eerste hulp van een ziekenhuis geweest? (Een andere naam voor spoedeisende eerste hulp is EHBO)	○ Nee<br>○ Ja
12.12.1	***If 'Bent u in de afgelopen 3 maanden op de spoedeisende eerste hulp van een ziekenhuis geweest? (Een andere naam voor spoedeisende eerste hulp is EHBO)' is equal to 'Ja' answer this question:*** Hoevaak bent u geweest?	______ keer
12.13	Bent u in de afgelopen 3 maanden met een ambulance naar het ziekenhuis gebracht? (Een andere naam voor ambulance is ziekenauto)	○ Nee<br>○ Ja

12.13.1	***If 'Bent u in de afgelopen 3 maanden met een ambulance naar het ziekenhuis gebracht? (Een andere naam voor ambulance is ziekenauto)' is equal to 'Ja' answer this question:***<br>Hoe vaak bent u naar het ziekenhuis gebracht?	☐ keer
12.14	Had u in de afgelopen 3 maanden een afspraak bij de polikliniek van het ziekenhuis? (Het gaat om afspraken voor uzelf met een dokter. Bijvoorbeeld met de cardioloog, reumatoloog of neuroloog)	◯ Ja<br>◯ Nee
12.14.1	***If 'Had u in de afgelopen 3 maanden een afspraak bij de polikliniek van het ziekenhuis? (Het gaat om afspraken voor uzelf met een dokter. Bijvoorbeeld met de cardioloog, reumatoloog of neuroloog)' is equal to 'Ja' answer this question:***<br>Bij welke soorten dokters bent u in de afgelopen 3 maanden in het ziekenhuis geweest? En hoe vaak?	☐
12.15	Bent u in de afgelopen 3 maanden overdag in het ziekenhuis geweest voor een behandeling? (U bleef dus niet slapen. U kwam bijvoorbeeld voor een bloedtransfusie, nierdialyse of chemokuur)	◯ Ja<br>◯ Nee
12.15.1	***If 'Bent u in de afgelopen 3 maanden overdag in het ziekenhuis geweest voor een behandeling? (U bleef dus niet slapen. U kwam bijvoorbeeld voor een bloedtransfusie, nierdialyse of chemokuur)' is equal to 'Ja' answer this question:***<br>Voor welke soort behandeling was dit?	☐
12.15.2	***If 'Bent u in de afgelopen 3 maanden overdag in het ziekenhuis geweest voor een behandeling? (U bleef dus niet slapen. U kwam bijvoorbeeld voor een bloedtransfusie, nierdialyse of chemokuur)' is equal to 'Ja' answer this question:***<br>Hoeveel keer moest u in de afgelopen 3 maanden voor deze behandelingen naar het ziekenhuis?	☐
12.16	Bent u in de afgelopen 3 maanden ergens anders geweest voor een behandeling overdag? (U bleef dus niet slapen. U ging bijvoorbeeld naar de dagopvang van een woon-/zorgcentrum of een psychiatrische instelling. Of naar de dagbehandeling van een revalidatiecentrum)	◯ Ja<br>◯ Nee
12.16.1	***If 'Bent u in de afgelopen 3 maanden ergens anders geweest voor een behandeling overdag? (U bleef dus niet slapen. U ging bijvoorbeeld naar de dagopvang van een woon-/zorgcentrum of een psychiatrische instelling. Of naar de dagbehandeling van een revalidatiecentrum)' is equal to 'Ja' answer this question:***<br>Wat voor instelling was dit?	☐ Woon-/zorgcentrum<br>☐ Revalidatiecentrum<br>☐ Psychiatrische instelling<br>☐ Een andere instelling, namelijk

12.16.1.1	***If 'Wat voor instelling was dit?' is equal to 'Een andere instelling, namelijk' answer this question:*** Wat voor instelling was dit?	
12.16.1.2	***If 'Wat voor instelling was dit?' is equal to 'Woon-/zorgcentrum' answer this question:*** Hoe vaak moest u hier in de afgelopen 3 maanden naartoe? Naar het woon-/zorgcentrum:	keer in de afgelopen 3 maanden
12.16.1.3	***If 'Wat voor instelling was dit?' is equal to 'Revalidatiecentrum' answer this question:*** Hoe vaak moest u hier in de afgelopen 3 maanden naartoe? Naar het revalidatiecentrum:	keer in de afgelopen 3 maanden
12.16.1.4	***If 'Wat voor instelling was dit?' is equal to 'Psychiatrische instelling' answer this question:*** Hoe vaak moest u hier in de afgelopen 3 maanden naartoe? Naar de psychiatrische instelling:	keer in de afgelopen 3 maanden
12.16.1.5	***If 'Wat voor instelling was dit?' is equal to 'Een andere instelling, namelijk' answer this question:*** Hoe vaak moest u hier in de afgelopen 3 maanden naartoe? Naar de andere instelling:	keer in de afgelopen 3 maanden
12.17	Heeft u in de afgelopen 3 maanden weleens in het ziekenhuis gelegen? (U moest dus blijven slapen. Bijvoorbeeld omdat u geopereerd was en niet meteen naar huis kon)	◯ Ja<br>◯ Nee
12.17.1	***If 'Heeft u in de afgelopen 3 maanden weleens in het ziekenhuis gelegen? (U moest dus blijven slapen. Bijvoorbeeld omdat u geopereerd was en niet meteen naar huis kon)' is equal to 'Ja' answer this question:*** Hoe vaak heeft u in de afgelopen 3 maanden in het ziekenhuis gelegen?	keer in de afgelopen 3 maanden
12.17.2	***If 'Heeft u in de afgelopen 3 maanden weleens in het ziekenhuis gelegen? (U moest dus blijven slapen. Bijvoorbeeld omdat u geopereerd was en niet meteen naar huis kon)' is equal to 'Ja' answer this question:*** Hoe lang heeft u in het ziekenhuis gelegen?	dagen in de afgelopen 3 maanden
12.18	Moest u in de afgelopen 3 maanden ergens anders blijven slapen voor uw gezondheid? (Bijvoorbeeld in een woon-/zorgcentrum, psychiatrische instelling of revalidatiecentrum)	◯ Ja<br>◯ Nee

12.18.1	***If 'Moest u in de afgelopen 3 maanden ergens anders blijven slapen voor uw gezondheid? (Bijvoorbeeld in een woon-/zorgcentrum, psychiatrische instelling of revalidatiecentrum)' is equal to 'Ja' answer this question:*** Wat voor instelling was dit?	☐ Woon-/zorgcentrum<br>☐ Revalidatiecentrum<br>☐ Psychiatrische instelling<br>☐ Een andere instelling, namelijk
12.18.1.1	***If 'Wat voor instelling was dit?' is equal to 'Een andere instelling, namelijk' answer this question:*** Een andere instelling, namelijk	
12.18.1.2	***If 'Wat voor instelling was dit?' is equal to 'Woon-/zorgcentrum' answer this question:*** Hoe lang bent u in deze instelling geweest? In het woon-/zorgcentrum:	____ dagen in de afgelopen 3 maanden
12.18.1.3	***If 'Wat voor instelling was dit?' is equal to 'Revalidatiecentrum' answer this question:*** Hoe lang bent u in deze instelling geweest? In het revalidatiecentrum:	____ dagen in de afgelopen 3 maanden
12.18.1.4	***If 'Wat voor instelling was dit?' is equal to 'Psychiatrische instelling' answer this question:*** Hoe lang bent u in deze instelling geweest? In de psychiatrische instelling:	____ dagen in de afgelopen 3 maanden
12.18.1.5	***If 'Wat voor instelling was dit?' is equal to 'Een andere instelling, namelijk' answer this question:*** Hoe lang bent u in deze instelling geweest? In de andere instelling:	____ dagen in de afgelopen 3 maanden
12.19	Heeft u in de afgelopen 3 maanden hulp gekregen van een familielid of een bekende vanwege uw lichamelijke of psychische problemen?	○ Ja<br>○ Nee
12.19.1	***If 'Heeft u in de afgelopen 3 maanden hulp gekregen van een familielid of een bekende vanwege uw lichamelijke of psychische problemen?' is equal to 'Ja' answer this question:*** Wat voor hulp van familieleden of bekenden heeft u gehad in de afgelopen 3 maanden?	☐ Huishoudelijke hulp - voorbeeld: stofzuigen, bed opmaken, boodschappen doen, klaarmaken van eten en drinken, verzorgen van kinderen<br>☐ Verzorging van uzelf - voorbeeld: hulp bij douchen of aankleden, hulp bij het eten en drinken of het geven van medicijnen<br>☐ Praktische hulp - voorbeeld: ondersteuning bij wandelen, het maken van uitstapjes of bezoekjes aan bekenden, bezoeken aan de huisarts of het ziekenhuis, het regelen van hulp of het regelen van financiële zaken

12.19.1.1	***If 'Wat voor hulp van familieleden of bekenden heeft u gehad in de afgelopen 3 maanden?' is equal to 'Huishoudelijke hulp - voorbeeld: stofzuigen, bed opmaken, boodschappen doen, klaarmaken van eten en drinken, verzorgen van kinderen' answer this question:***<br>Hoeveel weken heeft u deze hulp gehad? Huishoudelijke hulp:	weken in de afgelopen 3 maanden
12.19.1.2	***If 'Wat voor hulp van familieleden of bekenden heeft u gehad in de afgelopen 3 maanden?' is equal to 'Verzorging van uzelf - voorbeeld: hulp bij douchen of aankleden, hulp bij het eten en drinken of het geven van medicijnen' answer this question:***<br>Hoeveel weken heeft u deze hulp gehad? Verzorging van uzelf:	weken in de afgelopen 3 maanden
12.19.1.3	***If 'Wat voor hulp van familieleden of bekenden heeft u gehad in de afgelopen 3 maanden?' is equal to 'Praktische hulp - voorbeeld: ondersteuning bij wandelen, het maken van uitstapjes of bezoekjes aan bekenden, bezoeken aan de huisarts of het ziekenhuis, het regelen van hulp of het regelen van financiële zaken' answer this question:***<br>Hoeveel uur hulp kreeg u in deze weken gemiddeld? Praktische hulp:	weken in de afgelopen 3 maanden
12.19.1.4	***If 'Wat voor hulp van familieleden of bekenden heeft u gehad in de afgelopen 3 maanden?' is equal to 'Huishoudelijke hulp - voorbeeld: stofzuigen, bed opmaken, boodschappen doen, klaarmaken van eten en drinken, verzorgen van kinderen' answer this question:***<br>Hoeveel uur hulp kreeg u in deze weken gemiddeld? Huishoudelijke hulp: gemiddeld	uur in de week
12.19.1.5	***If 'Wat voor hulp van familieleden of bekenden heeft u gehad in de afgelopen 3 maanden?' is equal to 'Verzorging van uzelf - voorbeeld: hulp bij douchen of aankleden, hulp bij het eten en drinken of het geven van medicijnen' answer this question:***<br>Hoeveel uur hulp kreeg u in deze weken gemiddeld? Verzorging van uzelf: gemiddeld	uur in de week
12.19.1.6	***If 'Wat voor hulp van familieleden of bekenden heeft u gehad in de afgelopen 3 maanden?' is equal to 'Praktische hulp - voorbeeld: ondersteuning bij wandelen, het maken van uitstapjes of bezoekjes aan bekenden, bezoeken aan de huisarts of het ziekenhuis, het regelen van hulp of het regelen van financiële zaken' answer this question:***<br>Hoeveel uur hulp kreeg u in deze weken gemiddeld? Praktische hulp: gemiddeld	uur in de week

Vragenlijst PRIMA studie 26 weken - Dank

Number	Question	Answers
13.1	Hartelijk dank voor de tijd die u heeft genomen om de vragenlijsten in te vullen. Indien u nog opmerkingen heeft kunt u deze hierin plaatsen.	[ ]

Survey 'Vragenlijst PRIMA studie 39 weken'

Vragenlijst PRIMA studie 39 weken - PRODISQ - Gezondheid en werk

Number	Question	Answers
1.1	Wat is uw leeftijd?	[ ] jaar
1.2	Wat is uw geslacht?	◯ Man<br>◯ Vrouw
1.3	Wat is de hoogste opleiding die u heeft afgemaakt (Zoek uw hoogste opleiding en kruis het hokje daarvoor aan)?	☐ Ik heb geen school of opleiding afgemaakt<br>☐ Lagere school of basisschool<br>☐ Huishoudschool, vbo, lbo, lts, leao of lhno<br>☐ Mavo, mulo, ivo of vmbo<br>☐ Mbo, mts, meao, mhno, inas of intas<br>☐ Havo, vwo, hbs, mms, atheneum of gymnasium<br>☐ Hbo, hts, heao of hhno<br>☐ Universiteit<br>☐ Ik heb een andere opleiding afgemaakt, namelijk
1.3.1	***If 'Wat is de hoogste opleiding die u heeft afgemaakt (Zoek uw hoogste opleiding en kruis het hokje daarvoor aan)?' is equal to 'Ik heb een andere opleiding afgemaakt, namelijk' answer this question:***<br>Wat is de hoogste opleiding die u heeft afgemaakt?	[ ]

1.4	Wat doet u in het dagelijks leven?	☐ Ik zit op school, ik studeer<br>☐ Ik werk in loondienst<br>☐ Ik ben zelfstandig ondernemer<br>☐ Ik ben huisvrouw, huisman<br>☐ Ik ben werkloos<br>☐ Ik ben arbeidsongeschikt<br>☐ Ik ben met pensioen of prepensioen<br>☐ Ik doe iets anders, namelijk
1.4.1	***If 'Wat doet u in het dagelijks leven?' is equal to 'Ik ben arbeidsongeschikt' answer this question:***<br>Wat doet u in het dagelijks leven?	
1.4.2	***If 'Wat doet u in het dagelijks leven?' is equal to 'Ik doe iets anders, namelijk' answer this question:***<br>Wat doet u in het dagelijks leven?	
1.5	Hebt u betaald werk?	○ Nee<br>○ Ja
1.5.1	***If 'Hebt u betaald werk?' is equal to 'Ja' answer this question:***<br>Wat is uw beroep?	
1.5.2	***If 'Hebt u betaald werk?' is equal to 'Ja' answer this question:***<br>Hoeveel uur per week werkt u (Tel alleen de uren waarvoor u betaald wordt)?	uren
1.5.3	***If 'Hebt u betaald werk?' is equal to 'Ja' answer this question:***<br>Hoeveel dagen in de week werkt u?	Dagen
1.5.4	***If 'Hebt u betaald werk?' is equal to 'Ja' answer this question:***<br>Bent u in de afgelopen 4 weken afwezig geweest van uw werk omdat u ziek was?	○ Nee<br>○ Ja
1.5.4.1	***If 'Bent u in de afgelopen 4 weken afwezig geweest van uw werk omdat u ziek was?' is equal to 'Ja' answer this question:***<br>Bent u in de afgelopen 4 weken afwezig geweest van uw werk omdat u ziek was (Tel alleen de werkdagen in de afgelopen 4 weken)?	dagen afwezig geweest
1.5.4.2	***If 'Bent u in de afgelopen 4 weken afwezig geweest van uw werk omdat u ziek was?' is equal to 'Ja' answer this question:***<br>Was u langer dan de gehele periode van 4 weken afwezig van uw werk doordat u ziek was?	○ Nee<br>○ Ja

1.5.4.2.1	***If 'Was u langer dan de gehele periode van 4 weken afwezig van uw werk doordat u ziek was?' is equal to 'Ja' answer this question:***<br>Wanneer heeft u zich ziek gemeld?	[ ] [ ] [ ] *(dd-mm-yyyy)*
1.5.5	***If 'Hebt u betaald werk?' is equal to 'Ja' answer this question:***<br>Waren er in de afgelopen 4 weken, dagen waarop u wel gewerkt heeft, maar tijdens uw werk last had van lichamelijke of psychische problemen?	◯ Ja<br>◯ Nee
1.5.5.1	***If 'Waren er in de afgelopen 4 weken, dagen waarop u wel gewerkt heeft, maar tijdens uw werk last had van lichamelijke of psychische problemen?' is equal to 'Ja' answer this question:***<br>Op hoeveel werkdagen had u tijdens uw werk last van uw lichamelijke of psychische problemen?	[ ] werkdagen
1.5.5.2	***If 'Waren er in de afgelopen 4 weken, dagen waarop u wel gewerkt heeft, maar tijdens uw werk last had van lichamelijke of psychische problemen?' is equal to 'Ja' answer this question:***<br>Op de dagen dat u last had, kon u misschien niet zoveel werk doen als normaal. Hoeveel werk kon u op deze dagen gemiddeld doen?	Ik kon op deze dagen niks doen (0.00) … Ik kon net zoveel doen als normaal (10.00)
1.6	Waren er dagen waarop u minder onbetaald werk kon doen door uw lichamelijke of psychische problemen?	◯ Nee<br>◯ Ja
1.6.1	***If 'Waren er dagen waarop u minder onbetaald werk kon doen door uw lichamelijke of psychische problemen?' is equal to 'Ja' answer this question:***<br>Op hoeveel dagen was dit zo?	[ ] Dagen
1.6.2	***If 'Waren er dagen waarop u minder onbetaald werk kon doen door uw lichamelijke of psychische problemen?' is equal to 'Ja' answer this question:***<br>Stel dat iemand, bijvoorbeeld uw partner, familielid of een bekende, u op deze dagen had geholpen. En al het onbetaalde werk wat u niet kon doen, voor u had gedaan. Hoeveel uur was die persoon hier op deze dagen dan gemiddeld mee bezig geweest?	[ ] uur op deze dagen

Vragenlijst PRIMA studie 39 weken - PRODISQ - Zorggebruik

Number	Question	Answers
	Wij willen graag weten met welke dokters u in de afgelopen 3 maanden een afspraak had. Het gaat om afspraken voor uzelf. Ook andere zorgverleners tellen mee. Bijvoorbeeld de fysiotherapeut.	

2.1	Bent u in de afgelopen 3 maanden naar uw huisarts geweest?	◯ Nee<br>◯ Ja
2.1.1	***If 'Bent u in de afgelopen 3 maanden naar uw huisarts geweest?' is equal to 'Ja' answer this question:***<br>Hoeveel afspraken had u?	______ afspraken
2.2	Bent u in de afgelopen 3 maanden in contact geweest met een maatschappelijk werker?	◯ Nee<br>◯ Ja
2.2.1	***If 'Bent u in de afgelopen 3 maanden in contact geweest met een maatschappelijk werker?' is equal to 'Ja' answer this question:***<br>Hoeveel afspraken had u?	______ afspraken
2.3	Bent u in de afgelopen 3 maanden naar een fysiotherapeut geweest? Of een caesartherapeut, therapeut mensendieck of een manueel therapeut?	◯ Nee<br>◯ Ja
2.3.1	***If 'Bent u in de afgelopen 3 maanden naar een fysiotherapeut geweest? Of een caesartherapeut, therapeut mensendieck of een manueel therapeut?' is equal to 'Ja' answer this question:***<br>Hoeveel afspraken had u?	______ afspraken
2.4	Bent u in de afgelopen 3 maanden naar een ergotherapeut geweest?	◯ Nee<br>◯ Ja
2.4.1	***If 'Bent u in de afgelopen 3 maanden naar een ergotherapeut geweest?' is equal to 'Ja' answer this question:***<br>Hoeveel afspraken had u?	______ afspraken
2.5	Bent u in de afgelopen 3 maanden naar een logopedist geweest?	◯ Nee<br>◯ Ja
2.5.1	***If 'Bent u in de afgelopen 3 maanden naar een logopedist geweest?' is equal to 'Ja' answer this question:***<br>Hoeveel afspraken had u?	______ afspraken
2.6	Bent u in de afgelopen 3 maanden naar een diëtist geweest?	◯ Nee<br>◯ Ja
2.6.1	***If 'Bent u in de afgelopen 3 maanden naar een diëtist geweest?' is equal to 'Ja' answer this question:***<br>Hoeveel afspraken had u?	______ afspraken

2.7	Bent u in de afgelopen 3 maanden naar een homeopaat geweest? Of een acupuncturist?	◯ Nee<br>◯ Ja
2.7.1	***If 'Bent u in de afgelopen 3 maanden naar een homeopaat geweest? Of een acupuncturist?' is equal to 'Ja' answer this question:***<br>Hoeveel afspraken had u?	[ ] afspraken
2.8	Bent u in de afgelopen 3 maanden naar een psycholoog geweest? Of een psychotherapeut of psychiater?	◯ Nee<br>◯ Ja
2.8.1	***If 'Bent u in de afgelopen 3 maanden naar een psycholoog geweest? Of een psychotherapeut of psychiater?' is equal to 'Ja' answer this question:***<br>Hoeveel afspraken had u?	[ ] afspraken
2.9	Heeft u in de afgelopen 3 maanden afspraken gehad met de bedrijfsarts?	◯ Nee<br>◯ Ja
2.9.1	***If 'Heeft u in de afgelopen 3 maanden afspraken gehad met de bedrijfsarts?' is equal to 'Ja' answer this question:***<br>Hoeveel afspraken had u?	[ ] afspraken
2.10	Heeft u in de afgelopen 3 maanden hulp van de thuiszorg gehad?	◯ Ja<br>◯ Nee
2.10.1	***If 'Heeft u in de afgelopen 3 maanden hulp van de thuiszorg gehad?' is equal to 'Ja' answer this question:***<br>Wat voor hulp van de thuiszorg heeft u gehad in de afgelopen 3 maanden?	☐ Huishoudelijke hulp; voorbeeld: stofzuigen, bed opmaken, boodschappen doen<br>☐ Verzorging van uzelf; voorbeeld: hulp bij douchen of aankleden<br>☐ Verpleging; voorbeeld: verband omdoen, medicijnen geven, bloeddruk meten
2.10.1.1	***If 'Wat voor hulp van de thuiszorg heeft u gehad in de afgelopen 3 maanden?' is equal to 'Huishoudelijke hulp; voorbeeld: stofzuigen, bed opmaken, boodschappen doen' answer this question:***<br>Hoeveel weken heeft u deze thuiszorg gehad? Huishoudelijke hulp:	[ ] weken in de afgelopen 3 maanden
2.10.1.2	***If 'Wat voor hulp van de thuiszorg heeft u gehad in de afgelopen 3 maanden?' is equal to 'Verzorging van uzelf; voorbeeld: hulp bij douchen of aankleden' answer this question:***<br>Hoeveel weken heeft u deze thuiszorg gehad? Verzorging van uzelf:	[ ] weken in de afgelopen 3 maanden

2.10.1.3	***If 'Wat voor hulp van de thuiszorg heeft u gehad in de afgelopen 3 maanden?' is equal to 'Verpleging; voorbeeld: verband omdoen, medicijnen geven, bloeddruk meten' answer this question:*** Hoeveel weken heeft u deze thuiszorg gehad? Verpleging:	[ ] weken in de afgelopen 3 maanden
2.10.1.4	***If 'Wat voor hulp van de thuiszorg heeft u gehad in de afgelopen 3 maanden?' is equal to 'Huishoudelijke hulp; voorbeeld: stofzuigen, bed opmaken, boodschappen doen' answer this question:*** Hoeveel uur thuiszorg kreeg u in deze weken gemiddeld? Huishoudelijke hulp: gemiddeld	[ ] uur in de week
2.10.1.5	***If 'Wat voor hulp van de thuiszorg heeft u gehad in de afgelopen 3 maanden?' is equal to 'Verzorging van uzelf; voorbeeld: hulp bij douchen of aankleden' answer this question:*** Hoeveel uur thuiszorg kreeg u in deze weken gemiddeld? Verzorging van uzelf: gemiddeld	[ ] uur in de week
2.10.1.6	***If 'Wat voor hulp van de thuiszorg heeft u gehad in de afgelopen 3 maanden?' is equal to 'Verpleging; voorbeeld: verband omdoen, medicijnen geven, bloeddruk meten' answer this question:*** Hoeveel uur thuiszorg kreeg u in deze weken gemiddeld? Verpleging: gemiddeld	[ ] uur in de week
2.11	Heeft u in de afgelopen 3 maanden medicijnen gebruikt?	○ Ja<br>○ Nee
2.11.1	***If 'Heeft u in de afgelopen 3 maanden medicijnen gebruikt?' is equal to 'Ja' answer this question:*** Welke medicijnen heeft u in de afgelopen 3 maanden gebruikt?	[ ]
2.12	Bent u in de afgelopen 3 maanden op de spoedeisende eerste hulp van een ziekenhuis geweest? (Een andere naam voor spoedeisende eerste hulp is EHBO)	○ Nee<br>○ Ja
2.12.1	***If 'Bent u in de afgelopen 3 maanden op de spoedeisende eerste hulp van een ziekenhuis geweest? (Een andere naam voor spoedeisende eerste hulp is EHBO)' is equal to 'Ja' answer this question:*** Hoevaak bent u geweest?	[ ] keer
2.13	Bent u in de afgelopen 3 maanden met een ambulance naar het ziekenhuis gebracht? (Een andere naam voor ambulance is ziekenauto)	○ Nee<br>○ Ja

2.13.1	***If 'Bent u in de afgelopen 3 maanden met een ambulance naar het ziekenhuis gebracht? (Een andere naam voor ambulance is ziekenauto)' is equal to 'Ja' answer this question:*** Hoe vaak bent u naar het ziekenhuis gebracht?	keer
2.14	Had u in de afgelopen 3 maanden een afspraak bij de polikliniek van het ziekenhuis? (Het gaat om afspraken voor uzelf met een dokter. Bijvoorbeeld met de cardioloog, reumatoloog of neuroloog)	◯ Ja<br>◯ Nee
2.14.1	***If 'Had u in de afgelopen 3 maanden een afspraak bij de polikliniek van het ziekenhuis? (Het gaat om afspraken voor uzelf met een dokter. Bijvoorbeeld met de cardioloog, reumatoloog of neuroloog)' is equal to 'Ja' answer this question:*** Bij welke soorten dokters bent u in de afgelopen 3 maanden in het ziekenhuis geweest? En hoe vaak?	
2.15	Bent u in de afgelopen 3 maanden overdag in het ziekenhuis geweest voor een behandeling? (U bleef dus niet slapen. U kwam bijvoorbeeld voor een bloedtransfusie, nierdialyse of chemokuur)	◯ Ja<br>◯ Nee
2.15.1	***If 'Bent u in de afgelopen 3 maanden overdag in het ziekenhuis geweest voor een behandeling? (U bleef dus niet slapen. U kwam bijvoorbeeld voor een bloedtransfusie, nierdialyse of chemokuur)' is equal to 'Ja' answer this question:*** Voor welke soort behandeling was dit?	
2.15.2	***If 'Bent u in de afgelopen 3 maanden overdag in het ziekenhuis geweest voor een behandeling? (U bleef dus niet slapen. U kwam bijvoorbeeld voor een bloedtransfusie, nierdialyse of chemokuur)' is equal to 'Ja' answer this question:*** Hoeveel keer moest u in de afgelopen 3 maanden voor deze behandelingen naar het ziekenhuis?	
2.16	Bent u in de afgelopen 3 maanden ergens anders geweest voor een behandeling overdag? (U bleef dus niet slapen. U ging bijvoorbeeld naar de dagopvang van een woon-/zorgcentrum of een psychiatrische instelling. Of naar de dagbehandeling van een revalidatiecentrum)	◯ Ja<br>◯ Nee
2.16.1	***If 'Bent u in de afgelopen 3 maanden ergens anders geweest voor een behandeling overdag? (U bleef dus niet slapen. U ging bijvoorbeeld naar de dagopvang van een woon-/zorgcentrum of een psychiatrische instelling. Of naar de dagbehandeling van een revalidatiecentrum)' is equal to 'Ja' answer this question:*** Wat voor instelling was dit?	☐ Woon-/zorgcentrum<br>☐ Revalidatiecentrum<br>☐ Psychiatrische instelling<br>☐ Een andere instelling, namelijk

2.16.1.1	***If 'Wat voor instelling was dit?' is equal to 'Een andere instelling, namelijk' answer this question:***<br>Wat voor instelling was dit?	
2.16.1.2	***If 'Wat voor instelling was dit?' is equal to 'Woon-/zorgcentrum' answer this question:***<br>Hoe vaak moest u hier in de afgelopen 3 maanden naartoe? Naar het woon-/zorgcentrum:	keer in de afgelopen 3 maanden
2.16.1.3	***If 'Wat voor instelling was dit?' is equal to 'Revalidatiecentrum' answer this question:***<br>Hoe vaak moest u hier in de afgelopen 3 maanden naartoe? Naar het revalidatiecentrum:	keer in de afgelopen 3 maanden
2.16.1.4	***If 'Wat voor instelling was dit?' is equal to 'Psychiatrische instelling' answer this question:***<br>Hoe vaak moest u hier in de afgelopen 3 maanden naartoe? Naar de psychiatrische instelling:	keer in de afgelopen 3 maanden
2.16.1.5	***If 'Wat voor instelling was dit?' is equal to 'Een andere instelling, namelijk' answer this question:***<br>Hoe vaak moest u hier in de afgelopen 3 maanden naartoe? Naar de andere instelling:	keer in de afgelopen 3 maanden
2.17	Heeft u in de afgelopen 3 maanden weleens in het ziekenhuis gelegen? (U moest dus blijven slapen. Bijvoorbeeld omdat u geopereerd was en niet meteen naar huis kon)	◯ Ja<br>◯ Nee
2.17.1	***If 'Heeft u in de afgelopen 3 maanden weleens in het ziekenhuis gelegen? (U moest dus blijven slapen. Bijvoorbeeld omdat u geopereerd was en niet meteen naar huis kon)' is equal to 'Ja' answer this question:***<br>Hoe vaak heeft u in de afgelopen 3 maanden in het ziekenhuis gelegen?	keer in de afgelopen 3 maanden
2.17.2	***If 'Heeft u in de afgelopen 3 maanden weleens in het ziekenhuis gelegen? (U moest dus blijven slapen. Bijvoorbeeld omdat u geopereerd was en niet meteen naar huis kon)' is equal to 'Ja' answer this question:***<br>Hoe lang heeft u in het ziekenhuis gelegen?	dagen in de afgelopen 3 maanden
2.18	Moest u in de afgelopen 3 maanden ergens anders blijven slapen voor uw gezondheid? (Bijvoorbeeld in een woon-/zorgcentrum, psychiatrische instelling of revalidatiecentrum)	◯ Ja<br>◯ Nee

2.18.1	***If 'Moest u in de afgelopen 3 maanden ergens anders blijven slapen voor uw gezondheid? (Bijvoorbeeld in een woon-/zorgcentrum, psychiatrische instelling of revalidatiecentrum)' is equal to 'Ja' answer this question:*** Wat voor instelling was dit?	☐ Woon-/zorgcentrum<br>☐ Revalidatiecentrum<br>☐ Psychiatrische instelling<br>☐ Een andere instelling, namelijk
2.18.1.1	***If 'Wat voor instelling was dit?' is equal to 'Een andere instelling, namelijk' answer this question:*** Een andere instelling, namelijk	
2.18.1.2	***If 'Wat voor instelling was dit?' is equal to 'Woon-/zorgcentrum' answer this question:*** Hoe lang bent u in deze instelling geweest? In het woon-/zorgcentrum:	____ dagen in de afgelopen 3 maanden
2.18.1.3	***If 'Wat voor instelling was dit?' is equal to 'Revalidatiecentrum' answer this question:*** Hoe lang bent u in deze instelling geweest? In het revalidatiecentrum:	____ dagen in de afgelopen 3 maanden
2.18.1.4	***If 'Wat voor instelling was dit?' is equal to 'Psychiatrische instelling' answer this question:*** Hoe lang bent u in deze instelling geweest? In de psychiatrische instelling:	____ dagen in de afgelopen 3 maanden
2.18.1.5	***If 'Wat voor instelling was dit?' is equal to 'Een andere instelling, namelijk' answer this question:*** Hoe lang bent u in deze instelling geweest? In de andere instelling:	____ dagen in de afgelopen 3 maanden
2.19	Heeft u in de afgelopen 3 maanden hulp gekregen van een familielid of een bekende vanwege uw lichamelijke of psychische problemen?	◯ Ja<br>◯ Nee
2.19.1	***If 'Heeft u in de afgelopen 3 maanden hulp gekregen van een familielid of een bekende vanwege uw lichamelijke of psychische problemen?' is equal to 'Ja' answer this question:*** Wat voor hulp van familieleden of bekenden heeft u gehad in de afgelopen 3 maanden?	☐ Huishoudelijke hulp - voorbeeld: stofzuigen, bed opmaken, boodschappen doen, klaarmaken van eten en drinken, verzorgen van kinderen<br>☐ Verzorging van uzelf - voorbeeld: hulp bij douchen of aankleden, hulp bij het eten en drinken of het geven van medicijnen<br>☐ Praktische hulp - voorbeeld: ondersteuning bij wandelen, het maken van uitstapjes of bezoekjes aan bekenden, bezoeken aan de huisarts of het ziekenhuis, het regelen van hulp of het regelen van financiële zaken

2.19.1.1	***If 'Wat voor hulp van familieleden of bekenden heeft u gehad in de afgelopen 3 maanden?' is equal to 'Huishoudelijke hulp - voorbeeld: stofzuigen, bed opmaken, boodschappen doen, klaarmaken van eten en drinken, verzorgen van kinderen' answer this question:***<br>Hoeveel weken heeft u deze hulp gehad? Huishoudelijke hulp:	______ weken in de afgelopen 3 maanden
2.19.1.2	***If 'Wat voor hulp van familieleden of bekenden heeft u gehad in de afgelopen 3 maanden?' is equal to 'Verzorging van uzelf - voorbeeld: hulp bij douchen of aankleden, hulp bij het eten en drinken of het geven van medicijnen' answer this question:***<br>Hoeveel weken heeft u deze hulp gehad? Verzorging van uzelf:	______ weken in de afgelopen 3 maanden
2.19.1.3	***If 'Wat voor hulp van familieleden of bekenden heeft u gehad in de afgelopen 3 maanden?' is equal to 'Praktische hulp - voorbeeld: ondersteuning bij wandelen, het maken van uitstapjes of bezoekjes aan bekenden, bezoeken aan de huisarts of het ziekenhuis, het regelen van hulp of het regelen van financiële zaken' answer this question:***<br>Hoeveel uur hulp kreeg u in deze weken gemiddeld? Praktische hulp:	______ weken in de afgelopen 3 maanden
2.19.1.4	***If 'Wat voor hulp van familieleden of bekenden heeft u gehad in de afgelopen 3 maanden?' is equal to 'Huishoudelijke hulp - voorbeeld: stofzuigen, bed opmaken, boodschappen doen, klaarmaken van eten en drinken, verzorgen van kinderen' answer this question:***<br>Hoeveel uur hulp kreeg u in deze weken gemiddeld? Huishoudelijke hulp: gemiddeld	______ uur in de week
2.19.1.5	***If 'Wat voor hulp van familieleden of bekenden heeft u gehad in de afgelopen 3 maanden?' is equal to 'Verzorging van uzelf - voorbeeld: hulp bij douchen of aankleden, hulp bij het eten en drinken of het geven van medicijnen' answer this question:***<br>Hoeveel uur hulp kreeg u in deze weken gemiddeld? Verzorging van uzelf: gemiddeld	______ uur in de week
2.19.1.6	***If 'Wat voor hulp van familieleden of bekenden heeft u gehad in de afgelopen 3 maanden?' is equal to 'Praktische hulp - voorbeeld: ondersteuning bij wandelen, het maken van uitstapjes of bezoekjes aan bekenden, bezoeken aan de huisarts of het ziekenhuis, het regelen van hulp of het regelen van financiële zaken' answer this question:***<br>Hoeveel uur hulp kreeg u in deze weken gemiddeld? Praktische hulp: gemiddeld	______ uur in de week

Vragenlijst PRIMA studie 39 weken - Dank

Number	Question	Answers
3.1	Hartelijk dank voor de tijd die u heeft genomen om de vragenlijsten in te vullen. Indien u nog opmerkingen heeft kunt u deze hierin plaatsen.	

Survey 'Vragenlijst PRIMA studie 5 jaar'

Vragenlijst PRIMA studie 5 jaar - AOFAS - Pijn

Number	Question	Answers
1.1	Hoeveel pijn ervaart u aan uw voet?	☐ Geen<br>☐ Milde pijnklachten of af en toe pijn<br>☐ Matige pijnklachten, dagelijks<br>☐ Vrijwel continu erge pijn

Vragenlijst PRIMA studie 5 jaar - AOFAS resterende gedeelte

Number	Question	Answers
2.1	Hoeveel beperkingen in het dagelijks leven ervaart u?	○ Geen beperkingen, geen hulpmiddelen nodig<br>○ Geen beperkingen in algemeen dagelijkse bezigheden, wel beperkingen in recreatieve activiteiten (sport, hobbies, etc) geen ondersteuning<br>○ Beperkingen bij dagelijkse- en recreatieve activiteiten, stok<br>○ Ernstige beperkingen bij dagelijkse- en recreatieactiviteiten, walker, krukken, rolstoel, brace

2.2	Wat is de afstand die u in één keer kunt lopen?	◯ Meer dan 600 meter<br>◯ 400 - 600 meter<br>◯ 100 - 300 meter<br>◯ Minder dan 100 meter
2.3	Heeft u moeite met lopen op een bepaalde ondergrond?	◯ Geen problemen bij lopen<br>◯ Enig problemen op ongelijke ondergrond, trap, helling, ladders<br>◯ Ernstige problemen bij ongelijke ondergrond, trap, helling, ladders
2.4	Hoe zou u uw looppatroon beoordelen?	◯ Normaal of minimaal afwijkend<br>◯ Duidelijk<br>◯ Ernstig
2.5	Hoe zou u de beweeglijkheid van uw voet beoordelen ten opzicht van de andere zijde of ten opzicte van vóór het ongeval?	◯ Hetzelfde of licht beperkt t.o.v. de niet aangedane zijde of zoals voor het ongeval<br>◯ Ongeveer de helft beperkt<br>◯ Sterk verminderd, bijna geen bewegingen mogelijk
2.6	Hoe zou u de beweeglijkheid van uw enkel beoordelen ten opzichte van de andere zijde of ten opzichte van voor het ongeval?	◯ Hetzelfde of licht beperkt t.o.v. de niet aangedane zijde of zoals voor het ongeval<br>◯ Ongeveer de helft beperkt<br>◯ Sterk verminderd, bijna geen bewegingen mogelijk
2.7	Hoe zou u de stabiliteit van uw voet en enkel beoordelen	◯ Stabiel, ik verzwik mijn enkel hooguit incidenteel<br>◯ Niet stabiel, ik verzwik mijn enkel vaak
2.8	Hoe zou u de stand van uw voet beoordelen ten opzichte van de andere zijde of ten opzichte van voor het ongeval?	◯ Goed, ik kan mijn voet goed plat op de grond zetten, waarbij de stand niet afwijkend is<br>◯ Ik sta vrijwel alleen op de buitenzijde van mijn voet<br>◯ Ik kan mijn voet niet plat op de grond zetten en loop vrijwel alleen op mijn voorvoet

Vragenlijst PRIMA studie 5 jaar - VAS Pain score

Number	Question	Answers	
3.1	VAS pijnscore (Geef uw minimaal pijnscore aan tijdens dagelijkse activiteiten op een schaal van 0 tot 100, waarbij 0 geen pijn is en 100 de ergste pijn denkbaar is)	(0.00)	100 (100.00)

Vragenlijst PRIMA studie 5 jaar - AAS

Number	Question	Answers

4.1	Wat is het meest bij u van toepassing ten aanzien van uw acitviteit?	☐ American Football<br>☐ Basketbal<br>☐ Gymnastiek/turnen<br>☐ Handbal<br>☐ Rugby<br>☐ Voetbal<br>☐ Hockey<br>☐ Korfbal<br>☐ Gevechtssporten: judo, karate, kung fu, taekwando, jiu jitsu, etc.<br>☐ Oriëntatielopen<br>☐ Rhytmische gymnastiek<br>☐ Volleybal<br>☐ Boxen<br>☐ Freestyle snowboarden<br>☐ Ijshockey<br>☐ Tennis<br>☐ Worstelen<br>☐ Fitness, aerobics<br>☐ Badminton<br>☐ Baseball<br>☐ Cross-country hardlopen<br>☐ Moderne pentathlon<br>☐ Squash<br>☐ Surfen, windsurfen<br>☐ Tafel tennis<br>☐ Atletiek: spring-, werponderdelen<br>☐ Waterskieën<br>☐ Dans<br>☐ Schermen<br>☐ Zaalhockey<br>☐ Bergbeklimmen<br>☐ Langlauf<br>☐ Parachute springen<br>☐ Softball<br>☐ Speciale beroepen en werkactiviteiten; speciale beroepen: ballet, professioneel soldaat, speciale reddingswerker, stuntman, etc.<br>☐ Duiken<br>☐ Scubaduiken<br>☐ Skaten, in-line skaten<br>☐ Atletiek: looponderdelen<br>☐ Triatlon<br>☐ Gewichtsheffen, body-building

☐ Alle competitieve sporten hieronder met ‘seasonal’ conditioning
☐ Zwaar fysiek werk
☐ Alpine skiën en snowboarden
☐ Bowlen/curlen
☐ Golf
☐ Mountainbike/BMX
☐ Powerliften
☐ Zeilen
☐ Fysiek werk
☐ Wielrennen
☐ Paardrijden
☐ Motorsporten/technische sporten
☐ Roeien, kayakken
☐ Boogschieten
☐ Water polo en zwemmen
☐ Kunnen lopen op oneven grond
☐ Geen sport, echter geen limitatie in dagelijkse activiteiten
☐ Kunnen lopen op even grond, maar de dagelijkste activiteiten zijn gelimiteerd
☐ Niet kunnen lopen, fysieke handicap wegens enkelproblemen

4.1.1 ***If 'Wat is het meest bij u van toepassing ten aanzien van uw acitviteit?' is equal to 'American Football' answer this question:***
American Football

○ Professioneel
○ Competitief
○ Recreatief

4.1.2 ***If 'Wat is het meest bij u van toepassing ten aanzien van uw acitviteit?' is equal to 'Basketbal' answer this question:***
Basketbal

○ Professioneel
○ Competitief
○ Recreatief

4.1.3 ***If 'Wat is het meest bij u van toepassing ten aanzien van uw acitviteit?' is equal to 'Gymnastiek/turnen' answer this question:***
Gymnastiek/turnen

○ Professioneel
○ Competitief
○ Recreatief

4.1.4 ***If 'Wat is het meest bij u van toepassing ten aanzien van uw acitviteit?' is equal to 'Handbal' answer this question:***
Handbal

○ Professioneel
○ Competitief
○ Recreatief

4.1.5 ***If 'Wat is het meest bij u van toepassing ten aanzien van uw acitviteit?' is equal to 'Rugby' answer this question:***
Rugby

○ Professioneel
○ Competitief
○ Recreatief

4.1.6	***If 'Wat is het meest bij u van toepassing ten aanzien van uw acitviteit?' is equal to 'Voetbal' answer this question:***<br>Voetbal	○ Professioneel<br>○ Competitief<br>○ Recreatief
4.1.7	***If 'Wat is het meest bij u van toepassing ten aanzien van uw acitviteit?' is equal to 'Hockey' answer this question:***<br>Hockey	○ Professioneel<br>○ Competitief<br>○ Recreatief
4.1.8	***If 'Wat is het meest bij u van toepassing ten aanzien van uw acitviteit?' is equal to 'Korfbal' answer this question:***<br>Korfbal	○ Professioneel<br>○ Competitief<br>○ Recreatief
4.1.9	***If 'Wat is het meest bij u van toepassing ten aanzien van uw acitviteit?' is equal to 'Gevechtssporten: judo, karate, kung fu, taekwando, jiu jitsu, etc.' answer this question:***<br>Gevechtssporten: judo, karate, kung fu, taekwando, jiu jitsu, etc.	○ Professioneel<br>○ Competitief<br>○ Recreatief
4.1.10	***If 'Wat is het meest bij u van toepassing ten aanzien van uw acitviteit?' is equal to 'Oriëntatielopen' answer this question:***<br>Oriëntatielopen	○ Professioneel<br>○ Competitief<br>○ Recreatief
4.1.11	***If 'Wat is het meest bij u van toepassing ten aanzien van uw acitviteit?' is equal to 'Rhytmische gymnastiek' answer this question:***<br>Rhytmisch gymnastiek	○ Professioneel<br>○ Competitief<br>○ Recreatief
4.1.12	***If 'Wat is het meest bij u van toepassing ten aanzien van uw acitviteit?' is equal to 'Volleybal' answer this question:***<br>Volleybal	○ Professioneel<br>○ Competitief<br>○ Recreatief
4.1.13	***If 'Wat is het meest bij u van toepassing ten aanzien van uw acitviteit?' is equal to 'Boxen' answer this question:***<br>Boxen	○ Professioneel<br>○ Competitief<br>○ Recreatief
4.1.14	***If 'Wat is het meest bij u van toepassing ten aanzien van uw acitviteit?' is equal to 'Freestyle snowboarden' answer this question:***<br>Freestyle snowboarden	○ Professioneel<br>○ Competitief<br>○ Recreatief
4.1.15	***If 'Wat is het meest bij u van toepassing ten aanzien van uw acitviteit?' is equal to 'Freestyle snowboarden' answer this question:***<br>IJshockey	○ Professioneel<br>○ Competitief<br>○ Recreatief

4.1.16	***If 'Wat is het meest bij u van toepassing ten aanzien van uw acitviteit?' is equal to 'Tennis' answer this question:*** Tennis	◯ Professioneel ◯ Competitief ◯ Recreatief
4.1.17	***If 'Wat is het meest bij u van toepassing ten aanzien van uw acitviteit?' is equal to 'Worstelen' answer this question:*** Worstelen	◯ Professioneel ◯ Competitief ◯ Recreatief
4.1.18	***If 'Wat is het meest bij u van toepassing ten aanzien van uw acitviteit?' is equal to 'Fitness, aerobics' answer this question:*** Fitness, aerobics	◯ Professioneel ◯ Competitief ◯ Recreatief
4.1.19	***If 'Wat is het meest bij u van toepassing ten aanzien van uw acitviteit?' is equal to 'Badminton' answer this question:*** Badminton	◯ Professioneel ◯ Competitief ◯ Recreatief
4.1.20	***If 'Wat is het meest bij u van toepassing ten aanzien van uw acitviteit?' is equal to 'Baseball' answer this question:*** Baseball	◯ Professioneel ◯ Competitief ◯ Recreatief
4.1.21	***If 'Wat is het meest bij u van toepassing ten aanzien van uw acitviteit?' is equal to 'Cross-country hardlopen' answer this question:*** Cross-country hardlopen	◯ Professioneel ◯ Competitief ◯ Recreatief
4.1.22	***If 'Wat is het meest bij u van toepassing ten aanzien van uw acitviteit?' is equal to 'Moderne pentathlon' answer this question:*** Moderne pentathlon	◯ Professioneel ◯ Competitief ◯ Recreatief
4.1.23	***If 'Wat is het meest bij u van toepassing ten aanzien van uw acitviteit?' is equal to 'Squash' answer this question:*** Squash	◯ Professioneel ◯ Competitief ◯ Recreatief
4.1.24	***If 'Wat is het meest bij u van toepassing ten aanzien van uw acitviteit?' is equal to 'Surfen, windsurfen' answer this question:*** Surfen, windsurfen	◯ Professioneel ◯ Competitief ◯ Recreatief
4.1.25	***If 'Wat is het meest bij u van toepassing ten aanzien van uw acitviteit?' is equal to 'Tafel tennis' answer this question:*** Tafel tennis	◯ Professioneel ◯ Competitief ◯ Recreatief

4.1.26	***If 'Wat is het meest bij u van toepassing ten aanzien van uw acitviteit?' is equal to 'Atletiek: spring-, werponderdelen' answer this question:*** Atletiek: spring-, werponderdelen	◯ Professioneel<br>◯ Competitief<br>◯ Recreatief
4.1.27	***If 'Wat is het meest bij u van toepassing ten aanzien van uw acitviteit?' is equal to 'Waterskieën' answer this question:*** Waterskien	◯ Professioneel<br>◯ Competitief<br>◯ Recreatief
4.1.28	***If 'Wat is het meest bij u van toepassing ten aanzien van uw acitviteit?' is equal to 'Dans' answer this question:*** Dans	◯ Professioneel<br>◯ Competitief<br>◯ Recreatief
4.1.29	***If 'Wat is het meest bij u van toepassing ten aanzien van uw acitviteit?' is equal to 'Schermen' answer this question:*** Schermen	◯ Professioneel<br>◯ Competitief<br>◯ Recreatief
4.1.30	***If 'Wat is het meest bij u van toepassing ten aanzien van uw acitviteit?' is equal to 'Zaalhockey' answer this question:*** Zaalhockey	◯ Professioneel<br>◯ Competitief<br>◯ Recreatief
4.1.31	***If 'Wat is het meest bij u van toepassing ten aanzien van uw acitviteit?' is equal to 'Bergbeklimmen' answer this question:*** Bergbeklimmen	◯ Professioneel<br>◯ Competitief<br>◯ Recreatief
4.1.32	***If 'Wat is het meest bij u van toepassing ten aanzien van uw acitviteit?' is equal to 'Langlauf' answer this question:*** Langlauf	◯ Professioneel<br>◯ Competitief<br>◯ Recreatief
4.1.33	***If 'Wat is het meest bij u van toepassing ten aanzien van uw acitviteit?' is equal to 'Parachute springen' answer this question:*** Parachute springen	◯ Professioneel<br>◯ Competitief<br>◯ Recreatief
4.1.34	***If 'Wat is het meest bij u van toepassing ten aanzien van uw acitviteit?' is equal to 'Softball' answer this question:*** Softball	◯ Professioneel<br>◯ Competitief<br>◯ Recreatief

4.1.35	***If 'Wat is het meest bij u van toepassing ten aanzien van uw acitviteit?' is equal to 'Speciale beroepen en werkactiviteiten; speciale beroepen: ballet, professioneel soldaat, speciale reddingswerker, stuntman, etc.' answer this question:***<br>Speciale beroepen en werkactiviteiten speciale beroepen: ballet, professioneel soldaat, speciale reddingswerker, stuntman, etc.	○ ballet, professioneel soldaat, speciale reddingswerker, stuntman, etc.
4.1.36	***If 'Wat is het meest bij u van toepassing ten aanzien van uw acitviteit?' is equal to 'Duiken' answer this question:***<br>Duiken	○ Professioneel/competitief<br>○ Recreatief
4.1.37	***If 'Wat is het meest bij u van toepassing ten aanzien van uw acitviteit?' is equal to 'Scubaduiken' answer this question:***<br>Scubaduiken	○ Professioneel/competitief<br>○ Recreatief
4.1.38	***If 'Wat is het meest bij u van toepassing ten aanzien van uw acitviteit?' is equal to 'Skaten, in-line skaten' answer this question:***<br>skaten, in-linen skaten	○ Professioneel/competitief<br>○ Recreatief
4.1.39	***If 'Wat is het meest bij u van toepassing ten aanzien van uw acitviteit?' is equal to 'Atletiek: looponderdelen' answer this question:***<br>Atletiek: looponderdelen	○ Professioneel/competitief<br>○ Recreatief
4.1.40	***If 'Wat is het meest bij u van toepassing ten aanzien van uw acitviteit?' is equal to 'Triatlon' answer this question:***<br>triatlon	○ Professioneel/competitief<br>○ Recreatief
4.1.41	***If 'Wat is het meest bij u van toepassing ten aanzien van uw acitviteit?' is equal to 'Gewichtsheffen, body-building' answer this question:***<br>Gewichtsheffen, body-building	○ Professioneel/competitief<br>○ Recreatief
4.1.42	***If 'Wat is het meest bij u van toepassing ten aanzien van uw acitviteit?' is equal to 'Alle competitieve sporten hieronder met 'seasonal' conditioning' answer this question:***<br>Alle competitieve sporten hieronder met 'seasonal' conditioning	○ Alle competitieve sporten hieronder met 'seasonal' conditioning
4.1.43	***If 'Wat is het meest bij u van toepassing ten aanzien van uw acitviteit?' is equal to 'Zwaar fysiek werk' answer this question:***<br>Zwaar fysiek werk	○ Alle competitieve sporten hieronder met 'seasonal' conditioning

4.1.44	***If 'Wat is het meest bij u van toepassing ten aanzien van uw acitviteit?' is equal to 'Alpine skiën en snowboarden' answer this question:*** Alpine skien en snowboarden	◯ Alpine skien en snowboarden
4.1.45	***If 'Wat is het meest bij u van toepassing ten aanzien van uw acitviteit?' is equal to 'Bowlen/curlen' answer this question:*** Bowlen/curlen	◯ Bowlen/curlen
4.1.46	***If 'Wat is het meest bij u van toepassing ten aanzien van uw acitviteit?' is equal to 'Golf' answer this question:*** Golf	◯ Golf
4.1.47	***If 'Wat is het meest bij u van toepassing ten aanzien van uw acitviteit?' is equal to 'Mountainbike/BMX' answer this question:*** Mountainbike/BMX	◯ Mountainbike/BMX
4.1.48	***If 'Wat is het meest bij u van toepassing ten aanzien van uw acitviteit?' is equal to 'Powerliften' answer this question:*** Powerliften	◯ Powerliften
4.1.49	***If 'Wat is het meest bij u van toepassing ten aanzien van uw acitviteit?' is equal to 'Zeilen' answer this question:*** Zeilen	◯ Zeilen
4.1.50	***If 'Wat is het meest bij u van toepassing ten aanzien van uw acitviteit?' is equal to 'Fysiek werk' answer this question:*** Fysiek werk	◯ Fysiek werk
4.1.51	***If 'Wat is het meest bij u van toepassing ten aanzien van uw acitviteit?' is equal to 'Wielrennen' answer this question:*** Wielrennen	◯ Wielrennen
4.1.52	***If 'Wat is het meest bij u van toepassing ten aanzien van uw acitviteit?' is equal to 'Paardrijden' answer this question:*** Paardrijden	◯ Paardrijden
4.1.53	***If 'Wat is het meest bij u van toepassing ten aanzien van uw acitviteit?' is equal to 'Motorsporten/technische sporten' answer this question:*** Motorsporten/technische sporten	◯ Motorsporten/technische sporten
4.1.54	***If 'Wat is het meest bij u van toepassing ten aanzien van uw acitviteit?' is equal to 'Roeien, kayakken' answer this question:*** Roeien, kayakken	◯ Roeien, kayakken

4.1.55	***If 'Wat is het meest bij u van toepassing ten aanzien van uw acitviteit?' is equal to 'Boogschieten' answer this question:*** Boogschieten	○ Boogschieten
4.1.56	***If 'Wat is het meest bij u van toepassing ten aanzien van uw acitviteit?' is equal to 'Water polo en zwemmen' answer this question:*** Water polo en zwemmen	○ Water polo en zwemmen
4.1.57	***If 'Wat is het meest bij u van toepassing ten aanzien van uw acitviteit?' is equal to 'Kunnen lopen op oneven grond' answer this question:*** Kunnen lopen op oneven grond	○ Kunnen lopen op oneven grond
4.1.58	***If 'Wat is het meest bij u van toepassing ten aanzien van uw acitviteit?' is equal to 'Geen sport, echter geen limitatie in dagelijkse activiteiten' answer this question:*** Geen sport, echter geen limitatie in dagelijkse activiteiten	○ Geen sport, echter geen limitatie in dagelijkse activiteiten
4.1.59	***If 'Wat is het meest bij u van toepassing ten aanzien van uw acitviteit?' is equal to 'Kunnen lopen op even grond, maar de dagelijkste activiteiten zijn gelimiteerd' answer this question:*** Kunnen lopen op even grond, maar de dagelijkste activiteiten zijn gelimiteerd	○ Kunnen lopen op even grond, maar de dagelijkste activiteiten zijn gelimiteerd
4.1.60	***If 'Wat is het meest bij u van toepassing ten aanzien van uw acitviteit?' is equal to 'Niet kunnen lopen, fysieke handicap wegens enkelproblemen' answer this question:*** Niet kunnen lopen, fysieke handicap wegens enkelproblemen	○ Niet kunnen lopen, fysieke handicap wegens enkelproblemen

Vragenlijst PRIMA studie 5 jaar - Hoe tevreden bent u over de enkelklachten?

Number	Question	Answers
5.1	Hoe tevreden bent u over de enkelklachten?	○ Slecht<br>○ Matig<br>○ Goed<br>○ Uitstekend

Vragenlijst PRIMA studie 5 jaar - SF-36

Number	Question	Answers
	Deze vragenlijst gaat over uw standpunten t.a.v. uw gezondheid. Met behulp van deze gegevens kan worden bijgehouden hoe u zich voelt en hoe goed u in staat bent uw gebruikelijke bezigheden uit te voeren. Beantwoord elke vraag door een antwoord aan te klikken. Als u niet zeker weet hoe u een vraag moet beantwoorden, geef dan het best mogelijke antwoord.	
6.1	Hoe zou u over het algemeen uw gezondheid noemen?	◯ Uitstekend ◯ Zeer goed ◯ Goed ◯ Matig ◯ Slecht
6.2	Hoe beoordeelt u nu uw gezondheid over het algemeen vergeleken met een jaar geleden?	◯ Veel beter dan een jaar geleden ◯ Wat beter dan een jaar geleden ◯ Ongeveer hetzelfde als een jaar geleden ◯ Wat slechter dan een jaar geleden ◯ Veel slechter dan een jaar geleden
6.3	Wordt u door uw gezondheid op dit moment beperkt bij forse inspanning, zoals hardlopen, tillen van zware voorwerpen of een veeleisende sport beoefenen?	◯ Ja, ernstig beperkt ◯ Ja, een beetje beperkt ◯ Nee, helemaal niet beperkt
6.4	Wordt u door uw gezondheid op dit moment beperkt bij matige inspanning zoals een tafel verplaatsen, stofzuigen, zwemmen of fietsen?	◯ Ja, ernstig beperkt ◯ Ja, een beetje beperkt ◯ Nee, helemaal niet beperkt
6.5	Wordt u door uw gezondheid op dit moment beperkt bij boodschappen tillen of dragen?	◯ Ja, ernstig beperkt ◯ Ja, een beetje beperkt ◯ Nee, helemaal niet beperkt
6.6	Wordt u door uw gezondheid op dit moment beperkt bij een paar trappen oplopen?	◯ Ja, ernstig beperkt ◯ Ja, een beetje beperkt ◯ Nee, helemaal niet beperkt
6.7	Wordt u door uw gezondheid op dit moment beperkt bij één trap oplopen?	◯ Ja, ernstig beperkt ◯ Ja, een beetje beperkt ◯ Nee, helemaal niet beperkt
6.8	Wordt u door uw gezondheid op dit moment beperkt bij bukken, knielen of hurken?	◯ Ja, ernstig beperkt ◯ Ja, een beetje beperkt ◯ Nee, helemaal niet beperkt
6.9	Wordt u door uw gezondheid op dit moment beperkt bij meer dan één kilometer lopen?	◯ Ja, ernstig beperkt ◯ Ja, een beetje beperkt ◯ Nee, helemaal niet beperkt
6.10	Wordt u door uw gezondheid op dit moment beperkt bij een paar honderd meter lopen?	◯ Ja, ernstig beperkt ◯ Ja, een beetje beperkt ◯ Nee, helemaal niet beperkt

For peer review only - http://bmjopen.bmj.com/site/about/guidelines.xhtml

6.11	Wordt u door uw gezondheid op dit moment beperkt bij ongeveer honderd meter lopen?	◯ Ja, ernstig beperkt ◯ Ja, een beetje beperkt ◯ Nee, helemaal niet beperkt
6.12	Wordt u door uw gezondheid op dit moment beperkt bij uzelf wassen of aankleden?	◯ Ja, ernstig beperkt ◯ Ja, een beetje beperkt ◯ Nee, helemaal niet beperkt
6.13	U besteedde in de afgelopen 4 weken minder tijd aan werk of andere bezigheden	◯ Ja ◯ Nee
6.14	U heeft in de afgelopen 4 weken minder bereikt dan u zou willen	◯ Ja ◯ Nee
6.15	U was in de afgelopen 4 weken beperkt in het soort werk of andere bezigheden.	◯ Ja ◯ Nee
6.16	U had de afgelopen 4 weken moeite om uw werk of andere bezigheden uit te voeren (het kostte u bijvoorbeeld extra inspanning).	◯ Ja ◯ Nee
6.17	U besteedde in de afgelopen 4 weken minder tijd aan werk of andere bezigheden ten gevolge van emotionele problemen	◯ Ja ◯ Nee
6.18	U heeft in de afgelopen 4 weken minder bereikt dan u zou willen ten gevolge van emotionele problemen.	◯ Ja ◯ Nee
6.19	U deed de afgelopen 4 weken uw werk of andere bezigheden niet zo zorgvuldig als gewoonlijk ten gevolge van emotionele problemen.	◯ Ja ◯ Nee
6.20	In hoeverre hebben uw lichamelijke gezondheid of emotionele problemen u gedurende de afgelopen 4 weken gehinderd in uw normale omgang met familie, vrienden of buren, of bij activiteiten in groepsverband?	◯ Helemaal niet ◯ Enigszins ◯ Nogal ◯ Veel ◯ Heel erg veel
6.21	Hoeveel lichamelijke pijn heeft u de afgelopen 4 weken gehad?	◯ Geen<br>◯ Heel licht<br>◯ Licht<br>◯ Nogal<br>◯ Ernstig<br>◯ Heel ernstig
6.22	In welke mate bent u de afgelopen 4 weken door de pijn gehinderd in uw normale werk (zowel werk buitenshuis als huishoudelijk werk)?	◯ Helemaal niet<br>◯ Een klein beetje<br>◯ Nogal<br>◯ Veel<br>◯ Heel erg veel

6.23	Voelde u zich levenslustig?	◯ Altijd<br>◯ Meestal<br>◯ Vaak<br>◯ Soms<br>◯ Zelden<br>◯ Nooit
6.24	Was u erg zenuwachtig?	◯ Altijd<br>◯ Meestal<br>◯ Vaak<br>◯ Soms<br>◯ Zelden<br>◯ Nooit
6.25	Zat u zo in de put dat u niets kon?	◯ Altijd<br>◯ Meestal<br>◯ Vaak<br>◯ Soms<br>◯ Zelden<br>◯ Nooit
6.26	Voelde u zich rustig en tevreden?	◯ Altijd<br>◯ Meestal<br>◯ Vaak<br>◯ Soms<br>◯ Zelden<br>◯ Nooit
6.27	Had u veel energie?	◯ Altijd<br>◯ Meestal<br>◯ Vaak<br>◯ Soms<br>◯ Zelden<br>◯ Nooit
6.28	Voelde u zich somber en neerslachtig?	◯ Altijd<br>◯ Meestal<br>◯ Vaak<br>◯ Soms<br>◯ Zelden<br>◯ Nooit

6.29	Voelde u zich uitgeput?	◯ Altijd<br>◯ Meestal<br>◯ Vaak<br>◯ Soms<br>◯ Zelden<br>◯ Nooit
6.30	Was u een gelukkig mens?	◯ Altijd<br>◯ Meestal<br>◯ Vaak<br>◯ Soms<br>◯ Zelden<br>◯ Nooit
6.31	Voelde u zich moe?	◯ Altijd<br>◯ Meestal<br>◯ Vaak<br>◯ Soms<br>◯ Zelden<br>◯ Nooit
6.32	Hoe vaak hebben uw lichamelijke gezondheid of emotionele problemen u gedurende de afgelopen 4 weken gehinderd bij uw sociale activiteiten (zoals vrienden of familie bezoeken etc.)?	◯ Altijd<br>◯ Meestal<br>◯ Soms<br>◯ Zelden<br>◯ Nooit
6.33	Ik lijk wat gemakkelijker ziek te worden dan andere mensen	◯ Volkomen juist<br>◯ Grotendeels juist<br>◯ Weet ik niet<br>◯ Grotendeels onjuist<br>◯ Volkomen onjuist
6.34	Ik ben even gezond als andere mensen die ik ken	◯ Volkomen juist<br>◯ Grotendeels juist<br>◯ Weet ik niet<br>◯ Grotendeels onjuist<br>◯ Volkomen onjuist

6.35	Ik verwacht dat mijn gezondheid achteruit zal gaan	◯ Volkomen juist<br>◯ Grotendeels juist<br>◯ Weet ik niet<br>◯ Grotendeels onjuist<br>◯ Volkomen onjuist
6.36	Mijn gezondheid is uitstekend	◯ Volkomen juist<br>◯ Grotendeels juist<br>◯ Weet ik niet<br>◯ Grotendeels onjuist<br>◯ Volkomen onjuist

Vragenlijst PRIMA studie 5 jaar - GAS

Number	Question	Answers
	Tijdens de eerste afspraak werd een duidelijk doel met u afgesproken. Dit werd tevens per email naar u gestuurd. Hoe is de situatie nu ten aanzien van de destijds gestelde doelstelling?	
7.1	Wat is nu de situatie ten aanzien van het tijdens de eerste afspraak afgesproken doelstelling (Goal attainment Scaling)?	◯ Achteruitgang (minder dan de utigangssituatie)<br>◯ Uitgangssituatie<br>◯ Minder dan het doel<br>◯ Doel<br>◯ Meer dan het doel<br>◯ Veel meer dan het doel

Vragenlijst PRIMA studie 5 jaar - EQ-5D-3L

Number	Question	Answers
	Deze vragenlijst gaat over uw standpunten t.a.v. uw gezondheid. Met behulp van deze gegevens kan worden bijgehouden hoe u zich voelt en hoe goed u in staat bent uw gebruikelijke bezigheden uit te voeren. Beantwoord elke vraag door een antwoord aan te klikken. Als u niet zeker weet hoe u een vraag moet beantwoorden, geef dan het best mogelijke antwoord.	
8.1	Hoe is het met uw Mobiliteit gesteld?	◯ Ik heb geen problemen met lopen<br>◯ Ik heb enige problemen met lopen<br>◯ Ik ben bedlegerig

8.2	Hoe is het met uw Zelfzorg gesteld?	◯ Ik heb geen problemen om mijzelf te wassen of aan te kleden<br>◯ Ik heb enige problemen om mijzelf te wassen of aan te kleden<br>◯ Ik ben niet in staat om mijzelf te wassen of aan te kleden
8.3	Hoe is het met de Dagelijkse activiteiten (werk, studie, huishouden, gezins- en vrijetijdsactiviteiten) gesteld?	◯ Ik heb geen problemen met mijn dagelijkse activiteiten<br>◯ Ik heb enige problemen met mijn dagelijkse activiteiten<br>◯ Ik ben niet in staat om mijn dagelijkse activiteiten uit te voeren
8.4	Hoe is het met de Pijn/klachten gesteld?	◯ Ik heb geen pijn of andere klachten<br>◯ Ik heb matige pijn of andere klachten<br>◯ Ik heb zeer ernstige pijn of andere klachten
8.5	Hoe is het met de Stemming gesteld?	◯ Ik ben niet angstig of somber<br>◯ Ik ben matig angstig of somber<br>◯ Ik ben erg angstig of somber
	We willen weten hoe goed of slecht uw gezondheid VANDAAG is. Deze meetschaal loopt van 0 tot 100: 100 staat voor de BESTE gezondheid die u zich kunt voorstellen - 0 staat voor de SLECHTSTE gezondheid die u zich kunt voorstellen	
8.6	Uw Gezondheid vandaag	(0.00) (100.00)

Vragenlijst PRIMA studie 5 jaar - AOS

Number	Question	Answers
	Instructies: De lijn naast elke vraag staat voor hoeveel PIJN u heeft in verschillende situaties. De linker kant (0) is “geen pijn” en de rechter kant (100) is “ergste pijn denkbaar”. Geef voor de onderstaande situaties op de lijn aan hoeveel PIJN u in de afgelopen week in de enkel had. Als een of meerdere van deze situaties niet van toepassing waren, dan kiest u de "niet van toepassing" optie.	
9.1	Wat was de hoogte van de ergste pijn in de afgelopen week?	(0.00) (100.00)
9.2	Heeft u pijn als u 's ochtends opstaat?	◯ Ja<br>◯ Nee<br>◯ Niet van toepassing

9.2.1	***If 'Heeft u pijn als u 's ochtends opstaat?' is equal to 'Ja' answer this question:***<br>Hoeveel pijn heeft u voordat u 's ochtends opstaat?	(0.00)	(100.00)
9.3	Heeft u pijn wanneer u op blote voeten loopt?	◯ Ja<br>◯ Nee<br>◯ Niet van toepassing	
9.3.1	***If 'Heeft u pijn wanneer u op blote voeten loopt?' is equal to 'Ja' answer this question:***<br>Hoeveel pijn heeft u als u op blote voeten loopt?	(0.00)	(100.00)
9.4	Heeft u pijn wanneer u op blote voeten staat?	◯ Ja<br>◯ Nee<br>◯ Niet van toepassing	
9.4.1	***If 'Heeft u pijn wanneer u op blote voeten staat?' is equal to 'Ja' answer this question:***<br>Hoeveel pijn heeft u als u op blote voeten staat?	(0.00)	(100.00)
9.5	Heeft u pijn wanneer u met schoenen loopt?	◯ Ja<br>◯ Nee<br>◯ Niet van toepassing	
9.5.1	***If 'Heeft u pijn wanneer u met schoenen loopt?' is equal to 'Ja' answer this question:***<br>Hoeveel pijn heeft u wanneer u met schoenen loopt?	(0.00)	(100.00)
9.6	Heeft u pijn wanneer u met schoenen staat?	◯ Ja<br>◯ Nee<br>◯ Niet van toepassing	
9.6.1	***If 'Heeft u pijn wanneer u met schoenen staat?' is equal to 'Ja' answer this question:***<br>Hoeveel pijn heeft u wanneer u met schoenen staat?	(0.00)	(100.00)
9.7	Heeft u pijn wanneer u loopt met steunzolen of een brace?	◯ Ja<br>◯ Nee<br>◯ Niet van toepassing	
9.7.1	***If 'Heeft u pijn wanneer u loopt met steunzolen of een brace?' is equal to 'Ja' answer this question:***<br>Hoeveel pijn heeft u wanneer u loopt met steunzolen of een brace?	(0.00)	(100.00)

9.8	Heeft u pijn wanneer u staat met steunzolen of een brace?	◯ Ja<br>◯ Nee<br>◯ Niet van toepassing	
9.8.1	***If 'Heeft u pijn wanneer u staat met steunzolen of een brace?' is equal to 'Ja' answer this question:***<br>Hoeveel pijn heeft u wanneer u staat met steunzolen of een brace?	(0.00)	(100.00)
9.9	Heeft u pijn aan het einde van de dag?	◯ Ja<br>◯ Nee<br>◯ Niet van toepassing	
9.9.1	***If 'Heeft u pijn aan het einde van de dag?' is equal to 'Ja' answer this question:***<br>Hoeveel pijn heeft u aan het einde van de dag?	(0.00)	(100.00)
	Instructies:De lijn naast elke vraag staat voor hoeveel MOEITE u heeft met verschillende activiteiten. De linker kant (0) is “Niet moeilijk” en de rechter kant (100) is “Te moeilijk, niet uitvoerbaar”. Geef voor de onderstaande activiteiten op de lijn aan hoeveel MOEITE u in de afgelopen week door enkelklachten had met onderstaande activiteiten. Als een situatie niet van toepassing is, kies dan de optie "niet van toepassing".		
9.10	Heeft u moeite met door het huis lopen?	◯ Ja<br>◯ Nee<br>◯ Niet van toepassing	
9.10.1	***If 'Heeft u moeite met door het huis lopen?' is equal to 'Ja' answer this question:***<br>Hoeveel moeite heeft u met door het huis lopen?	(0.00)	(100.00)
9.11	Heeft u moeite met buiten lopen op oneven ondergrond?	◯ Ja<br>◯ Nee<br>◯ Niet van toepassing	
9.11.1	***If 'Heeft u moeite met buiten lopen op oneven ondergrond?' is equal to 'Ja' answer this question:***<br>Hoeveel moeite heeft u met buiten lopen op oneven ondergrond?	(0.00)	(100.00)
9.12	Heeft u moeite met een paar honderd meter lopen?	◯ Ja<br>◯ Nee<br>◯ Niet van toepassing	
9.12.1	***If 'Heeft u moeite met een paar honderd meter lopen?' is equal to 'Ja' answer this question:***<br>Hoeveel moeite heeft u met een paar honderd meter lopen?	(0.00)	(100.00)

9.13	Heeft u moeite met een trap oplopen?	◯ Ja<br>◯ Nee<br>◯ Niet van toepassing	
9.13.1	***If 'Heeft u moeite met een trap oplopen?' is equal to 'Ja' answer this question:***<br>Hoeveel moeite heeft u met een trap oplopen?	(0.00)	(100.00)
9.14	Heeft u moeite met een trap aflopen?	◯ Ja<br>◯ Nee<br>◯ Niet van toepassing	
9.14.1	***If 'Heeft u moeite met een trap aflopen?' is equal to 'Ja' answer this question:***<br>Hoeveel moeite heeft u met een trap aflopen?	(0.00)	(100.00)
9.15	Heeft u moeite met op de tenen staan?	◯ Ja<br>◯ Nee<br>◯ Niet van toepassing	
9.15.1	***If 'Heeft u moeite met op de tenen staan?' is equal to 'Ja' answer this question:***<br>Hoeveel moeite heeft u met op de tenen staan?	(0.00)	(100.00)
9.16	Heeft u moeite met opstaan uit de stoel?	◯ Ja<br>◯ Nee<br>◯ Niet van toepassing	
9.16.1	***If 'Heeft u moeite met opstaan uit de stoel?' is equal to 'Ja' answer this question:***<br>Hoeveel moeite heeft u met opstaan uit de stoel?	(0.00)	(100.00)
9.17	Heeft u moeite met het op- of afstappen van stoepranden?	◯ Ja<br>◯ Nee<br>◯ Niet van toepassing	
9.17.1	***If 'Heeft u moeite met het op- of afstappen van stoepranden?' is equal to 'Ja' answer this question:***<br>Hoeveel moeite heeft u met het op- of afstappen van stoepranden?	(0.00)	(100.00)
9.18	Heeft u moeite met snel lopen of rennen?	◯ Ja<br>◯ Nee<br>◯ Niet van toepassing	

9.18.1	***If 'Heeft u moeite met snel lopen of rennen?' is equal to 'Ja' answer this question:***<br>Hoeveel moeite heeft u met snel lopen of rennen?	(0.00)	(100.00)

Vragenlijst PRIMA studie 5 jaar - FAOS

Number	Question	Answers
	Deze lijst vraagt naar uw mening over uw voet/enkel. Uw antwoorden geven ons een beeld van uw voet/enkel klachten en hoe u in staat bent om alledaagse activiteiten uit te voeren in uw huidige situatie. Beantwoorden van een vraag doet u door het aanklikken van een vakje met het volgens u meest juiste antwoord (één vakje per vraag). Als u niet zeker weet hoe u een vraag moet beantwoorden, geeft u dan het antwoord dat volgens u het meest op uw situatie van toepassing is. Deze vraag heeft betrekking op het voorkomen van voet/enkel klachten in de afgelopen week.	
10.1	Is uw voet/enkel gezwollen?	◯ Nooit ◯ Zelden ◯ Soms ◯ Vaak ◯ Altijd
10.2	Voelt u gekraak of hoort u klikken of een ander vreemd geluid wanneer u de voet/enkel beweegt?	◯ Nooit ◯ Zelden ◯ Soms ◯ Vaak ◯ Altijd
10.3	Hapert uw enkel of blokkeert uw enkel ('op slot' gaan zitten) wanneer u deze beweegt?	◯ Nooit ◯ Zelden ◯ Soms ◯ Vaak ◯ Altijd
10.4	Kunt u de voet/enkel volledig strekken?	◯ Altijd ◯ Vaak ◯ Soms ◯ Zelden ◯ Nooit
10.5	Kunt u de voet volledig naar u toe buigen?	◯ Altijd ◯ Vaak ◯ Soms ◯ Zelden ◯ Nooit
10.6	In welke mate heeft u een stijf gevoel in de voet/enkel 's ochtends bij het wakker worden?	◯ Niet ◯ Mild ◯ Matig ◯ Ernstig ◯ Zeer ernstig
10.7	In welke mate heeft u een stijf gevoel in de voet/enkel na zitten, liggen of rusten later op de dag?	◯ Niet ◯ Mild ◯ Matig ◯ Ernstig ◯ Zeer ernstig
10.8	Hoe vaak heeft u pijn in uw voet/enkel?	◯ Nooit ◯ Maandelijks ◯ Wekelijks ◯ Dagelijks ◯ Altijd

10.9	Hoeveel voet/enkel pijn heeft u gehad in de afgelopen week bij draaien als uw voet/enkel op de grond staat?	◯ Geen<br>◯ Mild<br>◯ Matig<br>◯ Ernstig<br>◯ Zeer ernstig
10.10	Hoeveel voet/enkel pijn heeft u gehad in de afgelopen week bij het volledig uitstrekken van de voet/enkel?	◯ Geen<br>◯ Mild<br>◯ Matig<br>◯ Ernstig<br>◯ Zeer ernstig
10.11	Hoeveel voet/enkel pijn heeft u gehad in de afgelopen week bij het volledig naar u toe buigen/optrekken van de voet/enkel	◯ Geen<br>◯ Mild<br>◯ Matig<br>◯ Ernstig<br>◯ Zeer ernstig
10.12	Hoeveel voet/enkel pijn heeft u gehad in de afgelopen week bij het lopen op een vlakke ondergrond	◯ Geen<br>◯ Mild<br>◯ Matig<br>◯ Ernstig<br>◯ Zeer ernstig
10.13	Hoeveel voet/enkel pijn heeft u gehad in de afgelopen week bij het trap op- en trap af lopen?	◯ Geen<br>◯ Mild<br>◯ Matig<br>◯ Ernstig<br>◯ Zeer ernstig
10.14	Hoeveel voet/enkel pijn heeft u gehad in de afgelopen week 's nachts in bed?	◯ Geen<br>◯ Mild<br>◯ Matig<br>◯ Ernstig<br>◯ Zeer ernstig
10.15	Hoeveel voet/enkel pijn heeft u gehad in de afgelopen week bij het zitten of liggen?	◯ Geen<br>◯ Mild<br>◯ Matig<br>◯ Ernstig<br>◯ Zeer ernstig

10.16	Hoeveel voet/enkel pijn heeft u gehad in de afgelopen week bij het rechtop staan?	◯ Geen<br>◯ Mild<br>◯ Matig<br>◯ Ernstig<br>◯ Zeer ernstig
10.17	In welke mate werd u gehinderd bij het trap aflopen?	◯ Niet ◯ Mild ◯ Matig ◯ Ernstig ◯ Zeer ernstig
10.18	In welke mate werd u gehinderd bij het trap op lopen?	◯ Niet ◯ Mild ◯ Matig ◯ Ernstig ◯ Zeer ernstig
10.19	In welke mate werd u gehinderd als u vanuit een zittende positie ging staan?	◯ Niet ◯ Mild ◯ Matig ◯ Ernstig ◯ Zeer ernstig
10.20	In welke mate werd u gehinderd bij het staan?	◯ Niet ◯ Mild ◯ Matig ◯ Ernstig ◯ Zeer ernstig
10.21	In welke mate werd u gehinderd bij het naar de grond buigen/iets oprapen?	◯ Niet ◯ Mild ◯ Matig ◯ Ernstig ◯ Zeer ernstig
10.22	In welke mate werd u gehinderd bij het lopen op een vlakke ondergrond?	◯ Niet ◯ Mild ◯ Matig ◯ Ernstig ◯ Zeer ernstig
10.23	In welke mate werd u gehinderd bij het in- en uit de auto stappen?	◯ Niet ◯ Mild ◯ Matig ◯ Ernstig ◯ Zeer ernstig
10.24	In welke mate werd u gehinderd bij het boodschappen doen?	◯ Niet ◯ Mild ◯ Matig ◯ Ernstig ◯ Zeer ernstig
10.25	In welke mate werd u gehinderd bij sokken/panty's aantrekken?	◯ Niet ◯ Mild ◯ Matig ◯ Ernstig ◯ Zeer ernstig
10.26	In welke mate werd u gehinderd bij het opstaan uit bed?	◯ Niet ◯ Mild ◯ Matig ◯ Ernstig ◯ Zeer ernstig
10.27	In welke mate werd u gehinderd bij het sokken uittrekken?	◯ Niet ◯ Mild ◯ Matig ◯ Ernstig ◯ Zeer ernstig
10.28	In welke mate werd u gehinderd bij het in bed liggen (omdraaien, lange tijd uw voet/enkel in dezelfde positie houden)?	◯ Niet ◯ Mild ◯ Matig ◯ Ernstig ◯ Zeer ernstig

10.29	In welke mate werd u gehinderd bij het in/uit bad stappen?	◯ Niet ◯ Mild ◯ Matig ◯ Ernstig ◯ Zeer ernstig
10.30	In welke mate werd u gehinderd bij zitten?	◯ Niet ◯ Mild ◯ Matig ◯ Ernstig ◯ Zeer ernstig
10.31	In welke mate werd u gehinderd bij het toilet op en af gaan?	◯ Niet ◯ Mild ◯ Matig ◯ Ernstig ◯ Zeer ernstig
10.32	In welke mate werd u gehinderd bij zwaar huishoudelijk werk (bijvoorbeeld zware dozen sjouwen, vloer schrobben)?	◯ Niet ◯ Mild ◯ Matig ◯ Ernstig ◯ Zeer ernstig
10.33	In welke mate werd u gehinderd bij licht huishoudeijk werk (bijvoorbeeld koken, afstoffen)?	◯ Niet ◯ Mild ◯ Matig ◯ Ernstig ◯ Zeer ernstig
10.34	In welke mate werd u gehinderd bij hurken?	◯ Niet ◯ Mild ◯ Matig ◯ Ernstig ◯ Zeer ernstig
10.35	In welke mate werd u gehinderd bij hardlopen?	◯ Niet ◯ Mild ◯ Matig ◯ Ernstig ◯ Zeer ernstig
10.36	In welke mate werd u gehinderd bij springen?	◯ Niet ◯ Mild ◯ Matig ◯ Ernstig ◯ Zeer ernstig
10.37	In welke mate werd u gehinderd bij ronddraaien op uw aangedane voet/enkel?	◯ Niet ◯ Mild ◯ Matig ◯ Ernstig ◯ Zeer ernstig
10.38	In welke mate werd u gehinderd bij knielen?	◯ Niet ◯ Mild ◯ Matig ◯ Ernstig ◯ Zeer ernstig
10.39	Hoe vaak bent u zich bewust van uw voet/enkel probleem?	◯ Nooit ◯ Maandelijks ◯ Wekelijks ◯ Dagelijks ◯ Altijd
10.40	Heeft u uw leven veranderd om activiteiten te vermijden die schadelijk kunnen zijn voor uw voet/enkel?	◯ Niet<br>◯ Enigszins<br>◯ Matig<br>◯ Behoorlijk<br>◯ Volledig

10.41	In hoeverre kunt u op uw voet/enkel vertrouwen?	◯ Volledig<br>◯ Behoorlijk<br>◯ Matig<br>◯ Enigzins<br>◯ Niet
10.42	In het algemeen, in welke mate ondervindt u hinder van uw voet/enkel	◯ Geen<br>◯ Mild<br>◯ Matig<br>◯ Ernstig<br>◯ Zeer ernstig

Vragenlijst PRIMA studie 5 jaar - Dank

Number	Question	Answers
11.1	Hartelijk dank voor de tijd die u heeft genomen om de vragenlijsten in te vullen. Indien u nog opmerkingen heeft kunt u deze hierin plaatsen.	

Survey 'Vragenlijst PRIMA studie 52 weken'

Vragenlijst PRIMA studie 52 weken - AOFAS - Pijn

Number	Question	Answers
1.1	Hoeveel pijn ervaart u aan uw voet?	☐ Geen<br>☐ Milde pijnklachten of af en toe pijn<br>☐ Matige pijnklachten, dagelijks<br>☐ Vrijwel continu erge pijn

Vragenlijst PRIMA studie 52 weken - AOFAS resterende gedeelte

Number	Question	Answers
2.1	Hoeveel beperkingen in het dagelijks leven ervaart u?	◯ Geen beperkingen, geen hulpmiddelen nodig<br>◯ Geen beperkingen in algemeen dagelijkse bezigheden, wel beperkingen in recreatieve activiteiten (sport, hobbies, etc) geen ondersteuning<br>◯ Beperkingen bij dagelijkse- en recreatieve activiteiten, stok<br>◯ Ernstige beperkingen bij dagelijkse- en recreatieactiviteiten, walker, krukken, rolstoel, brace
2.2	Wat is de afstand die u in één keer kunt lopen?	◯ Meer dan 600 meter<br>◯ 400 - 600 meter<br>◯ 100 - 300 meter<br>◯ Minder dan 100 meter
2.3	Heeft u moeite met lopen op een bepaalde ondergrond?	◯ Geen problemen bij lopen<br>◯ Enig problemen op ongelijke ondergrond, trap, helling, ladders<br>◯ Ernstige problemen bij ongelijke ondergrond, trap, helling, ladders
2.4	Hoe zou u uw looppatroon beoordelen?	◯ Normaal of minimaal afwijkend<br>◯ Duidelijk<br>◯ Ernstig
2.5	Hoe zou u de beweeglijkheid van uw voet beoordelen ten opzicht van de andere zijde of ten opzicte van vóór het ongeval?	◯ Hetzelfde of licht beperkt t.o.v. de niet aangedane zijde of zoals voor het ongeval<br>◯ Ongeveer de helft beperkt<br>◯ Sterk verminderd, bijna geen bewegingen mogelijk
2.6	Hoe zou u de beweeglijkheid van uw enkel beoordelen ten opzichte van de andere zijde of ten opzichte van voor het ongeval?	◯ Hetzelfde of licht beperkt t.o.v. de niet aangedane zijde of zoals voor het ongeval<br>◯ Ongeveer de helft beperkt<br>◯ Sterk verminderd, bijna geen bewegingen mogelijk
2.7	Hoe zou u de stabiliteit van uw voet en enkel beoordelen	◯ Stabiel, ik verzwik mijn enkel hooguit incidenteel<br>◯ Niet stabiel, ik verzwik mijn enkel vaak

2.8	Hoe zou u de stand van uw voet beoordelen ten opzichte van de andere zijde of ten opzichte van voor het ongeval?	◯ Goed, ik kan mijn voet goed plat op de grond zetten, waarbij de stand niet afwijkend is<br>◯ Ik sta vrijwel alleen op de buitenzijde van mijn voet<br>◯ Ik kan mijn voet niet plat op de grond zetten en loop vrijwel alleen op mijn voorvoet

Vragenlijst PRIMA studie 52 weken - VAS Pain score

Number	Question	Answers	
3.1	VAS pijnscore (Geef uw minimaal pijnscore aan tijdens dagelijkse activiteiten op een schaal van 0 tot 100, waarbij 0 geen pijn is en 100 de ergste pijn denkbaar is)	(0.00)	100 (100.00)

Vragenlijst PRIMA studie 52 weken - AAS

Number	Question	Answers

4.1	Wat is het meest bij u van toepassing ten aanzien van uw acitviteit?	☐ American Football<br>☐ Basketbal<br>☐ Gymnastiek/turnen<br>☐ Handbal<br>☐ Rugby<br>☐ Voetbal<br>☐ Hockey<br>☐ Korfbal<br>☐ Gevechtssporten: judo, karate, kung fu, taekwando, jiu jitsu, etc.<br>☐ Oriëntatielopen<br>☐ Rhytmische gymnastiek<br>☐ Volleybal<br>☐ Boxen<br>☐ Freestyle snowboarden<br>☐ Ijshockey<br>☐ Tennis<br>☐ Worstelen<br>☐ Fitness, aerobics<br>☐ Badminton<br>☐ Baseball<br>☐ Cross-country hardlopen<br>☐ Moderne pentathlon<br>☐ Squash<br>☐ Surfen, windsurfen<br>☐ Tafel tennis<br>☐ Atletiek: spring-, werponderdelen<br>☐ Waterskieën<br>☐ Dans<br>☐ Schermen<br>☐ Zaalhockey<br>☐ Bergbeklimmen<br>☐ Langlauf<br>☐ Parachute springen<br>☐ Softball<br>☐ Speciale beroepen en werkactiviteiten; speciale beroepen: ballet, professioneel soldaat, speciale reddingswerker, stuntman, etc.<br>☐ Duiken<br>☐ Scubaduiken<br>☐ Skaten, in-line skaten<br>☐ Atletiek: looponderdelen<br>☐ Triatlon<br>☐ Gewichtsheffen, body-building

		☐ Alle competitieve sporten hieronder met 'seasonal' conditioning<br>☐ Zwaar fysiek werk<br>☐ Alpine skiën en snowboarden<br>☐ Bowlen/curlen<br>☐ Golf<br>☐ Mountainbike/BMX<br>☐ Powerliften<br>☐ Zeilen<br>☐ Fysiek werk<br>☐ Wielrennen<br>☐ Paardrijden<br>☐ Motorsporten/technische sporten<br>☐ Roeien, kayakken<br>☐ Boogschieten<br>☐ Water polo en zwemmen<br>☐ Kunnen lopen op oneven grond<br>☐ Geen sport, echter geen limitatie in dagelijkse activiteiten<br>☐ Kunnen lopen op even grond, maar de dagelijkste activiteiten zijn gelimiteerd<br>☐ Niet kunnen lopen, fysieke handicap wegens enkelproblemen
4.1.1	***If 'Wat is het meest bij u van toepassing ten aanzien van uw acitviteit?' is equal to 'American Football' answer this question:***<br>American Football	○ Professioneel<br>○ Competitief<br>○ Recreatief
4.1.2	***If 'Wat is het meest bij u van toepassing ten aanzien van uw acitviteit?' is equal to 'Basketbal' answer this question:***<br>Basketbal	○ Professioneel<br>○ Competitief<br>○ Recreatief
4.1.3	***If 'Wat is het meest bij u van toepassing ten aanzien van uw acitviteit?' is equal to 'Gymnastiek/turnen' answer this question:***<br>Gymnastiek/turnen	○ Professioneel<br>○ Competitief<br>○ Recreatief
4.1.4	***If 'Wat is het meest bij u van toepassing ten aanzien van uw acitviteit?' is equal to 'Handbal' answer this question:***<br>Handbal	○ Professioneel<br>○ Competitief<br>○ Recreatief
4.1.5	***If 'Wat is het meest bij u van toepassing ten aanzien van uw acitviteit?' is equal to 'Rugby' answer this question:***<br>Rugby	○ Professioneel<br>○ Competitief<br>○ Recreatief

4.1.6	***If 'Wat is het meest bij u van toepassing ten aanzien van uw acitviteit?' is equal to 'Voetbal' answer this question:***<br>Voetbal	◯ Professioneel<br>◯ Competitief<br>◯ Recreatief
4.1.7	***If 'Wat is het meest bij u van toepassing ten aanzien van uw acitviteit?' is equal to 'Hockey' answer this question:***<br>Hockey	◯ Professioneel<br>◯ Competitief<br>◯ Recreatief
4.1.8	***If 'Wat is het meest bij u van toepassing ten aanzien van uw acitviteit?' is equal to 'Korfbal' answer this question:***<br>Korfbal	◯ Professioneel<br>◯ Competitief<br>◯ Recreatief
4.1.9	***If 'Wat is het meest bij u van toepassing ten aanzien van uw acitviteit?' is equal to 'Gevechtssporten: judo, karate, kung fu, taekwando, jiu jitsu, etc.' answer this question:***<br>Gevechtssporten: judo, karate, kung fu, taekwando, jiu jitsu, etc.	◯ Professioneel<br>◯ Competitief<br>◯ Recreatief
4.1.10	***If 'Wat is het meest bij u van toepassing ten aanzien van uw acitviteit?' is equal to 'Oriëntatielopen' answer this question:***<br>Oriëntatielopen	◯ Professioneel<br>◯ Competitief<br>◯ Recreatief
4.1.11	***If 'Wat is het meest bij u van toepassing ten aanzien van uw acitviteit?' is equal to 'Rhytmische gymnastiek' answer this question:***<br>Rhytmisch gymnastiek	◯ Professioneel<br>◯ Competitief<br>◯ Recreatief
4.1.12	***If 'Wat is het meest bij u van toepassing ten aanzien van uw acitviteit?' is equal to 'Volleybal' answer this question:***<br>Volleybal	◯ Professioneel<br>◯ Competitief<br>◯ Recreatief
4.1.13	***If 'Wat is het meest bij u van toepassing ten aanzien van uw acitviteit?' is equal to 'Boxen' answer this question:***<br>Boxen	◯ Professioneel<br>◯ Competitief<br>◯ Recreatief
4.1.14	***If 'Wat is het meest bij u van toepassing ten aanzien van uw acitviteit?' is equal to 'Freestyle snowboarden' answer this question:***<br>Freestyle snowboarden	◯ Professioneel<br>◯ Competitief<br>◯ Recreatief
4.1.15	***If 'Wat is het meest bij u van toepassing ten aanzien van uw acitviteit?' is equal to 'Freestyle snowboarden' answer this question:***<br>IJshockey	◯ Professioneel<br>◯ Competitief<br>◯ Recreatief

4.1.16	***If 'Wat is het meest bij u van toepassing ten aanzien van uw acitviteit?' is equal to 'Tennis' answer this question:***<br>Tennis	○ Professioneel<br>○ Competitief<br>○ Recreatief
4.1.17	***If 'Wat is het meest bij u van toepassing ten aanzien van uw acitviteit?' is equal to 'Worstelen' answer this question:***<br>Worstelen	○ Professioneel<br>○ Competitief<br>○ Recreatief
4.1.18	***If 'Wat is het meest bij u van toepassing ten aanzien van uw acitviteit?' is equal to 'Fitness, aerobics' answer this question:***<br>Fitness, aerobics	○ Professioneel<br>○ Competitief<br>○ Recreatief
4.1.19	***If 'Wat is het meest bij u van toepassing ten aanzien van uw acitviteit?' is equal to 'Badminton' answer this question:***<br>Badminton	○ Professioneel<br>○ Competitief<br>○ Recreatief
4.1.20	***If 'Wat is het meest bij u van toepassing ten aanzien van uw acitviteit?' is equal to 'Baseball' answer this question:***<br>Baseball	○ Professioneel<br>○ Competitief<br>○ Recreatief
4.1.21	***If 'Wat is het meest bij u van toepassing ten aanzien van uw acitviteit?' is equal to 'Cross-country hardlopen' answer this question:***<br>Cross-country hardlopen	○ Professioneel<br>○ Competitief<br>○ Recreatief
4.1.22	***If 'Wat is het meest bij u van toepassing ten aanzien van uw acitviteit?' is equal to 'Moderne pentathlon' answer this question:***<br>Moderne pentathlon	○ Professioneel<br>○ Competitief<br>○ Recreatief
4.1.23	***If 'Wat is het meest bij u van toepassing ten aanzien van uw acitviteit?' is equal to 'Squash' answer this question:***<br>Squash	○ Professioneel<br>○ Competitief<br>○ Recreatief
4.1.24	***If 'Wat is het meest bij u van toepassing ten aanzien van uw acitviteit?' is equal to 'Surfen, windsurfen' answer this question:***<br>Surfen, windsurfen	○ Professioneel<br>○ Competitief<br>○ Recreatief
4.1.25	***If 'Wat is het meest bij u van toepassing ten aanzien van uw acitviteit?' is equal to 'Tafel tennis' answer this question:***<br>Tafel tennis	○ Professioneel<br>○ Competitief<br>○ Recreatief

4.1.26	***If 'Wat is het meest bij u van toepassing ten aanzien van uw acitviteit?' is equal to 'Atletiek: spring-, werponderdelen' answer this question:*** Atletiek: spring-, werponderdelen	◯ Professioneel ◯ Competitief ◯ Recreatief
4.1.27	***If 'Wat is het meest bij u van toepassing ten aanzien van uw acitviteit?' is equal to 'Waterskieën' answer this question:*** Waterskien	◯ Professioneel ◯ Competitief ◯ Recreatief
4.1.28	***If 'Wat is het meest bij u van toepassing ten aanzien van uw acitviteit?' is equal to 'Dans' answer this question:*** Dans	◯ Professioneel ◯ Competitief ◯ Recreatief
4.1.29	***If 'Wat is het meest bij u van toepassing ten aanzien van uw acitviteit?' is equal to 'Schermen' answer this question:*** Schermen	◯ Professioneel ◯ Competitief ◯ Recreatief
4.1.30	***If 'Wat is het meest bij u van toepassing ten aanzien van uw acitviteit?' is equal to 'Zaalhockey' answer this question:*** Zaalhockey	◯ Professioneel ◯ Competitief ◯ Recreatief
4.1.31	***If 'Wat is het meest bij u van toepassing ten aanzien van uw acitviteit?' is equal to 'Bergbeklimmen' answer this question:*** Bergbeklimmen	◯ Professioneel ◯ Competitief ◯ Recreatief
4.1.32	***If 'Wat is het meest bij u van toepassing ten aanzien van uw acitviteit?' is equal to 'Langlauf' answer this question:*** Langlauf	◯ Professioneel ◯ Competitief ◯ Recreatief
4.1.33	***If 'Wat is het meest bij u van toepassing ten aanzien van uw acitviteit?' is equal to 'Parachute springen' answer this question:*** Parachute springen	◯ Professioneel ◯ Competitief ◯ Recreatief
4.1.34	***If 'Wat is het meest bij u van toepassing ten aanzien van uw acitviteit?' is equal to 'Softball' answer this question:*** Softball	◯ Professioneel ◯ Competitief ◯ Recreatief

4.1.35	***If 'Wat is het meest bij u van toepassing ten aanzien van uw acitviteit?' is equal to 'Speciale beroepen en werkactiviteiten; speciale beroepen: ballet, professioneel soldaat, speciale reddingswerker, stuntman, etc.' answer this question:***<br>Speciale beroepen en werkactiviteiten speciale beroepen: ballet, professioneel soldaat, speciale reddingswerker, stuntman, etc.	○ ballet, professioneel soldaat, speciale reddingswerker, stuntman, etc.
4.1.36	***If 'Wat is het meest bij u van toepassing ten aanzien van uw acitviteit?' is equal to 'Duiken' answer this question:***<br>Duiken	○ Professioneel/competitief<br>○ Recreatief
4.1.37	***If 'Wat is het meest bij u van toepassing ten aanzien van uw acitviteit?' is equal to 'Scubaduiken' answer this question:***<br>Scubaduiken	○ Professioneel/competitief<br>○ Recreatief
4.1.38	***If 'Wat is het meest bij u van toepassing ten aanzien van uw acitviteit?' is equal to 'Skaten, in-line skaten' answer this question:***<br>skaten, in-linen skaten	○ Professioneel/competitief<br>○ Recreatief
4.1.39	***If 'Wat is het meest bij u van toepassing ten aanzien van uw acitviteit?' is equal to 'Atletiek: looponderdelen' answer this question:***<br>Atletiek: looponderdelen	○ Professioneel/competitief<br>○ Recreatief
4.1.40	***If 'Wat is het meest bij u van toepassing ten aanzien van uw acitviteit?' is equal to 'Triatlon' answer this question:***<br>triatlon	○ Professioneel/competitief<br>○ Recreatief
4.1.41	***If 'Wat is het meest bij u van toepassing ten aanzien van uw acitviteit?' is equal to 'Gewichtsheffen, body-building' answer this question:***<br>Gewichtsheffen, body-building	○ Professioneel/competitief<br>○ Recreatief
4.1.42	***If 'Wat is het meest bij u van toepassing ten aanzien van uw acitviteit?' is equal to 'Alle competitieve sporten hieronder met ‘seasonal’ conditioning' answer this question:***<br>Alle competitieve sporten hieronder met ‘seasonal’ conditioning	○ Alle competitieve sporten hieronder met ‘seasonal’ conditioning
4.1.43	***If 'Wat is het meest bij u van toepassing ten aanzien van uw acitviteit?' is equal to 'Zwaar fysiek werk' answer this question:***<br>Zwaar fysiek werk	○ Alle competitieve sporten hieronder met ‘seasonal’ conditioning

4.1.44	***If 'Wat is het meest bij u van toepassing ten aanzien van uw acitviteit?' is equal to 'Alpine skiën en snowboarden' answer this question:*** Alpine skien en snowboarden	○ Alpine skien en snowboarden
4.1.45	***If 'Wat is het meest bij u van toepassing ten aanzien van uw acitviteit?' is equal to 'Bowlen/curlen' answer this question:*** Bowlen/curlen	○ Bowlen/curlen
4.1.46	***If 'Wat is het meest bij u van toepassing ten aanzien van uw acitviteit?' is equal to 'Golf' answer this question:*** Golf	○ Golf
4.1.47	***If 'Wat is het meest bij u van toepassing ten aanzien van uw acitviteit?' is equal to 'Mountainbike/BMX' answer this question:*** Mountainbike/BMX	○ Mountainbike/BMX
4.1.48	***If 'Wat is het meest bij u van toepassing ten aanzien van uw acitviteit?' is equal to 'Powerliften' answer this question:*** Powerliften	○ Powerliften
4.1.49	***If 'Wat is het meest bij u van toepassing ten aanzien van uw acitviteit?' is equal to 'Zeilen' answer this question:*** Zeilen	○ Zeilen
4.1.50	***If 'Wat is het meest bij u van toepassing ten aanzien van uw acitviteit?' is equal to 'Fysiek werk' answer this question:*** Fysiek werk	○ Fysiek werk
4.1.51	***If 'Wat is het meest bij u van toepassing ten aanzien van uw acitviteit?' is equal to 'Wielrennen' answer this question:*** Wielrennen	○ Wielrennen
4.1.52	***If 'Wat is het meest bij u van toepassing ten aanzien van uw acitviteit?' is equal to 'Paardrijden' answer this question:*** Paardrijden	○ Paardrijden
4.1.53	***If 'Wat is het meest bij u van toepassing ten aanzien van uw acitviteit?' is equal to 'Motorsporten/technische sporten' answer this question:*** Motorsporten/technische sporten	○ Motorsporten/technische sporten
4.1.54	***If 'Wat is het meest bij u van toepassing ten aanzien van uw acitviteit?' is equal to 'Roeien, kayakken' answer this question:*** Roeien, kayakken	○ Roeien, kayakken

4.1.55	***If 'Wat is het meest bij u van toepassing ten aanzien van uw acitviteit?' is equal to 'Boogschieten' answer this question:*** Boogschieten	◯ Boogschieten
4.1.56	***If 'Wat is het meest bij u van toepassing ten aanzien van uw acitviteit?' is equal to 'Water polo en zwemmen' answer this question:*** Water polo en zwemmen	◯ Water polo en zwemmen
4.1.57	***If 'Wat is het meest bij u van toepassing ten aanzien van uw acitviteit?' is equal to 'Kunnen lopen op oneven grond' answer this question:*** Kunnen lopen op oneven grond	◯ Kunnen lopen op oneven grond
4.1.58	***If 'Wat is het meest bij u van toepassing ten aanzien van uw acitviteit?' is equal to 'Geen sport, echter geen limitatie in dagelijkse activiteiten' answer this question:*** Geen sport, echter geen limitatie in dagelijkse activiteiten	◯ Geen sport, echter geen limitatie in dagelijkse activiteiten
4.1.59	***If 'Wat is het meest bij u van toepassing ten aanzien van uw acitviteit?' is equal to 'Kunnen lopen op even grond, maar de dagelijkste activiteiten zijn gelimiteerd' answer this question:*** Kunnen lopen op even grond, maar de dagelijkste activiteiten zijn gelimiteerd	◯ Kunnen lopen op even grond, maar de dagelijkste activiteiten zijn gelimiteerd
4.1.60	***If 'Wat is het meest bij u van toepassing ten aanzien van uw acitviteit?' is equal to 'Niet kunnen lopen, fysieke handicap wegens enkelproblemen' answer this question:*** Niet kunnen lopen, fysieke handicap wegens enkelproblemen	◯ Niet kunnen lopen, fysieke handicap wegens enkelproblemen

Vragenlijst PRIMA studie 52 weken - Hoe tevreden bent u over de enkelklachten?

Number	Question	Answers
5.1	Hoe tevreden bent u over de enkelklachten?	◯ Slecht<br>◯ Matig<br>◯ Goed<br>◯ Uitstekend

Vragenlijst PRIMA studie 52 weken - SF-36

Number	Question	Answers
	Deze vragenlijst gaat over uw standpunten t.a.v. uw gezondheid. Met behulp van deze gegevens kan worden bijgehouden hoe u zich voelt en hoe goed u in staat bent uw gebruikelijke bezigheden uit te voeren. Beantwoord elke vraag door een antwoord aan te klikken. Als u niet zeker weet hoe u een vraag moet beantwoorden, geef dan het best mogelijke antwoord.	
6.1	Hoe zou u over het algemeen uw gezondheid noemen?	◯ Uitstekend ◯ Zeer goed ◯ Goed ◯ Matig ◯ Slecht
6.2	Hoe beoordeelt u nu uw gezondheid over het algemeen vergeleken met een jaar geleden?	◯ Veel beter dan een jaar geleden ◯ Wat beter dan een jaar geleden ◯ Ongeveer hetzelfde als een jaar geleden ◯ Wat slechter dan een jaar geleden ◯ Veel slechter dan een jaar geleden
6.3	Wordt u door uw gezondheid op dit moment beperkt bij forse inspanning, zoals hardlopen, tillen van zware voorwerpen of een veeleisende sport beoefenen?	◯ Ja, ernstig beperkt ◯ Ja, een beetje beperkt ◯ Nee, helemaal niet beperkt
6.4	Wordt u door uw gezondheid op dit moment beperkt bij matige inspanning zoals een tafel verplaatsen, stofzuigen, zwemmen of fietsen?	◯ Ja, ernstig beperkt ◯ Ja, een beetje beperkt ◯ Nee, helemaal niet beperkt
6.5	Wordt u door uw gezondheid op dit moment beperkt bij boodschappen tillen of dragen?	◯ Ja, ernstig beperkt ◯ Ja, een beetje beperkt ◯ Nee, helemaal niet beperkt
6.6	Wordt u door uw gezondheid op dit moment beperkt bij een paar trappen oplopen?	◯ Ja, ernstig beperkt ◯ Ja, een beetje beperkt ◯ Nee, helemaal niet beperkt
6.7	Wordt u door uw gezondheid op dit moment beperkt bij één trap oplopen?	◯ Ja, ernstig beperkt ◯ Ja, een beetje beperkt ◯ Nee, helemaal niet beperkt
6.8	Wordt u door uw gezondheid op dit moment beperkt bij bukken, knielen of hurken?	◯ Ja, ernstig beperkt ◯ Ja, een beetje beperkt ◯ Nee, helemaal niet beperkt
6.9	Wordt u door uw gezondheid op dit moment beperkt bij meer dan één kilometer lopen?	◯ Ja, ernstig beperkt ◯ Ja, een beetje beperkt ◯ Nee, helemaal niet beperkt
6.10	Wordt u door uw gezondheid op dit moment beperkt bij een paar honderd meter lopen?	◯ Ja, ernstig beperkt ◯ Ja, een beetje beperkt ◯ Nee, helemaal niet beperkt

6.11	Wordt u door uw gezondheid op dit moment beperkt bij ongeveer honderd meter lopen?	◯ Ja, ernstig beperkt ◯ Ja, een beetje beperkt ◯ Nee, helemaal niet beperkt
6.12	Wordt u door uw gezondheid op dit moment beperkt bij uzelf wassen of aankleden?	◯ Ja, ernstig beperkt ◯ Ja, een beetje beperkt ◯ Nee, helemaal niet beperkt
6.13	U besteedde in de afgelopen 4 weken minder tijd aan werk of andere bezigheden	◯ Ja ◯ Nee
6.14	U heeft in de afgelopen 4 weken minder bereikt dan u zou willen	◯ Ja ◯ Nee
6.15	U was in de afgelopen 4 weken beperkt in het soort werk of andere bezigheden.	◯ Ja ◯ Nee
6.16	U had de afgelopen 4 weken moeite om uw werk of andere bezigheden uit te voeren (het kostte u bijvoorbeeld extra inspanning).	◯ Ja ◯ Nee
6.17	U besteedde in de afgelopen 4 weken minder tijd aan werk of andere bezigheden ten gevolge van emotionele problemen	◯ Ja ◯ Nee
6.18	U heeft in de afgelopen 4 weken minder bereikt dan u zou willen ten gevolge van emotionele problemen.	◯ Ja ◯ Nee
6.19	U deed de afgelopen 4 weken uw werk of andere bezigheden niet zo zorgvuldig als gewoonlijk ten gevolge van emotionele problemen.	◯ Ja ◯ Nee
6.20	In hoeverre hebben uw lichamelijke gezondheid of emotionele problemen u gedurende de afgelopen 4 weken gehinderd in uw normale omgang met familie, vrienden of buren, of bij activiteiten in groepsverband?	◯ Helemaal niet ◯ Enigszins ◯ Nogal ◯ Veel ◯ Heel erg veel
6.21	Hoeveel lichamelijke pijn heeft u de afgelopen 4 weken gehad?	◯ Geen<br>◯ Heel licht<br>◯ Licht<br>◯ Nogal<br>◯ Ernstig<br>◯ Heel ernstig
6.22	In welke mate bent u de afgelopen 4 weken door de pijn gehinderd in uw normale werk (zowel werk buitenshuis als huishoudelijk werk)?	◯ Helemaal niet<br>◯ Een klein beetje<br>◯ Nogal<br>◯ Veel<br>◯ Heel erg veel

6.23 Voelde u zich levenslustig?

- Altijd
- Meestal
- Vaak
- Soms
- Zelden
- Nooit

6.24 Was u erg zenuwachtig?

- Altijd
- Meestal
- Vaak
- Soms
- Zelden
- Nooit

6.25 Zat u zo in de put dat u niets kon?

- Altijd
- Meestal
- Vaak
- Soms
- Zelden
- Nooit

6.26 Voelde u zich rustig en tevreden?

- Altijd
- Meestal
- Vaak
- Soms
- Zelden
- Nooit

6.27 Had u veel energie?

- Altijd
- Meestal
- Vaak
- Soms
- Zelden
- Nooit

6.28 Voelde u zich somber en neerslachtig?

- Altijd
- Meestal
- Vaak
- Soms
- Zelden
- Nooit

6.29	Voelde u zich uitgeput?	◯ Altijd<br>◯ Meestal<br>◯ Vaak<br>◯ Soms<br>◯ Zelden<br>◯ Nooit
6.30	Was u een gelukkig mens?	◯ Altijd<br>◯ Meestal<br>◯ Vaak<br>◯ Soms<br>◯ Zelden<br>◯ Nooit
6.31	Voelde u zich moe?	◯ Altijd<br>◯ Meestal<br>◯ Vaak<br>◯ Soms<br>◯ Zelden<br>◯ Nooit
6.32	Hoe vaak hebben uw lichamelijke gezondheid of emotionele problemen u gedurende de afgelopen 4 weken gehinderd bij uw sociale activiteiten (zoals vrienden of familie bezoeken etc.)?	◯ Altijd<br>◯ Meestal<br>◯ Soms<br>◯ Zelden<br>◯ Nooit
6.33	Ik lijk wat gemakkelijker ziek te worden dan andere mensen	◯ Volkomen juist<br>◯ Grotendeels juist<br>◯ Weet ik niet<br>◯ Grotendeels onjuist<br>◯ Volkomen onjuist
6.34	Ik ben even gezond als andere mensen die ik ken	◯ Volkomen juist<br>◯ Grotendeels juist<br>◯ Weet ik niet<br>◯ Grotendeels onjuist<br>◯ Volkomen onjuist

6.35	Ik verwacht dat mijn gezondheid achteruit zal gaan	◯ Volkomen juist<br>◯ Grotendeels juist<br>◯ Weet ik niet<br>◯ Grotendeels onjuist<br>◯ Volkomen onjuist
6.36	Mijn gezondheid is uitstekend	◯ Volkomen juist<br>◯ Grotendeels juist<br>◯ Weet ik niet<br>◯ Grotendeels onjuist<br>◯ Volkomen onjuist

Vragenlijst PRIMA studie 52 weken - GAS

Number	Question	Answers
	Tijdens de eerste afspraak werd een duidelijk doel met u afgesproken. Dit werd tevens per email naar u gestuurd. Hoe is de situatie nu ten aanzien van de destijds gestelde doelstelling?	
7.1	Wat is nu de situatie ten aanzien van het tijdens de eerste afspraak afgesproken doelstelling (Goal attainment Scaling)?	◯ Achteruitgang (minder dan de utigangssituatie)<br>◯ Uitgangssituatie<br>◯ Minder dan het doel<br>◯ Doel<br>◯ Meer dan het doel<br>◯ Veel meer dan het doel

Vragenlijst PRIMA studie 52 weken - EQ-5D-3L

Number	Question	Answers
	Deze vragenlijst gaat over uw standpunten t.a.v. uw gezondheid. Met behulp van deze gegevens kan worden bijgehouden hoe u zich voelt en hoe goed u in staat bent uw gebruikelijke bezigheden uit te voeren. Beantwoord elke vraag door een antwoord aan te klikken. Als u niet zeker weet hoe u een vraag moet beantwoorden, geef dan het best mogelijke antwoord.	
8.1	Hoe is het met uw Mobiliteit gesteld?	◯ Ik heb geen problemen met lopen<br>◯ Ik heb enige problemen met lopen<br>◯ Ik ben bedlegerig

8.2	Hoe is het met uw Zelfzorg gesteld?	◯ Ik heb geen problemen om mijzelf te wassen of aan te kleden<br>◯ Ik heb enige problemen om mijzelf te wassen of aan te kleden<br>◯ Ik ben niet in staat om mijzelf te wassen of aan te kleden
8.3	Hoe is het met de Dagelijkse activiteiten (werk, studie, huishouden, gezins- en vrijetijdsactiviteiten) gesteld?	◯ Ik heb geen problemen met mijn dagelijkse activiteiten<br>◯ Ik heb enige problemen met mijn dagelijkse activiteiten<br>◯ Ik ben niet in staat om mijn dagelijkse activiteiten uit te voeren
8.4	Hoe is het met de Pijn/klachten gesteld?	◯ Ik heb geen pijn of andere klachten<br>◯ Ik heb matige pijn of andere klachten<br>◯ Ik heb zeer ernstige pijn of andere klachten
8.5	Hoe is het met de Stemming gesteld?	◯ Ik ben niet angstig of somber<br>◯ Ik ben matig angstig of somber<br>◯ Ik ben erg angstig of somber
	We willen weten hoe goed of slecht uw gezondheid VANDAAG is. Deze meetschaal loopt van 0 tot 100: 100 staat voor de BESTE gezondheid die u zich kunt voorstellen - 0 staat voor de SLECHTSTE gezondheid die u zich kunt voorstellen	
8.6	Uw Gezondheid vandaag	(0.00) (100.00)

Vragenlijst PRIMA studie 52 weken - AOS

Number	Question	Answers
	Instructies: De lijn naast elke vraag staat voor hoeveel PIJN u heeft in verschillende situaties. De linker kant (0) is “geen pijn” en de rechter kant (100) is “ergste pijn denkbaar”. Geef voor de onderstaande situaties op de lijn aan hoeveel PIJN u in de afgelopen week in de enkel had. Als een of meerdere van deze situaties niet van toepassing waren, dan kiest u de "niet van toepassing" optie.	
9.1	Wat was de hoogte van de ergste pijn in de afgelopen week?	(0.00) (100.00)
9.2	Heeft u pijn als u 's ochtends opstaat?	◯ Ja<br>◯ Nee<br>◯ Niet van toepassing

9.2.1	***If 'Heeft u pijn als u 's ochtends opstaat?' is equal to 'Ja' answer this question:***<br>Hoeveel pijn heeft u voordat u 's ochtends opstaat?	(0.00)	(100.00)
9.3	Heeft u pijn wanneer u op blote voeten loopt?	◯ Ja<br>◯ Nee<br>◯ Niet van toepassing	
9.3.1	***If 'Heeft u pijn wanneer u op blote voeten loopt?' is equal to 'Ja' answer this question:***<br>Hoeveel pijn heeft u als u op blote voeten loopt?	(0.00)	(100.00)
9.4	Heeft u pijn wanneer u op blote voeten staat?	◯ Ja<br>◯ Nee<br>◯ Niet van toepassing	
9.4.1	***If 'Heeft u pijn wanneer u op blote voeten staat?' is equal to 'Ja' answer this question:***<br>Hoeveel pijn heeft u als u op blote voeten staat?	(0.00)	(100.00)
9.5	Heeft u pijn wanneer u met schoenen loopt?	◯ Ja<br>◯ Nee<br>◯ Niet van toepassing	
9.5.1	***If 'Heeft u pijn wanneer u met schoenen loopt?' is equal to 'Ja' answer this question:***<br>Hoeveel pijn heeft u wanneer u met schoenen loopt?	(0.00)	(100.00)
9.6	Heeft u pijn wanneer u met schoenen staat?	◯ Ja<br>◯ Nee<br>◯ Niet van toepassing	
9.6.1	***If 'Heeft u pijn wanneer u met schoenen staat?' is equal to 'Ja' answer this question:***<br>Hoeveel pijn heeft u wanneer u met schoenen staat?	(0.00)	(100.00)
9.7	Heeft u pijn wanneer u loopt met steunzolen of een brace?	◯ Ja<br>◯ Nee<br>◯ Niet van toepassing	
9.7.1	***If 'Heeft u pijn wanneer u loopt met steunzolen of een brace?' is equal to 'Ja' answer this question:***<br>Hoeveel pijn heeft u wanneer u loopt met steunzolen of een brace?	(0.00)	(100.00)

9.8	Heeft u pijn wanneer u staat met steunzolen of een brace?	◯ Ja<br>◯ Nee<br>◯ Niet van toepassing
9.8.1	***If 'Heeft u pijn wanneer u staat met steunzolen of een brace?' is equal to 'Ja' answer this question:***<br>Hoeveel pijn heeft u wanneer u staat met steunzolen of een brace?	(0.00) (100.00)
9.9	Heeft u pijn aan het einde van de dag?	◯ Ja<br>◯ Nee<br>◯ Niet van toepassing
9.9.1	***If 'Heeft u pijn aan het einde van de dag?' is equal to 'Ja' answer this question:***<br>Hoeveel pijn heeft u aan het einde van de dag?	(0.00) (100.00)
	Instructies:De lijn naast elke vraag staat voor hoeveel MOEITE u heeft met verschillende activiteiten. De linker kant (0) is "Niet moeilijk" en de rechter kant (100) is "Te moeilijk, niet uitvoerbaar". Geef voor de onderstaande activiteiten op de lijn aan hoeveel MOEITE u in de afgelopen week door enkelklachten had met onderstaande activiteiten. Als een situatie niet van toepassing is, kies dan de optie "niet van toepassing".	
9.10	Heeft u moeite met door het huis lopen?	◯ Ja<br>◯ Nee<br>◯ Niet van toepassing
9.10.1	***If 'Heeft u moeite met door het huis lopen?' is equal to 'Ja' answer this question:***<br>Hoeveel moeite heeft u met door het huis lopen?	(0.00) (100.00)
9.11	Heeft u moeite met buiten lopen op oneven ondergrond?	◯ Ja<br>◯ Nee<br>◯ Niet van toepassing
9.11.1	***If 'Heeft u moeite met buiten lopen op oneven ondergrond?' is equal to 'Ja' answer this question:***<br>Hoeveel moeite heeft u met buiten lopen op oneven ondergrond?	(0.00) (100.00)
9.12	Heeft u moeite met een paar honderd meter lopen?	◯ Ja<br>◯ Nee<br>◯ Niet van toepassing
9.12.1	***If 'Heeft u moeite met een paar honderd meter lopen?' is equal to 'Ja' answer this question:***<br>Hoeveel moeite heeft u met een paar honderd meter lopen?	(0.00) (100.00)

For peer review only - http://bmjopen.bmj.com/site/about/guidelines.xhtml

9.13	Heeft u moeite met een trap oplopen?	◯ Ja<br>◯ Nee<br>◯ Niet van toepassing	
9.13.1	***If 'Heeft u moeite met een trap oplopen?' is equal to 'Ja' answer this question:***<br>Hoeveel moeite heeft u met een trap oplopen?	(0.00)	(100.00)
9.14	Heeft u moeite met een trap aflopen?	◯ Ja<br>◯ Nee<br>◯ Niet van toepassing	
9.14.1	***If 'Heeft u moeite met een trap aflopen?' is equal to 'Ja' answer this question:***<br>Hoeveel moeite heeft u met een trap aflopen?	(0.00)	(100.00)
9.15	Heeft u moeite met op de tenen staan?	◯ Ja<br>◯ Nee<br>◯ Niet van toepassing	
9.15.1	***If 'Heeft u moeite met op de tenen staan?' is equal to 'Ja' answer this question:***<br>Hoeveel moeite heeft u met op de tenen staan?	(0.00)	(100.00)
9.16	Heeft u moeite met opstaan uit de stoel?	◯ Ja<br>◯ Nee<br>◯ Niet van toepassing	
9.16.1	***If 'Heeft u moeite met opstaan uit de stoel?' is equal to 'Ja' answer this question:***<br>Hoeveel moeite heeft u met opstaan uit de stoel?	(0.00)	(100.00)
9.17	Heeft u moeite met het op- of afstappen van stoepranden?	◯ Ja<br>◯ Nee<br>◯ Niet van toepassing	
9.17.1	***If 'Heeft u moeite met het op- of afstappen van stoepranden?' is equal to 'Ja' answer this question:***<br>Hoeveel moeite heeft u met het op- of afstappen van stoepranden?	(0.00)	(100.00)
9.18	Heeft u moeite met snel lopen of rennen?	◯ Ja<br>◯ Nee<br>◯ Niet van toepassing	

9.18.1	***If 'Heeft u moeite met snel lopen of rennen?' is equal to 'Ja' answer this question:*** Hoeveel moeite heeft u met snel lopen of rennen?	(0.00)	(100.00)

Vragenlijst PRIMA studie 52 weken - FAOS

Number	Question	Answers
	Deze lijst vraagt naar uw mening over uw voet/enkel. Uw antwoorden geven ons een beeld van uw voet/enkel klachten en hoe u in staat bent om alledaagse activiteiten uit te voeren in uw huidige situatie. Beantwoorden van een vraag doet u door het aanklikken van een vakje met het volgens u meest juiste antwoord (één vakje per vraag). Als u niet zeker weet hoe u een vraag moet beantwoorden, geeft u dan het antwoord dat volgens u het meest op uw situatie van toepassing is. Deze vraag heeft betrekking op het voorkomen van voet/enkel klachten in de afgelopen week.	
10.1	Is uw voet/enkel gezwollen?	◯ Nooit ◯ Zelden ◯ Soms ◯ Vaak ◯ Altijd
10.2	Voelt u gekraak of hoort u klikken of een ander vreemd geluid wanneer u de voet/enkel beweegt?	◯ Nooit ◯ Zelden ◯ Soms ◯ Vaak ◯ Altijd
10.3	Hapert uw enkel of blokkeert uw enkel ('op slot' gaan zitten) wanneer u deze beweegt?	◯ Nooit ◯ Zelden ◯ Soms ◯ Vaak ◯ Altijd
10.4	Kunt u de voet/enkel volledig strekken?	◯ Altijd ◯ Vaak ◯ Soms ◯ Zelden ◯ Nooit
10.5	Kunt u de voet volledig naar u toe buigen?	◯ Altijd ◯ Vaak ◯ Soms ◯ Zelden ◯ Nooit
10.6	In welke mate heeft u een stijf gevoel in de voet/enkel 's ochtends bij het wakker worden?	◯ Niet ◯ Mild ◯ Matig ◯ Ernstig ◯ Zeer ernstig
10.7	In welke mate heeft u een stijf gevoel in de voet/enkel na zitten, liggen of rusten later op de dag?	◯ Niet ◯ Mild ◯ Matig ◯ Ernstig ◯ Zeer ernstig
10.8	Hoe vaak heeft u pijn in uw voet/enkel?	◯ Nooit ◯ Maandelijks ◯ Wekelijks ◯ Dagelijks ◯ Altijd

10.9	Hoeveel voet/enkel pijn heeft u gehad in de afgelopen week bij draaien als uw voet/enkel op de grond staat?	◯ Geen<br>◯ Mild<br>◯ Matig<br>◯ Ernstig<br>◯ Zeer ernstig
10.10	Hoeveel voet/enkel pijn heeft u gehad in de afgelopen week bij het volledig uitstrekken van de voet/enkel?	◯ Geen<br>◯ Mild<br>◯ Matig<br>◯ Ernstig<br>◯ Zeer ernstig
10.11	Hoeveel voet/enkel pijn heeft u gehad in de afgelopen week bij het volledig naar u toe buigen/optrekken van de voet/enkel	◯ Geen<br>◯ Mild<br>◯ Matig<br>◯ Ernstig<br>◯ Zeer ernstig
10.12	Hoeveel voet/enkel pijn heeft u gehad in de afgelopen week bij het lopen op een vlakke ondergrond	◯ Geen<br>◯ Mild<br>◯ Matig<br>◯ Ernstig<br>◯ Zeer ernstig
10.13	Hoeveel voet/enkel pijn heeft u gehad in de afgelopen week bij het trap op- en trap af lopen?	◯ Geen<br>◯ Mild<br>◯ Matig<br>◯ Ernstig<br>◯ Zeer ernstig
10.14	Hoeveel voet/enkel pijn heeft u gehad in de afgelopen week 's nachts in bed?	◯ Geen<br>◯ Mild<br>◯ Matig<br>◯ Ernstig<br>◯ Zeer ernstig
10.15	Hoeveel voet/enkel pijn heeft u gehad in de afgelopen week bij het zitten of liggen?	◯ Geen<br>◯ Mild<br>◯ Matig<br>◯ Ernstig<br>◯ Zeer ernstig

For peer review only - http://bmjopen.bmj.com/site/about/guidelines.xhtml

10.16	Hoeveel voet/enkel pijn heeft u gehad in de afgelopen week bij het rechtop staan?	◯ Geen<br>◯ Mild<br>◯ Matig<br>◯ Ernstig<br>◯ Zeer ernstig
10.17	In welke mate werd u gehinderd bij het trap aflopen?	◯ Niet ◯ Mild ◯ Matig ◯ Ernstig ◯ Zeer ernstig
10.18	In welke mate werd u gehinderd bij het trap op lopen?	◯ Niet ◯ Mild ◯ Matig ◯ Ernstig ◯ Zeer ernstig
10.19	In welke mate werd u gehinderd als u vanuit een zittende positie ging staan?	◯ Niet ◯ Mild ◯ Matig ◯ Ernstig ◯ Zeer ernstig
10.20	In welke mate werd u gehinderd bij het staan?	◯ Niet ◯ Mild ◯ Matig ◯ Ernstig ◯ Zeer ernstig
10.21	In welke mate werd u gehinderd bij het naar de grond buigen/iets oprapen?	◯ Niet ◯ Mild ◯ Matig ◯ Ernstig ◯ Zeer ernstig
10.22	In welke mate werd u gehinderd bij het lopen op een vlakke ondergrond?	◯ Niet ◯ Mild ◯ Matig ◯ Ernstig ◯ Zeer ernstig
10.23	In welke mate werd u gehinderd bij het in- en uit de auto stappen?	◯ Niet ◯ Mild ◯ Matig ◯ Ernstig ◯ Zeer ernstig
10.24	In welke mate werd u gehinderd bij het boodschappen doen?	◯ Niet ◯ Mild ◯ Matig ◯ Ernstig ◯ Zeer ernstig
10.25	In welke mate werd u gehinderd bij sokken/panty's aantrekken?	◯ Niet ◯ Mild ◯ Matig ◯ Ernstig ◯ Zeer ernstig
10.26	In welke mate werd u gehinderd bij het opstaan uit bed?	◯ Niet ◯ Mild ◯ Matig ◯ Ernstig ◯ Zeer ernstig
10.27	In welke mate werd u gehinderd bij het sokken uittrekken?	◯ Niet ◯ Mild ◯ Matig ◯ Ernstig ◯ Zeer ernstig
10.28	In welke mate werd u gehinderd bij het in bed liggen (omdraaien, lange tijd uw voet/enkel in dezelfde positie houden)?	◯ Niet ◯ Mild ◯ Matig ◯ Ernstig ◯ Zeer ernstig

10.29	In welke mate werd u gehinderd bij het in/uit bad stappen?	◯ Niet ◯ Mild ◯ Matig ◯ Ernstig ◯ Zeer ernstig
10.30	In welke mate werd u gehinderd bij zitten?	◯ Niet ◯ Mild ◯ Matig ◯ Ernstig ◯ Zeer ernstig
10.31	In welke mate werd u gehinderd bij het toilet op en af gaan?	◯ Niet ◯ Mild ◯ Matig ◯ Ernstig ◯ Zeer ernstig
10.32	In welke mate werd u gehinderd bij zwaar huishoudelijk werk (bijvoorbeeld zware dozen sjouwen, vloer schrobben)?	◯ Niet ◯ Mild ◯ Matig ◯ Ernstig ◯ Zeer ernstig
10.33	In welke mate werd u gehinderd bij licht huishoudeijk werk (bijvoorbeeld koken, afstoffen)?	◯ Niet ◯ Mild ◯ Matig ◯ Ernstig ◯ Zeer ernstig
10.34	In welke mate werd u gehinderd bij hurken?	◯ Niet ◯ Mild ◯ Matig ◯ Ernstig ◯ Zeer ernstig
10.35	In welke mate werd u gehinderd bij hardlopen?	◯ Niet ◯ Mild ◯ Matig ◯ Ernstig ◯ Zeer ernstig
10.36	In welke mate werd u gehinderd bij springen?	◯ Niet ◯ Mild ◯ Matig ◯ Ernstig ◯ Zeer ernstig
10.37	In welke mate werd u gehinderd bij ronddraaien op uw aangedane voet/enkel?	◯ Niet ◯ Mild ◯ Matig ◯ Ernstig ◯ Zeer ernstig
10.38	In welke mate werd u gehinderd bij knielen?	◯ Niet ◯ Mild ◯ Matig ◯ Ernstig ◯ Zeer ernstig
10.39	Hoe vaak bent u zich bewust van uw voet/enkel probleem?	◯ Nooit ◯ Maandelijks ◯ Wekelijks ◯ Dagelijks ◯ Altijd
10.40	Heeft u uw leven veranderd om activiteiten te vermijden die schadelijk kunnen zijn voor uw voet/enkel?	◯ Niet<br>◯ Enigszins<br>◯ Matig<br>◯ Behoorlijk<br>◯ Volledig

10.41	In hoeverre kunt u op uw voet/enkel vertrouwen?	◯ Volledig<br>◯ Behoorlijk<br>◯ Matig<br>◯ Enigzins<br>◯ Niet
10.42	In het algemeen, in welke mate ondervindt u hinder van uw voet/enkel	◯ Geen<br>◯ Mild<br>◯ Matig<br>◯ Ernstig<br>◯ Zeer ernstig

Vragenlijst PRIMA studie 52 weken - PRODISQ - Gezondheid en werk

Number	Question	Answers
11.1	Wat is uw leeftijd?	[ ] jaar
11.2	Wat is uw geslacht?	◯ Man<br>◯ Vrouw
11.3	Wat is de hoogste opleiding die u heeft afgemaakt (Zoek uw hoogste opleiding en kruis het hokje daarvoor aan)?	☐ Ik heb geen school of opleiding afgemaakt<br>☐ Lagere school of basisschool<br>☐ Huishoudschool, vbo, lbo, lts, leao of lhno<br>☐ Mavo, mulo, ivo of vmbo<br>☐ Mbo, mts, meao, mhno, inas of intas<br>☐ Havo, vwo, hbs, mms, atheneum of gymnasium<br>☐ Hbo, hts, heao of hhno<br>☐ Universiteit<br>☐ Ik heb een andere opleiding afgemaakt, namelijk
11.3.1	***If 'Wat is de hoogste opleiding die u heeft afgemaakt (Zoek uw hoogste opleiding en kruis het hokje daarvoor aan)?' is equal to 'Ik heb een andere opleiding afgemaakt, namelijk' answer this question:***<br>Wat is de hoogste opleiding die u heeft afgemaakt?	[ ]

11.4	Wat doet u in het dagelijks leven?	☐ Ik zit op school, ik studeer<br>☐ Ik werk in loondienst<br>☐ Ik ben zelfstandig ondernemer<br>☐ Ik ben huisvrouw, huisman<br>☐ Ik ben werkloos<br>☐ Ik ben arbeidsongeschikt<br>☐ Ik ben met pensioen of prepensioen<br>☐ Ik doe iets anders, namelijk
11.4.1	***If 'Wat doet u in het dagelijks leven?' is equal to 'Ik ben arbeidsongeschikt' answer this question:***<br>Wat doet u in het dagelijks leven?	
11.4.2	***If 'Wat doet u in het dagelijks leven?' is equal to 'Ik doe iets anders, namelijk' answer this question:***<br>Wat doet u in het dagelijks leven?	
11.5	Hebt u betaald werk?	◯ Nee<br>◯ Ja
11.5.1	***If 'Hebt u betaald werk?' is equal to 'Ja' answer this question:***<br>Wat is uw beroep?	
11.5.2	***If 'Hebt u betaald werk?' is equal to 'Ja' answer this question:***<br>Hoeveel uur per week werkt u (Tel alleen de uren waarvoor u betaald wordt)?	uren
11.5.3	***If 'Hebt u betaald werk?' is equal to 'Ja' answer this question:***<br>Hoeveel dagen in de week werkt u?	Dagen
11.5.4	***If 'Hebt u betaald werk?' is equal to 'Ja' answer this question:***<br>Bent u in de afgelopen 4 weken afwezig geweest van uw werk omdat u ziek was?	◯ Nee<br>◯ Ja
11.5.4.1	***If 'Bent u in de afgelopen 4 weken afwezig geweest van uw werk omdat u ziek was?' is equal to 'Ja' answer this question:***<br>Bent u in de afgelopen 4 weken afwezig geweest van uw werk omdat u ziek was (Tel alleen de werkdagen in de afgelopen 4 weken)?	dagen afwezig geweest
11.5.4.2	***If 'Bent u in de afgelopen 4 weken afwezig geweest van uw werk omdat u ziek was?' is equal to 'Ja' answer this question:***<br>Was u langer dan de gehele periode van 4 weken afwezig van uw werk doordat u ziek was?	◯ Nee<br>◯ Ja

11.5.4.2.1	***If 'Was u langer dan de gehele periode van 4 weken afwezig van uw werk doordat u ziek was?' is equal to 'Ja' answer this question:*** Wanneer heeft u zich ziek gemeld?	[ ] [ ] [ ] *(dd-mm-yyyy)*
11.5.5	***If 'Hebt u betaald werk?' is equal to 'Ja' answer this question:*** Waren er in de afgelopen 4 weken, dagen waarop u wel gewerkt heeft, maar tijdens uw werk last had van lichamelijke of psychische problemen?	◯ Ja<br>◯ Nee
11.5.5.1	***If 'Waren er in de afgelopen 4 weken, dagen waarop u wel gewerkt heeft, maar tijdens uw werk last had van lichamelijke of psychische problemen?' is equal to 'Ja' answer this question:*** Op hoeveel werkdagen had u tijdens uw werk last van uw lichamelijke of psychische problemen?	[ ] werkdagen
11.5.5.2	***If 'Waren er in de afgelopen 4 weken, dagen waarop u wel gewerkt heeft, maar tijdens uw werk last had van lichamelijke of psychische problemen?' is equal to 'Ja' answer this question:*** Op de dagen dat u last had, kon u misschien niet zoveel werk doen als normaal. Hoeveel werk kon u op deze dagen gemiddeld doen?	Ik kon op deze dagen niks doen (0.00) — Ik kon net zoveel doen als normaal (10.00)
11.6	Waren er dagen waarop u minder onbetaald werk kon doen door uw lichamelijke of psychische problemen?	◯ Nee<br>◯ Ja
11.6.1	***If 'Waren er dagen waarop u minder onbetaald werk kon doen door uw lichamelijke of psychische problemen?' is equal to 'Ja' answer this question:*** Op hoeveel dagen was dit zo?	[ ] Dagen
11.6.2	***If 'Waren er dagen waarop u minder onbetaald werk kon doen door uw lichamelijke of psychische problemen?' is equal to 'Ja' answer this question:*** Stel dat iemand, bijvoorbeeld uw partner, familielid of een bekende, u op deze dagen had geholpen. En al het onbetaalde werk wat u niet kon doen, voor u had gedaan. Hoeveel uur was die persoon hier op deze dagen dan gemiddeld mee bezig geweest?	[ ] uur op deze dagen

Vragenlijst PRIMA studie 52 weken - PRODISQ - Zorggebruik

Number	Question	Answers
	Wij willen graag weten met welke dokters u in de afgelopen 3 maanden een afspraak had. Het gaat om afspraken voor uzelf. Ook andere zorgverleners tellen mee. Bijvoorbeeld de fysiotherapeut.	

12.1	Bent u in de afgelopen 3 maanden naar uw huisarts geweest?	◯ Nee<br>◯ Ja
12.1.1	***If 'Bent u in de afgelopen 3 maanden naar uw huisarts geweest?' is equal to 'Ja' answer this question:***<br>Hoeveel afspraken had u?	[ ] afspraken
12.2	Bent u in de afgelopen 3 maanden in contact geweest met een maatschappelijk werker?	◯ Nee<br>◯ Ja
12.2.1	***If 'Bent u in de afgelopen 3 maanden in contact geweest met een maatschappelijk werker?' is equal to 'Ja' answer this question:***<br>Hoeveel afspraken had u?	[ ] afspraken
12.3	Bent u in de afgelopen 3 maanden naar een fysiotherapeut geweest? Of een caesartherapeut, therapeut mensendieck of een manueel therapeut?	◯ Nee<br>◯ Ja
12.3.1	***If 'Bent u in de afgelopen 3 maanden naar een fysiotherapeut geweest? Of een caesartherapeut, therapeut mensendieck of een manueel therapeut?' is equal to 'Ja' answer this question:***<br>Hoeveel afspraken had u?	[ ] afspraken
12.4	Bent u in de afgelopen 3 maanden naar een ergotherapeut geweest?	◯ Nee<br>◯ Ja
12.4.1	***If 'Bent u in de afgelopen 3 maanden naar een ergotherapeut geweest?' is equal to 'Ja' answer this question:***<br>Hoeveel afspraken had u?	[ ] afspraken
12.5	Bent u in de afgelopen 3 maanden naar een logopedist geweest?	◯ Nee<br>◯ Ja
12.5.1	***If 'Bent u in de afgelopen 3 maanden naar een logopedist geweest?' is equal to 'Ja' answer this question:***<br>Hoeveel afspraken had u?	[ ] afspraken
12.6	Bent u in de afgelopen 3 maanden naar een diëtist geweest?	◯ Nee<br>◯ Ja
12.6.1	***If 'Bent u in de afgelopen 3 maanden naar een diëtist geweest?' is equal to 'Ja' answer this question:***<br>Hoeveel afspraken had u?	[ ] afspraken

12.7	Bent u in de afgelopen 3 maanden naar een homeopaat geweest? Of een acupuncturist?	◯ Nee<br>◯ Ja
12.7.1	***If 'Bent u in de afgelopen 3 maanden naar een homeopaat geweest? Of een acupuncturist?' is equal to 'Ja' answer this question:***<br>Hoeveel afspraken had u?	[ ] afspraken
12.8	Bent u in de afgelopen 3 maanden naar een psycholoog geweest? Of een psychotherapeut of psychiater?	◯ Nee<br>◯ Ja
12.8.1	***If 'Bent u in de afgelopen 3 maanden naar een psycholoog geweest? Of een psychotherapeut of psychiater?' is equal to 'Ja' answer this question:***<br>Hoeveel afspraken had u?	[ ] afspraken
12.9	Heeft u in de afgelopen 3 maanden afspraken gehad met de bedrijfsarts?	◯ Nee<br>◯ Ja
12.9.1	***If 'Heeft u in de afgelopen 3 maanden afspraken gehad met de bedrijfsarts?' is equal to 'Ja' answer this question:***<br>Hoeveel afspraken had u?	[ ] afspraken
12.10	Heeft u in de afgelopen 3 maanden hulp van de thuiszorg gehad?	◯ Ja<br>◯ Nee
12.10.1	***If 'Heeft u in de afgelopen 3 maanden hulp van de thuiszorg gehad?' is equal to 'Ja' answer this question:***<br>Wat voor hulp van de thuiszorg heeft u gehad in de afgelopen 3 maanden?	☐ Huishoudelijke hulp; voorbeeld: stofzuigen, bed opmaken, boodschappen doen<br>☐ Verzorging van uzelf; voorbeeld: hulp bij douchen of aankleden<br>☐ Verpleging; voorbeeld: verband omdoen, medicijnen geven, bloeddruk meten
12.10.1.1	***If 'Wat voor hulp van de thuiszorg heeft u gehad in de afgelopen 3 maanden?' is equal to 'Huishoudelijke hulp; voorbeeld: stofzuigen, bed opmaken, boodschappen doen' answer this question:***<br>Hoeveel weken heeft u deze thuiszorg gehad? Huishoudelijke hulp:	[ ] weken in de afgelopen 3 maanden
12.10.1.2	***If 'Wat voor hulp van de thuiszorg heeft u gehad in de afgelopen 3 maanden?' is equal to 'Verzorging van uzelf; voorbeeld: hulp bij douchen of aankleden' answer this question:***<br>Hoeveel weken heeft u deze thuiszorg gehad? Verzorging van uzelf:	[ ] weken in de afgelopen 3 maanden

12.10.1.3	***If 'Wat voor hulp van de thuiszorg heeft u gehad in de afgelopen 3 maanden?' is equal to 'Verpleging; voorbeeld: verband omdoen, medicijnen geven, bloeddruk meten' answer this question:***<br>Hoeveel weken heeft u deze thuiszorg gehad? Verpleging:	____ weken in de afgelopen 3 maanden
12.10.1.4	***If 'Wat voor hulp van de thuiszorg heeft u gehad in de afgelopen 3 maanden?' is equal to 'Huishoudelijke hulp; voorbeeld: stofzuigen, bed opmaken, boodschappen doen' answer this question:***<br>Hoeveel uur thuiszorg kreeg u in deze weken gemiddeld? Huishoudelijke hulp: gemiddeld	____ uur in de week
12.10.1.5	***If 'Wat voor hulp van de thuiszorg heeft u gehad in de afgelopen 3 maanden?' is equal to 'Verzorging van uzelf; voorbeeld: hulp bij douchen of aankleden' answer this question:***<br>Hoeveel uur thuiszorg kreeg u in deze weken gemiddeld? Verzorging van uzelf: gemiddeld	____ uur in de week
12.10.1.6	***If 'Wat voor hulp van de thuiszorg heeft u gehad in de afgelopen 3 maanden?' is equal to 'Verpleging; voorbeeld: verband omdoen, medicijnen geven, bloeddruk meten' answer this question:***<br>Hoeveel uur thuiszorg kreeg u in deze weken gemiddeld? Verpleging: gemiddeld	____ uur in de week
12.11	Heeft u in de afgelopen 3 maanden medicijnen gebruikt?	○ Ja<br>○ Nee
12.11.1	***If 'Heeft u in de afgelopen 3 maanden medicijnen gebruikt?' is equal to 'Ja' answer this question:***<br>Welke medicijnen heeft u in de afgelopen 3 maanden gebruikt?	____
12.12	Bent u in de afgelopen 3 maanden op de spoedeisende eerste hulp van een ziekenhuis geweest? (Een andere naam voor spoedeisende eerste hulp is EHBO)	○ Nee<br>○ Ja
12.12.1	***If 'Bent u in de afgelopen 3 maanden op de spoedeisende eerste hulp van een ziekenhuis geweest? (Een andere naam voor spoedeisende eerste hulp is EHBO)' is equal to 'Ja' answer this question:***<br>Hoevaak bent u geweest?	____ keer
12.13	Bent u in de afgelopen 3 maanden met een ambulance naar het ziekenhuis gebracht? (Een andere naam voor ambulance is ziekenauto)	○ Nee<br>○ Ja

12.13.1	***If 'Bent u in de afgelopen 3 maanden met een ambulance naar het ziekenhuis gebracht? (Een andere naam voor ambulance is ziekenauto)' is equal to 'Ja' answer this question:***<br>Hoe vaak bent u naar het ziekenhuis gebracht?	[ ] keer
12.14	Had u in de afgelopen 3 maanden een afspraak bij de polikliniek van het ziekenhuis? (Het gaat om afspraken voor uzelf met een dokter. Bijvoorbeeld met de cardioloog, reumatoloog of neuroloog)	◯ Ja<br>◯ Nee
12.14.1	***If 'Had u in de afgelopen 3 maanden een afspraak bij de polikliniek van het ziekenhuis? (Het gaat om afspraken voor uzelf met een dokter. Bijvoorbeeld met de cardioloog, reumatoloog of neuroloog)' is equal to 'Ja' answer this question:***<br>Bij welke soorten dokters bent u in de afgelopen 3 maanden in het ziekenhuis geweest? En hoe vaak?	[ ]
12.15	Bent u in de afgelopen 3 maanden overdag in het ziekenhuis geweest voor een behandeling? (U bleef dus niet slapen. U kwam bijvoorbeeld voor een bloedtransfusie, nierdialyse of chemokuur)	◯ Ja<br>◯ Nee
12.15.1	***If 'Bent u in de afgelopen 3 maanden overdag in het ziekenhuis geweest voor een behandeling? (U bleef dus niet slapen. U kwam bijvoorbeeld voor een bloedtransfusie, nierdialyse of chemokuur)' is equal to 'Ja' answer this question:***<br>Voor welke soort behandeling was dit?	[ ]
12.15.2	***If 'Bent u in de afgelopen 3 maanden overdag in het ziekenhuis geweest voor een behandeling? (U bleef dus niet slapen. U kwam bijvoorbeeld voor een bloedtransfusie, nierdialyse of chemokuur)' is equal to 'Ja' answer this question:***<br>Hoeveel keer moest u in de afgelopen 3 maanden voor deze behandelingen naar het ziekenhuis?	[ ]
12.16	Bent u in de afgelopen 3 maanden ergens anders geweest voor een behandeling overdag? (U bleef dus niet slapen. U ging bijvoorbeeld naar de dagopvang van een woon-/zorgcentrum of een psychiatrische instelling. Of naar de dagbehandeling van een revalidatiecentrum)	◯ Ja<br>◯ Nee
12.16.1	***If 'Bent u in de afgelopen 3 maanden ergens anders geweest voor een behandeling overdag? (U bleef dus niet slapen. U ging bijvoorbeeld naar de dagopvang van een woon-/zorgcentrum of een psychiatrische instelling. Of naar de dagbehandeling van een revalidatiecentrum)' is equal to 'Ja' answer this question:***<br>Wat voor instelling was dit?	☐ Woon-/zorgcentrum<br>☐ Revalidatiecentrum<br>☐ Psychiatrische instelling<br>☐ Een andere instelling, namelijk

12.16.1.1	***If 'Wat voor instelling was dit?' is equal to 'Een andere instelling, namelijk' answer this question:*** Wat voor instelling was dit?	
12.16.1.2	***If 'Wat voor instelling was dit?' is equal to 'Woon-/zorgcentrum' answer this question:*** Hoe vaak moest u hier in de afgelopen 3 maanden naartoe? Naar het woon-/zorgcentrum:	keer in de afgelopen 3 maanden
12.16.1.3	***If 'Wat voor instelling was dit?' is equal to 'Revalidatiecentrum' answer this question:*** Hoe vaak moest u hier in de afgelopen 3 maanden naartoe? Naar het revalidatiecentrum:	keer in de afgelopen 3 maanden
12.16.1.4	***If 'Wat voor instelling was dit?' is equal to 'Psychiatrische instelling' answer this question:*** Hoe vaak moest u hier in de afgelopen 3 maanden naartoe? Naar de psychiatrische instelling:	keer in de afgelopen 3 maanden
12.16.1.5	***If 'Wat voor instelling was dit?' is equal to 'Een andere instelling, namelijk' answer this question:*** Hoe vaak moest u hier in de afgelopen 3 maanden naartoe? Naar de andere instelling:	keer in de afgelopen 3 maanden
12.17	Heeft u in de afgelopen 3 maanden weleens in het ziekenhuis gelegen? (U moest dus blijven slapen. Bijvoorbeeld omdat u geopereerd was en niet meteen naar huis kon)	◯ Ja<br>◯ Nee
12.17.1	***If 'Heeft u in de afgelopen 3 maanden weleens in het ziekenhuis gelegen? (U moest dus blijven slapen. Bijvoorbeeld omdat u geopereerd was en niet meteen naar huis kon)' is equal to 'Ja' answer this question:*** Hoe vaak heeft u in de afgelopen 3 maanden in het ziekenhuis gelegen?	keer in de afgelopen 3 maanden
12.17.2	***If 'Heeft u in de afgelopen 3 maanden weleens in het ziekenhuis gelegen? (U moest dus blijven slapen. Bijvoorbeeld omdat u geopereerd was en niet meteen naar huis kon)' is equal to 'Ja' answer this question:*** Hoe lang heeft u in het ziekenhuis gelegen?	dagen in de afgelopen 3 maanden
12.18	Moest u in de afgelopen 3 maanden ergens anders blijven slapen voor uw gezondheid? (Bijvoorbeeld in een woon-/zorgcentrum, psychiatrische instelling of revalidatiecentrum)	◯ Ja<br>◯ Nee

12.18.1	***If 'Moest u in de afgelopen 3 maanden ergens anders blijven slapen voor uw gezondheid? (Bijvoorbeeld in een woon-/zorgcentrum, psychiatrische instelling of revalidatiecentrum)' is equal to 'Ja' answer this question:***<br>Wat voor instelling was dit?	☐ Woon-/zorgcentrum<br>☐ Revalidatiecentrum<br>☐ Psychiatrische instelling<br>☐ Een andere instelling, namelijk
12.18.1.1	***If 'Wat voor instelling was dit?' is equal to 'Een andere instelling, namelijk' answer this question:***<br>Een andere instelling, namelijk	
12.18.1.2	***If 'Wat voor instelling was dit?' is equal to 'Woon-/zorgcentrum' answer this question:***<br>Hoe lang bent u in deze instelling geweest? In het woon-/zorgcentrum:	______ dagen in de afgelopen 3 maanden
12.18.1.3	***If 'Wat voor instelling was dit?' is equal to 'Revalidatiecentrum' answer this question:***<br>Hoe lang bent u in deze instelling geweest? In het revalidatiecentrum:	______ dagen in de afgelopen 3 maanden
12.18.1.4	***If 'Wat voor instelling was dit?' is equal to 'Psychiatrische instelling' answer this question:***<br>Hoe lang bent u in deze instelling geweest? In de psychiatrische instelling:	______ dagen in de afgelopen 3 maanden
12.18.1.5	***If 'Wat voor instelling was dit?' is equal to 'Een andere instelling, namelijk' answer this question:***<br>Hoe lang bent u in deze instelling geweest? In de andere instelling:	______ dagen in de afgelopen 3 maanden
12.19	Heeft u in de afgelopen 3 maanden hulp gekregen van een familielid of een bekende vanwege uw lichamelijke of psychische problemen?	◯ Ja<br>◯ Nee
12.19.1	***If 'Heeft u in de afgelopen 3 maanden hulp gekregen van een familielid of een bekende vanwege uw lichamelijke of psychische problemen?' is equal to 'Ja' answer this question:***<br>Wat voor hulp van familieleden of bekenden heeft u gehad in de afgelopen 3 maanden?	☐ Huishoudelijke hulp - voorbeeld: stofzuigen, bed opmaken, boodschappen doen, klaarmaken van eten en drinken, verzorgen van kinderen<br>☐ Verzorging van uzelf - voorbeeld: hulp bij douchen of aankleden, hulp bij het eten en drinken of het geven van medicijnen<br>☐ Praktische hulp - voorbeeld: ondersteuning bij wandelen, het maken van uitstapjes of bezoekjes aan bekenden, bezoeken aan de huisarts of het ziekenhuis, het regelen van hulp of het regelen van financiële zaken

12.19.1.1	***If 'Wat voor hulp van familieleden of bekenden heeft u gehad in de afgelopen 3 maanden?' is equal to 'Huishoudelijke hulp - voorbeeld: stofzuigen, bed opmaken, boodschappen doen, klaarmaken van eten en drinken, verzorgen van kinderen' answer this question:***<br>Hoeveel weken heeft u deze hulp gehad? Huishoudelijke hulp:	______ weken in de afgelopen 3 maanden
12.19.1.2	***If 'Wat voor hulp van familieleden of bekenden heeft u gehad in de afgelopen 3 maanden?' is equal to 'Verzorging van uzelf - voorbeeld: hulp bij douchen of aankleden, hulp bij het eten en drinken of het geven van medicijnen' answer this question:***<br>Hoeveel weken heeft u deze hulp gehad? Verzorging van uzelf:	______ weken in de afgelopen 3 maanden
12.19.1.3	***If 'Wat voor hulp van familieleden of bekenden heeft u gehad in de afgelopen 3 maanden?' is equal to 'Praktische hulp - voorbeeld: ondersteuning bij wandelen, het maken van uitstapjes of bezoekjes aan bekenden, bezoeken aan de huisarts of het ziekenhuis, het regelen van hulp of het regelen van financiële zaken' answer this question:***<br>Hoeveel uur hulp kreeg u in deze weken gemiddeld? Praktische hulp:	______ weken in de afgelopen 3 maanden
12.19.1.4	***If 'Wat voor hulp van familieleden of bekenden heeft u gehad in de afgelopen 3 maanden?' is equal to 'Huishoudelijke hulp - voorbeeld: stofzuigen, bed opmaken, boodschappen doen, klaarmaken van eten en drinken, verzorgen van kinderen' answer this question:***<br>Hoeveel uur hulp kreeg u in deze weken gemiddeld? Huishoudelijke hulp: gemiddeld	______ uur in de week
12.19.1.5	***If 'Wat voor hulp van familieleden of bekenden heeft u gehad in de afgelopen 3 maanden?' is equal to 'Verzorging van uzelf - voorbeeld: hulp bij douchen of aankleden, hulp bij het eten en drinken of het geven van medicijnen' answer this question:***<br>Hoeveel uur hulp kreeg u in deze weken gemiddeld? Verzorging van uzelf: gemiddeld	______ uur in de week
12.19.1.6	***If 'Wat voor hulp van familieleden of bekenden heeft u gehad in de afgelopen 3 maanden?' is equal to 'Praktische hulp - voorbeeld: ondersteuning bij wandelen, het maken van uitstapjes of bezoekjes aan bekenden, bezoeken aan de huisarts of het ziekenhuis, het regelen van hulp of het regelen van financiële zaken' answer this question:***<br>Hoeveel uur hulp kreeg u in deze weken gemiddeld? Praktische hulp: gemiddeld	______ uur in de week

Vragenlijst PRIMA studie 52 weken - Dank

Number	Question	Answers
13.1	Hartelijk dank voor de tijd die u heeft genomen om de vragenlijsten in te vullen. Indien u nog opmerkingen heeft kunt u deze hierin plaatsen.	

Survey 'Vragenlijst PRIMA studie 6 weken'

Vragenlijst PRIMA studie 6 weken - AOFAS - Pijn

Number	Question	Answers
1.1	Hoeveel pijn ervaart u aan uw voet?	☐ Geen<br>☐ Milde pijnklachten of af en toe pijn<br>☐ Matige pijnklachten, dagelijks<br>☐ Vrijwel continu erge pijn

Vragenlijst PRIMA studie 6 weken - AOFAS resterende gedeelte

Number	Question	Answers
2.1	Hoeveel beperkingen in het dagelijks leven ervaart u?	○ Geen beperkingen, geen hulpmiddelen nodig<br>○ Geen beperkingen in algemeen dagelijkse bezigheden, wel beperkingen in recreatieve activiteiten (sport, hobbies, etc) geen ondersteuning<br>○ Beperkingen bij dagelijkse- en recreatieve activiteiten, stok<br>○ Ernstige beperkingen bij dagelijkse- en recreatieactiviteiten, walker, krukken, rolstoel, brace

2.2	Wat is de afstand die u in één keer kunt lopen?	◯ Meer dan 600 meter<br>◯ 400 - 600 meter<br>◯ 100 - 300 meter<br>◯ Minder dan 100 meter
2.3	Heeft u moeite met lopen op een bepaalde ondergrond?	◯ Geen problemen bij lopen<br>◯ Enig problemen op ongelijke ondergrond, trap, helling, ladders<br>◯ Ernstige problemen bij ongelijke ondergrond, trap, helling, ladders
2.4	Hoe zou u uw looppatroon beoordelen?	◯ Normaal of minimaal afwijkend<br>◯ Duidelijk<br>◯ Ernstig
2.5	Hoe zou u de beweeglijkheid van uw voet beoordelen ten opzicht van de andere zijde of ten opzicte van vóór het ongeval?	◯ Hetzelfde of licht beperkt t.o.v. de niet aangedane zijde of zoals voor het ongeval<br>◯ Ongeveer de helft beperkt<br>◯ Sterk verminderd, bijna geen bewegingen mogelijk
2.6	Hoe zou u de beweeglijkheid van uw enkel beoordelen ten opzichte van de andere zijde of ten opzichte van voor het ongeval?	◯ Hetzelfde of licht beperkt t.o.v. de niet aangedane zijde of zoals voor het ongeval<br>◯ Ongeveer de helft beperkt<br>◯ Sterk verminderd, bijna geen bewegingen mogelijk
2.7	Hoe zou u de stabiliteit van uw voet en enkel beoordelen	◯ Stabiel, ik verzwik mijn enkel hooguit incidenteel<br>◯ Niet stabiel, ik verzwik mijn enkel vaak
2.8	Hoe zou u de stand van uw voet beoordelen ten opzichte van de andere zijde of ten opzichte van voor het ongeval?	◯ Goed, ik kan mijn voet goed plat op de grond zetten, waarbij de stand niet afwijkend is<br>◯ Ik sta vrijwel alleen op de buitenzijde van mijn voet<br>◯ Ik kan mijn voet niet plat op de grond zetten en loop vrijwel alleen op mijn voorvoet

Vragenlijst PRIMA studie 6 weken - VAS Pain score

Number	Question	Answers	
3.1	VAS pijnscore (Geef uw minimaal pijnscore aan tijdens dagelijkse activiteiten op een schaal van 0 tot 100, waarbij 0 geen pijn is en 100 de ergste pijn denkbaar is)	(0.00)	100 (100.00)

Vragenlijst PRIMA studie 6 weken - AAS

Number	Question	Answers

4.1	Wat is het meest bij u van toepassing ten aanzien van uw acitviteit?	☐ American Football<br>☐ Basketbal<br>☐ Gymnastiek/turnen<br>☐ Handbal<br>☐ Rugby<br>☐ Voetbal<br>☐ Hockey<br>☐ Korfbal<br>☐ Gevechtssporten: judo, karate, kung fu, taekwando, jiu jitsu, etc.<br>☐ Oriëntatielopen<br>☐ Rhytmische gymnastiek<br>☐ Volleybal<br>☐ Boxen<br>☐ Freestyle snowboarden<br>☐ Ijshockey<br>☐ Tennis<br>☐ Worstelen<br>☐ Fitness, aerobics<br>☐ Badminton<br>☐ Baseball<br>☐ Cross-country hardlopen<br>☐ Moderne pentathlon<br>☐ Squash<br>☐ Surfen, windsurfen<br>☐ Tafel tennis<br>☐ Atletiek: spring-, werponderdelen<br>☐ Waterskieën<br>☐ Dans<br>☐ Schermen<br>☐ Zaalhockey<br>☐ Bergbeklimmen<br>☐ Langlauf<br>☐ Parachute springen<br>☐ Softball<br>☐ Speciale beroepen en werkactiviteiten; speciale beroepen: ballet, professioneel soldaat, speciale reddingswerker, stuntman, etc.<br>☐ Duiken<br>☐ Scubaduiken<br>☐ Skaten, in-line skaten<br>☐ Atletiek: looponderdelen<br>☐ Triatlon<br>☐ Gewichtsheffen, body-building

		☐ Alle competitieve sporten hieronder met 'seasonal' conditioning<br>☐ Zwaar fysiek werk<br>☐ Alpine skiën en snowboarden<br>☐ Bowlen/curlen<br>☐ Golf<br>☐ Mountainbike/BMX<br>☐ Powerliften<br>☐ Zeilen<br>☐ Fysiek werk<br>☐ Wielrennen<br>☐ Paardrijden<br>☐ Motorsporten/technische sporten<br>☐ Roeien, kayakken<br>☐ Boogschieten<br>☐ Water polo en zwemmen<br>☐ Kunnen lopen op oneven grond<br>☐ Geen sport, echter geen limitatie in dagelijkse activiteiten<br>☐ Kunnen lopen op even grond, maar de dagelijkste activiteiten zijn gelimiteerd<br>☐ Niet kunnen lopen, fysieke handicap wegens enkelproblemen
4.1.1	***If 'Wat is het meest bij u van toepassing ten aanzien van uw acitviteit?' is equal to 'American Football' answer this question:***<br>American Football	○ Professioneel<br>○ Competitief<br>○ Recreatief
4.1.2	***If 'Wat is het meest bij u van toepassing ten aanzien van uw acitviteit?' is equal to 'Basketbal' answer this question:***<br>Basketbal	○ Professioneel<br>○ Competitief<br>○ Recreatief
4.1.3	***If 'Wat is het meest bij u van toepassing ten aanzien van uw acitviteit?' is equal to 'Gymnastiek/turnen' answer this question:***<br>Gymnastiek/turnen	○ Professioneel<br>○ Competitief<br>○ Recreatief
4.1.4	***If 'Wat is het meest bij u van toepassing ten aanzien van uw acitviteit?' is equal to 'Handbal' answer this question:***<br>Handbal	○ Professioneel<br>○ Competitief<br>○ Recreatief
4.1.5	***If 'Wat is het meest bij u van toepassing ten aanzien van uw acitviteit?' is equal to 'Rugby' answer this question:***<br>Rugby	○ Professioneel<br>○ Competitief<br>○ Recreatief

4.1.6	***If 'Wat is het meest bij u van toepassing ten aanzien van uw acitviteit?' is equal to 'Voetbal' answer this question:***<br>Voetbal	◯ Professioneel<br>◯ Competitief<br>◯ Recreatief
4.1.7	***If 'Wat is het meest bij u van toepassing ten aanzien van uw acitviteit?' is equal to 'Hockey' answer this question:***<br>Hockey	◯ Professioneel<br>◯ Competitief<br>◯ Recreatief
4.1.8	***If 'Wat is het meest bij u van toepassing ten aanzien van uw acitviteit?' is equal to 'Korfbal' answer this question:***<br>Korfbal	◯ Professioneel<br>◯ Competitief<br>◯ Recreatief
4.1.9	***If 'Wat is het meest bij u van toepassing ten aanzien van uw acitviteit?' is equal to 'Gevechtssporten: judo, karate, kung fu, taekwando, jiu jitsu, etc.' answer this question:***<br>Gevechtssporten: judo, karate, kung fu, taekwando, jiu jitsu, etc.	◯ Professioneel<br>◯ Competitief<br>◯ Recreatief
4.1.10	***If 'Wat is het meest bij u van toepassing ten aanzien van uw acitviteit?' is equal to 'Oriëntatielopen' answer this question:***<br>Oriëntatielopen	◯ Professioneel<br>◯ Competitief<br>◯ Recreatief
4.1.11	***If 'Wat is het meest bij u van toepassing ten aanzien van uw acitviteit?' is equal to 'Rhytmische gymnastiek' answer this question:***<br>Rhytmisch gymnastiek	◯ Professioneel<br>◯ Competitief<br>◯ Recreatief
4.1.12	***If 'Wat is het meest bij u van toepassing ten aanzien van uw acitviteit?' is equal to 'Volleybal' answer this question:***<br>Volleybal	◯ Professioneel<br>◯ Competitief<br>◯ Recreatief
4.1.13	***If 'Wat is het meest bij u van toepassing ten aanzien van uw acitviteit?' is equal to 'Boxen' answer this question:***<br>Boxen	◯ Professioneel<br>◯ Competitief<br>◯ Recreatief
4.1.14	***If 'Wat is het meest bij u van toepassing ten aanzien van uw acitviteit?' is equal to 'Freestyle snowboarden' answer this question:***<br>Freestyle snowboarden	◯ Professioneel<br>◯ Competitief<br>◯ Recreatief
4.1.15	***If 'Wat is het meest bij u van toepassing ten aanzien van uw acitviteit?' is equal to 'Freestyle snowboarden' answer this question:***<br>IJshockey	◯ Professioneel<br>◯ Competitief<br>◯ Recreatief

4.1.16	***If 'Wat is het meest bij u van toepassing ten aanzien van uw acitviteit?' is equal to 'Tennis' answer this question:***<br>Tennis	◯ Professioneel<br>◯ Competitief<br>◯ Recreatief
4.1.17	***If 'Wat is het meest bij u van toepassing ten aanzien van uw acitviteit?' is equal to 'Worstelen' answer this question:***<br>Worstelen	◯ Professioneel<br>◯ Competitief<br>◯ Recreatief
4.1.18	***If 'Wat is het meest bij u van toepassing ten aanzien van uw acitviteit?' is equal to 'Fitness, aerobics' answer this question:***<br>Fitness, aerobics	◯ Professioneel<br>◯ Competitief<br>◯ Recreatief
4.1.19	***If 'Wat is het meest bij u van toepassing ten aanzien van uw acitviteit?' is equal to 'Badminton' answer this question:***<br>Badminton	◯ Professioneel<br>◯ Competitief<br>◯ Recreatief
4.1.20	***If 'Wat is het meest bij u van toepassing ten aanzien van uw acitviteit?' is equal to 'Baseball' answer this question:***<br>Baseball	◯ Professioneel<br>◯ Competitief<br>◯ Recreatief
4.1.21	***If 'Wat is het meest bij u van toepassing ten aanzien van uw acitviteit?' is equal to 'Cross-country hardlopen' answer this question:***<br>Cross-country hardlopen	◯ Professioneel<br>◯ Competitief<br>◯ Recreatief
4.1.22	***If 'Wat is het meest bij u van toepassing ten aanzien van uw acitviteit?' is equal to 'Moderne pentathlon' answer this question:***<br>Moderne pentathlon	◯ Professioneel<br>◯ Competitief<br>◯ Recreatief
4.1.23	***If 'Wat is het meest bij u van toepassing ten aanzien van uw acitviteit?' is equal to 'Squash' answer this question:***<br>Squash	◯ Professioneel<br>◯ Competitief<br>◯ Recreatief
4.1.24	***If 'Wat is het meest bij u van toepassing ten aanzien van uw acitviteit?' is equal to 'Surfen, windsurfen' answer this question:***<br>Surfen, windsurfen	◯ Professioneel<br>◯ Competitief<br>◯ Recreatief
4.1.25	***If 'Wat is het meest bij u van toepassing ten aanzien van uw acitviteit?' is equal to 'Tafel tennis' answer this question:***<br>Tafel tennis	◯ Professioneel<br>◯ Competitief<br>◯ Recreatief

4.1.26	***If 'Wat is het meest bij u van toepassing ten aanzien van uw acitviteit?' is equal to 'Atletiek: spring-, werponderdelen' answer this question:***<br>Atletiek: spring-, werponderdelen	◯ Professioneel<br>◯ Competitief<br>◯ Recreatief
4.1.27	***If 'Wat is het meest bij u van toepassing ten aanzien van uw acitviteit?' is equal to 'Waterskieën' answer this question:***<br>Waterskien	◯ Professioneel<br>◯ Competitief<br>◯ Recreatief
4.1.28	***If 'Wat is het meest bij u van toepassing ten aanzien van uw acitviteit?' is equal to 'Dans' answer this question:***<br>Dans	◯ Professioneel<br>◯ Competitief<br>◯ Recreatief
4.1.29	***If 'Wat is het meest bij u van toepassing ten aanzien van uw acitviteit?' is equal to 'Schermen' answer this question:***<br>Schermen	◯ Professioneel<br>◯ Competitief<br>◯ Recreatief
4.1.30	***If 'Wat is het meest bij u van toepassing ten aanzien van uw acitviteit?' is equal to 'Zaalhockey' answer this question:***<br>Zaalhockey	◯ Professioneel<br>◯ Competitief<br>◯ Recreatief
4.1.31	***If 'Wat is het meest bij u van toepassing ten aanzien van uw acitviteit?' is equal to 'Bergbeklimmen' answer this question:***<br>Bergbeklimmen	◯ Professioneel<br>◯ Competitief<br>◯ Recreatief
4.1.32	***If 'Wat is het meest bij u van toepassing ten aanzien van uw acitviteit?' is equal to 'Langlauf' answer this question:***<br>Langlauf	◯ Professioneel<br>◯ Competitief<br>◯ Recreatief
4.1.33	***If 'Wat is het meest bij u van toepassing ten aanzien van uw acitviteit?' is equal to 'Parachute springen' answer this question:***<br>Parachute springen	◯ Professioneel<br>◯ Competitief<br>◯ Recreatief
4.1.34	***If 'Wat is het meest bij u van toepassing ten aanzien van uw acitviteit?' is equal to 'Softball' answer this question:***<br>Softball	◯ Professioneel<br>◯ Competitief<br>◯ Recreatief

4.1.35	***If 'Wat is het meest bij u van toepassing ten aanzien van uw acitviteit?' is equal to 'Speciale beroepen en werkactiviteiten; speciale beroepen: ballet, professioneel soldaat, speciale reddingswerker, stuntman, etc.' answer this question:***<br>Speciale beroepen en werkactiviteiten speciale beroepen: ballet, professioneel soldaat, speciale reddingswerker, stuntman, etc.	◯ ballet, professioneel soldaat, speciale reddingswerker, stuntman, etc.
4.1.36	***If 'Wat is het meest bij u van toepassing ten aanzien van uw acitviteit?' is equal to 'Duiken' answer this question:***<br>Duiken	◯ Professioneel/competitief<br>◯ Recreatief
4.1.37	***If 'Wat is het meest bij u van toepassing ten aanzien van uw acitviteit?' is equal to 'Scubaduiken' answer this question:***<br>Scubaduiken	◯ Professioneel/competitief<br>◯ Recreatief
4.1.38	***If 'Wat is het meest bij u van toepassing ten aanzien van uw acitviteit?' is equal to 'Skaten, in-line skaten' answer this question:***<br>skaten, in-linen skaten	◯ Professioneel/competitief<br>◯ Recreatief
4.1.39	***If 'Wat is het meest bij u van toepassing ten aanzien van uw acitviteit?' is equal to 'Atletiek: looponderdelen' answer this question:***<br>Atletiek: looponderdelen	◯ Professioneel/competitief<br>◯ Recreatief
4.1.40	***If 'Wat is het meest bij u van toepassing ten aanzien van uw acitviteit?' is equal to 'Triatlon' answer this question:***<br>triatlon	◯ Professioneel/competitief<br>◯ Recreatief
4.1.41	***If 'Wat is het meest bij u van toepassing ten aanzien van uw acitviteit?' is equal to 'Gewichtsheffen, body-building' answer this question:***<br>Gewichtsheffen, body-building	◯ Professioneel/competitief<br>◯ Recreatief
4.1.42	***If 'Wat is het meest bij u van toepassing ten aanzien van uw acitviteit?' is equal to 'Alle competitieve sporten hieronder met ‘seasonal’ conditioning' answer this question:***<br>Alle competitieve sporten hieronder met ‘seasonal’ conditioning	◯ Alle competitieve sporten hieronder met ‘seasonal’ conditioning
4.1.43	***If 'Wat is het meest bij u van toepassing ten aanzien van uw acitviteit?' is equal to 'Zwaar fysiek werk' answer this question:***<br>Zwaar fysiek werk	◯ Alle competitieve sporten hieronder met ‘seasonal’ conditioning

4.1.44	***If 'Wat is het meest bij u van toepassing ten aanzien van uw acitviteit?' is equal to 'Alpine skiën en snowboarden' answer this question:***<br>Alpine skien en snowboarden	○ Alpine skien en snowboarden
4.1.45	***If 'Wat is het meest bij u van toepassing ten aanzien van uw acitviteit?' is equal to 'Bowlen/curlen' answer this question:***<br>Bowlen/curlen	○ Bowlen/curlen
4.1.46	***If 'Wat is het meest bij u van toepassing ten aanzien van uw acitviteit?' is equal to 'Golf' answer this question:***<br>Golf	○ Golf
4.1.47	***If 'Wat is het meest bij u van toepassing ten aanzien van uw acitviteit?' is equal to 'Mountainbike/BMX' answer this question:***<br>Mountainbike/BMX	○ Mountainbike/BMX
4.1.48	***If 'Wat is het meest bij u van toepassing ten aanzien van uw acitviteit?' is equal to 'Powerliften' answer this question:***<br>Powerliften	○ Powerliften
4.1.49	***If 'Wat is het meest bij u van toepassing ten aanzien van uw acitviteit?' is equal to 'Zeilen' answer this question:***<br>Zeilen	○ Zeilen
4.1.50	***If 'Wat is het meest bij u van toepassing ten aanzien van uw acitviteit?' is equal to 'Fysiek werk' answer this question:***<br>Fysiek werk	○ Fysiek werk
4.1.51	***If 'Wat is het meest bij u van toepassing ten aanzien van uw acitviteit?' is equal to 'Wielrennen' answer this question:***<br>Wielrennen	○ Wielrennen
4.1.52	***If 'Wat is het meest bij u van toepassing ten aanzien van uw acitviteit?' is equal to 'Paardrijden' answer this question:***<br>Paardrijden	○ Paardrijden
4.1.53	***If 'Wat is het meest bij u van toepassing ten aanzien van uw acitviteit?' is equal to 'Motorsporten/technische sporten' answer this question:***<br>Motorsporten/technische sporten	○ Motorsporten/technische sporten
4.1.54	***If 'Wat is het meest bij u van toepassing ten aanzien van uw acitviteit?' is equal to 'Roeien, kayakken' answer this question:***<br>Roeien, kayakken	○ Roeien, kayakken

4.1.55	***If 'Wat is het meest bij u van toepassing ten aanzien van uw acitviteit?' is equal to 'Boogschieten' answer this question:*** Boogschieten	◯ Boogschieten
4.1.56	***If 'Wat is het meest bij u van toepassing ten aanzien van uw acitviteit?' is equal to 'Water polo en zwemmen' answer this question:*** Water polo en zwemmen	◯ Water polo en zwemmen
4.1.57	***If 'Wat is het meest bij u van toepassing ten aanzien van uw acitviteit?' is equal to 'Kunnen lopen op oneven grond' answer this question:*** Kunnen lopen op oneven grond	◯ Kunnen lopen op oneven grond
4.1.58	***If 'Wat is het meest bij u van toepassing ten aanzien van uw acitviteit?' is equal to 'Geen sport, echter geen limitatie in dagelijkse activiteiten' answer this question:*** Geen sport, echter geen limitatie in dagelijkse activiteiten	◯ Geen sport, echter geen limitatie in dagelijkse activiteiten
4.1.59	***If 'Wat is het meest bij u van toepassing ten aanzien van uw acitviteit?' is equal to 'Kunnen lopen op even grond, maar de dagelijkste activiteiten zijn gelimiteerd' answer this question:*** Kunnen lopen op even grond, maar de dagelijkste activiteiten zijn gelimiteerd	◯ Kunnen lopen op even grond, maar de dagelijkste activiteiten zijn gelimiteerd
4.1.60	***If 'Wat is het meest bij u van toepassing ten aanzien van uw acitviteit?' is equal to 'Niet kunnen lopen, fysieke handicap wegens enkelproblemen' answer this question:*** Niet kunnen lopen, fysieke handicap wegens enkelproblemen	◯ Niet kunnen lopen, fysieke handicap wegens enkelproblemen

Vragenlijst PRIMA studie 6 weken - Hoe tevreden bent u over de enkelklachten?

Number	Question	Answers
5.1	Hoe tevreden bent u over de enkelklachten?	◯ Slecht<br>◯ Matig<br>◯ Goed<br>◯ Uitstekend

Vragenlijst PRIMA studie 6 weken - SF-36

Number	Question	Answers
	Deze vragenlijst gaat over uw standpunten t.a.v. uw gezondheid. Met behulp van deze gegevens kan worden bijgehouden hoe u zich voelt en hoe goed u in staat bent uw gebruikelijke bezigheden uit te voeren. Beantwoord elke vraag door een antwoord aan te klikken. Als u niet zeker weet hoe u een vraag moet beantwoorden, geef dan het best mogelijke antwoord.	
6.1	Hoe zou u over het algemeen uw gezondheid noemen?	◯ Uitstekend ◯ Zeer goed ◯ Goed ◯ Matig ◯ Slecht
6.2	Hoe beoordeelt u nu uw gezondheid over het algemeen vergeleken met een jaar geleden?	◯ Veel beter dan een jaar geleden ◯ Wat beter dan een jaar geleden ◯ Ongeveer hetzelfde als een jaar geleden ◯ Wat slechter dan een jaar geleden ◯ Veel slechter dan een jaar geleden
6.3	Wordt u door uw gezondheid op dit moment beperkt bij forse inspanning, zoals hardlopen, tillen van zware voorwerpen of een veeleisende sport beoefenen?	◯ Ja, ernstig beperkt ◯ Ja, een beetje beperkt ◯ Nee, helemaal niet beperkt
6.4	Wordt u door uw gezondheid op dit moment beperkt bij matige inspanning zoals een tafel verplaatsen, stofzuigen, zwemmen of fietsen?	◯ Ja, ernstig beperkt ◯ Ja, een beetje beperkt ◯ Nee, helemaal niet beperkt
6.5	Wordt u door uw gezondheid op dit moment beperkt bij boodschappen tillen of dragen?	◯ Ja, ernstig beperkt ◯ Ja, een beetje beperkt ◯ Nee, helemaal niet beperkt
6.6	Wordt u door uw gezondheid op dit moment beperkt bij een paar trappen oplopen?	◯ Ja, ernstig beperkt ◯ Ja, een beetje beperkt ◯ Nee, helemaal niet beperkt
6.7	Wordt u door uw gezondheid op dit moment beperkt bij één trap oplopen?	◯ Ja, ernstig beperkt ◯ Ja, een beetje beperkt ◯ Nee, helemaal niet beperkt
6.8	Wordt u door uw gezondheid op dit moment beperkt bij bukken, knielen of hurken?	◯ Ja, ernstig beperkt ◯ Ja, een beetje beperkt ◯ Nee, helemaal niet beperkt
6.9	Wordt u door uw gezondheid op dit moment beperkt bij meer dan één kilometer lopen?	◯ Ja, ernstig beperkt ◯ Ja, een beetje beperkt ◯ Nee, helemaal niet beperkt
6.10	Wordt u door uw gezondheid op dit moment beperkt bij een paar honderd meter lopen?	◯ Ja, ernstig beperkt ◯ Ja, een beetje beperkt ◯ Nee, helemaal niet beperkt

6.11	Wordt u door uw gezondheid op dit moment beperkt bij ongeveer honderd meter lopen?	◯ Ja, ernstig beperkt ◯ Ja, een beetje beperkt ◯ Nee, helemaal niet beperkt
6.12	Wordt u door uw gezondheid op dit moment beperkt bij uzelf wassen of aankleden?	◯ Ja, ernstig beperkt ◯ Ja, een beetje beperkt ◯ Nee, helemaal niet beperkt
6.13	U besteedde in de afgelopen 4 weken minder tijd aan werk of andere bezigheden	◯ Ja ◯ Nee
6.14	U heeft in de afgelopen 4 weken minder bereikt dan u zou willen	◯ Ja ◯ Nee
6.15	U was in de afgelopen 4 weken beperkt in het soort werk of andere bezigheden.	◯ Ja ◯ Nee
6.16	U had de afgelopen 4 weken moeite om uw werk of andere bezigheden uit te voeren (het kostte u bijvoorbeeld extra inspanning).	◯ Ja ◯ Nee
6.17	U besteedde in de afgelopen 4 weken minder tijd aan werk of andere bezigheden ten gevolge van emotionele problemen	◯ Ja ◯ Nee
6.18	U heeft in de afgelopen 4 weken minder bereikt dan u zou willen ten gevolge van emotionele problemen.	◯ Ja ◯ Nee
6.19	U deed de afgelopen 4 weken uw werk of andere bezigheden niet zo zorgvuldig als gewoonlijk ten gevolge van emotionele problemen.	◯ Ja ◯ Nee
6.20	In hoeverre hebben uw lichamelijke gezondheid of emotionele problemen u gedurende de afgelopen 4 weken gehinderd in uw normale omgang met familie, vrienden of buren, of bij activiteiten in groepsverband?	◯ Helemaal niet ◯ Enigszins ◯ Nogal ◯ Veel ◯ Heel erg veel
6.21	Hoeveel lichamelijke pijn heeft u de afgelopen 4 weken gehad?	◯ Geen<br>◯ Heel licht<br>◯ Licht<br>◯ Nogal<br>◯ Ernstig<br>◯ Heel ernstig
6.22	In welke mate bent u de afgelopen 4 weken door de pijn gehinderd in uw normale werk (zowel werk buitenshuis als huishoudelijk werk)?	◯ Helemaal niet<br>◯ Een klein beetje<br>◯ Nogal<br>◯ Veel<br>◯ Heel erg veel

6.23	Voelde u zich levenslustig?	○ Altijd<br>○ Meestal<br>○ Vaak<br>○ Soms<br>○ Zelden<br>○ Nooit
6.24	Was u erg zenuwachtig?	○ Altijd<br>○ Meestal<br>○ Vaak<br>○ Soms<br>○ Zelden<br>○ Nooit
6.25	Zat u zo in de put dat u niets kon?	○ Altijd<br>○ Meestal<br>○ Vaak<br>○ Soms<br>○ Zelden<br>○ Nooit
6.26	Voelde u zich rustig en tevreden?	○ Altijd<br>○ Meestal<br>○ Vaak<br>○ Soms<br>○ Zelden<br>○ Nooit
6.27	Had u veel energie?	○ Altijd<br>○ Meestal<br>○ Vaak<br>○ Soms<br>○ Zelden<br>○ Nooit
6.28	Voelde u zich somber en neerslachtig?	○ Altijd<br>○ Meestal<br>○ Vaak<br>○ Soms<br>○ Zelden<br>○ Nooit

6.29	Voelde u zich uitgeput?	◯ Altijd<br>◯ Meestal<br>◯ Vaak<br>◯ Soms<br>◯ Zelden<br>◯ Nooit
6.30	Was u een gelukkig mens?	◯ Altijd<br>◯ Meestal<br>◯ Vaak<br>◯ Soms<br>◯ Zelden<br>◯ Nooit
6.31	Voelde u zich moe?	◯ Altijd<br>◯ Meestal<br>◯ Vaak<br>◯ Soms<br>◯ Zelden<br>◯ Nooit
6.32	Hoe vaak hebben uw lichamelijke gezondheid of emotionele problemen u gedurende de afgelopen 4 weken gehinderd bij uw sociale activiteiten (zoals vrienden of familie bezoeken etc.)?	◯ Altijd<br>◯ Meestal<br>◯ Soms<br>◯ Zelden<br>◯ Nooit
6.33	Ik lijk wat gemakkelijker ziek te worden dan andere mensen	◯ Volkomen juist<br>◯ Grotendeels juist<br>◯ Weet ik niet<br>◯ Grotendeels onjuist<br>◯ Volkomen onjuist
6.34	Ik ben even gezond als andere mensen die ik ken	◯ Volkomen juist<br>◯ Grotendeels juist<br>◯ Weet ik niet<br>◯ Grotendeels onjuist<br>◯ Volkomen onjuist

6.35	Ik verwacht dat mijn gezondheid achteruit zal gaan	◯ Volkomen juist<br>◯ Grotendeels juist<br>◯ Weet ik niet<br>◯ Grotendeels onjuist<br>◯ Volkomen onjuist
6.36	Mijn gezondheid is uitstekend	◯ Volkomen juist<br>◯ Grotendeels juist<br>◯ Weet ik niet<br>◯ Grotendeels onjuist<br>◯ Volkomen onjuist

Vragenlijst PRIMA studie 6 weken - GAS

Number	Question	Answers
	Tijdens de eerste afspraak werd een duidelijk doel met u afgesproken. Dit werd tevens per email naar u gestuurd. Hoe is de situatie nu ten aanzien van de destijds gestelde doelstelling?	
7.1	Wat is nu de situatie ten aanzien van het tijdens de eerste afspraak afgesproken doelstelling (Goal attainment Scaling)?	◯ Achteruitgang (minder dan de utigangssituatie)<br>◯ Uitgangssituatie<br>◯ Minder dan het doel<br>◯ Doel<br>◯ Meer dan het doel<br>◯ Veel meer dan het doel

Vragenlijst PRIMA studie 6 weken - EQ-5D-3L

Number	Question	Answers
	Deze vragenlijst gaat over uw standpunten t.a.v. uw gezondheid. Met behulp van deze gegevens kan worden bijgehouden hoe u zich voelt en hoe goed u in staat bent uw gebruikelijke bezigheden uit te voeren. Beantwoord elke vraag door een antwoord aan te klikken. Als u niet zeker weet hoe u een vraag moet beantwoorden, geef dan het best mogelijke antwoord.	
8.1	Hoe is het met uw Mobiliteit gesteld?	◯ Ik heb geen problemen met lopen<br>◯ Ik heb enige problemen met lopen<br>◯ Ik ben bedlegerig

8.2	Hoe is het met uw Zelfzorg gesteld?	◯ Ik heb geen problemen om mijzelf te wassen of aan te kleden<br>◯ Ik heb enige problemen om mijzelf te wassen of aan te kleden<br>◯ Ik ben niet in staat om mijzelf te wassen of aan te kleden
8.3	Hoe is het met de Dagelijkse activiteiten (werk, studie, huishouden, gezins- en vrijetijdsactiviteiten) gesteld?	◯ Ik heb geen problemen met mijn dagelijkse activiteiten<br>◯ Ik heb enige problemen met mijn dagelijkse activiteiten<br>◯ Ik ben niet in staat om mijn dagelijkse activiteiten uit te voeren
8.4	Hoe is het met de Pijn/klachten gesteld?	◯ Ik heb geen pijn of andere klachten<br>◯ Ik heb matige pijn of andere klachten<br>◯ Ik heb zeer ernstige pijn of andere klachten
8.5	Hoe is het met de Stemming gesteld?	◯ Ik ben niet angstig of somber<br>◯ Ik ben matig angstig of somber<br>◯ Ik ben erg angstig of somber
	We willen weten hoe goed of slecht uw gezondheid VANDAAG is. Deze meetschaal loopt van 0 tot 100: 100 staat voor de BESTE gezondheid die u zich kunt voorstellen - 0 staat voor de SLECHTSTE gezondheid die u zich kunt voorstellen	
8.6	Uw Gezondheid vandaag	(0.00) (100.00)

Vragenlijst PRIMA studie 6 weken - AOS

Number	Question	Answers
	Instructies: De lijn naast elke vraag staat voor hoeveel PIJN u heeft in verschillende situaties. De linker kant (0) is “geen pijn” en de rechter kant (100) is “ergste pijn denkbaar”. Geef voor de onderstaande situaties op de lijn aan hoeveel PIJN u in de afgelopen week in de enkel had. Als een of meerdere van deze situaties niet van toepassing waren, dan kiest u de "niet van toepassing" optie.	
9.1	Wat was de hoogte van de ergste pijn in de afgelopen week?	(0.00) (100.00)
9.2	Heeft u pijn als u 's ochtends opstaat?	◯ Ja<br>◯ Nee<br>◯ Niet van toepassing

9.2.1	***If 'Heeft u pijn als u 's ochtends opstaat?' is equal to 'Ja' answer this question:***<br>Hoeveel pijn heeft u voordat u 's ochtends opstaat?	(0.00)	(100.00)
9.3	Heeft u pijn wanneer u op blote voeten loopt?	◯ Ja<br>◯ Nee<br>◯ Niet van toepassing	
9.3.1	***If 'Heeft u pijn wanneer u op blote voeten loopt?' is equal to 'Ja' answer this question:***<br>Hoeveel pijn heeft u als u op blote voeten loopt?	(0.00)	(100.00)
9.4	Heeft u pijn wanneer u op blote voeten staat?	◯ Ja<br>◯ Nee<br>◯ Niet van toepassing	
9.4.1	***If 'Heeft u pijn wanneer u op blote voeten staat?' is equal to 'Ja' answer this question:***<br>Hoeveel pijn heeft u als u op blote voeten staat?	(0.00)	(100.00)
9.5	Heeft u pijn wanneer u met schoenen loopt?	◯ Ja<br>◯ Nee<br>◯ Niet van toepassing	
9.5.1	***If 'Heeft u pijn wanneer u met schoenen loopt?' is equal to 'Ja' answer this question:***<br>Hoeveel pijn heeft u wanneer u met schoenen loopt?	(0.00)	(100.00)
9.6	Heeft u pijn wanneer u met schoenen staat?	◯ Ja<br>◯ Nee<br>◯ Niet van toepassing	
9.6.1	***If 'Heeft u pijn wanneer u met schoenen staat?' is equal to 'Ja' answer this question:***<br>Hoeveel pijn heeft u wanneer u met schoenen staat?	(0.00)	(100.00)
9.7	Heeft u pijn wanneer u loopt met steunzolen of een brace?	◯ Ja<br>◯ Nee<br>◯ Niet van toepassing	
9.7.1	***If 'Heeft u pijn wanneer u loopt met steunzolen of een brace?' is equal to 'Ja' answer this question:***<br>Hoeveel pijn heeft u wanneer u loopt met steunzolen of een brace?	(0.00)	(100.00)

9.8	Heeft u pijn wanneer u staat met steunzolen of een brace?	◯ Ja<br>◯ Nee<br>◯ Niet van toepassing	
9.8.1	***If 'Heeft u pijn wanneer u staat met steunzolen of een brace?' is equal to 'Ja' answer this question:***<br>Hoeveel pijn heeft u wanneer u staat met steunzolen of een brace?	(0.00)	(100.00)
9.9	Heeft u pijn aan het einde van de dag?	◯ Ja<br>◯ Nee<br>◯ Niet van toepassing	
9.9.1	***If 'Heeft u pijn aan het einde van de dag?' is equal to 'Ja' answer this question:***<br>Hoeveel pijn heeft u aan het einde van de dag?	(0.00)	(100.00)
	Instructies:De lijn naast elke vraag staat voor hoeveel MOEITE u heeft met verschillende activiteiten. De linker kant (0) is “Niet moeilijk” en de rechter kant (100) is “Te moeilijk, niet uitvoerbaar”. Geef voor de onderstaande activiteiten op de lijn aan hoeveel MOEITE u in de afgelopen week door enkelklachten had met onderstaande activiteiten. Als een situatie niet van toepassing is, kies dan de optie "niet van toepassing".		
9.10	Heeft u moeite met door het huis lopen?	◯ Ja<br>◯ Nee<br>◯ Niet van toepassing	
9.10.1	***If 'Heeft u moeite met door het huis lopen?' is equal to 'Ja' answer this question:***<br>Hoeveel moeite heeft u met door het huis lopen?	(0.00)	(100.00)
9.11	Heeft u moeite met buiten lopen op oneven ondergrond?	◯ Ja<br>◯ Nee<br>◯ Niet van toepassing	
9.11.1	***If 'Heeft u moeite met buiten lopen op oneven ondergrond?' is equal to 'Ja' answer this question:***<br>Hoeveel moeite heeft u met buiten lopen op oneven ondergrond?	(0.00)	(100.00)
9.12	Heeft u moeite met een paar honderd meter lopen?	◯ Ja<br>◯ Nee<br>◯ Niet van toepassing	
9.12.1	***If 'Heeft u moeite met een paar honderd meter lopen?' is equal to 'Ja' answer this question:***<br>Hoeveel moeite heeft u met een paar honderd meter lopen?	(0.00)	(100.00)

9.13	Heeft u moeite met een trap oplopen?	◯ Ja<br>◯ Nee<br>◯ Niet van toepassing	
9.13.1	***If 'Heeft u moeite met een trap oplopen?' is equal to 'Ja' answer this question:***<br>Hoeveel moeite heeft u met een trap oplopen?	(0.00)	(100.00)
9.14	Heeft u moeite met een trap aflopen?	◯ Ja<br>◯ Nee<br>◯ Niet van toepassing	
9.14.1	***If 'Heeft u moeite met een trap aflopen?' is equal to 'Ja' answer this question:***<br>Hoeveel moeite heeft u met een trap aflopen?	(0.00)	(100.00)
9.15	Heeft u moeite met op de tenen staan?	◯ Ja<br>◯ Nee<br>◯ Niet van toepassing	
9.15.1	***If 'Heeft u moeite met op de tenen staan?' is equal to 'Ja' answer this question:***<br>Hoeveel moeite heeft u met op de tenen staan?	(0.00)	(100.00)
9.16	Heeft u moeite met opstaan uit de stoel?	◯ Ja<br>◯ Nee<br>◯ Niet van toepassing	
9.16.1	***If 'Heeft u moeite met opstaan uit de stoel?' is equal to 'Ja' answer this question:***<br>Hoeveel moeite heeft u met opstaan uit de stoel?	(0.00)	(100.00)
9.17	Heeft u moeite met het op- of afstappen van stoepranden?	◯ Ja<br>◯ Nee<br>◯ Niet van toepassing	
9.17.1	***If 'Heeft u moeite met het op- of afstappen van stoepranden?' is equal to 'Ja' answer this question:***<br>Hoeveel moeite heeft u met het op- of afstappen van stoepranden?	(0.00)	(100.00)
9.18	Heeft u moeite met snel lopen of rennen?	◯ Ja<br>◯ Nee<br>◯ Niet van toepassing	

9.18.1	***If 'Heeft u moeite met snel lopen of rennen?' is equal to 'Ja' answer this question:***<br>Hoeveel moeite heeft u met snel lopen of rennen?	(0.00)	(100.00)

Vragenlijst PRIMA studie 6 weken - FAOS

Number	Question	Answers
	Deze lijst vraagt naar uw mening over uw voet/enkel. Uw antwoorden geven ons een beeld van uw voet/enkel klachten en hoe u in staat bent om alledaagse activiteiten uit te voeren in uw huidige situatie. Beantwoorden van een vraag doet u door het aanklikken van een vakje met het volgens u meest juiste antwoord (één vakje per vraag). Als u niet zeker weet hoe u een vraag moet beantwoorden, geeft u dan het antwoord dat volgens u het meest op uw situatie van toepassing is. Deze vraag heeft betrekking op het voorkomen van voet/enkel klachten in de afgelopen week.	
10.1	Is uw voet/enkel gezwollen?	○ Nooit ○ Zelden ○ Soms ○ Vaak ○ Altijd
10.2	Voelt u gekraak of hoort u klikken of een ander vreemd geluid wanneer u de voet/enkel beweegt?	○ Nooit ○ Zelden ○ Soms ○ Vaak ○ Altijd
10.3	Hapert uw enkel of blokkeert uw enkel ('op slot' gaan zitten) wanneer u deze beweegt?	○ Nooit ○ Zelden ○ Soms ○ Vaak ○ Altijd
10.4	Kunt u de voet/enkel volledig strekken?	○ Altijd ○ Vaak ○ Soms ○ Zelden ○ Nooit
10.5	Kunt u de voet volledig naar u toe buigen?	○ Altijd ○ Vaak ○ Soms ○ Zelden ○ Nooit
10.6	In welke mate heeft u een stijf gevoel in de voet/enkel 's ochtends bij het wakker worden?	○ Niet ○ Mild ○ Matig ○ Ernstig ○ Zeer ernstig
10.7	In welke mate heeft u een stijf gevoel in de voet/enkel na zitten, liggen of rusten later op de dag?	○ Niet ○ Mild ○ Matig ○ Ernstig ○ Zeer ernstig
10.8	Hoe vaak heeft u pijn in uw voet/enkel?	○ Nooit ○ Maandelijks ○ Wekelijks ○ Dagelijks ○ Altijd

10.9	Hoeveel voet/enkel pijn heeft u gehad in de afgelopen week bij draaien als uw voet/enkel op de grond staat?	◯ Geen<br>◯ Mild<br>◯ Matig<br>◯ Ernstig<br>◯ Zeer ernstig
10.10	Hoeveel voet/enkel pijn heeft u gehad in de afgelopen week bij het volledig uitstrekken van de voet/enkel?	◯ Geen<br>◯ Mild<br>◯ Matig<br>◯ Ernstig<br>◯ Zeer ernstig
10.11	Hoeveel voet/enkel pijn heeft u gehad in de afgelopen week bij het volledig naar u toe buigen/optrekken van de voet/enkel	◯ Geen<br>◯ Mild<br>◯ Matig<br>◯ Ernstig<br>◯ Zeer ernstig
10.12	Hoeveel voet/enkel pijn heeft u gehad in de afgelopen week bij het lopen op een vlakke ondergrond	◯ Geen<br>◯ Mild<br>◯ Matig<br>◯ Ernstig<br>◯ Zeer ernstig
10.13	Hoeveel voet/enkel pijn heeft u gehad in de afgelopen week bij het trap op- en trap af lopen?	◯ Geen<br>◯ Mild<br>◯ Matig<br>◯ Ernstig<br>◯ Zeer ernstig
10.14	Hoeveel voet/enkel pijn heeft u gehad in de afgelopen week 's nachts in bed?	◯ Geen<br>◯ Mild<br>◯ Matig<br>◯ Ernstig<br>◯ Zeer ernstig
10.15	Hoeveel voet/enkel pijn heeft u gehad in de afgelopen week bij het zitten of liggen?	◯ Geen<br>◯ Mild<br>◯ Matig<br>◯ Ernstig<br>◯ Zeer ernstig

10.16	Hoeveel voet/enkel pijn heeft u gehad in de afgelopen week bij het rechtop staan?	◯ Geen<br>◯ Mild<br>◯ Matig<br>◯ Ernstig<br>◯ Zeer ernstig
10.17	In welke mate werd u gehinderd bij het trap aflopen?	◯ Niet ◯ Mild ◯ Matig ◯ Ernstig ◯ Zeer ernstig
10.18	In welke mate werd u gehinderd bij het trap op lopen?	◯ Niet ◯ Mild ◯ Matig ◯ Ernstig ◯ Zeer ernstig
10.19	In welke mate werd u gehinderd als u vanuit een zittende positie ging staan?	◯ Niet ◯ Mild ◯ Matig ◯ Ernstig ◯ Zeer ernstig
10.20	In welke mate werd u gehinderd bij het staan?	◯ Niet ◯ Mild ◯ Matig ◯ Ernstig ◯ Zeer ernstig
10.21	In welke mate werd u gehinderd bij het naar de grond buigen/iets oprapen?	◯ Niet ◯ Mild ◯ Matig ◯ Ernstig ◯ Zeer ernstig
10.22	In welke mate werd u gehinderd bij het lopen op een vlakke ondergrond?	◯ Niet ◯ Mild ◯ Matig ◯ Ernstig ◯ Zeer ernstig
10.23	In welke mate werd u gehinderd bij het in- en uit de auto stappen?	◯ Niet ◯ Mild ◯ Matig ◯ Ernstig ◯ Zeer ernstig
10.24	In welke mate werd u gehinderd bij het boodschappen doen?	◯ Niet ◯ Mild ◯ Matig ◯ Ernstig ◯ Zeer ernstig
10.25	In welke mate werd u gehinderd bij sokken/panty's aantrekken?	◯ Niet ◯ Mild ◯ Matig ◯ Ernstig ◯ Zeer ernstig
10.26	In welke mate werd u gehinderd bij het opstaan uit bed?	◯ Niet ◯ Mild ◯ Matig ◯ Ernstig ◯ Zeer ernstig
10.27	In welke mate werd u gehinderd bij het sokken uittrekken?	◯ Niet ◯ Mild ◯ Matig ◯ Ernstig ◯ Zeer ernstig
10.28	In welke mate werd u gehinderd bij het in bed liggen (omdraaien, lange tijd uw voet/enkel in dezelfde positie houden)?	◯ Niet ◯ Mild ◯ Matig ◯ Ernstig ◯ Zeer ernstig

10.29	In welke mate werd u gehinderd bij het in/uit bad stappen?	◯ Niet ◯ Mild ◯ Matig ◯ Ernstig ◯ Zeer ernstig
10.30	In welke mate werd u gehinderd bij zitten?	◯ Niet ◯ Mild ◯ Matig ◯ Ernstig ◯ Zeer ernstig
10.31	In welke mate werd u gehinderd bij het toilet op en af gaan?	◯ Niet ◯ Mild ◯ Matig ◯ Ernstig ◯ Zeer ernstig
10.32	In welke mate werd u gehinderd bij zwaar huishoudelijk werk (bijvoorbeeld zware dozen sjouwen, vloer schrobben)?	◯ Niet ◯ Mild ◯ Matig ◯ Ernstig ◯ Zeer ernstig
10.33	In welke mate werd u gehinderd bij licht huishoudeijk werk (bijvoorbeeld koken, afstoffen)?	◯ Niet ◯ Mild ◯ Matig ◯ Ernstig ◯ Zeer ernstig
10.34	In welke mate werd u gehinderd bij hurken?	◯ Niet ◯ Mild ◯ Matig ◯ Ernstig ◯ Zeer ernstig
10.35	In welke mate werd u gehinderd bij hardlopen?	◯ Niet ◯ Mild ◯ Matig ◯ Ernstig ◯ Zeer ernstig
10.36	In welke mate werd u gehinderd bij springen?	◯ Niet ◯ Mild ◯ Matig ◯ Ernstig ◯ Zeer ernstig
10.37	In welke mate werd u gehinderd bij ronddraaien op uw aangedane voet/enkel?	◯ Niet ◯ Mild ◯ Matig ◯ Ernstig ◯ Zeer ernstig
10.38	In welke mate werd u gehinderd bij knielen?	◯ Niet ◯ Mild ◯ Matig ◯ Ernstig ◯ Zeer ernstig
10.39	Hoe vaak bent u zich bewust van uw voet/enkel probleem?	◯ Nooit ◯ Maandelijks ◯ Wekelijks ◯ Dagelijks ◯ Altijd
10.40	Heeft u uw leven veranderd om activiteiten te vermijden die schadelijk kunnen zijn voor uw voet/enkel?	◯ Niet<br>◯ Enigszins<br>◯ Matig<br>◯ Behoorlijk<br>◯ Volledig

10.41	In hoeverre kunt u op uw voet/enkel vertrouwen?	◯ Volledig<br>◯ Behoorlijk<br>◯ Matig<br>◯ Enigzins<br>◯ Niet
10.42	In het algemeen, in welke mate ondervindt u hinder van uw voet/enkel	◯ Geen<br>◯ Mild<br>◯ Matig<br>◯ Ernstig<br>◯ Zeer ernstig

Vragenlijst PRIMA studie 6 weken - Dank

Number	Question	Answers
11.1	Hartelijk dank voor de tijd die u heeft genomen om de vragenlijsten in te vullen. Indien u nog opmerkingen heeft kunt u deze hierin plaatsen.	

STANDARD PROTOCOL ITEMS: RECOMMENDATIONS FOR INTERVENTIONAL TRIALS

SPIRIT 2013 Checklist: Recommended items to address in a clinical trial protocol and related documents*

Section/item	Item No	Description	Addressed on page number
**Administrative information**			
Title	1	Descriptive title identifying the study design, population, interventions, and, if applicable, trial acronym	Page 1, line 1-4
Trial registration	2a	Trial identifier and registry name. If not yet registered, name of intended registry	Page 2, line 64-65
	2b	All items from the World Health Organization Trial Registration Data Set	Page 20, line 416-417
Protocol version	3	Date and version identifier	Every page at the bottom
Funding	4	Sources and types of financial, material, and other support	Page 23, Line 493-498
Roles and responsibilities	5a	Names, affiliations, and roles of protocol contributors	Page 1,Line 5-28
	5b	Name and contact information for the trial sponsor	Page 1, Line 33-39
	5c	Role of study sponsor and funders, if any, in study design; collection, management, analysis, and interpretation of data; writing of the report; and the decision to submit the report for publication, including whether they will have ultimate authority over any of these activities	Page 23, Line 493-498

For peer review only

	5d	Composition, roles, and responsibilities of the coordinating centre, steering committee, endpoint adjudication committee, data management team, and other individuals or groups overseeing the trial, if applicable (see Item 21a for data monitoring committee)	Page 19, line 399-411
**Introduction**			
Background and rationale	6a	Description of research question and justification for undertaking the trial, including summary of relevant studies (published and unpublished) examining benefits and harms for each intervention	Page 5 line 89-121
	6b	Explanation for choice of comparators	Page 6, line 111-121 & page 7 line 125-128
Objectives	7	Specific objectives or hypotheses	Page 7, Line 124-128
Trial design	8	Description of trial design including type of trial (eg, parallel group, crossover, factorial, single group), allocation ratio, and framework (eg, superiority, equivalence, noninferiority, exploratory)	Page 7, line 130-132
**Methods: Participants, interventions, and outcomes**			
Study setting	9	Description of study settings (eg, community clinic, academic hospital) and list of countries where data will be collected. Reference to where list of study sites can be obtained	Page 8, line 164-165
Eligibility criteria	10	Inclusion and exclusion criteria for participants. If applicable, eligibility criteria for study centres and individuals who will perform the interventions (eg, surgeons, psychotherapists)	Page 8-9, line 168-188
Interventions	11a	Interventions for each group with sufficient detail to allow replication, including how and when they will be administered	Page 10-11, line 211-239
	11b	Criteria for discontinuing or modifying allocated interventions for a given trial participant (eg, drug dose change in response to harms, participant request, or improving/worsening disease)	Page 14, line 281-285

For peer review only - http://bmjopen.bmj.com/site/about/guidelines.xhtml

	11c	Strategies to improve adherence to intervention protocols, and any procedures for monitoring adherence (eg, drug tablet return, laboratory tests)	Page 15-16, line 329-335
	11d	Relevant concomitant care and interventions that are permitted or prohibited during the trial	Page 12, line 242-247
Outcomes	12	Primary, secondary, and other outcomes, including the specific measurement variable (eg, systolic blood pressure), analysis metric (eg, change from baseline, final value, time to event), method of aggregation (eg, median, proportion), and time point for each outcome. Explanation of the clinical relevance of chosen efficacy and harm outcomes is strongly recommended	Page 14-15, line 288-327
Participant timeline	13	Time schedule of enrolment, interventions (including any run-ins and washouts), assessments, and visits for participants. A schematic diagram is highly recommended (see Figure)	Page 13, table 1
Sample size	14	Estimated number of participants needed to achieve study objectives and how it was determined, including clinical and statistical assumptions supporting any sample size calculations	Page 17, line 344-353
Recruitment	15	Strategies for achieving adequate participant enrolment to reach target sample size	Page 12, line 256

Methods: Assignment of interventions (for controlled trials)

Allocation:

Sequence generation	16a	Method of generating the allocation sequence (eg, computer-generated random numbers), and list of any factors for stratification. To reduce predictability of a random sequence, details of any planned restriction (eg, blocking) should be provided in a separate document that is unavailable to those who enrol participants or assign interventions	Page 9-10. Line 190-205
Allocation concealment mechanism	16b	Mechanism of implementing the allocation sequence (eg, central telephone; sequentially numbered, opaque, sealed envelopes), describing any steps to conceal the sequence until interventions are assigned	Page 9-10. Line 190-205
Implementation	16c	Who will generate the allocation sequence, who will enrol participants, and who will assign participants to interventions	Page 9-10. Line 190-205
Blinding (masking)	17a	Who will be blinded after assignment to interventions (eg, trial participants, care providers, outcome assessors, data analysts), and how	Page 9-10. Line 190-210

	17b	If blinded, circumstances under which unblinding is permissible, and procedure for revealing a participant's allocated intervention during the trial	Page 10, line 207-208
**Methods: Data collection, management, and analysis**			
Data collection methods	18a	Plans for assessment and collection of outcome, baseline, and other trial data, including any related processes to promote data quality (eg, duplicate measurements, training of assessors) and a description of study instruments (eg, questionnaires, laboratory tests) along with their reliability and validity, if known. Reference to where data collection forms can be found, if not in the protocol	Page 14-15, line 288-327
	18b	Plans to promote participant retention and complete follow-up, including list of any outcome data to be collected for participants who discontinue or deviate from intervention protocols	Page 15-16, line 329-335
Data management	19	Plans for data entry, coding, security, and storage, including any related processes to promote data quality (eg, double data entry; range checks for data values). Reference to where details of data management procedures can be found, if not in the protocol	Page 17, Line 354-361
Statistical methods	20a	Statistical methods for analysing primary and secondary outcomes. Reference to where other details of the statistical analysis plan can be found, if not in the protocol	Page 17-18, Line 362-367
	20b	Methods for any additional analyses (eg, subgroup and adjusted analyses)	Page 17-18, Line 362-396
	20c	Definition of analysis population relating to protocol non-adherence (eg, as randomised analysis), and any statistical methods to handle missing data (eg, multiple imputation)	Page 17, line 338-342
**Methods: Monitoring**			
Data monitoring	21a	Composition of data monitoring committee (DMC); summary of its role and reporting structure; statement of whether it is independent from the sponsor and competing interests; and reference to where further details about its charter can be found, if not in the protocol. Alternatively, an explanation of why a DMC is not needed	Page 19, line 398-411
	21b	Description of any interim analyses and stopping guidelines, including who will have access to these interim results and make the final decision to terminate the trial	Page 14, line 281-286

For peer review only - http://bmjopen.bmj.com/site/about/guidelines.xhtml

Harms	22	Plans for collecting, assessing, reporting, and managing solicited and spontaneously reported adverse events and other unintended effects of trial interventions or trial conduct	Page 13-14, line 278-286
Auditing	23	Frequency and procedures for auditing trial conduct, if any, and whether the process will be independent from investigators and the sponsor	Page 19, Line 398-411
**Ethics and dissemination**			
Research ethics approval	24	Plans for seeking research ethics committee/institutional review board (REC/IRB) approval	Page 20, line 414
Protocol amendments	25	Plans for communicating important protocol modifications (eg, changes to eligibility criteria, outcomes, analyses) to relevant parties (eg, investigators, REC/IRBs, trial participants, trial registries, journals, regulators)	Page 20, line 417-418
Consent or assent	26a	Who will obtain informed consent or assent from potential trial participants or authorised surrogates, and how (see Item 32)	Page 20, line 436-437
	26b	Additional consent provisions for collection and use of participant data and biological specimens in ancillary studies, if applicable	Not applicable
Confidentiality	27	How personal information about potential and enrolled participants will be collected, shared, and maintained in order to protect confidentiality before, during, and after the trial	Page 17, line 354-361
Declaration of interests	28	Financial and other competing interests for principal investigators for the overall trial and each study site	Page 23, line 487-491
Access to data	29	Statement of who will have access to the final trial dataset, and disclosure of contractual agreements that limit such access for investigators	Page 20, line 418-425
Ancillary and post-trial care	30	Provisions, if any, for ancillary and post-trial care, and for compensation to those who suffer harm from trial participation	Page 20, line 437-438
Dissemination policy	31a	Plans for investigators and sponsor to communicate trial results to participants, healthcare professionals, the public, and other relevant groups (eg, via publication, reporting in results databases, or other data sharing arrangements), including any publication restrictions	Page 8 line 154-156 and Page 20, line 427-431

For peer review only

	31b	Authorship eligibility guidelines and any intended use of professional writers	Page 22, line 467-484
	31c	Plans, if any, for granting public access to the full protocol, participant-level dataset, and statistical code	Page 20, line 418-425
**Appendices**			
Informed consent materials	32	Model consent form and other related documentation given to participants and authorised surrogates	Appendix A
Biological specimens	33	Plans for collection, laboratory evaluation, and storage of biological specimens for genetic or molecular analysis in the current trial and for future use in ancillary studies, if applicable	Not applicable

*It is strongly recommended that this checklist be read in conjunction with the SPIRIT 2013 Explanation & Elaboration for important clarification on the items. Amendments to the protocol should be tracked and dated. The SPIRIT checklist is copyrighted by the SPIRIT Group under the Creative Commons "Attribution-NonCommercial-NoDerivs 3.0 Unported" license.

For peer review only
